# An engineered T7 RNA polymerase that produces mRNA free of immunostimulatory byproducts

**DOI:** 10.1038/s41587-022-01525-6

**Published:** 2022-11-10

**Authors:** Athanasios Dousis, Kanchana Ravichandran, Elissa M. Hobert, Melissa J. Moore, Amy E. Rabideau

**Affiliations:** 1 Moderna, Inc., Cambridge, MA USA; 2 Present Address: Tessera Therapeutics, Somerville, MA USA; 3 Present Address: Laronde, Cambridge, MA USA

**Keywords:** Protein design, RNA

## Abstract

In vitro transcription (IVT) is a DNA-templated process for synthesizing long RNA transcripts, including messenger RNA (mRNA). For many research and commercial applications, IVT of mRNA is typically performed using bacteriophage T7 RNA polymerase (T7 RNAP) owing to its ability to produce full-length RNA transcripts with high fidelity; however, T7 RNAP can also produce immunostimulatory byproducts such as double-stranded RNA that can affect protein expression. Such byproducts require complex purification processes, using methods such as reversed-phase high-performance liquid chromatography, to yield safe and effective mRNA-based medicines. To minimize the need for downstream purification processes, we rationally and computationally engineered a double mutant of T7 RNAP that produces substantially less immunostimulatory RNA during IVT compared with wild-type T7 RNAP. The resulting mutant allows for a simplified production process with similar mRNA potency, lower immunostimulatory content and quicker manufacturing time compared with wild-type T7 RNAP. Herein, we describe the computational design and development of this improved T7 RNAP variant.

## Abstract (web summary)

The purity of synthetic mRNA is improved with a double-mutant T7 RNA polymerase.

## Main

mRNA therapeutics are an emerging class of medicines that have several advantages over large-molecule biologics and small-molecule pharmaceuticals. A key advantage from a manufacturing perspective is that mRNA therapeutics can use a single standardized manufacturing process applicable across a range of targets^1,2^. To ensure high mRNA purity for therapeutic applications that are repeat or chronically dosed, several capital-intensive and time-consuming purification steps are required^3^. These additional unit operations often result in increased RNA degradation, decreased quality and/or decreased yield, highlighting the need for a more efficient synthesis process that reduces the impurity burden on the downstream purification steps.

IVT is an enzymatic process used in the synthesis of mRNA products. During IVT, an RNAP is responsible for the transcription of RNA from a DNA template. Although IVT is a recognized process in research laboratories, optimization for industrial-scale production has only recently begun^4–10^.

**Fig. 1:**
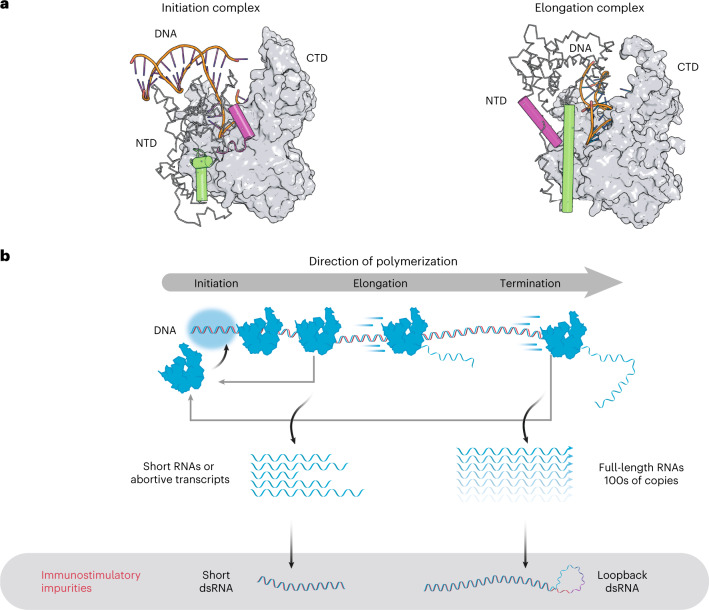
T7 RNAP transcription cycle. **a**, T7 RNAP takes on two distinct conformational states during catalysis, the IC and EC. **b**, In the IC, T7 RNAP binds the T7 promoter and generates short RNAs or abortive transcripts (2–10 nucleotides) until it transitions into the EC; this transition may require several initiations before it is successful. The short RNAs produced can interact with T7 RNAP via RNA-templated transcription, producing short dsRNAs. Only after transitioning into the EC is T7 RNAP highly processive and capable of generating full-length RNA. As an enzyme, T7 RNAP can catalyze the formation of hundreds of copies of full-length RNA. These full-length molecules can also be used as templates by T7 RNAP to generate long loopback dsRNA species. Both types of dsRNA impurities are immunostimulatory in vitro and in vivo.

The most commonly used polymerase for IVT is T7 RNAP, a 99-kDa enzyme comprising an amino-terminal domain (NTD; residues 1–266, Fig. 1 ribbon) and a carboxy-terminal domain (CTD; residues 267–883, Fig. 1 grey surface). Its popularity in research and commercial development is due to its applicability for in vitro RNA production, its high yield and the high fidelity of the transcripts produced^11,12^. However, T7 RNAP also creates multiple byproducts, including immunostimulatory double-stranded RNAs (dsRNAs) that arise from product-templated transcription; these can affect potency and safety, particularly in therapeutic applications^3^.

The production of dsRNA and other byproducts during IVT is influenced by the conformation of T7 RNAP during the catalytic cycle. T7 RNAP-mediated transcription encompasses three distinct stages: initiation, elongation and termination^12–14^. During initiation, the NTD of T7 RNAP binds to the promoter sequence, forming the initiation complex (IC; Fig. 1a)^12,15,16^. This IC is unstable and produces short RNA transcripts known as abortive transcripts of 2–10 nucleotides in length, in a process called abortive cycling (Fig. 1b)^16^. Once the transcript length is greater than ~10 nucleotides, the promoter-binding residues in the NTD rearrange, releasing the DNA promoter region and forming a stable and processive enzyme elongation complex (EC; Fig. 1a)^17–19^. Termination occurs either in response to specific signal sequences or upon reaching the end of a linearized DNA template, a process known as ‘run-off transcription’, which typically yields full-length RNA but sometimes results in nontemplated additions to the 3′ end (Fig. 1b)^20–24^. T7 RNAP also has cryptic RNA-templated transcription capabilities, resulting in the formation of dsRNA products and loopback dsRNA products (Fig. 1b)^25–27^. The exact mechanisms behind 3′ heterogeneity and loopback transcription remain unknown. Thus, devising rational approaches for preventing either activity is challenging.

It has been well established that dsRNA molecules are innate immune response activators capable of triggering Toll-like receptor 3 in the endosome and retinoic acid gene-I-like receptors such as RIG-I, MDA5 and LGP2 in the cytoplasm^28–30^. Consequently, such dsRNA molecules can affect the potency and safety of the mRNA product, necessitating additional purification steps to remove dsRNA from the final mRNA product. Two methods are commonly used during manufacturing to reduce the dsRNA burden. One is purification of mRNA products using chromatography, such as reversed-phase high-performance liquid chromatography (RP-HPLC) or cellulose-based isolation of dsRNA; the other is modification of the IVT conditions to decrease byproduct formation^3,6,31^. Although effective in reducing dsRNA burden, modifying IVT conditions is not sufficient to eliminate the need for downstream purification of mRNA products for therapeutic applications by RP-HPLC, which is a costly and time-consuming step^6^.

Herein, we report the identification of a double-mutant T7 RNAP (G47A + 884G) that dramatically reduces dsRNA IVT byproducts while also maintaining RNA yield and purity, thereby reducing the burden on downstream purification processes to control transcription-related dsRNA impurities.

## Results

### Engineering the C-terminal ‘foot’ of T7 RNAP to decrease dsRNA formation

**Fig. 2:**
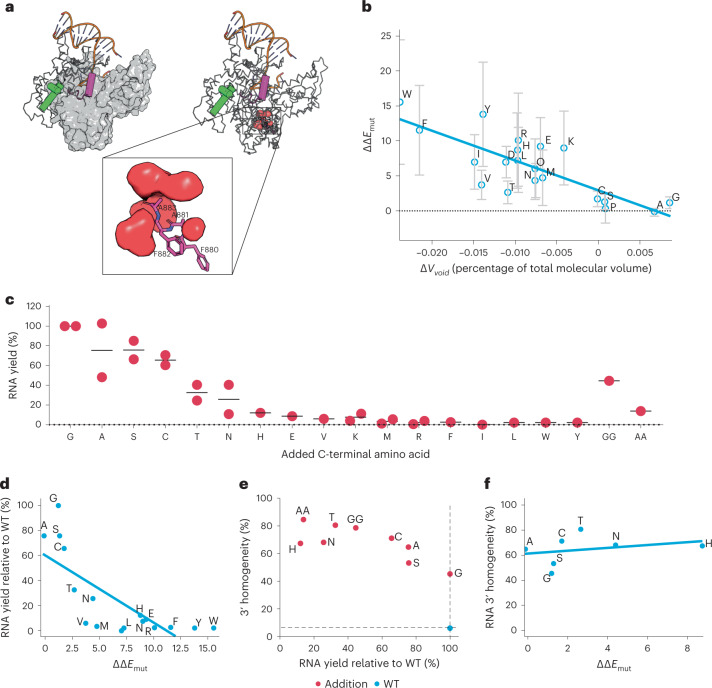
C-terminal ‘foot’ engineering to decrease dsRNA impurities. **a**, Cutaway of T7 RNAP indicating the buried C-terminal ‘foot’ (FAFA^883^; purple) and associated void volume (red). The CTD (grey surface), bound DNA template (orange) and targeted helices (purple and green cylinders) in the NTD (ribbon) are shown for reference. **b**, Scatter plot of change in void volume (∆*V*_void_; as a percent of total molecular volume) compared with the change in ∆∆*E*_mut_ due to C-terminal substitutions relative to 884A. The line indicates the best-fit linear regression (plotted with Seaborn.lmplot), with a Pearson correlation of −79% (calculated with the scipy.stats Python library); data are presented as mean ± s.d.; *n* = 25 for ∆∆*E*_mut_; error bars for ∆*V*_void_ are excluded for clarity, *n* = 120. **c**, RNA yield as a function of C-terminal foot substitution. The *x* axis represents the identity of the added amino acid in single-letter code. Data indicate that yield generally decreases as a function of amino acid size; *n* = 2. **d**, Scatter plot of ∆∆*E*_mut_ versus RNA yield relative to WT. The line indicates the best-fit linear regression, with a Pearson correlation of −76%; data are presented as mean for ∆∆*E*_mut_, *n* = 25; *n* = 1 for RNA yield. **e**, Scatter plot of 3′ homogeneity compared with total RNA yield of selected mutants; *n* = 1. WT T7 RNAP is represented by the dotted lines and blue circle. Here, 884G (shown as G) was selected owing to improved 3′ homogeneity while maintaining RNA yield. The 3′ homogeneity generally improved with increasing amino acid size. **f**, Scatter plot of ∆∆*E*_mut_ versus RNA 3′ homogeneity. The line indicates the best-fit linear regression, with a Pearson correlation of 29%; data are presented as mean for ∆∆*E*_mut_, *n* = 25; *n* = 1 for RNA 3′ homogeneity. Source data

The C-terminal Phe-Ala-Phe-Ala^883^ (FAFA^883^) or ‘foot’ of the enzyme is situated within the CTD. Residues in the foot reside in proximity to the magnesium-ion-coordinating residues within the active site; when mutated, these residues have been shown to influence initiation rates and elongation^32–34^. Structural analysis revealed a small solvent-inaccessible cavity adjacent to the C terminus that allows for the insertion of additional amino acid residues (Fig. 2a). This cavity was computationally probed for structural compatibility of foot insertions.

Modeling of five crystal structures of T7 RNAP in the IC, the EC and an intermediate state (Protein Data Bank (PDB) identifiers: 1CEZ, 2PI4, 1MSW, 1S76, 3E2E) were used to introduce mutations and estimate the change in folding free energy due to each mutation (∆∆*G*_mut_; using Rosetta software^35^). The change in void volume (∆*V*_void_) for each of the mutant structure models was also calculated (using ProteinVolume software^36^). Our findings indicate a positive correlation between ∆*V*_void_ and the free energy change calculated through Rosetta (∆∆*E*_mut_; reported as Rosetta Energy Units and distinguishable from ∆∆*G*_mut_; Fig. 2b). Charged residues (for example, Arg, Lys, Glu) are the exception to this phenomenon, as electrostatic interactions are factored into ∆∆*E*_mut_ but not ∆*V*_void_.

Various mutant T7 RNAP constructs were generated by randomizing amino acid residues at position 884 using PCR and NNK degenerate primers. For each PCR, plasmids were isolated from 96 transformed *Escherichia coli* colonies (using standard chemical transformation) and analyzed via Sanger sequencing; 17 of the desired 20 substitutions were identified by this process. Two additional double mutants, 884G + 885G (GG) and 884A + 885A (AA), were generated using site-directed mutagenesis. The resulting recombinant proteins were expressed, purified and characterized via standard IVT reactions. Mutants with smaller residues (for example, Gly, Ala, Ser and Cys) maintained RNA yields comparable with those of the wild-type (WT) T7 RNAP (Fig. 2c). However, RNA yield decreased when larger (for example, Phe, Trp, Tyr, Lys or Glu) or multiple (GG and AA) residues were incorporated. RNA yield was correlated negatively with increased (less favorable) ∆∆E_mut_ (Fig. 2d), indicating that T7 RNAP is sensitive to structural changes near the foot. Mutants that incorporated Glu, Val, Lys, Met, Arg, Phe, Ile, Leu, Trp or Tyr were excluded from further analysis owing to low RNA yields.

RNA generated by the resulting T7 RNAP mutants was subjected to RNase T1 digestion, which can be used to monitor run-off or run-on transcription based on the presence of a 3′-hydroxide or 3′-phosphate on the templated terminal guanosine, respectively^37^. The presence of a 3′-hydroxide on the 3′-templated terminal guanosine indicates proper run-off transcription or 3′ homogeneity^37^. All nine mutants selected for continuation showed increased 3′ homogeneity (45–85%) relative to WT T7 RNAP (6%; Fig. 2e). No strong correlation between 3′ homogeneity and ∆∆*E*_mut_ (Fig. 2f) was observed.

As RNA yield is a crucial cost driver for large-scale mRNA manufacturing, the 884G mutant (45% 3′ homogeneity and 100% RNA yield relative to WT) was selected for further analysis.

### Engineering the NTD of T7 RNAP to further decrease dsRNA impurities

The formation of short RNA or abortive transcripts, which decreases RNA yields and purity, is commonly associated with the IC. Mutations predicted to stabilize the highly processive EC mode were evaluated with the hypothesis that a smoother transition from the IC to the EC would decrease byproduct formation during IVT.

In engineering T7 RNAP to reduce dsRNA impurities while maintaining RNA purity and yield, a major constraint is the labor-intensity of the characterization experiments, which entail multiple analytical assays requiring individually purified mutants. To focus our screening, we therefore used structure-guided rational design combined with a reduced alphabet of amino acids^38–40^. This reduced alphabet captures important physicochemical amino acid sidechain properties using single representatives of the following classes: small (Ala) and large (Leu) hydrophobic, positively (Arg) and negatively charged (Glu), and aromatic (Trp).

**Fig. 3:**
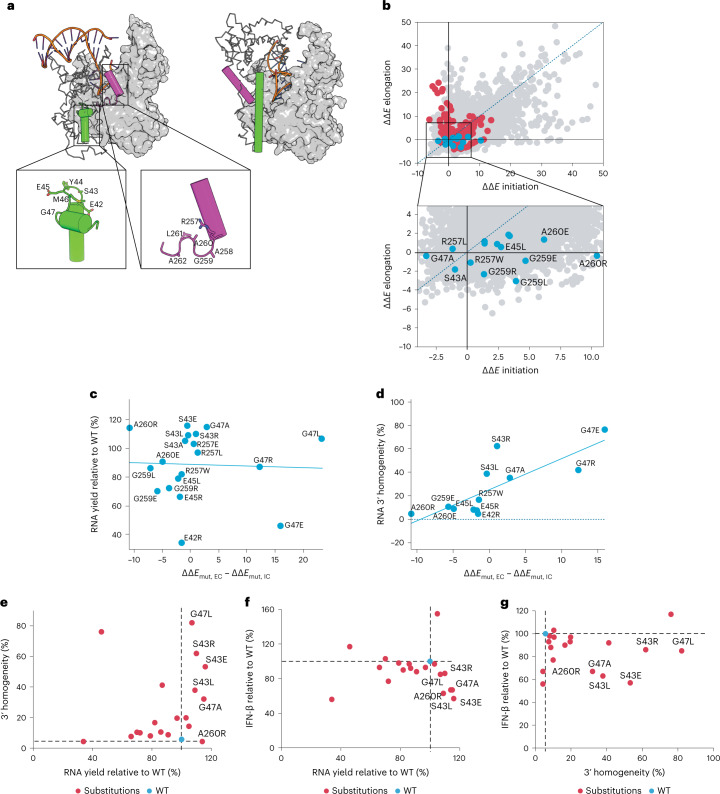
NTD engineered mutants for decreased dsRNA impurities. **a**, Conformational change in the C-helix (green) and C-linker (purple) from the IC (PDB: 2PI4; left) to the EC (PBD: 1H38; right) for T7 RNAP. **b**, Computational prediction of EC-favoring substitutions using Rosetta. Any point below the dotted blue line represents a substitution for which the calculated ∆∆*E*_mut_ (reported as Rosetta Energy Units and distinguishable from ∆∆*G*_mut_) was more favorable for the EC than the IC. ∆∆*E*_mut_ values for WT and all 19 possible substitutions at all 878 modeled positions within the T7 RNAP IC and EC crystal structures are plotted as grey dots (20 × 878 = 17560 total points). The C-helix and C-linker substitutions are shown as red dots (20 × 14 = 280 points), with blue dots representing 20 substitutions selected for experimental characterization. **c**, Scatter plot of 𝚪 (ΔΔ*E*_mut,EC_ − ΔΔE_mut,IC_) compared with RNA yield (percentage relative to WT) for NTD substitutions; the blue line represents the line of best fit; *n* = 1. **d**, Scatter plot of 𝚪 versus 3′ homogeneity for the NTD substitutions; Pearson correlation of 80%; *n* = 1. **e**, Scatter plot of RNA 3′ homogeneity compared with RNA yield for the NTD substitutions. **f**, Scatter plot of IFN-β response compared with RNA yield for the NTD mutants; *n* = 2. **g**, Scatter plot of IFN-β response compared with RNA 3′ homogeneity for the NTD mutants; *n* = 1. In **e**–**f**, black dotted lines and blue circle indicate WT T7 RNAP. Red dots represent all mutants; mutants with desired outcomes as compared to WT T7 RNAP are labeled with their identity. Source data

As T7 RNAP undergoes conformational transition from IC to EC, the NTD structurally rearranges to release the DNA promoter, and two helix-turn-helix motifs in the IC switch to form single helices (Fig. 3a). The loop (residues 42–47) in the first IC helix-turn-helix changes to form the C-helix (residues 28–55) in the EC, and transformation of a second loop (residues 258–265) likewise converts the C-linker region (residues 251–296) into a helix (Fig. 3a)^41–43^. Helix-favoring substitutions were introduced in the IC loops of the C-helix and C-linker under the hypothesis that this would facilitate the transition from the IC to EC.

Of the 20 common amino acids, Ala is considered to have the highest helix propensity and Gly the lowest^44^; consequently, G47A and G259A were selected as primary candidates. Additional substitutions from a reduced alphabet were considered at positions in the C-helix and C-linker. These substitutions were filtered and further rationalized using the calculated mean ∆∆*E*_mut_ for IC (PDB: 1QLN) and EC (PDB: 1MSW) T7 RNAP crystal structures (Fig. 3b and Supplementary Fig. 1).

A subset of 20 T7 RNAP substitutions were selected to generate RNA by IVT. The IVT reactions were evaluated for yield (Fig. 3c), 3′ homogeneity (Fig. 3d) and influence on innate immune response via interferon-β (IFN-β) response (Fig. 3f and Supplementary Fig. 2). No correlation between EC-favoring mutations (∆∆*E*_mut,EC_ − ∆∆*E*_mut,IC_ < 0) and RNA yield was observed (Fig. 3c); however, an inverse correlation was evident between EC-favoring mutations and 3′ homogeneity (Fig. 3d).

Of the mutants characterized, five (S43L, S43E, S43R, G47A and G47L) were found to have increased 3′ homogeneity (Fig. 3e), decreased IFN-β response (Fig. 3f) and increased RNA yields relative to WT T7 RNAP (Fig. 3e,f); IFN-β response decreased by more than 30% for RNA produced by S43L, S43E and G47A mutations (Fig. 3f). Only one substitution, A260R, decreased IFN-β response by ~33% with minimal effect on 3′ homogeneity. The majority of mutants that showed improved 3′ homogeneity and lower IFN-β responses had C-helix loop modifications.

Three mutants (S43L, S43E and G47A) were selected based on increased 3′ homogeneity and decreased IFN-β responses (Fig. 3g), both of which are favorable properties for manufacturing mRNA therapeutics. The combination of G47A and 884G was selected for further analysis based on its robust performance across mRNAs of different lengths, as described in the next section.

### mRNA produced by the G47A + 884G T7 RNAP double mutant eliminates dsRNA and cytokine production in vitro

**Fig. 4:**
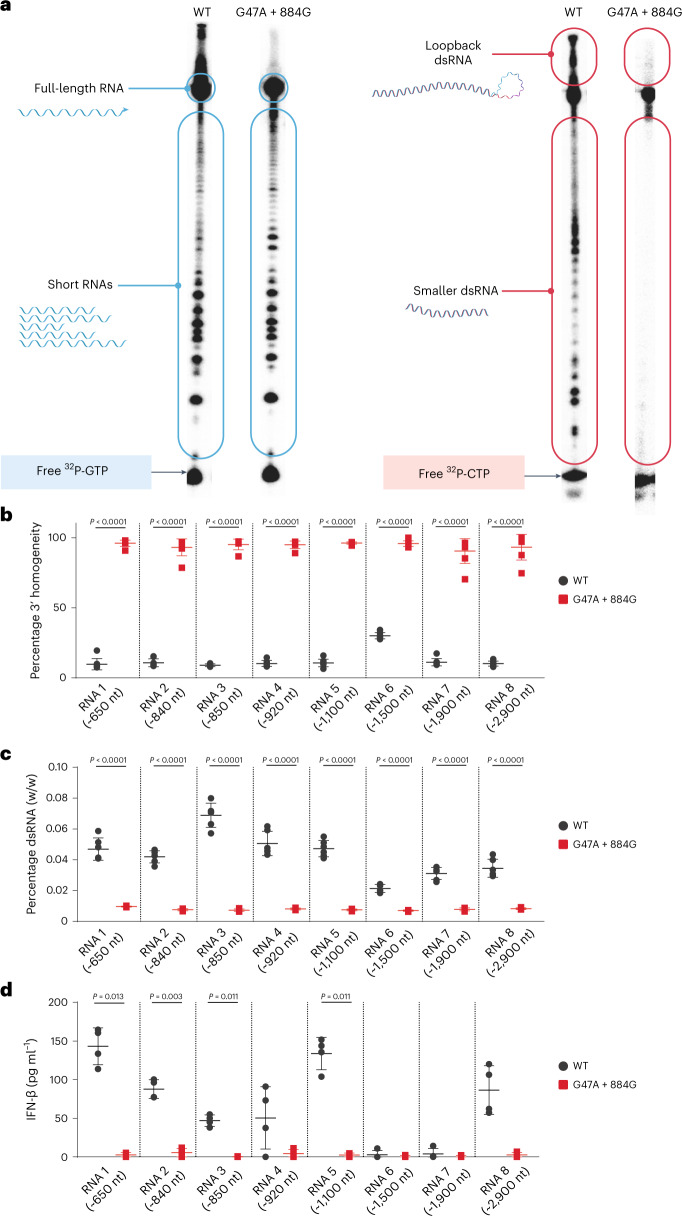
Comparison of IVT impurities between WT and G47A + 884G T7 RNAPs. **a**, Radioactive sequencing gels monitoring the formation of short RNA species and dsRNA by WT T7 RNAP and G47A + 884G T7 RNAP. Reactions using α-^32^P-GTP selectively label abortive species, full-length RNA, run-on transcripts and large RNA species generated from RNA-templated and loopback transcription (blue panels). By contrast, reactions incorporating α-^32^P-CTP label short and long dsRNA species in addition to full-length and run-on transcripts (red panels); *n* = 3. Full images of the radioactive gels and images from trials 2 and 3 are shown in Supplementary Fig. 3. **b**–**d**, 3′-homogeneity (*n* = 8 mRNA lots) (**b**), dsRNA levels (*n* = 6 mRNA lots) (**c**) and IFN-β responses (*n* = 4 mRNA lots) (**d**) between WT (black) and G47A + 884G (red) across eight different mRNAs of varying length and sequence composition. Data are presented as mean ± s.d. Statistical significance was determined using two-sided Brown–Forsythe analysis of variance followed by Dunnett’s multiple comparison test for hypothesis testing. Family-wise correction was used for multiple testing correction with alpha level of 0.05. nt, nucleotides. Source data

When G47A + 884G was compared with WT T7 RNAP using radioactive denaturing polyacrylamide gel electrophoresis, a substantial decrease in dsRNA was observed in vitro (Fig. 4a). The template used in these reactions is guanosine-rich, with the first instance of cytosine at position 43. This allows the polymerase to transition from IC to EC before incorporation of the first cytosine into the product. Thus, reactions using α-^32^P-GTP selectively label abortive species (that is, RNAs <20 nucleotides, Fig. 1), full-length RNA and any run-on or loopback transcripts. By contrast, reverse complements are cytosine-heavy and labeled by α-^32^P-CTP. α-^32^P-CTP also labels any product that contains position 43 and beyond (that is, both full-length RNA and loopback transcripts). As shown in Fig. 4a, both WT and mutant T7 RNAP produced similar abortive transcript profiles (blue panels), but the amounts of short and long dsRNA species were below the detection limit of the assay for G47A + 884G (red panels). These observations were confirmed by two additional independent repeats of the experiments (Supplementary Fig. 3).

The absence of loopback transcription was further confirmed across eight different mRNAs varying in length and sequence composition by RNaseT1 assay (Fig. 4b), with G47A + 884G producing significantly more (*P* < 0.0001) homogeneous mRNA compared with WT T7 RNAP. Combination of the G47A and 884G mutations increased 3′ homogeneity more than two-fold compared with the 3′ homogeneities of 32% and 45% observed separately for G47A and 884G, respectively (Figs. 2e and 3e). Total dsRNA levels were significantly lower with G47A + 884G than WT T7 (*P* < 0.0001) regardless of mRNA length or sequence composition (Fig. 4c). Four of eight mRNA constructs synthesized with G47A + 884G also showed statistically significant decreases in IFN-β levels (*P* < 0.05; Fig. 4d). We hypothesize that the lack of differences within the remaining constructs might be due to a combination of assay sensitivity and mRNA sequence-specific effects. Yield and purity of all eight mRNAs were comparable between WT and G47A + 884G (Supplementary Fig. 4).

### G47A + 884G eliminates the need for downstream RP-HPLC purification

**Fig. 5:**
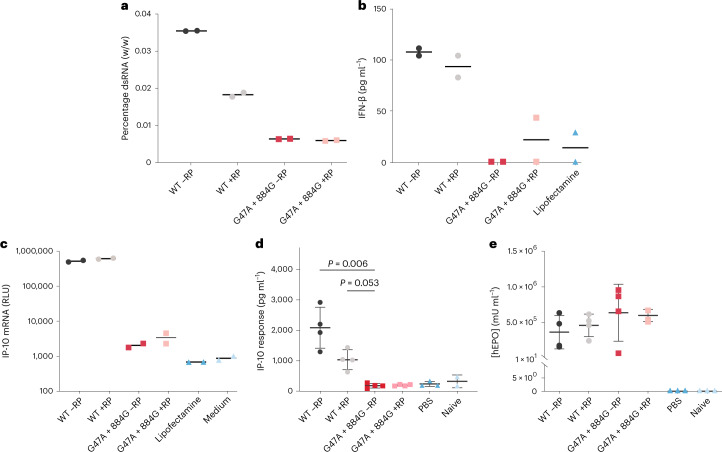
In vitro and in vivo performance of mRNAs synthesized by WT and G47A + 884G T7 RNAPs. **a**, Quantification of dsRNA using ELISA indicated that the RNAs produced by G47A + 884G had lower dsRNA content than WT T7 RNAP, whether purified by RP chromatography or not (all mRNAs were purified by oligo-dT affinity chromatography); *n* = 1 mRNA, *n* = 2 technical replicates. **b**, IFN-β response in BJ fibroblasts indicates that RNAs produced by G47A + 884G with (+RP) or without RP chromatography have baseline response, similar to the Lipofectamine control; *n* = 1 mRNA, *n* = 2 technical replicates. **c**, IP-10 response measured in MDMs confirmed that G47A + 884G generates immune-silent mRNA; *n* = 1 mRNA, *n* = 2 technical replicates. **d**, IP-10 response was measured in serum 6 h after mice were dosed intravenously with mRNA generated with WT T7 RNAP or G47A + 884G with or without RP chromatography. IP-10 response in vivo confirmed in vitro observations that G47A + 884G generates mRNA that does not stimulate a detectible innate immune response in vivo. Data are presented as mean ± s.d.; *n* = 4 mice for WT T7 RNAP and mutant, *n* = 3 mice for PBS and naive. Statistical significance was determined using Kruskal–Wallis testing followed by Dunn’s multiple comparison test for hypothesis testing. Family-wise correction was used for multiple testing correction with an alpha level of 0.05. **e**, hEPO expression was measured in serum 6 h after mice were dosed intravenously with mRNA synthesized with WT or G47A + 884G with or without RP chromatography. All mRNAs were expressed equally. Data are presented as mean ± s.d; *n* = 4 mice for WT T7 RNAP and mutant, *n* = 3 mice for PBS and naive. Source data

mRNA products synthesized by G47A + 884G were compared with those generated by WT T7 RNAP to assess the need for RP-HPLC in the downstream purification process. mRNAs coding for human erythropoietin (hEPO) were generated by G47A + 884G and WT T7 RNAP, followed by poly-deoxythymidine (dT) affinity chromatography, then optionally purified by RP-HPLC. The mRNA products synthesized by WT T7 RNAP had higher dsRNA burden (Fig. 5a) and higher cytokine generation (Fig. 5b) compared with those synthesized by G47A + 884G, regardless of the purification method used. In addition, purification of G47A + 884G mRNA products did not further reduce the already low dsRNA content or IFN-β response. This response was confirmed using a monocyte-derived macrophage (MDM) assay measuring IP-10 (Fig. 5c), which showed similar trends to the BJ fibroblast assay measuring IFN-β.

In vivo analysis of hEPO mRNA was used to evaluate levels of serum cytokines and chemokines, including IFN-α, IFN-γ, interleukin (IL)-12p70, IL-6, IFN-γ inducible protein (IP)-10, monocyte chemoattractant protein-1, macrophage inflammatory protein-1β, RANTES and tumor necrosis factor-α (TNF-α). The in vivo IP-10 responses (Fig. 5d) corroborated the results of the in vitro IP-10 assay (Fig. 5c), with statistically significant differences (*P* = 0.006) between G47A + 884G and WT in the absence of RP purification. No significant responses were observed for a panel of other cytokines tested (Supplementary Fig. 5). hEPO protein expression was comparable in vivo between mRNA products synthesized with WT T7 RNAP and G47A + 884G (Fig. 5e). These results confirm that G47A + 884G produces mRNA of high potency and low cytokine response in the absence of RP purification.

## Discussion

Currently, the production of mRNA products with low innate immune stimulation suitable for therapeutic applications requires stringent downstream purification, typically involving RP-HPLC^3^. Previous studies have shown that immunostimulatory byproducts can be reduced by altering nucleoside triphosphate (NTP) ratios during transcription^6^. The current investigation demonstrates that production of dsRNA byproducts can also be significantly decreased by engineering a mutant T7 RNAP using a combination of structural, mechanistic, computational and laboratory screening approaches. The resulting G47A + 884G T7 RNAP mutant largely eliminated immunostimulatory impurities during IVT, indicating the potential for substantially streamlining mRNA manufacturing processes.

Other groups have also reported T7 RNAP protein engineering to control impurity formation during IVT. Wu et al.^9^ reported that thermostable mutants of T7 RNAP modulated IVT impurity profiles while maintaining yields when transcription reactions were performed at higher temperatures (>48 °C). They proposed that altering the reaction temperature could prevent loopback transcription by preventing the enzyme from rebinding RNA transcripts. Although this study offered an elegant solution to the problem of controlling product-related immune responses, high-temperature IVTs are difficult to scale and may cause RNA degradation.

Work by the Coleman laboratory exploring mutagenesis of the FAFA^883^ foot to AAFA^883^ indicated the importance of the CTD in improving RNA purity^34^. Although only a single T7 RNAP mutant that improved 3′ homogeneity was identified in that study, our study identified 11 T7 RNAP variants with C-terminal additions that decreased byproduct formation by 7–15-fold compared with WT. Among the mutants identified in our studies, the added steric bulk in the foot may disfavor an active or processive conformation, reducing the affinity for poor substrates such as RNA templates. A positive correlation between the size of the added amino acid and increased 3′ homogeneity accompanied by a negative correlation between size and enzyme productivity, as measured by RNA yields, suggests a trade-off between these parameters (Fig. 2). As specificity is closely linked to enzyme processivity, it is highly unlikely that RNA-templated transcription and abortive cycling can be eliminated without severely compromising DNA-templated RNAP activity.

A second engineering strategy crucial for this study involved amino acid substitutions designed to affect the IC-to-EC transition. The P266L mutation was identified previously by random mutagenesis as a variant that substantially decreases the production of short RNAs by facilitating promoter clearance^43,45^. P266L also shifted the abortive RNA profile from short (4–7 nucleotides) to longer (9–11 nucleotides) sequences by stabilizing the IC when the transcript was 5–8 nucleotides long^43,45^. These observations suggested that lowering the IC-to-EC transition barrier could reduce the production of short RNAs, which might decrease dsRNA byproducts. Analysis of RNA products generated by 20 putative EC-stabilizing mutants identified several T7 RNAP variants that substantially decreased dsRNA levels (Fig. 3). G47A, which was predicted to be a helix-stabilizing variant favoring the EC conformation, emerged as a prime candidate owing to improved 3′ homogeneity (~20%) and decreased IFN-β response (~30%). Mu et al.^46^ hypothesized that undesirable promoter-independent transcription might start in the EC conformation and that stabilizing IC relative to EC might thus be more advantageous for reducing dsRNA formation than the original EC-stabilizing design rationale of this study. The ∆∆*E*_mut_ of G47A suggested that it might be slightly IC-favoring, in contrast to the strongly helix-favoring propensity of this substitution. Termination by T7 RNAP has also been hypothesized to involve reversion of the polymerase from EC to IC^22^; the observation that G47A improves mRNA 3′ homogeneity may also indicate that this mutant favors IC. Studies identifying the IC:EC equilibrium for the G47A variant are ongoing.

In this work, G47A and 884G individually had a modest impact on 3′ homogeneity of the resulting RNA products (Figs. 2e and 3e); each mutation increased 3′ homogeneity from 6–12% (as seen with WT T7 RNAP) to 30–45% and decreased IFN-β production by ~50% compared with WT T7 RNAP. When the G47A and 884G mutations were combined, an additive effect was observed with >90% 3′ homogeneity, reduced dsRNA content and low immunostimulatory responses detected by in vitro IFN-β induction across multiple sequence lengths and sequence compositions (Fig. 4b–d). Radioactive sequencing results for ^32^P-GTP-labeled products generated by G47A + 884G versus WT T7 RNAP (Fig. 4a) showed minimal differences in amounts and composition of the abortive transcripts. This could indicate that G47A and 884G have opposing effects on abortive transcript production, where one mutation increases and the other mutation decreases abortives, or that G47A does not stabilize EC (as hypothesized above) or, finally, that EC stabilization alone does not decrease the production of short RNAs. Regardless, G47A + 884G was not capable of abrogating abortive short RNA formation. However, G47A + 884G could decrease both short and loopback dsRNA populations (Fig. 4a) below levels of detection. A potential explanation could be that G47A + 884G maintains high affinity for the DNA template but has substantially decreased affinity (or a very high *K*_m_) for RNA templates; additional studies are needed to confirm this hypothesis. Whether the individual substitutions G47A and 884G operate by similar or distinct mechanisms remains unknown. The repeated observation that 884G generates slightly more homogeneous RNA than G47A while providing similar reductions in cytokine responses suggests that the two mutations may have different yet synergistic mechanisms of action.

In summary, this study demonstrates that T7 RNAP can be modified to decrease immunostimulatory byproduct formation. This discovery opens the door to streamlining the development of processes for therapeutic mRNA production, improving cost-effectiveness and generating mRNA-based products with suitable attributes for clinical or commercial use.

## Methods

### Materials

Synthetic oligonucleotides were obtained from Integrated DNA Technologies. NTP solutions were acquired from Hongene. α-^32^P-GTP and α-^32^P-CTP were purchased from Perkin Elmer. Pyrophosphatase was purchased from New England Biolabs. Plasmids were transformed into BL21 chemically competent *E. coli* cells. WT and mutant T7 RNAP were overexpressed as N-terminal hexahistidine-tagged variants and purified by Ni-NTA affinity chromatography. C-terminal foot substitutions were generated by PCR using NNK degenerate primers. A total of 96 bacterial colonies were Sanger-sequenced to identify 17 of the 20 desired substitutions.

### Computational structure preparation

Crystal structures of T7 RNAP in the IC (PDB: 1CEZ, 2PI4, 1QLN), the EC (PDB: 1MSW, 1H38, 1S76) and an intermediate state (PDB: 3E2E) were computationally prepared by removing water, ions and nucleic acids and then rebuilding missing loops and sidechains using the comparative modeling protocol RosettaCM (flags in Supplementary Tables 1–4)^47^. DNA and RNA were reintroduced into the rebuilt protein structures by aligning the protein in the original complex with the rebuilt lone protein and then copying the coordinates of the nucleic acids into the rebuilt structure. The resulting DNA–(RNA)–protein complexes were subsequently minimized with heavy-atom crystallographic constraints using the Rosetta constrained minimization protocol (flags in Supplementary Table 5). These minimized models were assessed for quality by checking root mean square deviation from the reference crystal structures and filtering out models with any steric clashes or other residue-level abnormalities highlighted by the Rosetta score function.

### Computational modeling of folding free energy change

Fixed-backbone folding free energies of mutation changes (∆∆*E*_mut_; in Rosetta Energy Units and distinguishable from ∆∆G_mut_) were calculated using the Rosetta macromolecular modeling suite^35,48^. Structures were first prepared using the structure preparation protocol discussed earlier. Next, all possible NTD single substitutions in the prepared IC and EC structures were scanned using the Rosetta ddg_monomer protocol (flags in Supplementary Table 6, with example table provided as Supplementary Table 7)^48^. Results were tabulated and summarized using the Python Pandas package. Secondary structures of IC and EC structures were determined from the PDB secondary structure records and by DSSP^49^.

### Computational modeling of protein cavities

Changes in protein void volume due to C-terminal foot insertions were calculated using the ProteinVolume software tool^36^. First, insertion mutants were modeled by building structures with the Ala insertion (884A) using RosettaCM (as described in ‘Computational structure preparation’ but with an 884-mer insertion sequence instead of the 883-mer native sequence) and then using Rosetta’s ddg_monomer protocol to introduce the other 19 residue types at position 884. Twenty-four 884A models were generated from a set of five parental T7 RNAP crystal structures representing the IC (PDB: 1CEZ, 2PI4), the EC (PDB:, 1MSW, 1S76) and an intermediate state (PDB: 3E2E). These 24 884A models served as starting points for a second ddg_monomer stage, in which the ddg_monomer protocol yielded five structure models for each insertion mutant (including A884A), resulting in a total of 2,400 structure models (24 starting structures × 20 mutants × 5 structures per mutant). The solvent-excluded, van der Waals and void volumes for each of these 2,400 structures were calculated using ProteinVolume v.1.3 with default parameters (starting probe size 0.8, ending probe size 0.2 and surface minimum distance 0.1).

### Statistics

The Pearson correlation coefficient was calculated using the pearsonr function from the Python package scipy.stats. This correlation coefficient is calculated as:

$$r = \frac{{\mathop {\sum }\nolimits_{i = 1}^n \left( {x_i - m_x} \right)\left( {y_i - m_y} \right)}}{{\sqrt {\mathop {\sum }\nolimits_{i = 1}^n \left( {x_i - m_x} \right)^2\mathop {\sum }\nolimits_{i = 1}^n \left( {y_i - m_y} \right)^2} }}$$

where *m*_*x*_ is the mean of vector ${{{\boldsymbol{x}}}} = \{ x_1 \ldots x_n\}$, *m*_*y*_ is the mean of vector ${{{\boldsymbol{y}}}} = \{ y_1 \ldots y_n\}$ and *n* is the sample size.

### mRNA synthesis and characterization

mRNA was synthesized in vitro by T7 RNAP-mediated transcription at 37 °C using 100% substituted N1-methylpseudouridine-triphosphate and a linearized DNA template, which incorporated the 5′ and 3′ untranslated regions and a polyadenosine tail. Reactions with WT and mutant enzymes were treated similarly. NTPs were included at equimolar concentrations. After transcription, the Cap 1 structure was added to the 5′ end using Vaccinia capping enzyme (New England Biolabs) and Vaccinia 2′O-methyltransferase (New England Biolabs). The mRNA was purified by oligo-dT affinity purification. For mRNAs described as ‘with RP’, ion-paired reversed-phase (RP) chromatography was subsequently used for purification. All mRNAs were buffer exchanged by tangential flow filtration into sodium citrate, pH 6.5, and sterile filtered. The mRNA was kept frozen at –20 °C until further use.

### LNP production and characterization

The mRNA was encapsulated in a lipid nanoparticle through a modified ethanol-drop nanoprecipitation process described previously^50^. Briefly, ionizable, structural, helper and PEG lipids were mixed with mRNA in acetate buffer, pH 5.0, at a ratio of 3:1 (lipids/mRNA). The mixture was neutralized with Tris-Cl, pH 7.5, sucrose was added as a cryoprotectant and the final solution was sterile filtered. Vials were filled with formulated LNP and stored frozen at –70 °C until further use. The final drug product underwent analytical characterization, which included the determination of particle size and polydispersity, encapsulation, mRNA purity, osmolality, pH, endotoxin and bioburden, and the material was deemed acceptable for in vivo study.

### RNA yields

Yields of purified RNA were measured by ultraviolet spectrophotometry at 260 nm. A standard extinction coefficient for RNA of 40 ng-cm µl^−1^ was used to determine concentration.

### Radioactive-gel-based analysis of IVT products

The formation of short RNAs or abortive transcripts and dsRNA products was assessed using IVT in combination with ^32^P-labeled NTPs. In vitro transcription reactions were performed in a total volume of 50 µl containing 400 nM WT or mutant T7 RNAP; 7.5 mM each of ATP, CTP, GTP and UTP; 0.5 µCi µl^−1^ α-^32^P-GTP (to label abortive species) or α-^32^P-CTP (to label reverse-complement species); 100 U ml^−1^ pyrophosphatase and 1.8 µM dsDNA template in 1× IVT buffer (New England Biolabs). The dsDNA template was generated by annealing synthetic DNA sequences containing the T7 promoter (5′-TAATACGACTCACTATAGGGAAATAAGAGAGAAAAGAAGAGTAAGAAGAAATATAAGAGCCACCAAAAAAAAAAAAAAAAAAAATCTAG-3′) in 1× phosphate-buffered saline (PBS). This template has a G-rich initiation sequence of 42 nucleotides, enhancing T7 RNAP EC transition, before the first incorporation of CTP at position 43. Thus, reactions using α-^32^P-GTP selectively label abortive species, full-length RNA, run-on transcripts and large RNA species generated from loopback transcription. By contrast, reactions incorporating α-^32^P-CTP label selectively labeled reverse complements, full-length RNA, run-on and loopback products. IVT reactions were incubated for 2 h at 37 °C and quenched by addition of 80 mM EDTA. RNA was isolated by ethanol precipitation, and pellets were resuspended in 1× RNA loading dye. Samples were briefly heated (70 °C for 1 min) and loaded onto 20% acrylamide, 6 M urea gels and electrophoresed for 30 min at 20 W followed by 2 h at 40 W. Gels were exposed to phosphor screens (1 h to overnight) and imaged using a Typhoon FLA9500 Biomolecular Imager. The gels were viewed under two contrast levels: low contrast levels were used to compare full-length mRNA yields, and high contrast levels were used to determine the impurity profile (original gels are shown in Supplementary Fig. 3). The radioactive assay offers the advantage of high sensitivity and the ability to label all IVT impurities.

### RNase T1 digest assay

To allow for high-throughput, quantitative analysis of run-on and loopback transcripts, an RNase T1-based assay was used as described previously by Jiang et al.^37^. RNase T1 selectively cleaves single-stranded RNA after guanosine residues, which facilitates the identification of run-on transcripts when using DNA templates encoding terminal 3′ guanosine. RNase T1 treatment does not affect run-off transcripts, allowing them to maintain their 3′-hydroxide; however, in the presence of run-on transcripts, cleavage of the 3′ end results in a 3′-monophosphate scar. For RNase T1 digestion, 40 μl mRNA (1 mg ml^−1^) was mixed with 60 μl urea (8 M; Sigma-Aldrich), 12 μl Tris-HCl (1 M at pH 7.0; Invitrogen) and 0.8 μl EDTA (0.5 M; Invitrogen, Thermo Fisher). The samples were then denatured at 90 °C for 10 min and cooled at room temperature. RNase T1 (20 μl of 1,000 U μl^−1^, Thermo Fisher Scientific) was added, and the samples were incubated at 37 °C for 15 min. Treated samples were separated and analyzed via reversed-phase ion-pairing liquid chromatography using an Agilent 1290 UPLC and Agilent 6530 quadrupole time-of-flight mass spectrometer.

### dsRNA ELISA assay

A complementary method to the RNase T1 digest assay is the dsRNA sandwich enzyme-linked immunosorbent assay (ELISA), which selectively identifies dsRNA species that are at least ~40 bp in length, as described previously by Nelson et al.^6^ K1 mouse monoclonal antibodies (SCICONS) were immobilized on 96-well Nunc Immuno plates (Thermo Fisher Scientific) for 3 h and blocked with 10% nonfat dry milk in PBS overnight at 4 °C. mRNA samples were added to the plates and incubated for 2 h at room temperature, washed and exposed to K2 mouse monoclonal antibody solution (SCICONS) at room temperature for 1 h. Following the incubation steps, plates were exposed to horseradish peroxidase-conjugated goat anti-mouse immunoglobulin G detection antibody (Thermo Fisher Scientific) for 1 h following a final wash. Signal detection was performed at 450 nm on a Synergy H1 plate reader (BioTek). Determination of dsRNA concentration in total RNA was calculated using a standard curve generated with 400-bp dsRNA prepared using 100% N1-methyl-pseudouridine chemistry.

### IFN-β response in BJ fibroblasts

IFN-β response in BJ fibroblasts is used to provide a qualitative measurement of innate immune response. As previously described by Nelson et al.^6^, BJ fibroblasts acquired from the American Type Culture Collection (ATCC) were cultured in complete media comprising Eagle’s minimal essential medium with l-glutamine (ATCC) supplemented with 10% heat-inactivated fetal bovine serum (HI-FBS) (Life Technologies). Cells were seeded in 96-well cell culture plates (Corning Inc.) at 20,000 cells per well and maintained for 24 h before transfection. Cells were transfected with mRNA (250 ng per well) or poly(I:C) (10 ng μl^−1^; InvivoGen) using Lipofectamine 2000 (Thermo Fisher Scientific). At 48 h after transfection, supernatants were harvested and analyzed, and IFN-β protein levels in culture supernatants and mouse sera were determined via Ella microfluidic ELISA (ProteinSimple) per the manufacturer’s protocol.

### Culture of human MDMs

As previously described by Nelson et al.^6^, frozen aliquots of human monocytes were thawed and suspended in complete media comprising Roswell Park Memorial Institute (RPMI) 1640 growth medium (Life Technologies) supplemented with 10% HI-FBS (Life Technologies) and human recombinant macrophage colony-stimulating factor (M-CSF; 20 ng ml^−1^; Invitrogen). The resuspended cells were seeded in 96-well flat-bottomed culture plates (Corning) at 150,000 cells per well, followed by incubation for 4 days to facilitate macrophage differentiation. At 24 h before transfection, the growth medium was replaced with fresh RPMI 1640 supplemented with 10% HI-FBS. Thereafter, MDMs were transfected with mRNA (250 ng per well). Following 5 h of incubation, the supernatant was discarded, and the cells were lysed with branched DNA lysis buffer.

### IP-10 branched DNA assay

As previously described by Nelson et al.^6^, all reagents used for the IP-10 branched DNA assay formed part of the QuantiGene Singleplex Assay Kit (Thermo Fisher Scientific). A working probe set was prepared, including a capture extender, label extender, blocking probe and IP-10 probe per the instructions, and added to the capture plate. The prepared MDM cell lysates were incubated with the working probe solution for 16 to 22 h at 55 °C to facilitate hybridization. Reaction amplification was performed by the addition of a preamplifier, amplifier and label probe, with intermittent incubation and wash steps. The signal was detected on a Synergy H1 luminometer (BioTek) via chemiluminescent substrate.

### In vivo mouse models

All animal procedures and experiments were approved by the Institutional Animal Care and Use Committee at Moderna and conducted in accordance to ARRIVE guidelines. Female C57BL/6 mice (*n* = 4) of ~8 weeks of age were obtained from Charles River Laboratories. Mice were injected intravenously via the tail vein with lipid nanoparticle formulations containing hEPO mRNA at 0.5 mg kg^−1^ and euthanized 6 h after injection. Blood was collected by cardiac puncture after euthanasia and processed for serum.

### Mouse ProcartaPlex immunoassay

Cytokine levels in mouse sera were evaluated per the manufacturer’s instructions using a bead-based ProcartaPlex immunoassay kit (Thermo Fisher Scientific) consisting of granulocyte CSF, IFN-α, IFN-γ, IL-12p70, IL-6, CXCL10, CCL2, CCL4, CCL7 and TNFα.

### hEPO ELISA

hEPO expression levels were measured from serum collected 6 h after dosing in mice by an Ella microfluidic ELISA (ProteinSimple) per the manufacturer’s recommendations.

### Reporting summary

Further information on research design is available in the Nature Research Reporting Summary linked to this article.

## Online content

Any methods, additional references, Nature Research reporting summaries, source data, extended data, supplementary information, acknowledgements, peer review information; details of author contributions and competing interests; and statements of data and code availability are available at 10.1038/s41587-022-01525-6.

## Supplementary information

Supplementary Information Supplementary Figs. 1–5 and Supplementary Tables 1–7.

Reporting Summary

Supplementary Data 1 Full validation report for PDB entry 1CEZ.

Supplementary Data 2 Full validation report for PDB entry 1H38.

Supplementary Data 3 Full validation report for PDB entry 1MSW.

Supplementary Data 4 Full validation report for PDB entry 1QLN.

Supplementary Data 5 Full validation report for PDB entry 2PI4.

Supplementary Data 6 Full validation report for PDB entry 3E2E.

Supplementary Data 7 Full validation report for PDB entry 1S76.

Supplementary Data 8 Source data for Supplementary Fig. 2.

Supplementary Data 9 Source data for Supplementary Fig. S4.

Supplementary Data 10 Source data for Supplementary Fig. S5.

## Data Availability

All relevant data supporting the findings of this study are available within the article and its Supplementary Information files. Source data are provided with this paper for Figs. 2–5 and Supplementary Figs. 2, 4 and 5. All crystal structures used in this study were obtained from the PDB and have been referenced accordingly (PDB identification codes: 1CEZ, 1H38, 1MSW, 1QLN, 2PI4, 1S76, 3E2E; full validation reports are provided with this paper). Any other structure models mentioned in the manuscript were predicted using the computational protocols described in the Methods.

## Source data

Source Data Fig. 2 Source data for Fig. 2.
Source Data Fig. 3 Source data for Fig. 3.
Source Data Fig. 4 Source data for Fig. 4.
Source Data Fig. 5 Source data for Fig. 5.

## Code availability

All relevant code supporting the findings of this study are available within the Supplementary Information files.
