# Supplementary material for: An engineered T7 RNA polymerase that produces mRNA free of immunostimulatory byproducts
Source: Nat Biotechnol. 2022 Nov 10;41(4):560–8. doi: 10.1038/s41587-022-01525-6 (PMC10110463; doi:10.1038/s41587-022-01525-6)
Supplement: Supplementary file 1 — Supplementary Figs. 1–5 and Supplementary Tables 1–7. [file 41587_2022_1525_MOESM1_ESM.pdf]

nature biotechnology

Article

https://doi.org/10.1038/s41587-022-01525-6

An engineered T7 RNA polymerase that produces mRNA free of immunostimulatory byproducts

In the format provided by the
authors and unedited

SUPPLEMENTARY INFORMATION

An engineered T7 RNA polymerase that produces mRNA free of immunostimulatory byproducts

Dousis, et al.

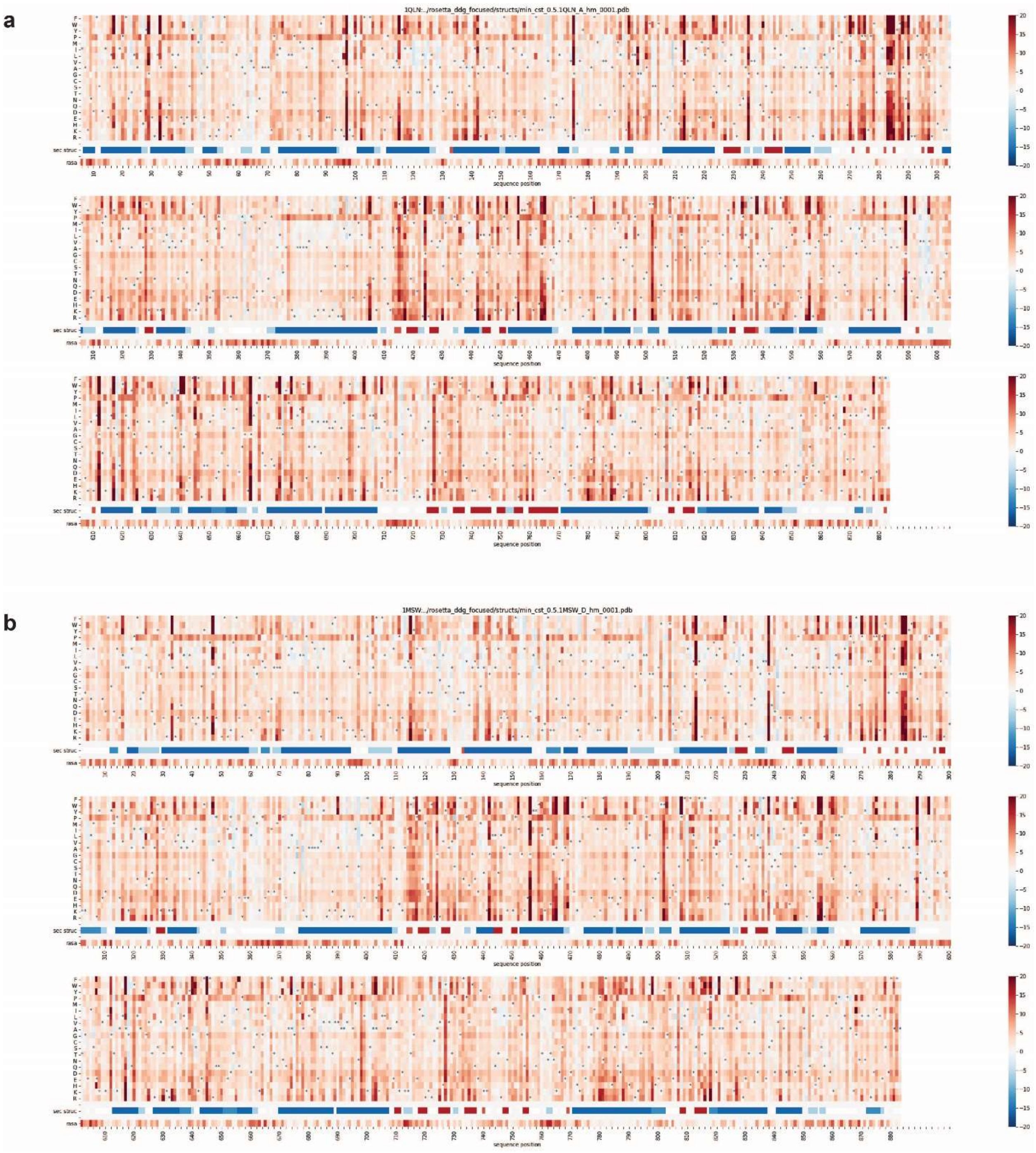

Figure S1. a) $\Delta\Delta E_{mut}$ heatmap for structure of initiation complex (pdb 1QlN). The columns correspond to amino acid sequence positions (1-883), and the rows correspond to amino acid

identities. The amino acids are grouped and ordered as per Kowalsky.[1] Asterisks ('*') denote the wildtype amino acid at each position. The $\Delta\Delta E_{mut}$ values are in Rosetta energy units (REUs), and the heatmap spans -20 REUs (blue) to 20 REUs (red). The bottom two rows correspond to secondary structure (blue: alpha helix, light blue: helix turn, red: beta strand) and relative solvent accessible surface area. **b**) $\Delta\Delta E_{mut}$ heatmap for structure of elongation complex (pdb 1MSW). The columns correspond to amino acid sequence positions (1-883), and the rows correspond to amino acid identities. The amino acids are grouped and ordered as per Kowalsky.[1] Asterisks ('*') denote the wildtype amino acid at each position. The $\Delta\Delta E_{mut}$ values are in Rosetta energy units (REUs), and the heatmap spans -20 REUs (blue) to 20 REUs (red). The bottom two rows correspond to secondary structure (blue: alpha helix, light blue: helix turn, red: beta strand) and relative solvent accessible surface area.

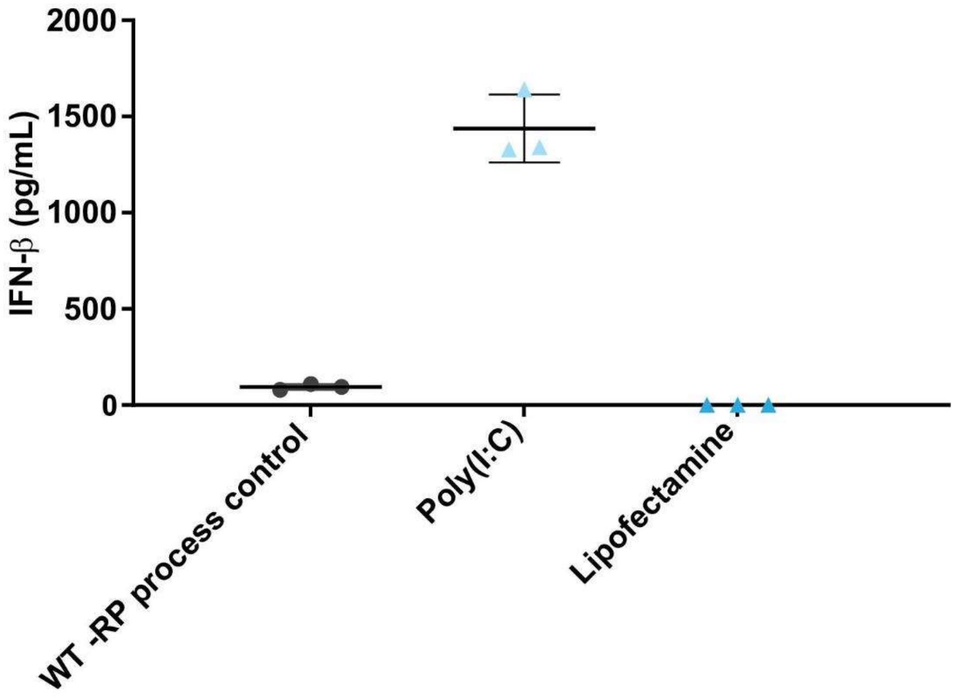

Figure S2: IFN-β response for the commercially available known RIG-I agonist, Poly(I:C) (InvivoGen), was tested in BJ Fibroblasts using n=3 technical replicates (error bars represent standard deviation). Cells were transfected with 250 ng/µL mRNA (WT -RP process control) or 10 ng/µL Poly(I:C) using Lipofectamine 2000 (Thermo Fisher Scientific). Cell culture supernatants were harvested 48 hours after transfection for IFN-β analysis.

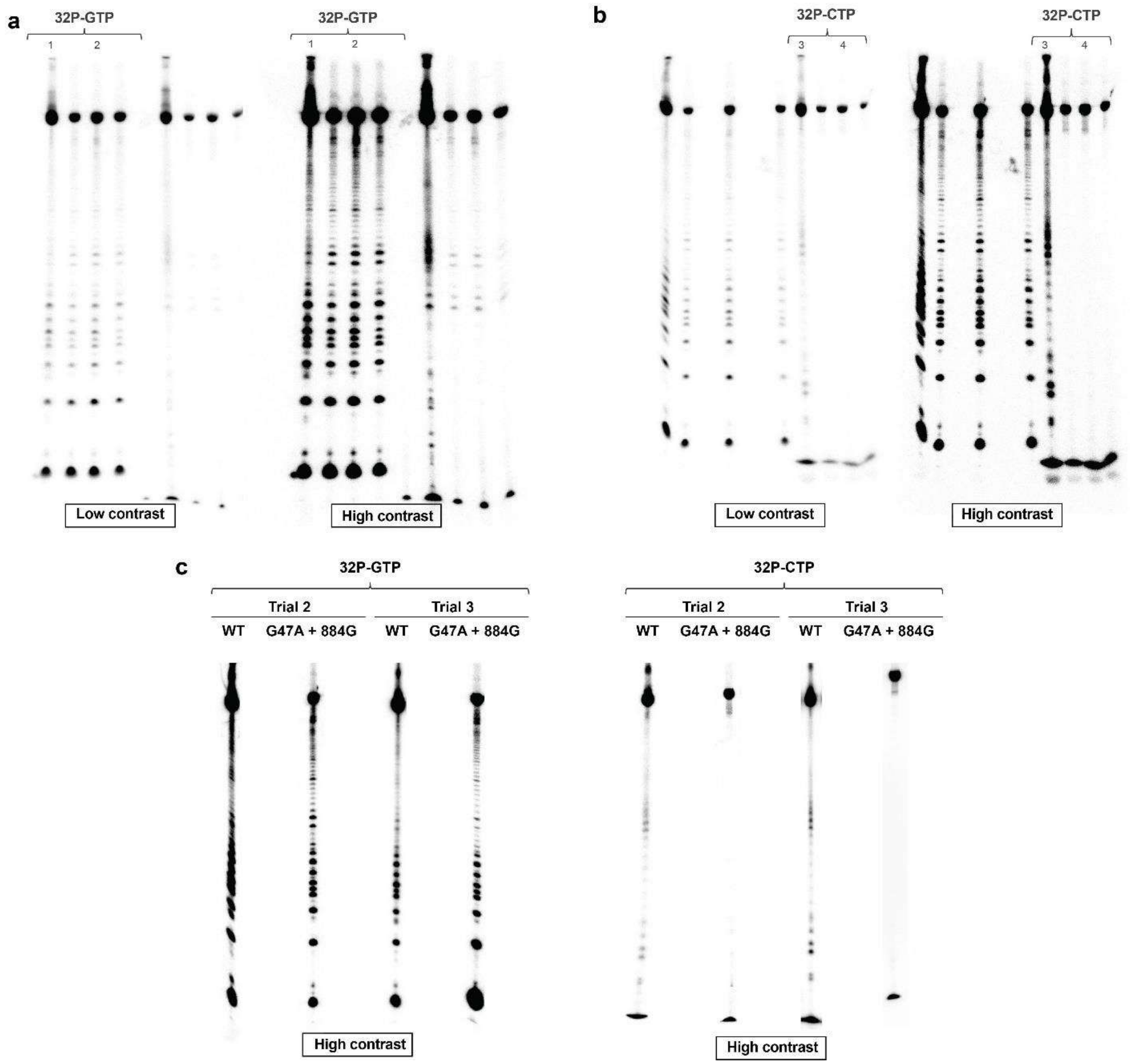

Figure S3: a and c) Original radioactive gels of IVT products generated by T7 RNAP WT and G47A+884G mutant. Only high contrast figures are displayed in the manuscript. **a**) Low and high contrast uncropped gel for ^{32}P-GTP; lanes marked 1 and 2 correspond to WT and G47A+884G, respectively in Figure 4A (blue panels). **b**) Low and high contrast uncropped gels for ^{32}P-GTP; lanes marked 3 and 4 correspond to WT and G47A+884G, respectively in Figure 4A (red panels). **c)** Two additional independent trials of IVT and cropped radioactive sequencing

gels (only high contrast cropped gels are shown) to monitor abortive (^{32}P-GTP) and reverse complements (^{32}P-CTP).

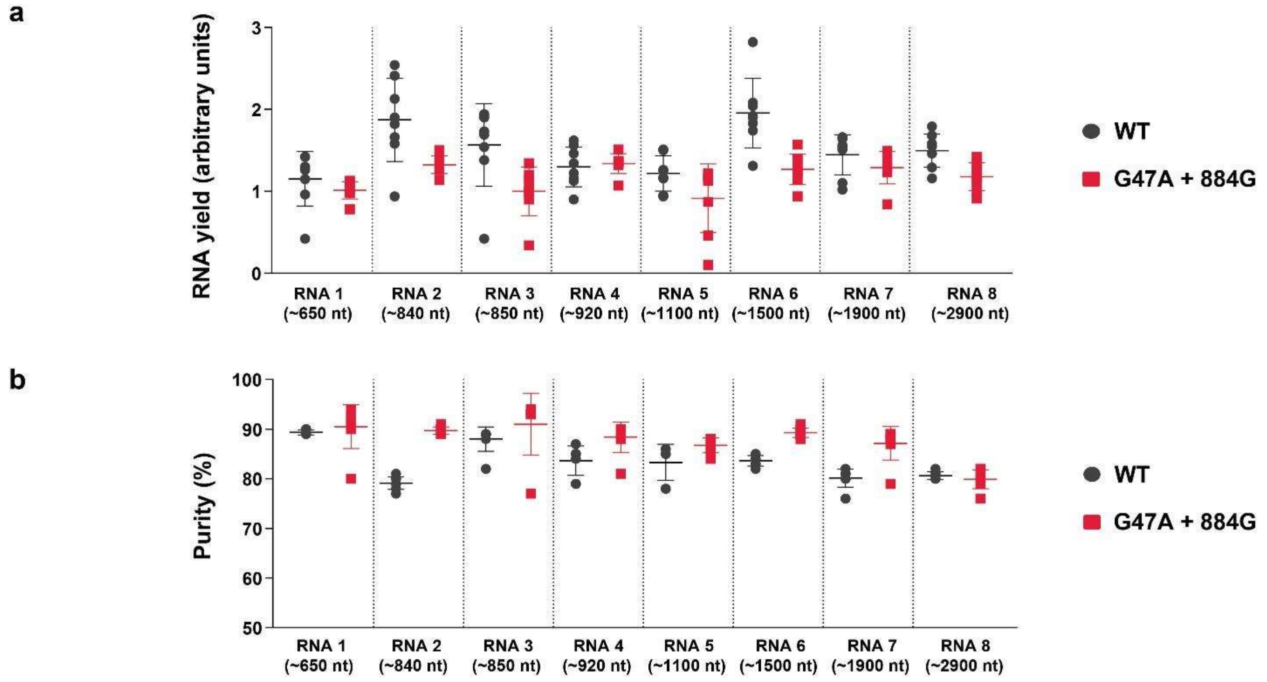

Figure S4: **a)** RNA yield and **b)** purity were evaluated for the 8 mRNAs with varying lengths and sequence composition presented in **Figure 4b-d** (N=8 mRNA lots; error bars represent standard deviation). RNA yield was measured by UV using A260 nm and RNA purity was measured by capillary electrophoresis via Fragment Analyzer.

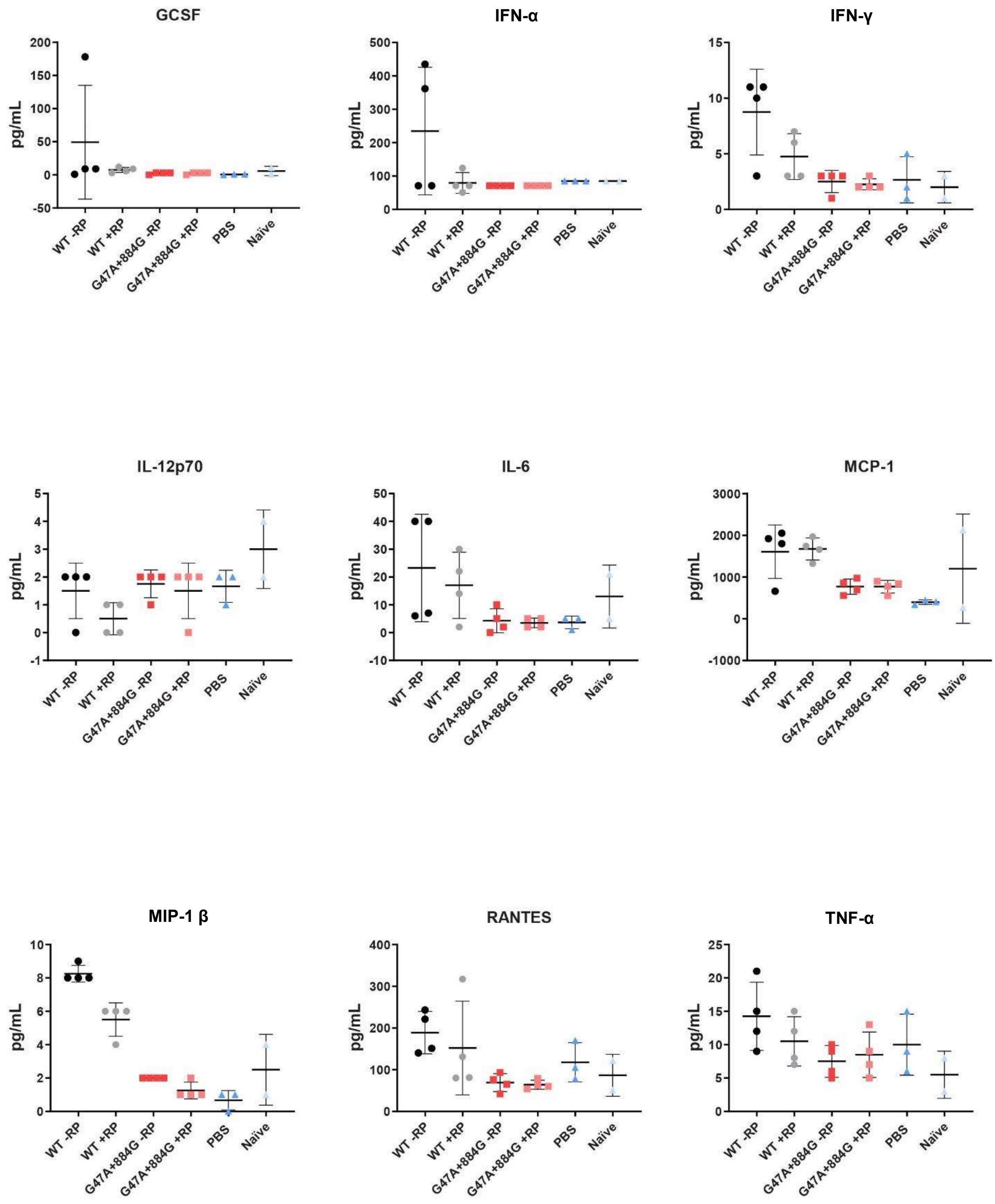

Figure S5 *In vivo* analysis of hEPO mRNA evaluated serum cytokine and chemokines using an innate immune response Luminex panel. Data are presented as mean ± standard deviation,

N=4 for all treatment groups, N=3 for PBS and naive controls. GCSF, granulocyte-colony stimulating factor; hEPO, human erythropoietin; IL, interleukin; MCP-1, monocyte chemoattractant protein-1; MIP-1, macrophage inflammatory protein-1; mRNA, messenger RNA; PBS, phosphate-buffered saline; RP, reverse phase; TNF, tumor necrosis factor; WT, wild-type.

Table S1. Flags used with RosettaCM: partial threading.

<table>
<tr><td>-in:file:template_pdb 1MSW_D2A_renumber.pdb
-ignore_unrecognized_res
-in:file:fasta target.fasta
-in:file:alignment target_template_aligned.aln
-relax:constrain_relax_to_start_coords</td></tr>
</table>

Table S2. Target-template alignments (target_template_aligned.aln).

<table>
<tr><td>## sp|P00573|RPOL_BPT7 1QLN_GTP_renumber.pdb_thread
#
scores from program: 0
0
NTINIAKNDFSDIELAAIPFNTLADHYGERLAREQLALEHESYEMGEARFRKMFERQLKAGEVADNAAAKPLITTLLPKMIARINDW
FEEVKAKRGKRPTAFQFLQEIKPEAVAYITIKTTLACLTSADNTTVQAVASAIGRAIEDEARFGRIRDLEAKHFKKNVEEQLNKRVGH
VYKKAFMQVVEADMLSKGLLGGEAWSSWHKEDSIHVGVRCIEMLIESTGMVSLHRQNAGVVGQDSETIELAPEYAEAIATRAGAL
AGISPMFQPCVVPPKPWTGITGGGYWANGRRPLALVRTHSKKALMRYEDVYMPEVYKAINIAQNTAWKINKKVLAVANVITKWK
HCPVEDIPAIEREELPMKPEDIDMNPEALTAWKRAAAAVYRKDKARKSRRISLEFMLEQANKFANHKAIWFPYNMDWRGRVYAVS
MFNPQGNDMTKGLLTLAKGKPIGKEGYYWLKIHGANCAGVDKVPFPERIKFIEENHENIMACAKSPLENTWWAEQDSPFCFLAFCF
EYAGVQHHGLSYNCSLPLAFDGSCSGIQHFSAMLRDEVGGRAVNLLPSETVQDIYGIVAKKVNEILQADAINGTDNEVVTVTDENT
GEISEKVKLGTKALAGQWLAYGVTRSVTKRSVMTLAYGSKEFGFRQQVLEDTIQPAIDSGKGLMFTQPNQAAGYMAKLIWESVSV
TVVAAVEAMNWLKSAAKLLAAEVKDKKTGEILRKRCAVHWVTPDGFPVWQEYKKPIQTRLNLMFLGQFRLQPTINTNKDSEIDAH
KQESGIAPNFVHSQDGSHLRKTVVWAHEKYGIESFALIHDSFGTIPADAANLFKAVRETMVDTYESCDVLADFYDQFADQLHESQL
DKMPALPAKGNLNLRDILESDFAFA
0 ----IAKNDFSDIELAAIPFNTLADHYGERLAREQLALEHESYEMGEARFRKMF----------------
PLITTLLPKMIARINDWFEEVKAKRGKRPTAFQFLQEIKPEAVAYITIKTTLACLTSADNTTVQAVASAIGRAIEDEARFGRIRDLEAK
HFKKNVEEQLNKRVGHVYKKAFMQVVEADMLSKGLLGGEAWSSWHKEDSIHVGVRCIEMLIESTGMVSLHRQNAGVVGQDSETI
ELAPEYAEAIATRAGALAGISPMFQPCVVPPKPWTGITGGGYWANGRRPLALVRTHSKKALMRYEDVYMPEVYKAINIAQNTAWK
INKKVLAVANVITKWKHCPVEDIPAIEREELPMKPEDIDMNPEALTAWKRAAAAVYRKDKARKSRRISLEFMLEQANKFANHKAIW
FPYNMDWRGRVYAVSMFNPQGNDMTKGLLTLAKGKPIGKEGYYWLKIHGANCAGVDKVPFPERIKFIEENHENIMACAKSPLENT
WWAEQDSPFCFLAFCFEYAGVQHHGLSYNCSLPLAFDGSCSGIQHFSAMLRDEVGGRAVNLLPSETVQDIYGIVAKKVNEILQADAI
NGTDNEVVTVTDENTGEISEKVKLGTKALAGQWLAYGVTRSVTKRSVMTLAYGSKEFGFRQQVLEDTIQPAIDSGKGLMFTQPNQ
AAGYMAKLIWESVSVTVVAAVEAMNWLKSAAKLLAAEVKDKKTGEILRKRCAVHWVTPDGFPVWQEYKKPIQTRLNLMFLGQF
RLQPTINTNKDSEIDAHKQESGIAPNFVHSQDGSHLRKTVVWAHEKYGIESFALIHDSFGTIPADAANLFKAVRETMVDTYESCDVL
ADFYDQFADQLHESQLDKMPALPAKGNLNLRDILESDFAFA</td></tr>
<tr><td>## sp|P00573|RPOL_BPT7 1MSW_D2A_renumber.pdb_thread
#
scores from program: 0
0
NTINIAKNDFSDIELAAIPFNTLADHYGERLAREQLALEHESYEMGEARFRKMFERQLKAGEVADNAAAKPLITTLLPKMIARINDW
FEEVKAKRGKRPTAFQFLQEIKPEAVAYITIKTTLACLTSADNTTVQAVASAIGRAIEDEARFGRIRDLEAKHFKKNVEEQLNKRVGH
VYKKAFMQVVEADMLSKGLLGGEAWSSWHKEDSIHVGVRCIEMLIESTGMVSLHRQNAGVVGQDSETIELAPEYAEAIATRAGAL
AGISPMFQPCVVPPKPWTGITGGGYWANGRRPLALVRTHSKKALMRYEDVYMPEVYKAINIAQNTAWKINKKVLAVANVITKWK
HCPVEDIPAIEREELPMKPEDIDMNPEALTAWKRAAAAVYRKDKARKSRRISLEFMLEQANKFANHKAIWFPYNMDWRGRVYAVS
MFNPQGNDMTKGLLTLAKGKPIGKEGYYWLKIHGANCAGVDKVPFPERIKFIEENHENIMACAKSPLENTWWAEQDSPFCFLAFCF
EYAGVQHHGLSYNCSLPLAFDGSCSGIQHFSAMLRDEVGGRAVNLLPSETVQDIYGIVAKKVNEILQADAINGTDNEVVTVTDENT
GEISEKVKLGTKALAGQWLAYGVTRSVTKRSVMTLAYGSKEFGFRQQVLEDTIQPAIDSGKGLMFTQPNQAAGYMAKLIWESVSV
TVVAAVEAMNWLKSAAKLLAAEVKDKKTGEILRKRCAVHWVTPDGFPVWQEYKKPIQTRLNLMFLGQFRLQPTINTNKDSEIDAH
KQESGIAPNFVHSQDGSHLRKTVVWAHEKYGIESFALIHDSFGTIPADAANLFKAVRETMVDTYESCDVLADFYDQFADQLHESQL
DKMPALPAKGNLNLRDILESDFAFA
0
NTINIAKNDFSDIELAAIPFNTLADHYGERLAREQLALEHESYEMGEARFRKMFERQLKAGEVADNAAAKPLITTLLPKMIARINDW
FEEVKAKRGKRPTAFQFLQEIKPEAVAYITIKTTLACLTSADNTTVQAVASAIGRAIEDEARFGRIRDLEAKHFKKNVEEQLNKRVGH
VYKKAFMQVVEADMLSKGLLGGEAWSSWHKEDSIHVGVRCIEMLIESTGMVSLHRQ--------
SETIELAPEYAEAIATRAGALAGISPMFQPCVVPPKPWTGITGGGYWANGRRPLALVRTHSKKALMRYEDVYMPEVYKAINIAQNT
AWKINKKVLAVANVITKWKHCPVEDIPAIEREELPMK-----------
TAWKRAAAAVYRKDKARKSRRISLEFMLEQANKFANHKAIWFPYNMDWRGRVYAVSMFNPQGNDMTKGLLTLAKGKPIGKEGY
YWLKIHGANCAGVDKVPFPERIKFIEENHENIMACAKSPLENTWWAEQDSPFCFLAFCFEYAGVQHHGLSYNCSLPLAFDGSCSGIQ
HFSAMLRDEVGGRAVNLLPSETVQDIYGIVAKKVNEILQADAINGTDNEVVTVTDENTGEISEKVKLGTKALAGQWLAYGVTRSVT
KRSVMTLAYGSKEFGFRQQVLEDTIQPAIDSGKGLMFTQPNQAAGYMAKLIWESVSVTVVAAVEAMNWLKSAAKLLAAEVKDKK
TGEILRKRCAVHWVTPDGFPVWQEYKKPIQTRLNLMFLGQFRLQPTINTNKDSEIDAHKQESGIAPNFVHSQDGSHLRKTVVWAHE
KYGIESFALIHDSFGTIPADAANLFKAVRETMVDTYESCDVLADFYDQFADQLHESQLDKMPALPAKGNLNLRDILESDFAFA</td></tr>
</table>

Table S3. Flags used with RosettaCM: hybridize

```
-in:file:fasta target.fasta
-parser:protocol hybridize.xml
-default_max_cycles 200
-dualspace
-nstruct 5
```

Table S4. Hybridize XML script (hybridize.xml)

```
<ROSETTASCRIPTS>
<TASKOPERATIONS>
</TASKOPERATIONS>
<SCOREFXNS>
   <ScoreFunction name="stage1" weights="score3" symmetric="0">
      <Reweight scoretype="cart_bonded" weight="0.5"/>
      <Reweight scoretype="atom_pair_constraint" weight="0.5"/>
   </ScoreFunction>
   <ScoreFunction name="stage2" weights="score4_smooth_cart" symmetric="0">
      <Reweight scoretype="cart_bonded" weight="0.5"/>
      <Reweight scoretype="atom_pair_constraint" weight="0.5"/>
   </ScoreFunction>
   <ScoreFunction name="fullatom" weights="ref2015" symmetric="0">
      <Reweight scoretype="pro_close" weight="0.0"/>
      <Reweight scoretype="cart_bonded" weight="0.5"/>
      <Reweight scoretype="atom_pair_constraint" weight="0.5"/>
   </ScoreFunction>
</SCOREFXNS>
<MOVERS>
   <Hybridize name="hybridize" stage1_scorefxn="stage1" stage2_scorefxn="stage2" fa_scorefxn="fullatom" batch="1"
stage1_increase_cycles="1.0" stage2_increase_cycles="1.0" linmin_only="1" add_hetatm="1">
      <Template pdb="1MSW_D2A_renumber.pdb_thread.pdb" cst_file="AUTO" weight="1.000" />
   </Hybridize>
   <FastRelax name="relax" scorefxn="fullatom" />
</MOVERS>
<PROTOCOLS>
   <Add mover="hybridize" />
   <Add mover="relax" />
</PROTOCOLS>
<OUTPUT scorefxn="fullatom" />
</ROSETTASCRIPTS>
```

Table S5. Flags used with min_with_cst

```
-in:file:l pdblist.txt
-in:file:fullatom
-ignore_unrecognized_res
-ex1
-ex2
-ex1aro
-ex2aro
-use_input_sc
-fa_max_dis 9.0
-ddg::harmonic_ca_tether 0.5
-ddg::constraint_weight 1.0
-ddg::sc_min_only false
-ddg::out_pdb_prefix min_cst_0.5
```

Table S6. Flags used with ddg_monomer

```
-in:file:s <pdbfile>
-in:file:fullatom
-ddg:mut_file <mutfile>
```

```
-ignore_unrecognized_res
-ex1
-ex2
-ex1aro
-ex2aro
-use_input_sc
-ddg:mean false
-ddg:min true
-ddg:weight_file ddg.wts
-ddg:min_cst false
-ddg:sc_min_only false
-ddg:iterations 5
-ddg:output_silent true
-ddg:dump_pdbs false
-ddg:suppress_checkpointing true
-delete_old_poses
```

Table S7. Example mutfile for ddg_monomer (min_cst_0.5.model-3_0001.119.mutfile)

```
total 20
1
S 119 A
1
S 119 C
1
S 119 D
1
S 119 E
1
S 119 F
1
S 119 G
1
S 119 H
1
S 119 I
1
S 119 K
1
S 119 L
1
S 119 M
1
S 119 N
1
S 119 P
1
S 119 Q
1
S 119 R
1
S 119 S
1
S 119 T
1
S 119 V
1
S 119 W
1
S 119 Y
```

REFERENCES

1. Kowalsky, C.A., et al. Rapid fine conformational epitope mapping using comprehensive mutagenesis and deep sequencing. *J Biol Chem* **290,** 26457-26470 (2015)
