# Supplementary material for: An engineered T7 RNA polymerase that produces mRNA free of immunostimulatory byproducts
Source: Nat Biotechnol. 2022 Nov 10;41(4):560–8. doi: 10.1038/s41587-022-01525-6 (PMC10110463; doi:10.1038/s41587-022-01525-6)
Supplement: Supplementary file 2 — Reporting Summary [file 41587_2022_1525_MOESM2_ESM.pdf]

nature research

Corresponding author(s): Amy Rabideau

Last updated by author(s): Sep 6, 2022

Reporting Summary

Nature Research wishes to improve the reproducibility of the work that we publish. This form provides structure for consistency and transparency in reporting. For further information on Nature Research policies, see our Editorial Policies and the Editorial Policy Checklist.

Statistics

For all statistical analyses, confirm that the following items are present in the figure legend, table legend, main text, or Methods section.

n/a	Confirmed	
☐	☒	The exact sample size (*n*) for each experimental group/condition, given as a discrete number and unit of measurement
☐	☒	A statement on whether measurements were taken from distinct samples or whether the same sample was measured repeatedly
☐	☒	The statistical test(s) used AND whether they are one- or two-sided<br>*Only common tests should be described solely by name; describe more complex techniques in the Methods section.*
☒	☐	A description of all covariates tested
☐	☒	A description of any assumptions or corrections, such as tests of normality and adjustment for multiple comparisons
☐	☒	A full description of the statistical parameters including central tendency (e.g. means) or other basic estimates (e.g. regression coefficient) AND variation (e.g. standard deviation) or associated estimates of uncertainty (e.g. confidence intervals)
☐	☒	For null hypothesis testing, the test statistic (e.g. F, t, r) with confidence intervals, effect sizes, degrees of freedom and P value noted<br>*Give P values as exact values whenever suitable.*
☒	☐	For Bayesian analysis, information on the choice of priors and Markov chain Monte Carlo settings
☒	☐	For hierarchical and complex designs, identification of the appropriate level for tests and full reporting of outcomes
☐	☒	Estimates of effect sizes (e.g. Cohen's *d*, Pearson's *r*), indicating how they were calculated

Our web collection on statistics for biologists contains articles on many of the points above.

Software and code

Policy information about availability of computer code

Data collection	Data and source materials were collected from publicly available protein data bank (PDB) entries
Data analysis	Rosetta macromolecular modeling suite (v3.7) , Python Pandas package (v1.3.4), Python scipy.stats package (v1.5.0) and ProteinVolume software tool (v1.3). All other data analysis was performed by GraphPad Prism 8.0.

For manuscripts utilizing custom algorithms or software that are central to the research but not yet described in published literature, software must be made available to editors and reviewers. We strongly encourage code deposition in a community repository (e.g. GitHub). See the Nature Research guidelines for submitting code & software for further information.

Data

Policy information about availability of data

All manuscripts must include a data availability statement. This statement should provide the following information, where applicable:

- Accession codes, unique identifiers, or web links for publicly available datasets
- A list of figures that have associated raw data
- A description of any restrictions on data availability

The authors declare that a data availability statement was included within the manuscript which describes the availability of source data respective to specific figures, supplementary information and associated source data, and PDB accession codes. Any additional information may be obtained from the corresponding authors upon reasonable request.

Field-specific reporting

Please select the one below that is the best fit for your research. If you are not sure, read the appropriate sections before making your selection.

☒ Life sciences ☐ Behavioural & social sciences ☐ Ecological, evolutionary & environmental sciences

For a reference copy of the document with all sections, see nature.com/documents/nr-reporting-summary-flat.pdf

Life sciences study design

All studies must disclose on these points even when the disclosure is negative.

Sample size	The in vivo study sample size was determined based on prior experiments and experience. We chose the sample size of (n=4) to keep the study small, so the serum samples could be handled altogether.
Data exclusions	The available data in in vivo studies were fully presented in relevant figures.
Replication	Key in vivo findings were replicated a minimum of two independent studies. All findings were reliably reproduced.
Randomization	Mice were randomly assigned to different treatment groups.
Blinding	Investigators were blinded to treatment groups during data collection. Where possible, all laboratory parameters were quantified in a blinded fashion.

Reporting for specific materials, systems and methods

We require information from authors about some types of materials, experimental systems and methods used in many studies. Here, indicate whether each material, system or method listed is relevant to your study. If you are not sure if a list item applies to your research, read the appropriate section before selecting a response.

Materials & experimental systems

n/a	Involved in the study	
☐	☒	Antibodies
☐	☒	Eukaryotic cell lines
☒	☐	Palaeontology and archaeology
☐	☒	Animals and other organisms
☒	☐	Human research participants
☒	☐	Clinical data
☒	☐	Dual use research of concern

Methods

n/a	Involved in the study	
☒	☐	ChIP-seq
☒	☐	Flow cytometry
☒	☐	MRI-based neuroimaging

Antibodies

Antibodies used	K1 mouse monoclonal antibody; K2 mouse monoclonal antibody, hEPO antibody
Validation	These antibodies form part of validated ELISA kits manufactured by SCICONS and ProteinSimple

Eukaryotic cell lines

Policy information about cell lines

Cell line source(s)	ATCC for BJ fibroblasts; MDM from human Leukopak collected from healthy donors
Authentication	Cell lines were authenticated by Short Tandem Repeat (STR) profiling
Mycoplasma contamination	All cells tested negative
Commonly misidentified lines (See ICLAC register)	No commonly misidentified cell lines were included

Animals and other organisms

Policy information about studies involving animals; ARRIVE guidelines recommended for reporting animal research

Laboratory animals	Female C57BL/6 mice (8 weeks) were used.
Wild animals	No wild animals were used.
Field-collected samples	No samples were field-collected.
Ethics oversight	All experimental protocols were approved by the Institutional Animal Care and Use Committee at Moderna and complied with all relevant ethical regulations regarding the use of research animals.

Note that full information on the approval of the study protocol must also be provided in the manuscript.
