# Supplementary material for: An engineered T7 RNA polymerase that produces mRNA free of immunostimulatory byproducts
Source: Nat Biotechnol. 2022 Nov 10;41(4):560–8. doi: 10.1038/s41587-022-01525-6 (PMC10110463; doi:10.1038/s41587-022-01525-6)
Supplement: Supplementary file 3 — Full validation report for PDB entry 1CEZ. [file 41587_2022_1525_MOESM3_ESM.pdf]

Full wwPDB X-ray Structure Validation Report ⓘ

May 13, 2020 – 02:37 pm BST

PDB ID	:	1CEZ
Title	:	CRYSTAL STRUCTURE OF A T7 RNA POLYMERASE-T7 PROMOTER COMPLEX
Authors	:	Cheetham, G.M.T.; Jeruzalmi, D.; Steitz, T.A.
Deposited on	:	1999-03-11
Resolution	:	2.40 Å(reported)

This is a Full wwPDB X-ray Structure Validation Report for a publicly released PDB entry.

We welcome your comments at *validation@mail.wwpdb.org*
A user guide is available at
https://www.wwpdb.org/validation/2017/XrayValidationReportHelp
with specific help available everywhere you see the ⓘ symbol.

The following versions of software and data (see references ⓘ) were used in the production of this report:

MolProbity	:	4.02b-467
Xtriage (Phenix)	:	NOT EXECUTED
EDS	:	NOT EXECUTED
Percentile statistics	:	20191225.v01 (using entries in the PDB archive December 25th 2019)
Ideal geometry (proteins)	:	Engh & Huber (2001)
Ideal geometry (DNA, RNA)	:	Parkinson et al. (1996)
Validation Pipeline (wwPDB-VP)	:	2.11

1 Overall quality at a glance (i)

The following experimental techniques were used to determine the structure:
X-RAY DIFFRACTION

The reported resolution of this entry is 2.40 Å.

Percentile scores (ranging between 0-100) for global validation metrics of the entry are shown in the following graphic. The table shows the number of entries on which the scores are based.

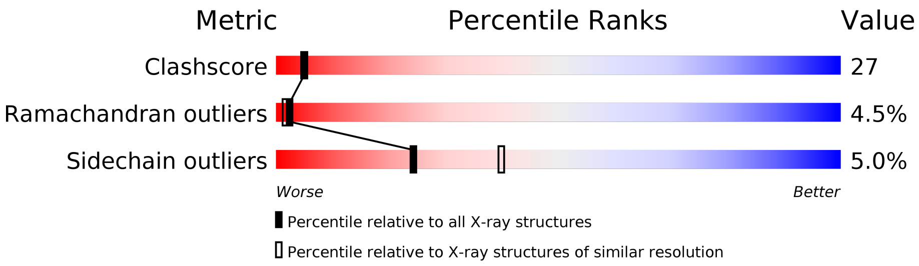

Metric	Whole archive (#Entries)	Similar resolution (#Entries, resolution range(Å))
Clashscore	141614	4398 (2.40-2.40)
Ramachandran outliers	138981	4318 (2.40-2.40)
Sidechain outliers	138945	4319 (2.40-2.40)

The table below summarises the geometric issues observed across the polymeric chains and their fit to the electron density. The red, orange, yellow and green segments on the lower bar indicate the fraction of residues that contain outliers for >=3, 2, 1 and 0 types of geometric quality criteria respectively. A grey segment represents the fraction of residues that are not modelled. The numeric value for each fraction is indicated below the corresponding segment, with a dot representing fractions <=5%

Note EDS was not executed.

Mol	Chain	Length	Quality of chain
1	T	17	24% 65% 12%
2	N	15	27% 60% 13%
3	A	883	59% 33% 6% •

2 Entry composition (i)

There are 4 unique types of molecules in this entry. The entry contains 7766 atoms, of which 0 are hydrogens and 0 are deuteriums.

In the tables below, the ZeroOcc column contains the number of atoms modelled with zero occupancy, the AltConf column contains the number of residues with at least one atom in alternate conformation and the Trace column contains the number of residues modelled with at most 2 atoms.

- Molecule 1 is a DNA chain called DNA (5'-D(P*TP*AP*TP*AP*GP*TP*GP*AP*GP*TP *CP*GP*TP*AP*TP*TP*A)-3').

Mol	Chain	Residues	Atoms					ZeroOcc	AltConf	Trace
			Total	C	N	O	P			
1	T	17	352	169	62	104	17	0	0	0

- Molecule 2 is a DNA chain called DNA (5'-D(P*TP*AP*AP*TP*AP*CP*GP*AP*CP*TP *CP*AP*CP*TP*A)-3').

Mol	Chain	Residues	Atoms					ZeroOcc	AltConf	Trace
			Total	C	N	O	P			
2	N	15	304	146	55	88	15	0	0	0

- Molecule 3 is a protein called PROTEIN (BACTERIOPHAGE T7 RNA POLYMERASE).

Mol	Chain	Residues	Atoms					ZeroOcc	AltConf	Trace
			Total	C	N	O	S			
3	A	862	6639	4228	1164	1211	36	0	0	0

- Molecule 4 is water.

Mol	Chain	Residues	Atoms		ZeroOcc	AltConf
			Total	O		
4	T	14	14	14	0	0
4	N	18	18	18	0	0
4	A	439	439	439	0	0

3 Residue-property plots (i)

These plots are drawn for all protein, RNA and DNA chains in the entry. The first graphic for a chain summarises the proportions of the various outlier classes displayed in the second graphic. The second graphic shows the sequence view annotated by issues in geometry. Residues are color-coded according to the number of geometric quality criteria for which they contain at least one outlier: green = 0, yellow = 1, orange = 2 and red = 3 or more. Stretches of 2 or more consecutive residues without any outlier are shown as a green connector. Residues present in the sample, but not in the model, are shown in grey.

Note EDS was not executed.

- Molecule 1: DNA (5'-D(P*TP*AP*TP*AP*GP*TP*GP*AP*GP*TP*CP*GP*TP*AP*TP*TP*A)-3')

Chain T:

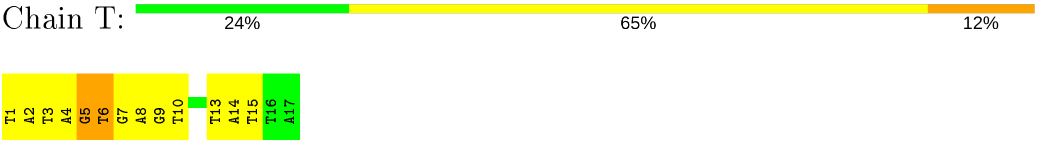

- Molecule 2: DNA (5'-D(P*TP*AP*AP*TP*AP*CP*GP*AP*CP*TP*CP*AP*CP*TP*A)-3')

Chain N:

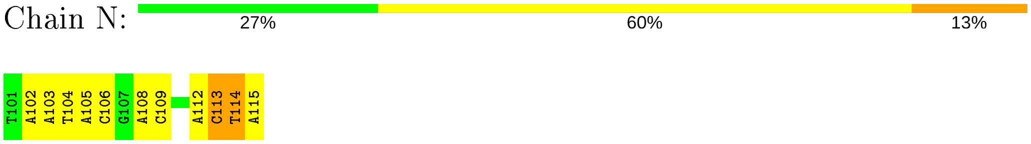

- Molecule 3: PROTEIN (BACTERIOPHAGE T7 RNA POLYMERASE)

Chain A:

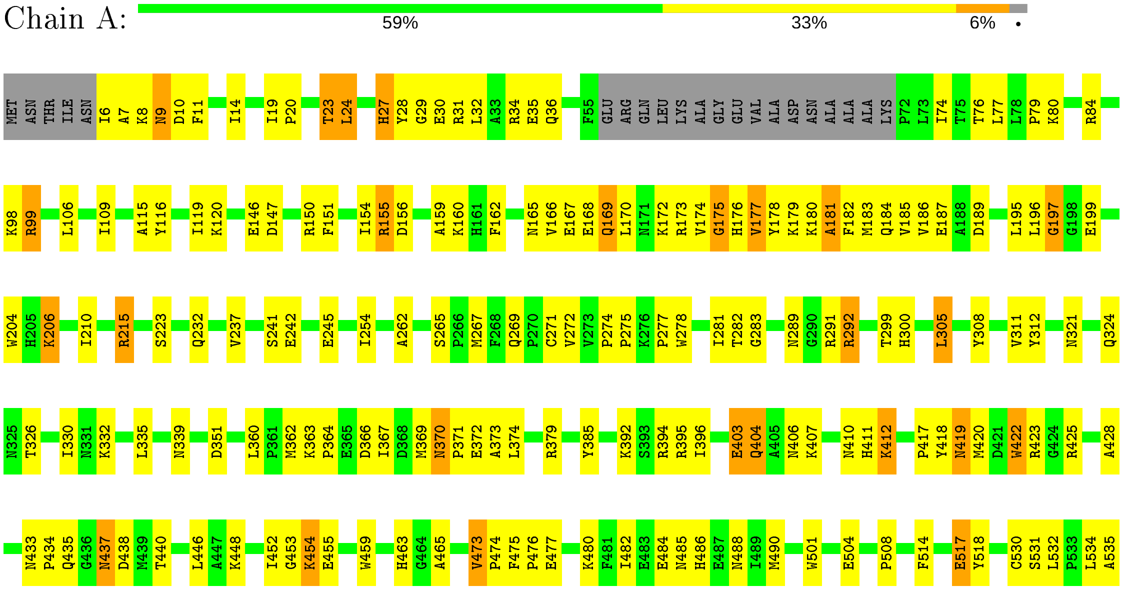

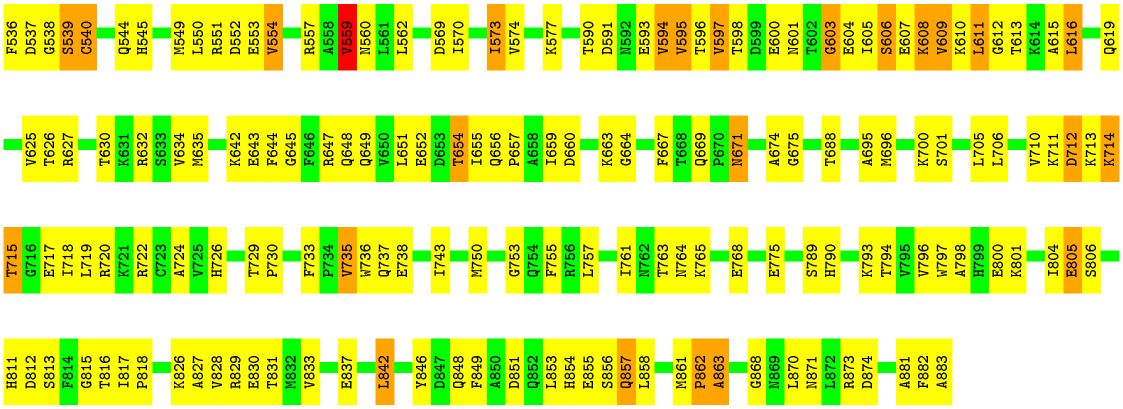
F536 D537 G538 S539 C540 Q544 H545 M549 L550 R551 D552 E553 V554 R557 A558 V559 N560 L561 L562 D569 I570 I573 V574 K577 T590 D591 N592 E593 V594 V595 T596 V597 T598 D599 E600 N601 T602 G603 E604 I605 S606 E607 K608 V609 K610 L611 G612 T613 K614 A615 L616 Q619
V625 T626 R627 T630 K631 R632 S633 V634 M635 K642 E643 F644 G645 F646 R647 Q648 Q649 V650 L651 E652 D653 T654 I655 Q656 P657 A658 I659 D660 K663 G664 F667 T668 Q669 P670 N671 A674 G675 T688 A695 M696 K700 S701 L705 L706 V710 K711 D712 K713 K714
T715 G716 E717 I718 L719 R720 K721 R722 C723 A724 V725 H726 T729 P730 F733 P734 V735 W736 Q737 E738 I743 M750 G753 Q754 F755 R756 L757 I761 N762 T763 N764 K765 E768 E775 S789 H790 K793 T794 V795 V796 W797 A798 H799 E800 K801 I804 E805 S806
H811 D812 S813 F814 G815 T816 I817 P818 K826 A827 V828 R829 E830 T831 M832 V833 E837 L842 Y846 D847 Q848 F849 A850 D851 Q852 L853 H854 E855 S856 Q857 L858 M861 P862 A863 G868 N869 L870 N871 L872 R873 D874 A881 F882 A883

4 Data and refinement statistics (i)

Xtriage (Phenix) and EDS were not executed - this section is therefore incomplete.

Property	Value	Source
Space group	P 21 21 2	Depositor
Cell constants<br>a, b, c, α, β, γ	220.10Å 73.30Å 80.90Å<br>90.00° 90.00° 90.00°	Depositor
Resolution (Å)	40.00 – 2.40	Depositor
% Data completeness<br>(in resolution range)	81.3 (40.00-2.40)	Depositor
R_{merge}	(Not available)	Depositor
R_{sym}	0.08	Depositor
Refinement program	CNS 0.5	Depositor
R, R_{free}	0.224 , 0.270	Depositor
Estimated twinning fraction	No twinning to report.	Xtriage
Total number of atoms	7766	wwPDB-VP
Average B, all atoms ($Å^2$)	53.0	wwPDB-VP

5 Model quality (i)

5.1 Standard geometry (i)

The Z score for a bond length (or angle) is the number of standard deviations the observed value is removed from the expected value. A bond length (or angle) with $|Z| > 5$ is considered an outlier worth inspection. RMSZ is the root-mean-square of all Z scores of the bond lengths (or angles).

Mol	Chain	Bond lengths		Bond angles	
		RMSZ	#|Z| >5	RMSZ	#|Z| >5
1	T	0.60	0/394	0.76	0/607
2	N	1.29	3/340 (0.9%)	1.05	0/521
3	A	0.49	1/6791 (0.0%)	0.69	3/9207 (0.0%)
All	All	0.56	4/7525 (0.1%)	0.72	3/10335 (0.0%)

Chiral center outliers are detected by calculating the chiral volume of a chiral center and verifying if the center is modelled as a planar moiety or with the opposite hand.A planarity outlier is detected by checking planarity of atoms in a peptide group, atoms in a mainchain group or atoms of a sidechain that are expected to be planar.

Mol	Chain	#Chirality outliers	#Planarity outliers
1	T	0	2
2	N	0	1
All	All	0	3

All (4) bond length outliers are listed below:

Mol	Chain	Res	Type	Atoms	Z	Observed(Å)	Ideal(Å)
3	A	805	GLU	CD-OE2	7.42	1.33	1.25
2	N	114	DT	C4-C5	6.42	1.50	1.45
2	N	114	DT	N1-C2	5.57	1.42	1.38
2	N	113	DC	O3'-P	5.44	1.67	1.61

All (3) bond angle outliers are listed below:

Mol	Chain	Res	Type	Atoms	Z	Observed(^{o})	Ideal(^{o})
3	A	559	VAL	N-CA-C	-5.55	96.01	111.00
3	A	181	ALA	N-CA-C	-5.50	96.16	111.00
3	A	540	CYS	N-CA-C	-5.26	96.80	111.00

There are no chirality outliers.

All (3) planarity outliers are listed below:

Mol	Chain	Res	Type	Group
2	N	106	DC	Sidechain
1	T	5	DG	Sidechain
1	T	6	DT	Sidechain

5.2 Too-close contacts (i)

In the following table, the Non-H and H(model) columns list the number of non-hydrogen atoms and hydrogen atoms in the chain respectively. The H(added) column lists the number of hydrogen atoms added and optimized by MolProbity. The Clashes column lists the number of clashes within the asymmetric unit, whereas Symm-Clashes lists symmetry related clashes.

Mol	Chain	Non-H	H(model)	H(added)	Clashes	Symm-Clashes
1	T	352	0	195	22	0
2	N	304	0	170	20	0
3	A	6639	0	6470	350	0
4	A	439	0	0	29	0
4	N	18	0	0	0	0
4	T	14	0	0	0	0
All	All	7766	0	6835	384	0

The all-atom clashscore is defined as the number of clashes found per 1000 atoms (including hydrogen atoms). The all-atom clashscore for this structure is 27.

All (384) close contacts within the same asymmetric unit are listed below, sorted by their clash magnitude.

Atom-1	Atom-2	Interatomic distance (Å)	Clash overlap (Å)
3:A:281:ILE:HG23	3:A:282:THR:HG23	1.30	1.13
2:N:113:DC:H3’	2:N:115:DA:OP1	1.49	1.11
3:A:710:VAL:HG11	3:A:720:ARG:H	1.10	1.08
3:A:829:ARG:HG3	3:A:829:ARG:HH11	1.16	1.07
3:A:763:THR:HG22	3:A:765:LYS:H	1.15	1.05
3:A:663:LYS:HG2	3:A:664:GLY:H	1.15	1.03
3:A:473:VAL:HG22	3:A:474:PRO:HD2	1.45	0.99
3:A:714:LYS:HA	3:A:714:LYS:HE2	1.47	0.96
3:A:155:ARG:HG3	3:A:155:ARG:HH11	1.28	0.95
3:A:595:VAL:HA	3:A:608:LYS:HA	1.44	0.94
3:A:537:ASP:O	3:A:882:PHE:HB2	1.67	0.93
2:N:103:DA:H2”	2:N:104:DT:H5’	1.50	0.93
3:A:560:ASN:O	3:A:881:ALA:HB2	1.71	0.90
3:A:269:GLN:HE22	3:A:407:LYS:NZ	1.68	0.90
3:A:647:ARG:HD2	3:A:675:GLY:HA2	1.53	0.89

Continued on next page...

Continued from previous page...

Atom-1	Atom-2	Interatomic distance (Å)	Clash overlap (Å)
3:A:710:VAL:CG1	3:A:720:ARG:H	1.86	0.87
3:A:324:GLN:HE21	3:A:418:TYR:H	1.21	0.86
3:A:710:VAL:HG11	3:A:720:ARG:N	1.89	0.86
3:A:816:THR:HG22	3:A:817:ILE:H	1.38	0.86
3:A:19:ILE:HG22	3:A:20:PRO:HD3	1.57	0.85
3:A:713:LYS:HD3	3:A:714:LYS:N	1.91	0.85
3:A:663:LYS:HG2	3:A:664:GLY:N	1.93	0.84
3:A:794:THR:OG1	3:A:831:THR:HG21	1.78	0.83
3:A:485:ASN:HD22	3:A:488:ASN:HD22	1.25	0.83
3:A:710:VAL:HG21	3:A:719:LEU:HB3	1.60	0.83
3:A:278:TRP:H	3:A:321:ASN:HD21	1.26	0.83
3:A:729:THR:CG2	3:A:733:PHE:H	1.92	0.82
3:A:711:LYS:HG2	3:A:712:ASP:H	1.45	0.81
3:A:713:LYS:HD3	3:A:714:LYS:H	1.46	0.81
3:A:861:MET:N	3:A:862:PRO:HD2	1.94	0.81
3:A:269:GLN:HE22	3:A:407:LYS:HZ3	1.28	0.81
3:A:395:ARG:HD3	4:A:1290:HOH:O	1.81	0.81
3:A:696:MET:O	3:A:700:LYS:HG3	1.80	0.80
3:A:34:ARG:HH22	3:A:165:ASN:HD22	1.27	0.80
3:A:798:ALA:HB1	3:A:804:ILE:HD12	1.62	0.80
3:A:846:TYR:HA	3:A:849:PHE:CE1	2.15	0.80
1:T:14:DA:H1’	1:T:15:DT:H5”	1.62	0.80
3:A:763:THR:HG22	3:A:765:LYS:N	1.96	0.79
3:A:711:LYS:HA	4:A:1295:HOH:O	1.83	0.79
3:A:729:THR:HG21	3:A:733:PHE:HB3	1.64	0.78
3:A:705:LEU:HD11	3:A:861:MET:HB2	1.65	0.78
3:A:632:ARG:HH21	3:A:654:THR:HG23	1.50	0.77
3:A:156:ASP:OD1	3:A:160:LYS:HE2	1.85	0.76
3:A:29:GLY:HA3	3:A:175:GLY:HA3	1.68	0.76
3:A:829:ARG:HG3	3:A:829:ARG:NH1	1.95	0.76
3:A:816:THR:HG22	3:A:817:ILE:N	2.00	0.76
3:A:147:ASP:O	3:A:150:ARG:O	2.05	0.75
3:A:170:LEU:O	3:A:174:VAL:HG23	1.86	0.75
3:A:19:ILE:O	3:A:23:THR:HG22	1.87	0.75
3:A:24:LEU:O	3:A:27:HIS:O	2.05	0.74
3:A:871:ASN:HB3	3:A:874:ASP:OD2	1.87	0.73
3:A:729:THR:HG22	3:A:733:PHE:H	1.55	0.72
2:N:114:DT:OP1	2:N:114:DT:H2’	1.90	0.72
3:A:177:VAL:O	3:A:179:LYS:N	2.23	0.72
3:A:729:THR:CG2	3:A:733:PHE:HB3	2.19	0.72
3:A:615:ALA:O	3:A:619:GLN:HG3	1.89	0.72

Continued on next page...

Continued from previous page...

Atom-1	Atom-2	Interatomic distance (Å)	Clash overlap (Å)
3:A:829:ARG:HH11	3:A:829:ARG:CG	1.94	0.72
3:A:706:LEU:HD21	3:A:849:PHE:HB2	1.72	0.71
3:A:713:LYS:HA	4:A:1219:HOH:O	1.88	0.71
3:A:169:GLN:HG2	3:A:172:LYS:HE3	1.71	0.71
3:A:517:GLU:HG3	3:A:530:CYS:SG	2.30	0.71
3:A:177:VAL:O	3:A:179:LYS:HG3	1.90	0.71
2:N:113:DC:O3'	2:N:114:DT:H3'	1.91	0.70
3:A:281:ILE:CG2	3:A:282:THR:HG23	2.15	0.69
3:A:635:MET:HA	4:A:1137:HOH:O	1.91	0.69
3:A:485:ASN:HD22	3:A:488:ASN:ND2	1.91	0.69
3:A:155:ARG:NH1	3:A:155:ARG:HG3	2.06	0.69
3:A:147:ASP:OD1	3:A:292:ARG:HD2	1.94	0.68
3:A:29:GLY:HA3	3:A:175:GLY:CA	2.23	0.68
3:A:23:THR:HG21	3:A:195:LEU:HD11	1.73	0.68
3:A:710:VAL:HG11	3:A:719:LEU:N	2.08	0.68
3:A:184:GLN:HE21	3:A:185:VAL:HG12	1.59	0.68
3:A:729:THR:HG22	3:A:733:PHE:N	2.08	0.68
2:N:103:DA:H2"	2:N:104:DT:C5'	2.23	0.68
3:A:311:VAL:O	3:A:312:TYR:HB3	1.94	0.67
3:A:642:LYS:HA	3:A:649:GLN:HE22	1.59	0.67
2:N:113:DC:C3'	2:N:115:DA:OP1	2.36	0.67
3:A:550:LEU:HD11	3:A:695:ALA:HB2	1.76	0.66
3:A:371:PRO:C	3:A:373:ALA:H	1.98	0.66
3:A:463:HIS:HE1	4:A:1101:HOH:O	1.78	0.66
1:T:4:DA:N1	3:A:206:LYS:HE2	2.11	0.66
3:A:630:THR:O	3:A:634:VAL:HG23	1.95	0.66
3:A:790:HIS:NE2	3:A:831:THR:HG23	2.11	0.66
3:A:364:PRO:C	3:A:366:ASP:H	1.98	0.66
2:N:113:DC:H3'	2:N:115:DA:P	2.36	0.66
2:N:113:DC:H2"	2:N:115:DA:OP2	1.96	0.65
3:A:34:ARG:HH22	3:A:165:ASN:ND2	1.93	0.65
3:A:710:VAL:HG11	3:A:719:LEU:H	1.60	0.65
3:A:797:TRP:CZ2	3:A:801:LYS:HG3	2.31	0.65
3:A:701:SER:HB3	3:A:861:MET:HG2	1.79	0.64
3:A:32:LEU:HD22	3:A:272:VAL:HG12	1.80	0.64
3:A:573:ILE:HD11	3:A:688:THR:HG23	1.78	0.64
3:A:438:ASP:OD2	3:A:508:PRO:HG2	1.97	0.64
2:N:113:DC:H4'	2:N:114:DT:OP2	1.96	0.64
3:A:169:GLN:HB3	3:A:172:LYS:HD2	1.78	0.63
3:A:23:THR:O	3:A:27:HIS:HD2	1.81	0.63
1:T:9:DG:H2"	1:T:10:DT:C5'	2.28	0.63

Continued on next page...

Continued from previous page...

Atom-1	Atom-2	Interatomic distance (Å)	Clash overlap (Å)
3:A:861:MET:H	3:A:862:PRO:HD2	1.60	0.63
3:A:651:LEU:HD23	3:A:651:LEU:O	1.99	0.63
3:A:553:GLU:CD	4:A:1176:HOH:O	2.36	0.63
3:A:826:LYS:O	3:A:830:GLU:HG3	1.98	0.63
3:A:116:TYR:CZ	3:A:120:LYS:HD2	2.33	0.63
3:A:369:MET:C	3:A:371:PRO:HD2	2.20	0.63
1:T:2:DA:H1'	1:T:3:DT:H5'	1.80	0.62
3:A:486:HIS:O	3:A:490:MET:HG2	1.99	0.62
3:A:159:ALA:HA	3:A:162:PHE:CE2	2.34	0.62
3:A:35:GLU:OE2	3:A:411:HIS:HE1	1.82	0.62
2:N:114:DT:H3'	2:N:114:DT:P	2.39	0.62
3:A:115:ALA:O	3:A:119:ILE:HG12	1.99	0.62
3:A:36:GLN:NE2	3:A:272:VAL:H	1.97	0.61
3:A:412:LYS:HA	3:A:412:LYS:HE2	1.82	0.61
3:A:595:VAL:CA	3:A:608:LYS:HA	2.23	0.61
3:A:610:LYS:O	3:A:611:LEU:O	2.18	0.61
3:A:750:MET:HE1	3:A:755:PHE:N	2.16	0.60
3:A:612:GLY:O	3:A:616:LEU:HD22	2.01	0.60
3:A:19:ILE:CG2	3:A:20:PRO:HD3	2.30	0.60
3:A:179:LYS:O	3:A:181:ALA:N	2.35	0.60
3:A:269:GLN:HE22	3:A:407:LYS:HZ2	1.49	0.60
3:A:321:ASN:HB3	4:A:1138:HOH:O	2.01	0.60
3:A:330:ILE:HG21	3:A:335:LEU:HD12	1.84	0.60
3:A:714:LYS:HA	3:A:714:LYS:CE	2.26	0.59
3:A:120:LYS:HE2	3:A:265:SER:O	2.00	0.59
3:A:179:LYS:HG2	4:A:1025:HOH:O	2.01	0.59
3:A:647:ARG:HD2	3:A:675:GLY:CA	2.29	0.59
3:A:715:THR:O	3:A:715:THR:HG22	2.02	0.59
3:A:480:LYS:O	3:A:484:GLU:HG3	2.03	0.59
3:A:647:ARG:C	3:A:649:GLN:H	2.05	0.58
3:A:710:VAL:CG1	3:A:719:LEU:H	2.16	0.58
3:A:169:GLN:HG2	3:A:172:LYS:CE	2.33	0.58
1:T:9:DG:H1'	1:T:10:DT:H5"	1.84	0.58
3:A:663:LYS:CG	3:A:664:GLY:H	2.00	0.58
3:A:215:ARG:NH2	3:A:750:MET:O	2.37	0.58
3:A:278:TRP:H	3:A:321:ASN:ND2	2.00	0.58
3:A:858:LEU:N	4:A:1062:HOH:O	2.36	0.58
3:A:155:ARG:CG	3:A:155:ARG:HH11	2.06	0.57
3:A:242:GLU:HG2	3:A:757:LEU:HD11	1.86	0.57
3:A:36:GLN:HE22	3:A:271:CYS:HA	1.70	0.57
3:A:632:ARG:HH21	3:A:654:THR:CG2	2.16	0.57

Continued on next page...

Continued from previous page...

Atom-1	Atom-2	Interatomic distance (Å)	Clash overlap (Å)
3:A:540:CYS:O	3:A:544:GLN:HG3	2.05	0.57
3:A:485:ASN:ND2	3:A:488:ASN:HD22	1.99	0.56
3:A:448:LYS:HE3	3:A:805:GLU:HB2	1.88	0.56
3:A:655:ILE:HG23	3:A:667:PHE:CD2	2.40	0.56
3:A:713:LYS:O	3:A:714:LYS:C	2.43	0.56
3:A:335:LEU:CD2	3:A:339:ASN:ND2	2.69	0.56
3:A:816:THR:CG2	3:A:817:ILE:H	2.13	0.56
3:A:167:GLU:HA	4:A:1156:HOH:O	2.05	0.56
1:T:2:DA:H2"	1:T:3:DT:OP2	2.06	0.56
3:A:177:VAL:HB	3:A:179:LYS:HE3	1.86	0.56
3:A:608:LYS:O	3:A:609:VAL:HB	2.05	0.56
3:A:166:VAL:HG13	3:A:173:ARG:CD	2.36	0.56
3:A:729:THR:OG1	3:A:789:SER:HB3	2.06	0.56
3:A:632:ARG:NH2	3:A:654:THR:HG23	2.20	0.55
1:T:13:DT:H2"	1:T:14:DA:C8	2.41	0.55
1:T:9:DG:H2"	1:T:10:DT:H5'	1.88	0.55
3:A:412:LYS:NZ	4:A:985:HOH:O	2.34	0.55
3:A:379:ARG:HD3	3:A:660:ASP:OD2	2.06	0.55
3:A:829:ARG:NH1	3:A:829:ARG:CG	2.58	0.55
3:A:645:GLY:O	3:A:649:GLN:HG3	2.05	0.55
3:A:166:VAL:HG13	3:A:173:ARG:HD2	1.89	0.55
3:A:232:GLN:HB2	3:A:241:SER:OG	2.07	0.55
1:T:5:DG:C5	3:A:237:VAL:HG13	2.42	0.54
3:A:486:HIS:HD2	3:A:518:TYR:OH	1.89	0.54
3:A:651:LEU:O	3:A:656:GLN:HB2	2.06	0.54
1:T:1:DT:H2"	1:T:2:DA:OP1	2.08	0.54
3:A:711:LYS:O	3:A:712:ASP:HB2	2.07	0.54
3:A:729:THR:HG22	3:A:733:PHE:CA	2.37	0.54
3:A:743:ILE:O	3:A:763:THR:HB	2.07	0.54
3:A:272:VAL:O	3:A:272:VAL:HG12	2.08	0.53
3:A:862:PRO:O	3:A:863:ALA:HB3	2.08	0.53
3:A:607:GLU:O	3:A:609:VAL:N	2.36	0.53
3:A:729:THR:HG22	3:A:733:PHE:O	2.09	0.53
3:A:738:GLU:O	3:A:738:GLU:HG3	2.08	0.53
3:A:562:LEU:HD21	3:A:870:LEU:HD12	1.90	0.53
3:A:19:ILE:O	3:A:23:THR:CG2	2.55	0.53
3:A:463:HIS:HD2	3:A:535:ALA:H	1.56	0.53
3:A:603:GLY:O	3:A:605:ILE:N	2.42	0.53
3:A:626:THR:O	3:A:630:THR:HG23	2.09	0.53
3:A:196:LEU:HB2	4:A:930:HOH:O	2.08	0.53
3:A:392:LYS:O	3:A:396:ILE:HG12	2.09	0.53

Continued on next page...

Continued from previous page...

Atom-1	Atom-2	Interatomic distance (Å)	Clash overlap (Å)
3:A:433:ASN:HB2	3:A:434:PRO:CD	2.39	0.53
2:N:115:DA:OP2	2:N:115:DA:H2'	2.09	0.53
3:A:185:VAL:CG2	3:A:277:PRO:HG3	2.39	0.52
3:A:367:ILE:O	3:A:374:LEU:HD22	2.09	0.52
3:A:269:GLN:NE2	3:A:407:LYS:HZ3	2.04	0.52
3:A:570:ILE:O	3:A:574:VAL:HG23	2.09	0.52
3:A:609:VAL:CG2	3:A:611:LEU:HG	2.38	0.52
3:A:609:VAL:HG11	3:A:669:GLN:OE1	2.09	0.52
3:A:35:GLU:OE2	3:A:272:VAL:HG21	2.10	0.52
3:A:32:LEU:HD22	3:A:272:VAL:CG1	2.40	0.52
3:A:403:GLU:CD	4:A:1208:HOH:O	2.47	0.52
3:A:562:LEU:HD21	3:A:870:LEU:CD1	2.39	0.52
3:A:701:SER:CB	3:A:861:MET:HG2	2.40	0.52
3:A:713:LYS:HA	3:A:713:LYS:HE2	1.91	0.52
3:A:199:GLU:OE1	3:A:199:GLU:HA	2.09	0.52
2:N:113:DC:C3'	2:N:115:DA:P	2.97	0.52
3:A:557:ARG:HB2	3:A:562:LEU:HD12	1.92	0.52
3:A:278:TRP:HE1	3:A:324:GLN:HE22	1.56	0.51
3:A:446:LEU:HD22	3:A:806:SER:HB3	1.91	0.51
3:A:452:ILE:HG23	3:A:453:GLY:N	2.25	0.51
3:A:454:LYS:HE3	3:A:455:GLU:N	2.24	0.51
3:A:871:ASN:OD1	3:A:873:ARG:HB2	2.10	0.51
3:A:362:MET:HE3	3:A:363:LYS:H	1.73	0.51
3:A:537:ASP:H	3:A:882:PHE:HD2	1.59	0.51
3:A:31:ARG:HD3	3:A:176:HIS:CE1	2.44	0.51
3:A:80:LYS:O	3:A:223:SER:HB2	2.11	0.51
3:A:335:LEU:HD23	3:A:335:LEU:O	2.11	0.51
1:T:1:DT:O4	3:A:738:GLU:N	2.40	0.51
3:A:816:THR:CG2	3:A:817:ILE:N	2.71	0.51
3:A:710:VAL:CG1	3:A:718:ILE:HG23	2.41	0.51
2:N:113:DC:C3'	2:N:114:DT:H3'	2.40	0.51
3:A:428:ALA:H	3:A:435:GLN:NE2	2.09	0.51
3:A:711:LYS:CG	3:A:712:ASP:H	2.15	0.50
3:A:729:THR:HG23	3:A:733:PHE:H	1.73	0.50
3:A:185:VAL:HG22	3:A:186:VAL:N	2.27	0.50
3:A:197:GLY:HA3	3:A:283:GLY:HA3	1.92	0.50
3:A:552:ASP:OD1	3:A:554:VAL:HG13	2.10	0.50
3:A:790:HIS:CD2	3:A:831:THR:HG23	2.47	0.50
3:A:371:PRO:HG2	3:A:373:ALA:CB	2.41	0.50
3:A:99:ARG:HD2	3:A:99:ARG:N	2.26	0.50
3:A:729:THR:OG1	3:A:789:SER:CB	2.60	0.50

Continued on next page...

Continued from previous page...

Atom-1	Atom-2	Interatomic distance (Å)	Clash overlap (Å)
1:T:9:DG:C2'	1:T:10:DT:H5"	2.41	0.50
3:A:176:HIS:O	3:A:177:VAL:O	2.29	0.50
3:A:371:PRO:C	3:A:373:ALA:N	2.65	0.50
3:A:501:TRP:HA	3:A:504:GLU:OE1	2.11	0.50
3:A:437:ASN:ND2	3:A:440:THR:H	2.10	0.49
3:A:446:LEU:HB2	3:A:531:SER:O	2.12	0.49
3:A:861:MET:O	3:A:862:PRO:O	2.30	0.49
3:A:655:ILE:HD12	3:A:674:ALA:HB2	1.95	0.49
3:A:717:GLU:HG2	3:A:718:ILE:N	2.28	0.49
3:A:6:ILE:O	3:A:7:ALA:C	2.51	0.49
3:A:308:TYR:HA	3:A:311:VAL:CG2	2.42	0.49
3:A:710:VAL:O	3:A:710:VAL:HG13	2.12	0.49
3:A:351:ASP:O	3:A:394:ARG:NH2	2.44	0.49
3:A:420:MET:HA	3:A:425:ARG:O	2.12	0.49
3:A:643:GLU:HA	4:A:1168:HOH:O	2.12	0.49
3:A:763:THR:CG2	3:A:764:ASN:N	2.75	0.49
3:A:106:LEU:HA	3:A:109:ILE:CD1	2.43	0.48
3:A:722:ARG:NH1	3:A:768:GLU:CD	2.67	0.48
3:A:710:VAL:HG12	3:A:720:ARG:O	2.13	0.48
3:A:789:SER:O	3:A:793:LYS:HG3	2.13	0.48
3:A:206:LYS:O	3:A:210:ILE:HG12	2.13	0.48
3:A:534:LEU:HD11	3:A:818:PRO:HG3	1.95	0.48
3:A:150:ARG:O	3:A:151:PHE:HB2	2.14	0.48
3:A:517:GLU:HG2	3:A:532:LEU:HB2	1.96	0.48
3:A:473:VAL:HG13	3:A:477:GLU:HB2	1.96	0.48
3:A:856:SER:O	3:A:857:GLN:C	2.52	0.48
3:A:364:PRO:C	3:A:366:ASP:N	2.66	0.47
2:N:108:DA:H1'	2:N:109:DC:H5"	1.95	0.47
3:A:155:ARG:NH1	3:A:155:ARG:CG	2.70	0.47
2:N:105:DA:N3	3:A:98:LYS:HE2	2.29	0.47
3:A:569:ASP:OD2	3:A:627:ARG:NE	2.48	0.47
3:A:360:LEU:HD11	3:A:385:TYR:OH	2.15	0.47
3:A:448:LYS:HG2	4:A:947:HOH:O	2.15	0.47
3:A:332:LYS:NZ	3:A:410:ASN:HD22	2.12	0.46
3:A:281:ILE:HG23	3:A:282:THR:CG2	2.22	0.46
3:A:324:GLN:NE2	3:A:418:TYR:H	2.01	0.46
1:T:1:DT:OP1	1:T:1:DT:H3'	2.16	0.46
3:A:833:VAL:O	3:A:837:GLU:HG3	2.14	0.46
3:A:269:GLN:HE21	3:A:404:GLN:HE22	1.63	0.46
3:A:724:ALA:HB1	3:A:737:GLN:O	2.16	0.46
2:N:112:DA:H1'	2:N:113:DC:H5"	1.96	0.46

Continued on next page...

Continued from previous page...

Atom-1	Atom-2	Interatomic distance (Å)	Clash overlap (Å)
3:A:335:LEU:HD21	3:A:406:ASN:OD1	2.14	0.46
3:A:573:ILE:O	3:A:577:LYS:HG3	2.16	0.46
3:A:590:THR:HB	3:A:613:THR:H	1.80	0.46
3:A:647:ARG:C	3:A:649:GLN:N	2.66	0.46
1:T:9:DG:H2"	1:T:10:DT:H5"	1.97	0.46
3:A:437:ASN:C	3:A:437:ASN:HD22	2.18	0.46
3:A:711:LYS:O	3:A:712:ASP:CB	2.62	0.46
3:A:649:GLN:HA	3:A:652:GLU:HG2	1.96	0.46
3:A:861:MET:N	3:A:862:PRO:CD	2.75	0.46
2:N:102:DA:H1'	2:N:103:DA:C8	2.51	0.46
3:A:711:LYS:HG2	3:A:712:ASP:N	2.24	0.45
3:A:364:PRO:O	3:A:366:ASP:N	2.49	0.45
3:A:722:ARG:HH11	3:A:768:GLU:CD	2.19	0.45
3:A:169:GLN:HG2	3:A:172:LYS:CD	2.46	0.45
3:A:84:ARG:HB2	3:A:223:SER:HB3	1.98	0.45
3:A:590:THR:HG22	3:A:591:ASP:N	2.31	0.45
3:A:553:GLU:CG	4:A:1176:HOH:O	2.65	0.45
3:A:593:GLU:O	3:A:594:VAL:O	2.35	0.45
3:A:609:VAL:HG11	3:A:669:GLN:CD	2.37	0.45
3:A:326:THR:HA	4:A:1161:HOH:O	2.16	0.45
3:A:729:THR:CG2	3:A:733:PHE:N	2.67	0.45
3:A:806:SER:O	3:A:816:THR:HG23	2.17	0.45
3:A:534:LEU:O	3:A:815:GLY:HA2	2.16	0.45
3:A:881:ALA:O	3:A:882:PHE:C	2.54	0.45
1:T:1:DT:O2	3:A:300:HIS:N	2.50	0.45
3:A:242:GLU:HG2	3:A:757:LEU:CD1	2.46	0.45
3:A:254:ILE:HD13	3:A:396:ILE:HD12	1.99	0.45
3:A:551:ARG:NH1	4:A:1148:HOH:O	2.45	0.45
3:A:851:ASP:CB	4:A:1059:HOH:O	2.64	0.45
3:A:36:GLN:HE22	3:A:272:VAL:H	1.65	0.44
3:A:465:ALA:HB2	3:A:482:ILE:CD1	2.47	0.44
3:A:729:THR:OG1	3:A:730:PRO:HD2	2.17	0.44
3:A:422:TRP:C	3:A:422:TRP:CD1	2.90	0.44
3:A:30:GLU:OE1	3:A:166:VAL:HG12	2.16	0.44
3:A:371:PRO:O	3:A:373:ALA:N	2.50	0.44
3:A:324:GLN:HG3	3:A:417:PRO:HA	1.99	0.44
3:A:545:HIS:O	3:A:549:MET:HG2	2.16	0.44
3:A:669:GLN:HA	3:A:669:GLN:NE2	2.31	0.44
3:A:177:VAL:HG21	4:A:1248:HOH:O	2.16	0.44
3:A:551:ARG:O	3:A:868:GLY:HA3	2.18	0.44
3:A:536:PHE:O	3:A:813:SER:HA	2.18	0.44

Continued on next page...

Continued from previous page...

Atom-1	Atom-2	Interatomic distance (Å)	Clash overlap (Å)
3:A:538:GLY:CA	3:A:883:ALA:HB3	2.47	0.44
3:A:729:THR:CG2	3:A:733:PHE:CB	2.93	0.44
1:T:7:DG:H2"	1:T:8:DA:OP2	2.17	0.44
3:A:473:VAL:CG2	3:A:474:PRO:HD2	2.32	0.44
1:T:4:DA:H4'	1:T:5:DG:OP2	2.18	0.44
3:A:8:LYS:O	3:A:9:ASN:CB	2.65	0.44
1:T:5:DG:C6	3:A:237:VAL:HG22	2.53	0.44
3:A:448:LYS:HE3	3:A:805:GLU:CB	2.47	0.43
3:A:281:ILE:HD11	3:A:305:LEU:O	2.18	0.43
3:A:419:ASN:HA	3:A:419:ASN:HD22	1.46	0.43
3:A:538:GLY:O	3:A:539:SER:O	2.35	0.43
3:A:74:ILE:HG13	3:A:74:ILE:O	2.18	0.43
3:A:606:SER:C	3:A:608:LYS:N	2.68	0.43
3:A:881:ALA:C	3:A:882:PHE:O	2.52	0.43
2:N:114:DT:P	2:N:114:DT:C3'	3.06	0.43
3:A:14:ILE:HG13	4:A:975:HOH:O	2.17	0.43
3:A:827:ALA:O	3:A:831:THR:HG22	2.18	0.43
3:A:308:TYR:CZ	3:A:736:TRP:HZ3	2.36	0.43
3:A:656:GLN:HB3	3:A:657:PRO:CD	2.49	0.43
3:A:182:PHE:CE1	3:A:448:LYS:HB3	2.54	0.43
3:A:189:ASP:CA	4:A:1116:HOH:O	2.65	0.43
3:A:761:ILE:HG13	3:A:761:ILE:O	2.18	0.43
2:N:113:DC:C2'	2:N:115:DA:OP2	2.66	0.43
3:A:146:GLU:HG3	3:A:204:TRP:CE2	2.54	0.43
3:A:269:GLN:HE21	3:A:404:GLN:NE2	2.16	0.43
3:A:454:LYS:HE3	3:A:455:GLU:HA	2.01	0.43
3:A:729:THR:HG22	3:A:733:PHE:HB3	1.99	0.43
3:A:463:HIS:CD2	3:A:534:LEU:HA	2.53	0.42
3:A:557:ARG:NE	4:A:1048:HOH:O	2.50	0.42
3:A:540:CYS:HB3	3:A:559:VAL:HG12	2.01	0.42
3:A:595:VAL:HA	3:A:608:LYS:CA	2.33	0.42
1:T:5:DG:C8	1:T:6:DT:H72	2.54	0.42
1:T:5:DG:N7	3:A:237:VAL:HG13	2.34	0.42
3:A:796:VAL:O	3:A:800:GLU:HG3	2.19	0.42
3:A:553:GLU:HA	3:A:870:LEU:HD13	2.01	0.42
3:A:274:PRO:HA	3:A:275:PRO:HD3	1.82	0.42
3:A:454:LYS:HE3	3:A:455:GLU:CA	2.49	0.42
3:A:106:LEU:HA	3:A:109:ILE:HD12	2.01	0.42
3:A:174:VAL:CG1	3:A:177:VAL:HG22	2.49	0.42
3:A:360:LEU:HD21	3:A:385:TYR:CZ	2.54	0.42
3:A:362:MET:CE	3:A:363:LYS:H	2.32	0.42

Continued on next page...

Continued from previous page...

Atom-1	Atom-2	Interatomic distance (Å)	Clash overlap (Å)
3:A:842:LEU:HA	3:A:842:LEU:HD12	1.82	0.42
3:A:448:LYS:HG3	4:A:894:HOH:O	2.20	0.42
3:A:463:HIS:CD2	3:A:535:ALA:H	2.36	0.42
3:A:154:ILE:O	3:A:154:ILE:CG2	2.67	0.42
3:A:651:LEU:HD23	3:A:651:LEU:C	2.40	0.42
3:A:182:PHE:HE2	3:A:412:LYS:HD3	1.83	0.42
3:A:19:ILE:CG2	3:A:20:PRO:CD	2.97	0.42
3:A:289:ASN:HA	3:A:289:ASN:HD22	1.59	0.42
3:A:154:ILE:HG22	3:A:154:ILE:O	2.18	0.41
3:A:486:HIS:HE1	4:A:1087:HOH:O	2.03	0.41
3:A:659:ILE:HA	3:A:663:LYS:O	2.20	0.41
3:A:726:HIS:HD2	3:A:735:VAL:O	2.03	0.41
3:A:370:ASN:N	3:A:371:PRO:CD	2.83	0.41
3:A:324:GLN:HE21	3:A:418:TYR:N	2.00	0.41
3:A:28:TYR:HA	3:A:183:MET:HE1	2.02	0.41
3:A:332:LYS:NZ	3:A:410:ASN:ND2	2.68	0.41
3:A:475:PHE:N	3:A:476:PRO:HD2	2.36	0.41
3:A:189:ASP:HA	4:A:1116:HOH:O	2.20	0.41
3:A:554:VAL:HG11	4:A:1319:HOH:O	2.20	0.41
3:A:596:THR:O	3:A:597:VAL:C	2.58	0.41
3:A:609:VAL:O	3:A:609:VAL:HG13	2.20	0.41
3:A:750:MET:HE2	3:A:753:GLY:C	2.40	0.41
3:A:186:VAL:HG12	3:A:187:GLU:N	2.36	0.41
3:A:27:HIS:O	3:A:28:TYR:CB	2.69	0.41
3:A:651:LEU:HD12	3:A:671:ASN:HA	2.03	0.41
3:A:150:ARG:NE	4:A:1244:HOH:O	2.52	0.41
3:A:308:TYR:HA	3:A:311:VAL:HG23	2.02	0.41
3:A:412:LYS:CA	3:A:412:LYS:HE2	2.48	0.41
3:A:27:HIS:O	3:A:28:TYR:HB2	2.21	0.41
3:A:76:THR:O	3:A:79:PRO:HG2	2.20	0.41
3:A:750:MET:HE1	3:A:755:PHE:O	2.21	0.41
3:A:763:THR:HG22	3:A:764:ASN:N	2.36	0.41
2:N:108:DA:H2”	2:N:109:DC:C5’	2.50	0.41
3:A:120:LYS:NZ	3:A:267:MET:SD	2.94	0.40
3:A:714:LYS:CA	3:A:714:LYS:HE2	2.33	0.40
1:T:1:DT:O2	3:A:299:THR:HA	2.21	0.40
1:T:2:DA:H1’	1:T:3:DT:C5’	2.47	0.40
3:A:648:GLN:HG3	4:A:993:HOH:O	2.21	0.40
3:A:9:ASN:O	3:A:11:PHE:N	2.55	0.40
3:A:846:TYR:C	3:A:848:GLN:H	2.24	0.40
3:A:816:THR:HG22	3:A:817:ILE:HG12	2.04	0.40

There are no symmetry-related clashes.

5.3 Torsion angles (i)

5.3.1 Protein backbone (i)

In the following table, the Percentiles column shows the percent Ramachandran outliers of the chain as a percentile score with respect to all X-ray entries followed by that with respect to entries of similar resolution.

The Analysed column shows the number of residues for which the backbone conformation was analysed, and the total number of residues.

Mol	Chain	Analysed	Favoured	Allowed	Outliers	Percentiles
3	A	858/883 (97%)	754 (88%)	65 (8%)	39 (4%)	2 2

All (39) Ramachandran outliers are listed below:

Mol	Chain	Res	Type
3	A	168	GLU
3	A	178	TYR
3	A	180	LYS
3	A	539	SER
3	A	594	VAL
3	A	597	VAL
3	A	600	GLU
3	A	604	GLU
3	A	606	SER
3	A	609	VAL
3	A	611	LEU
3	A	644	PHE
3	A	712	ASP
3	A	714	LYS
3	A	715	THR
3	A	854	HIS
3	A	857	GLN
3	A	862	PRO
3	A	169	GLN
3	A	598	THR
3	A	603	GLY
3	A	811	HIS
3	A	812	ASP
3	A	10	ASP
3	A	175	GLY

Continued on next page...

Continued from previous page...

Mol	Chain	Res	Type
3	A	197	GLY
3	A	372	GLU
3	A	608	LYS
3	A	853	LEU
3	A	855	GLU
3	A	863	ALA
3	A	9	ASN
3	A	177	VAL
3	A	262	ALA
3	A	601	ASN
3	A	370	ASN
3	A	559	VAL
3	A	595	VAL
3	A	625	VAL

5.3.2 Protein sidechains (i)

In the following table, the Percentiles column shows the percent sidechain outliers of the chain as a percentile score with respect to all X-ray entries followed by that with respect to entries of similar resolution.

The Analysed column shows the number of residues for which the sidechain conformation was analysed, and the total number of residues.

Mol	Chain	Analysed	Rotameric	Outliers	Percentiles
3	A	666/729 (91%)	633 (95%)	33 (5%)	24 40

All (33) residues with a non-rotameric sidechain are listed below:

Mol	Chain	Res	Type
3	A	23	THR
3	A	24	LEU
3	A	27	HIS
3	A	77	LEU
3	A	99	ARG
3	A	155	ARG
3	A	206	LYS
3	A	215	ARG
3	A	245	GLU
3	A	291	ARG
3	A	292	ARG
3	A	305	LEU

Continued on next page...

Continued from previous page...

Mol	Chain	Res	Type
3	A	403	GLU
3	A	404	GLN
3	A	412	LYS
3	A	419	ASN
3	A	422	TRP
3	A	423	ARG
3	A	437	ASN
3	A	454	LYS
3	A	459	TRP
3	A	473	VAL
3	A	514	PHE
3	A	517	GLU
3	A	554	VAL
3	A	573	ILE
3	A	616	LEU
3	A	654	THR
3	A	671	ASN
3	A	735	VAL
3	A	775	GLU
3	A	828	VAL
3	A	842	LEU

Some sidechains can be flipped to improve hydrogen bonding and reduce clashes. All (34) such sidechains are listed below:

Mol	Chain	Res	Type
3	A	22	ASN
3	A	27	HIS
3	A	36	GLN
3	A	86	ASN
3	A	107	GLN
3	A	165	ASN
3	A	171	ASN
3	A	184	GLN
3	A	230	HIS
3	A	239	GLN
3	A	269	GLN
3	A	289	ASN
3	A	300	HIS
3	A	321	ASN
3	A	324	GLN
3	A	410	ASN
3	A	411	HIS

Continued on next page...

Continued from previous page...

Mol	Chain	Res	Type
3	A	419	ASN
3	A	435	GLN
3	A	437	ASN
3	A	463	HIS
3	A	486	HIS
3	A	488	ASN
3	A	649	GLN
3	A	669	GLN
3	A	671	ASN
3	A	697	ASN
3	A	726	HIS
3	A	737	GLN
3	A	764	ASN
3	A	781	ASN
3	A	786	GLN
3	A	823	ASN
3	A	869	ASN

5.3.3 RNA (i)

There are no RNA molecules in this entry.

5.4 Non-standard residues in protein, DNA, RNA chains (i)

There are no non-standard protein/DNA/RNA residues in this entry.

5.5 Carbohydrates (i)

There are no carbohydrates in this entry.

5.6 Ligand geometry (i)

There are no ligands in this entry.

5.7 Other polymers (i)

There are no such residues in this entry.

5.8 Polymer linkage issues (i)

There are no chain breaks in this entry.

6 Fit of model and data (i)

6.1 Protein, DNA and RNA chains (i)

EDS was not executed - this section is therefore empty.

6.2 Non-standard residues in protein, DNA, RNA chains (i)

EDS was not executed - this section is therefore empty.

6.3 Carbohydrates (i)

EDS was not executed - this section is therefore empty.

6.4 Ligands (i)

EDS was not executed - this section is therefore empty.

6.5 Other polymers (i)

EDS was not executed - this section is therefore empty.
