# Supplementary material for: An engineered T7 RNA polymerase that produces mRNA free of immunostimulatory byproducts
Source: Nat Biotechnol. 2022 Nov 10;41(4):560–8. doi: 10.1038/s41587-022-01525-6 (PMC10110463; doi:10.1038/s41587-022-01525-6)
Supplement: Supplementary file 4 — Full validation report for PDB entry 1H38. [file 41587_2022_1525_MOESM4_ESM.pdf]

Full wwPDB X-ray Structure Validation Report (i)

May 27, 2020 – 02:57 am BST

PDB ID	:	1H38
Title	:	Structure of a T7 RNA polymerase elongation complex at 2.9A resolution
Authors	:	Tahirov, T.H.; Temyakov, D.; Anikin, M.; Patlan, V.; McAllister, W.T.; Vassylyev, D.G.; Yokoyama, S.
Deposited on	:	2002-08-24
Resolution	:	2.90 Å(reported)

This is a Full wwPDB X-ray Structure Validation Report for a publicly released PDB entry.

We welcome your comments at *validation@mail.wwpdb.org*
A user guide is available at
https://www.wwpdb.org/validation/2017/XrayValidationReportHelp
with specific help available everywhere you see the (i) symbol.

The following versions of software and data (see references (i)) were used in the production of this report:

MolProbity	:	4.02b-467
Xtriage (Phenix)	:	NOT EXECUTED
EDS	:	NOT EXECUTED
Percentile statistics	:	20191225.v01 (using entries in the PDB archive December 25th 2019)
Ideal geometry (proteins)	:	Engh & Huber (2001)
Ideal geometry (DNA, RNA)	:	Parkinson et al. (1996)
Validation Pipeline (wwPDB-VP)	:	2.11

1 Overall quality at a glance (i)

The following experimental techniques were used to determine the structure:
X-RAY DIFFRACTION

The reported resolution of this entry is 2.90 Å.

Percentile scores (ranging between 0-100) for global validation metrics of the entry are shown in the following graphic. The table shows the number of entries on which the scores are based.

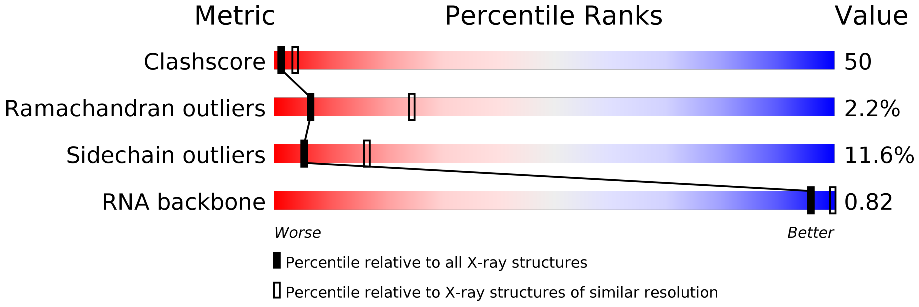

Metric	Whole archive (#Entries)	Similar resolution (#Entries, resolution range(Å))
Clashscore	141614	2172 (2.90-2.90)
Ramachandran outliers	138981	2115 (2.90-2.90)
Sidechain outliers	138945	2117 (2.90-2.90)
RNA backbone	3102	1007 (3.16-2.64)

The table below summarises the geometric issues observed across the polymeric chains and their fit to the electron density. The red, orange, yellow and green segments on the lower bar indicate the fraction of residues that contain outliers for >=3, 2, 1 and 0 types of geometric quality criteria respectively. A grey segment represents the fraction of residues that are not modelled. The numeric value for each fraction is indicated below the corresponding segment, with a dot representing fractions <=5%

Note EDS was not executed.

Mol	Chain	Length	Quality of chain
1	A	883	38% 50% 9% •
1	B	883	38% 49% 9% •
1	C	883	39% 49% 8% •
1	D	883	38% 51% 8% •
2	E	18	61% 28% 6% 6%
2	H	18	17% 39% 39% 6%
2	K	18	56% 39% 6%

Continued on next page...

Continued from previous page...

Mol	Chain	Length	Quality of chain
2	N	18	6% 61% 28% 6%
3	F	12	8% 50% 8% 33%
3	I	12	17% 50% 33%
3	L	12	25% 42% 33%
3	O	12	25% 42% 33%
4	G	10	10% 70% 10% 10%
4	J	10	20% 70% 10%
4	M	10	90% 10%
4	P	10	80% 10% 10%

2 Entry composition (i)

There are 5 unique types of molecules in this entry. The entry contains 30948 atoms, of which 0 are hydrogens and 0 are deuteriums.

In the tables below, the ZeroOcc column contains the number of atoms modelled with zero occupancy, the AltConf column contains the number of residues with at least one atom in alternate conformation and the Trace column contains the number of residues modelled with at most 2 atoms.

- Molecule 1 is a protein called DNA-DIRECTED RNA POLYMERASE.

Mol	Chain	Residues	Atoms					ZeroOcc	AltConf	Trace
1	A	857	Total 6746	C 4296	N 1173	O 1242	S 35	0	0	0
1	B	857	Total 6746	C 4296	N 1173	O 1242	S 35	0	0	0
1	C	857	Total 6746	C 4296	N 1173	O 1242	S 35	0	0	0
1	D	857	Total 6746	C 4296	N 1173	O 1242	S 35	0	0	0

- Molecule 2 is a DNA chain called 5'-D(*GP*GP*GP*AP*AP*TP*CP*GP*AP*CP *AP*TP*CP*GP*CP*CP*GP*C)-3'.

Mol	Chain	Residues	Atoms					ZeroOcc	AltConf	Trace
2	E	17	Total 345	C 164	N 67	O 98	P 16	0	0	0
2	H	18	Total 367	C 174	N 72	O 104	P 17	0	0	0
2	K	17	Total 345	C 164	N 67	O 98	P 16	0	0	0
2	N	17	Total 345	C 164	N 67	O 98	P 16	0	0	0

- Molecule 3 is a RNA chain called 5'-R(*AP*AP*CP*UP*GP*CP*GP*GP*CP*GP *AP*U)-3'.

Mol	Chain	Residues	Atoms					ZeroOcc	AltConf	Trace
3	F	8	Total 171	C 77	N 33	O 54	P 7	0	0	0
3	I	8	Total 171	C 77	N 33	O 54	P 7	0	0	0
3	L	8	Total 171	C 77	N 33	O 54	P 7	0	0	0

Continued on next page...

Continued from previous page...

Mol	Chain	Residues	Atoms					ZeroOcc	AltConf	Trace
3	O	8	Total 171	C 77	N 33	O 54	P 7	0	0	0

- Molecule 4 is a DNA chain called 5'-D(*GP*TP*CP*GP*AP*TP*TP*CP*CP*CP)-3'.

Mol	Chain	Residues	Atoms					ZeroOcc	AltConf	Trace
4	G	9	Total 179	C 87	N 30	O 54	P 8	0	0	0
4	J	9	Total 179	C 87	N 30	O 54	P 8	0	0	0
4	M	9	Total 179	C 87	N 30	O 54	P 8	0	0	0
4	P	9	Total 179	C 87	N 30	O 54	P 8	0	0	0

- Molecule 5 is water.

Mol	Chain	Residues	Atoms		ZeroOcc	AltConf
5	A	310	Total 310	O 310	0	0
5	B	352	Total 352	O 352	0	0
5	C	185	Total 185	O 185	0	0
5	D	177	Total 177	O 177	0	0
5	E	13	Total 13	O 13	0	0
5	F	16	Total 16	O 16	0	0
5	G	12	Total 12	O 12	0	0
5	H	20	Total 20	O 20	0	0
5	I	11	Total 11	O 11	0	0
5	J	9	Total 9	O 9	0	0
5	K	17	Total 17	O 17	0	0
5	L	5	Total 5	O 5	0	0

Continued on next page...

Continued from previous page...

Mol	Chain	Residues	Atoms	ZeroOcc	AltConf
5	M	8	Total O<br>8 8	0	0
5	N	12	Total O<br>12 12	0	0
5	O	6	Total O<br>6 6	0	0
5	P	9	Total O<br>9 9	0	0

3 Residue-property plots

These plots are drawn for all protein, RNA and DNA chains in the entry. The first graphic for a chain summarises the proportions of the various outlier classes displayed in the second graphic. The second graphic shows the sequence view annotated by issues in geometry. Residues are color-coded according to the number of geometric quality criteria for which they contain at least one outlier: green = 0, yellow = 1, orange = 2 and red = 3 or more. Stretches of 2 or more consecutive residues without any outlier are shown as a green connector. Residues present in the sample, but not in the model, are shown in grey.

Note EDS was not executed.

- Molecule 1: DNA-DIRECTED RNA POLYMERASE

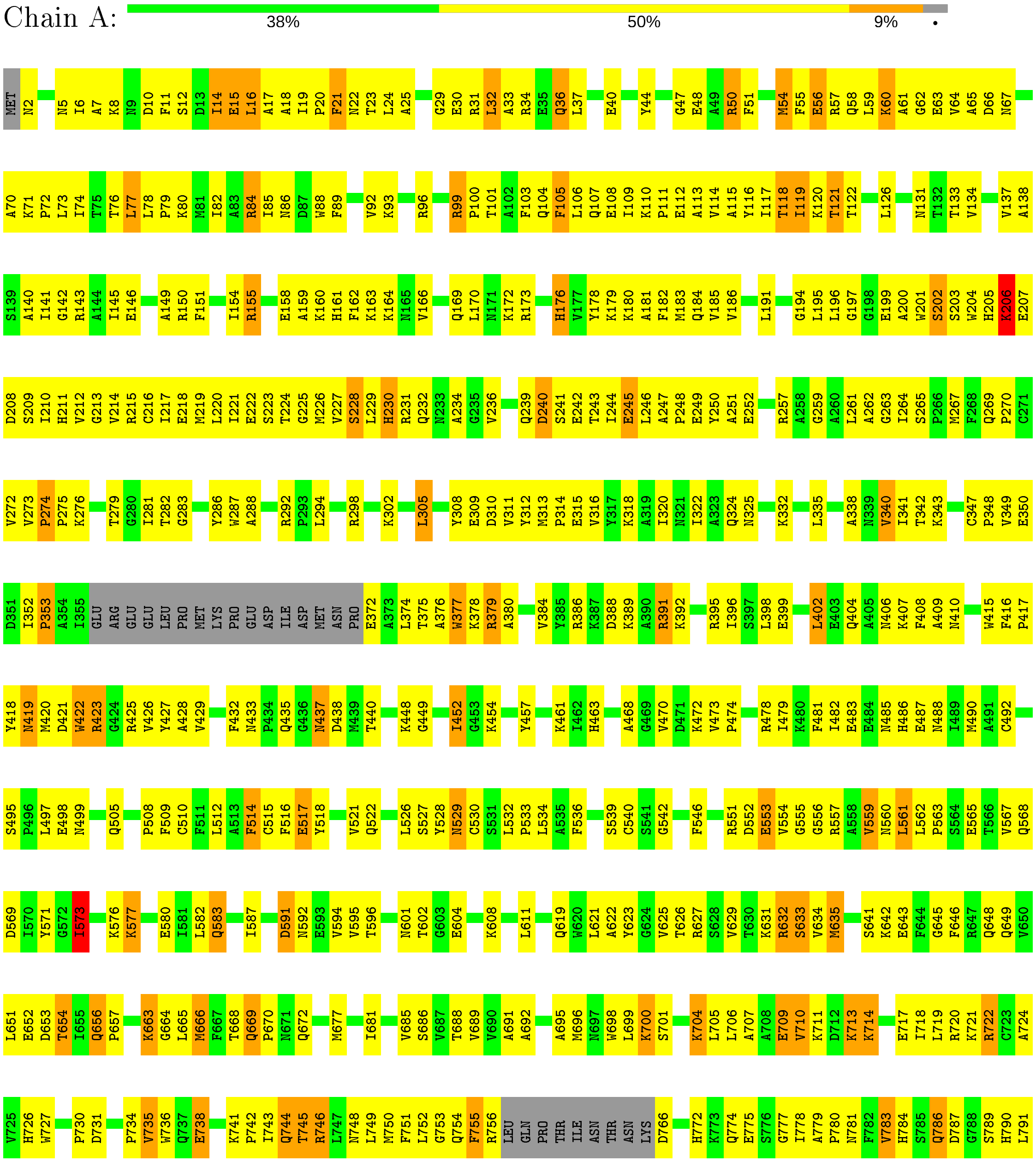

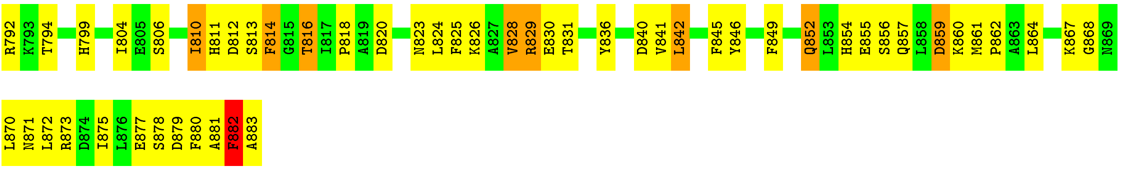

• Molecule 1: DNA-DIRECTED RNA POLYMERASE

Chain B:

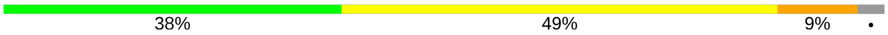

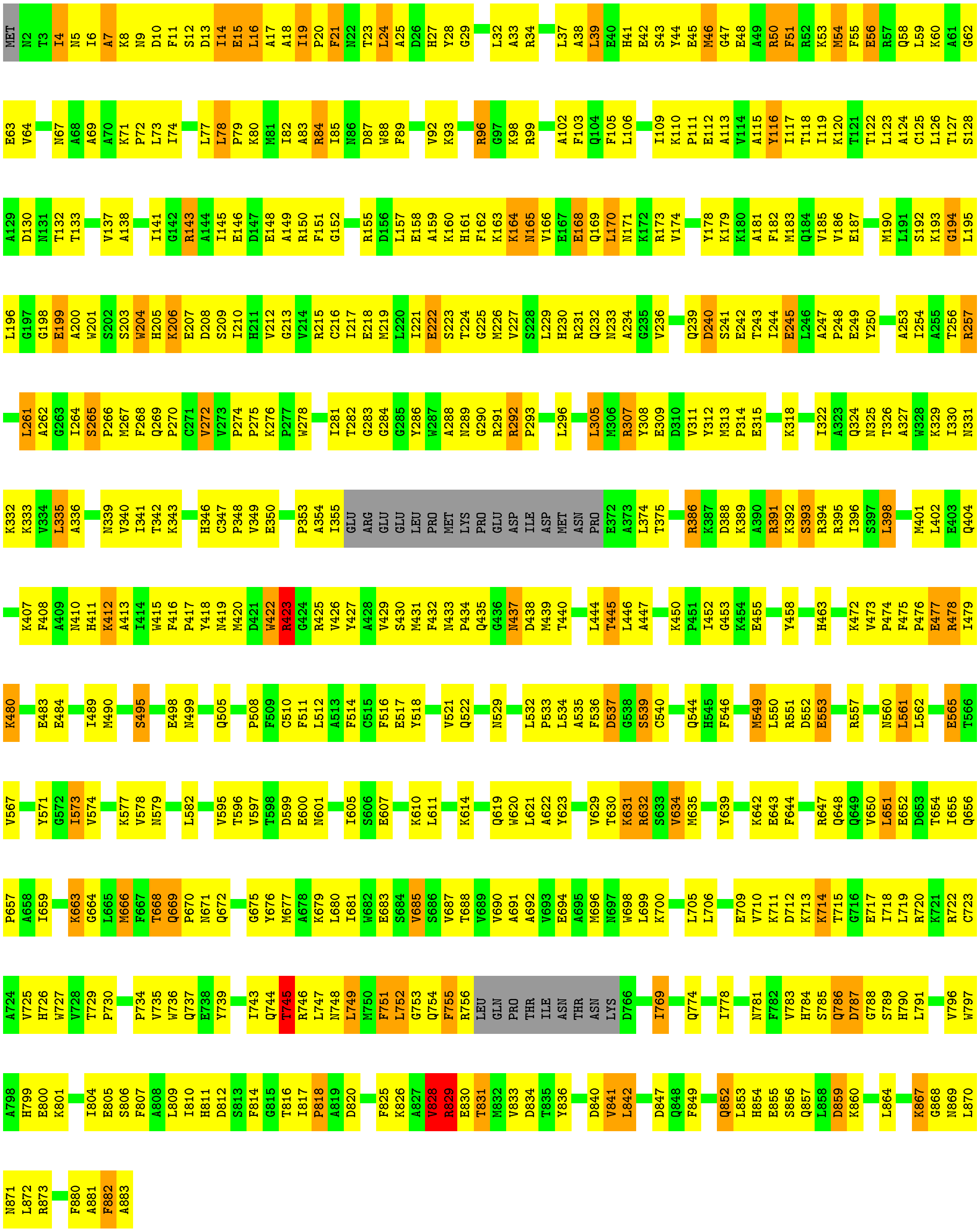

- Molecule 1: DNA-DIRECTED RNA POLYMERASE

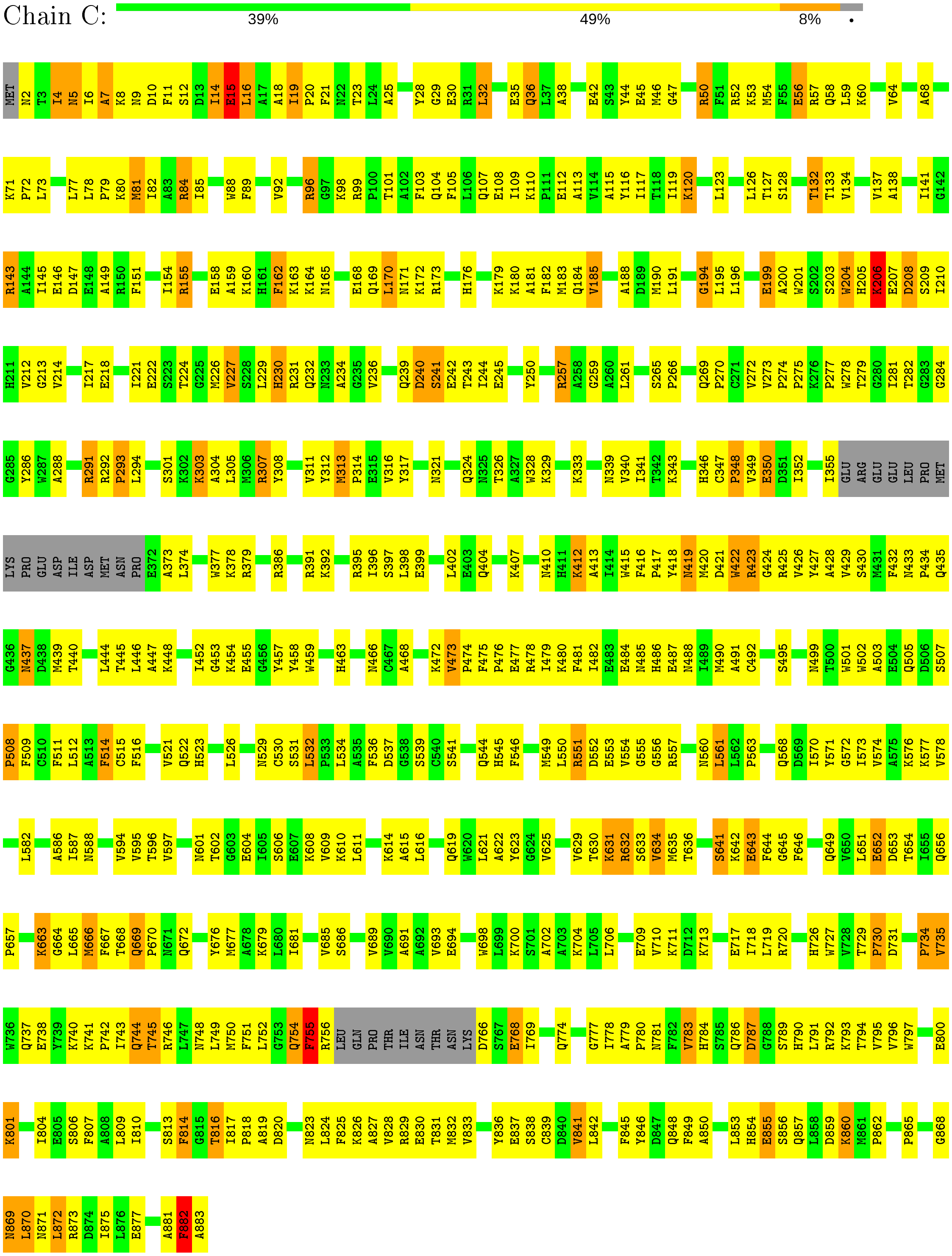

- Molecule 1: DNA-DIRECTED RNA POLYMERASE

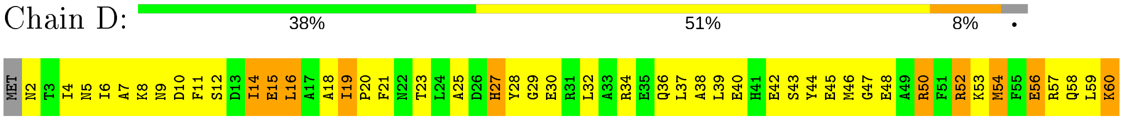

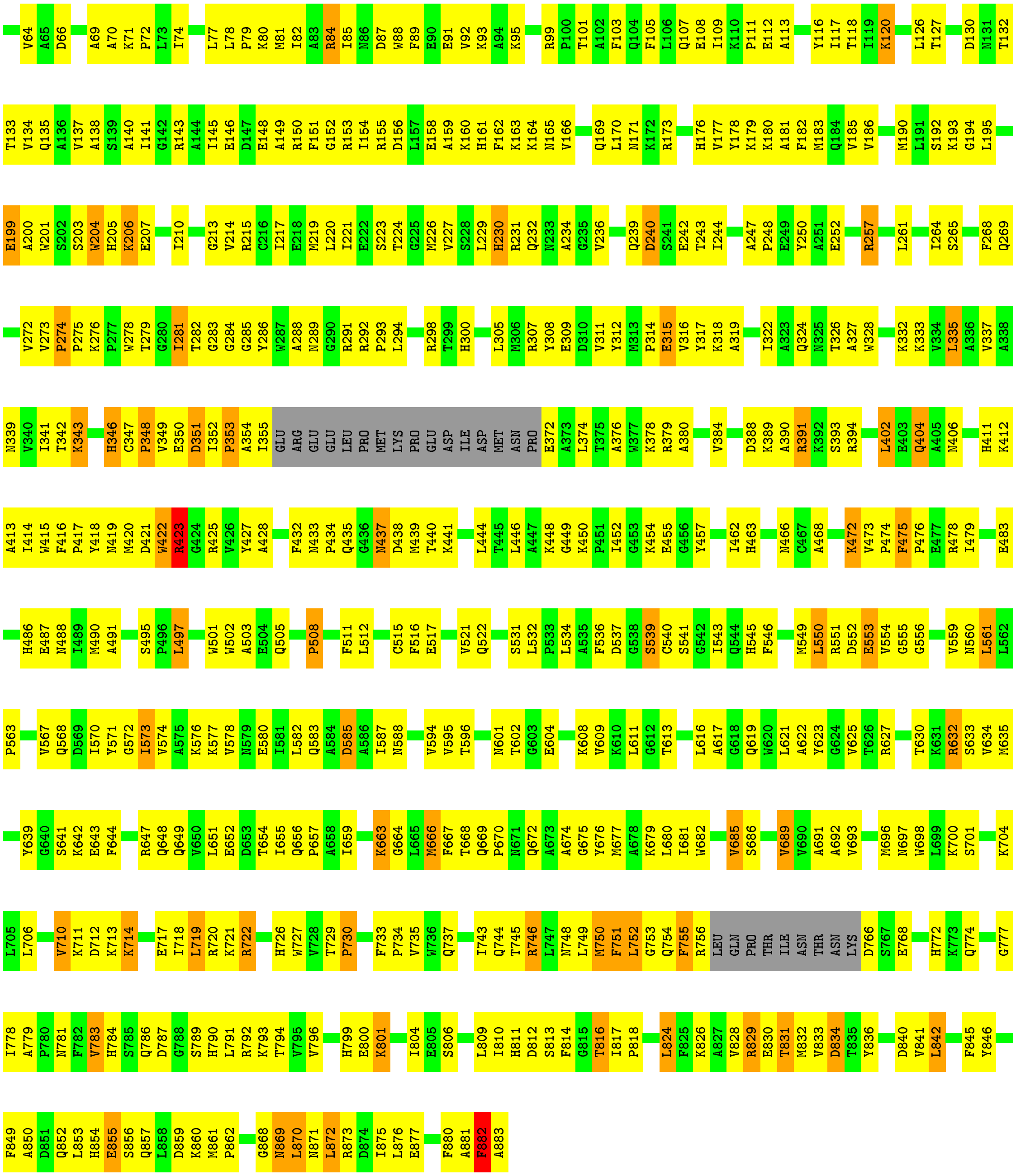

• Molecule 2: 5'-D(*GP*GP*GP*AP*AP*TP*CP*GP*AP*CP *AP*TP*CP*GP*CP*CP*GP*C)-3'

Chain E:
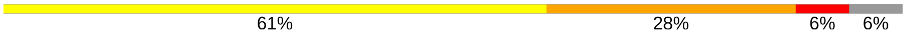

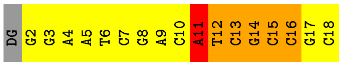

• Molecule 2: 5'-D(*GP*GP*GP*AP*AP*TP*CP*GP*AP*CP *AP*TP*CP*GP*CP*CP*GP*C)-3'

Chain H:
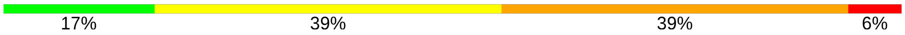

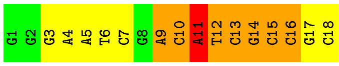

• Molecule 2: 5’-D(*GP*GP*GP*AP*AP*TP*CP*GP*AP*CP *AP*TP*CP*GP*CP*CP*GP*C)-3’

Chain K: 56% 39% 6%

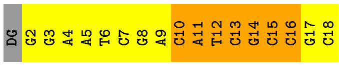

• Molecule 2: 5’-D(*GP*GP*GP*AP*AP*TP*CP*GP*AP*CP *AP*TP*CP*GP*CP*CP*GP*C)-3’

Chain N: 6% 61% 28% 6%

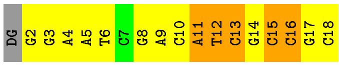

• Molecule 3: 5’-R(*AP*AP*CP*UP*GP*CP*GP*GP*CP*GP *AP*U)-3’

Chain F: 8% 50% 8% 33%

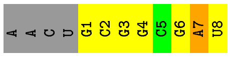

• Molecule 3: 5’-R(*AP*AP*CP*UP*GP*CP*GP*GP*CP*GP *AP*U)-3’

Chain I: 17% 50% 33%

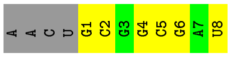

• Molecule 3: 5’-R(*AP*AP*CP*UP*GP*CP*GP*GP*CP*GP *AP*U)-3’

Chain L: 25% 42% 33%

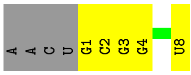

• Molecule 3: 5’-R(*AP*AP*CP*UP*GP*CP*GP*GP*CP*GP *AP*U)-3’

Chain O: 25% 42% 33%

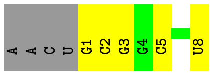

• Molecule 4: 5’-D(*GP*TP*CP*GP*AP*TP*TP*CP*CP*CP)-3’

Chain G: 10% 70% 10% 10%

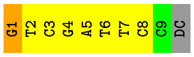

- Molecule 4: 5'-D(*GP*TP*CP*GP*AP*TP*TP*CP*CP*CP)-3'

Chain J:

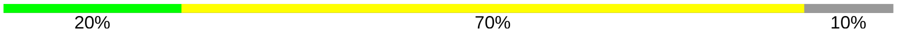

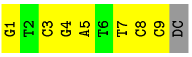

- Molecule 4: 5'-D(*GP*TP*CP*GP*AP*TP*TP*CP*CP*CP)-3'

Chain M:

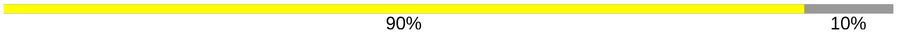

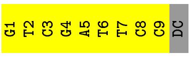

- Molecule 4: 5'-D(*GP*TP*CP*GP*AP*TP*TP*CP*CP*CP)-3'

Chain P:

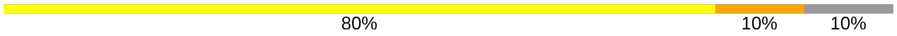

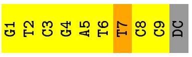

4 Data and refinement statistics ⓘ

Xtriage (Phenix) and EDS were not executed - this section is therefore incomplete.

Property	Value	Source
Space group	P 1	Depositor
Cell constants<br>a, b, c, α, β, γ	79.91Å 84.97Å 202.00Å<br>90.36° 92.97° 109.94°	Depositor
Resolution (Å)	39.93 – 2.90	Depositor
% Data completeness<br>(in resolution range)	98.0 (39.93-2.90)	Depositor
R_{merge}	0.08	Depositor
R_{sym}	(Not available)	Depositor
Refinement program	CNS 1.1	Depositor
R, R_{free}	0.236 , 0.284	Depositor
Estimated twinning fraction	No twinning to report.	Xtriage
Total number of atoms	30948	wwPDB-VP
Average B, all atoms (Å^2)	71.0	wwPDB-VP

5 Model quality (i)

5.1 Standard geometry (i)

The Z score for a bond length (or angle) is the number of standard deviations the observed value is removed from the expected value. A bond length (or angle) with $|Z| > 5$ is considered an outlier worth inspection. RMSZ is the root-mean-square of all Z scores of the bond lengths (or angles).

Mol	Chain	Bond lengths		Bond angles	
		RMSZ	#$\|Z\|$ >5	RMSZ	#$\|Z\|$ >5
1	A	0.89	9/6897 (0.1%)	0.85	5/9329 (0.1%)
1	B	0.93	2/6897 (0.0%)	0.87	9/9329 (0.1%)
1	C	0.64	0/6897	0.73	0/9329
1	D	0.58	0/6897	0.70	2/9329 (0.0%)
2	E	0.89	0/387	1.06	1/595 (0.2%)
2	H	0.94	0/412	1.03	1/634 (0.2%)
2	K	0.90	0/387	0.97	0/595
2	N	0.80	0/387	0.95	0/595
3	F	1.09	1/191 (0.5%)	0.85	0/297
3	I	0.94	0/191	0.81	0/297
3	L	0.81	0/191	0.79	0/297
3	O	0.61	0/191	0.74	0/297
4	G	0.88	0/199	0.84	0/305
4	J	0.77	0/199	0.93	0/305
4	M	0.68	0/199	0.85	0/305
4	P	0.74	0/199	1.05	0/305
All	All	0.78	12/30721 (0.0%)	0.81	18/42143 (0.0%)

Chiral center outliers are detected by calculating the chiral volume of a chiral center and verifying if the center is modelled as a planar moiety or with the opposite hand.A planarity outlier is detected by checking planarity of atoms in a peptide group, atoms in a mainchain group or atoms of a sidechain that are expected to be planar.

Mol	Chain	#Chirality outliers	#Planarity outliers
2	E	0	6
2	H	0	8
2	K	0	7
2	N	0	6
4	G	0	1
4	J	0	1
4	P	0	1
All	All	0	30

All (12) bond length outliers are listed below:

Mol	Chain	Res	Type	Atoms	Z	Observed(Å)	Ideal(Å)
1	A	510	CYS	CB-SG	-8.42	1.68	1.82
1	A	573	ILE	CA-CB	7.10	1.71	1.54
1	A	654	THR	CA-CB	5.53	1.67	1.53
1	A	783	VAL	CA-CB	-5.40	1.43	1.54
3	F	7	A	C5-C6	-5.31	1.36	1.41
1	B	828	VAL	CB-CG2	-5.30	1.41	1.52
1	B	805	GLU	CB-CG	-5.28	1.42	1.52
1	A	340	VAL	CB-CG2	-5.16	1.42	1.52
1	A	492	CYS	CB-SG	-5.12	1.73	1.81
1	A	783	VAL	CB-CG2	-5.09	1.42	1.52
1	A	709	GLU	CG-CD	5.01	1.59	1.51
1	A	426	VAL	CB-CG2	-5.00	1.42	1.52

All (18) bond angle outliers are listed below:

Mol	Chain	Res	Type	Atoms	Z	Observed(^{o})	Ideal(^{o})
1	B	537	ASP	CB-CG-OD1	-8.22	110.90	118.30
1	A	557	ARG	NE-CZ-NH2	-8.21	116.20	120.30
1	A	829	ARG	NE-CZ-NH1	-7.68	116.46	120.30
1	B	549	MET	CB-CG-SD	-6.86	91.82	112.40
1	A	787	ASP	CB-CG-OD2	-6.55	112.41	118.30
1	B	478	ARG	NE-CZ-NH2	6.00	123.30	120.30
1	D	550	LEU	CA-CB-CG	-6.00	101.51	115.30
1	B	829	ARG	NE-CZ-NH2	-5.95	117.33	120.30
1	A	419	ASN	CB-CA-C	-5.92	98.56	110.40
1	A	510	CYS	CA-CB-SG	-5.90	103.38	114.00
2	H	11	DA	N9-C1'-C2'	-5.88	101.42	112.60
1	B	423	ARG	NE-CZ-NH1	5.82	123.21	120.30
1	B	828	VAL	CB-CA-C	-5.44	101.07	111.40
1	B	24	LEU	CA-CB-CG	5.39	127.70	115.30
1	D	423	ARG	NE-CZ-NH1	-5.25	117.67	120.30
1	B	537	ASP	CB-CG-OD2	5.18	122.96	118.30
2	E	11	DA	N9-C1'-C2'	-5.13	102.86	112.60
1	B	78	LEU	CA-CB-CG	5.09	127.00	115.30

There are no chirality outliers.

All (30) planarity outliers are listed below:

Mol	Chain	Res	Type	Group
2	E	11	DA	Sidechain
2	E	12	DT	Sidechain

Continued on next page...

Continued from previous page...

Mol	Chain	Res	Type	Group
2	E	13	DC	Sidechain
2	E	14	DG	Sidechain
2	E	15	DC	Sidechain
2	E	16	DC	Sidechain
4	G	1	DG	Sidechain
2	H	10	DC	Sidechain
2	H	11	DA	Sidechain
2	H	12	DT	Sidechain
2	H	13	DC	Sidechain
2	H	14	DG	Sidechain
2	H	15	DC	Sidechain
2	H	16	DC	Sidechain
2	H	9	DA	Sidechain
4	J	7	DT	Sidechain
2	K	10	DC	Sidechain
2	K	11	DA	Sidechain
2	K	12	DT	Sidechain
2	K	13	DC	Sidechain
2	K	14	DG	Sidechain
2	K	15	DC	Sidechain
2	K	16	DC	Sidechain
2	N	11	DA	Sidechain
2	N	12	DT	Sidechain
2	N	13	DC	Sidechain
2	N	14	DG	Sidechain
2	N	15	DC	Sidechain
2	N	16	DC	Sidechain
4	P	7	DT	Sidechain

5.2 Too-close contacts (i)

In the following table, the Non-H and H(model) columns list the number of non-hydrogen atoms and hydrogen atoms in the chain respectively. The H(added) column lists the number of hydrogen atoms added and optimized by MolProbity. The Clashes column lists the number of clashes within the asymmetric unit, whereas Symm-Clashes lists symmetry related clashes.

Mol	Chain	Non-H	H(model)	H(added)	Clashes	Symm-Clashes
1	A	6746	0	6708	722	0
1	B	6746	0	6708	733	0
1	C	6746	0	6708	680	0
1	D	6746	0	6708	653	0
2	E	345	0	191	30	0

Continued on next page...

Continued from previous page...

Mol	Chain	Non-H	H(model)	H(added)	Clashes	Symm-Clashes
2	H	367	0	202	28	0
2	K	345	0	191	38	0
2	N	345	0	191	21	0
3	F	171	0	89	7	0
3	I	171	0	89	11	0
3	L	171	0	89	9	0
3	O	171	0	89	5	0
4	G	179	0	104	14	0
4	J	179	0	104	8	0
4	M	179	0	104	18	0
4	P	179	0	104	11	0
5	A	310	0	0	102	0
5	B	352	0	0	133	0
5	C	185	0	0	79	0
5	D	177	0	0	105	0
5	E	13	0	0	2	0
5	F	16	0	0	2	0
5	G	12	0	0	5	0
5	H	20	0	0	6	0
5	I	11	0	0	4	0
5	J	9	0	0	4	0
5	K	17	0	0	7	0
5	L	5	0	0	2	0
5	M	8	0	0	1	0
5	N	12	0	0	4	0
5	O	6	0	0	0	0
5	P	9	0	0	3	0
All	All	30948	0	28379	2933	0

The all-atom clashscore is defined as the number of clashes found per 1000 atoms (including hydrogen atoms). The all-atom clashscore for this structure is 50.

All (2933) close contacts within the same asymmetric unit are listed below, sorted by their clash magnitude.

Atom-1	Atom-2	Interatomic distance (Å)	Clash overlap (Å)
1:A:668:THR:HG22	1:A:669:GLN:NE2	1.51	1.24
1:C:428:ALA:H	1:C:435:GLN:NE2	1.44	1.15
1:B:50:ARG:HG2	1:B:50:ARG:HH11	1.01	1.14
1:C:133:THR:HA	1:C:243:THR:HG22	1.28	1.10
1:A:120:LYS:HE3	1:A:752:LEU:HD21	1.31	1.10
1:B:806:SER:O	1:B:816:THR:HG22	1.51	1.09

Continued on next page...

Continued from previous page...

Atom-1	Atom-2	Interatomic distance (Å)	Clash overlap (Å)
1:C:839:CYS:HB3	5:C:2179:HOH:O	1.49	1.09
1:A:562:LEU:HD21	1:A:870:LEU:HD11	1.11	1.08
1:B:663:LYS:HG2	1:B:664:GLY:H	1.15	1.07
1:B:647:ARG:HH22	1:B:671:ASN:ND2	1.53	1.06
1:A:669:GLN:HG2	1:A:672:GLN:HE21	1.14	1.06
1:B:330:ILE:HG22	5:B:2150:HOH:O	1.54	1.06
1:B:194:GLY:HA3	5:B:2099:HOH:O	1.55	1.06
1:A:269:GLN:HE22	1:A:407:LYS:NZ	1.54	1.05
4:G:5:DA:H2”	4:G:6:DT:H71	1.34	1.05
1:B:157:LEU:HB3	5:B:2089:HOH:O	1.55	1.04
1:C:536:PHE:HB3	1:C:882:PHE:HB3	1.36	1.04
1:B:291:ARG:HB2	5:B:2136:HOH:O	1.56	1.04
1:C:333:LYS:HB3	1:C:516:PHE:CE2	1.93	1.04
1:B:5:ASN:HB3	5:B:2024:HOH:O	1.59	1.03
1:A:562:LEU:HD21	1:A:870:LEU:CD1	1.89	1.02
1:A:669:GLN:HG2	1:A:672:GLN:NE2	1.74	1.02
1:C:347:CYS:SG	1:C:350:GLU:HG2	2.00	1.01
1:D:536:PHE:HB3	1:D:882:PHE:HB3	1.42	1.01
1:A:668:THR:HG22	1:A:669:GLN:HE22	1.04	1.01
1:D:351:ASP:HB2	5:D:2099:HOH:O	1.58	1.01
1:B:720:ARG:HH11	1:B:720:ARG:HG2	1.26	1.01
1:A:536:PHE:HB3	1:A:882:PHE:HB3	1.43	1.00
1:D:281:ILE:HG22	1:D:282:THR:HG23	1.42	1.00
1:A:594:VAL:HB	5:A:2236:HOH:O	1.60	0.99
1:B:80:LYS:HD2	1:B:224:THR:HG22	1.45	0.98
1:C:307:ARG:HH11	1:C:307:ARG:HG3	1.24	0.98
1:D:298:ARG:HH21	1:D:427:TYR:HB2	1.26	0.98
1:A:711:LYS:NZ	1:A:711:LYS:HB2	1.75	0.98
1:A:720:ARG:HG2	1:A:720:ARG:HH11	1.29	0.98
1:A:196:LEU:HA	5:A:2085:HOH:O	1.64	0.98
1:A:632:ARG:HB2	1:A:632:ARG:NH1	1.79	0.97
1:D:829:ARG:O	1:D:833:VAL:HG23	1.62	0.97
1:B:423:ARG:HE	2:H:12:DT:H4’	1.29	0.97
1:D:348:PRO:HG3	5:D:2092:HOH:O	1.62	0.97
1:D:663:LYS:HG2	1:D:664:GLY:H	1.27	0.97
1:D:681:ILE:O	1:D:685:VAL:HG22	1.64	0.97
1:C:457:TYR:CE1	1:C:521:VAL:HG11	1.98	0.97
1:C:806:SER:O	1:C:816:THR:HG23	1.65	0.97
1:A:668:THR:CG2	1:A:669:GLN:HE22	1.79	0.96
1:B:573:ILE:HD12	1:B:573:ILE:C	1.85	0.96
1:B:290:GLY:HA2	5:B:2134:HOH:O	1.64	0.96

Continued on next page...

Continued from previous page...

Atom-1	Atom-2	Interatomic distance (Å)	Clash overlap (Å)
1:A:92:VAL:HG12	1:A:99:ARG:HG3	1.44	0.96
1:B:623:TYR:HA	1:B:666:MET:HE2	1.47	0.96
1:A:99:ARG:HG2	1:A:99:ARG:HH11	1.27	0.95
1:D:275:PRO:HD2	5:D:2086:HOH:O	1.66	0.95
1:B:748:ASN:HD21	1:B:751:PHE:H	1.09	0.95
1:A:113:ALA:O	1:A:117:ILE:HG13	1.64	0.95
1:C:45:GLU:HG2	5:C:2038:HOH:O	1.65	0.95
1:A:93:LYS:HA	1:A:99:ARG:NH2	1.80	0.95
1:A:704:LYS:HE2	4:G:5:DA:OP1	1.66	0.95
1:A:342:THR:HG22	1:A:348:PRO:HG2	1.47	0.95
1:C:532:LEU:HD23	1:C:534:LEU:HD23	1.49	0.95
1:A:711:LYS:HZ3	1:A:711:LYS:HB2	1.25	0.94
1:C:422:TRP:HB3	5:C:2101:HOH:O	1.66	0.94
1:A:202:SER:HB2	5:A:2088:HOH:O	1.64	0.94
1:B:58:GLN:HG3	1:B:67:ASN:HD22	1.30	0.93
1:A:428:ALA:H	1:A:435:GLN:HE21	1.15	0.93
1:B:532:LEU:HD12	1:B:533:PRO:HD2	1.50	0.93
1:A:746:ARG:NH1	1:A:746:ARG:HB3	1.83	0.92
1:D:272:VAL:HA	5:D:2115:HOH:O	1.67	0.92
1:B:158:GLU:HG2	1:B:195:LEU:HD22	1.50	0.92
1:C:423:ARG:NH2	1:C:784:HIS:ND1	2.18	0.92
1:A:428:ALA:H	1:A:435:GLN:NE2	1.68	0.92
1:C:80:LYS:HD3	1:C:224:THR:HG22	1.52	0.92
1:B:59:LEU:HA	1:B:64:VAL:HG22	1.52	0.91
1:C:428:ALA:H	1:C:435:GLN:HE22	1.10	0.91
1:B:744:GLN:HA	1:B:756:ARG:HE	1.31	0.91
1:B:423:ARG:NE	2:H:12:DT:H4'	1.84	0.91
1:B:854:HIS:CD2	1:B:856:SER:H	1.88	0.91
1:A:751:PHE:HB3	1:A:752:LEU:HD12	1.52	0.91
1:C:485:ASN:HD22	1:C:488:ASN:HD22	1.09	0.91
1:D:560:ASN:O	1:D:881:ALA:HB2	1.70	0.91
1:A:562:LEU:CD2	1:A:870:LEU:HD11	2.00	0.91
1:D:806:SER:O	1:D:816:THR:HG23	1.72	0.90
2:K:2:DG:H2"	2:K:3:DG:H8	1.35	0.90
1:D:153:ARG:HH22	1:D:201:TRP:HE1	1.20	0.90
1:B:236:VAL:HG21	1:B:239:GLN:HE21	1.34	0.90
1:C:340:VAL:O	1:C:343:LYS:HG2	1.69	0.90
1:A:641:SER:HA	2:E:10:DC:H5'	1.53	0.89
1:B:647:ARG:HH22	1:B:671:ASN:HD22	1.01	0.89
1:D:155:ARG:HA	1:D:163:LYS:HD3	1.54	0.89
1:B:133:THR:HA	1:B:243:THR:HG22	1.54	0.89

Continued on next page...

Continued from previous page...

Atom-1	Atom-2	Interatomic distance (Å)	Clash overlap (Å)
1:D:633:SER:HA	1:D:649:GLN:HE22	1.37	0.89
1:D:794:THR:OG1	1:D:831:THR:HG21	1.73	0.89
2:E:2:DG:H2"	2:E:3:DG:N7	1.87	0.89
1:A:109:ILE:HD11	1:A:149:ALA:HB2	1.54	0.89
1:A:155:ARG:HB3	1:A:155:ARG:HH11	1.35	0.89
1:B:748:ASN:ND2	1:B:751:PHE:H	1.71	0.89
1:A:208:ASP:O	1:A:212:VAL:HG23	1.71	0.89
1:C:141:ILE:O	1:C:145:ILE:HG12	1.71	0.89
1:A:269:GLN:HE22	1:A:407:LYS:HZ3	1.15	0.89
1:C:16:LEU:H	1:C:16:LEU:HD23	1.38	0.88
1:C:147:ASP:HB3	1:C:750:MET:HE1	1.52	0.88
1:A:169:GLN:O	1:A:173:ARG:HG2	1.72	0.88
1:B:244:ILE:HB	5:B:2084:HOH:O	1.74	0.88
1:C:14:ILE:HG23	1:C:288:ALA:HB1	1.54	0.88
1:B:438:ASP:OD2	1:B:508:PRO:HD2	1.73	0.88
1:A:140:ALA:HA	5:A:2071:HOH:O	1.72	0.88
1:A:398:LEU:HD23	1:A:398:LEU:C	1.95	0.88
1:C:374:LEU:HD12	1:C:374:LEU:H	1.39	0.87
1:C:551:ARG:CG	1:C:551:ARG:HH11	1.88	0.87
1:C:560:ASN:O	1:C:881:ALA:HB2	1.72	0.87
1:D:751:PHE:HB3	1:D:752:LEU:HD12	1.54	0.87
1:A:155:ARG:HB3	1:A:155:ARG:NH1	1.88	0.87
1:B:560:ASN:O	1:B:881:ALA:HB2	1.73	0.87
1:C:720:ARG:HH11	1:C:720:ARG:HG2	1.40	0.87
1:B:18:ALA:HA	5:B:2040:HOH:O	1.74	0.87
1:C:281:ILE:HG22	1:C:282:THR:HG23	1.54	0.87
2:K:3:DG:H2"	2:K:4:DA:C8	2.08	0.87
1:C:549:MET:HB3	1:C:836:TYR:HE1	1.40	0.86
1:C:154:ILE:HG23	1:C:190:MET:HE1	1.54	0.86
1:B:607:GLU:HG2	5:B:2274:HOH:O	1.73	0.86
1:D:352:ILE:HA	5:D:2098:HOH:O	1.76	0.86
1:D:231:ARG:HD2	1:D:240:ASP:OD1	1.76	0.86
1:D:315:GLU:HA	1:D:315:GLU:OE2	1.72	0.86
1:A:100:PRO:HG2	1:A:103:PHE:HB2	1.56	0.86
1:C:870:LEU:HD23	1:C:872:LEU:HD23	1.58	0.86
1:C:481:PHE:HB2	5:C:2113:HOH:O	1.74	0.85
1:B:347:CYS:HB3	1:B:350:GLU:HG3	1.56	0.85
1:C:169:GLN:HB3	5:C:2062:HOH:O	1.74	0.85
1:B:546:PHE:HD1	1:B:549:MET:HE3	1.39	0.85
1:B:790:HIS:CD2	1:B:831:THR:CG2	2.59	0.85
1:D:43:SER:HA	1:D:46:MET:HE2	1.56	0.85

Continued on next page...

Continued from previous page...

Atom-1	Atom-2	Interatomic distance (Å)	Clash overlap (Å)
1:A:59:LEU:HA	1:A:64:VAL:HG22	1.59	0.85
1:A:730:PRO:HD3	1:A:786:GLN:HE22	1.41	0.85
1:C:164:LYS:N	1:C:164:LYS:HE2	1.90	0.85
1:A:84:ARG:HG3	1:A:223:SER:HB3	1.55	0.85
1:B:146:GLU:OE2	1:B:201:TRP:HB3	1.77	0.85
1:A:195:LEU:HD11	5:A:2076:HOH:O	1.76	0.84
1:B:59:LEU:HD23	1:B:64:VAL:HG21	1.59	0.84
1:B:715:THR:HB	5:B:2302:HOH:O	1.75	0.84
2:K:3:DG:H3’	5:K:2007:HOH:O	1.76	0.84
1:B:152:GLY:HA2	5:B:2088:HOH:O	1.75	0.84
1:B:208:ASP:O	1:B:212:VAL:HG23	1.77	0.84
1:B:43:SER:OG	1:B:269:GLN:HG3	1.76	0.84
1:C:826:LYS:O	1:C:830:GLU:HG3	1.77	0.84
1:C:849:PHE:HD2	1:C:853:LEU:HD21	1.41	0.84
1:A:854:HIS:CD2	1:A:856:SER:H	1.96	0.84
1:B:249:GLU:HB2	5:B:2120:HOH:O	1.76	0.84
1:A:126:LEU:HB3	5:A:2070:HOH:O	1.76	0.84
1:B:579:ASN:HA	1:B:582:LEU:HD12	1.57	0.84
1:B:790:HIS:CD2	1:B:831:THR:HG23	2.13	0.84
1:D:146:GLU:OE2	1:D:201:TRP:HB3	1.78	0.84
1:D:632:ARG:HG2	5:N:2010:HOH:O	1.77	0.84
1:A:560:ASN:O	1:A:881:ALA:HB2	1.78	0.84
1:C:428:ALA:N	1:C:435:GLN:NE2	2.26	0.84
1:D:438:ASP:OD2	1:D:508:PRO:HG2	1.78	0.84
1:B:332:LYS:HG2	5:B:2152:HOH:O	1.77	0.83
1:D:729:THR:HG23	1:D:733:PHE:O	1.78	0.83
1:B:123:LEU:HD11	5:B:2082:HOH:O	1.78	0.83
1:A:143:ARG:HB3	5:A:2071:HOH:O	1.77	0.83
1:A:92:VAL:CG1	1:A:99:ARG:HG3	2.08	0.83
1:B:120:LYS:HD2	1:B:752:LEU:HD21	1.58	0.83
1:B:113:ALA:O	1:B:117:ILE:HG13	1.77	0.83
1:A:24:LEU:HD21	1:A:287:TRP:CD2	2.14	0.83
1:D:153:ARG:NH2	1:D:201:TRP:HE1	1.76	0.83
1:D:582:LEU:HB3	1:D:621:LEU:HD21	1.61	0.83
1:A:724:ALA:HB2	1:A:738:GLU:HG3	1.61	0.83
1:B:281:ILE:HG22	1:B:282:THR:HG23	1.58	0.83
1:B:50:ARG:HG2	1:B:50:ARG:NH1	1.74	0.83
1:C:485:ASN:ND2	1:C:488:ASN:HD22	1.76	0.83
1:D:347:CYS:SG	1:D:350:GLU:HG2	2.19	0.83
1:D:11:PHE:CZ	1:D:44:TYR:HB3	2.13	0.82
1:A:347:CYS:HB3	1:A:350:GLU:HG3	1.60	0.82

Continued on next page...

Continued from previous page...

Atom-1	Atom-2	Interatomic distance (Å)	Clash overlap (Å)
1:A:560:ASN:HD21	1:A:567:VAL:HG13	1.43	0.82
1:A:641:SER:CA	2:E:10:DC:H5’	2.09	0.82
1:A:398:LEU:HD23	1:A:398:LEU:O	1.80	0.82
4:M:1:DG:H1’	4:M:2:DT:H71	1.60	0.82
1:A:632:ARG:HD3	5:A:2249:HOH:O	1.78	0.82
1:A:744:GLN:O	1:A:745:THR:HG23	1.79	0.82
1:C:423:ARG:HE	1:C:781:ASN:ND2	1.78	0.82
1:D:585:ASP:OD2	1:D:613:THR:HB	1.79	0.82
1:B:169:GLN:O	1:B:173:ARG:HG2	1.80	0.82
1:D:587:ILE:HG13	5:D:2136:HOH:O	1.80	0.82
3:L:1:G:H5”	5:L:2001:HOH:O	1.78	0.82
1:A:705:LEU:HD22	1:A:857:GLN:HB2	1.62	0.82
1:B:329:LYS:HD3	1:B:447:ALA:HA	1.60	0.82
1:B:769:ILE:HG12	5:B:2306:HOH:O	1.80	0.82
1:D:292:ARG:HG3	1:D:292:ARG:O	1.77	0.82
1:A:425:ARG:HD3	1:A:811:HIS:CD2	2.14	0.82
1:A:100:PRO:HB3	5:A:2064:HOH:O	1.78	0.81
1:D:486:HIS:O	1:D:490:MET:HG2	1.80	0.81
1:D:573:ILE:HA	1:D:576:LYS:HD3	1.62	0.81
1:D:711:LYS:HG2	1:D:718:ILE:HA	1.62	0.81
1:A:217:ILE:O	1:A:221:ILE:HG13	1.80	0.81
1:A:89:PHE:HA	1:A:103:PHE:HE1	1.45	0.81
1:C:769:ILE:HG21	5:C:2154:HOH:O	1.78	0.81
1:A:226:MET:HG3	1:A:250:TYR:HD1	1.45	0.81
1:D:116:TYR:OH	1:D:752:LEU:HD22	1.79	0.81
1:D:155:ARG:HB2	1:D:163:LYS:HE2	1.63	0.81
1:A:18:ALA:O	1:A:19:ILE:HG12	1.80	0.81
1:B:401:MET:HA	1:B:401:MET:HE3	1.61	0.81
1:B:751:PHE:HB3	1:B:752:LEU:HD12	1.61	0.81
1:D:339:ASN:O	1:D:343:LYS:HD2	1.81	0.81
1:A:379:ARG:HA	5:A:2149:HOH:O	1.80	0.81
1:B:236:VAL:HG21	1:B:239:GLN:NE2	1.94	0.81
1:B:16:LEU:H	1:B:16:LEU:HD23	1.46	0.81
1:B:206:LYS:O	1:B:210:ILE:HG12	1.81	0.81
1:B:647:ARG:NH2	1:B:671:ASN:ND2	2.29	0.80
1:C:530:CYS:SG	1:C:818:PRO:HG2	2.20	0.80
1:C:751:PHE:HB3	1:C:752:LEU:HD12	1.62	0.80
1:D:206:LYS:O	1:D:210:ILE:HG12	1.81	0.80
1:D:744:GLN:HA	1:D:756:ARG:HH11	1.45	0.80
1:A:120:LYS:HE3	1:A:752:LEU:CD2	2.10	0.80
1:A:30:GLU:HG2	1:A:34:ARG:CZ	2.09	0.80

Continued on next page...

Continued from previous page...

Atom-1	Atom-2	Interatomic distance (Å)	Clash overlap (Å)
1:B:546:PHE:CD1	1:B:549:MET:HE3	2.16	0.80
1:D:332:LYS:HE2	5:D:2011:HOH:O	1.80	0.80
1:D:648:GLN:O	1:D:652:GLU:HG2	1.82	0.80
1:C:11:PHE:HB2	5:C:2018:HOH:O	1.80	0.80
1:C:711:LYS:NZ	1:C:711:LYS:HB2	1.96	0.80
1:D:91:GLU:O	1:D:95:LYS:HD2	1.82	0.80
1:A:315:GLU:OE2	1:A:318:LYS:HD3	1.82	0.80
1:D:154:ILE:HG23	1:D:190:MET:HE1	1.63	0.80
1:B:59:LEU:HD23	1:B:64:VAL:CG2	2.12	0.80
1:A:421:ASP:OD2	1:A:427:TYR:HE1	1.65	0.80
1:B:730:PRO:CD	1:B:786:GLN:HE22	1.95	0.80
1:C:229:LEU:HD12	1:C:243:THR:O	1.82	0.80
1:C:551:ARG:HG3	1:C:551:ARG:HH11	1.45	0.80
1:B:7:ALA:HB3	5:B:2024:HOH:O	1.81	0.79
2:H:6:DT:H1’	5:H:2008:HOH:O	1.81	0.79
1:A:134:VAL:HB	1:A:244:ILE:HD11	1.64	0.79
2:E:8:DG:H5’	5:E:2005:HOH:O	1.82	0.79
1:A:746:ARG:HH11	1:A:746:ARG:HB3	1.47	0.79
1:C:452:ILE:HD11	1:C:457:TYR:HA	1.64	0.79
1:C:50:ARG:HH11	1:C:50:ARG:HG2	1.45	0.79
1:B:711:LYS:HA	1:B:719:LEU:HD13	1.64	0.79
1:C:181:ALA:O	1:C:185:VAL:HG22	1.82	0.79
1:C:428:ALA:N	1:C:435:GLN:HE22	1.79	0.79
1:D:450:LYS:HD3	5:D:2119:HOH:O	1.82	0.79
1:A:155:ARG:O	1:A:155:ARG:HD2	1.83	0.79
1:A:804:ILE:HG23	1:A:816:THR:HG21	1.63	0.79
1:B:714:LYS:NZ	1:B:714:LYS:HA	1.98	0.79
1:C:468:ALA:HB2	1:C:511:PHE:CE1	2.18	0.79
1:D:570:ILE:O	1:D:574:VAL:HG23	1.82	0.79
1:D:84:ARG:HD3	1:D:84:ARG:C	2.03	0.79
1:A:608:LYS:HG2	5:A:2240:HOH:O	1.81	0.79
1:C:147:ASP:HB3	1:C:750:MET:CE	2.13	0.78
1:A:495:SER:HB3	1:A:498:GLU:HG3	1.65	0.78
1:B:829:ARG:HH11	1:B:829:ARG:HG3	1.47	0.78
1:C:19:ILE:HG21	5:C:2027:HOH:O	1.83	0.78
1:C:455:GLU:HA	5:C:2110:HOH:O	1.81	0.78
1:B:790:HIS:HD2	1:B:831:THR:HG23	1.48	0.78
1:C:663:LYS:HG2	1:C:664:GLY:H	1.47	0.78
1:A:648:GLN:NE2	1:A:652:GLU:OE1	2.16	0.78
1:B:489:ILE:HG21	1:B:518:TYR:CD1	2.19	0.78
1:A:51:PHE:CZ	1:A:261:LEU:HD23	2.19	0.78

Continued on next page...

Continued from previous page...

Atom-1	Atom-2	Interatomic distance (Å)	Clash overlap (Å)
1:B:84:ARG:HD2	1:B:219:MET:HB3	1.64	0.78
1:D:109:ILE:HD12	1:D:109:ILE:H	1.49	0.78
1:C:84:ARG:NE	1:C:222:GLU:OE1	2.16	0.77
1:B:663:LYS:HG2	1:B:664:GLY:N	1.97	0.77
1:A:423:ARG:HE	1:A:781:ASN:HD22	1.31	0.77
1:C:236:VAL:HB	1:C:239:GLN:HB2	1.66	0.77
1:A:281:ILE:HG22	1:A:282:THR:HG23	1.66	0.77
1:A:372:GLU:HG3	5:A:2014:HOH:O	1.83	0.77
1:D:497:LEU:HD12	5:D:2125:HOH:O	1.84	0.77
2:K:2:DG:H2”	2:K:3:DG:C8	2.19	0.77
1:B:537:ASP:O	1:B:882:PHE:HB2	1.84	0.77
1:C:871:ASN:HA	5:C:2183:HOH:O	1.82	0.77
1:D:355:ILE:HA	5:D:2102:HOH:O	1.82	0.77
1:D:82:ILE:HD13	1:D:112:GLU:OE2	1.84	0.77
1:B:257:ARG:HG2	5:B:2125:HOH:O	1.85	0.77
1:D:219:MET:HE2	5:D:2030:HOH:O	1.85	0.77
1:D:572:GLY:O	1:D:576:LYS:HG3	1.85	0.77
1:D:103:PHE:HB2	5:D:2029:HOH:O	1.83	0.77
2:N:5:DA:H1’	2:N:6:DT:H5’	1.67	0.77
1:A:88:TRP:CH2	1:A:100:PRO:HG3	2.20	0.77
1:A:14:ILE:HG23	1:A:288:ALA:HB1	1.67	0.76
1:B:96:ARG:HG3	5:B:2031:HOH:O	1.85	0.76
1:C:210:ILE:O	1:C:214:VAL:HG23	1.85	0.76
4:M:4:DG:H4’	4:M:5:DA:OP1	1.84	0.76
1:D:744:GLN:HG2	1:D:756:ARG:HD3	1.67	0.76
1:A:122:THR:HG22	1:A:126:LEU:HD12	1.66	0.76
1:A:88:TRP:HH2	1:A:100:PRO:HG3	1.50	0.76
1:B:151:PHE:CD1	1:B:183:MET:HB3	2.20	0.76
1:B:150:ARG:HD3	1:B:151:PHE:CE2	2.20	0.76
1:B:137:VAL:HG12	1:B:141:ILE:HD11	1.68	0.76
1:D:16:LEU:HD13	1:D:38:ALA:HB2	1.68	0.76
1:A:632:ARG:CZ	1:A:632:ARG:HB2	2.15	0.76
1:B:50:ARG:HH11	1:B:50:ARG:CG	1.89	0.76
1:A:197:GLY:HA2	5:A:2087:HOH:O	1.84	0.76
1:B:159:ALA:HB1	1:B:163:LYS:N	1.99	0.76
1:D:16:LEU:HA	1:D:37:LEU:HD12	1.67	0.76
1:D:853:LEU:HD22	1:D:857:GLN:HG3	1.66	0.76
1:A:217:ILE:HG22	1:A:221:ILE:HD11	1.68	0.76
1:A:281:ILE:HD11	1:A:308:TYR:HB3	1.67	0.76
1:B:853:LEU:HD22	1:B:857:GLN:HG3	1.66	0.76
1:D:34:ARG:HB3	5:D:2022:HOH:O	1.84	0.76

Continued on next page...

Continued from previous page...

Atom-1	Atom-2	Interatomic distance (Å)	Clash overlap (Å)
1:A:219:MET:SD	5:A:2060:HOH:O	2.43	0.76
1:B:692:ALA:O	1:B:696:MET:HG3	1.85	0.76
1:D:829:ARG:HG3	1:D:829:ARG:HH11	1.50	0.76
1:B:748:ASN:HD21	1:B:751:PHE:N	1.83	0.75
1:C:231:ARG:HG2	1:C:234:ALA:HB2	1.65	0.75
1:C:794:THR:OG1	1:C:831:THR:HG21	1.86	0.75
1:A:143:ARG:HA	5:A:2073:HOH:O	1.86	0.75
1:A:724:ALA:CB	1:A:738:GLU:HG3	2.16	0.75
1:A:150:ARG:NH1	5:A:2075:HOH:O	2.20	0.75
1:A:720:ARG:HG2	1:A:720:ARG:NH1	2.02	0.75
1:C:738:GLU:OE2	5:C:2158:HOH:O	2.03	0.75
5:C:2147:HOH:O	4:M:1:DG:H4’	1.85	0.75
1:D:314:PRO:HA	5:D:2084:HOH:O	1.86	0.75
1:B:309:GLU:HG2	5:B:2142:HOH:O	1.86	0.75
1:B:79:PRO:HB3	5:B:2071:HOH:O	1.85	0.75
1:C:437:ASN:H	1:C:437:ASN:HD22	1.34	0.75
1:B:50:ARG:NH2	1:B:267:MET:HG2	2.00	0.75
1:D:307:ARG:HD2	5:D:2081:HOH:O	1.85	0.75
1:A:84:ARG:HE	1:A:222:GLU:HB2	1.52	0.75
1:B:236:VAL:HB	1:B:239:GLN:HB2	1.67	0.75
1:D:298:ARG:NH2	1:D:427:TYR:HB2	2.01	0.75
2:E:4:DA:H2”	2:E:5:DA:C8	2.21	0.75
1:A:325:ASN:HB3	5:A:2134:HOH:O	1.86	0.75
1:A:60:LYS:HG2	1:A:60:LYS:O	1.84	0.75
1:A:882:PHE:CD1	1:A:882:PHE:N	2.52	0.75
1:D:126:LEU:HD21	1:D:244:ILE:HG22	1.69	0.75
1:A:452:ILE:HD12	1:A:818:PRO:HB2	1.68	0.75
1:A:669:GLN:HE21	1:A:669:GLN:N	1.85	0.75
1:D:479:ILE:O	1:D:483:GLU:HG3	1.87	0.75
1:A:592:ASN:OD1	1:A:611:LEU:HA	1.87	0.74
1:B:473:VAL:HG11	1:B:477:GLU:HB3	1.69	0.74
1:B:679:LYS:HG2	5:B:2299:HOH:O	1.86	0.74
1:C:78:LEU:O	1:C:82:ILE:HG13	1.87	0.74
1:D:402:LEU:HG	1:D:439:MET:HE1	1.69	0.74
1:A:631:LYS:O	1:A:635:MET:HG2	1.87	0.74
1:C:422:TRP:HE3	5:C:2101:HOH:O	1.69	0.74
1:B:106:LEU:HD21	1:B:212:VAL:CG1	2.17	0.74
1:C:549:MET:HB3	1:C:836:TYR:CE1	2.23	0.74
1:A:269:GLN:HA	5:A:2113:HOH:O	1.85	0.74
1:A:347:CYS:SG	1:A:350:GLU:HG2	2.28	0.74
1:A:591:ASP:OD2	1:A:591:ASP:N	2.20	0.74

Continued on next page...

Continued from previous page...

Atom-1	Atom-2	Interatomic distance (Å)	Clash overlap (Å)
1:B:712:ASP:HB3	5:B:2302:HOH:O	1.87	0.74
1:B:882:PHE:O	1:B:883:ALA:HB3	1.87	0.74
1:D:355:ILE:HG13	5:D:2104:HOH:O	1.87	0.74
1:C:59:LEU:HA	1:C:64:VAL:HG22	1.69	0.74
1:A:109:ILE:CD1	1:A:149:ALA:HB2	2.17	0.74
1:B:15:GLU:OE2	1:B:19:ILE:HG12	1.87	0.74
4:G:3:DC:H2"	4:G:4:DG:OP2	1.85	0.74
1:C:171:ASN:HB3	3:L:2:C:H4'	1.69	0.74
1:B:43:SER:HG	1:B:269:GLN:HG3	1.49	0.74
1:C:557:ARG:HD2	5:C:2129:HOH:O	1.87	0.74
1:B:234:ALA:HB1	5:B:2112:HOH:O	1.85	0.74
1:C:423:ARG:HE	1:C:781:ASN:HD22	1.35	0.74
4:P:1:DG:N2	5:P:2001:HOH:O	2.20	0.74
1:A:206:LYS:HD3	1:A:206:LYS:H	1.53	0.74
1:A:292:ARG:HH11	1:A:292:ARG:HG2	1.53	0.74
1:A:269:GLN:NE2	1:A:407:LYS:HZ3	1.86	0.74
1:B:119:ILE:HG13	5:B:2069:HOH:O	1.88	0.74
1:A:665:LEU:HG	5:A:2262:HOH:O	1.86	0.74
1:B:551:ARG:HG2	5:B:2240:HOH:O	1.87	0.74
1:D:11:PHE:CE1	1:D:44:TYR:HB3	2.23	0.74
1:A:663:LYS:CG	1:A:664:GLY:H	2.01	0.73
1:B:15:GLU:HA	5:B:2034:HOH:O	1.88	0.73
1:C:218:GLU:HG2	5:C:2070:HOH:O	1.88	0.73
1:D:810:ILE:HB	1:D:813:SER:HB3	1.69	0.73
1:A:206:LYS:O	1:A:210:ILE:HG12	1.88	0.73
1:B:744:GLN:CA	1:B:756:ARG:HE	2.01	0.73
1:C:303:LYS:CE	1:C:740:LYS:HZ1	2.01	0.73
1:C:711:LYS:CG	1:C:718:ILE:HA	2.17	0.73
1:C:88:TRP:O	1:C:92:VAL:HG23	1.89	0.73
1:A:269:GLN:NE2	1:A:407:LYS:NZ	2.33	0.73
1:B:407:LYS:NZ	5:B:2175:HOH:O	2.18	0.73
1:D:164:LYS:HA	1:D:164:LYS:HE2	1.69	0.73
1:D:272:VAL:HB	5:D:2021:HOH:O	1.87	0.73
1:A:84:ARG:HE	1:A:222:GLU:CB	2.02	0.73
1:B:215:ARG:C	1:B:219:MET:HE2	2.09	0.73
1:B:726:HIS:HB2	1:B:736:TRP:CD1	2.23	0.73
1:B:796:VAL:O	1:B:800:GLU:HG3	1.87	0.73
1:C:134:VAL:HG12	1:C:242:GLU:O	1.89	0.73
1:A:126:LEU:HD22	1:A:246:LEU:HB2	1.71	0.73
1:B:158:GLU:HA	1:B:195:LEU:HD22	1.68	0.73
1:C:199:GLU:HG2	1:C:201:TRP:HD1	1.54	0.73

Continued on next page...

Continued from previous page...

Atom-1	Atom-2	Interatomic distance (Å)	Clash overlap (Å)
2:K:18:DC:OP2	5:K:2015:HOH:O	2.07	0.73
1:A:698:TRP:HE3	1:A:699:LEU:HD23	1.53	0.73
1:B:422:TRP:HB3	5:B:2182:HOH:O	1.88	0.73
1:B:748:ASN:ND2	1:B:752:LEU:H	1.86	0.73
1:B:423:ARG:NH2	1:B:784:HIS:ND1	2.35	0.73
1:D:573:ILE:HA	1:D:576:LYS:CD	2.19	0.73
1:C:109:ILE:HD11	1:C:149:ALA:HB2	1.68	0.73
1:C:374:LEU:H	1:C:374:LEU:CD1	2.02	0.73
1:B:480:LYS:O	1:B:484:GLU:HG3	1.89	0.73
1:B:681:ILE:O	1:B:685:VAL:HG13	1.89	0.73
1:D:176:HIS:HA	5:D:2052:HOH:O	1.89	0.73
1:A:51:PHE:HZ	1:A:261:LEU:HD23	1.53	0.73
1:C:882:PHE:HD1	1:C:882:PHE:H	1.34	0.73
1:B:744:GLN:HA	1:B:756:ARG:NE	2.03	0.72
1:C:597:VAL:HB	5:C:2141:HOH:O	1.87	0.72
1:C:668:THR:HG22	1:C:669:GLN:OE1	1.88	0.72
1:D:882:PHE:HD1	1:D:882:PHE:H	1.36	0.72
1:B:829:ARG:HH11	1:B:829:ARG:CG	2.01	0.72
1:C:727:TRP:HA	1:C:848:GLN:HE21	1.53	0.72
1:B:250:TYR:HD2	5:B:2121:HOH:O	1.71	0.72
1:B:347:CYS:CB	1:B:350:GLU:HG3	2.18	0.72
1:B:596:THR:O	5:B:2264:HOH:O	2.07	0.72
1:B:73:LEU:HD11	1:B:254:ILE:HG13	1.70	0.72
1:C:711:LYS:HG2	1:C:718:ILE:HA	1.69	0.72
4:J:4:DG:H2"	4:J:5:DA:C8	2.23	0.72
4:J:5:DA:H3'	5:J:2003:HOH:O	1.89	0.72
1:A:349:VAL:O	1:A:349:VAL:HG12	1.87	0.72
1:B:354:ALA:HB2	5:B:2221:HOH:O	1.88	0.72
1:C:120:LYS:HZ3	1:C:752:LEU:HD11	1.54	0.72
1:C:437:ASN:ND2	1:C:440:THR:H	1.86	0.72
1:D:109:ILE:HG21	5:D:2041:HOH:O	1.89	0.72
1:D:378:LYS:HD2	5:D:2007:HOH:O	1.88	0.72
1:D:45:GLU:HG2	5:D:2024:HOH:O	1.88	0.72
1:A:134:VAL:CB	1:A:244:ILE:HD11	2.19	0.72
1:B:546:PHE:HD1	1:B:549:MET:CE	2.02	0.72
1:B:730:PRO:HD3	5:B:2308:HOH:O	1.89	0.72
1:C:574:VAL:O	1:C:578:VAL:HG23	1.90	0.72
1:D:300:HIS:HB2	5:D:2078:HOH:O	1.90	0.72
1:B:14:ILE:HG23	1:B:288:ALA:HB1	1.72	0.72
1:D:778:ILE:HG23	1:D:779:ALA:N	2.04	0.72
1:D:80:LYS:HD3	1:D:224:THR:HG22	1.72	0.72

Continued on next page...

Continued from previous page...

Atom-1	Atom-2	Interatomic distance (Å)	Clash overlap (Å)
4:G:5:DA:H2”	4:G:6:DT:C7	2.17	0.72
4:P:4:DG:H2”	4:P:5:DA:C8	2.25	0.72
1:A:58:GLN:OE1	1:A:67:ASN:HB2	1.89	0.72
1:B:882:PHE:O	1:B:883:ALA:CB	2.37	0.72
1:D:347:CYS:HB3	1:D:350:GLU:HG2	1.70	0.72
1:D:582:LEU:HD11	1:D:625:VAL:HG21	1.70	0.72
1:B:165:ASN:HA	5:B:2093:HOH:O	1.89	0.72
1:C:486:HIS:CE1	1:C:490:MET:HG3	2.25	0.72
1:C:630:THR:O	1:C:634:VAL:HG12	1.89	0.72
1:C:96:ARG:HG2	1:C:96:ARG:HH11	1.55	0.72
1:A:745:THR:H	1:A:756:ARG:HD3	1.55	0.71
1:B:720:ARG:NH1	1:B:720:ARG:HG2	2.01	0.71
1:C:333:LYS:HB3	1:C:516:PHE:CD2	2.24	0.71
1:A:84:ARG:HB3	5:A:2060:HOH:O	1.88	0.71
1:A:324:GLN:HE21	1:A:417:PRO:HA	1.55	0.71
1:A:882:PHE:O	1:A:883:ALA:HB3	1.90	0.71
1:A:568:GLN:OE1	1:B:565:GLU:HB2	1.89	0.71
1:C:485:ASN:HD22	1:C:488:ASN:ND2	1.86	0.71
1:C:737:GLN:HE22	1:C:778:ILE:HA	1.54	0.71
1:A:247:ALA:HB1	5:A:2098:HOH:O	1.89	0.71
1:A:72:PRO:HB3	5:A:2110:HOH:O	1.90	0.71
1:A:872:LEU:O	1:A:875:ILE:HG13	1.89	0.71
1:B:50:ARG:NH1	1:B:50:ARG:CG	2.51	0.71
1:B:826:LYS:O	1:B:830:GLU:HG3	1.89	0.71
1:D:347:CYS:CB	1:D:350:GLU:HG2	2.20	0.71
1:A:425:ARG:HD3	1:A:811:HIS:HD2	1.56	0.71
1:D:854:HIS:CD2	1:D:856:SER:H	2.08	0.71
1:A:57:ARG:O	1:A:60:LYS:HE2	1.90	0.71
1:D:810:ILE:HG22	3:O:8:U:H5’	1.73	0.71
1:A:77:LEU:HD21	1:A:226:MET:SD	2.30	0.71
1:B:118:THR:HG23	1:B:141:ILE:HG21	1.71	0.71
1:B:546:PHE:CD1	1:B:549:MET:CE	2.73	0.71
1:C:154:ILE:CG2	1:C:190:MET:HE1	2.21	0.71
1:C:59:LEU:HD23	1:C:64:VAL:HG22	1.73	0.71
1:A:342:THR:HG22	1:A:348:PRO:CG	2.18	0.71
1:C:720:ARG:NH1	1:C:720:ARG:HG2	2.05	0.71
1:D:315:GLU:OE2	1:D:318:LYS:HD3	1.90	0.71
1:A:220:LEU:HG	5:A:2101:HOH:O	1.91	0.70
1:B:72:PRO:HG3	1:B:257:ARG:HG3	1.72	0.70
1:B:452:ILE:HG23	1:B:453:GLY:N	2.06	0.70
1:C:713:LYS:HA	1:C:713:LYS:HZ3	1.56	0.70

Continued on next page...

Continued from previous page...

Atom-1	Atom-2	Interatomic distance (Å)	Clash overlap (Å)
1:A:479:ILE:O	1:A:483:GLU:HG3	1.90	0.70
1:C:529:ASN:HB2	5:C:2124:HOH:O	1.92	0.70
1:D:14:ILE:HG23	1:D:288:ALA:HB1	1.72	0.70
1:D:421:ASP:OD2	1:D:423:ARG:NH1	2.25	0.70
2:E:12:DT:H2"	2:E:13:DC:H5'	1.74	0.70
1:A:746:ARG:CB	1:A:746:ARG:HH11	2.04	0.70
1:B:236:VAL:CB	1:B:239:GLN:HB2	2.20	0.70
1:C:109:ILE:CG1	1:C:149:ALA:HB2	2.21	0.70
1:C:437:ASN:H	1:C:437:ASN:ND2	1.88	0.70
1:C:869:ASN:N	1:C:869:ASN:HD22	1.88	0.70
1:D:182:PHE:O	1:D:185:VAL:HG22	1.91	0.70
1:D:553:GLU:HA	1:D:870:LEU:HD12	1.72	0.70
1:A:17:ALA:HA	5:A:2048:HOH:O	1.91	0.70
1:A:51:PHE:CE2	1:A:261:LEU:HB3	2.26	0.70
1:D:289:ASN:HB3	5:D:2074:HOH:O	1.90	0.70
1:D:402:LEU:HG	1:D:439:MET:CE	2.22	0.70
2:K:12:DT:H2"	2:K:13:DC:H5'	1.73	0.70
1:A:191:LEU:HA	5:A:2085:HOH:O	1.92	0.70
1:C:208:ASP:N	1:C:208:ASP:OD2	2.24	0.70
1:C:355:ILE:HA	5:C:2094:HOH:O	1.91	0.70
1:D:441:LYS:HD3	5:D:2117:HOH:O	1.90	0.70
1:A:105:PHE:HB3	1:A:204:TRP:CZ2	2.26	0.70
1:A:308:TYR:HE2	1:A:734:PRO:HG2	1.56	0.70
1:B:355:ILE:HA	5:B:2165:HOH:O	1.91	0.70
1:A:347:CYS:HB3	1:A:350:GLU:CG	2.20	0.70
1:A:871:ASN:HD21	1:A:873:ARG:HB2	1.56	0.70
1:A:51:PHE:HE2	1:A:261:LEU:HB3	1.56	0.70
1:C:201:TRP:O	1:C:204:TRP:HB2	1.92	0.70
1:A:308:TYR:HA	1:A:311:VAL:HG23	1.74	0.69
1:A:698:TRP:CE3	1:A:699:LEU:HD23	2.26	0.69
1:B:236:VAL:CG1	1:B:239:GLN:HB2	2.22	0.69
1:C:307:ARG:NH1	1:C:307:ARG:HG3	2.03	0.69
1:A:236:VAL:HB	1:A:239:GLN:HB2	1.74	0.69
1:A:32:LEU:HD12	1:A:272:VAL:CG1	2.22	0.69
1:A:120:LYS:CE	1:A:752:LEU:HD21	2.15	0.69
1:B:423:ARG:HE	2:H:12:DT:C4'	2.03	0.69
1:C:402:LEU:HG	1:C:439:MET:CE	2.23	0.69
1:D:113:ALA:O	1:D:117:ILE:HG13	1.92	0.69
1:A:619:GLN:O	1:A:622:ALA:HB3	1.92	0.69
1:D:710:VAL:HG13	1:D:720:ARG:HB3	1.74	0.69
3:I:6:G:H3'	5:I:2011:HOH:O	1.91	0.69

Continued on next page...

Continued from previous page...

Atom-1	Atom-2	Interatomic distance (Å)	Clash overlap (Å)
1:C:108:GLU:OE1	1:C:108:GLU:HA	1.91	0.69
1:C:6:ILE:HD11	1:C:259:GLY:C	2.13	0.69
1:A:663:LYS:HG2	1:A:664:GLY:H	1.57	0.69
1:B:21:PHE:HD1	1:B:21:PHE:O	1.76	0.69
1:D:248:PRO:O	1:D:252:GLU:HG3	1.93	0.69
1:D:40:GLU:OE1	1:D:286:TYR:HB3	1.91	0.69
1:A:402:LEU:O	1:A:406:ASN:ND2	2.24	0.69
1:A:860:LYS:O	1:A:860:LYS:HD3	1.93	0.69
1:B:85:ILE:HG12	1:B:219:MET:SD	2.33	0.69
1:A:427:TYR:HA	1:A:435:GLN:HE22	1.57	0.69
1:C:706:LEU:HD21	1:C:849:PHE:HB2	1.74	0.69
1:C:860:LYS:HD2	1:C:860:LYS:O	1.93	0.69
4:G:1:DG:H2”	4:G:2:DT:OP2	1.93	0.69
1:A:159:ALA:HB1	1:A:163:LYS:N	2.08	0.69
1:A:571:TYR:HD1	1:A:634:VAL:HG11	1.57	0.69
1:A:308:TYR:CE2	1:A:734:PRO:HG2	2.28	0.69
1:B:58:GLN:HG3	1:B:67:ASN:ND2	2.05	0.69
1:C:308:TYR:CE2	1:C:734:PRO:HG2	2.28	0.69
1:D:170:LEU:O	1:D:179:LYS:HE2	1.93	0.69
1:A:121:THR:HG22	5:A:2069:HOH:O	1.93	0.69
1:A:88:TRP:O	1:A:92:VAL:HG23	1.93	0.69
1:A:172:LYS:HA	5:A:2080:HOH:O	1.92	0.69
1:B:553:GLU:OE1	1:B:869:ASN:N	2.18	0.69
1:C:713:LYS:NZ	1:C:713:LYS:HA	2.07	0.69
1:D:109:ILE:HG13	1:D:149:ALA:HB2	1.74	0.69
1:B:386:ARG:NH2	3:I:5:C:OP2	2.26	0.69
4:M:7:DT:H2”	4:M:8:DC:C6	2.28	0.69
2:N:12:DT:H2”	2:N:13:DC:H5’	1.75	0.69
1:A:375:THR:HG22	1:A:375:THR:O	1.93	0.69
1:A:517:GLU:OE1	1:A:517:GLU:HA	1.93	0.69
1:B:120:LYS:HD2	1:B:752:LEU:CD2	2.23	0.69
1:D:64:VAL:HA	5:D:2026:HOH:O	1.92	0.69
1:A:118:THR:CG2	1:A:141:ILE:HD13	2.23	0.68
1:C:437:ASN:HD22	1:C:437:ASN:N	1.91	0.68
1:C:505:GLN:O	1:C:508:PRO:HD3	1.92	0.68
1:C:656:GLN:HB3	1:C:657:PRO:CD	2.22	0.68
1:B:870:LEU:HD23	1:B:871:ASN:N	2.07	0.68
1:C:752:LEU:HB3	5:C:2160:HOH:O	1.91	0.68
1:D:281:ILE:HD11	1:D:308:TYR:HB3	1.75	0.68
1:D:704:LYS:HE3	1:D:860:LYS:NZ	2.08	0.68
1:B:126:LEU:HD23	5:B:2083:HOH:O	1.92	0.68

Continued on next page...

Continued from previous page...

Atom-1	Atom-2	Interatomic distance (Å)	Clash overlap (Å)
1:C:92:VAL:HG12	1:C:99:ARG:HG3	1.76	0.68
1:A:308:TYR:HA	1:A:311:VAL:CG2	2.23	0.68
1:C:373:ALA:HB1	1:C:377:TRP:HE1	1.59	0.68
1:C:669:GLN:HG2	1:C:672:GLN:HE21	1.58	0.68
3:I:4:G:N7	5:I:2006:HOH:O	2.24	0.68
2:N:5:DA:H2"	2:N:6:DT:OP2	1.92	0.68
1:A:425:ARG:NH2	1:A:784:HIS:HD2	1.91	0.68
1:A:592:ASN:OD1	1:A:611:LEU:HD23	1.92	0.68
1:B:335:LEU:HD22	1:B:339:ASN:ND2	2.09	0.68
1:B:680:LEU:N	1:B:680:LEU:HD12	2.09	0.68
1:D:19:ILE:HG23	1:D:20:PRO:HD2	1.75	0.68
1:D:236:VAL:HG11	1:D:239:GLN:HB2	1.75	0.68
1:A:448:LYS:HE2	5:A:2287:HOH:O	1.94	0.68
1:A:546:PHE:CZ	1:A:783:VAL:CG2	2.75	0.68
1:A:730:PRO:CD	1:A:786:GLN:HE22	2.06	0.68
1:B:215:ARG:O	1:B:219:MET:HG3	1.93	0.68
1:C:616:LEU:HD23	1:C:619:GLN:OE1	1.93	0.68
1:B:553:GLU:CD	1:B:553:GLU:H	1.96	0.68
1:D:608:LYS:HG3	5:D:2142:HOH:O	1.93	0.68
1:D:804:ILE:HG23	1:D:816:THR:HG21	1.76	0.68
1:A:116:TYR:CE2	1:A:752:LEU:HD22	2.29	0.68
1:B:143:ARG:NH1	1:B:209:SER:OG	2.27	0.68
1:B:325:ASN:HB3	5:B:2147:HOH:O	1.93	0.68
1:C:159:ALA:HB1	1:C:163:LYS:N	2.08	0.68
1:B:51:PHE:HD2	1:B:51:PHE:O	1.77	0.68
1:B:92:VAL:HG22	5:B:2075:HOH:O	1.94	0.68
1:C:801:LYS:HE3	1:C:801:LYS:O	1.93	0.68
1:A:226:MET:HB3	5:A:2101:HOH:O	1.93	0.68
1:A:423:ARG:HE	1:A:781:ASN:ND2	1.91	0.68
1:B:790:HIS:NE2	1:B:831:THR:HG22	2.09	0.68
1:D:84:ARG:HD2	1:D:219:MET:HG2	1.74	0.68
1:C:849:PHE:CD2	1:C:853:LEU:HD21	2.27	0.67
1:D:596:THR:HG23	5:D:2143:HOH:O	1.92	0.67
1:D:84:ARG:HG2	1:D:84:ARG:HH11	1.57	0.67
1:A:112:GLU:H	1:A:112:GLU:CD	1.98	0.67
1:B:165:ASN:OD1	1:B:165:ASN:N	2.27	0.67
1:C:232:GLN:HB2	1:C:241:SER:O	1.94	0.67
1:D:181:ALA:O	1:D:185:VAL:HG13	1.94	0.67
1:A:713:LYS:O	1:A:714:LYS:HE2	1.94	0.67
1:A:882:PHE:O	1:A:883:ALA:CB	2.42	0.67
1:B:650:VAL:HA	5:B:2281:HOH:O	1.93	0.67

Continued on next page...

Continued from previous page...

Atom-1	Atom-2	Interatomic distance (Å)	Clash overlap (Å)
1:B:656:GLN:HB3	1:B:657:PRO:CD	2.24	0.67
1:A:89:PHE:HE2	1:A:107:GLN:HG3	1.60	0.67
1:A:485:ASN:HD22	1:A:488:ASN:HD22	1.41	0.67
1:D:816:THR:OG1	1:D:824:LEU:HD23	1.94	0.67
2:H:12:DT:H2”	2:H:13:DC:H5’	1.76	0.67
4:P:8:DC:H2”	4:P:9:DC:OP2	1.94	0.67
1:B:505:GLN:HG3	1:B:511:PHE:CD2	2.30	0.67
1:C:109:ILE:CD1	1:C:149:ALA:HB2	2.24	0.67
1:C:126:LEU:HD23	1:C:132:THR:CG2	2.24	0.67
1:C:72:PRO:HG2	5:C:2043:HOH:O	1.93	0.67
1:A:22:ASN:ND2	5:A:2043:HOH:O	2.27	0.67
1:B:138:ALA:O	1:B:213:GLY:HA3	1.94	0.67
1:A:150:ARG:HD2	1:A:201:TRP:CG	2.30	0.67
1:A:225:GLY:HA3	5:A:2099:HOH:O	1.95	0.67
1:A:421:ASP:OD2	1:A:427:TYR:CE1	2.47	0.67
1:B:324:GLN:NE2	1:B:418:TYR:H	1.91	0.67
1:C:151:PHE:CD1	1:C:183:MET:HB3	2.30	0.67
1:C:324:GLN:HE21	1:C:418:TYR:H	1.40	0.67
1:D:351:ASP:N	5:D:2097:HOH:O	2.19	0.67
1:A:794:THR:OG1	1:A:831:THR:HG21	1.93	0.67
1:D:326:THR:HG23	1:D:806:SER:HA	1.74	0.67
1:D:488:ASN:HB3	1:D:501:TRP:CE3	2.30	0.67
1:D:630:THR:O	1:D:634:VAL:HG12	1.95	0.67
1:C:15:GLU:HG2	1:C:18:ALA:O	1.94	0.67
1:C:711:LYS:HG2	1:C:717:GLU:C	2.15	0.67
1:C:374:LEU:HD12	1:C:374:LEU:N	2.10	0.67
1:D:726:HIS:CD2	1:D:727:TRP:N	2.63	0.67
1:A:109:ILE:N	1:A:109:ILE:HD12	2.10	0.66
1:A:879:ASP:HB2	5:A:2307:HOH:O	1.95	0.66
1:B:215:ARG:O	1:B:219:MET:HE2	1.95	0.66
1:B:346:HIS:HA	1:B:395:ARG:HH11	1.60	0.66
1:B:55:PHE:HE1	1:B:69:ALA:CB	2.08	0.66
1:C:191:LEU:HB2	5:C:2066:HOH:O	1.94	0.66
1:C:324:GLN:NE2	1:C:418:TYR:H	1.93	0.66
1:A:105:PHE:HB3	1:A:204:TRP:CH2	2.30	0.66
1:A:55:PHE:CE2	1:A:59:LEU:HD11	2.30	0.66
1:B:737:GLN:NE2	1:B:739:TYR:HE2	1.93	0.66
1:D:298:ARG:HH21	1:D:427:TYR:CB	2.05	0.66
1:B:881:ALA:HA	5:B:2352:HOH:O	1.94	0.66
1:A:118:THR:HG21	1:A:216:CYS:HB3	1.77	0.66
1:B:422:TRP:HE3	5:B:2182:HOH:O	1.78	0.66

Continued on next page...

Continued from previous page...

Atom-1	Atom-2	Interatomic distance (Å)	Clash overlap (Å)
1:D:183:MET:HE3	5:D:2048:HOH:O	1.95	0.66
1:A:15:GLU:HB2	1:A:19:ILE:HG12	1.76	0.66
1:A:220:LEU:HA	5:A:2097:HOH:O	1.95	0.66
1:B:416:PHE:HD1	1:B:430:SER:HG	1.43	0.66
1:C:21:PHE:HE1	5:C:2031:HOH:O	1.77	0.66
4:P:4:DG:H1'	5:P:2005:HOH:O	1.95	0.66
1:A:623:TYR:CD1	1:A:663:LYS:HE3	2.31	0.66
1:D:766:ASP:HA	5:D:2161:HOH:O	1.95	0.66
1:B:432:PHE:CE2	1:B:444:LEU:HD21	2.31	0.66
1:C:292:ARG:N	1:C:293:PRO:HD3	2.11	0.66
1:D:669:GLN:HG2	1:D:672:GLN:NE2	2.10	0.66
3:L:4:G:N7	5:L:2004:HOH:O	2.29	0.66
1:A:205:HIS:O	1:A:207:GLU:N	2.29	0.66
1:A:882:PHE:H	1:A:882:PHE:HD1	1.37	0.66
1:B:215:ARG:HG3	1:B:219:MET:CE	2.26	0.66
1:C:303:LYS:HE2	1:C:740:LYS:NZ	2.10	0.66
1:C:417:PRO:HG2	1:C:429:VAL:HB	1.77	0.66
1:C:552:ASP:HB2	1:C:691:ALA:HB2	1.77	0.66
1:A:118:THR:HG22	1:A:141:ILE:HD13	1.78	0.66
1:B:324:GLN:HE21	1:B:418:TYR:H	1.44	0.66
1:B:231:ARG:HH11	1:B:242:GLU:HB2	1.61	0.66
1:B:24:LEU:HD13	1:B:33:ALA:HA	1.79	0.66
1:B:536:PHE:HB3	1:B:882:PHE:HB3	1.76	0.66
1:B:423:ARG:HH11	1:B:423:ARG:HB2	1.61	0.65
1:C:606:SER:HB2	5:C:2141:HOH:O	1.96	0.65
1:A:227:VAL:HB	1:A:244:ILE:HG22	1.77	0.65
1:A:398:LEU:CD2	1:A:398:LEU:C	2.64	0.65
1:A:452:ILE:HG22	1:A:528:TYR:O	1.95	0.65
1:B:110:LYS:HG2	1:B:112:GLU:OE1	1.96	0.65
1:B:573:ILE:HD11	1:B:688:THR:HG21	1.78	0.65
1:B:574:VAL:O	1:B:578:VAL:HG23	1.96	0.65
1:C:116:TYR:OH	1:C:752:LEU:HD22	1.96	0.65
1:C:312:TYR:CZ	1:C:314:PRO:HG2	2.30	0.65
1:C:656:GLN:HG2	5:C:2148:HOH:O	1.96	0.65
1:D:155:ARG:HB2	5:D:2044:HOH:O	1.95	0.65
1:A:470:VAL:HG12	1:A:470:VAL:O	1.97	0.65
1:A:711:LYS:NZ	1:A:711:LYS:CB	2.52	0.65
1:B:21:PHE:C	1:B:21:PHE:CD1	2.69	0.65
1:B:331:ASN:HB2	1:B:445:THR:HG22	1.79	0.65
1:B:730:PRO:HD3	1:B:786:GLN:HE22	1.61	0.65
1:B:96:ARG:HD3	5:B:2078:HOH:O	1.95	0.65

Continued on next page...

Continued from previous page...

Atom-1	Atom-2	Interatomic distance (Å)	Clash overlap (Å)
1:D:337:VAL:O	1:D:341:ILE:HG12	1.96	0.65
1:A:219:MET:HB3	5:A:2060:HOH:O	1.96	0.65
1:A:36:GLN:HG3	1:A:273:VAL:HG22	1.79	0.65
1:B:182:PHE:O	1:B:185:VAL:HG22	1.96	0.65
1:B:867:LYS:HE2	5:B:2240:HOH:O	1.95	0.65
1:C:103:PHE:CE2	1:C:107:GLN:NE2	2.64	0.65
1:C:15:GLU:CG	1:C:18:ALA:H	2.08	0.65
1:C:577:LYS:HE3	5:C:2135:HOH:O	1.96	0.65
1:C:551:ARG:HE	1:C:872:LEU:HD11	1.60	0.65
1:B:54:MET:O	1:B:58:GLN:HG2	1.96	0.65
1:C:307:ARG:CG	1:C:307:ARG:HH11	2.04	0.65
1:C:32:LEU:HG	1:C:272:VAL:HG12	1.77	0.65
1:D:692:ALA:O	1:D:696:MET:HG3	1.96	0.65
1:A:457:TYR:CD1	1:A:521:VAL:HG11	2.32	0.65
1:A:720:ARG:NH1	1:A:721:LYS:O	2.29	0.65
1:D:663:LYS:HG2	1:D:664:GLY:N	2.07	0.65
3:F:1:G:H2'	3:F:2:C:C6	2.32	0.65
1:B:111:PRO:HG2	1:B:112:GLU:OE2	1.97	0.65
1:B:391:ARG:HH11	1:B:391:ARG:HB3	1.61	0.65
1:C:333:LYS:HB3	1:C:516:PHE:HE2	1.56	0.65
1:D:155:ARG:CB	1:D:163:LYS:HE2	2.27	0.65
4:G:5:DA:C2'	4:G:6:DT:H71	2.19	0.65
2:K:7:DC:H2"	2:K:8:DG:O5'	1.94	0.65
1:B:278:TRP:HE1	1:B:324:GLN:HE22	1.45	0.65
1:B:690:VAL:O	1:B:694:GLU:HG3	1.97	0.65
1:D:319:ALA:CB	1:D:792:ARG:HG2	2.27	0.65
1:A:571:TYR:CD1	1:A:634:VAL:HG11	2.32	0.65
1:B:256:THR:HG22	5:B:2124:HOH:O	1.96	0.65
1:B:374:LEU:HD12	1:B:374:LEU:H	1.60	0.65
1:D:36:GLN:HG3	1:D:273:VAL:HG22	1.79	0.65
1:B:599:ASP:HA	5:B:2267:HOH:O	1.97	0.65
1:C:333:LYS:CB	1:C:516:PHE:CE2	2.76	0.65
1:D:281:ILE:HG12	1:D:309:GLU:HA	1.78	0.65
1:A:276:LYS:HD2	1:A:283:GLY:O	1.96	0.64
1:A:540:CYS:HB3	5:A:2024:HOH:O	1.97	0.64
1:A:99:ARG:NH1	1:A:99:ARG:HG2	1.98	0.64
1:C:704:LYS:HE3	5:M:2006:HOH:O	1.97	0.64
1:D:220:LEU:HG	5:D:2059:HOH:O	1.97	0.64
1:D:719:LEU:CD1	1:D:719:LEU:N	2.59	0.64
1:A:281:ILE:HD11	1:A:308:TYR:CB	2.27	0.64
1:B:717:GLU:HA	5:B:2305:HOH:O	1.96	0.64

Continued on next page...

Continued from previous page...

Atom-1	Atom-2	Interatomic distance (Å)	Clash overlap (Å)
1:B:791:LEU:HD21	1:B:809:LEU:HD13	1.79	0.64
1:D:159:ALA:HB1	1:D:163:LYS:N	2.11	0.64
1:D:59:LEU:HD23	1:D:64:VAL:HG22	1.79	0.64
1:D:786:GLN:HG3	1:D:836:TYR:OH	1.96	0.64
1:A:119:ILE:HD12	5:A:2059:HOH:O	1.96	0.64
1:A:170:LEU:HD12	1:A:183:MET:HE1	1.80	0.64
1:A:485:ASN:HD22	1:A:488:ASN:ND2	1.96	0.64
1:A:714:LYS:NZ	1:A:714:LYS:HA	2.13	0.64
1:C:751:PHE:C	1:C:752:LEU:HD12	2.18	0.64
1:D:824:LEU:HD12	1:D:824:LEU:O	1.97	0.64
1:A:292:ARG:HG3	1:A:292:ARG:O	1.97	0.64
1:D:663:LYS:HE3	1:D:666:MET:CE	2.28	0.64
1:A:40:GLU:OE1	1:A:286:TYR:HB3	1.98	0.64
1:A:595:VAL:HG22	5:A:2237:HOH:O	1.97	0.64
1:C:73:LEU:HG	5:C:2043:HOH:O	1.96	0.64
1:D:141:ILE:O	1:D:145:ILE:HG12	1.98	0.64
2:K:9:DA:N6	4:M:1:DG:N2	2.45	0.64
1:C:291:ARG:HB2	5:C:2079:HOH:O	1.96	0.64
1:C:341:ILE:HD12	1:C:348:PRO:HB3	1.78	0.64
1:A:425:ARG:NH2	1:A:784:HIS:CD2	2.66	0.64
1:A:668:THR:CG2	1:A:669:GLN:NE2	2.43	0.64
1:B:77:LEU:HD12	1:B:224:THR:HG21	1.79	0.64
3:L:1:G:H2'	3:L:2:C:C6	2.32	0.64
1:A:80:LYS:HE2	1:A:224:THR:HG22	1.79	0.64
1:A:32:LEU:HD12	1:A:272:VAL:HG12	1.79	0.64
1:B:148:GLU:OE2	1:B:148:GLU:HA	1.97	0.64
1:B:663:LYS:CG	1:B:664:GLY:H	1.94	0.64
1:C:115:ALA:O	1:C:119:ILE:HG12	1.97	0.64
1:C:68:ALA:HB3	1:C:261:LEU:HD21	1.79	0.64
1:C:455:GLU:OE1	1:C:455:GLU:HA	1.98	0.64
1:C:816:THR:OG1	1:C:824:LEU:HD22	1.98	0.64
1:D:790:HIS:NE2	1:D:832:MET:HB2	2.13	0.64
1:C:422:TRP:CH2	2:K:12:DT:H5”	2.33	0.64
1:A:391:ARG:HG2	1:A:391:ARG:HH11	1.63	0.64
1:B:132:THR:HB	5:B:2083:HOH:O	1.98	0.64
1:B:55:PHE:HE1	1:B:69:ALA:HB3	1.62	0.64
1:C:828:VAL:HB	1:C:883:ALA:HA	1.78	0.64
1:A:150:ARG:HD2	1:A:201:TRP:CD1	2.33	0.64
1:A:565:GLU:HG3	5:A:2225:HOH:O	1.97	0.64
5:A:2025:HOH:O	1:B:553:GLU:HG3	1.97	0.64
1:D:423:ARG:NH2	1:D:784:HIS:ND1	2.45	0.64

Continued on next page...

Continued from previous page...

Atom-1	Atom-2	Interatomic distance (Å)	Clash overlap (Å)
1:B:401:MET:HE1	1:B:432:PHE:HA	1.80	0.63
1:C:162:PHE:CG	5:C:2059:HOH:O	2.51	0.63
1:C:457:TYR:CD1	1:C:521:VAL:HG11	2.32	0.63
1:D:335:LEU:HD22	1:D:339:ASN:ND2	2.13	0.63
3:I:1:G:H2'	3:I:2:C:C6	2.34	0.63
2:K:18:DC:H2"	5:K:2016:HOH:O	1.99	0.63
1:B:71:LYS:N	1:B:72:PRO:HD2	2.14	0.63
1:C:804:ILE:HG23	1:C:816:THR:HG21	1.80	0.63
1:B:28:TYR:O	1:B:32:LEU:HD23	1.98	0.63
1:C:162:PHE:HB3	5:C:2059:HOH:O	1.99	0.63
1:B:639:TYR:O	2:H:10:DC:H4'	1.97	0.63
1:A:648:GLN:O	1:A:652:GLU:HG2	1.97	0.63
1:A:58:GLN:HG3	1:A:67:ASN:HD22	1.64	0.63
1:B:473:VAL:CG1	1:B:477:GLU:CB	2.76	0.63
1:B:475:PHE:HE1	1:B:478:ARG:NH1	1.97	0.63
1:C:15:GLU:HG2	1:C:18:ALA:H	1.62	0.63
1:C:522:GLN:OE1	1:C:522:GLN:HA	1.97	0.63
1:C:549:MET:HE2	1:C:841:VAL:HG21	1.81	0.63
1:D:133:THR:HA	1:D:243:THR:HG22	1.79	0.63
1:A:428:ALA:HB3	1:A:433:ASN:ND2	2.13	0.63
1:B:225:GLY:O	1:B:247:ALA:HB2	1.98	0.63
1:B:34:ARG:HD3	5:B:2049:HOH:O	1.97	0.63
1:C:183:MET:CE	1:C:183:MET:HA	2.29	0.63
1:C:532:LEU:HD23	1:C:534:LEU:CD2	2.24	0.63
1:C:652:GLU:HA	1:C:656:GLN:HB2	1.81	0.63
1:D:291:ARG:HB2	5:D:2076:HOH:O	1.97	0.63
1:D:537:ASP:H	1:D:882:PHE:HD2	1.46	0.63
1:D:84:ARG:NH1	1:D:84:ARG:HG2	2.13	0.63
1:A:181:ALA:O	1:A:185:VAL:HG13	1.99	0.63
1:A:881:ALA:O	1:A:882:PHE:C	2.34	0.63
1:C:113:ALA:O	1:C:117:ILE:HG13	1.97	0.63
1:C:303:LYS:NZ	1:C:740:LYS:NZ	2.46	0.63
1:C:726:HIS:HB3	5:C:2155:HOH:O	1.97	0.63
1:D:552:ASP:HB2	1:D:691:ALA:HB2	1.81	0.63
1:A:568:GLN:CD	1:B:565:GLU:HB2	2.19	0.63
1:B:138:ALA:HA	1:B:141:ILE:HD12	1.80	0.63
1:C:532:LEU:CD2	1:C:534:LEU:HD23	2.27	0.63
1:D:34:ARG:HG2	5:D:2023:HOH:O	1.98	0.63
1:D:796:VAL:HA	5:D:2167:HOH:O	1.98	0.63
1:A:710:VAL:HG13	1:A:720:ARG:HB3	1.81	0.63
1:B:375:THR:HG22	1:B:375:THR:O	1.99	0.63

Continued on next page...

Continued from previous page...

Atom-1	Atom-2	Interatomic distance (Å)	Clash overlap (Å)
1:C:123:LEU:O	1:C:127:THR:HG23	1.99	0.63
1:C:183:MET:HE2	1:C:183:MET:HA	1.81	0.63
1:A:437:ASN:ND2	1:A:440:THR:H	1.97	0.63
1:A:499:ASN:HA	5:A:2188:HOH:O	1.97	0.63
1:A:529:ASN:HD22	1:A:529:ASN:C	2.02	0.63
1:B:714:LYS:HA	1:B:714:LYS:HZ3	1.63	0.63
1:C:146:GLU:OE2	1:C:201:TRP:HB3	1.99	0.63
1:C:755:PHE:N	1:C:755:PHE:HD1	1.95	0.63
1:D:161:HIS:O	1:D:164:LYS:HG2	1.98	0.63
1:A:205:HIS:NE2	1:A:206:LYS:HE2	2.13	0.62
1:B:573:ILE:O	1:B:573:ILE:HD12	1.98	0.62
1:B:322:ILE:HD13	1:B:799:HIS:CG	2.34	0.62
1:C:587:ILE:HG22	1:C:588:ASN:ND2	2.13	0.62
1:C:779:ALA:O	1:C:783:VAL:HG22	1.99	0.62
1:D:824:LEU:HD12	1:D:828:VAL:HG13	1.81	0.62
1:A:138:ALA:O	1:A:213:GLY:HA3	1.99	0.62
1:A:159:ALA:O	1:A:163:LYS:HB2	1.99	0.62
1:B:335:LEU:HB2	5:B:2150:HOH:O	1.97	0.62
1:C:711:LYS:HG2	1:C:717:GLU:O	1.99	0.62
1:D:36:GLN:CG	1:D:273:VAL:HG22	2.29	0.62
1:D:748:ASN:HB2	1:D:753:GLY:CA	2.29	0.62
1:D:744:GLN:HA	1:D:756:ARG:NH1	2.14	0.62
1:A:77:LEU:CD2	1:A:226:MET:SD	2.87	0.62
1:B:350:GLU:OE2	1:B:394:ARG:NH2	2.27	0.62
1:B:790:HIS:NE2	1:B:831:THR:CG2	2.62	0.62
1:B:812:ASP:OD2	3:I:8:U:O2'	2.09	0.62
1:C:128:SER:HA	5:C:2055:HOH:O	1.98	0.62
1:C:133:THR:CA	1:C:243:THR:HG22	2.16	0.62
1:C:42:GLU:OE1	1:C:407:LYS:NZ	2.27	0.62
1:C:491:ALA:HB1	1:C:499:ASN:HD22	1.62	0.62
1:D:138:ALA:O	1:D:213:GLY:HA3	1.98	0.62
1:D:698:TRP:CZ3	1:D:842:LEU:HG	2.34	0.62
1:B:162:PHE:HE1	1:B:190:MET:HG2	1.64	0.62
1:B:236:VAL:HG11	1:B:239:GLN:HB2	1.81	0.62
1:B:281:ILE:HG23	1:B:305:LEU:HD21	1.81	0.62
1:C:303:LYS:HE2	1:C:740:LYS:HZ1	1.63	0.62
1:A:33:ALA:O	1:A:37:LEU:HG	1.99	0.62
1:B:881:ALA:CA	5:B:2352:HOH:O	2.47	0.62
1:C:169:GLN:O	1:C:173:ARG:HG2	2.00	0.62
1:D:164:LYS:HE2	1:D:164:LYS:CA	2.30	0.62
1:D:553:GLU:HA	1:D:870:LEU:CD1	2.28	0.62

Continued on next page...

Continued from previous page...

Atom-1	Atom-2	Interatomic distance (Å)	Clash overlap (Å)
1:D:882:PHE:O	1:D:883:ALA:HB3	1.98	0.62
1:A:881:ALA:C	5:A:2310:HOH:O	2.38	0.62
1:C:50:ARG:HH11	1:C:50:ARG:CG	2.12	0.62
1:D:109:ILE:CG2	5:D:2041:HOH:O	2.47	0.62
1:D:632:ARG:NH1	1:D:632:ARG:H	1.97	0.62
3:O:1:G:H2'	3:O:2:C:C6	2.34	0.62
1:B:183:MET:HE1	1:B:186:VAL:HG21	1.82	0.62
1:C:428:ALA:H	1:C:435:GLN:HE21	1.41	0.62
1:D:677:MET:O	1:D:681:ILE:HG13	2.00	0.62
1:D:700:LYS:HB2	1:D:700:LYS:NZ	2.14	0.62
1:D:713:LYS:HZ3	1:D:713:LYS:HA	1.63	0.62
1:D:746:ARG:NH1	1:D:746:ARG:HB3	2.15	0.62
1:B:268:PHE:HB3	1:B:286:TYR:OH	1.99	0.62
1:B:748:ASN:ND2	1:B:751:PHE:N	2.43	0.62
1:C:4:ILE:HG21	5:C:2019:HOH:O	1.99	0.62
1:D:236:VAL:CG1	1:D:239:GLN:HB2	2.29	0.62
1:A:113:ALA:O	1:A:117:ILE:CG1	2.44	0.62
1:C:50:ARG:HG2	1:C:50:ARG:NH1	2.09	0.62
1:D:335:LEU:HD22	1:D:335:LEU:O	1.99	0.62
1:D:88:TRP:HA	1:D:91:GLU:CD	2.21	0.62
1:B:195:LEU:HB3	5:B:2100:HOH:O	2.00	0.62
1:C:636:THR:O	1:C:641:SER:OG	2.18	0.62
1:D:814:PHE:HE1	1:D:883:ALA:CB	2.12	0.62
2:H:5:DA:H2"	2:H:6:DT:OP2	1.98	0.62
5:C:2147:HOH:O	4:M:2:DT:H5'	2.00	0.62
1:A:21:PHE:HD1	1:A:21:PHE:O	1.83	0.61
1:B:744:GLN:HA	1:B:756:ARG:HH21	1.65	0.61
1:C:560:ASN:OD1	1:C:568:GLN:HB2	1.99	0.61
1:C:755:PHE:N	1:C:755:PHE:CD1	2.67	0.61
1:C:846:TYR:CD1	1:C:850:ALA:HB2	2.35	0.61
1:D:56:GLU:CD	1:D:57:ARG:N	2.54	0.61
2:N:9:DA:OP2	5:N:2009:HOH:O	2.16	0.61
1:A:116:TYR:OH	1:A:752:LEU:HD22	2.00	0.61
1:A:332:LYS:HE2	1:A:410:ASN:ND2	2.15	0.61
1:C:418:TYR:HD2	1:C:426:VAL:CG1	2.13	0.61
1:C:570:ILE:HA	1:C:573:ILE:HG22	1.82	0.61
1:D:350:GLU:OE1	1:D:350:GLU:HA	2.00	0.61
1:D:826:LYS:O	1:D:830:GLU:HG3	1.99	0.61
1:B:109:ILE:H	1:B:109:ILE:HD12	1.65	0.61
1:B:158:GLU:HG2	1:B:195:LEU:CD2	2.26	0.61
1:B:80:LYS:CD	1:B:224:THR:HG22	2.28	0.61

Continued on next page...

Continued from previous page...

Atom-1	Atom-2	Interatomic distance (Å)	Clash overlap (Å)
1:B:6:ILE:HB	1:B:48:GLU:OE2	2.01	0.61
1:C:514:PHE:CD1	1:C:515:CYS:N	2.68	0.61
1:D:347:CYS:HB3	1:D:350:GLU:CG	2.31	0.61
1:D:746:ARG:HH12	1:D:754:GLN:H	1.48	0.61
1:D:730:PRO:HD2	1:D:786:GLN:HE22	1.65	0.61
1:D:881:ALA:O	1:D:882:PHE:C	2.38	0.61
1:A:11:PHE:HZ	1:A:263:GLY:HA2	1.66	0.61
1:C:303:LYS:CE	1:C:740:LYS:NZ	2.63	0.61
1:D:391:ARG:HB3	1:D:391:ARG:HH11	1.66	0.61
1:D:828:VAL:O	1:D:831:THR:HG22	2.00	0.61
1:D:869:ASN:N	1:D:869:ASN:ND2	2.47	0.61
1:A:249:GLU:HA	1:A:252:GLU:OE1	2.01	0.61
1:A:742:PRO:HB3	1:A:744:GLN:OE1	2.00	0.61
1:B:14:ILE:HG13	5:B:2134:HOH:O	2.00	0.61
1:B:51:PHE:CD2	1:B:51:PHE:O	2.53	0.61
1:B:881:ALA:O	1:B:882:PHE:C	2.37	0.61
1:C:226:MET:HA	1:C:250:TYR:CD1	2.36	0.61
2:H:3:DG:H2”	2:H:4:DA:OP2	2.00	0.61
1:A:71:LYS:N	1:A:72:PRO:HD2	2.15	0.61
1:B:155:ARG:NH2	1:B:749:LEU:HD23	2.15	0.61
1:B:472:LYS:HB2	5:B:2206:HOH:O	2.00	0.61
1:B:571:TYR:CD1	1:B:631:LYS:HA	2.35	0.61
1:C:410:ASN:HA	5:C:2098:HOH:O	1.98	0.61
1:C:869:ASN:N	1:C:869:ASN:ND2	2.46	0.61
1:C:882:PHE:O	1:C:883:ALA:HB3	1.99	0.61
1:D:66:ASP:OD2	1:D:752:LEU:HD23	1.99	0.61
1:A:229:LEU:HD13	1:A:244:ILE:CD1	2.31	0.61
1:B:743:ILE:O	1:B:743:ILE:HG22	1.99	0.61
1:C:768:GLU:OE2	5:C:2165:HOH:O	2.16	0.61
1:D:120:LYS:HG3	1:D:752:LEU:HD21	1.82	0.61
1:A:30:GLU:HG2	1:A:34:ARG:NH1	2.15	0.61
1:C:109:ILE:HG13	1:C:149:ALA:HB2	1.83	0.61
1:D:326:THR:HA	5:D:2087:HOH:O	2.01	0.61
1:D:43:SER:HA	1:D:46:MET:CE	2.28	0.61
1:A:246:LEU:HB3	5:A:2108:HOH:O	1.99	0.61
1:B:552:ASP:HB2	1:B:691:ALA:HB2	1.82	0.61
1:C:105:PHE:CE1	1:C:208:ASP:HB3	2.36	0.61
1:D:332:LYS:HG2	5:D:2011:HOH:O	2.00	0.61
1:D:534:LEU:HD11	1:D:818:PRO:HG3	1.81	0.61
1:A:380:ALA:O	1:A:384:VAL:HG23	2.01	0.61
1:A:553:GLU:HB2	5:A:2222:HOH:O	2.01	0.61

Continued on next page...

Continued from previous page...

Atom-1	Atom-2	Interatomic distance (Å)	Clash overlap (Å)
1:B:401:MET:CE	1:B:432:PHE:HD1	2.14	0.61
1:B:537:ASP:H	1:B:882:PHE:HD2	1.47	0.61
1:C:19:ILE:CD1	1:C:20:PRO:HD2	2.31	0.61
1:C:38:ALA:HA	5:C:2034:HOH:O	2.01	0.61
1:D:132:THR:HA	5:D:2037:HOH:O	2.00	0.61
5:A:2258:HOH:O	2:E:10:DC:H5	1.82	0.61
4:J:3:DC:H2'	5:J:2002:HOH:O	2.01	0.61
1:A:871:ASN:ND2	1:A:873:ARG:HB2	2.15	0.60
1:B:632:ARG:CD	1:B:632:ARG:H	2.14	0.60
1:C:536:PHE:HB3	1:C:882:PHE:CB	2.24	0.60
1:C:809:LEU:C	1:C:810:ILE:HG13	2.22	0.60
1:D:16:LEU:HB2	5:D:2022:HOH:O	2.00	0.60
2:E:4:DA:H5"	5:E:2003:HOH:O	2.00	0.60
2:N:12:DT:H2"	2:N:13:DC:C5'	2.31	0.60
1:A:182:PHE:O	1:A:185:VAL:HG22	2.00	0.60
1:B:16:LEU:HD13	1:B:38:ALA:HA	1.83	0.60
1:B:355:ILE:HB	5:B:2167:HOH:O	2.01	0.60
1:D:226:MET:HB3	5:D:2059:HOH:O	2.01	0.60
1:D:56:GLU:C	1:D:56:GLU:CD	2.59	0.60
1:D:9:ASN:HA	1:D:12:SER:HB3	1.83	0.60
1:A:546:PHE:CE2	1:A:783:VAL:HG21	2.36	0.60
1:A:84:ARG:HH21	1:A:222:GLU:HB3	1.67	0.60
1:B:216:CYS:HA	1:B:219:MET:CE	2.31	0.60
1:D:34:ARG:HD3	5:D:2016:HOH:O	2.00	0.60
1:A:825:PHE:CZ	1:A:829:ARG:NH2	2.68	0.60
1:B:437:ASN:HD22	1:B:437:ASN:C	2.05	0.60
1:C:173:ARG:NH2	5:C:2062:HOH:O	2.34	0.60
1:C:227:VAL:HG12	1:C:245:GLU:O	2.02	0.60
1:C:554:VAL:HG23	5:C:2128:HOH:O	2.00	0.60
1:D:16:LEU:HA	1:D:37:LEU:CD1	2.31	0.60
2:K:12:DT:H2"	2:K:13:DC:C5'	2.31	0.60
1:A:117:ILE:O	1:A:121:THR:OG1	2.16	0.60
1:A:388:ASP:OD1	1:A:392:LYS:HE2	2.01	0.60
1:A:632:ARG:HB2	1:A:632:ARG:HH11	1.63	0.60
1:B:221:ILE:HG12	1:B:227:VAL:O	2.01	0.60
1:B:881:ALA:C	5:B:2352:HOH:O	2.37	0.60
1:C:229:LEU:HD13	1:C:244:ILE:CD1	2.31	0.60
1:C:4:ILE:CG2	5:C:2019:HOH:O	2.48	0.60
1:D:120:LYS:HD3	1:D:120:LYS:C	2.22	0.60
1:D:64:VAL:HG21	1:D:127:THR:HG21	1.82	0.60
1:D:99:ARG:HD3	1:D:103:PHE:HE2	1.67	0.60

Continued on next page...

Continued from previous page...

Atom-1	Atom-2	Interatomic distance (Å)	Clash overlap (Å)
1:D:298:ARG:NH2	1:D:427:TYR:CB	2.64	0.60
1:A:663:LYS:HA	5:A:2262:HOH:O	2.01	0.60
1:A:882:PHE:N	1:A:882:PHE:HD1	1.96	0.60
1:B:499:ASN:HB3	5:B:2219:HOH:O	2.02	0.60
1:D:87:ASP:O	1:D:91:GLU:HG3	2.02	0.60
2:E:9:DA:H2"	2:E:10:DC:OP1	2.00	0.60
1:A:583:GLN:O	1:A:587:ILE:HG12	2.02	0.60
1:A:730:PRO:CD	1:A:786:GLN:NE2	2.64	0.60
1:B:37:LEU:HD22	1:B:288:ALA:HB2	1.84	0.60
1:C:727:TRP:NE1	1:C:735:VAL:HG11	2.17	0.60
1:D:473:VAL:O	1:D:478:ARG:NE	2.20	0.60
1:A:110:LYS:HG2	1:A:112:GLU:OE1	2.02	0.60
1:A:861:MET:CE	1:A:862:PRO:HD2	2.32	0.60
1:B:401:MET:CE	1:B:432:PHE:HB2	2.32	0.60
1:C:226:MET:HA	1:C:250:TYR:HD1	1.66	0.60
1:C:421:ASP:OD2	1:C:423:ARG:NH1	2.34	0.60
1:A:536:PHE:HB3	1:A:882:PHE:CB	2.25	0.60
1:B:178:TYR:HE2	5:B:2095:HOH:O	1.84	0.60
1:B:162:PHE:CD1	1:B:190:MET:SD	2.95	0.60
1:B:752:LEU:N	1:B:752:LEU:HD12	2.16	0.60
1:C:347:CYS:HG	1:C:350:GLU:HG2	1.67	0.60
1:A:6:ILE:HG23	1:A:10:ASP:OD2	2.02	0.59
1:B:262:ALA:HB2	5:B:2059:HOH:O	2.02	0.59
1:B:116:TYR:CE2	1:B:752:LEU:HD22	2.36	0.59
1:C:719:LEU:N	1:C:719:LEU:HD12	2.16	0.59
1:A:110:LYS:O	1:A:114:VAL:HG23	2.01	0.59
1:A:115:ALA:O	1:A:119:ILE:HG12	2.02	0.59
1:A:78:LEU:N	1:A:79:PRO:HD2	2.16	0.59
1:B:307:ARG:HB3	1:B:736:TRP:CZ3	2.38	0.59
1:C:333:LYS:CB	1:C:516:PHE:CD2	2.85	0.59
1:C:54:MET:O	1:C:58:GLN:HG2	2.02	0.59
1:A:30:GLU:CD	1:A:34:ARG:NH2	2.56	0.59
1:B:565:GLU:N	5:B:2252:HOH:O	2.25	0.59
1:D:778:ILE:HG23	1:D:779:ALA:H	1.66	0.59
2:K:6:DT:H5'	2:K:6:DT:C6	2.37	0.59
1:B:539:SER:HB2	1:B:544:GLN:OE1	2.02	0.59
1:B:739:TYR:HB2	1:B:774:GLN:OE1	2.02	0.59
1:C:482:ILE:HD12	1:C:514:PHE:CZ	2.37	0.59
1:C:551:ARG:NH1	1:C:551:ARG:CG	2.52	0.59
2:H:9:DA:H2"	2:H:10:DC:OP1	2.02	0.59
1:A:21:PHE:CD1	1:A:21:PHE:C	2.76	0.59

Continued on next page...

Continued from previous page...

Atom-1	Atom-2	Interatomic distance (Å)	Clash overlap (Å)
1:A:84:ARG:HH21	1:A:222:GLU:CD	2.04	0.59
1:A:236:VAL:HG11	1:A:239:GLN:CG	2.31	0.59
1:A:275:PRO:HD3	1:A:415:TRP:CB	2.32	0.59
1:B:19:ILE:HD12	1:B:20:PRO:HD2	1.83	0.59
1:B:99:ARG:H	1:B:99:ARG:HD2	1.68	0.59
1:C:573:ILE:O	1:C:577:LYS:HG3	2.03	0.59
1:C:854:HIS:CD2	1:C:856:SER:H	2.21	0.59
1:A:120:LYS:HG3	1:A:752:LEU:HD21	1.83	0.59
1:A:30:GLU:OE1	1:A:34:ARG:NH2	2.34	0.59
1:A:438:ASP:OD2	1:A:509:PHE:N	2.35	0.59
1:A:89:PHE:HA	1:A:103:PHE:CE1	2.33	0.59
1:B:517:GLU:O	1:B:521:VAL:HG23	2.02	0.59
1:B:751:PHE:HB3	1:B:752:LEU:CD1	2.33	0.59
1:D:292:ARG:HA	5:D:2075:HOH:O	2.02	0.59
1:D:32:LEU:N	1:D:32:LEU:HD12	2.18	0.59
1:D:81:MET:HE2	1:D:220:LEU:HD13	1.84	0.59
1:D:88:TRP:HA	1:D:91:GLU:OE2	2.02	0.59
1:A:55:PHE:CD2	1:A:59:LEU:HD11	2.37	0.59
1:B:116:TYR:HE1	5:B:2315:HOH:O	1.85	0.59
1:C:14:ILE:HG23	1:C:288:ALA:CB	2.32	0.59
1:C:689:VAL:O	1:C:689:VAL:HG23	2.02	0.59
1:D:227:VAL:HG13	5:D:2059:HOH:O	2.03	0.59
1:D:573:ILE:HD12	1:D:573:ILE:O	2.01	0.59
1:A:814:PHE:CE1	1:A:883:ALA:HB2	2.38	0.59
1:C:492:CYS:SG	1:C:501:TRP:HE3	2.26	0.59
1:D:810:ILE:CG2	3:O:8:U:H5’	2.33	0.59
1:A:269:GLN:HE22	1:A:407:LYS:HZ2	1.46	0.59
1:A:468:ALA:HA	1:A:505:GLN:HB3	1.85	0.59
1:A:649:GLN:O	1:A:653:ASP:HB2	2.03	0.59
1:B:473:VAL:CG1	1:B:477:GLU:HB2	2.33	0.59
1:B:669:GLN:HB3	1:B:672:GLN:HB2	1.85	0.59
1:A:6:ILE:O	1:A:10:ASP:HB3	2.03	0.59
1:A:264:ILE:HG22	1:A:264:ILE:O	2.03	0.59
1:A:47:GLY:O	1:A:50:ARG:HB3	2.02	0.59
1:B:256:THR:HB	5:B:2122:HOH:O	2.03	0.59
1:B:410:ASN:HA	5:B:2177:HOH:O	2.03	0.59
1:C:236:VAL:CB	1:C:239:GLN:HB2	2.33	0.59
1:C:743:ILE:HG12	5:C:2164:HOH:O	2.03	0.59
1:C:881:ALA:O	1:C:883:ALA:N	2.35	0.59
1:D:380:ALA:O	1:D:384:VAL:HG23	2.03	0.59
1:D:663:LYS:HE3	1:D:666:MET:HE1	1.85	0.59

Continued on next page...

Continued from previous page...

Atom-1	Atom-2	Interatomic distance (Å)	Clash overlap (Å)
1:A:170:LEU:HD12	1:A:183:MET:CE	2.33	0.58
1:C:110:LYS:HE2	1:C:112:GLU:OE1	2.02	0.58
1:C:5:ASN:OD1	1:C:7:ALA:HB3	2.02	0.58
1:C:536:PHE:HA	1:C:882:PHE:HD2	1.67	0.58
1:D:462:ILE:HG22	1:D:466:ASN:ND2	2.18	0.58
1:A:142:GLY:HA2	5:A:2072:HOH:O	2.01	0.58
1:A:209:SER:HB2	5:A:2092:HOH:O	2.02	0.58
1:A:236:VAL:CB	1:A:239:GLN:HB2	2.33	0.58
1:B:749:LEU:HD21	5:B:2088:HOH:O	2.03	0.58
1:C:711:LYS:NZ	1:C:711:LYS:CB	2.64	0.58
1:D:432:PHE:CE2	1:D:444:LEU:HD21	2.39	0.58
1:D:437:ASN:ND2	1:D:440:THR:H	2.01	0.58
1:D:582:LEU:CB	1:D:621:LEU:HD21	2.31	0.58
1:A:377:TRP:HB3	5:A:2148:HOH:O	2.03	0.58
1:A:668:THR:HG22	1:A:669:GLN:HE21	1.59	0.58
1:A:806:SER:O	1:A:816:THR:CG2	2.51	0.58
1:B:623:TYR:CA	1:B:666:MET:HE2	2.29	0.58
1:B:787:ASP:OD1	1:B:787:ASP:C	2.41	0.58
1:D:475:PHE:H	1:D:475:PHE:HD1	1.51	0.58
1:D:686:SER:HA	1:D:693:VAL:HG21	1.85	0.58
2:H:12:DT:H2"	2:H:13:DC:C5'	2.33	0.58
1:B:109:ILE:HG13	1:B:149:ALA:HB2	1.84	0.58
1:B:82:ILE:HG22	5:B:2073:HOH:O	2.03	0.58
1:C:188:ALA:HA	5:C:2066:HOH:O	2.03	0.58
1:C:437:ASN:HD21	1:C:440:THR:H	1.50	0.58
1:C:791:LEU:O	1:C:795:VAL:HG23	2.03	0.58
1:D:116:TYR:CD2	1:D:746:ARG:NH2	2.72	0.58
1:D:158:GLU:HG2	1:D:195:LEU:HD13	1.86	0.58
1:A:84:ARG:CG	1:A:223:SER:HB3	2.31	0.58
1:A:854:HIS:HD2	1:A:856:SER:OG	1.86	0.58
1:B:561:LEU:C	1:B:562:LEU:HD23	2.24	0.58
1:C:138:ALA:O	1:C:213:GLY:HA3	2.02	0.58
1:D:534:LEU:HD23	1:D:534:LEU:N	2.19	0.58
2:K:3:DG:H2"	2:K:4:DA:N7	2.18	0.58
4:M:2:DT:H2"	4:M:3:DC:O5'	2.02	0.58
1:A:689:VAL:HG23	1:A:689:VAL:O	2.04	0.58
1:B:43:SER:HA	1:B:46:MET:CE	2.34	0.58
1:C:881:ALA:O	1:C:882:PHE:C	2.42	0.58
1:D:226:MET:HA	1:D:250:TYR:CD1	2.39	0.58
1:A:105:PHE:CD2	1:A:105:PHE:N	2.69	0.58
1:A:70:ALA:C	1:A:72:PRO:HD2	2.24	0.58

Continued on next page...

Continued from previous page...

Atom-1	Atom-2	Interatomic distance (Å)	Clash overlap (Å)
1:B:59:LEU:HA	1:B:64:VAL:CG2	2.32	0.58
1:B:77:LEU:HD21	1:B:226:MET:SD	2.44	0.58
1:C:301:SER:HB2	1:C:303:LYS:HG3	1.86	0.58
1:C:551:ARG:HB2	1:C:868:GLY:H	1.68	0.58
1:D:226:MET:HA	1:D:250:TYR:HD1	1.68	0.58
1:D:286:TYR:CE1	1:D:417:PRO:HG3	2.39	0.58
1:D:609:VAL:HG12	5:D:2148:HOH:O	2.03	0.58
1:D:551:ARG:NH2	1:D:836:TYR:O	2.22	0.58
1:A:881:ALA:O	1:A:883:ALA:N	2.37	0.58
1:C:229:LEU:HD13	1:C:244:ILE:HD13	1.85	0.58
1:D:348:PRO:CG	5:D:2092:HOH:O	2.32	0.58
2:H:17:DG:H2”	2:H:18:DC:C6	2.38	0.58
1:A:163:LYS:HB3	1:A:164:LYS:NZ	2.18	0.58
1:A:571:TYR:CD1	1:A:634:VAL:CG1	2.87	0.58
1:A:632:ARG:NH1	1:A:632:ARG:CB	2.62	0.58
1:A:116:TYR:HE2	1:A:752:LEU:HD22	1.68	0.58
1:C:56:GLU:OE1	1:C:57:ARG:N	2.36	0.58
1:D:617:ALA:O	1:D:621:LEU:HG	2.03	0.58
1:A:423:ARG:NH2	1:A:784:HIS:ND1	2.50	0.58
1:B:313:MET:HE3	5:B:2143:HOH:O	2.04	0.58
1:B:479:ILE:O	1:B:483:GLU:HG3	2.03	0.58
1:B:748:ASN:ND2	1:B:752:LEU:N	2.52	0.58
1:B:155:ARG:CZ	1:B:749:LEU:HD23	2.34	0.58
1:C:825:PHE:O	1:C:829:ARG:NH1	2.37	0.58
1:D:6:ILE:O	1:D:10:ASP:HB3	2.04	0.58
1:D:182:PHE:O	1:D:186:VAL:HG23	2.02	0.58
1:D:677:MET:HG3	1:D:681:ILE:HD11	1.85	0.58
1:D:475:PHE:HE1	1:D:880:PHE:CD1	2.20	0.58
2:K:17:DG:H2”	2:K:18:DC:C6	2.39	0.58
1:A:552:ASP:HB2	1:A:691:ALA:HB2	1.86	0.57
1:A:806:SER:O	1:A:816:THR:HG23	2.03	0.57
1:C:120:LYS:NZ	1:C:752:LEU:HD11	2.19	0.57
1:C:829:ARG:HH11	1:C:829:ARG:HG3	1.69	0.57
1:D:352:ILE:HG22	5:D:2101:HOH:O	2.04	0.57
4:G:2:DT:H2’	5:G:2001:HOH:O	2.04	0.57
2:N:5:DA:H1’	2:N:6:DT:C5’	2.34	0.57
1:B:6:ILE:HG23	1:B:10:ASP:CG	2.24	0.57
1:B:746:ARG:HB3	1:B:746:ARG:CZ	2.33	0.57
1:C:105:PHE:HE1	1:C:208:ASP:HB3	1.69	0.57
1:A:229:LEU:HD13	1:A:244:ILE:HD13	1.85	0.57
1:B:314:PRO:HD2	5:B:2144:HOH:O	2.04	0.57

Continued on next page...

Continued from previous page...

Atom-1	Atom-2	Interatomic distance (Å)	Clash overlap (Å)
1:B:433:ASN:HB2	1:B:434:PRO:CD	2.34	0.57
1:B:475:PHE:CE1	1:B:478:ARG:NH1	2.72	0.57
2:N:17:DG:H2"	2:N:18:DC:C6	2.39	0.57
1:A:828:VAL:CG1	1:A:883:ALA:HA	2.34	0.57
1:A:829:ARG:HG3	1:A:829:ARG:HH11	1.69	0.57
1:B:187:GLU:CD	1:B:199:GLU:OE2	2.43	0.57
1:B:419:ASN:HD22	1:B:419:ASN:N	2.01	0.57
1:B:671:ASN:HB2	5:B:2297:HOH:O	2.03	0.57
1:C:281:ILE:CG2	1:C:282:THR:HG23	2.32	0.57
1:C:551:ARG:NH1	1:C:551:ARG:HG2	2.19	0.57
1:C:711:LYS:HB2	1:C:711:LYS:HZ3	1.67	0.57
1:C:777:GLY:O	1:C:781:ASN:HB2	2.05	0.57
1:C:425:ARG:HH21	1:C:784:HIS:CD2	2.23	0.57
1:D:105:PHE:HB3	5:D:2033:HOH:O	2.03	0.57
1:D:555:GLY:O	1:D:559:VAL:HG22	2.05	0.57
1:D:583:GLN:O	1:D:587:ILE:HG12	2.02	0.57
1:D:873:ARG:HH11	1:D:876:LEU:HD11	1.69	0.57
2:H:7:DC:H5'	5:H:2008:HOH:O	2.05	0.57
1:B:532:LEU:HD12	1:B:533:PRO:CD	2.30	0.57
1:C:491:ALA:HB1	1:C:499:ASN:ND2	2.19	0.57
1:D:74:ILE:HD12	5:D:2027:HOH:O	2.04	0.57
1:D:551:ARG:HB2	1:D:868:GLY:H	1.69	0.57
1:D:832:MET:HG2	1:D:875:ILE:HD13	1.85	0.57
1:D:881:ALA:O	1:D:883:ALA:N	2.37	0.57
1:A:452:ILE:HD12	1:A:818:PRO:CB	2.34	0.57
1:B:15:GLU:OE2	1:B:18:ALA:O	2.22	0.57
1:B:326:THR:HG23	1:B:806:SER:HA	1.86	0.57
1:C:158:GLU:OE2	1:C:195:LEU:HB3	2.04	0.57
1:C:681:ILE:O	1:C:685:VAL:HG13	2.05	0.57
1:C:810:ILE:O	1:C:810:ILE:HG22	2.03	0.57
1:D:134:VAL:HG12	1:D:242:GLU:O	2.04	0.57
1:D:322:ILE:HG13	5:D:2167:HOH:O	2.04	0.57
1:D:437:ASN:C	1:D:437:ASN:HD22	2.08	0.57
1:A:111:PRO:HG2	1:A:112:GLU:OE2	2.04	0.57
1:A:164:LYS:HZ3	1:A:164:LYS:N	2.03	0.57
1:B:51:PHE:C	1:B:51:PHE:CD2	2.78	0.57
1:C:719:LEU:N	1:C:719:LEU:CD1	2.68	0.57
1:C:96:ARG:HG2	1:C:96:ARG:NH1	2.18	0.57
1:D:154:ILE:HG23	1:D:190:MET:CE	2.33	0.57
1:A:338:ALA:O	1:A:342:THR:HG23	2.04	0.57
1:C:172:LYS:HD2	3:L:3:G:H5"	1.85	0.57

Continued on next page...

Continued from previous page...

Atom-1	Atom-2	Interatomic distance (Å)	Clash overlap (Å)
1:C:463:HIS:HA	1:C:466:ASN:HD22	1.68	0.57
1:D:604:GLU:HG3	5:D:2141:HOH:O	2.04	0.57
1:A:204:TRP:HA	1:A:208:ASP:OD2	2.04	0.57
1:A:332:LYS:CE	1:A:410:ASN:HD22	2.18	0.57
1:A:347:CYS:CB	1:A:350:GLU:HG3	2.34	0.57
1:A:78:LEU:O	1:A:82:ILE:HG13	2.05	0.57
1:C:30:GLU:HA	5:C:2031:HOH:O	2.05	0.57
1:C:860:LYS:HB2	5:C:2182:HOH:O	2.05	0.57
1:C:96:ARG:H	1:C:96:ARG:HD2	1.69	0.57
1:B:389:LYS:HA	1:B:392:LYS:HE2	1.86	0.57
1:B:573:ILE:HD12	1:B:574:VAL:N	2.19	0.57
1:C:312:TYR:HA	5:C:2087:HOH:O	2.05	0.57
1:D:281:ILE:HD11	1:D:308:TYR:CB	2.34	0.57
1:D:52:ARG:HG3	1:D:52:ARG:HH21	1.70	0.57
1:A:213:GLY:O	1:A:217:ILE:HG13	2.05	0.56
1:C:154:ILE:HG23	1:C:190:MET:CE	2.32	0.56
1:C:555:GLY:HA3	5:C:2126:HOH:O	2.05	0.56
1:C:563:PRO:HB3	1:C:877:GLU:O	2.04	0.56
1:C:810:ILE:CG2	3:L:8:U:H5’	2.35	0.56
1:C:882:PHE:N	1:C:882:PHE:HD1	2.03	0.56
2:E:12:DT:H2”	2:E:13:DC:C5’	2.35	0.56
1:B:336:ALA:O	1:B:340:VAL:HG23	2.04	0.56
1:C:199:GLU:HG2	1:C:201:TRP:CD1	2.37	0.56
1:C:502:TRP:CG	1:C:512:LEU:HD13	2.40	0.56
1:D:475:PHE:N	1:D:476:PRO:HD2	2.20	0.56
4:J:4:DG:H2”	4:J:5:DA:H8	1.68	0.56
1:A:214:VAL:O	1:A:218:GLU:HG3	2.05	0.56
1:A:56:GLU:C	1:A:56:GLU:OE1	2.43	0.56
1:B:158:GLU:HA	1:B:195:LEU:CD2	2.36	0.56
1:B:84:ARG:HH11	1:B:84:ARG:HA	1.68	0.56
1:C:633:SER:HB3	1:C:646:PHE:CE1	2.41	0.56
2:E:6:DT:H2”	2:E:7:DC:C5	2.40	0.56
1:A:155:ARG:CB	1:A:749:LEU:HD21	2.34	0.56
1:A:211:HIS:HB2	5:A:2093:HOH:O	2.05	0.56
1:B:6:ILE:O	1:B:10:ASP:HB3	2.05	0.56
1:B:417:PRO:HG2	1:B:429:VAL:HB	1.87	0.56
1:B:710:VAL:CG1	1:B:720:ARG:HB3	2.36	0.56
1:B:84:ARG:HD2	1:B:219:MET:CB	2.33	0.56
1:B:873:ARG:HD2	5:B:2345:HOH:O	2.03	0.56
1:D:406:ASN:HB2	5:D:2112:HOH:O	2.04	0.56
1:D:632:ARG:HA	1:D:635:MET:HG2	1.86	0.56

Continued on next page...

Continued from previous page...

Atom-1	Atom-2	Interatomic distance (Å)	Clash overlap (Å)
1:D:719:LEU:HD12	1:D:719:LEU:N	2.21	0.56
1:D:737:GLN:HE22	1:D:778:ILE:HA	1.68	0.56
4:M:5:DA:H2”	4:M:6:DT:H72	1.87	0.56
1:A:118:THR:CG2	1:A:216:CYS:HB3	2.35	0.56
1:A:84:ARG:NH2	1:A:222:GLU:HB3	2.20	0.56
1:A:391:ARG:NH1	1:A:391:ARG:HG2	2.19	0.56
1:B:342:THR:HG22	1:B:348:PRO:HG3	1.87	0.56
1:C:778:ILE:HG23	1:C:779:ALA:N	2.19	0.56
1:D:422:TRP:C	1:D:422:TRP:CD1	2.79	0.56
1:A:487:GLU:OE1	1:A:487:GLU:HA	2.05	0.56
1:A:663:LYS:CG	1:A:664:GLY:N	2.68	0.56
1:A:721:LYS:HE2	5:A:2268:HOH:O	2.06	0.56
1:B:14:ILE:CG1	5:B:2134:HOH:O	2.54	0.56
1:B:89:PHE:O	1:B:93:LYS:HG3	2.05	0.56
1:C:6:ILE:O	1:C:10:ASP:HB3	2.06	0.56
1:D:43:SER:CA	1:D:46:MET:HE2	2.31	0.56
1:D:52:ARG:HG3	1:D:52:ARG:NH2	2.20	0.56
1:A:109:ILE:CG1	1:A:149:ALA:HB2	2.36	0.56
1:A:176:HIS:HA	5:A:2081:HOH:O	2.05	0.56
1:A:669:GLN:NE2	1:A:669:GLN:N	2.53	0.56
1:A:92:VAL:HG11	1:A:100:PRO:HD2	1.87	0.56
1:B:450:LYS:HE2	1:B:817:ILE:HD11	1.88	0.56
1:C:871:ASN:ND2	1:C:873:ARG:HB2	2.21	0.56
1:D:335:LEU:CD2	1:D:339:ASN:ND2	2.69	0.56
2:K:2:DG:H5’	5:K:2004:HOH:O	2.06	0.56
1:A:227:VAL:HB	1:A:244:ILE:CG2	2.35	0.56
1:A:312:TYR:CZ	1:A:314:PRO:HG3	2.41	0.56
1:B:854:HIS:HD2	1:B:856:SER:OG	1.89	0.56
1:D:339:ASN:O	1:D:343:LYS:CD	2.53	0.56
1:D:811:HIS:HD2	5:D:2168:HOH:O	1.89	0.56
4:G:4:DG:H2”	4:G:5:DA:C8	2.41	0.56
1:A:215:ARG:HG2	5:A:2065:HOH:O	2.05	0.56
1:A:6:ILE:HG23	1:A:10:ASP:CG	2.25	0.56
1:B:67:ASN:O	1:B:71:LYS:HG3	2.06	0.56
1:C:711:LYS:HG3	1:C:718:ILE:HA	1.86	0.56
1:C:726:HIS:HD2	1:C:735:VAL:O	1.89	0.56
1:D:268:PHE:HB3	1:D:286:TYR:OH	2.05	0.56
1:A:473:VAL:O	1:A:478:ARG:NE	2.36	0.56
1:A:592:ASN:OD1	1:A:611:LEU:CD2	2.53	0.56
1:A:778:ILE:HG23	1:A:779:ALA:N	2.21	0.56
1:B:21:PHE:C	1:B:23:THR:H	2.09	0.56

Continued on next page...

Continued from previous page...

Atom-1	Atom-2	Interatomic distance (Å)	Clash overlap (Å)
1:B:349:VAL:HG12	1:B:349:VAL:O	2.06	0.56
1:B:473:VAL:CG1	1:B:477:GLU:HB3	2.34	0.56
1:C:346:HIS:CE1	1:C:391:ARG:NH2	2.74	0.56
1:C:631:LYS:O	1:C:634:VAL:HG13	2.05	0.56
1:A:218:GLU:O	1:A:222:GLU:HG3	2.06	0.56
1:A:580:GLU:OE2	5:A:2231:HOH:O	2.17	0.56
1:B:281:ILE:CG2	1:B:282:THR:HG23	2.33	0.56
1:B:423:ARG:NE	1:B:781:ASN:ND2	2.53	0.56
1:B:698:TRP:CZ2	1:B:864:LEU:HD21	2.41	0.56
1:C:313:MET:HA	5:C:2156:HOH:O	2.06	0.56
1:C:631:LYS:HE2	1:C:635:MET:SD	2.46	0.56
1:C:829:ARG:HD3	1:C:875:ILE:O	2.05	0.56
1:D:318:LYS:NZ	1:D:800:GLU:OE2	2.34	0.56
1:A:182:PHE:HB2	5:A:2082:HOH:O	2.05	0.55
1:B:324:GLN:CG	1:B:417:PRO:HA	2.36	0.55
1:B:657:PRO:HA	5:B:2292:HOH:O	2.06	0.55
1:C:155:ARG:NH1	1:C:155:ARG:HB3	2.21	0.55
1:D:335:LEU:HD11	1:D:406:ASN:OD1	2.06	0.55
1:C:2:ASN:N	5:C:2003:HOH:O	2.40	0.55
1:C:78:LEU:N	1:C:79:PRO:HD2	2.22	0.55
1:D:213:GLY:O	1:D:217:ILE:HG13	2.06	0.55
1:D:428:ALA:H	1:D:435:GLN:HE22	1.54	0.55
1:A:595:VAL:HG13	5:A:2237:HOH:O	2.06	0.55
1:B:308:TYR:HA	1:B:311:VAL:HG23	1.87	0.55
1:B:332:LYS:N	5:B:2151:HOH:O	2.35	0.55
1:C:791:LEU:HD21	1:C:809:LEU:HD22	1.87	0.55
1:D:201:TRP:O	1:D:204:TRP:HB2	2.05	0.55
1:D:220:LEU:O	1:D:220:LEU:HD12	2.05	0.55
1:D:711:LYS:HA	1:D:719:LEU:HD13	1.87	0.55
4:M:6:DT:H2"	4:M:7:DT:OP2	2.07	0.55
1:A:210:ILE:O	1:A:214:VAL:HG23	2.07	0.55
1:B:170:LEU:CD2	1:B:179:LYS:HD3	2.37	0.55
1:B:422:TRP:CD1	1:B:422:TRP:O	2.59	0.55
1:C:553:GLU:HG2	1:C:554:VAL:N	2.22	0.55
1:C:614:LYS:HE2	5:C:2137:HOH:O	2.06	0.55
1:D:231:ARG:HG2	1:D:234:ALA:HB2	1.88	0.55
1:D:462:ILE:HG22	1:D:466:ASN:HD21	1.71	0.55
1:A:230:HIS:HE2	1:A:245:GLU:CG	2.19	0.55
1:B:374:LEU:N	1:B:374:LEU:HD12	2.21	0.55
1:B:391:ARG:NH1	1:B:391:ARG:HB3	2.21	0.55
1:B:148:GLU:OE2	1:B:749:LEU:HB2	2.05	0.55

Continued on next page...

Continued from previous page...

Atom-1	Atom-2	Interatomic distance (Å)	Clash overlap (Å)
1:C:373:ALA:O	1:C:377:TRP:CD1	2.60	0.55
1:C:272:VAL:O	1:C:415:TRP:HZ3	1.89	0.55
1:C:71:LYS:N	1:C:72:PRO:HD2	2.22	0.55
1:D:342:THR:HA	5:D:2092:HOH:O	2.07	0.55
1:A:105:PHE:HE1	5:A:2093:HOH:O	1.89	0.55
1:B:158:GLU:HB3	1:B:190:MET:CE	2.36	0.55
1:B:401:MET:HE1	1:B:432:PHE:CA	2.36	0.55
1:B:669:GLN:HG2	1:B:672:GLN:HE21	1.71	0.55
1:B:99:ARG:HG2	1:B:103:PHE:CE2	2.42	0.55
1:C:230:HIS:CD2	1:C:230:HIS:H	2.24	0.55
1:C:432:PHE:CE1	1:C:440:THR:HG23	2.42	0.55
1:D:150:ARG:HG3	1:D:201:TRP:NE1	2.20	0.55
1:D:59:LEU:HA	1:D:64:VAL:HG22	1.87	0.55
1:A:179:LYS:HD3	5:A:2081:HOH:O	2.06	0.55
1:A:633:SER:HB3	1:A:646:PHE:CD1	2.42	0.55
1:A:551:ARG:NH2	1:A:836:TYR:O	2.39	0.55
1:B:158:GLU:HA	1:B:195:LEU:HD13	1.89	0.55
1:B:676:TYR:O	1:B:679:LYS:HB3	2.06	0.55
1:C:291:ARG:C	1:C:293:PRO:HD3	2.26	0.55
1:D:278:TRP:CD2	1:D:284:GLY:HA3	2.42	0.55
1:D:428:ALA:H	1:D:435:GLN:NE2	2.04	0.55
1:D:448:LYS:HE3	5:D:2087:HOH:O	2.07	0.55
2:K:2:DG:C2’	2:K:3:DG:C8	2.89	0.55
1:A:169:GLN:HB2	1:A:182:PHE:CZ	2.42	0.55
1:A:448:LYS:HG2	5:A:2287:HOH:O	2.07	0.55
1:A:714:LYS:HZ3	1:A:714:LYS:HA	1.71	0.55
1:B:126:LEU:HA	5:B:2083:HOH:O	2.05	0.55
1:B:157:LEU:HG	1:B:158:GLU:N	2.21	0.55
1:B:711:LYS:HB3	1:B:717:GLU:O	2.07	0.55
1:C:324:GLN:HE21	1:C:418:TYR:N	2.05	0.55
1:D:420:MET:HA	1:D:425:ARG:O	2.07	0.55
1:A:154:ILE:HA	1:A:158:GLU:HB2	1.88	0.55
1:A:313:MET:HB2	1:A:316:VAL:CG2	2.37	0.55
1:B:159:ALA:O	1:B:163:LYS:HB2	2.06	0.55
1:B:315:GLU:OE2	1:B:318:LYS:HD3	2.07	0.55
1:B:99:ARG:HB3	1:B:103:PHE:CD2	2.42	0.55
1:C:21:PHE:C	1:C:23:THR:H	2.10	0.55
1:C:16:LEU:HD13	1:C:38:ALA:HB2	1.88	0.55
1:C:422:TRP:CE2	1:C:423:ARG:HD2	2.41	0.55
1:C:553:GLU:HG2	1:C:554:VAL:H	1.72	0.55
1:C:882:PHE:O	1:C:883:ALA:CB	2.54	0.55

Continued on next page...

Continued from previous page...

Atom-1	Atom-2	Interatomic distance (Å)	Clash overlap (Å)
1:D:158:GLU:OE1	1:D:195:LEU:HD22	2.07	0.55
1:D:327:ALA:HB3	1:D:448:LYS:HE2	1.88	0.55
1:D:552:ASP:OD1	1:D:555:GLY:N	2.38	0.55
2:K:4:DA:H2"	2:K:5:DA:OP2	2.07	0.55
1:A:110:LYS:HE2	1:A:112:GLU:OE1	2.07	0.55
1:B:632:ARG:HD3	1:B:632:ARG:H	1.71	0.55
1:C:120:LYS:HG3	1:C:752:LEU:HD21	1.89	0.55
1:D:14:ILE:HG22	5:D:2014:HOH:O	2.06	0.55
1:D:69:ALA:HA	1:D:257:ARG:HD2	1.89	0.55
1:D:84:ARG:CG	1:D:84:ARG:HH11	2.19	0.55
2:K:6:DT:H2"	2:K:7:DC:C5	2.42	0.55
1:A:142:GLY:CA	5:A:2072:HOH:O	2.55	0.54
1:B:489:ILE:CG2	1:B:518:TYR:CD1	2.88	0.54
1:B:567:VAL:HG22	1:B:880:PHE:CG	2.43	0.54
1:C:36:GLN:HA	1:C:36:GLN:OE1	2.05	0.54
1:C:434:PRO:HG3	1:C:444:LEU:HD13	1.89	0.54
1:C:711:LYS:HG2	1:C:718:ILE:CA	2.36	0.54
1:C:754:GLN:HG2	5:C:2160:HOH:O	2.05	0.54
1:D:227:VAL:HB	1:D:244:ILE:HG23	1.89	0.54
1:D:150:ARG:HA	1:D:201:TRP:CZ2	2.42	0.54
1:D:551:ARG:NH1	1:D:872:LEU:CD1	2.71	0.54
1:A:804:ILE:HG12	1:A:820:ASP:HB3	1.89	0.54
1:B:12:SER:HA	5:B:2033:HOH:O	2.06	0.54
1:B:804:ILE:HG12	1:B:820:ASP:HB3	1.89	0.54
1:B:80:LYS:HD3	1:B:223:SER:O	2.08	0.54
1:C:656:GLN:HB3	1:C:657:PRO:HD3	1.89	0.54
1:C:676:TYR:O	1:C:679:LYS:HB3	2.08	0.54
1:D:713:LYS:NZ	1:D:713:LYS:HA	2.22	0.54
1:D:794:THR:HG21	1:D:828:VAL:HG12	1.89	0.54
1:D:873:ARG:NH1	1:D:876:LEU:HD11	2.23	0.54
1:A:204:TRP:HZ2	5:A:2067:HOH:O	1.89	0.54
1:A:230:HIS:CD2	1:A:230:HIS:H	2.25	0.54
1:A:571:TYR:CD1	1:A:631:LYS:HA	2.42	0.54
1:A:826:LYS:HG2	1:A:830:GLU:OE2	2.08	0.54
1:B:272:VAL:CG1	1:B:411:HIS:HD2	2.20	0.54
1:C:395:ARG:O	1:C:399:GLU:HG3	2.07	0.54
1:C:475:PHE:N	1:C:476:PRO:HD2	2.22	0.54
1:C:846:TYR:HA	1:C:849:PHE:CE1	2.43	0.54
1:D:217:ILE:O	1:D:221:ILE:HG13	2.07	0.54
1:A:428:ALA:HB3	1:A:433:ASN:HD22	1.72	0.54
1:A:726:HIS:HB2	1:A:736:TRP:CD1	2.43	0.54

Continued on next page...

Continued from previous page...

Atom-1	Atom-2	Interatomic distance (Å)	Clash overlap (Å)
1:B:216:CYS:HA	1:B:219:MET:HE3	1.90	0.54
1:B:401:MET:HE2	1:B:432:PHE:HD1	1.73	0.54
1:B:710:VAL:HG12	1:B:720:ARG:HB3	1.89	0.54
1:B:73:LEU:CD1	1:B:254:ILE:HG13	2.38	0.54
1:C:702:ALA:O	1:C:706:LEU:HD12	2.07	0.54
1:C:790:HIS:NE2	1:C:832:MET:HB2	2.22	0.54
1:D:169:GLN:O	1:D:173:ARG:HG2	2.07	0.54
1:B:155:ARG:HB3	5:B:2088:HOH:O	2.07	0.54
1:C:47:GLY:O	1:C:50:ARG:HB3	2.08	0.54
1:C:514:PHE:C	1:C:514:PHE:CD1	2.80	0.54
1:C:57:ARG:HD2	5:C:2041:HOH:O	2.07	0.54
1:D:134:VAL:HA	5:D:2038:HOH:O	2.06	0.54
1:D:56:GLU:OE1	1:D:60:LYS:HB2	2.06	0.54
1:D:56:GLU:OE2	1:D:57:ARG:N	2.41	0.54
1:D:84:ARG:HD3	1:D:84:ARG:O	2.06	0.54
1:A:14:ILE:HG23	1:A:288:ALA:CB	2.37	0.54
1:A:468:ALA:HB3	5:A:2175:HOH:O	2.08	0.54
1:A:71:LYS:N	1:A:72:PRO:CD	2.71	0.54
1:A:881:ALA:CA	5:A:2310:HOH:O	2.55	0.54
1:B:130:ASP:O	1:B:132:THR:HG23	2.07	0.54
1:B:221:ILE:HG12	1:B:227:VAL:HG23	1.89	0.54
1:B:21:PHE:C	1:B:23:THR:N	2.61	0.54
1:B:264:ILE:O	1:B:264:ILE:HG22	2.08	0.54
1:D:108:GLU:HG3	5:D:2034:HOH:O	2.07	0.54
1:D:869:ASN:N	1:D:869:ASN:HD22	2.06	0.54
2:E:17:DG:H2"	2:E:18:DC:C6	2.41	0.54
1:A:6:ILE:HB	1:A:48:GLU:OE2	2.08	0.54
1:A:814:PHE:N	1:A:814:PHE:CD1	2.75	0.54
1:B:18:ALA:O	1:B:19:ILE:HG12	2.08	0.54
1:B:432:PHE:CZ	1:B:444:LEU:HD21	2.43	0.54
1:B:720:ARG:CG	1:B:720:ARG:HH11	2.06	0.54
1:C:19:ILE:O	1:C:21:PHE:N	2.39	0.54
1:C:229:LEU:HD11	1:C:242:GLU:HG2	1.89	0.54
1:D:80:LYS:CD	1:D:224:THR:HG22	2.37	0.54
1:A:308:TYR:O	1:A:311:VAL:HG23	2.08	0.54
1:A:486:HIS:HD2	1:A:518:TYR:OH	1.90	0.54
1:B:21:PHE:O	1:B:21:PHE:CD1	2.59	0.54
1:B:391:ARG:CB	1:B:391:ARG:HH11	2.20	0.54
1:B:404:GLN:HG2	1:B:432:PHE:HB3	1.90	0.54
1:B:51:PHE:C	1:B:51:PHE:HD2	2.10	0.54
1:B:631:LYS:HD3	1:B:632:ARG:HH11	1.72	0.54

Continued on next page...

Continued from previous page...

Atom-1	Atom-2	Interatomic distance (Å)	Clash overlap (Å)
1:C:324:GLN:HE21	1:C:417:PRO:HA	1.73	0.54
1:D:446:LEU:HD12	1:D:817:ILE:HG22	1.90	0.54
1:D:824:LEU:O	1:D:828:VAL:HG13	2.08	0.54
1:D:814:PHE:CE1	1:D:883:ALA:HB2	2.43	0.54
1:B:644:PHE:CE2	2:H:9:DA:C4	2.95	0.54
3:I:4:G:H5'	5:I:2003:HOH:O	2.07	0.54
1:A:341:ILE:HD12	1:A:348:PRO:HB3	1.90	0.54
1:A:713:LYS:C	1:A:713:LYS:HE3	2.28	0.54
1:C:825:PHE:CZ	1:C:829:ARG:NH2	2.76	0.54
1:D:230:HIS:H	1:D:230:HIS:CD2	2.26	0.54
3:F:6:G:N7	5:F:2013:HOH:O	2.34	0.54
1:A:422:TRP:CD1	1:A:422:TRP:O	2.62	0.53
1:A:623:TYR:HD1	1:A:663:LYS:HE3	1.72	0.53
1:A:669:GLN:CA	1:A:669:GLN:HE21	2.21	0.53
1:B:115:ALA:O	1:B:119:ILE:HG12	2.09	0.53
1:C:646:PHE:HD1	1:C:649:GLN:OE1	1.90	0.53
1:D:16:LEU:HD22	1:D:38:ALA:HA	1.90	0.53
1:B:116:TYR:CD1	1:B:746:ARG:NH2	2.76	0.53
1:B:754:GLN:HG3	5:B:2065:HOH:O	2.07	0.53
1:B:99:ARG:HD2	1:B:99:ARG:N	2.23	0.53
1:C:473:VAL:HG13	1:C:477:GLU:HB2	1.89	0.53
1:C:582:LEU:HB3	1:C:621:LEU:HD21	1.90	0.53
1:C:92:VAL:CG1	1:C:99:ARG:HG3	2.38	0.53
1:D:58:GLN:HB3	5:D:2026:HOH:O	2.09	0.53
1:A:109:ILE:H	1:A:109:ILE:HD12	1.71	0.53
1:A:63:GLU:HA	5:A:2057:HOH:O	2.08	0.53
1:A:80:LYS:HE2	1:A:223:SER:O	2.08	0.53
1:A:842:LEU:O	1:A:845:PHE:HB3	2.08	0.53
1:B:651:LEU:O	1:B:656:GLN:HB2	2.09	0.53
1:C:120:LYS:HZ3	1:C:752:LEU:CD1	2.20	0.53
1:C:570:ILE:HA	1:C:573:ILE:CG2	2.37	0.53
1:D:162:PHE:HB2	5:D:2045:HOH:O	2.07	0.53
1:A:161:HIS:O	1:A:164:LYS:HG2	2.08	0.53
1:B:50:ARG:HH21	1:B:267:MET:HG2	1.72	0.53
1:B:396:ILE:HB	5:B:2174:HOH:O	2.08	0.53
1:B:416:PHE:HD1	1:B:430:SER:OG	1.90	0.53
1:B:552:ASP:O	1:B:870:LEU:HD12	2.08	0.53
1:B:748:ASN:HD22	1:B:752:LEU:N	2.05	0.53
1:B:7:ALA:HB1	5:B:2057:HOH:O	2.09	0.53
1:C:551:ARG:HE	1:C:872:LEU:HD21	1.72	0.53
1:D:32:LEU:CD1	1:D:32:LEU:N	2.71	0.53

Continued on next page...

Continued from previous page...

Atom-1	Atom-2	Interatomic distance (Å)	Clash overlap (Å)
1:D:349:VAL:C	5:D:2096:HOH:O	2.46	0.53
1:A:100:PRO:HG2	1:A:103:PHE:CB	2.35	0.53
1:B:278:TRP:CD2	1:B:284:GLY:HA3	2.44	0.53
1:B:324:GLN:HE21	1:B:418:TYR:N	2.06	0.53
1:C:182:PHE:O	1:C:185:VAL:HG23	2.09	0.53
1:C:205:HIS:O	1:C:207:GLU:N	2.41	0.53
1:A:141:ILE:O	1:A:145:ILE:HG12	2.08	0.53
1:A:652:GLU:HA	1:A:656:GLN:HB2	1.91	0.53
1:B:155:ARG:O	1:B:155:ARG:HG2	2.07	0.53
1:B:162:PHE:CE1	1:B:190:MET:SD	3.02	0.53
1:B:726:HIS:CD2	1:B:736:TRP:NE1	2.76	0.53
1:B:797:TRP:CH2	1:B:801:LYS:HG3	2.42	0.53
1:B:840:ASP:O	1:B:842:LEU:N	2.42	0.53
1:C:64:VAL:HG21	1:C:127:THR:HG21	1.90	0.53
1:D:151:PHE:CD1	1:D:183:MET:HB3	2.44	0.53
1:D:379:ARG:HA	5:D:2109:HOH:O	2.09	0.53
1:D:37:LEU:HD13	5:D:2014:HOH:O	2.09	0.53
1:D:414:ILE:HA	5:D:2115:HOH:O	2.08	0.53
1:D:743:ILE:HG13	1:D:766:ASP:O	2.08	0.53
1:D:730:PRO:CD	1:D:786:GLN:HE22	2.21	0.53
2:E:7:DC:C4	2:E:8:DG:N7	2.77	0.53
1:A:21:PHE:C	1:A:23:THR:H	2.12	0.53
1:A:846:TYR:HD2	1:A:849:PHE:CZ	2.27	0.53
1:B:158:GLU:HB3	1:B:190:MET:HE1	1.90	0.53
1:B:346:HIS:HA	1:B:395:ARG:NH1	2.23	0.53
1:B:712:ASP:OD2	1:B:714:LYS:HB2	2.09	0.53
1:C:164:LYS:CA	1:C:164:LYS:HE2	2.39	0.53
1:C:205:HIS:C	1:C:207:GLU:N	2.62	0.53
1:C:227:VAL:CG1	1:C:244:ILE:HG22	2.38	0.53
1:C:544:GLN:HG2	1:C:561:LEU:HD13	1.90	0.53
1:D:32:LEU:CD1	1:D:32:LEU:H	2.22	0.53
1:D:486:HIS:C	1:D:486:HIS:HD1	2.12	0.53
1:A:108:GLU:OE1	1:A:108:GLU:HA	2.09	0.53
1:C:180:LYS:HG2	1:C:184:GLN:OE1	2.08	0.53
1:C:146:GLU:HB2	1:C:204:TRP:CZ3	2.43	0.53
1:D:319:ALA:HB1	1:D:792:ARG:HG2	1.91	0.53
4:P:4:DG:H2"	4:P:5:DA:H8	1.71	0.53
1:A:114:VAL:HG13	1:A:145:ILE:HD12	1.91	0.53
1:A:308:TYR:CA	1:A:311:VAL:HG23	2.39	0.53
1:B:719:LEU:HD12	1:B:719:LEU:N	2.23	0.53
1:B:537:ASP:N	1:B:882:PHE:HD2	2.07	0.53

Continued on next page...

Continued from previous page...

Atom-1	Atom-2	Interatomic distance (Å)	Clash overlap (Å)
1:D:201:TRP:HA	1:D:204:TRP:CD1	2.44	0.53
1:D:36:GLN:HG3	1:D:273:VAL:CG2	2.38	0.53
2:K:15:DC:H2"	2:K:16:DC:O5'	2.08	0.53
1:A:19:ILE:O	1:A:21:PHE:N	2.39	0.53
1:B:44:TYR:OH	1:B:292:ARG:HB3	2.08	0.53
1:C:35:GLU:OE2	1:C:272:VAL:HG11	2.09	0.53
1:C:416:PHE:CE2	1:C:434:PRO:HD3	2.44	0.53
1:C:5:ASN:HD21	1:C:7:ALA:C	2.12	0.53
1:C:854:HIS:HD2	1:C:856:SER:OG	1.92	0.53
1:D:412:LYS:N	5:D:2114:HOH:O	2.36	0.53
1:A:217:ILE:HG22	1:A:221:ILE:CD1	2.38	0.52
1:A:134:VAL:CG2	1:A:244:ILE:HD11	2.39	0.52
1:A:656:GLN:HB3	1:A:657:PRO:CD	2.39	0.52
1:A:82:ILE:HG21	5:A:2068:HOH:O	2.10	0.52
1:B:393:SER:HB3	5:H:2017:HOH:O	2.09	0.52
1:B:490:MET:HE2	1:B:522:GLN:HG3	1.91	0.52
1:B:614:LYS:HB3	5:B:2261:HOH:O	2.08	0.52
1:B:551:ARG:HB2	1:B:868:GLY:H	1.72	0.52
1:C:68:ALA:CB	1:C:261:LEU:HD21	2.39	0.52
1:D:71:LYS:N	1:D:72:PRO:HD2	2.25	0.52
1:A:105:PHE:CE1	1:A:208:ASP:HB3	2.44	0.52
1:A:395:ARG:HG3	1:A:395:ARG:O	2.09	0.52
1:A:423:ARG:NE	1:A:781:ASN:ND2	2.56	0.52
1:B:105:PHE:HB3	1:B:204:TRP:CZ2	2.45	0.52
1:B:231:ARG:HD2	1:B:240:ASP:OD1	2.09	0.52
1:B:257:ARG:HB2	5:B:2123:HOH:O	2.09	0.52
1:B:39:LEU:HD13	1:B:272:VAL:CG2	2.39	0.52
1:B:573:ILE:CD1	1:B:573:ILE:C	2.60	0.52
1:C:120:LYS:HG3	1:C:752:LEU:CD2	2.39	0.52
1:C:16:LEU:HD13	1:C:38:ALA:CB	2.39	0.52
1:D:109:ILE:HG12	5:D:2041:HOH:O	2.09	0.52
1:D:411:HIS:HB3	5:D:2114:HOH:O	2.08	0.52
1:D:623:TYR:CD1	1:D:663:LYS:HE2	2.43	0.52
1:D:85:ILE:HG22	1:D:89:PHE:CE1	2.45	0.52
2:E:4:DA:H2"	2:E:5:DA:N7	2.24	0.52
1:A:428:ALA:N	1:A:435:GLN:NE2	2.48	0.52
1:A:632:ARG:HH11	1:A:632:ARG:CB	2.20	0.52
1:B:168:GLU:OE1	1:B:169:GLN:N	2.33	0.52
1:B:551:ARG:N	5:B:2240:HOH:O	2.41	0.52
1:B:116:TYR:HE2	1:B:752:LEU:HD22	1.74	0.52
1:C:424:GLY:HA3	5:C:2102:HOH:O	2.09	0.52

Continued on next page...

Continued from previous page...

Atom-1	Atom-2	Interatomic distance (Å)	Clash overlap (Å)
1:D:16:LEU:HD13	1:D:38:ALA:CB	2.38	0.52
1:D:353:PRO:HD3	5:D:2098:HOH:O	2.10	0.52
1:D:574:VAL:O	1:D:578:VAL:HG23	2.09	0.52
1:D:588:ASN:N	1:D:588:ASN:HD22	2.06	0.52
1:D:710:VAL:HG21	1:D:854:HIS:CD2	2.44	0.52
1:D:92:VAL:HG21	5:D:2029:HOH:O	2.09	0.52
1:A:106:LEU:HD21	5:A:2094:HOH:O	2.09	0.52
1:A:312:TYR:HB3	5:A:2131:HOH:O	2.10	0.52
1:A:372:GLU:N	5:A:2145:HOH:O	2.41	0.52
1:B:74:ILE:HG22	1:B:755:PHE:HE2	1.74	0.52
1:C:556:GLY:O	1:C:561:LEU:HB2	2.09	0.52
1:B:63:GLU:OE1	2:H:18:DC:H2”	2.09	0.52
1:A:122:THR:HG22	1:A:126:LEU:CD1	2.35	0.52
1:A:205:HIS:C	1:A:207:GLU:H	2.13	0.52
1:A:347:CYS:CB	1:A:350:GLU:CG	2.87	0.52
1:B:881:ALA:O	1:B:883:ALA:N	2.42	0.52
1:B:9:ASN:HA	1:B:12:SER:HB3	1.91	0.52
1:C:756:ARG:HG2	5:C:2161:HOH:O	2.09	0.52
1:D:21:PHE:C	1:D:23:THR:H	2.12	0.52
1:D:278:TRP:CE3	1:D:284:GLY:HA3	2.45	0.52
1:A:555:GLY:O	1:A:559:VAL:HG22	2.08	0.52
1:B:595:VAL:HG12	1:B:596:THR:N	2.25	0.52
1:C:792:ARG:O	1:C:796:VAL:HG23	2.10	0.52
1:D:806:SER:O	1:D:816:THR:CG2	2.51	0.52
1:A:146:GLU:HG3	1:A:204:TRP:CE3	2.44	0.52
1:A:21:PHE:C	1:A:23:THR:N	2.63	0.52
1:A:629:VAL:HG11	1:A:677:MET:HE3	1.92	0.52
1:A:668:THR:C	1:A:669:GLN:HE21	2.12	0.52
1:A:677:MET:O	1:A:681:ILE:HG13	2.10	0.52
1:A:743:ILE:HG22	1:A:743:ILE:O	2.10	0.52
1:B:557:ARG:NH1	5:B:2244:HOH:O	2.43	0.52
1:C:402:LEU:HG	1:C:439:MET:HE2	1.91	0.52
1:C:752:LEU:N	1:C:752:LEU:HD12	2.25	0.52
1:D:177:VAL:HG12	1:D:178:TYR:HD2	1.75	0.52
1:D:15:GLU:HB2	1:D:18:ALA:O	2.09	0.52
1:D:264:ILE:HB	5:D:2068:HOH:O	2.10	0.52
1:D:36:GLN:HG2	5:D:2021:HOH:O	2.10	0.52
1:D:545:HIS:O	1:D:549:MET:HG2	2.10	0.52
1:D:59:LEU:CD2	1:D:64:VAL:HG22	2.39	0.52
1:A:163:LYS:HB3	1:A:164:LYS:HZ3	1.73	0.52
1:A:243:THR:O	1:A:244:ILE:HD13	2.09	0.52

Continued on next page...

Continued from previous page...

Atom-1	Atom-2	Interatomic distance (Å)	Clash overlap (Å)
1:B:203:SER:O	1:B:205:HIS:N	2.43	0.52
1:B:401:MET:HE1	1:B:432:PHE:HB2	1.91	0.52
1:C:814:PHE:N	1:C:814:PHE:CD1	2.78	0.52
1:D:651:LEU:HD13	1:D:651:LEU:O	2.10	0.52
1:D:81:MET:HE1	1:D:220:LEU:HB2	1.91	0.52
2:E:2:DG:H2"	2:E:3:DG:C8	2.42	0.52
2:N:11:DA:H2"	2:N:12:DT:O5'	2.10	0.52
1:A:692:ALA:O	1:A:696:MET:HG3	2.09	0.52
1:B:146:GLU:HG3	1:B:204:TRP:CE3	2.45	0.52
1:B:232:GLN:HG2	1:B:241:SER:O	2.10	0.52
1:B:812:ASP:OD1	3:I:8:U:O3'	2.26	0.52
1:D:347:CYS:O	1:D:349:VAL:N	2.43	0.52
1:A:50:ARG:HG2	1:A:50:ARG:NH1	2.24	0.52
1:A:551:ARG:CZ	1:A:872:LEU:HD11	2.40	0.52
1:B:158:GLU:CA	1:B:195:LEU:HD22	2.37	0.52
1:B:422:TRP:CD1	1:B:422:TRP:C	2.83	0.52
1:B:754:GLN:HE22	3:I:1:G:H4'	1.75	0.52
1:B:99:ARG:HB3	1:B:103:PHE:HD2	1.74	0.52
1:D:215:ARG:CG	5:D:2030:HOH:O	2.58	0.52
1:D:743:ILE:HD12	1:D:766:ASP:HB2	1.92	0.52
1:A:777:GLY:O	1:A:781:ASN:HB2	2.11	0.51
1:B:14:ILE:HG22	1:B:14:ILE:O	2.11	0.51
1:B:185:VAL:HG23	1:B:186:VAL:N	2.24	0.51
1:B:374:LEU:H	1:B:374:LEU:CD1	2.23	0.51
1:C:19:ILE:HG23	1:C:20:PRO:HD2	1.91	0.51
1:D:422:TRP:O	1:D:422:TRP:CD1	2.63	0.51
1:B:341:ILE:HG13	1:B:348:PRO:HB3	1.91	0.51
1:C:21:PHE:C	1:C:23:THR:N	2.61	0.51
1:D:154:ILE:CG2	1:D:190:MET:HE1	2.38	0.51
1:D:882:PHE:O	1:D:883:ALA:CB	2.58	0.51
2:H:6:DT:H2"	2:H:7:DC:C5	2.44	0.51
1:A:105:PHE:HA	5:A:2067:HOH:O	2.11	0.51
1:A:706:LEU:HD21	1:A:849:PHE:HB2	1.91	0.51
1:B:398:LEU:C	1:B:398:LEU:CD2	2.78	0.51
1:C:329:LYS:HG3	1:C:445:THR:HG23	1.92	0.51
1:C:572:GLY:O	1:C:576:LYS:HG3	2.11	0.51
1:C:425:ARG:HH21	1:C:784:HIS:CG	2.28	0.51
1:D:318:LYS:O	1:D:322:ILE:HG12	2.11	0.51
1:D:560:ASN:OD1	1:D:568:GLN:HB2	2.09	0.51
1:D:623:TYR:HD1	1:D:666:MET:HE1	1.75	0.51
1:D:721:LYS:HG2	1:D:722:ARG:N	2.24	0.51

Continued on next page...

Continued from previous page...

Atom-1	Atom-2	Interatomic distance (Å)	Clash overlap (Å)
1:A:65:ALA:CB	1:A:120:LYS:HG2	2.39	0.51
1:A:143:ARG:NH1	5:A:2073:HOH:O	2.42	0.51
1:A:228:SER:HA	5:A:2100:HOH:O	2.09	0.51
1:A:824:LEU:O	1:A:828:VAL:HG23	2.09	0.51
1:C:546:PHE:CE1	1:C:783:VAL:HG13	2.46	0.51
1:D:89:PHE:CD2	1:D:103:PHE:HE1	2.28	0.51
1:D:199:GLU:HG3	1:D:201:TRP:HD1	1.75	0.51
1:A:183:MET:HB2	5:A:2084:HOH:O	2.10	0.51
1:A:386:ARG:HG3	3:F:4:G:H5”	1.93	0.51
1:A:517:GLU:HG3	1:A:532:LEU:HB2	1.92	0.51
1:B:253:ALA:HB1	5:B:2001:HOH:O	2.10	0.51
1:B:747:LEU:HD13	5:B:2316:HOH:O	2.10	0.51
1:D:556:GLY:O	1:D:561:LEU:HB2	2.10	0.51
1:D:655:ILE:HD12	1:D:674:ALA:HB2	1.92	0.51
1:A:142:GLY:N	5:A:2072:HOH:O	2.43	0.51
1:A:47:GLY:HA3	1:A:265:SER:O	2.10	0.51
1:B:227:VAL:HB	1:B:244:ILE:CG2	2.41	0.51
1:B:308:TYR:HA	1:B:311:VAL:CG2	2.41	0.51
1:C:308:TYR:HA	1:C:311:VAL:HG23	1.93	0.51
1:C:373:ALA:C	1:C:377:TRP:NE1	2.64	0.51
1:C:398:LEU:HD23	1:C:398:LEU:C	2.31	0.51
1:C:480:LYS:O	1:C:484:GLU:HG3	2.11	0.51
1:C:743:ILE:HD12	1:C:766:ASP:HB3	1.92	0.51
1:D:19:ILE:HG23	1:D:20:PRO:CD	2.39	0.51
1:D:829:ARG:HD3	1:D:875:ILE:CG2	2.40	0.51
1:A:704:LYS:HZ2	1:A:860:LYS:HD2	1.75	0.51
1:B:53:LYS:O	1:B:56:GLU:HB3	2.11	0.51
1:B:92:VAL:HG12	1:B:99:ARG:HG3	1.93	0.51
1:C:25:ALA:HA	1:C:29:GLY:O	2.11	0.51
1:C:275:PRO:HB2	1:C:324:GLN:HG2	1.93	0.51
1:C:828:VAL:O	1:C:831:THR:HG22	2.11	0.51
1:D:153:ARG:NH2	1:D:201:TRP:NE1	2.52	0.51
1:D:2:ASN:HB2	5:D:2002:HOH:O	2.10	0.51
1:D:871:ASN:HD21	1:D:873:ARG:HB2	1.75	0.51
1:D:814:PHE:CE1	1:D:883:ALA:CB	2.92	0.51
2:K:6:DT:H2”	2:K:7:DC:OP2	2.10	0.51
1:A:860:LYS:O	1:A:862:PRO:HD3	2.10	0.51
1:B:178:TYR:CD1	1:B:178:TYR:N	2.77	0.51
1:C:120:LYS:CD	1:C:752:LEU:HD11	2.41	0.51
1:C:473:VAL:CG1	1:C:477:GLU:HB2	2.41	0.51
1:C:730:PRO:HD2	1:C:786:GLN:NE2	2.25	0.51

Continued on next page...

Continued from previous page...

Atom-1	Atom-2	Interatomic distance (Å)	Clash overlap (Å)
1:D:25:ALA:HA	1:D:29:GLY:O	2.11	0.51
1:D:275:PRO:HG2	1:D:324:GLN:HG2	1.92	0.51
1:D:454:LYS:HG3	1:D:455:GLU:N	2.25	0.51
1:D:78:LEU:N	1:D:79:PRO:HD2	2.25	0.51
1:A:208:ASP:HA	5:A:2093:HOH:O	2.10	0.51
1:B:726:HIS:HD2	1:B:736:TRP:CE2	2.28	0.51
1:C:154:ILE:HD13	1:C:190:MET:HE1	1.92	0.51
1:C:191:LEU:HD23	1:C:195:LEU:O	2.11	0.51
1:C:744:GLN:HB3	1:C:756:ARG:HB3	1.92	0.51
1:D:47:GLY:HA3	1:D:265:SER:O	2.11	0.51
1:D:272:VAL:O	1:D:272:VAL:HG12	2.11	0.51
1:D:633:SER:CA	1:D:649:GLN:HE22	2.14	0.51
1:D:882:PHE:N	1:D:882:PHE:CD1	2.71	0.51
4:M:4:DG:H2"	4:M:5:DA:C8	2.46	0.51
1:A:720:ARG:HD2	1:A:852:GLN:O	2.10	0.51
1:A:861:MET:HE3	1:A:862:PRO:HD2	1.93	0.51
1:B:648:GLN:O	1:B:652:GLU:HG2	2.11	0.51
1:C:199:GLU:O	1:C:199:GLU:HG2	2.11	0.51
1:C:205:HIS:C	1:C:207:GLU:H	2.14	0.51
1:C:59:LEU:HA	1:C:64:VAL:CG2	2.41	0.51
1:C:751:PHE:HB3	1:C:752:LEU:CD1	2.39	0.51
1:D:242:GLU:HG3	5:D:2060:HOH:O	2.11	0.51
1:D:281:ILE:CG2	1:D:282:THR:HG23	2.30	0.51
1:B:423:ARG:NH2	2:H:12:DT:O4'	2.44	0.51
1:A:230:HIS:CD2	1:A:232:GLN:HE22	2.29	0.50
1:A:457:TYR:CE2	1:A:461:LYS:HD3	2.45	0.50
1:B:540:CYS:HB3	5:B:2238:HOH:O	2.11	0.50
1:B:600:GLU:HG3	5:B:2268:HOH:O	2.11	0.50
1:C:317:TYR:O	1:C:321:ASN:ND2	2.42	0.50
1:C:608:LYS:HG3	5:C:2141:HOH:O	2.12	0.50
1:D:335:LEU:HD12	5:D:2090:HOH:O	2.11	0.50
1:D:791:LEU:HG	1:D:809:LEU:HD22	1.93	0.50
1:A:115:ALA:O	1:A:119:ILE:CD1	2.59	0.50
1:B:25:ALA:HA	1:B:29:GLY:O	2.11	0.50
1:B:562:LEU:N	1:B:562:LEU:HD23	2.26	0.50
1:B:597:VAL:HG22	5:B:2264:HOH:O	2.12	0.50
1:B:737:GLN:HE21	1:B:739:TYR:HE2	1.57	0.50
1:B:729:THR:HB	1:B:789:SER:HB2	1.93	0.50
1:B:797:TRP:CZ2	1:B:801:LYS:HG3	2.46	0.50
1:C:373:ALA:HB3	1:C:374:LEU:HD12	1.93	0.50
1:C:422:TRP:CE3	5:C:2101:HOH:O	2.53	0.50

Continued on next page...

Continued from previous page...

Atom-1	Atom-2	Interatomic distance (Å)	Clash overlap (Å)
1:D:165:ASN:O	1:D:169:GLN:NE2	2.45	0.50
1:D:210:ILE:O	1:D:214:VAL:HG23	2.11	0.50
1:D:84:ARG:HB2	1:D:223:SER:HB3	1.94	0.50
1:D:546:PHE:CE1	1:D:783:VAL:HG13	2.46	0.50
1:D:553:GLU:HG2	1:D:554:VAL:N	2.26	0.50
1:A:160:LYS:NZ	1:A:161:HIS:NE2	2.58	0.50
1:A:272:VAL:O	1:A:272:VAL:HG12	2.11	0.50
1:B:47:GLY:HA3	1:B:265:SER:O	2.12	0.50
1:B:93:LYS:HB2	5:B:2076:HOH:O	2.11	0.50
1:C:386:ARG:HD3	3:L:4:G:H5”	1.94	0.50
1:C:57:ARG:HD3	5:K:2015:HOH:O	2.11	0.50
1:C:663:LYS:HG2	1:C:664:GLY:N	2.24	0.50
1:D:720:ARG:HG2	1:D:720:ARG:NH1	2.26	0.50
1:D:778:ILE:CG2	1:D:779:ALA:N	2.74	0.50
1:D:85:ILE:HG22	1:D:89:PHE:CD1	2.46	0.50
1:D:871:ASN:ND2	1:D:873:ARG:HB2	2.26	0.50
2:E:10:DC:H2”	2:E:11:DA:OP2	2.12	0.50
1:D:394:ARG:HD3	3:O:5:C:O3’	2.11	0.50
1:A:236:VAL:HG11	1:A:239:GLN:HG3	1.92	0.50
1:A:713:LYS:C	1:A:714:LYS:HG2	2.32	0.50
1:C:11:PHE:CE1	1:C:44:TYR:HB3	2.47	0.50
1:D:511:PHE:CE2	1:D:515:CYS:SG	3.04	0.50
1:D:659:ILE:HG12	1:D:664:GLY:HA3	1.94	0.50
2:H:9:DA:H5”	5:H:2011:HOH:O	2.10	0.50
1:B:744:GLN:HA	1:B:756:ARG:NH2	2.25	0.50
1:C:269:GLN:HE22	1:C:407:LYS:NZ	2.09	0.50
1:D:814:PHE:HE1	1:D:883:ALA:HB1	1.77	0.50
1:A:21:PHE:CD1	1:A:21:PHE:O	2.64	0.50
1:A:281:ILE:CG2	1:A:282:THR:HG23	2.39	0.50
1:A:48:GLU:HB2	5:A:2112:HOH:O	2.11	0.50
1:A:50:ARG:HH11	1:A:50:ARG:CG	2.24	0.50
1:B:714:LYS:HZ2	1:B:714:LYS:HA	1.76	0.50
1:C:133:THR:HA	1:C:243:THR:CG2	2.20	0.50
1:C:162:PHE:CD2	1:C:162:PHE:O	2.65	0.50
1:C:495:SER:HB3	5:C:2118:HOH:O	2.12	0.50
1:C:846:TYR:CE1	1:C:850:ALA:HB2	2.47	0.50
1:D:551:ARG:NH1	1:D:872:LEU:HD12	2.27	0.50
1:B:19:ILE:CD1	1:B:20:PRO:HD2	2.42	0.50
1:B:322:ILE:CD1	1:B:799:HIS:CG	2.95	0.50
1:B:418:TYR:C	1:B:419:ASN:HD22	2.15	0.50
1:B:60:LYS:O	1:B:60:LYS:HG2	2.12	0.50

Continued on next page...

Continued from previous page...

Atom-1	Atom-2	Interatomic distance (Å)	Clash overlap (Å)
1:B:748:ASN:HD22	1:B:752:LEU:H	1.59	0.50
1:B:854:HIS:CG	1:B:855:GLU:N	2.80	0.50
1:C:809:LEU:O	1:C:810:ILE:HG13	2.12	0.50
1:D:704:LYS:HE3	5:D:2175:HOH:O	2.12	0.50
1:A:313:MET:HB2	1:A:316:VAL:HG23	1.94	0.50
1:B:828:VAL:HB	1:B:883:ALA:HA	1.94	0.50
1:C:568:GLN:HB3	5:C:2130:HOH:O	2.11	0.50
1:C:729:THR:HB	1:C:789:SER:HB2	1.92	0.50
1:D:21:PHE:C	1:D:23:THR:N	2.63	0.50
1:D:414:ILE:HG22	5:D:2115:HOH:O	2.12	0.50
1:D:639:TYR:O	2:N:10:DC:H4’	2.12	0.50
1:B:427:TYR:HA	1:B:435:GLN:HE22	1.76	0.50
1:B:452:ILE:CG2	1:B:453:GLY:N	2.75	0.50
1:B:791:LEU:C	1:B:791:LEU:HD23	2.32	0.50
1:C:164:LYS:CE	1:C:164:LYS:N	2.69	0.50
1:D:422:TRP:CD1	1:D:423:ARG:HG3	2.47	0.50
1:A:176:HIS:ND1	1:A:176:HIS:C	2.66	0.49
1:A:19:ILE:HG23	1:A:20:PRO:HD2	1.94	0.49
1:B:407:LYS:HG2	1:B:408:PHE:CE2	2.47	0.49
1:C:47:GLY:HA3	1:C:265:SER:O	2.12	0.49
1:C:328:TRP:O	1:C:413:ALA:HA	2.12	0.49
1:D:229:LEU:HD12	1:D:243:THR:O	2.13	0.49
1:D:585:ASP:OD2	1:D:613:THR:CB	2.57	0.49
1:D:311:VAL:HG11	1:D:734:PRO:HG3	1.93	0.49
1:A:404:GLN:HG2	1:A:432:PHE:HB2	1.92	0.49
1:A:56:GLU:OE1	1:A:57:ARG:N	2.45	0.49
1:A:59:LEU:HD23	1:A:64:VAL:CG1	2.43	0.49
1:A:861:MET:HE2	1:A:862:PRO:HD2	1.93	0.49
1:C:304:ALA:HB1	5:C:2085:HOH:O	2.12	0.49
1:D:261:LEU:HD12	5:D:2068:HOH:O	2.13	0.49
1:D:180:LYS:HG3	1:D:750:MET:CE	2.43	0.49
1:A:65:ALA:HB3	5:A:2057:HOH:O	2.12	0.49
1:B:215:ARG:HG3	1:B:219:MET:HE2	1.95	0.49
5:A:2233:HOH:O	1:B:683:GLU:HB3	2.12	0.49
1:C:120:LYS:HD2	1:C:752:LEU:HD11	1.94	0.49
1:C:292:ARG:HG3	1:C:292:ARG:O	2.12	0.49
1:C:742:PRO:HD2	5:C:2159:HOH:O	2.11	0.49
1:C:774:GLN:HG2	5:C:2154:HOH:O	2.12	0.49
1:D:576:LYS:O	1:D:580:GLU:HG3	2.11	0.49
1:D:656:GLN:N	1:D:657:PRO:HD2	2.27	0.49
1:D:322:ILE:HD12	1:D:799:HIS:CD2	2.46	0.49

Continued on next page...

Continued from previous page...

Atom-1	Atom-2	Interatomic distance (Å)	Clash overlap (Å)
1:A:158:GLU:HA	1:A:195:LEU:HD22	1.94	0.49
1:A:473:VAL:HG22	1:A:474:PRO:HD2	1.93	0.49
1:A:681:ILE:O	1:A:685:VAL:HG22	2.12	0.49
1:A:116:TYR:CZ	1:A:752:LEU:HD22	2.48	0.49
1:B:164:LYS:HD3	5:B:2092:HOH:O	2.12	0.49
1:B:181:ALA:O	1:B:185:VAL:HG13	2.12	0.49
1:B:19:ILE:HD12	5:B:2046:HOH:O	2.12	0.49
1:B:350:GLU:HG3	5:B:2161:HOH:O	2.12	0.49
1:B:452:ILE:HG23	1:B:453:GLY:H	1.76	0.49
1:B:495:SER:CB	5:B:2218:HOH:O	2.59	0.49
1:B:718:ILE:HG13	5:B:2305:HOH:O	2.12	0.49
1:B:74:ILE:HG13	1:B:119:ILE:HG21	1.95	0.49
1:B:788:GLY:HA2	5:B:2323:HOH:O	2.11	0.49
1:C:101:THR:O	1:C:104:GLN:HG2	2.12	0.49
1:C:203:SER:O	1:C:205:HIS:N	2.46	0.49
1:C:392:LYS:O	1:C:396:ILE:HG12	2.12	0.49
1:C:329:LYS:HG3	1:C:445:THR:CG2	2.43	0.49
1:C:78:LEU:N	1:C:79:PRO:CD	2.75	0.49
1:D:203:SER:O	1:D:205:HIS:N	2.46	0.49
1:A:246:LEU:CD2	1:A:251:ALA:HB2	2.42	0.49
1:A:332:LYS:CE	1:A:410:ASN:ND2	2.76	0.49
1:A:275:PRO:HD3	1:A:415:TRP:HB2	1.94	0.49
1:A:425:ARG:HH21	1:A:784:HIS:HD2	1.57	0.49
1:B:56:GLU:OE1	1:B:56:GLU:C	2.51	0.49
1:C:339:ASN:O	1:C:343:LYS:HD2	2.12	0.49
1:C:420:MET:HA	1:C:425:ARG:O	2.13	0.49
1:C:537:ASP:OD1	1:C:813:SER:HB2	2.13	0.49
1:C:602:THR:CG2	1:C:604:GLU:HB2	2.43	0.49
1:D:616:LEU:HD13	1:D:676:TYR:HB2	1.95	0.49
1:D:706:LEU:HD21	1:D:849:PHE:HB2	1.93	0.49
1:A:309:GLU:HG2	1:A:310:ASP:OD1	2.13	0.49
1:A:391:ARG:CG	1:A:391:ARG:HH11	2.25	0.49
1:A:44:TYR:OH	1:A:292:ARG:HB3	2.12	0.49
1:A:828:VAL:O	1:A:831:THR:HG22	2.12	0.49
1:B:401:MET:CE	1:B:432:PHE:HA	2.42	0.49
1:B:84:ARG:CA	1:B:84:ARG:HH11	2.26	0.49
1:C:437:ASN:ND2	1:C:440:THR:HB	2.27	0.49
4:P:5:DA:H2”	4:P:6:DT:C7	2.43	0.49
1:A:270:PRO:HD2	1:A:408:PHE:HE2	1.76	0.49
1:A:31:ARG:NH2	5:A:2047:HOH:O	2.45	0.49
1:B:231:ARG:NH1	1:B:242:GLU:HB2	2.28	0.49

Continued on next page...

Continued from previous page...

Atom-1	Atom-2	Interatomic distance (Å)	Clash overlap (Å)
1:B:312:TYR:CE1	1:B:314:PRO:HG3	2.48	0.49
1:B:532:LEU:CD1	1:B:533:PRO:HD2	2.32	0.49
1:B:705:LEU:HD23	1:B:705:LEU:N	2.24	0.49
1:C:16:LEU:HD13	1:C:38:ALA:HA	1.95	0.49
1:C:402:LEU:HG	1:C:439:MET:HE1	1.93	0.49
1:C:870:LEU:CD2	1:C:872:LEU:HD23	2.37	0.49
1:D:9:ASN:HA	1:D:12:SER:CB	2.42	0.49
2:E:15:DC:H2"	2:E:16:DC:O5'	2.12	0.49
4:J:8:DC:H2"	4:J:9:DC:OP2	2.13	0.49
4:M:1:DG:H1'	4:M:2:DT:C7	2.38	0.49
1:A:602:THR:CG2	1:A:604:GLU:HB2	2.42	0.49
1:B:233:ASN:CB	1:B:239:GLN:HB3	2.42	0.49
1:B:322:ILE:HD13	1:B:799:HIS:CD2	2.48	0.49
1:C:352:ILE:C	5:C:2093:HOH:O	2.50	0.49
1:C:452:ILE:HG23	1:C:453:GLY:N	2.28	0.49
1:C:546:PHE:CZ	1:C:783:VAL:HG13	2.48	0.49
1:C:727:TRP:HA	1:C:848:GLN:NE2	2.23	0.49
4:P:5:DA:H2"	4:P:6:DT:H72	1.95	0.49
1:B:141:ILE:O	1:B:145:ILE:HG12	2.13	0.49
1:B:159:ALA:HA	1:B:162:PHE:HB3	1.93	0.49
1:B:278:TRP:HE1	1:B:324:GLN:NE2	2.09	0.49
1:B:331:ASN:C	5:B:2150:HOH:O	2.52	0.49
1:D:155:ARG:CG	1:D:163:LYS:HE2	2.43	0.49
1:A:112:GLU:N	1:A:112:GLU:CD	2.64	0.49
1:A:553:GLU:CD	1:A:553:GLU:H	2.16	0.49
1:B:15:GLU:HB2	1:B:19:ILE:HG12	1.95	0.49
1:B:230:HIS:HB2	5:B:2109:HOH:O	2.11	0.49
1:B:347:CYS:SG	1:B:350:GLU:HG2	2.53	0.49
1:B:51:PHE:HE1	1:B:261:LEU:HB3	1.78	0.49
1:C:463:HIS:CD2	5:C:2111:HOH:O	2.66	0.49
1:C:446:LEU:HB2	1:C:531:SER:O	2.12	0.49
1:D:422:TRP:O	1:D:422:TRP:HD1	1.95	0.49
1:D:99:ARG:HD3	1:D:103:PHE:CE2	2.46	0.49
2:N:16:DC:H2"	2:N:17:DG:O5'	2.12	0.49
1:A:205:HIS:C	1:A:207:GLU:N	2.64	0.48
1:A:230:HIS:HE2	1:A:245:GLU:CD	2.16	0.48
1:A:85:ILE:HG13	5:A:2060:HOH:O	2.13	0.48
1:B:159:ALA:O	1:B:160:LYS:C	2.52	0.48
1:B:423:ARG:HE	1:B:781:ASN:ND2	2.11	0.48
1:C:778:ILE:CG2	1:C:779:ALA:N	2.76	0.48
1:D:337:VAL:HG12	1:D:341:ILE:HD11	1.94	0.48

Continued on next page...

Continued from previous page...

Atom-1	Atom-2	Interatomic distance (Å)	Clash overlap (Å)
1:D:418:TYR:C	1:D:419:ASN:HD22	2.16	0.48
1:D:82:ILE:HG21	1:D:112:GLU:OE2	2.13	0.48
1:D:854:HIS:CG	1:D:855:GLU:N	2.81	0.48
1:A:118:THR:HG23	1:A:141:ILE:HD13	1.92	0.48
1:A:243:THR:C	1:A:244:ILE:HD13	2.34	0.48
1:A:229:LEU:HD12	1:A:243:THR:O	2.13	0.48
1:A:275:PRO:HB2	1:A:324:GLN:HG2	1.95	0.48
1:B:78:LEU:N	1:B:79:PRO:HD2	2.27	0.48
1:C:42:GLU:O	1:C:46:MET:HG3	2.13	0.48
1:C:582:LEU:HD11	1:C:625:VAL:HG21	1.95	0.48
1:D:475:PHE:CD1	1:D:475:PHE:N	2.81	0.48
2:H:17:DG:H2”	2:H:18:DC:C5	2.49	0.48
1:A:571:TYR:CE1	1:A:634:VAL:CG1	2.97	0.48
1:A:842:LEU:HD23	1:A:864:LEU:HD23	1.94	0.48
1:B:206:LYS:HE2	1:B:207:GLU:OE2	2.13	0.48
1:B:347:CYS:SG	1:B:350:GLU:CG	3.02	0.48
1:B:401:MET:CE	1:B:432:PHE:CD1	2.94	0.48
1:B:437:ASN:ND2	1:B:440:THR:H	2.11	0.48
1:B:60:LYS:HD3	5:B:2062:HOH:O	2.13	0.48
1:C:19:ILE:HD12	1:C:20:PRO:HD2	1.94	0.48
1:C:205:HIS:CG	1:C:207:GLU:HG2	2.48	0.48
1:C:59:LEU:HD23	1:C:64:VAL:CG2	2.43	0.48
1:C:633:SER:HB3	1:C:646:PHE:CD1	2.47	0.48
1:D:475:PHE:HE1	1:D:880:PHE:HD1	1.60	0.48
1:A:6:ILE:HG22	1:A:10:ASP:HB3	1.95	0.48
1:A:242:GLU:N	5:A:2106:HOH:O	2.46	0.48
1:B:437:ASN:C	1:B:437:ASN:ND2	2.67	0.48
1:B:78:LEU:HD21	1:B:116:TYR:CD1	2.48	0.48
1:C:609:VAL:O	1:C:672:GLN:NE2	2.46	0.48
1:C:711:LYS:HZ2	1:C:711:LYS:HB2	1.77	0.48
1:D:595:VAL:HG12	1:D:596:THR:N	2.27	0.48
1:D:828:VAL:HB	1:D:883:ALA:HA	1.95	0.48
1:A:170:LEU:HD23	1:A:170:LEU:C	2.33	0.48
1:A:710:VAL:CG1	1:A:720:ARG:HB3	2.42	0.48
1:B:170:LEU:HD22	1:B:179:LYS:HD3	1.96	0.48
1:B:41:HIS:NE2	1:B:45:GLU:OE2	2.46	0.48
1:B:490:MET:HE1	1:B:522:GLN:HB2	1.94	0.48
1:C:789:SER:O	1:C:793:LYS:HG3	2.14	0.48
1:C:84:ARG:HH21	1:C:222:GLU:CD	2.16	0.48
1:D:269:GLN:NE2	1:D:404:GLN:OE1	2.40	0.48
1:D:727:TRP:HB3	1:D:845:PHE:CD1	2.48	0.48

Continued on next page...

Continued from previous page...

Atom-1	Atom-2	Interatomic distance (Å)	Clash overlap (Å)
1:D:868:GLY:C	1:D:869:ASN:HD22	2.16	0.48
2:N:4:DA:N3	5:N:2006:HOH:O	2.35	0.48
1:A:292:ARG:NH1	1:A:292:ARG:HG2	2.26	0.48
1:A:315:GLU:OE2	1:A:318:LYS:CD	2.58	0.48
1:B:230:HIS:CE1	1:B:232:GLN:HE22	2.32	0.48
1:B:401:MET:HE2	1:B:432:PHE:CD1	2.48	0.48
1:B:463:HIS:HD2	1:B:535:ALA:H	1.62	0.48
1:C:229:LEU:CD1	1:C:244:ILE:HD13	2.44	0.48
1:C:28:TYR:CE2	1:C:274:PRO:HD2	2.47	0.48
1:C:9:ASN:HA	1:C:12:SER:HB3	1.95	0.48
1:D:150:ARG:HG3	1:D:201:TRP:CD1	2.49	0.48
1:D:423:ARG:HE	1:D:781:ASN:ND2	2.11	0.48
1:A:164:LYS:NZ	1:A:164:LYS:N	2.62	0.48
1:A:374:LEU:C	1:A:376:ALA:H	2.15	0.48
1:A:457:TYR:CZ	1:A:461:LYS:HD3	2.48	0.48
1:A:854:HIS:CG	1:A:855:GLU:N	2.82	0.48
1:B:404:GLN:HG2	1:B:432:PHE:CB	2.43	0.48
1:B:698:TRP:CZ2	1:B:864:LEU:CD2	2.97	0.48
1:C:551:ARG:NE	1:C:872:LEU:HD21	2.28	0.48
1:D:27:HIS:HB3	1:D:28:TYR:CD1	2.48	0.48
1:D:537:ASP:N	1:D:882:PHE:HD2	2.11	0.48
1:A:563:PRO:HB3	1:A:877:GLU:O	2.14	0.48
1:A:695:ALA:O	1:A:699:LEU:HG	2.14	0.48
1:B:84:ARG:HG3	1:B:223:SER:HB3	1.95	0.48
1:C:5:ASN:C	1:C:5:ASN:ND2	2.67	0.48
1:C:816:THR:HG22	1:C:817:ILE:H	1.79	0.48
1:C:84:ARG:C	1:C:84:ARG:HD3	2.33	0.48
4:M:5:DA:H2"	4:M:6:DT:C7	2.43	0.48
2:N:15:DC:H2"	2:N:16:DC:O5'	2.14	0.48
1:A:281:ILE:HD11	1:A:308:TYR:C	2.34	0.48
1:A:315:GLU:HB3	5:A:2132:HOH:O	2.13	0.48
1:A:324:GLN:NE2	1:A:418:TYR:H	2.12	0.48
1:B:226:MET:HA	1:B:250:TYR:CD1	2.49	0.48
1:B:401:MET:CE	1:B:432:PHE:CB	2.91	0.48
1:B:829:ARG:NH1	1:B:829:ARG:CG	2.68	0.48
1:C:162:PHE:CB	5:C:2059:HOH:O	2.59	0.48
1:C:316:VAL:CG1	1:C:420:MET:HE1	2.44	0.48
1:C:329:LYS:HD2	1:C:447:ALA:HA	1.94	0.48
1:C:619:GLN:O	1:C:622:ALA:HB3	2.14	0.48
1:C:789:SER:HA	1:C:792:ARG:NH2	2.29	0.48
1:D:42:GLU:HG2	1:D:46:MET:CE	2.44	0.48

Continued on next page...

Continued from previous page...

Atom-1	Atom-2	Interatomic distance (Å)	Clash overlap (Å)
2:K:17:DG:H2"	2:K:18:DC:C5	2.48	0.48
2:K:16:DC:H2"	2:K:17:DG:O5'	2.14	0.48
2:K:6:DT:H5"	5:K:2009:HOH:O	2.14	0.48
4:M:6:DT:C2'	4:M:7:DT:H72	2.44	0.48
1:A:82:ILE:O	1:A:86:ASN:ND2	2.47	0.48
1:C:194:GLY:O	1:C:196:LEU:HD23	2.14	0.48
1:C:706:LEU:HD11	1:C:849:PHE:CD2	2.49	0.48
1:D:315:GLU:OE2	1:D:315:GLU:CA	2.50	0.48
1:D:829:ARG:HH11	1:D:829:ARG:CG	2.21	0.48
2:N:2:DG:H2"	2:N:3:DG:C8	2.49	0.48
2:N:4:DA:H2"	2:N:5:DA:OP2	2.14	0.48
1:A:78:LEU:N	1:A:79:PRO:CD	2.77	0.47
1:B:291:ARG:C	1:B:293:PRO:HD3	2.34	0.47
1:B:305:LEU:O	1:B:305:LEU:HD23	2.13	0.47
1:C:278:TRP:N	1:C:321:ASN:OD1	2.45	0.47
1:C:468:ALA:HB2	1:C:511:PHE:HE1	1.74	0.47
1:C:475:PHE:N	5:C:2112:HOH:O	2.28	0.47
1:C:56:GLU:OE1	1:C:57:ARG:CA	2.62	0.47
1:D:111:PRO:HG2	1:D:112:GLU:OE1	2.14	0.47
1:D:19:ILE:O	1:D:21:PHE:N	2.39	0.47
1:D:53:LYS:O	1:D:56:GLU:HB3	2.13	0.47
2:K:8:DG:OP2	5:K:2011:HOH:O	2.20	0.47
1:A:11:PHE:N	5:A:2034:HOH:O	2.30	0.47
1:A:155:ARG:HH11	1:A:155:ARG:CB	2.18	0.47
1:A:425:ARG:CZ	1:A:784:HIS:CD2	2.97	0.47
1:B:151:PHE:HD1	1:B:183:MET:HB3	1.78	0.47
1:B:218:GLU:O	1:B:222:GLU:HB2	2.14	0.47
1:C:303:LYS:NZ	1:C:740:LYS:HZ1	2.11	0.47
1:C:36:GLN:HG2	1:C:273:VAL:HG22	1.95	0.47
1:C:463:HIS:ND1	1:C:532:LEU:HD21	2.29	0.47
1:C:797:TRP:CZ2	1:C:801:LYS:HG3	2.49	0.47
1:D:158:GLU:CD	1:D:195:LEU:HB3	2.35	0.47
1:D:19:ILE:CD1	1:D:20:PRO:HD2	2.43	0.47
2:H:16:DC:H2"	2:H:17:DG:O5'	2.14	0.47
1:A:318:LYS:O	1:A:322:ILE:HG13	2.14	0.47
1:B:577:LYS:HD2	5:B:2301:HOH:O	2.13	0.47
1:B:711:LYS:CA	1:B:719:LEU:HD13	2.39	0.47
1:C:19:ILE:HG23	1:C:20:PRO:CD	2.44	0.47
1:D:28:TYR:CE2	1:D:274:PRO:HD2	2.49	0.47
1:D:352:ILE:HG12	5:D:2098:HOH:O	2.14	0.47
1:D:693:VAL:O	1:D:697:ASN:ND2	2.34	0.47

Continued on next page...

Continued from previous page...

Atom-1	Atom-2	Interatomic distance (Å)	Clash overlap (Å)
2:K:6:DT:C2'	2:K:7:DC:C5	2.96	0.47
1:A:120:LYS:HD2	1:A:752:LEU:HD11	1.96	0.47
1:A:221:ILE:O	5:A:2096:HOH:O	2.20	0.47
1:B:236:VAL:CG2	1:B:239:GLN:HE21	2.16	0.47
1:B:324:GLN:HE21	1:B:417:PRO:CA	2.27	0.47
1:C:105:PHE:HE2	5:C:2015:HOH:O	1.98	0.47
1:C:163:LYS:C	1:C:164:LYS:HE2	2.34	0.47
1:C:488:ASN:HB3	1:C:501:TRP:CE3	2.50	0.47
1:D:179:LYS:HB2	5:D:2052:HOH:O	2.15	0.47
1:D:2:ASN:N	5:D:2001:HOH:O	2.47	0.47
2:H:5:DA:H1'	2:H:6:DT:H5'	1.96	0.47
1:A:6:ILE:HD11	1:A:259:GLY:O	2.14	0.47
1:A:51:PHE:HD2	1:A:262:ALA:HB2	1.79	0.47
1:A:707:ALA:HB2	1:A:774:GLN:HG2	1.97	0.47
1:B:215:ARG:HG3	1:B:219:MET:HE1	1.97	0.47
1:C:134:VAL:HG22	1:C:134:VAL:O	2.15	0.47
1:D:550:LEU:HD23	1:D:550:LEU:HA	1.62	0.47
1:A:120:LYS:HE2	5:A:2057:HOH:O	2.14	0.47
1:A:532:LEU:HD12	1:A:533:PRO:HD2	1.97	0.47
1:A:556:GLY:O	1:A:561:LEU:HB2	2.14	0.47
1:A:720:ARG:CG	1:A:720:ARG:NH1	2.69	0.47
1:B:122:THR:HG22	1:B:126:LEU:HD11	1.97	0.47
1:B:51:PHE:CE1	1:B:261:LEU:HB3	2.50	0.47
1:B:804:ILE:HG22	1:B:807:PHE:CZ	2.49	0.47
1:B:720:ARG:NH2	1:B:857:GLN:OE1	2.43	0.47
1:C:146:GLU:HG3	1:C:204:TRP:CD2	2.49	0.47
1:C:796:VAL:O	1:C:800:GLU:HG3	2.13	0.47
1:D:229:LEU:HD11	1:D:242:GLU:HG2	1.96	0.47
1:D:354:ALA:HB2	5:D:2100:HOH:O	2.14	0.47
1:D:651:LEU:HD13	1:D:651:LEU:C	2.35	0.47
1:C:57:ARG:HH12	2:K:17:DG:H3'	1.80	0.47
2:N:17:DG:H2"	2:N:18:DC:C5	2.50	0.47
1:A:32:LEU:HD12	1:A:272:VAL:HG11	1.94	0.47
1:B:39:LEU:HA	5:B:2052:HOH:O	2.15	0.47
1:C:278:TRP:CD2	1:C:284:GLY:HA3	2.49	0.47
1:C:349:VAL:HG12	1:C:349:VAL:O	2.15	0.47
1:C:686:SER:HA	1:C:693:VAL:HG21	1.97	0.47
1:D:666:MET:SD	1:D:666:MET:N	2.86	0.47
1:D:829:ARG:HD3	1:D:875:ILE:HG22	1.96	0.47
1:D:861:MET:CE	1:D:862:PRO:HD2	2.45	0.47
2:K:6:DT:H2'	2:K:6:DT:H6	1.57	0.47

Continued on next page...

Continued from previous page...

Atom-1	Atom-2	Interatomic distance (Å)	Clash overlap (Å)
1:A:180:LYS:HA	5:A:2084:HOH:O	2.15	0.47
1:A:745:THR:N	1:A:756:ARG:HD3	2.27	0.47
1:A:705:LEU:CD2	1:A:857:GLN:HB2	2.39	0.47
1:B:810:ILE:O	1:B:811:HIS:HB2	2.14	0.47
1:C:72:PRO:HG3	1:C:257:ARG:HG3	1.97	0.47
1:C:427:TYR:HA	1:C:435:GLN:HE22	1.79	0.47
1:D:324:GLN:HG3	1:D:417:PRO:HA	1.96	0.47
1:D:333:LYS:HD2	1:D:516:PHE:CD1	2.50	0.47
1:D:84:ARG:HH11	1:D:84:ARG:HA	1.80	0.47
1:A:231:ARG:HG2	1:A:234:ALA:HB2	1.95	0.47
1:A:324:GLN:HE21	1:A:417:PRO:CA	2.25	0.47
1:B:327:ALA:HB2	5:B:2195:HOH:O	2.13	0.47
1:B:698:TRP:CH2	1:B:864:LEU:HD21	2.50	0.47
1:B:811:HIS:HD2	5:B:2185:HOH:O	1.97	0.47
1:C:748:ASN:HD22	1:C:751:PHE:H	1.61	0.47
1:D:134:VAL:HG22	1:D:134:VAL:O	2.14	0.47
1:D:726:HIS:HD2	1:D:727:TRP:H	1.63	0.47
1:A:292:ARG:CG	1:A:292:ARG:HH11	2.26	0.47
1:B:125:CYS:O	1:B:128:SER:CB	2.63	0.47
1:B:458:TYR:CD1	1:B:479:ILE:HD11	2.50	0.47
1:B:642:LYS:O	1:B:643:GLU:C	2.53	0.47
1:B:713:LYS:HG2	5:H:2003:HOH:O	2.15	0.47
1:D:224:THR:HA	5:D:2028:HOH:O	2.15	0.47
4:M:2:DT:H1'	4:M:3:DC:H5'	1.96	0.47
1:A:134:VAL:HG23	1:A:244:ILE:HD11	1.96	0.47
1:A:30:GLU:HG2	1:A:34:ARG:NH2	2.28	0.47
1:A:332:LYS:HE3	1:A:409:ALA:O	2.14	0.47
1:A:576:LYS:O	1:A:580:GLU:HG3	2.14	0.47
1:A:745:THR:HA	5:A:2271:HOH:O	2.15	0.47
1:B:474:PRO:HG2	1:B:477:GLU:OE2	2.15	0.47
1:B:854:HIS:HD2	1:B:856:SER:H	1.55	0.47
1:B:828:VAL:CG1	1:B:883:ALA:HA	2.45	0.47
1:C:463:HIS:HD2	5:C:2111:HOH:O	1.98	0.47
1:D:154:ILE:HD12	1:D:183:MET:SD	2.54	0.47
1:D:335:LEU:CD1	1:D:339:ASN:HD21	2.28	0.47
1:D:517:GLU:HG3	1:D:532:LEU:HB2	1.97	0.47
1:A:829:ARG:NH1	1:A:878:SER:O	2.48	0.46
1:B:164:LYS:CD	5:B:2092:HOH:O	2.62	0.46
1:B:490:MET:CE	1:B:522:GLN:HG3	2.45	0.46
1:C:159:ALA:O	1:C:160:LYS:C	2.52	0.46
1:C:16:LEU:CD1	1:C:38:ALA:HB2	2.44	0.46

Continued on next page...

Continued from previous page...

Atom-1	Atom-2	Interatomic distance (Å)	Clash overlap (Å)
1:D:140:ALA:HA	5:D:2040:HOH:O	2.15	0.46
1:D:268:PHE:CD1	1:D:286:TYR:HE2	2.32	0.46
1:D:353:PRO:HB2	5:D:2101:HOH:O	2.15	0.46
1:D:720:ARG:HH11	1:D:720:ARG:HG2	1.79	0.46
2:E:4:DA:H2"	2:E:5:DA:H8	1.76	0.46
1:A:160:LYS:NZ	5:A:2077:HOH:O	2.47	0.46
1:A:201:TRP:O	1:A:204:TRP:HB2	2.15	0.46
1:A:404:GLN:HG2	1:A:432:PHE:CB	2.45	0.46
1:A:553:GLU:HG2	1:A:554:VAL:N	2.29	0.46
1:A:562:LEU:N	1:A:562:LEU:HD23	2.28	0.46
1:A:859:ASP:OD1	1:A:860:LYS:N	2.48	0.46
1:B:42:GLU:HA	1:B:45:GLU:HG3	1.96	0.46
1:C:412:LYS:N	1:C:412:LYS:HD2	2.29	0.46
1:C:502:TRP:CE2	1:C:512:LEU:HD22	2.50	0.46
1:C:53:LYS:HB3	1:C:53:LYS:HE2	1.66	0.46
1:C:619:GLN:NE2	1:C:666:MET:O	2.46	0.46
1:D:268:PHE:CD1	1:D:286:TYR:CE2	3.04	0.46
1:D:644:PHE:CE1	4:P:1:DG:N2	2.83	0.46
1:D:829:ARG:CD	1:D:875:ILE:O	2.63	0.46
1:D:793:LYS:NZ	1:D:834:ASP:OD2	2.42	0.46
1:D:89:PHE:HE2	1:D:107:GLN:HA	1.80	0.46
1:A:854:HIS:CD2	1:A:856:SER:OG	2.68	0.46
1:B:269:GLN:HB3	1:B:270:PRO:CD	2.46	0.46
1:B:402:LEU:HG	1:B:439:MET:HE1	1.96	0.46
1:B:840:ASP:O	1:B:841:VAL:C	2.54	0.46
1:C:15:GLU:HG3	1:C:18:ALA:H	1.77	0.46
1:C:229:LEU:HD21	1:C:242:GLU:OE2	2.16	0.46
1:C:595:VAL:HG12	1:C:596:THR:N	2.30	0.46
1:C:651:LEU:C	1:C:651:LEU:HD13	2.35	0.46
1:C:711:LYS:HG2	1:C:718:ILE:N	2.30	0.46
1:C:804:ILE:HG12	1:C:820:ASP:HB3	1.97	0.46
1:D:390:ALA:O	1:D:394:ARG:HG3	2.16	0.46
1:D:412:LYS:O	1:D:413:ALA:HB2	2.15	0.46
1:D:700:LYS:HB2	1:D:700:LYS:HZ2	1.80	0.46
1:D:78:LEU:O	1:D:82:ILE:HG13	2.14	0.46
5:A:2080:HOH:O	3:F:3:G:H5'	2.16	0.46
1:A:745:THR:O	1:A:746:ARG:HG3	2.16	0.46
1:B:173:ARG:O	1:B:179:LYS:HE2	2.15	0.46
1:B:433:ASN:HB2	1:B:434:PRO:HD2	1.97	0.46
1:B:619:GLN:O	1:B:622:ALA:HB3	2.16	0.46
1:B:720:ARG:CG	1:B:720:ARG:NH1	2.68	0.46

Continued on next page...

Continued from previous page...

Atom-1	Atom-2	Interatomic distance (Å)	Clash overlap (Å)
1:C:642:LYS:O	1:C:643:GLU:C	2.51	0.46
1:C:833:VAL:HG22	1:C:872:LEU:O	2.16	0.46
1:D:18:ALA:O	1:D:19:ILE:HG12	2.15	0.46
1:D:337:VAL:HG21	1:D:512:LEU:HD21	1.96	0.46
1:D:571:TYR:HD2	1:D:627:ARG:NH2	2.14	0.46
1:D:801:LYS:HD3	1:D:801:LYS:C	2.36	0.46
2:H:6:DT:C1'	5:H:2008:HOH:O	2.52	0.46
1:A:137:VAL:HG12	1:A:217:ILE:HD11	1.96	0.46
1:A:546:PHE:CZ	1:A:783:VAL:HG21	2.47	0.46
1:B:174:VAL:HG22	5:I:2002:HOH:O	2.15	0.46
1:B:276:LYS:HD2	1:B:283:GLY:O	2.15	0.46
1:B:445:THR:OG1	1:B:446:LEU:N	2.48	0.46
1:B:565:GLU:OE2	1:B:565:GLU:O	2.33	0.46
1:C:452:ILE:HD11	1:C:457:TYR:CA	2.41	0.46
1:C:56:GLU:OE1	1:C:57:ARG:HA	2.15	0.46
1:C:698:TRP:HZ2	1:C:846:TYR:HD2	1.63	0.46
1:D:437:ASN:ND2	1:D:437:ASN:H	2.13	0.46
4:G:7:DT:H72	5:G:2002:HOH:O	2.15	0.46
4:J:1:DG:H1'	5:J:2001:HOH:O	2.14	0.46
1:C:57:ARG:NH1	2:K:17:DG:H3'	2.31	0.46
4:P:2:DT:H4'	5:P:2003:HOH:O	2.15	0.46
1:A:342:THR:HA	1:A:348:PRO:HG3	1.97	0.46
1:B:102:ALA:HB1	1:B:106:LEU:HD12	1.98	0.46
1:B:216:CYS:HA	1:B:219:MET:HE2	1.97	0.46
1:B:257:ARG:HH11	1:B:257:ARG:HG2	1.81	0.46
1:B:463:HIS:CD2	1:B:535:ALA:H	2.34	0.46
1:B:510:CYS:HB3	5:B:2225:HOH:O	2.14	0.46
1:B:706:LEU:HD11	1:B:849:PHE:CG	2.50	0.46
1:C:270:PRO:HG3	1:C:416:PHE:HE1	1.79	0.46
1:C:718:ILE:HB	5:C:2153:HOH:O	2.14	0.46
1:D:118:THR:HG23	1:D:141:ILE:HD13	1.98	0.46
2:E:17:DG:H2"	2:E:18:DC:C5	2.51	0.46
4:P:3:DC:H2"	4:P:4:DG:OP2	2.16	0.46
1:A:230:HIS:CD2	1:A:232:GLN:NE2	2.84	0.46
1:A:281:ILE:HD13	1:A:309:GLU:HA	1.96	0.46
1:A:433:ASN:C	1:A:433:ASN:OD1	2.54	0.46
1:A:778:ILE:HG23	1:A:779:ALA:H	1.81	0.46
1:B:166:VAL:C	1:B:168:GLU:OE1	2.54	0.46
1:B:16:LEU:H	1:B:16:LEU:CD2	2.20	0.46
1:B:781:ASN:HD21	2:H:12:DT:H5'	1.81	0.46
1:C:155:ARG:HB3	1:C:155:ARG:CZ	2.46	0.46

Continued on next page...

Continued from previous page...

Atom-1	Atom-2	Interatomic distance (Å)	Clash overlap (Å)
1:C:199:GLU:O	1:C:199:GLU:CG	2.63	0.46
1:C:487:GLU:HB2	5:C:2116:HOH:O	2.15	0.46
1:C:577:LYS:HB3	1:C:577:LYS:HE2	1.59	0.46
1:C:710:VAL:CG1	1:C:720:ARG:HB3	2.45	0.46
1:C:727:TRP:NE1	1:C:735:VAL:CG1	2.78	0.46
1:C:817:ILE:HD11	1:C:820:ASP:OD2	2.16	0.46
1:D:623:TYR:CD1	1:D:666:MET:HE1	2.51	0.46
1:D:679:LYS:HE2	1:D:679:LYS:HB2	1.66	0.46
2:E:13:DC:H2”	2:E:14:DG:C5’	2.46	0.46
1:A:561:LEU:HD12	1:A:561:LEU:HA	1.69	0.46
1:A:57:ARG:CZ	2:E:18:DC:OP2	2.64	0.46
1:A:57:ARG:HG2	1:A:60:LYS:HZ3	1.80	0.46
1:B:223:SER:HA	5:B:2106:HOH:O	2.16	0.46
1:B:324:GLN:HE21	1:B:417:PRO:HA	1.80	0.46
1:B:676:TYR:O	1:B:680:LEU:HD13	2.16	0.46
1:B:842:LEU:HA	1:B:842:LEU:HD12	1.72	0.46
1:C:502:TRP:CD1	1:C:512:LEU:HD13	2.51	0.46
1:C:645:GLY:O	1:C:649:GLN:HG3	2.16	0.46
1:C:711:LYS:HZ3	1:C:711:LYS:CB	2.27	0.46
1:D:656:GLN:HB3	1:D:657:PRO:CD	2.45	0.46
1:A:375:THR:O	1:A:375:THR:CG2	2.63	0.46
1:A:595:VAL:HG12	1:A:596:THR:N	2.31	0.46
1:A:65:ALA:N	5:A:2057:HOH:O	2.42	0.46
1:B:21:PHE:HD1	1:B:21:PHE:C	2.15	0.46
1:B:416:PHE:HE1	1:B:432:PHE:O	1.98	0.46
1:B:512:LEU:HD11	1:B:516:PHE:CZ	2.51	0.46
1:B:706:LEU:HD21	1:B:849:PHE:HB2	1.98	0.46
1:C:109:ILE:HD12	1:C:109:ILE:N	2.31	0.46
1:D:274:PRO:HD3	1:D:415:TRP:CZ3	2.51	0.46
3:F:1:G:H5”	5:F:2002:HOH:O	2.16	0.46
1:C:644:PHE:CD1	2:K:9:DA:C2	3.04	0.46
1:A:185:VAL:HG23	1:A:186:VAL:N	2.31	0.46
1:A:6:ILE:CG2	1:A:10:ASP:CG	2.84	0.46
1:B:17:ALA:HA	5:B:2049:HOH:O	2.16	0.46
1:B:232:GLN:O	1:B:240:ASP:HA	2.16	0.46
1:B:71:LYS:N	1:B:72:PRO:CD	2.78	0.46
1:B:787:ASP:OD1	1:B:788:GLY:N	2.49	0.46
1:C:19:ILE:HD13	1:C:20:PRO:HD2	1.97	0.46
1:C:744:GLN:O	1:C:745:THR:OG1	2.27	0.46
1:C:868:GLY:C	1:C:869:ASN:HD22	2.19	0.46
1:D:39:LEU:CD2	1:D:272:VAL:HG23	2.46	0.46

Continued on next page...

Continued from previous page...

Atom-1	Atom-2	Interatomic distance (Å)	Clash overlap (Å)
1:D:746:ARG:NH1	1:D:754:GLN:O	2.49	0.46
1:A:89:PHE:CE2	1:A:107:GLN:HG3	2.46	0.45
1:A:24:LEU:HD21	1:A:287:TRP:CE2	2.51	0.45
1:A:577:LYS:HE2	1:A:580:GLU:OE1	2.16	0.45
1:B:123:LEU:O	1:B:127:THR:HG23	2.16	0.45
1:B:418:TYR:HD2	1:B:426:VAL:HG12	1.80	0.45
1:B:619:GLN:NE2	1:B:666:MET:O	2.49	0.45
1:B:663:LYS:HE3	1:B:666:MET:HE2	1.98	0.45
1:B:727:TRP:HB2	5:B:2307:HOH:O	2.15	0.45
1:C:208:ASP:O	1:C:212:VAL:HG23	2.16	0.45
1:C:453:GLY:O	1:C:526:LEU:HD22	2.17	0.45
1:C:610:LYS:O	1:C:611:LEU:C	2.52	0.45
1:C:89:PHE:HE2	1:C:107:GLN:HA	1.80	0.45
1:A:481:PHE:HD2	1:A:482:ILE:HD13	1.81	0.45
1:A:80:LYS:CE	1:A:224:THR:HG22	2.44	0.45
1:A:84:ARG:NH2	1:A:222:GLU:CD	2.70	0.45
1:B:233:ASN:OD1	1:B:239:GLN:HB3	2.16	0.45
1:B:64:VAL:HG21	1:B:127:THR:HG21	1.96	0.45
1:B:677:MET:HG3	1:B:681:ILE:HD12	1.97	0.45
1:B:746:ARG:NH1	5:B:2315:HOH:O	2.49	0.45
1:C:126:LEU:HD23	1:C:132:THR:HG22	1.96	0.45
1:C:16:LEU:HD13	5:C:2034:HOH:O	2.16	0.45
1:D:567:VAL:HG22	1:D:880:PHE:CG	2.51	0.45
1:B:688:THR:HG23	5:B:2301:HOH:O	2.15	0.45
1:B:804:ILE:HG23	1:B:816:THR:HG21	1.99	0.45
1:B:859:ASP:OD1	1:B:859:ASP:C	2.54	0.45
1:C:422:TRP:CZ2	1:C:423:ARG:HD2	2.51	0.45
1:C:437:ASN:HD22	1:C:440:THR:HB	1.82	0.45
1:C:730:PRO:CD	1:C:786:GLN:NE2	2.80	0.45
1:D:173:ARG:O	1:D:179:LYS:HE3	2.17	0.45
1:D:379:ARG:HG2	5:D:2146:HOH:O	2.16	0.45
1:D:449:GLY:HA3	1:D:531:SER:HB3	1.97	0.45
1:D:582:LEU:CD1	1:D:625:VAL:HG21	2.43	0.45
1:D:749:LEU:HD11	5:D:2044:HOH:O	2.15	0.45
1:D:828:VAL:HG23	1:D:829:ARG:N	2.31	0.45
1:A:486:HIS:O	1:A:490:MET:HG2	2.17	0.45
1:A:706:LEU:HB2	5:A:2265:HOH:O	2.15	0.45
1:A:709:GLU:O	1:A:711:LYS:HG3	2.16	0.45
1:A:551:ARG:HB2	1:A:868:GLY:H	1.80	0.45
1:B:16:LEU:HD13	1:B:38:ALA:CA	2.46	0.45
1:B:744:GLN:C	1:B:745:THR:HG1	2.19	0.45

Continued on next page...

Continued from previous page...

Atom-1	Atom-2	Interatomic distance (Å)	Clash overlap (Å)
1:C:77:LEU:C	1:C:79:PRO:HD2	2.37	0.45
1:D:717:GLU:O	1:D:719:LEU:CD1	2.64	0.45
1:D:810:ILE:O	1:D:812:ASP:N	2.50	0.45
1:C:422:TRP:HH2	2:K:12:DT:H5"	1.78	0.45
1:A:185:VAL:CG2	1:A:186:VAL:N	2.79	0.45
1:A:269:GLN:NE2	1:A:407:LYS:HZ2	2.09	0.45
1:B:420:MET:HA	1:B:425:ARG:O	2.17	0.45
1:B:561:LEU:HB3	1:B:562:LEU:HD23	1.97	0.45
1:B:62:GLY:CA	1:B:124:ALA:HA	2.45	0.45
1:B:6:ILE:HG23	1:B:10:ASP:OD2	2.16	0.45
1:B:561:LEU:HD23	1:B:870:LEU:HD11	1.98	0.45
1:C:173:ARG:CZ	5:C:2062:HOH:O	2.63	0.45
1:C:550:LEU:HD21	1:C:865:PRO:HG2	1.97	0.45
1:C:706:LEU:HD21	1:C:849:PHE:CB	2.43	0.45
1:D:700:LYS:HA	1:D:778:ILE:HG21	1.98	0.45
1:A:352:ILE:HA	1:A:353:PRO:HD3	1.88	0.45
1:A:481:PHE:CD2	1:A:482:ILE:HD13	2.52	0.45
1:A:50:ARG:CG	1:A:50:ARG:NH1	2.77	0.45
1:A:529:ASN:HD22	1:A:530:CYS:N	2.14	0.45
1:B:19:ILE:O	1:B:21:PHE:N	2.42	0.45
1:B:244:ILE:O	1:B:245:GLU:OE2	2.34	0.45
1:B:632:ARG:HA	1:B:635:MET:HG2	1.97	0.45
1:B:680:LEU:N	1:B:680:LEU:CD1	2.79	0.45
1:C:532:LEU:CD2	1:C:534:LEU:CD2	2.92	0.45
1:C:854:HIS:CG	1:C:855:GLU:N	2.84	0.45
1:D:159:ALA:HB2	1:D:190:MET:HE1	1.99	0.45
1:D:72:PRO:HG3	1:D:257:ARG:HG3	1.99	0.45
1:D:433:ASN:HB2	1:D:434:PRO:CD	2.47	0.45
1:D:457:TYR:CE1	1:D:521:VAL:HG11	2.52	0.45
4:G:5:DA:P	5:G:2004:HOH:O	2.75	0.45
1:A:84:ARG:NE	1:A:222:GLU:CB	2.76	0.45
1:A:25:ALA:HA	1:A:29:GLY:O	2.17	0.45
1:B:247:ALA:HB3	1:B:250:TYR:HD1	1.82	0.45
1:B:577:LYS:HE2	1:B:687:VAL:CG2	2.47	0.45
1:C:549:MET:SD	1:C:842:LEU:HD13	2.56	0.45
1:C:6:ILE:HD11	1:C:259:GLY:O	2.16	0.45
1:C:850:ALA:O	1:C:853:LEU:HG	2.16	0.45
1:D:474:PRO:O	1:D:478:ARG:HG3	2.16	0.45
1:D:644:PHE:CD2	1:D:644:PHE:C	2.89	0.45
1:A:420:MET:HA	1:A:425:ARG:O	2.16	0.45
1:A:59:LEU:HA	1:A:64:VAL:CG2	2.39	0.45

Continued on next page...

Continued from previous page...

Atom-1	Atom-2	Interatomic distance (Å)	Clash overlap (Å)
1:A:629:VAL:HG11	1:A:677:MET:CE	2.46	0.45
1:B:84:ARG:HB2	1:B:223:SER:HB3	1.97	0.45
1:C:571:TYR:HD1	1:C:634:VAL:HG11	1.82	0.45
1:C:685:VAL:HG23	1:C:686:SER:N	2.32	0.45
1:C:727:TRP:CE2	1:C:735:VAL:HG11	2.52	0.45
1:C:819:ALA:C	5:C:2175:HOH:O	2.55	0.45
1:A:392:LYS:O	1:A:396:ILE:HG12	2.17	0.45
1:A:417:PRO:HG2	1:A:429:VAL:HB	1.99	0.45
1:A:82:ILE:O	1:A:86:ASN:CG	2.56	0.45
1:B:112:GLU:CD	1:B:112:GLU:H	2.20	0.45
1:B:161:HIS:O	1:B:164:LYS:HG2	2.16	0.45
1:B:331:ASN:HB2	1:B:445:THR:CG2	2.45	0.45
1:B:458:TYR:CE1	1:B:479:ILE:HD11	2.52	0.45
1:B:463:HIS:CD2	1:B:534:LEU:HA	2.52	0.45
1:B:595:VAL:HG12	5:B:2264:HOH:O	2.16	0.45
1:B:744:GLN:HA	1:B:756:ARG:CZ	2.47	0.45
1:C:629:VAL:O	1:C:629:VAL:HG12	2.16	0.45
2:E:13:DC:H2”	2:E:14:DG:H5’	1.99	0.45
1:A:109:ILE:N	1:A:109:ILE:CD1	2.80	0.45
1:A:133:THR:HG22	1:A:243:THR:HG22	1.99	0.45
1:A:463:HIS:CD2	1:A:534:LEU:HA	2.51	0.45
1:A:54:MET:HG3	5:A:2055:HOH:O	2.16	0.45
1:A:553:GLU:HG2	1:A:554:VAL:H	1.82	0.45
1:A:573:ILE:HD12	1:A:573:ILE:C	2.38	0.45
1:A:632:ARG:HA	1:A:635:MET:HG3	1.99	0.45
1:A:58:GLN:OE1	1:A:66:ASP:O	2.34	0.45
1:A:840:ASP:O	1:A:842:LEU:N	2.50	0.45
1:A:560:ASN:ND2	1:A:880:PHE:CB	2.80	0.45
1:A:89:PHE:HZ	1:A:106:LEU:O	2.00	0.45
1:B:102:ALA:HB1	1:B:106:LEU:CD1	2.47	0.45
1:B:125:CYS:O	1:B:128:SER:HB3	2.17	0.45
1:B:206:LYS:HG2	1:B:207:GLU:OE2	2.17	0.45
1:B:247:ALA:HB3	1:B:250:TYR:CD1	2.52	0.45
1:C:162:PHE:CE2	1:C:165:ASN:HB2	2.52	0.45
1:C:419:ASN:HD22	1:C:419:ASN:HA	1.48	0.45
1:C:780:PRO:O	1:C:783:VAL:HG23	2.17	0.45
1:C:882:PHE:CD1	1:C:882:PHE:N	2.67	0.45
1:D:632:ARG:HH11	1:D:632:ARG:HB2	1.81	0.45
2:E:3:DG:H2”	2:E:4:DA:OP2	2.16	0.45
1:B:171:ASN:HB3	3:I:2:C:H4’	1.99	0.45
1:A:514:PHE:CD1	1:A:515:CYS:N	2.85	0.44

Continued on next page...

Continued from previous page...

Atom-1	Atom-2	Interatomic distance (Å)	Clash overlap (Å)
1:A:560:ASN:ND2	1:A:880:PHE:HB2	2.31	0.44
1:A:76:THR:C	1:A:79:PRO:HD2	2.38	0.44
1:B:221:ILE:CD1	1:B:229:LEU:HB2	2.47	0.44
1:B:332:LYS:HA	5:B:2150:HOH:O	2.17	0.44
1:B:423:ARG:NE	2:H:12:DT:C4'	2.68	0.44
1:B:532:LEU:O	1:B:818:PRO:HD3	2.18	0.44
1:B:647:ARG:HG2	1:B:675:GLY:HA2	1.98	0.44
1:B:752:LEU:HB2	1:B:753:GLY:H	1.48	0.44
1:C:206:LYS:O	1:C:210:ILE:HG12	2.18	0.44
1:C:541:SER:O	1:C:545:HIS:HD2	1.99	0.44
1:C:611:LEU:HD22	1:C:615:ALA:CB	2.47	0.44
1:C:147:ASP:CB	1:C:750:MET:HE1	2.36	0.44
1:C:860:LYS:CD	1:C:860:LYS:O	2.63	0.44
1:D:27:HIS:HB3	1:D:28:TYR:CE1	2.51	0.44
1:D:737:GLN:HG2	1:D:774:GLN:HE22	1.83	0.44
1:A:560:ASN:ND2	1:A:567:VAL:HG13	2.21	0.44
1:A:726:HIS:CD2	1:A:736:TRP:NE1	2.86	0.44
1:A:811:HIS:HA	5:A:2283:HOH:O	2.16	0.44
1:A:854:HIS:HD2	1:A:856:SER:H	1.53	0.44
1:B:185:VAL:CG2	1:B:186:VAL:N	2.79	0.44
1:B:573:ILE:CD1	1:B:688:THR:HG21	2.45	0.44
1:B:847:ASP:OD1	1:B:847:ASP:N	2.50	0.44
1:B:857:GLN:HA	5:B:2340:HOH:O	2.18	0.44
1:C:257:ARG:HD3	1:C:261:LEU:HD13	1.97	0.44
1:C:275:PRO:HB2	1:C:324:GLN:CG	2.47	0.44
1:C:526:LEU:HA	1:C:526:LEU:HD23	1.72	0.44
1:C:80:LYS:HD3	1:C:224:THR:CG2	2.35	0.44
1:D:170:LEU:O	1:D:173:ARG:HB2	2.17	0.44
1:A:332:LYS:HE2	1:A:410:ASN:HD22	1.78	0.44
1:A:666:MET:HB3	1:A:666:MET:HE3	1.83	0.44
1:B:183:MET:CE	1:B:183:MET:HA	2.47	0.44
1:B:401:MET:HE1	1:B:432:PHE:CB	2.46	0.44
1:B:748:ASN:HB3	1:B:752:LEU:C	2.38	0.44
1:C:726:HIS:CD2	1:C:727:TRP:N	2.86	0.44
1:C:81:MET:O	1:C:85:ILE:HG13	2.18	0.44
1:D:276:LYS:HD2	1:D:283:GLY:O	2.16	0.44
1:D:54:MET:O	1:D:58:GLN:HG2	2.17	0.44
1:D:789:SER:HA	1:D:792:ARG:NH2	2.32	0.44
1:A:711:LYS:HG2	1:A:718:ILE:HA	1.99	0.44
1:A:746:ARG:CZ	1:A:746:ARG:HB3	2.43	0.44
1:C:89:PHE:HE2	1:C:107:GLN:HG3	1.83	0.44

Continued on next page...

Continued from previous page...

Atom-1	Atom-2	Interatomic distance (Å)	Clash overlap (Å)
1:C:108:GLU:OE1	1:C:108:GLU:CA	2.64	0.44
1:C:448:LYS:HG3	1:C:448:LYS:O	2.17	0.44
1:C:482:ILE:HD12	1:C:514:PHE:HZ	1.81	0.44
1:C:801:LYS:CE	1:C:801:LYS:O	2.64	0.44
1:D:543:ILE:HG21	1:D:689:VAL:HG11	1.99	0.44
1:D:859:ASP:OD2	1:D:860:LYS:N	2.51	0.44
2:K:5:DA:H2”	2:K:6:DT:OP2	2.17	0.44
1:A:320:ILE:HG12	1:A:320:ILE:H	1.62	0.44
1:A:625:VAL:C	1:A:626:THR:HG23	2.37	0.44
1:A:744:GLN:H	1:A:744:GLN:HG3	1.51	0.44
1:C:709:GLU:HG2	1:C:709:GLU:O	2.16	0.44
1:C:860:LYS:O	1:C:862:PRO:HD3	2.18	0.44
1:D:463:HIS:CD2	1:D:534:LEU:HA	2.52	0.44
1:D:319:ALA:HB3	1:D:792:ARG:HG2	1.97	0.44
1:D:532:LEU:O	1:D:818:PRO:HD3	2.17	0.44
1:A:133:THR:HA	1:A:243:THR:HA	2.00	0.44
1:A:203:SER:O	1:A:205:HIS:N	2.49	0.44
1:A:229:LEU:HD11	1:A:242:GLU:HG2	2.00	0.44
1:A:569:ASP:CG	1:A:627:ARG:HH21	2.21	0.44
1:A:711:LYS:HB3	1:A:717:GLU:O	2.17	0.44
1:A:804:ILE:CG2	1:A:816:THR:HG21	2.38	0.44
1:B:230:HIS:O	1:B:232:GLN:NE2	2.48	0.44
1:B:272:VAL:HG13	1:B:411:HIS:CD2	2.53	0.44
1:B:427:TYR:N	1:B:427:TYR:CD1	2.85	0.44
1:C:164:LYS:NZ	5:C:2060:HOH:O	2.50	0.44
1:C:5:ASN:HA	1:C:52:ARG:HH11	1.82	0.44
1:D:159:ALA:O	1:D:160:LYS:C	2.56	0.44
1:D:81:MET:HE2	1:D:220:LEU:HA	2.00	0.44
1:D:619:GLN:O	1:D:622:ALA:HB3	2.17	0.44
2:K:15:DC:H2”	2:K:16:DC:C5’	2.47	0.44
1:A:65:ALA:HB3	1:A:120:LYS:HE2	2.00	0.44
1:A:155:ARG:HB3	1:A:749:LEU:HD21	2.00	0.44
1:A:159:ALA:O	1:A:160:LYS:C	2.56	0.44
1:A:744:GLN:HB3	1:A:756:ARG:HB2	2.00	0.44
1:B:195:LEU:N	5:B:2099:HOH:O	2.49	0.44
1:B:534:LEU:HD11	1:B:818:PRO:HG3	2.00	0.44
1:C:16:LEU:HD13	1:C:38:ALA:CA	2.47	0.44
1:C:227:VAL:HG12	1:C:244:ILE:HG22	2.00	0.44
1:C:416:PHE:O	1:C:418:TYR:CD1	2.70	0.44
1:C:630:THR:HG22	1:C:681:ILE:HD13	2.00	0.44
1:D:120:LYS:O	1:D:120:LYS:HD3	2.18	0.44

Continued on next page...

Continued from previous page...

Atom-1	Atom-2	Interatomic distance (Å)	Clash overlap (Å)
1:D:185:VAL:HG23	1:D:186:VAL:N	2.32	0.44
1:D:328:TRP:O	1:D:413:ALA:HA	2.18	0.44
1:D:327:ALA:CB	1:D:448:LYS:HE2	2.47	0.44
1:D:463:HIS:CE1	5:D:2121:HOH:O	2.70	0.44
1:D:663:LYS:CG	1:D:664:GLY:N	2.76	0.44
1:D:717:GLU:O	1:D:719:LEU:HD13	2.18	0.44
2:H:13:DC:H2"	2:H:14:DG:C5'	2.48	0.44
1:D:389:LYS:NZ	3:O:3:G:O2'	2.51	0.44
1:B:335:LEU:HD22	1:B:339:ASN:CG	2.37	0.44
1:B:630:THR:O	1:B:634:VAL:CG1	2.66	0.44
1:B:720:ARG:HE	1:B:854:HIS:H	1.65	0.44
1:B:880:PHE:N	1:B:880:PHE:CD1	2.81	0.44
1:C:313:MET:N	1:C:314:PRO:HD3	2.32	0.44
1:C:349:VAL:HG13	1:C:503:ALA:O	2.17	0.44
1:C:273:VAL:HA	1:C:415:TRP:CZ3	2.52	0.44
1:D:232:GLN:NE2	1:D:243:THR:OG1	2.51	0.44
1:D:349:VAL:HG12	5:D:2096:HOH:O	2.18	0.44
1:A:176:HIS:HD2	1:A:751:PHE:CE1	2.36	0.44
1:A:247:ALA:HA	1:A:248:PRO:HD3	1.88	0.44
1:A:378:LYS:HD2	1:A:378:LYS:HA	1.90	0.44
1:A:631:LYS:O	1:A:634:VAL:HG12	2.18	0.44
1:B:257:ARG:CG	5:B:2125:HOH:O	2.57	0.44
1:C:326:THR:O	1:C:415:TRP:CD1	2.71	0.44
1:C:454:LYS:HG3	1:C:455:GLU:N	2.32	0.44
1:C:479:ILE:HA	1:C:479:ILE:HD13	1.88	0.44
1:C:718:ILE:HD12	5:C:2153:HOH:O	2.17	0.44
1:D:236:VAL:CB	1:D:239:GLN:HB2	2.48	0.44
1:D:30:GLU:O	1:D:34:ARG:HG3	2.18	0.44
2:E:15:DC:H2"	2:E:16:DC:C5'	2.47	0.44
1:A:115:ALA:O	1:A:119:ILE:CG1	2.66	0.43
1:A:178:TYR:N	1:A:178:TYR:CD2	2.86	0.43
1:B:281:ILE:HG23	1:B:305:LEU:CD2	2.46	0.43
1:B:412:LYS:O	1:B:413:ALA:HB2	2.17	0.43
1:D:42:GLU:HG2	1:D:46:MET:HE1	1.99	0.43
1:D:487:GLU:OE1	1:D:487:GLU:HA	2.17	0.43
1:D:337:VAL:HG11	1:D:502:TRP:CZ2	2.52	0.43
1:D:641:SER:HA	2:N:10:DC:H5'	1.99	0.43
1:D:751:PHE:HD2	1:D:752:LEU:HD11	1.82	0.43
1:D:873:ARG:HH11	1:D:876:LEU:CD1	2.30	0.43
1:A:645:GLY:HA3	2:E:10:DC:OP2	2.18	0.43
1:A:84:ARG:NE	1:A:222:GLU:HB3	2.33	0.43

Continued on next page...

Continued from previous page...

Atom-1	Atom-2	Interatomic distance (Å)	Clash overlap (Å)
1:A:449:GLY:C	1:A:529:ASN:HD21	2.22	0.43
1:C:143:ARG:NH1	1:C:209:SER:HB2	2.33	0.43
1:C:455:GLU:O	1:C:458:TYR:HB3	2.18	0.43
1:C:790:HIS:HD1	1:C:790:HIS:C	2.21	0.43
1:D:348:PRO:CD	5:D:2092:HOH:O	2.65	0.43
1:A:104:GLN:HB2	1:A:105:PHE:CD2	2.54	0.43
1:A:232:GLN:HB2	1:A:241:SER:O	2.19	0.43
1:A:269:GLN:HB3	1:A:270:PRO:CD	2.49	0.43
1:A:308:TYR:C	1:A:311:VAL:HG23	2.38	0.43
1:A:422:TRP:C	1:A:422:TRP:CD1	2.90	0.43
1:A:700:LYS:HA	1:A:778:ILE:HG21	2.00	0.43
1:A:825:PHE:CE2	1:A:829:ARG:NH2	2.87	0.43
1:B:193:LYS:HB2	1:B:194:GLY:H	1.55	0.43
1:B:231:ARG:HG2	1:B:234:ALA:HB2	2.00	0.43
1:B:88:TRP:O	1:B:92:VAL:HG23	2.18	0.43
1:C:404:GLN:HG2	1:C:432:PHE:HB2	2.00	0.43
1:C:536:PHE:CE1	1:C:825:PHE:HD2	2.36	0.43
1:C:779:ALA:HB3	1:C:780:PRO:CD	2.48	0.43
1:C:720:ARG:NH2	1:C:857:GLN:OE1	2.34	0.43
1:D:651:LEU:O	1:D:656:GLN:HB2	2.18	0.43
1:D:750:MET:HG2	1:D:750:MET:H	1.64	0.43
1:A:641:SER:CB	2:E:10:DC:H5'	2.48	0.43
1:A:115:ALA:O	1:A:119:ILE:HD11	2.17	0.43
1:A:249:GLU:CG	5:A:2109:HOH:O	2.66	0.43
1:A:779:ALA:O	1:A:780:PRO:C	2.56	0.43
1:B:6:ILE:O	1:B:8:LYS:N	2.40	0.43
1:C:741:LYS:HG2	5:C:2159:HOH:O	2.16	0.43
1:D:148:GLU:CG	5:D:2041:HOH:O	2.65	0.43
1:D:177:VAL:HG12	1:D:178:TYR:CD2	2.53	0.43
1:D:231:ARG:NH1	1:D:242:GLU:HB2	2.33	0.43
1:D:446:LEU:HD12	1:D:817:ILE:CG2	2.48	0.43
1:D:450:LYS:HD2	1:D:817:ILE:HD11	2.01	0.43
1:D:663:LYS:HE3	1:D:666:MET:HE3	2.01	0.43
1:D:726:HIS:CD2	1:D:727:TRP:H	2.33	0.43
1:A:14:ILE:O	1:A:16:LEU:HD23	2.18	0.43
1:A:57:ARG:HB3	1:A:60:LYS:HZ1	1.83	0.43
1:A:829:ARG:HG3	1:A:829:ARG:NH1	2.32	0.43
1:A:842:LEU:HD23	1:A:864:LEU:CD2	2.47	0.43
1:B:221:ILE:CG1	1:B:227:VAL:HG23	2.49	0.43
1:B:663:LYS:CG	1:B:664:GLY:N	2.64	0.43
1:B:825:PHE:CE2	1:B:829:ARG:NH2	2.87	0.43

Continued on next page...

Continued from previous page...

Atom-1	Atom-2	Interatomic distance (Å)	Clash overlap (Å)
1:C:514:PHE:HD1	1:C:515:CYS:N	2.15	0.43
1:D:372:GLU:HB2	5:D:2105:HOH:O	2.17	0.43
1:D:446:LEU:HB2	1:D:531:SER:O	2.19	0.43
1:A:230:HIS:HE2	1:A:245:GLU:HG2	1.84	0.43
1:A:481:PHE:C	1:A:481:PHE:CD2	2.91	0.43
1:B:157:LEU:HG	1:B:195:LEU:CD2	2.48	0.43
1:B:719:LEU:CD1	1:B:719:LEU:N	2.81	0.43
1:B:754:GLN:HB3	1:B:755:PHE:H	1.54	0.43
1:C:217:ILE:O	1:C:221:ILE:HG13	2.19	0.43
1:C:871:ASN:O	1:C:873:ARG:N	2.52	0.43
1:D:163:LYS:O	1:D:166:VAL:HB	2.19	0.43
1:D:281:ILE:HA	1:D:281:ILE:HD12	1.67	0.43
1:D:571:TYR:HD1	1:D:634:VAL:HG11	1.82	0.43
1:B:781:ASN:HD21	2:H:12:DT:C5’	2.31	0.43
1:B:272:VAL:HG13	1:B:411:HIS:HD2	1.82	0.43
1:B:656:GLN:HB3	1:B:657:PRO:HD2	2.00	0.43
1:B:668:THR:O	1:B:670:PRO:HD3	2.19	0.43
1:B:730:PRO:CG	1:B:786:GLN:HE22	2.31	0.43
1:C:147:ASP:OD1	1:C:180:LYS:HE3	2.18	0.43
1:C:459:TRP:HB3	1:C:534:LEU:CD1	2.49	0.43
1:C:523:HIS:N	1:C:523:HIS:CD2	2.84	0.43
1:C:623:TYR:HA	1:C:666:MET:HE1	1.99	0.43
1:C:700:LYS:HA	1:C:778:ILE:HG21	2.01	0.43
1:D:148:GLU:HG3	5:D:2041:HOH:O	2.18	0.43
1:D:46:MET:HE1	1:D:269:GLN:OE1	2.19	0.43
1:D:374:LEU:C	1:D:376:ALA:H	2.21	0.43
1:D:828:VAL:CG2	1:D:883:ALA:HA	2.48	0.43
2:N:6:DT:H2’	5:N:2008:HOH:O	2.19	0.43
2:N:5:DA:C2	4:P:7:DT:O2	2.72	0.43
1:A:651:LEU:HD13	1:A:651:LEU:C	2.39	0.43
1:B:236:VAL:HG11	1:B:239:GLN:NE2	2.34	0.43
1:B:275:PRO:HD3	1:B:415:TRP:CB	2.48	0.43
1:C:154:ILE:HD13	1:C:190:MET:CE	2.48	0.43
1:C:664:GLY:HA2	1:C:667:PHE:CD2	2.54	0.43
1:D:343:LYS:HE3	1:D:343:LYS:HB3	1.90	0.43
1:D:353:PRO:CD	5:D:2098:HOH:O	2.65	0.43
2:H:13:DC:H2”	2:H:14:DG:H5’	2.01	0.43
1:A:24:LEU:HD21	1:A:287:TRP:CG	2.51	0.43
1:A:294:LEU:HA	1:A:294:LEU:HD23	1.79	0.43
1:A:61:ALA:HB3	1:A:63:GLU:HG3	2.01	0.43
1:A:621:LEU:HA	1:A:621:LEU:HD23	1.82	0.43

Continued on next page...

Continued from previous page...

Atom-1	Atom-2	Interatomic distance (Å)	Clash overlap (Å)
1:A:881:ALA:HA	5:A:2310:HOH:O	2.16	0.43
1:A:814:PHE:HE1	1:A:883:ALA:HB2	1.80	0.43
1:B:106:LEU:HD21	1:B:212:VAL:HG13	1.97	0.43
1:B:201:TRP:O	1:B:204:TRP:HB2	2.19	0.43
1:B:224:THR:HB	5:B:2107:HOH:O	2.18	0.43
1:B:833:VAL:HG12	1:B:834:ASP:N	2.32	0.43
1:C:632:ARG:HG2	5:C:2145:HOH:O	2.19	0.43
1:C:828:VAL:C	1:C:831:THR:HG22	2.38	0.43
1:D:215:ARG:HA	1:D:215:ARG:HD2	1.81	0.43
1:D:406:ASN:CB	5:D:2112:HOH:O	2.64	0.43
1:D:567:VAL:HA	1:D:880:PHE:CD2	2.54	0.43
5:B:2341:HOH:O	4:J:5:DA:H5”	2.18	0.43
1:A:772:HIS:CE1	4:G:6:DT:H5’	2.53	0.43
1:B:162:PHE:O	1:B:162:PHE:CD2	2.71	0.43
1:B:164:LYS:HE3	1:B:164:LYS:N	2.33	0.43
1:B:16:LEU:HD13	1:B:38:ALA:CB	2.49	0.43
1:B:216:CYS:N	1:B:219:MET:HE2	2.33	0.43
1:B:296:LEU:O	1:B:296:LEU:HG	2.18	0.43
1:B:42:GLU:HG2	1:B:46:MET:CE	2.49	0.43
1:B:610:LYS:O	1:B:611:LEU:C	2.57	0.43
1:B:7:ALA:CB	5:B:2057:HOH:O	2.67	0.43
1:C:158:GLU:HG2	1:C:195:LEU:CD2	2.49	0.43
1:C:195:LEU:HA	1:C:195:LEU:HD12	1.81	0.43
1:D:101:THR:HG21	5:D:2055:HOH:O	2.18	0.43
1:D:126:LEU:HD21	1:D:244:ILE:CG2	2.42	0.43
1:D:490:MET:CE	1:D:522:GLN:HG3	2.49	0.43
1:D:668:THR:O	1:D:670:PRO:HD3	2.19	0.43
1:D:829:ARG:HD3	1:D:875:ILE:O	2.19	0.43
1:A:234:ALA:HA	1:A:240:ASP:OD2	2.19	0.42
1:B:343:LYS:HB3	1:B:343:LYS:HE3	1.76	0.42
1:B:401:MET:HE3	1:B:432:PHE:HD1	1.83	0.42
1:B:629:VAL:HG12	1:B:629:VAL:O	2.18	0.42
1:B:726:HIS:CD2	1:B:736:TRP:CE2	3.07	0.42
1:C:231:ARG:CZ	1:C:242:GLU:HB2	2.49	0.42
1:C:509:PHE:CD2	1:C:509:PHE:N	2.87	0.42
1:C:807:PHE:N	1:C:807:PHE:CD1	2.87	0.42
1:C:810:ILE:HB	1:C:813:SER:HB3	1.99	0.42
1:C:828:VAL:O	1:C:831:THR:CG2	2.67	0.42
1:D:274:PRO:HD3	1:D:415:TRP:CH2	2.54	0.42
1:D:722:ARG:HD2	1:D:768:GLU:OE2	2.19	0.42
1:D:93:LYS:HA	1:D:99:ARG:HH12	1.83	0.42

Continued on next page...

Continued from previous page...

Atom-1	Atom-2	Interatomic distance (Å)	Clash overlap (Å)
1:A:8:LYS:O	1:A:12:SER:HB2	2.18	0.42
1:A:298:ARG:HG3	1:A:420:MET:O	2.18	0.42
1:A:724:ALA:HA	1:A:774:GLN:NE2	2.34	0.42
1:B:141:ILE:H	1:B:141:ILE:HG13	1.58	0.42
1:B:699:LEU:O	1:B:778:ILE:HG21	2.19	0.42
1:B:709:GLU:O	1:B:711:LYS:HG3	2.20	0.42
1:B:83:ALA:O	1:B:87:ASP:OD2	2.37	0.42
1:C:641:SER:HA	2:K:10:DC:H5'	2.01	0.42
1:C:746:ARG:HB2	1:C:754:GLN:O	2.19	0.42
1:D:298:ARG:HG3	1:D:420:MET:O	2.19	0.42
1:D:578:VAL:HG13	1:D:680:LEU:HB3	2.00	0.42
1:D:6:ILE:HG23	1:D:10:ASP:CG	2.40	0.42
2:N:12:DT:H2"	2:N:13:DC:O5'	2.20	0.42
1:A:131:ASN:C	1:A:131:ASN:OD1	2.57	0.42
1:A:163:LYS:HD2	1:A:166:VAL:HB	1.99	0.42
1:A:20:PRO:HA	5:A:2043:HOH:O	2.19	0.42
1:A:512:LEU:HG	1:A:516:PHE:CE2	2.53	0.42
1:A:63:GLU:C	5:A:2057:HOH:O	2.57	0.42
1:A:828:VAL:HB	1:A:883:ALA:HA	2.01	0.42
1:B:146:GLU:HG3	1:B:204:TRP:CD2	2.54	0.42
1:B:322:ILE:CD1	1:B:799:HIS:CD2	3.02	0.42
1:B:605:ILE:N	5:B:2273:HOH:O	2.52	0.42
1:B:717:GLU:HB3	5:B:2302:HOH:O	2.19	0.42
1:B:98:LYS:NZ	5:B:2080:HOH:O	2.52	0.42
1:C:231:ARG:NH2	1:C:242:GLU:HB2	2.34	0.42
1:C:291:ARG:HA	1:C:291:ARG:HD3	1.74	0.42
1:C:485:ASN:ND2	1:C:488:ASN:ND2	2.53	0.42
1:C:615:ALA:O	1:C:619:GLN:HG3	2.19	0.42
1:C:677:MET:O	1:C:681:ILE:HG13	2.18	0.42
1:C:833:VAL:O	1:C:837:GLU:HG3	2.19	0.42
1:D:316:VAL:HG12	1:D:317:TYR:N	2.34	0.42
1:D:337:VAL:HG11	1:D:502:TRP:CH2	2.54	0.42
1:D:505:GLN:O	1:D:508:PRO:HD3	2.18	0.42
1:D:846:TYR:CD1	1:D:846:TYR:C	2.93	0.42
1:A:226:MET:HG3	1:A:250:TYR:CD1	2.37	0.42
1:A:625:VAL:C	1:A:626:THR:CG2	2.85	0.42
1:B:253:ALA:CB	5:B:2001:HOH:O	2.67	0.42
1:B:331:ASN:HA	5:B:2151:HOH:O	2.18	0.42
1:B:6:ILE:CB	1:B:48:GLU:OE2	2.66	0.42
1:B:700:LYS:HA	1:B:778:ILE:HG21	2.01	0.42
1:C:274:PRO:HA	1:C:275:PRO:HD3	1.90	0.42

Continued on next page...

Continued from previous page...

Atom-1	Atom-2	Interatomic distance (Å)	Clash overlap (Å)
1:C:502:TRP:CZ2	1:C:512:LEU:HD22	2.55	0.42
1:C:349:VAL:CG1	1:C:503:ALA:O	2.68	0.42
1:C:457:TYR:HD1	1:C:521:VAL:HG21	1.84	0.42
1:D:643:GLU:HG3	1:D:682:TRP:CB	2.50	0.42
1:A:372:GLU:HB2	5:A:2014:HOH:O	2.19	0.42
1:A:685:VAL:HG23	1:A:686:SER:N	2.35	0.42
1:B:182:PHE:O	1:B:186:VAL:HG23	2.20	0.42
1:B:852:GLN:HB2	1:B:852:GLN:HE21	1.47	0.42
1:C:418:TYR:HD2	1:C:426:VAL:HG12	1.82	0.42
1:C:551:ARG:HD3	1:C:872:LEU:HD21	2.01	0.42
1:D:9:ASN:CA	1:D:12:SER:HB3	2.49	0.42
1:D:137:VAL:HG21	5:D:2038:HOH:O	2.20	0.42
1:D:275:PRO:HG2	1:D:324:GLN:CG	2.50	0.42
1:D:326:THR:HG23	1:D:806:SER:CA	2.46	0.42
1:D:659:ILE:HA	1:D:663:LYS:O	2.19	0.42
1:D:6:ILE:O	1:D:8:LYS:N	2.44	0.42
2:E:11:DA:H2”	2:E:12:DT:O5’	2.19	0.42
1:A:14:ILE:HG22	1:A:14:ILE:O	2.20	0.42
1:A:205:HIS:CD2	1:A:206:LYS:HE2	2.53	0.42
1:A:322:ILE:HD13	1:A:799:HIS:CD2	2.54	0.42
1:A:59:LEU:HD23	1:A:64:VAL:HG13	2.00	0.42
1:A:722:ARG:HB3	1:A:722:ARG:HE	1.27	0.42
1:B:313:MET:N	1:B:314:PRO:HD3	2.33	0.42
1:B:412:LYS:HA	1:B:412:LYS:HD2	1.83	0.42
1:B:455:GLU:O	1:B:458:TYR:HB3	2.19	0.42
1:B:475:PHE:HE1	1:B:478:ARG:HH12	1.65	0.42
1:B:490:MET:HE1	1:B:522:GLN:CB	2.50	0.42
1:B:582:LEU:CD2	1:B:620:TRP:HB2	2.50	0.42
1:B:59:LEU:HD23	1:B:64:VAL:HG22	1.99	0.42
1:C:448:LYS:CG	1:C:448:LYS:O	2.67	0.42
1:C:453:GLY:C	1:C:526:LEU:HD22	2.40	0.42
1:C:632:ARG:HA	1:C:635:MET:HG2	2.02	0.42
1:C:748:ASN:ND2	1:C:751:PHE:H	2.17	0.42
1:C:98:LYS:HB3	1:C:98:LYS:HE2	1.91	0.42
4:J:3:DC:C2’	5:J:2002:HOH:O	2.62	0.42
1:A:270:PRO:HD2	1:A:408:PHE:CE2	2.53	0.42
1:A:274:PRO:HA	1:A:275:PRO:HD3	1.94	0.42
1:B:242:GLU:HB3	5:B:2117:HOH:O	2.19	0.42
1:C:89:PHE:CE2	1:C:107:GLN:HA	2.55	0.42
1:C:346:HIS:ND1	1:C:391:ARG:NH2	2.67	0.42
1:C:79:PRO:HG2	1:C:80:LYS:H	1.84	0.42

Continued on next page...

Continued from previous page...

Atom-1	Atom-2	Interatomic distance (Å)	Clash overlap (Å)
1:D:109:ILE:HG23	1:D:148:GLU:HG3	2.01	0.42
1:D:152:GLY:O	1:D:156:ASP:OD2	2.38	0.42
1:D:488:ASN:HB3	1:D:501:TRP:CZ3	2.54	0.42
1:D:666:MET:HG2	1:D:667:PHE:CD1	2.54	0.42
1:D:748:ASN:HB2	1:D:753:GLY:HA2	1.99	0.42
1:D:846:TYR:CD1	1:D:850:ALA:HB2	2.55	0.42
1:D:852:GLN:HB2	1:D:852:GLN:HE21	1.52	0.42
1:C:810:ILE:HG22	3:L:8:U:H5'	2.01	0.42
4:M:7:DT:H2"	4:M:8:DC:C5	2.55	0.42
4:M:8:DC:H2"	4:M:9:DC:OP2	2.20	0.42
1:A:77:LEU:HD22	1:A:226:MET:SD	2.60	0.42
1:A:249:GLU:N	1:A:249:GLU:OE1	2.50	0.42
1:A:292:ARG:NH1	1:A:292:ARG:CG	2.83	0.42
1:A:340:VAL:HG12	1:A:341:ILE:N	2.34	0.42
1:A:573:ILE:HD11	1:A:688:THR:CG2	2.49	0.42
1:A:790:HIS:CD2	1:A:790:HIS:O	2.73	0.42
1:A:814:PHE:CE1	1:A:883:ALA:CB	3.02	0.42
1:B:14:ILE:HG23	1:B:288:ALA:CB	2.46	0.42
1:B:713:LYS:HD2	1:B:713:LYS:HA	1.79	0.42
1:B:860:LYS:HG2	1:B:860:LYS:O	2.20	0.42
1:C:15:GLU:HG2	1:C:18:ALA:N	2.32	0.42
1:C:68:ALA:HB3	1:C:261:LEU:CD2	2.45	0.42
1:C:474:PRO:HB2	1:C:476:PRO:HD2	2.02	0.42
1:C:57:ARG:CD	5:C:2041:HOH:O	2.67	0.42
1:C:586:ALA:HB3	5:C:2136:HOH:O	2.20	0.42
1:C:665:LEU:HA	1:C:665:LEU:HD23	1.88	0.42
1:C:845:PHE:O	1:C:848:GLN:HB2	2.20	0.42
1:D:229:LEU:HG	1:D:229:LEU:O	2.18	0.42
1:D:754:GLN:O	1:D:755:PHE:O	2.37	0.42
1:D:836:TYR:HB2	1:D:872:LEU:HD13	2.02	0.42
2:K:11:DA:H2"	2:K:12:DT:O5'	2.19	0.42
1:A:57:ARG:HA	1:A:60:LYS:HB3	2.02	0.42
1:A:810:ILE:O	1:A:812:ASP:N	2.53	0.42
1:B:11:PHE:N	5:B:2030:HOH:O	2.47	0.42
1:B:160:LYS:NZ	1:B:161:HIS:CE1	2.88	0.42
1:B:231:ARG:CD	1:B:240:ASP:OD1	2.67	0.42
1:B:550:LEU:HD23	1:B:550:LEU:HA	1.68	0.42
1:C:231:ARG:HD2	1:C:240:ASP:OD1	2.20	0.42
1:C:313:MET:HE3	1:C:317:TYR:CE2	2.55	0.42
1:C:71:LYS:N	1:C:72:PRO:CD	2.83	0.42
1:C:827:ALA:O	1:C:831:THR:HG22	2.19	0.42

Continued on next page...

Continued from previous page...

Atom-1	Atom-2	Interatomic distance (Å)	Clash overlap (Å)
1:D:220:LEU:CG	5:D:2059:HOH:O	2.63	0.42
1:D:247:ALA:HA	1:D:248:PRO:HD3	1.87	0.42
1:D:829:ARG:HG3	1:D:829:ARG:NH1	2.26	0.42
4:G:8:DC:N4	5:G:2010:HOH:O	2.52	0.42
1:B:754:GLN:NE2	3:I:1:G:H4'	2.34	0.42
1:A:522:GLN:HG2	1:A:522:GLN:H	1.50	0.42
1:A:542:GLY:HA2	1:A:783:VAL:HG11	2.01	0.42
1:A:571:TYR:HE1	1:A:634:VAL:HG13	1.85	0.42
1:A:713:LYS:CA	1:A:713:LYS:HE3	2.50	0.42
1:A:823:ASN:N	5:A:2294:HOH:O	2.45	0.42
1:B:43:SER:HA	1:B:46:MET:HE3	2.01	0.42
1:B:706:LEU:HD11	1:B:849:PHE:CB	2.50	0.42
1:B:746:ARG:NH1	1:B:754:GLN:O	2.53	0.42
1:C:16:LEU:H	1:C:16:LEU:CD2	2.14	0.42
1:C:333:LYS:C	1:C:516:PHE:HE2	2.24	0.42
1:C:473:VAL:O	1:C:478:ARG:NE	2.33	0.42
1:C:611:LEU:HD22	1:C:615:ALA:HB1	2.02	0.42
1:D:326:THR:O	1:D:415:TRP:CD1	2.73	0.42
1:D:346:HIS:CD2	1:D:391:ARG:NH2	2.88	0.42
1:D:416:PHE:HA	1:D:417:PRO:HD2	1.95	0.42
1:D:47:GLY:O	1:D:50:ARG:HB3	2.20	0.42
1:D:573:ILE:HD12	1:D:573:ILE:C	2.40	0.42
1:D:689:VAL:HA	5:D:2133:HOH:O	2.19	0.42
1:D:698:TRP:CE3	1:D:842:LEU:HG	2.55	0.42
1:C:171:ASN:O	3:L:2:C:O2'	2.37	0.42
1:A:583:GLN:HB3	1:A:583:GLN:HE21	1.44	0.41
1:B:84:ARG:CG	1:B:223:SER:HB3	2.50	0.41
1:B:431:MET:HB3	1:B:431:MET:HE2	1.86	0.41
1:B:54:MET:HA	5:B:2061:HOH:O	2.19	0.41
1:B:655:ILE:O	1:B:659:ILE:HG13	2.20	0.41
1:C:191:LEU:HD23	1:C:191:LEU:HA	1.92	0.41
1:C:329:LYS:HE3	1:C:329:LYS:HB2	1.84	0.41
1:C:350:GLU:HA	1:C:350:GLU:OE1	2.20	0.41
1:C:457:TYR:CE1	1:C:521:VAL:CG1	2.88	0.41
1:D:207:GLU:HA	1:D:207:GLU:OE1	2.20	0.41
1:D:402:LEU:HG	1:D:439:MET:HE2	1.99	0.41
1:D:349:VAL:HG11	1:D:503:ALA:O	2.20	0.41
1:D:710:VAL:CG1	1:D:720:ARG:HB3	2.46	0.41
2:K:13:DC:H2"	2:K:14:DG:C5'	2.50	0.41
1:D:639:TYR:CE2	2:N:11:DA:C6	3.07	0.41
1:A:179:LYS:HE2	1:A:750:MET:HA	2.02	0.41

Continued on next page...

Continued from previous page...

Atom-1	Atom-2	Interatomic distance (Å)	Clash overlap (Å)
1:B:166:VAL:O	1:B:168:GLU:OE1	2.38	0.41
1:B:15:GLU:HB2	1:B:18:ALA:O	2.19	0.41
1:B:551:ARG:NH2	1:B:836:TYR:O	2.53	0.41
1:C:205:HIS:HD2	1:C:206:LYS:HD3	1.86	0.41
1:C:292:ARG:N	1:C:293:PRO:CD	2.83	0.41
1:D:19:ILE:HD12	1:D:20:PRO:HD2	2.01	0.41
1:D:322:ILE:HD12	1:D:799:HIS:NE2	2.35	0.41
1:D:588:ASN:N	1:D:588:ASN:ND2	2.69	0.41
5:A:2164:HOH:O	3:F:7:A:H1'	2.20	0.41
1:A:727:TRP:CZ2	1:A:735:VAL:HG21	2.55	0.41
1:B:475:PHE:HA	1:B:478:ARG:HD2	2.01	0.41
1:C:137:VAL:O	1:C:141:ILE:HG13	2.20	0.41
1:C:304:ALA:HA	1:C:307:ARG:NH1	2.36	0.41
1:C:402:LEU:HD23	1:C:402:LEU:HA	1.78	0.41
1:C:454:LYS:HG3	1:C:455:GLU:H	1.85	0.41
1:C:801:LYS:NZ	5:C:2174:HOH:O	2.51	0.41
1:D:21:PHE:C	1:D:21:PHE:CD1	2.94	0.41
1:D:488:ASN:O	1:D:491:ALA:HB3	2.20	0.41
1:D:647:ARG:HG2	1:D:675:GLY:HA2	2.00	0.41
1:D:801:LYS:O	1:D:801:LYS:CE	2.69	0.41
1:A:109:ILE:HG13	1:A:149:ALA:HB2	2.01	0.41
1:A:151:PHE:CD1	1:A:183:MET:HB3	2.56	0.41
1:A:810:ILE:HB	1:A:813:SER:HB3	2.02	0.41
1:A:828:VAL:HG11	1:A:883:ALA:HA	2.02	0.41
1:B:110:LYS:HE2	1:B:112:GLU:OE1	2.20	0.41
1:B:473:VAL:HG13	1:B:474:PRO:HD2	2.03	0.41
1:B:476:PRO:HG2	5:B:2209:HOH:O	2.19	0.41
1:B:596:THR:HB	5:B:2263:HOH:O	2.20	0.41
1:B:882:PHE:N	1:B:882:PHE:CD1	2.84	0.41
1:C:272:VAL:HG12	1:C:272:VAL:O	2.20	0.41
1:C:32:LEU:HD12	1:C:32:LEU:HA	1.74	0.41
1:C:416:PHE:O	1:C:418:TYR:CE1	2.74	0.41
1:C:860:LYS:O	1:C:862:PRO:CD	2.69	0.41
1:D:109:ILE:HD12	1:D:109:ILE:N	2.26	0.41
1:D:23:THR:O	1:D:27:HIS:HB2	2.20	0.41
1:D:285:GLY:HA2	1:D:324:GLN:NE2	2.34	0.41
1:D:335:LEU:CD2	1:D:339:ASN:HD21	2.33	0.41
1:D:335:LEU:HD21	1:D:406:ASN:OD1	2.19	0.41
1:D:553:GLU:HB2	5:D:2135:HOH:O	2.19	0.41
1:D:543:ILE:CG2	1:D:559:VAL:HG11	2.50	0.41
1:D:602:THR:CG2	1:D:604:GLU:HB2	2.49	0.41

Continued on next page...

Continued from previous page...

Atom-1	Atom-2	Interatomic distance (Å)	Clash overlap (Å)
1:D:712:ASP:OD2	1:D:714:LYS:HB2	2.20	0.41
1:D:422:TRP:HZ2	1:D:781:ASN:OD1	2.02	0.41
2:H:11:DA:H2”	2:H:12:DT:O5’	2.19	0.41
1:A:269:GLN:HG2	1:A:404:GLN:OE1	2.21	0.41
1:A:748:ASN:HB2	1:A:753:GLY:CA	2.51	0.41
1:B:4:ILE:HD12	1:B:256:THR:HA	2.02	0.41
1:B:264:ILE:C	1:B:266:PRO:HD3	2.41	0.41
1:B:723:CYS:C	5:B:2306:HOH:O	2.58	0.41
1:C:15:GLU:HB3	1:C:18:ALA:O	2.20	0.41
1:C:277:PRO:HA	1:C:321:ASN:OD1	2.21	0.41
1:C:50:ARG:HG2	5:C:2039:HOH:O	2.19	0.41
1:D:594:VAL:HA	1:D:609:VAL:HA	2.02	0.41
1:D:737:GLN:OE1	1:D:774:GLN:NE2	2.53	0.41
1:D:829:ARG:NH1	1:D:829:ARG:CG	2.79	0.41
2:E:5:DA:H2”	2:E:6:DT:O5’	2.19	0.41
1:A:529:ASN:ND2	1:A:529:ASN:C	2.72	0.41
1:A:642:LYS:O	1:A:643:GLU:C	2.57	0.41
1:A:665:LEU:HB2	5:A:2027:HOH:O	2.20	0.41
1:B:347:CYS:HA	1:B:348:PRO:HD3	1.93	0.41
1:B:516:PHE:HB2	5:B:2226:HOH:O	2.21	0.41
1:C:206:LYS:H	1:C:206:LYS:HD3	1.85	0.41
1:C:229:LEU:HD11	1:C:242:GLU:CG	2.50	0.41
1:C:778:ILE:HA	1:C:778:ILE:HD12	1.87	0.41
1:C:549:MET:CE	1:C:841:VAL:HG21	2.50	0.41
1:D:656:GLN:HB3	1:D:657:PRO:HD3	2.03	0.41
4:G:1:DG:C2	4:G:2:DT:O4	2.73	0.41
1:D:772:HIS:CD2	2:N:8:DG:H5”	2.55	0.41
1:A:332:LYS:NZ	1:A:410:ASN:ND2	2.68	0.41
1:A:656:GLN:HB3	1:A:657:PRO:HD3	2.03	0.41
1:A:669:GLN:CG	1:A:672:GLN:NE2	2.64	0.41
1:A:810:ILE:HG22	3:F:8:U:H5’	2.01	0.41
1:A:93:LYS:HA	1:A:99:ARG:HH22	1.76	0.41
1:B:257:ARG:HA	5:B:2125:HOH:O	2.20	0.41
1:B:489:ILE:CG2	1:B:518:TYR:HD1	2.32	0.41
1:B:551:ARG:NE	5:B:2240:HOH:O	2.54	0.41
1:B:663:LYS:HE3	1:B:666:MET:CE	2.51	0.41
1:B:577:LYS:HE2	1:B:687:VAL:HG23	2.01	0.41
1:B:871:ASN:O	1:B:873:ARG:N	2.53	0.41
1:C:176:HIS:C	1:C:176:HIS:ND1	2.73	0.41
1:C:269:GLN:HE22	1:C:407:LYS:HZ3	1.68	0.41
1:C:286:TYR:CD2	1:C:294:LEU:HD11	2.56	0.41

Continued on next page...

Continued from previous page...

Atom-1	Atom-2	Interatomic distance (Å)	Clash overlap (Å)
1:C:308:TYR:HA	1:C:311:VAL:CG2	2.50	0.41
1:C:437:ASN:C	1:C:437:ASN:ND2	2.74	0.41
1:C:507:SER:HA	1:C:508:PRO:HD2	1.85	0.41
1:C:632:ARG:HD2	1:C:653:ASP:OD2	2.20	0.41
1:C:6:ILE:HG22	1:C:10:ASP:CG	2.41	0.41
1:C:754:GLN:HB3	1:C:755:PHE:H	1.53	0.41
1:D:150:ARG:O	1:D:153:ARG:HB3	2.21	0.41
1:D:777:GLY:O	1:D:781:ASN:HB2	2.20	0.41
1:D:846:TYR:CE1	1:D:850:ALA:HB2	2.55	0.41
4:G:7:DT:C7	5:G:2002:HOH:O	2.68	0.41
1:A:163:LYS:C	1:A:164:LYS:HZ3	2.23	0.41
1:A:249:GLU:N	1:A:249:GLU:CD	2.74	0.41
1:A:2:ASN:N	5:A:2002:HOH:O	2.53	0.41
1:A:402:LEU:HD23	1:A:402:LEU:HA	1.82	0.41
1:A:526:LEU:HA	1:A:526:LEU:HD23	1.86	0.41
1:B:231:ARG:NH2	5:B:2110:HOH:O	2.54	0.41
1:B:582:LEU:HB3	1:B:621:LEU:HD21	2.03	0.41
1:B:791:LEU:HA	1:B:814:PHE:HE2	1.85	0.41
1:C:164:LYS:O	1:C:168:GLU:OE1	2.39	0.41
1:C:630:THR:O	1:C:634:VAL:CG1	2.63	0.41
1:C:645:GLY:HA3	2:K:10:DC:OP2	2.21	0.41
1:D:204:TRP:HB3	5:D:2057:HOH:O	2.20	0.41
1:D:611:LEU:HB2	1:D:616:LEU:HD21	2.02	0.41
1:D:654:THR:C	1:D:657:PRO:HD2	2.41	0.41
1:D:423:ARG:HE	1:D:781:ASN:HD22	1.67	0.41
1:D:860:LYS:O	1:D:860:LYS:HD3	2.21	0.41
2:H:15:DC:H2”	2:H:16:DC:O5’	2.21	0.41
1:A:497:LEU:HA	1:A:497:LEU:HD23	1.77	0.41
1:A:582:LEU:HD23	1:A:582:LEU:HA	1.89	0.41
1:A:62:GLY:C	1:A:64:VAL:H	2.23	0.41
1:A:120:LYS:CG	1:A:752:LEU:HD21	2.49	0.41
1:A:753:GLY:O	1:A:754:GLN:HG2	2.20	0.41
1:A:775:GLU:HG2	1:A:775:GLU:O	2.20	0.41
1:B:13:ASP:OD1	1:B:291:ARG:NH1	2.51	0.41
1:B:198:GLY:O	1:B:199:GLU:HB2	2.21	0.41
1:B:137:VAL:HG12	1:B:217:ILE:HD11	2.01	0.41
1:B:395:ARG:O	1:B:395:ARG:HG3	2.20	0.41
1:C:787:ASP:C	1:C:787:ASP:OD1	2.58	0.41
1:D:192:SER:C	1:D:193:LYS:HG3	2.42	0.41
1:D:326:THR:HG23	5:D:2087:HOH:O	2.20	0.41
1:D:452:ILE:HD11	1:D:457:TYR:HA	2.02	0.41

Continued on next page...

Continued from previous page...

Atom-1	Atom-2	Interatomic distance (Å)	Clash overlap (Å)
1:D:700:LYS:NZ	1:D:700:LYS:CB	2.83	0.41
1:A:229:LEU:HD11	1:A:242:GLU:CG	2.51	0.41
1:A:267:MET:HE3	2:E:16:DC:OP1	2.21	0.41
1:A:518:TYR:O	1:A:522:GLN:HG2	2.21	0.41
1:A:6:ILE:O	1:A:8:LYS:N	2.42	0.41
1:A:711:LYS:HA	1:A:719:LEU:HD13	2.03	0.41
1:A:73:LEU:HD23	1:A:73:LEU:HA	1.60	0.41
1:A:806:SER:O	1:A:816:THR:HG22	2.19	0.41
1:B:276:LYS:NZ	1:B:283:GLY:O	2.40	0.41
1:B:495:SER:HB2	1:B:498:GLU:HB2	2.03	0.41
1:B:98:LYS:HB3	1:B:98:LYS:HE2	1.78	0.41
1:C:44:TYR:OH	1:C:292:ARG:HB3	2.21	0.41
1:C:6:ILE:O	1:C:8:LYS:N	2.43	0.41
1:D:291:ARG:C	1:D:293:PRO:HD3	2.41	0.41
1:D:312:TYR:CZ	1:D:314:PRO:HG3	2.55	0.41
1:D:341:ILE:HG12	1:D:341:ILE:H	1.73	0.41
1:D:425:ARG:NH1	1:D:784:HIS:CE1	2.89	0.41
1:D:437:ASN:O	1:D:441:LYS:HG3	2.21	0.41
1:D:472:LYS:HD2	1:D:472:LYS:HA	1.96	0.41
1:D:468:ALA:HA	1:D:505:GLN:HB3	2.03	0.41
1:D:60:LYS:HE2	1:D:60:LYS:HB3	1.68	0.41
1:D:8:LYS:HB3	1:D:9:ASN:H	1.49	0.41
1:B:386:ARG:HE	3:I:5:C:P	2.44	0.41
1:A:416:PHE:HA	1:A:417:PRO:HD2	1.99	0.41
1:A:437:ASN:C	1:A:437:ASN:HD22	2.24	0.41
1:B:247:ALA:HA	1:B:248:PRO:HD3	1.92	0.41
1:B:476:PRO:HG2	5:B:2249:HOH:O	2.21	0.41
1:C:664:GLY:HA2	1:C:667:PHE:HD2	1.86	0.41
1:D:563:PRO:HB3	1:D:877:GLU:O	2.21	0.41
1:D:619:GLN:NE2	1:D:666:MET:O	2.54	0.41
1:D:652:GLU:HA	1:D:656:GLN:HB2	2.02	0.41
1:D:70:ALA:O	1:D:74:ILE:HG13	2.21	0.41
1:A:244:ILE:O	1:A:245:GLU:CD	2.59	0.40
1:A:720:ARG:HD3	1:A:854:HIS:HB2	2.02	0.40
1:B:347:CYS:SG	1:B:350:GLU:HG3	2.62	0.40
1:B:353:PRO:HB2	5:B:2164:HOH:O	2.21	0.40
1:B:324:GLN:HG3	1:B:417:PRO:HA	2.01	0.40
1:B:416:PHE:CE1	1:B:432:PHE:O	2.75	0.40
1:C:154:ILE:HA	1:C:158:GLU:HB2	2.02	0.40
1:C:313:MET:CE	1:C:317:TYR:CE2	3.04	0.40
1:C:430:SER:O	1:C:433:ASN:ND2	2.46	0.40

Continued on next page...

Continued from previous page...

Atom-1	Atom-2	Interatomic distance (Å)	Clash overlap (Å)
1:C:120:LYS:HZ2	1:C:752:LEU:HG	1.85	0.40
1:D:352:ILE:CG2	5:D:2101:HOH:O	2.65	0.40
1:D:540:CYS:O	1:D:541:SER:C	2.59	0.40
1:D:840:ASP:O	1:D:842:LEU:N	2.54	0.40
4:M:4:DG:H1'	4:M:5:DA:C8	2.56	0.40
1:A:51:PHE:CE2	1:A:261:LEU:HD23	2.56	0.40
1:A:292:ARG:CG	1:A:292:ARG:O	2.66	0.40
1:A:305:LEU:HA	1:A:305:LEU:HD23	1.85	0.40
1:A:389:LYS:HA	1:A:392:LYS:HE3	2.03	0.40
1:A:419:ASN:HB3	1:A:420:MET:H	1.77	0.40
1:B:292:ARG:N	1:B:293:PRO:HD3	2.37	0.40
1:B:710:VAL:O	1:B:710:VAL:HG13	2.20	0.40
1:B:749:LEU:CD2	5:B:2088:HOH:O	2.64	0.40
1:C:126:LEU:HD11	1:C:227:VAL:HG11	2.04	0.40
1:C:468:ALA:HA	1:C:505:GLN:HB3	2.02	0.40
1:C:53:LYS:O	1:C:56:GLU:HB3	2.21	0.40
1:C:651:LEU:O	1:C:656:GLN:HB2	2.21	0.40
1:D:134:VAL:CG1	5:D:2067:HOH:O	2.69	0.40
1:D:247:ALA:HB3	1:D:250:TYR:HB2	2.03	0.40
1:D:291:ARG:HA	1:D:291:ARG:HD3	1.81	0.40
1:D:551:ARG:HH12	1:D:872:LEU:HD12	1.85	0.40
1:D:93:LYS:NZ	5:D:2032:HOH:O	2.52	0.40
1:A:632:ARG:NH1	5:A:2251:HOH:O	2.55	0.40
1:A:74:ILE:CG2	1:A:755:PHE:HZ	2.34	0.40
1:A:789:SER:HA	1:A:792:ARG:NH2	2.36	0.40
1:D:236:VAL:HB	1:D:239:GLN:HB2	2.04	0.40
1:D:475:PHE:N	1:D:475:PHE:HD1	2.17	0.40
1:D:642:LYS:O	1:D:643:GLU:C	2.56	0.40
1:D:729:THR:H	1:D:729:THR:HG23	1.60	0.40
1:D:849:PHE:O	1:D:850:ALA:C	2.59	0.40
1:A:141:ILE:HG22	5:A:2072:HOH:O	2.21	0.40
1:A:308:TYR:HA	1:A:311:VAL:HG21	2.03	0.40
1:B:158:GLU:CG	1:B:195:LEU:HD22	2.36	0.40
1:B:439:MET:O	1:B:440:THR:C	2.58	0.40
1:C:373:ALA:CB	1:C:377:TRP:HE1	2.31	0.40
1:C:463:HIS:HA	1:C:466:ASN:ND2	2.34	0.40
1:C:632:ARG:CG	5:C:2145:HOH:O	2.69	0.40
1:D:85:ILE:H	1:D:85:ILE:HG13	1.62	0.40
2:E:16:DC:H2"	2:E:17:DG:O5'	2.22	0.40
1:C:57:ARG:NH1	2:K:18:DC:OP2	2.55	0.40
1:A:30:GLU:CG	1:A:34:ARG:NH2	2.84	0.40

Continued on next page...

Continued from previous page...

Atom-1	Atom-2	Interatomic distance (Å)	Clash overlap (Å)
1:A:505:GLN:O	1:A:508:PRO:HD3	2.22	0.40
1:A:59:LEU:O	1:A:61:ALA:N	2.55	0.40
1:A:58:GLN:CG	1:A:67:ASN:HD22	2.34	0.40
1:B:155:ARG:CB	5:B:2088:HOH:O	2.65	0.40
1:B:171:ASN:ND2	1:B:171:ASN:N	2.69	0.40
1:B:737:GLN:O	1:B:774:GLN:NE2	2.55	0.40
1:C:172:LYS:HE3	1:C:172:LYS:HB3	1.78	0.40
1:C:170:LEU:HD22	1:C:179:LYS:HE2	2.04	0.40
1:C:594:VAL:HA	1:C:609:VAL:HA	2.03	0.40
1:C:663:LYS:HE3	1:C:666:MET:HE1	2.02	0.40
1:C:791:LEU:HA	1:C:814:PHE:HE2	1.86	0.40
1:D:109:ILE:H	1:D:109:ILE:CD1	2.27	0.40
1:D:130:ASP:O	1:D:132:THR:HG23	2.22	0.40
1:D:486:HIS:C	1:D:486:HIS:ND1	2.72	0.40
1:D:882:PHE:N	1:D:882:PHE:HD1	2.09	0.40
2:K:9:DA:N6	4:M:1:DG:H21	2.18	0.40

There are no symmetry-related clashes.

5.3 Torsion angles (i)

5.3.1 Protein backbone (i)

In the following table, the Percentiles column shows the percent Ramachandran outliers of the chain as a percentile score with respect to all X-ray entries followed by that with respect to entries of similar resolution.

The Analysed column shows the number of residues for which the backbone conformation was analysed, and the total number of residues.

Mol	Chain	Analysed	Favoured	Allowed	Outliers	Percentiles	
1	A	851/883 (96%)	740 (87%)	94 (11%)	17 (2%)	7	27
1	B	851/883 (96%)	736 (86%)	98 (12%)	17 (2%)	7	27
1	C	851/883 (96%)	737 (87%)	93 (11%)	21 (2%)	5	21
1	D	851/883 (96%)	735 (86%)	96 (11%)	20 (2%)	6	22
All	All	3404/3532 (96%)	2948 (87%)	381 (11%)	75 (2%)	6	24

All (75) Ramachandran outliers are listed below:

Mol	Chain	Res	Type
1	A	194	GLY
1	A	199	GLU
1	A	539	SER
1	A	663	LYS
1	A	755	PHE
1	B	194	GLY
1	B	199	GLU
1	B	539	SER
1	B	755	PHE
1	C	194	GLY
1	C	199	GLU
1	C	539	SER
1	C	755	PHE
1	D	194	GLY
1	D	199	GLU
1	D	539	SER
1	D	755	PHE
1	A	7	ALA
1	A	206	LYS
1	B	7	ALA
1	B	15	GLU
1	B	200	ALA
1	B	663	LYS
1	B	882	PHE
1	C	7	ALA
1	C	15	GLU
1	C	200	ALA
1	C	240	ASP
1	C	663	LYS
1	D	7	ALA
1	D	15	GLU
1	D	200	ALA
1	D	663	LYS
1	D	882	PHE
1	A	15	GLU
1	A	60	LYS
1	A	200	ALA
1	A	841	VAL
1	B	14	ILE
1	B	204	TRP
1	B	872	LEU
1	C	60	LYS
1	C	204	TRP

Continued on next page...

Continued from previous page...

Mol	Chain	Res	Type
1	C	206	LYS
1	C	872	LEU
1	C	882	PHE
1	D	14	ILE
1	D	204	TRP
1	D	240	ASP
1	D	841	VAL
1	A	14	ILE
1	A	240	ASP
1	B	745	THR
1	C	631	LYS
1	C	745	THR
1	C	841	VAL
1	D	872	LEU
1	A	745	THR
1	A	882	PHE
1	C	14	ILE
1	C	508	PRO
1	D	348	PRO
1	D	353	PRO
1	D	745	THR
1	A	353	PRO
1	B	240	ASP
1	B	631	LYS
1	B	841	VAL
1	D	60	LYS
1	D	508	PRO
1	B	4	ILE
1	C	4	ILE
1	C	348	PRO
1	D	4	ILE
1	A	810	ILE

5.3.2 Protein sidechains ⓘ

In the following table, the Percentiles column shows the percent sidechain outliers of the chain as a percentile score with respect to all X-ray entries followed by that with respect to entries of similar resolution.

The Analysed column shows the number of residues for which the sidechain conformation was analysed, and the total number of residues.

Mol	Chain	Analysed	Rotameric	Outliers	Percentiles	
1	A	703/729 (96%)	615 (88%)	88 (12%)	4	14
1	B	703/729 (96%)	619 (88%)	84 (12%)	5	15
1	C	703/729 (96%)	623 (89%)	80 (11%)	5	17
1	D	703/729 (96%)	628 (89%)	75 (11%)	6	20
All	All	2812/2916 (96%)	2485 (88%)	327 (12%)	5	16

All (327) residues with a non-rotameric sidechain are listed below:

Mol	Chain	Res	Type
1	A	5	ASN
1	A	16	LEU
1	A	21	PHE
1	A	32	LEU
1	A	36	GLN
1	A	50	ARG
1	A	54	MET
1	A	56	GLU
1	A	77	LEU
1	A	84	ARG
1	A	96	ARG
1	A	99	ARG
1	A	101	THR
1	A	105	PHE
1	A	118	THR
1	A	119	ILE
1	A	121	THR
1	A	155	ARG
1	A	162	PHE
1	A	176	HIS
1	A	184	GLN
1	A	202	SER
1	A	206	LYS
1	A	228	SER
1	A	230	HIS
1	A	245	GLU
1	A	257	ARG
1	A	274	PRO
1	A	279	THR
1	A	302	LYS
1	A	305	LEU
1	A	335	LEU
1	A	343	LYS

Continued on next page...

Continued from previous page...

Mol	Chain	Res	Type
1	A	377	TRP
1	A	379	ARG
1	A	391	ARG
1	A	399	GLU
1	A	402	LEU
1	A	422	TRP
1	A	423	ARG
1	A	437	ASN
1	A	452	ILE
1	A	454	LYS
1	A	472	LYS
1	A	514	PHE
1	A	517	GLU
1	A	527	SER
1	A	529	ASN
1	A	553	GLU
1	A	559	VAL
1	A	561	LEU
1	A	573	ILE
1	A	577	LYS
1	A	583	GLN
1	A	591	ASP
1	A	601	ASN
1	A	632	ARG
1	A	633	SER
1	A	635	MET
1	A	654	THR
1	A	656	GLN
1	A	666	MET
1	A	669	GLN
1	A	670	PRO
1	A	700	LYS
1	A	701	SER
1	A	704	LYS
1	A	710	VAL
1	A	713	LYS
1	A	714	LYS
1	A	722	ARG
1	A	731	ASP
1	A	735	VAL
1	A	738	GLU
1	A	741	LYS

Continued on next page...

Continued from previous page...

Mol	Chain	Res	Type
1	A	744	GLN
1	A	746	ARG
1	A	766	ASP
1	A	786	GLN
1	A	791	LEU
1	A	814	PHE
1	A	816	THR
1	A	828	VAL
1	A	842	LEU
1	A	852	GLN
1	A	859	ASP
1	A	867	LYS
1	A	882	PHE
1	B	16	LEU
1	B	19	ILE
1	B	21	PHE
1	B	27	HIS
1	B	39	LEU
1	B	46	MET
1	B	50	ARG
1	B	51	PHE
1	B	54	MET
1	B	56	GLU
1	B	84	ARG
1	B	96	ARG
1	B	116	TYR
1	B	143	ARG
1	B	164	LYS
1	B	165	ASN
1	B	168	GLU
1	B	170	LEU
1	B	192	SER
1	B	196	LEU
1	B	206	LYS
1	B	222	GLU
1	B	245	GLU
1	B	257	ARG
1	B	261	LEU
1	B	265	SER
1	B	272	VAL
1	B	274	PRO
1	B	289	ASN

Continued on next page...

Continued from previous page...

Mol	Chain	Res	Type
1	B	292	ARG
1	B	305	LEU
1	B	307	ARG
1	B	333	LYS
1	B	335	LEU
1	B	386	ARG
1	B	388	ASP
1	B	391	ARG
1	B	393	SER
1	B	398	LEU
1	B	412	LYS
1	B	422	TRP
1	B	423	ARG
1	B	437	ASN
1	B	445	THR
1	B	477	GLU
1	B	480	LYS
1	B	495	SER
1	B	514	PHE
1	B	529	ASN
1	B	553	GLU
1	B	561	LEU
1	B	565	GLU
1	B	573	ILE
1	B	601	ASN
1	B	632	ARG
1	B	634	VAL
1	B	651	LEU
1	B	654	THR
1	B	666	MET
1	B	668	THR
1	B	669	GLN
1	B	685	VAL
1	B	714	LYS
1	B	722	ARG
1	B	725	VAL
1	B	734	PRO
1	B	735	VAL
1	B	745	THR
1	B	749	LEU
1	B	751	PHE
1	B	752	LEU

Continued on next page...

Continued from previous page...

Mol	Chain	Res	Type
1	B	769	ILE
1	B	783	VAL
1	B	785	SER
1	B	786	GLN
1	B	787	ASP
1	B	818	PRO
1	B	828	VAL
1	B	829	ARG
1	B	831	THR
1	B	842	LEU
1	B	852	GLN
1	B	859	ASP
1	B	867	LYS
1	C	5	ASN
1	C	15	GLU
1	C	16	LEU
1	C	19	ILE
1	C	32	LEU
1	C	36	GLN
1	C	50	ARG
1	C	56	GLU
1	C	81	MET
1	C	84	ARG
1	C	96	ARG
1	C	120	LYS
1	C	132	THR
1	C	143	ARG
1	C	155	ARG
1	C	162	PHE
1	C	170	LEU
1	C	185	VAL
1	C	206	LYS
1	C	208	ASP
1	C	227	VAL
1	C	230	HIS
1	C	241	SER
1	C	257	ARG
1	C	266	PRO
1	C	279	THR
1	C	291	ARG
1	C	293	PRO
1	C	303	LYS

Continued on next page...

Continued from previous page...

Mol	Chain	Res	Type
1	C	305	LEU
1	C	307	ARG
1	C	313	MET
1	C	350	GLU
1	C	378	LYS
1	C	379	ARG
1	C	397	SER
1	C	412	LYS
1	C	419	ASN
1	C	422	TRP
1	C	423	ARG
1	C	437	ASN
1	C	472	LYS
1	C	473	VAL
1	C	514	PHE
1	C	532	LEU
1	C	551	ARG
1	C	561	LEU
1	C	601	ASN
1	C	632	ARG
1	C	634	VAL
1	C	641	SER
1	C	643	GLU
1	C	652	GLU
1	C	654	THR
1	C	666	MET
1	C	669	GLN
1	C	670	PRO
1	C	694	GLU
1	C	730	PRO
1	C	731	ASP
1	C	734	PRO
1	C	735	VAL
1	C	744	GLN
1	C	749	LEU
1	C	754	GLN
1	C	755	PHE
1	C	768	GLU
1	C	783	VAL
1	C	787	ASP
1	C	801	LYS
1	C	814	PHE

Continued on next page...

Continued from previous page...

Mol	Chain	Res	Type
1	C	816	THR
1	C	823	ASN
1	C	838	SER
1	C	855	GLU
1	C	859	ASP
1	C	860	LYS
1	C	869	ASN
1	C	870	LEU
1	C	882	PHE
1	D	5	ASN
1	D	16	LEU
1	D	19	ILE
1	D	27	HIS
1	D	48	GLU
1	D	50	ARG
1	D	52	ARG
1	D	54	MET
1	D	56	GLU
1	D	77	LEU
1	D	84	ARG
1	D	120	LYS
1	D	135	GLN
1	D	143	ARG
1	D	171	ASN
1	D	206	LYS
1	D	230	HIS
1	D	257	ARG
1	D	274	PRO
1	D	279	THR
1	D	281	ILE
1	D	294	LEU
1	D	305	LEU
1	D	315	GLU
1	D	335	LEU
1	D	343	LYS
1	D	346	HIS
1	D	351	ASP
1	D	388	ASP
1	D	391	ARG
1	D	393	SER
1	D	402	LEU
1	D	404	GLN

Continued on next page...

Continued from previous page...

Mol	Chain	Res	Type
1	D	422	TRP
1	D	423	ARG
1	D	437	ASN
1	D	472	LYS
1	D	475	PHE
1	D	495	SER
1	D	497	LEU
1	D	539	SER
1	D	553	GLU
1	D	561	LEU
1	D	573	ILE
1	D	577	LYS
1	D	585	ASP
1	D	601	ASN
1	D	632	ARG
1	D	666	MET
1	D	685	VAL
1	D	689	VAL
1	D	701	SER
1	D	710	VAL
1	D	714	LYS
1	D	719	LEU
1	D	722	ARG
1	D	730	PRO
1	D	735	VAL
1	D	746	ARG
1	D	750	MET
1	D	751	PHE
1	D	752	LEU
1	D	783	VAL
1	D	787	ASP
1	D	801	LYS
1	D	816	THR
1	D	824	LEU
1	D	829	ARG
1	D	831	THR
1	D	834	ASP
1	D	842	LEU
1	D	855	GLU
1	D	869	ASN
1	D	870	LEU
1	D	882	PHE

Some sidechains can be flipped to improve hydrogen bonding and reduce clashes. All (130) such sidechains are listed below:

Mol	Chain	Res	Type
1	A	22	ASN
1	A	107	GLN
1	A	131	ASN
1	A	171	ASN
1	A	184	GLN
1	A	232	GLN
1	A	239	GLN
1	A	269	GLN
1	A	289	ASN
1	A	324	GLN
1	A	410	ASN
1	A	419	ASN
1	A	435	GLN
1	A	437	ASN
1	A	463	HIS
1	A	485	ASN
1	A	486	HIS
1	A	529	ASN
1	A	544	GLN
1	A	568	GLN
1	A	583	GLN
1	A	588	ASN
1	A	669	GLN
1	A	672	GLN
1	A	726	HIS
1	A	737	GLN
1	A	781	ASN
1	A	786	GLN
1	A	811	HIS
1	A	823	ASN
1	A	852	GLN
1	A	854	HIS
1	A	869	ASN
1	A	871	ASN
1	B	161	HIS
1	B	171	ASN
1	B	211	HIS
1	B	239	GLN
1	B	324	GLN
1	B	404	GLN
1	B	411	HIS

Continued on next page...

Continued from previous page...

Mol	Chain	Res	Type
1	B	419	ASN
1	B	435	GLN
1	B	437	ASN
1	B	463	HIS
1	B	485	ASN
1	B	499	ASN
1	B	529	ASN
1	B	619	GLN
1	B	656	GLN
1	B	671	ASN
1	B	672	GLN
1	B	726	HIS
1	B	737	GLN
1	B	744	GLN
1	B	748	ASN
1	B	754	GLN
1	B	781	ASN
1	B	786	GLN
1	B	811	HIS
1	B	823	ASN
1	B	852	GLN
1	B	854	HIS
1	C	5	ASN
1	C	86	ASN
1	C	107	GLN
1	C	171	ASN
1	C	211	HIS
1	C	230	HIS
1	C	233	ASN
1	C	239	GLN
1	C	269	GLN
1	C	289	ASN
1	C	324	GLN
1	C	404	GLN
1	C	410	ASN
1	C	419	ASN
1	C	435	GLN
1	C	437	ASN
1	C	466	ASN
1	C	485	ASN
1	C	486	HIS
1	C	499	ASN

Continued on next page...

Continued from previous page...

Mol	Chain	Res	Type
1	C	545	HIS
1	C	588	ASN
1	C	726	HIS
1	C	737	GLN
1	C	744	GLN
1	C	748	ASN
1	C	754	GLN
1	C	781	ASN
1	C	786	GLN
1	C	823	ASN
1	C	848	GLN
1	C	854	HIS
1	C	869	ASN
1	C	871	ASN
1	D	86	ASN
1	D	171	ASN
1	D	184	GLN
1	D	232	GLN
1	D	239	GLN
1	D	289	ASN
1	D	324	GLN
1	D	339	ASN
1	D	346	HIS
1	D	406	ASN
1	D	410	ASN
1	D	419	ASN
1	D	435	GLN
1	D	437	ASN
1	D	463	HIS
1	D	485	ASN
1	D	522	GLN
1	D	544	GLN
1	D	588	ASN
1	D	649	GLN
1	D	671	ASN
1	D	726	HIS
1	D	737	GLN
1	D	744	GLN
1	D	772	HIS
1	D	774	GLN
1	D	781	ASN
1	D	786	GLN

Continued on next page...

Continued from previous page...

Mol	Chain	Res	Type
1	D	823	ASN
1	D	852	GLN
1	D	854	HIS
1	D	869	ASN
1	D	871	ASN

5.3.3 RNA (i)

Mol	Chain	Analysed	Backbone Outliers	Pucker Outliers
3	F	7/12 (58%)	0	0
3	I	7/12 (58%)	0	0
3	L	7/12 (58%)	0	0
3	O	7/12 (58%)	0	0
All	All	28/48 (58%)	0	0

There are no RNA backbone outliers to report.

There are no RNA pucker outliers to report.

5.4 Non-standard residues in protein, DNA, RNA chains (i)

There are no non-standard protein/DNA/RNA residues in this entry.

5.5 Carbohydrates (i)

There are no carbohydrates in this entry.

5.6 Ligand geometry (i)

There are no ligands in this entry.

5.7 Other polymers (i)

There are no such residues in this entry.

5.8 Polymer linkage issues (i)

There are no chain breaks in this entry.

6 Fit of model and data (i)

6.1 Protein, DNA and RNA chains (i)

EDS was not executed - this section is therefore empty.

6.2 Non-standard residues in protein, DNA, RNA chains (i)

EDS was not executed - this section is therefore empty.

6.3 Carbohydrates (i)

EDS was not executed - this section is therefore empty.

6.4 Ligands (i)

EDS was not executed - this section is therefore empty.

6.5 Other polymers (i)

EDS was not executed - this section is therefore empty.
