# Supplementary material for: An engineered T7 RNA polymerase that produces mRNA free of immunostimulatory byproducts
Source: Nat Biotechnol. 2022 Nov 10;41(4):560–8. doi: 10.1038/s41587-022-01525-6 (PMC10110463; doi:10.1038/s41587-022-01525-6)
Supplement: Supplementary file 5 — Full validation report for PDB entry 1MSW. [file 41587_2022_1525_MOESM5_ESM.pdf]

# Full wwPDB X-ray Structure Validation Report ⓘ

May 27, 2020 – 01:26 am BST

| | | |
|---|---|---|
| PDB ID | : | 1MSW |
| Title | : | Structural basis for the transition from initiation to elongation transcription in T7 RNA polymerase |
| Authors | : | Yin, Y.W.; Steitz, T.A. |
| Deposited on | : | 2002-09-19 |
| Resolution | : | 2.10 Å(reported) |

This is a Full wwPDB X-ray Structure Validation Report for a publicly released PDB entry.

We welcome your comments at *validation@mail.wwpdb.org*
A user guide is available at
https://www.wwpdb.org/validation/2017/XrayValidationReportHelp
with specific help available everywhere you see the ⓘ symbol.

The following versions of software and data (see references ⓘ) were used in the production of this report:

| | | |
|---|---|---|
| MolProbity | : | 4.02b-467 |
| Xtriage (Phenix) | : | NOT EXECUTED |
| EDS | : | NOT EXECUTED |
| Percentile statistics | : | 20191225.v01 (using entries in the PDB archive December 25th 2019) |
| Ideal geometry (proteins) | : | Engh & Huber (2001) |
| Ideal geometry (DNA, RNA) | : | Parkinson et al. (1996) |
| Validation Pipeline (wwPDB-VP) | : | 2.11 |

# 1 Overall quality at a glance (i)

The following experimental techniques were used to determine the structure:
*X-RAY DIFFRACTION*

The reported resolution of this entry is 2.10 Å.

Percentile scores (ranging between 0-100) for global validation metrics of the entry are shown in the following graphic. The table shows the number of entries on which the scores are based.

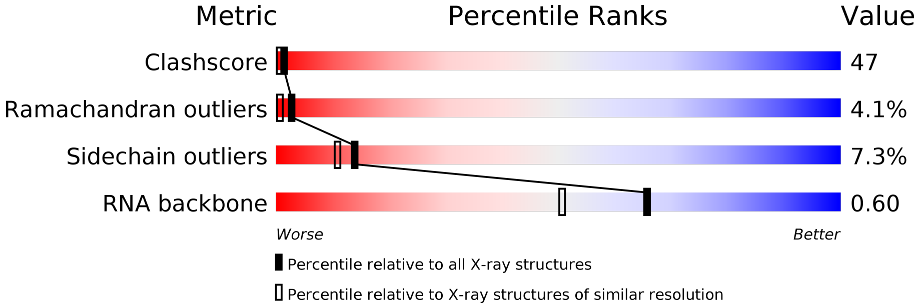


| Metric | Whole archive (#Entries) | Similar resolution (#Entries, resolution range(Å)) |
|---|---|---|
| Clashscore | 141614 | 5710 (2.10-2.10) |
| Ramachandran outliers | 138981 | 5647 (2.10-2.10) |
| Sidechain outliers | 138945 | 5648 (2.10-2.10) |
| RNA backbone | 3102 | 1000 (2.54-1.66) |

The table below summarises the geometric issues observed across the polymeric chains and their fit to the electron density. The red, orange, yellow and green segments on the lower bar indicate the fraction of residues that contain outliers for >=3, 2, 1 and 0 types of geometric quality criteria respectively. A grey segment represents the fraction of residues that are not modelled. The numeric value for each fraction is indicated below the corresponding segment, with a dot representing fractions <=5%

Note EDS was not executed.

| Mol | Chain | Length | Quality of chain |
|---|---|---|---|
| 1 | T | 20 | 5% 60% 30% 5% |
| 2 | N | 17 | 18% 82% |
| 3 | R | 10 | 20% 80% |
| 4 | D | 883 | 44% 46% 7% • |

# 2 Entry composition (i)

There are 5 unique types of molecules in this entry. The entry contains 7956 atoms, of which 0 are hydrogens and 0 are deuteriums.

In the tables below, the ZeroOcc column contains the number of atoms modelled with zero occupancy, the AltConf column contains the number of residues with at least one atom in alternate conformation and the Trace column contains the number of residues modelled with at most 2 atoms.

- Molecule 1 is a DNA chain called Template DNA.

| Mol | Chain | Residues | Atoms | | | | | ZeroOcc | AltConf | Trace |
|---|---|---|---|---|---|---|---|---|---|---|
| | | | Total | C | N | O | P | | | |
| 1 | T | 20 | 405 | 193 | 71 | 122 | 19 | 0 | 0 | 0 |

- Molecule 2 is a DNA chain called Non-Template DNA.

| Mol | Chain | Residues | Atoms | | | | | ZeroOcc | AltConf | Trace |
|---|---|---|---|---|---|---|---|---|---|---|
| | | | Total | C | N | O | P | | | |
| 2 | N | 17 | 344 | 165 | 60 | 103 | 16 | 0 | 0 | 0 |

- Molecule 3 is a RNA chain called RNA message.

| Mol | Chain | Residues | Atoms | | | | | ZeroOcc | AltConf | Trace |
|---|---|---|---|---|---|---|---|---|---|---|
| | | | Total | C | N | O | P | | | |
| 3 | R | 10 | 215 | 97 | 44 | 65 | 9 | 0 | 0 | 0 |

- Molecule 4 is a protein called DNA-directed RNA polymerase.

| Mol | Chain | Residues | Atoms | | | | | ZeroOcc | AltConf | Trace |
|---|---|---|---|---|---|---|---|---|---|---|
| | | | Total | C | N | O | S | | | |
| 4 | D | 863 | 6802 | 4333 | 1182 | 1251 | 36 | 0 | 0 | 0 |

- Molecule 5 is water.

| Mol | Chain | Residues | Atoms | | ZeroOcc | AltConf |
|---|---|---|---|---|---|---|
| | | | Total | O | | |
| 5 | T | 13 | 13 | 13 | 0 | 0 |
| 5 | N | 2 | 2 | 2 | 0 | 0 |
| 5 | R | 9 | 9 | 9 | 0 | 0 |
| 5 | D | 166 | 166 | 166 | 0 | 0 |

# 3 Residue-property plots (i)

These plots are drawn for all protein, RNA and DNA chains in the entry. The first graphic for a chain summarises the proportions of the various outlier classes displayed in the second graphic. The second graphic shows the sequence view annotated by issues in geometry. Residues are color-coded according to the number of geometric quality criteria for which they contain at least one outlier: green = 0, yellow = 1, orange = 2 and red = 3 or more. Stretches of 2 or more consecutive residues without any outlier are shown as a green connector. Residues present in the sample, but not in the model, are shown in grey.

Note EDS was not executed.

- Molecule 1: Template DNA

Chain T:

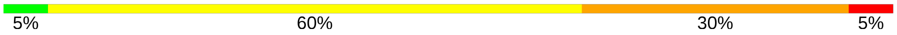


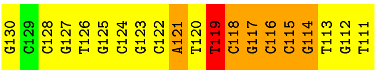


- Molecule 2: Non-Template DNA

Chain N:

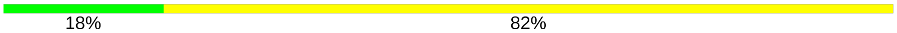


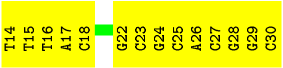


- Molecule 3: RNA message

Chain R:

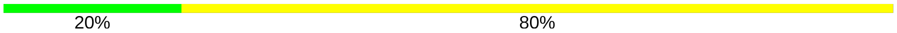


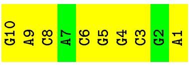


- Molecule 4: DNA-directed RNA polymerase

Chain D:

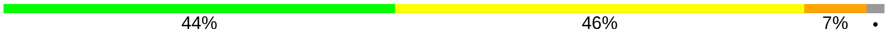


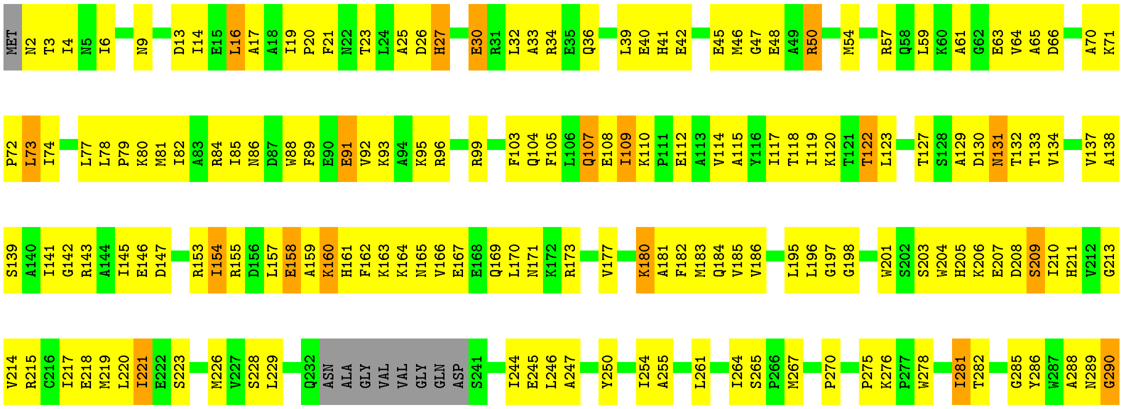

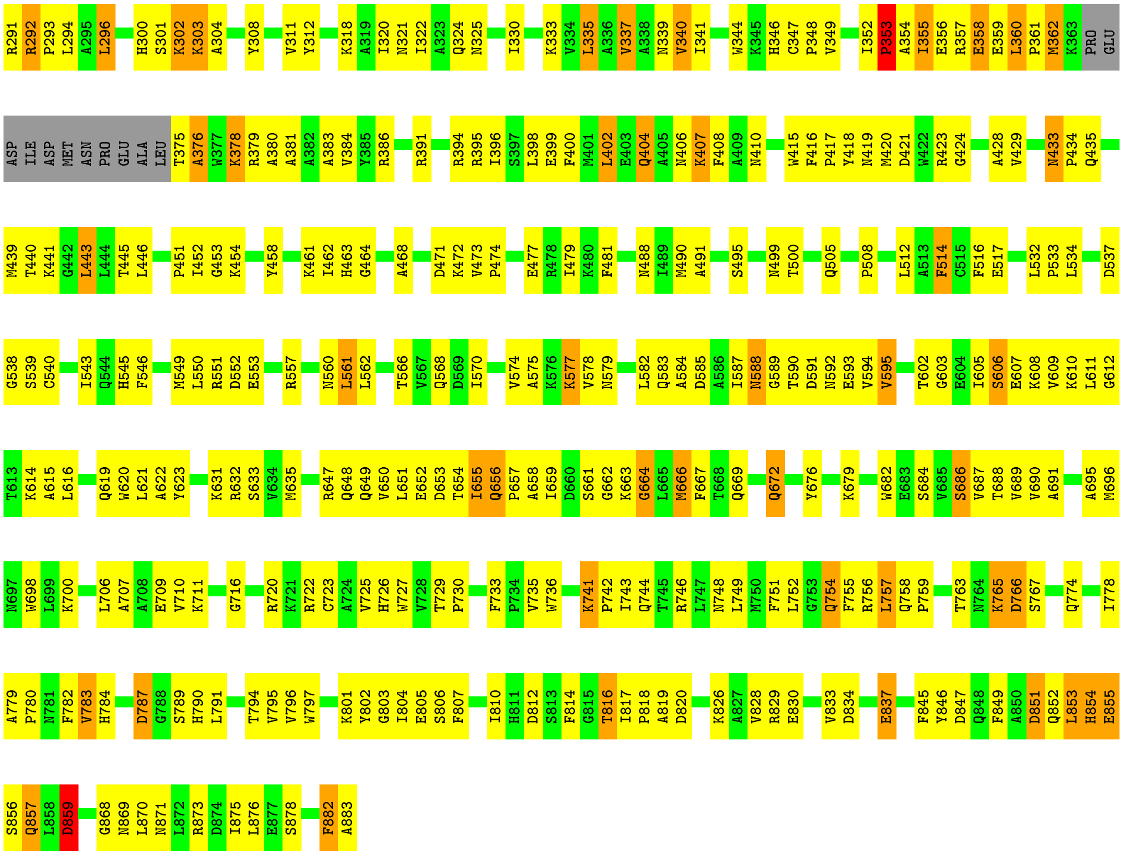
R291 R292 P293 L294 A295 L296 H300 S301 K302 K303 A304 Y308 V311 Y312 K318 A319 I320 N321 I322 A323 Q324 N325 I330 K333 V334 L335 A336 V337 A338 N339 V340 I341 W344 K345 H346 C347 P348 V349 I352 P353 A354 I355 E356 R357 E358 E359 L360 P361 M362 K363 PRO GLU
ASP ILE ASP MET ASN PRO GLU ALA LEU T375 A376 W377 K378 R379 A380 A381 A382 A383 V384 Y385 R386 R391 R394 R395 I396 S397 L398 E399 F400 M401 L402 E403 Q404 A405 N406 K407 F408 A409 N410 W415 F416 P417 Y418 N419 M420 D421 W422 R423 G424 A428 V429 N433 P434 Q435
M439 T440 K441 G442 L443 L444 T445 L446 P451 I452 G453 K454 Y458 K461 I462 H463 G464 A468 D471 K472 V473 P474 E477 R478 I479 K480 F481 N488 I489 M490 A491 S495 N499 T500 Q505 P508 L512 A513 F514 C515 F516 E517 L532 P533 L534 D537
G538 S539 C540 I543 Q544 H545 F546 M549 L550 R551 D552 E553 R557 N560 L561 L562 T566 V567 Q568 D569 I570 V574 A575 K576 K577 V578 N579 L582 Q583 A584 D585 A586 I587 N588 G589 T590 D591 N592 E593 V594 V595 T602 G603 E604 I605 S606 E607 K608 V609 K610 L611 G612
T613 K614 A615 L616 Q619 W620 L621 A622 Y623 K631 R632 S633 V634 M635 R647 Q648 Q649 V650 L651 E652 D653 T654 I655 Q656 P657 A658 I659 D660 S661 G662 K663 G664 L665 M666 F667 T668 Q669 Q672 Y676 K679 W682 E683 S684 V685 S686 V687 T688 V689 V690 A691 A695 M696
N697 W698 L699 K700 L706 A707 A708 E709 V710 K711 G716 R720 K721 R722 C723 A724 V725 H726 W727 V728 T729 P730 F733 P734 V735 W736 K741 P742 I743 Q744 T745 R746 L747 N748 L749 M750 F751 L752 G753 Q754 F755 R756 L757 Q758 P759 T763 N764 K765 D766 S767 Q774 I778
A779 P780 N781 F782 V783 H784 D787 G788 S789 H790 L791 T794 V795 V796 W797 K801 Y802 G803 I804 E805 S806 F807 I810 H811 D812 S813 F814 G815 T816 I817 P818 A819 D820 K826 A827 V828 R829 E830 V833 D834 E837 F845 Y846 D847 Q848 F849 A850 D851 Q852 L853 H854 E855
S856 Q857 L858 D859 G868 N869 L870 N871 L872 R873 D874 I875 L876 E877 S878 F882 A883

# 4 Data and refinement statistics (i)

Xtriage (Phenix) and EDS were not executed - this section is therefore incomplete.

| Property | Value | Source |
|---|---|---|
| Space group | C 2 2 21 | Depositor |
| Cell constants<br>a, b, c, $\alpha$, $\beta$, $\gamma$ | 142.91Å 145.46Å 145.60Å<br>90.00° 90.00° 90.00° | Depositor |
| Resolution (Å) | 20.00 – 2.10 | Depositor |
| % Data completeness<br>(in resolution range) | 85.0 (20.00-2.10) | Depositor |
| $R_{merge}$ | (Not available) | Depositor |
| $R_{sym}$ | (Not available) | Depositor |
| Refinement program | CNS | Depositor |
| R, $R_{free}$ | 0.241 , 0.275 | Depositor |
| Estimated twinning fraction | No twinning to report. | Xtriage |
| Total number of atoms | 7956 | wwPDB-VP |
| Average B, all atoms (Å$^2$) | 75.0 | wwPDB-VP |

# 5 Model quality (i)

## 5.1 Standard geometry (i)

The Z score for a bond length (or angle) is the number of standard deviations the observed value is removed from the expected value. A bond length (or angle) with $|Z| > 5$ is considered an outlier worth inspection. RMSZ is the root-mean-square of all Z scores of the bond lengths (or angles).

| Mol | Chain | Bond lengths | | Bond angles | |
|---|---|---|---|---|---|
| | | RMSZ | #$|Z|$ >5 | RMSZ | #$|Z|$ >5 |
| 1 | T | 0.46 | 0/452 | 0.90 | 2/696 (0.3%) |
| 2 | N | 0.31 | 0/384 | 0.67 | 0/591 |
| 3 | R | 0.47 | 0/241 | 0.80 | 0/375 |
| 4 | D | 0.48 | 1/6955 (0.0%) | 0.65 | 0/9407 |
| All | All | 0.47 | 1/8032 (0.0%) | 0.67 | 2/11069 (0.0%) |

Chiral center outliers are detected by calculating the chiral volume of a chiral center and verifying if the center is modelled as a planar moiety or with the opposite hand.A planarity outlier is detected by checking planarity of atoms in a peptide group, atoms in a mainchain group or atoms of a sidechain that are expected to be planar.

| Mol | Chain | #Chirality outliers | #Planarity outliers |
|---|---|---|---|
| 1 | T | 0 | 7 |

All (1) bond length outliers are listed below:

| Mol | Chain | Res | Type | Atoms | Z | Observed(Å) | Ideal(Å) |
|---|---|---|---|---|---|---|---|
| 4 | D | 407 | LYS | CD-CE | 5.13 | 1.64 | 1.51 |

All (2) bond angle outliers are listed below:

| Mol | Chain | Res | Type | Atoms | Z | Observed($^o$) | Ideal($^o$) |
|---|---|---|---|---|---|---|---|
| 1 | T | 119 | DT | N1-C1'-C2' | -5.28 | 102.57 | 112.60 |
| 1 | T | 119 | DT | C5'-C4'-C3' | -5.10 | 104.92 | 114.10 |

There are no chirality outliers.

All (7) planarity outliers are listed below:

| Mol | Chain | Res | Type | Group |
|---|---|---|---|---|
| 1 | T | 114 | DG | Sidechain |
| 1 | T | 115 | DC | Sidechain |
| 1 | T | 116 | DC | Sidechain |

*Continued on next page...*

*Continued from previous page...*

| Mol | Chain | Res | Type | Group |
|---|---|---|---|---|
| 1 | T | 117 | DG | Sidechain |
| 1 | T | 118 | DC | Sidechain |
| 1 | T | 119 | DT | Sidechain |
| 1 | T | 121 | DA | Sidechain |

## 5.2 Too-close contacts (i)

In the following table, the Non-H and H(model) columns list the number of non-hydrogen atoms and hydrogen atoms in the chain respectively. The H(added) column lists the number of hydrogen atoms added and optimized by MolProbity. The Clashes column lists the number of clashes within the asymmetric unit, whereas Symm-Clashes lists symmetry related clashes.

| Mol | Chain | Non-H | H(model) | H(added) | Clashes | Symm-Clashes |
|---|---|---|---|---|---|---|
| 1 | T | 405 | 0 | 227 | 49 | 0 |
| 2 | N | 344 | 0 | 194 | 17 | 0 |
| 3 | R | 215 | 0 | 112 | 15 | 0 |
| 4 | D | 6802 | 0 | 6770 | 645 | 0 |
| 5 | D | 166 | 0 | 0 | 246 | 0 |
| 5 | N | 2 | 0 | 0 | 0 | 0 |
| 5 | R | 9 | 0 | 0 | 8 | 0 |
| 5 | T | 13 | 0 | 0 | 27 | 0 |
| All | All | 7956 | 0 | 7303 | 715 | 0 |

The all-atom clashscore is defined as the number of clashes found per 1000 atoms (including hydrogen atoms). The all-atom clashscore for this structure is 47.

All (715) close contacts within the same asymmetric unit are listed below, sorted by their clash magnitude.

| Atom-1 | Atom-2 | Interatomic distance (Å) | Clash overlap (Å) |
|---|---|---|---|
| 4:D:360:LEU:HB2 | 5:D:968:HOH:O | 1.29 | 1.29 |
| 4:D:104:GLN:HB3 | 5:D:979:HOH:O | 1.23 | 1.27 |
| 4:D:276:LYS:HE2 | 5:D:1032:HOH:O | 1.22 | 1.26 |
| 4:D:217:ILE:O | 4:D:221:ILE:HD13 | 1.24 | 1.24 |
| 4:D:428:ALA:HA | 5:D:944:HOH:O | 1.39 | 1.22 |
| 3:R:5:G:H3' | 5:R:99:HOH:O | 1.43 | 1.18 |
| 4:D:383:ALA:HB3 | 5:D:986:HOH:O | 1.42 | 1.18 |
| 4:D:648:GLN:HA | 5:D:1028:HOH:O | 1.44 | 1.17 |
| 4:D:647:ARG:HG2 | 5:D:958:HOH:O | 1.42 | 1.17 |
| 4:D:647:ARG:HD3 | 5:D:913:HOH:O | 1.44 | 1.15 |
| 1:T:111:DT:H3' | 5:T:132:HOH:O | 1.50 | 1.12 |

*Continued on next page...*

*Continued from previous page...*

| Atom-1 | Atom-2 | Interatomic distance (Å) | Clash overlap (Å) |
|---|---|---|---|
| 4:D:360:LEU:HD23 | 5:D:917:HOH:O | 1.49 | 1.11 |
| 4:D:386:ARG:HG3 | 5:D:933:HOH:O | 1.52 | 1.08 |
| 4:D:285:GLY:HA2 | 5:D:928:HOH:O | 1.54 | 1.07 |
| 4:D:312:TYR:HB3 | 5:D:976:HOH:O | 1.51 | 1.06 |
| 4:D:735:VAL:HG12 | 5:D:1012:HOH:O | 1.53 | 1.05 |
| 1:T:119:DT:H71 | 5:T:141:HOH:O | 1.55 | 1.05 |
| 1:T:111:DT:C3' | 5:T:132:HOH:O | 2.02 | 1.04 |
| 4:D:794:THR:HB | 5:D:892:HOH:O | 1.55 | 1.04 |
| 4:D:330:ILE:HD13 | 5:D:895:HOH:O | 1.54 | 1.04 |
| 4:D:817:ILE:HD11 | 5:D:947:HOH:O | 1.55 | 1.03 |
| 4:D:141:ILE:O | 4:D:145:ILE:HD13 | 1.57 | 1.03 |
| 4:D:344:TRP:CB | 5:D:1015:HOH:O | 2.06 | 1.02 |
| 4:D:96:ARG:HG3 | 5:D:891:HOH:O | 1.60 | 1.01 |
| 4:D:451:PRO:HD3 | 5:D:1001:HOH:O | 1.59 | 1.01 |
| 4:D:445:THR:HA | 5:D:974:HOH:O | 1.60 | 1.00 |
| 4:D:4:ILE:HG21 | 5:D:942:HOH:O | 1.60 | 1.00 |
| 4:D:733:PHE:CZ | 5:D:1002:HOH:O | 2.15 | 1.00 |
| 4:D:344:TRP:HB2 | 5:D:1015:HOH:O | 1.63 | 0.97 |
| 4:D:344:TRP:O | 4:D:355:ILE:HD11 | 1.62 | 0.97 |
| 4:D:177:VAL:O | 4:D:180:LYS:HG3 | 1.64 | 0.97 |
| 4:D:296:LEU:HD21 | 4:D:320:ILE:HD13 | 1.45 | 0.97 |
| 4:D:664:GLY:HA2 | 5:D:1031:HOH:O | 1.66 | 0.95 |
| 4:D:65:ALA:HA | 5:D:1017:HOH:O | 1.65 | 0.94 |
| 4:D:118:THR:O | 4:D:122:THR:HG23 | 1.67 | 0.94 |
| 4:D:664:GLY:CA | 5:D:1031:HOH:O | 2.14 | 0.94 |
| 4:D:270:PRO:HD3 | 5:D:935:HOH:O | 1.69 | 0.93 |
| 4:D:796:VAL:HB | 5:D:1016:HOH:O | 1.67 | 0.93 |
| 4:D:795:VAL:N | 5:D:892:HOH:O | 2.00 | 0.93 |
| 4:D:816:THR:CG2 | 5:D:989:HOH:O | 2.18 | 0.92 |
| 4:D:132:THR:CG2 | 5:D:983:HOH:O | 2.17 | 0.92 |
| 4:D:464:GLY:HA3 | 5:D:1036:HOH:O | 1.69 | 0.91 |
| 4:D:289:ASN:C | 5:D:960:HOH:O | 2.07 | 0.91 |
| 4:D:74:ILE:HG13 | 5:D:1017:HOH:O | 1.70 | 0.90 |
| 4:D:355:ILE:HD13 | 4:D:355:ILE:H | 1.35 | 0.90 |
| 3:R:10:G:N3 | 5:R:46:HOH:O | 2.05 | 0.90 |
| 3:R:9:A:H1' | 4:D:756:ARG:NH2 | 1.87 | 0.90 |
| 4:D:433:ASN:HD22 | 4:D:435:GLN:H | 1.15 | 0.90 |
| 4:D:746:ARG:HE | 4:D:754:GLN:HE22 | 1.14 | 0.89 |
| 4:D:816:THR:HG22 | 5:D:989:HOH:O | 1.72 | 0.89 |
| 4:D:302:LYS:O | 4:D:304:ALA:N | 2.04 | 0.89 |
| 4:D:378:LYS:HD2 | 4:D:379:ARG:N | 1.88 | 0.88 |

*Continued on next page...*

*Continued from previous page...*

| Atom-1 | Atom-2 | Interatomic distance (Å) | Clash overlap (Å) |
|---|---|---|---|
| 4:D:830:GLU:HB2 | 5:D:910:HOH:O | 1.71 | 0.88 |
| 4:D:270:PRO:CG | 5:D:935:HOH:O | 2.21 | 0.88 |
| 4:D:137:VAL:HG21 | 4:D:244:ILE:HD11 | 1.54 | 0.88 |
| 4:D:491:ALA:HB1 | 4:D:499:ASN:ND2 | 1.90 | 0.87 |
| 4:D:419:ASN:C | 5:D:957:HOH:O | 2.10 | 0.87 |
| 3:R:10:G:C2 | 5:R:46:HOH:O | 2.26 | 0.87 |
| 4:D:217:ILE:N | 5:D:1005:HOH:O | 2.08 | 0.87 |
| 4:D:546:PHE:HB3 | 5:D:909:HOH:O | 1.74 | 0.86 |
| 4:D:118:THR:CA | 5:D:955:HOH:O | 2.23 | 0.86 |
| 4:D:468:ALA:HB2 | 5:D:987:HOH:O | 1.74 | 0.86 |
| 4:D:118:THR:HA | 5:D:955:HOH:O | 1.74 | 0.85 |
| 4:D:129:ALA:HB3 | 5:D:1045:HOH:O | 1.75 | 0.85 |
| 4:D:292:ARG:HA | 4:D:292:ARG:HE | 1.40 | 0.85 |
| 4:D:720:ARG:CZ | 5:D:1029:HOH:O | 2.22 | 0.85 |
| 4:D:359:GLU:HA | 5:D:917:HOH:O | 1.75 | 0.85 |
| 3:R:9:A:H1' | 4:D:756:ARG:HH21 | 1.42 | 0.85 |
| 4:D:118:THR:N | 5:D:955:HOH:O | 2.10 | 0.84 |
| 4:D:441:LYS:N | 5:D:967:HOH:O | 2.11 | 0.84 |
| 4:D:876:LEU:HD12 | 5:D:1018:HOH:O | 1.78 | 0.83 |
| 3:R:3:C:H5' | 5:R:91:HOH:O | 1.76 | 0.83 |
| 4:D:360:LEU:N | 5:D:917:HOH:O | 2.02 | 0.83 |
| 4:D:3:THR:HA | 5:D:985:HOH:O | 1.78 | 0.83 |
| 4:D:361:PRO:HD2 | 5:D:968:HOH:O | 1.78 | 0.83 |
| 4:D:441:LYS:HG3 | 5:D:967:HOH:O | 1.80 | 0.82 |
| 1:T:126:DT:H2" | 1:T:125:DG:H5' | 1.58 | 0.82 |
| 4:D:780:PRO:CD | 5:D:1010:HOH:O | 2.26 | 0.82 |
| 4:D:59:LEU:HD12 | 4:D:64:VAL:HG22 | 1.60 | 0.82 |
| 4:D:875:ILE:HG13 | 5:D:1014:HOH:O | 1.80 | 0.82 |
| 4:D:270:PRO:CD | 5:D:935:HOH:O | 2.26 | 0.81 |
| 4:D:428:ALA:CB | 5:D:944:HOH:O | 2.24 | 0.81 |
| 4:D:359:GLU:CA | 5:D:917:HOH:O | 2.27 | 0.81 |
| 4:D:281:ILE:HG12 | 4:D:282:THR:HG23 | 1.62 | 0.81 |
| 4:D:632:ARG:HB3 | 5:D:916:HOH:O | 1.80 | 0.81 |
| 4:D:873:ARG:HD3 | 5:D:1018:HOH:O | 1.79 | 0.81 |
| 4:D:361:PRO:CD | 5:D:968:HOH:O | 2.29 | 0.81 |
| 4:D:741:LYS:HZ2 | 4:D:742:PRO:HD3 | 1.45 | 0.81 |
| 4:D:270:PRO:HG3 | 5:D:935:HOH:O | 1.81 | 0.81 |
| 4:D:733:PHE:HZ | 5:D:1002:HOH:O | 1.53 | 0.80 |
| 4:D:780:PRO:HG3 | 5:D:1010:HOH:O | 1.81 | 0.80 |
| 1:T:121:DA:H1' | 5:T:135:HOH:O | 1.81 | 0.80 |
| 4:D:473:VAL:HG22 | 4:D:474:PRO:HD2 | 1.63 | 0.80 |

*Continued on next page...*

*Continued from previous page...*

| Atom-1 | Atom-2 | Interatomic distance (Å) | Clash overlap (Å) |
|---|---|---|---|
| 4:D:276:LYS:HG2 | 5:D:1032:HOH:O | 1.80 | 0.80 |
| 4:D:856:SER:HB3 | 5:D:884:HOH:O | 1.82 | 0.80 |
| 4:D:157:LEU:HG | 4:D:158:GLU:HG2 | 1.63 | 0.80 |
| 4:D:115:ALA:O | 4:D:119:ILE:HG13 | 1.82 | 0.80 |
| 4:D:780:PRO:CG | 5:D:1010:HOH:O | 2.31 | 0.79 |
| 4:D:741:LYS:HZ2 | 4:D:741:LYS:HA | 1.47 | 0.79 |
| 4:D:790:HIS:CD2 | 5:D:995:HOH:O | 2.36 | 0.79 |
| 4:D:505:GLN:OE1 | 5:D:1046:HOH:O | 2.00 | 0.78 |
| 4:D:561:LEU:O | 5:D:1014:HOH:O | 2.01 | 0.78 |
| 1:T:128:DC:H1’ | 1:T:127:DG:N7 | 1.97 | 0.78 |
| 4:D:361:PRO:N | 5:D:968:HOH:O | 2.17 | 0.78 |
| 4:D:119:ILE:O | 4:D:123:LEU:HB2 | 1.85 | 0.77 |
| 4:D:756:ARG:HH11 | 4:D:756:ARG:HG3 | 1.48 | 0.77 |
| 3:R:5:G:C8 | 5:R:99:HOH:O | 2.37 | 0.77 |
| 4:D:214:VAL:O | 4:D:218:GLU:HG3 | 1.85 | 0.77 |
| 4:D:716:GLY:O | 5:D:1013:HOH:O | 2.02 | 0.77 |
| 4:D:454:LYS:HE3 | 5:D:948:HOH:O | 1.85 | 0.76 |
| 1:T:121:DA:C1’ | 5:T:135:HOH:O | 2.33 | 0.76 |
| 4:D:433:ASN:ND2 | 4:D:435:GLN:H | 1.84 | 0.76 |
| 4:D:817:ILE:CD1 | 5:D:947:HOH:O | 2.23 | 0.76 |
| 1:T:127:DG:H1’ | 1:T:126:DT:H5’ | 1.66 | 0.76 |
| 4:D:255:ALA:HA | 5:D:942:HOH:O | 1.84 | 0.76 |
| 4:D:514:PHE:CE2 | 5:D:1036:HOH:O | 2.39 | 0.76 |
| 4:D:137:VAL:O | 4:D:141:ILE:HD12 | 1.85 | 0.76 |
| 4:D:289:ASN:CA | 5:D:960:HOH:O | 2.33 | 0.75 |
| 4:D:791:LEU:O | 5:D:892:HOH:O | 2.04 | 0.75 |
| 1:T:125:DG:H1’ | 1:T:124:DC:H5’ | 1.67 | 0.75 |
| 4:D:741:LYS:NZ | 4:D:742:PRO:HD3 | 2.01 | 0.75 |
| 4:D:820:ASP:OD2 | 5:D:989:HOH:O | 2.05 | 0.75 |
| 4:D:117:ILE:C | 5:D:955:HOH:O | 2.25 | 0.75 |
| 4:D:428:ALA:CA | 5:D:944:HOH:O | 2.08 | 0.74 |
| 4:D:468:ALA:CB | 5:D:987:HOH:O | 2.33 | 0.74 |
| 4:D:594:VAL:O | 4:D:607:GLU:HB2 | 1.87 | 0.74 |
| 4:D:688:THR:O | 5:D:1007:HOH:O | 2.04 | 0.74 |
| 5:T:139:HOH:O | 4:D:780:PRO:HB2 | 1.87 | 0.74 |
| 4:D:873:ARG:CA | 5:D:1018:HOH:O | 2.35 | 0.74 |
| 4:D:103:PHE:HB3 | 5:D:946:HOH:O | 1.87 | 0.74 |
| 4:D:291:ARG:O | 4:D:293:PRO:HD3 | 1.88 | 0.74 |
| 4:D:748:ASN:OD1 | 4:D:754:GLN:HB3 | 1.86 | 0.74 |
| 4:D:453:GLY:HA3 | 5:D:980:HOH:O | 1.86 | 0.74 |
| 4:D:213:GLY:O | 4:D:217:ILE:HG12 | 1.88 | 0.73 |

*Continued on next page...*

*Continued from previous page...*

| Atom-1 | Atom-2 | Interatomic distance (Å) | Clash overlap (Å) |
|---|---|---|---|
| 4:D:729:THR:HA | 5:D:1041:HOH:O | 1.88 | 0.73 |
| 4:D:441:LYS:CG | 5:D:967:HOH:O | 2.35 | 0.73 |
| 4:D:454:LYS:O | 5:D:951:HOH:O | 2.06 | 0.73 |
| 4:D:691:ALA:HB1 | 5:D:909:HOH:O | 1.87 | 0.73 |
| 1:T:128:DC:O2 | 5:T:134:HOH:O | 2.05 | 0.73 |
| 4:D:129:ALA:O | 5:D:983:HOH:O | 2.04 | 0.73 |
| 4:D:552:ASP:HB2 | 4:D:691:ALA:HB2 | 1.68 | 0.73 |
| 4:D:587:ILE:O | 4:D:588:ASN:HB2 | 1.88 | 0.73 |
| 4:D:787:ASP:HB3 | 5:D:1026:HOH:O | 1.87 | 0.73 |
| 4:D:54:MET:HG3 | 4:D:57:ARG:NH2 | 2.04 | 0.73 |
| 4:D:352:ILE:HG22 | 4:D:352:ILE:O | 1.89 | 0.73 |
| 4:D:355:ILE:H | 4:D:355:ILE:CD1 | 1.97 | 0.73 |
| 4:D:360:LEU:H | 4:D:360:LEU:HD23 | 1.53 | 0.72 |
| 4:D:289:ASN:N | 5:D:960:HOH:O | 2.21 | 0.72 |
| 4:D:452:ILE:O | 5:D:980:HOH:O | 2.07 | 0.72 |
| 4:D:88:TRP:O | 4:D:92:VAL:HG12 | 1.89 | 0.72 |
| 4:D:819:ALA:HB3 | 5:D:947:HOH:O | 1.88 | 0.72 |
| 4:D:804:ILE:HD11 | 5:D:1004:HOH:O | 1.88 | 0.72 |
| 4:D:141:ILE:O | 4:D:145:ILE:CD1 | 2.36 | 0.72 |
| 4:D:247:ALA:HB3 | 4:D:250:TYR:HD2 | 1.55 | 0.72 |
| 4:D:795:VAL:HG23 | 5:D:892:HOH:O | 1.90 | 0.71 |
| 4:D:420:MET:HE2 | 4:D:424:GLY:HA2 | 1.73 | 0.71 |
| 4:D:682:TRP:O | 4:D:686:SER:HB2 | 1.91 | 0.71 |
| 3:R:5:G:O5' | 5:R:99:HOH:O | 2.09 | 0.71 |
| 4:D:594:VAL:HA | 4:D:608:LYS:O | 1.90 | 0.71 |
| 4:D:875:ILE:N | 5:D:1014:HOH:O | 2.23 | 0.71 |
| 4:D:107:GLN:OE1 | 5:D:1011:HOH:O | 2.09 | 0.70 |
| 4:D:117:ILE:HG22 | 5:D:955:HOH:O | 1.91 | 0.70 |
| 4:D:585:ASP:O | 4:D:614:LYS:HG2 | 1.91 | 0.70 |
| 4:D:217:ILE:O | 4:D:221:ILE:CD1 | 2.20 | 0.70 |
| 4:D:70:ALA:O | 4:D:74:ILE:HG12 | 1.90 | 0.70 |
| 4:D:577:LYS:NZ | 4:D:577:LYS:HB2 | 2.06 | 0.70 |
| 4:D:741:LYS:HD3 | 4:D:742:PRO:HD2 | 1.73 | 0.70 |
| 4:D:73:LEU:HD23 | 4:D:74:ILE:N | 2.06 | 0.69 |
| 4:D:278:TRP:H | 4:D:321:ASN:HD21 | 1.38 | 0.69 |
| 4:D:454:LYS:CD | 5:D:948:HOH:O | 2.40 | 0.69 |
| 4:D:423:ARG:HH12 | 4:D:784:HIS:HD2 | 1.39 | 0.69 |
| 4:D:488:ASN:ND2 | 5:D:993:HOH:O | 2.24 | 0.69 |
| 4:D:4:ILE:N | 5:D:985:HOH:O | 2.05 | 0.69 |
| 4:D:538:GLY:O | 5:D:984:HOH:O | 2.09 | 0.69 |
| 1:T:111:DT:C2' | 5:T:132:HOH:O | 2.36 | 0.69 |

*Continued on next page...*

*Continued from previous page...*

| Atom-1 | Atom-2 | Interatomic distance (Å) | Clash overlap (Å) |
|---|---|---|---|
| 4:D:859:ASP:O | 5:D:988:HOH:O | 2.11 | 0.69 |
| 2:N:14:DT:H2” | 2:N:15:DT:OP1 | 1.93 | 0.69 |
| 4:D:420:MET:CB | 5:D:957:HOH:O | 2.41 | 0.69 |
| 4:D:873:ARG:HA | 5:D:1018:HOH:O | 1.92 | 0.69 |
| 4:D:473:VAL:CG2 | 4:D:474:PRO:HD2 | 2.23 | 0.69 |
| 4:D:93:LYS:NZ | 5:D:1011:HOH:O | 2.26 | 0.69 |
| 4:D:146:GLU:OE2 | 5:D:971:HOH:O | 2.10 | 0.69 |
| 4:D:709:GLU:CA | 5:D:940:HOH:O | 2.41 | 0.68 |
| 4:D:730:PRO:HD3 | 5:D:1041:HOH:O | 1.93 | 0.68 |
| 3:R:1:A:H5” | 5:R:75:HOH:O | 1.93 | 0.68 |
| 4:D:324:GLN:HB3 | 4:D:418:TYR:HD1 | 1.58 | 0.68 |
| 4:D:765:LYS:HD3 | 4:D:766:ASP:N | 2.09 | 0.68 |
| 4:D:587:ILE:HA | 4:D:614:LYS:HE3 | 1.76 | 0.68 |
| 4:D:324:GLN:NE2 | 5:D:928:HOH:O | 2.27 | 0.67 |
| 4:D:546:PHE:O | 5:D:909:HOH:O | 2.11 | 0.67 |
| 4:D:709:GLU:HA | 5:D:940:HOH:O | 1.93 | 0.67 |
| 4:D:109:ILE:HD13 | 4:D:114:VAL:CG2 | 2.25 | 0.67 |
| 4:D:755:PHE:HB2 | 5:D:1024:HOH:O | 1.95 | 0.67 |
| 4:D:803:GLY:O | 5:D:966:HOH:O | 2.13 | 0.67 |
| 4:D:74:ILE:CG1 | 5:D:1017:HOH:O | 2.36 | 0.67 |
| 4:D:281:ILE:HD13 | 4:D:281:ILE:H | 1.59 | 0.67 |
| 1:T:130:DG:HO5’ | 1:T:130:DG:H8 | 1.41 | 0.67 |
| 4:D:804:ILE:HD12 | 4:D:820:ASP:CG | 2.15 | 0.66 |
| 4:D:354:ALA:HB3 | 4:D:391:ARG:HD2 | 1.77 | 0.66 |
| 4:D:81:MET:O | 4:D:85:ILE:HG12 | 1.95 | 0.66 |
| 4:D:458:TYR:O | 4:D:462:ILE:HD12 | 1.95 | 0.66 |
| 4:D:655:ILE:HD12 | 4:D:655:ILE:H | 1.60 | 0.66 |
| 4:D:78:LEU:HD13 | 4:D:119:ILE:HD12 | 1.77 | 0.66 |
| 4:D:209:SER:OG | 5:D:971:HOH:O | 2.12 | 0.66 |
| 1:T:127:DG:C5 | 5:T:134:HOH:O | 2.47 | 0.66 |
| 4:D:137:VAL:HG12 | 4:D:141:ILE:HD11 | 1.78 | 0.66 |
| 4:D:546:PHE:C | 5:D:909:HOH:O | 2.33 | 0.66 |
| 4:D:857:GLN:HA | 4:D:857:GLN:OE1 | 1.95 | 0.66 |
| 4:D:134:VAL:HG12 | 4:D:244:ILE:HG23 | 1.77 | 0.66 |
| 4:D:756:ARG:NH1 | 4:D:756:ARG:HG3 | 2.07 | 0.66 |
| 4:D:797:TRP:N | 5:D:1016:HOH:O | 2.27 | 0.66 |
| 4:D:95:LYS:O | 5:D:902:HOH:O | 2.12 | 0.66 |
| 4:D:505:GLN:HB3 | 5:D:952:HOH:O | 1.94 | 0.66 |
| 4:D:807:PHE:O | 5:D:941:HOH:O | 2.13 | 0.66 |
| 4:D:196:LEU:H | 4:D:196:LEU:HD22 | 1.61 | 0.66 |
| 4:D:4:ILE:CG2 | 5:D:942:HOH:O | 2.30 | 0.66 |

*Continued on next page...*

*Continued from previous page...*

| Atom-1 | Atom-2 | Interatomic distance (Å) | Clash overlap (Å) |
|---|---|---|---|
| 4:D:344:TRP:CE3 | 5:D:1015:HOH:O | 2.50 | 0.65 |
| 4:D:537:ASP:OD2 | 5:D:984:HOH:O | 2.13 | 0.65 |
| 4:D:217:ILE:HD13 | 5:D:1005:HOH:O | 1.95 | 0.65 |
| 4:D:352:ILE:O | 4:D:353:PRO:O | 2.15 | 0.65 |
| 4:D:308:TYR:O | 4:D:311:VAL:HG12 | 1.97 | 0.65 |
| 4:D:54:MET:HG3 | 4:D:57:ARG:HH21 | 1.61 | 0.65 |
| 4:D:779:ALA:O | 4:D:783:VAL:HG23 | 1.96 | 0.65 |
| 5:T:139:HOH:O | 4:D:780:PRO:HG2 | 1.97 | 0.65 |
| 4:D:829:ARG:NH2 | 4:D:882:PHE:HA | 2.11 | 0.65 |
| 4:D:25:ALA:HA | 5:D:937:HOH:O | 1.96 | 0.65 |
| 1:T:119:DT:O4’ | 5:T:139:HOH:O | 2.13 | 0.65 |
| 4:D:500:THR:OG1 | 5:D:1009:HOH:O | 2.15 | 0.65 |
| 4:D:593:GLU:HG2 | 4:D:594:VAL:H | 1.62 | 0.65 |
| 4:D:228:SER:HB3 | 4:D:245:GLU:OE1 | 1.96 | 0.64 |
| 4:D:488:ASN:CG | 5:D:993:HOH:O | 2.36 | 0.64 |
| 4:D:360:LEU:N | 4:D:360:LEU:HD23 | 2.13 | 0.64 |
| 4:D:650:VAL:HG12 | 4:D:655:ILE:CD1 | 2.27 | 0.64 |
| 4:D:669:GLN:HG3 | 4:D:672:GLN:HB2 | 1.79 | 0.64 |
| 4:D:250:TYR:O | 4:D:254:ILE:HD12 | 1.97 | 0.64 |
| 4:D:355:ILE:HD13 | 4:D:355:ILE:N | 2.09 | 0.64 |
| 5:T:139:HOH:O | 4:D:780:PRO:CG | 2.46 | 0.63 |
| 4:D:490:MET:HE3 | 5:D:926:HOH:O | 1.97 | 0.63 |
| 4:D:787:ASP:CB | 5:D:1026:HOH:O | 2.43 | 0.63 |
| 1:T:126:DT:C4 | 5:T:134:HOH:O | 2.51 | 0.63 |
| 4:D:99:ARG:O | 5:D:982:HOH:O | 2.15 | 0.63 |
| 4:D:595:VAL:HB | 4:D:607:GLU:HA | 1.81 | 0.63 |
| 4:D:71:LYS:HA | 5:D:1034:HOH:O | 1.98 | 0.63 |
| 4:D:664:GLY:HA3 | 5:D:1031:HOH:O | 1.88 | 0.63 |
| 4:D:746:ARG:HE | 4:D:754:GLN:NE2 | 1.92 | 0.63 |
| 4:D:720:ARG:NH2 | 5:D:1029:HOH:O | 2.31 | 0.63 |
| 4:D:353:PRO:HG3 | 4:D:394:ARG:HB2 | 1.80 | 0.63 |
| 4:D:532:LEU:O | 4:D:818:PRO:HD3 | 1.99 | 0.63 |
| 4:D:423:ARG:HH12 | 4:D:784:HIS:CD2 | 2.16 | 0.62 |
| 4:D:579:ASN:HA | 4:D:582:LEU:HB2 | 1.81 | 0.62 |
| 4:D:74:ILE:CD1 | 5:D:1017:HOH:O | 2.47 | 0.62 |
| 4:D:806:SER:O | 4:D:816:THR:HG23 | 1.99 | 0.62 |
| 1:T:119:DT:P | 5:T:141:HOH:O | 2.57 | 0.62 |
| 4:D:207:GLU:HG2 | 4:D:211:HIS:HE1 | 1.65 | 0.62 |
| 4:D:445:THR:CB | 5:D:974:HOH:O | 2.47 | 0.62 |
| 1:T:119:DT:OP2 | 5:T:141:HOH:O | 2.16 | 0.62 |
| 1:T:123:DG:H2” | 1:T:122:DC:H5’ | 1.82 | 0.62 |

*Continued on next page...*

*Continued from previous page...*

| Atom-1 | Atom-2 | Interatomic distance (Å) | Clash overlap (Å) |
|---|---|---|---|
| 4:D:562:LEU:HD21 | 4:D:870:LEU:CD1 | 2.30 | 0.62 |
| 4:D:14:ILE:HG23 | 4:D:288:ALA:HB1 | 1.81 | 0.61 |
| 4:D:180:LYS:HD2 | 4:D:181:ALA:N | 2.15 | 0.61 |
| 1:T:126:DT:C5 | 5:T:134:HOH:O | 2.52 | 0.61 |
| 4:D:340:VAL:CG2 | 4:D:341:ILE:N | 2.64 | 0.61 |
| 4:D:725:VAL:HG23 | 4:D:774:GLN:NE2 | 2.15 | 0.61 |
| 4:D:117:ILE:HG21 | 4:D:145:ILE:HD12 | 1.80 | 0.61 |
| 4:D:25:ALA:O | 4:D:26:ASP:HB2 | 2.00 | 0.61 |
| 4:D:50:ARG:NH2 | 4:D:267:MET:HG2 | 2.15 | 0.61 |
| 4:D:104:GLN:N | 5:D:946:HOH:O | 2.17 | 0.61 |
| 4:D:330:ILE:HD12 | 4:D:330:ILE:N | 2.16 | 0.61 |
| 1:T:120:DT:H1' | 1:T:119:DT:H71 | 1.81 | 0.61 |
| 4:D:710:VAL:HG13 | 4:D:720:ARG:H | 1.64 | 0.60 |
| 4:D:330:ILE:HD13 | 4:D:408:PHE:O | 1.99 | 0.60 |
| 4:D:666:MET:HE1 | 5:D:1031:HOH:O | 2.01 | 0.60 |
| 4:D:790:HIS:HD2 | 5:D:995:HOH:O | 1.80 | 0.60 |
| 4:D:264:ILE:HB | 4:D:292:ARG:HD2 | 1.83 | 0.60 |
| 3:R:4:G:O3' | 4:D:394:ARG:NH1 | 2.33 | 0.60 |
| 4:D:420:MET:N | 5:D:957:HOH:O | 2.28 | 0.60 |
| 4:D:797:TRP:CE2 | 5:D:910:HOH:O | 2.51 | 0.60 |
| 2:N:25:DC:H1' | 2:N:26:DA:H5" | 1.83 | 0.60 |
| 4:D:873:ARG:O | 5:D:1018:HOH:O | 2.17 | 0.60 |
| 4:D:746:ARG:NE | 4:D:754:GLN:HE22 | 1.94 | 0.60 |
| 4:D:19:ILE:HB | 4:D:20:PRO:HD3 | 1.82 | 0.60 |
| 4:D:292:ARG:HA | 4:D:292:ARG:NE | 2.14 | 0.60 |
| 4:D:445:THR:CA | 5:D:974:HOH:O | 2.33 | 0.60 |
| 4:D:816:THR:HG21 | 5:D:989:HOH:O | 1.94 | 0.60 |
| 1:T:119:DT:C4' | 5:T:139:HOH:O | 2.49 | 0.60 |
| 4:D:141:ILE:CD1 | 4:D:217:ILE:HD11 | 2.32 | 0.60 |
| 4:D:82:ILE:HG12 | 4:D:112:GLU:HA | 1.83 | 0.59 |
| 1:T:117:DG:H2" | 1:T:116:DC:H5' | 1.82 | 0.59 |
| 4:D:157:LEU:HD23 | 4:D:196:LEU:HD12 | 1.83 | 0.59 |
| 4:D:320:ILE:HD11 | 4:D:420:MET:SD | 2.41 | 0.59 |
| 4:D:360:LEU:HB3 | 4:D:384:VAL:HG21 | 1.83 | 0.59 |
| 4:D:765:LYS:HA | 4:D:765:LYS:HZ3 | 1.68 | 0.59 |
| 4:D:539:SER:O | 4:D:540:CYS:HB2 | 2.03 | 0.59 |
| 4:D:796:VAL:CB | 5:D:1016:HOH:O | 2.36 | 0.59 |
| 4:D:16:LEU:HG | 4:D:20:PRO:HB2 | 1.85 | 0.59 |
| 4:D:141:ILE:HD13 | 4:D:217:ILE:HD11 | 1.84 | 0.59 |
| 4:D:439:MET:HB2 | 5:D:934:HOH:O | 2.03 | 0.59 |
| 4:D:857:GLN:HE22 | 4:D:859:ASP:HB2 | 1.67 | 0.59 |

*Continued on next page...*

*Continued from previous page...*

| Atom-1 | Atom-2 | Interatomic distance (Å) | Clash overlap (Å) |
|---|---|---|---|
| 4:D:404:GLN:N | 5:D:973:HOH:O | 2.35 | 0.58 |
| 4:D:655:ILE:O | 4:D:658:ALA:N | 2.34 | 0.58 |
| 1:T:119:DT:H6 | 5:T:141:HOH:O | 1.85 | 0.58 |
| 4:D:13:ASP:CG | 4:D:14:ILE:HD12 | 2.24 | 0.58 |
| 4:D:157:LEU:O | 4:D:159:ALA:N | 2.35 | 0.58 |
| 4:D:169:GLN:H | 4:D:169:GLN:CD | 2.07 | 0.58 |
| 3:R:9:A:H2 | 5:D:991:HOH:O | 1.86 | 0.58 |
| 1:T:119:DT:C7 | 5:T:141:HOH:O | 2.32 | 0.58 |
| 4:D:826:LYS:HE2 | 4:D:830:GLU:OE2 | 2.04 | 0.58 |
| 4:D:85:ILE:HA | 4:D:219:MET:HE1 | 1.86 | 0.58 |
| 2:N:17:DA:H3’ | 2:N:18:DC:H5” | 1.85 | 0.58 |
| 4:D:337:VAL:HG11 | 4:D:512:LEU:HD21 | 1.86 | 0.58 |
| 4:D:562:LEU:HD21 | 4:D:870:LEU:HD12 | 1.84 | 0.58 |
| 4:D:80:LYS:O | 5:D:1048:HOH:O | 2.16 | 0.58 |
| 4:D:153:ARG:O | 4:D:157:LEU:HB3 | 2.04 | 0.58 |
| 4:D:153:ARG:C | 4:D:155:ARG:H | 2.06 | 0.58 |
| 4:D:25:ALA:CA | 5:D:937:HOH:O | 2.52 | 0.58 |
| 3:R:10:G:H8 | 4:D:301:SER:OG | 1.87 | 0.58 |
| 4:D:812:ASP:OD2 | 5:D:984:HOH:O | 2.17 | 0.58 |
| 4:D:85:ILE:HA | 4:D:219:MET:CE | 2.34 | 0.58 |
| 4:D:340:VAL:HG23 | 4:D:341:ILE:N | 2.19 | 0.58 |
| 4:D:344:TRP:HB3 | 5:D:1015:HOH:O | 1.88 | 0.57 |
| 4:D:130:ASP:C | 4:D:132:THR:H | 2.07 | 0.57 |
| 4:D:318:LYS:O | 4:D:322:ILE:HG12 | 2.03 | 0.57 |
| 4:D:137:VAL:HG12 | 4:D:141:ILE:CD1 | 2.34 | 0.57 |
| 4:D:420:MET:HB3 | 5:D:957:HOH:O | 2.04 | 0.57 |
| 4:D:454:LYS:CE | 5:D:948:HOH:O | 2.45 | 0.57 |
| 4:D:557:ARG:O | 4:D:568:GLN:HG3 | 2.04 | 0.57 |
| 4:D:611:LEU:HD22 | 4:D:615:ALA:HB1 | 1.85 | 0.57 |
| 4:D:633:SER:HA | 4:D:649:GLN:HE22 | 1.69 | 0.57 |
| 4:D:729:THR:HG22 | 5:D:1041:HOH:O | 2.03 | 0.57 |
| 4:D:154:ILE:HD11 | 4:D:183:MET:CE | 2.33 | 0.57 |
| 4:D:420:MET:CE | 4:D:424:GLY:HA2 | 2.34 | 0.57 |
| 4:D:335:LEU:HD22 | 4:D:339:ASN:ND2 | 2.19 | 0.57 |
| 4:D:404:GLN:CA | 5:D:973:HOH:O | 2.53 | 0.57 |
| 4:D:727:TRP:NE1 | 5:D:1037:HOH:O | 2.38 | 0.57 |
| 4:D:110:LYS:HB3 | 4:D:112:GLU:OE2 | 2.05 | 0.57 |
| 4:D:420:MET:HE3 | 4:D:424:GLY:O | 2.05 | 0.57 |
| 4:D:589:GLY:C | 4:D:614:LYS:HG3 | 2.25 | 0.57 |
| 4:D:689:VAL:C | 5:D:972:HOH:O | 2.42 | 0.57 |
| 4:D:797:TRP:CZ3 | 4:D:801:LYS:HE3 | 2.40 | 0.57 |

*Continued on next page...*

*Continued from previous page...*

| Atom-1 | Atom-2 | Interatomic distance (Å) | Clash overlap (Å) |
|---|---|---|---|
| 4:D:14:ILE:HG13 | 4:D:290:GLY:HA2 | 1.87 | 0.57 |
| 4:D:851:ASP:O | 4:D:853:LEU:N | 2.38 | 0.57 |
| 4:D:407:LYS:HB2 | 5:D:973:HOH:O | 2.04 | 0.56 |
| 2:N:22:DG:H2" | 2:N:23:DC:C6 | 2.40 | 0.56 |
| 1:T:120:DT:H1' | 5:T:141:HOH:O | 2.04 | 0.56 |
| 4:D:208:ASP:C | 4:D:210:ILE:H | 2.09 | 0.56 |
| 4:D:25:ALA:HB2 | 5:D:937:HOH:O | 2.03 | 0.56 |
| 4:D:30:GLU:N | 5:D:937:HOH:O | 2.38 | 0.56 |
| 4:D:440:THR:HG22 | 5:D:967:HOH:O | 2.04 | 0.56 |
| 4:D:710:VAL:N | 5:D:940:HOH:O | 2.38 | 0.56 |
| 4:D:147:ASP:HB3 | 4:D:751:PHE:HE2 | 1.70 | 0.56 |
| 4:D:281:ILE:CD1 | 4:D:281:ILE:H | 2.19 | 0.56 |
| 4:D:344:TRP:HE3 | 5:D:1015:HOH:O | 1.87 | 0.56 |
| 1:T:111:DT:OP2 | 1:T:111:DT:H3' | 2.05 | 0.56 |
| 5:T:139:HOH:O | 4:D:780:PRO:CB | 2.50 | 0.56 |
| 4:D:517:GLU:HG3 | 4:D:532:LEU:HB2 | 1.86 | 0.56 |
| 4:D:325:ASN:HB3 | 5:D:919:HOH:O | 2.06 | 0.56 |
| 4:D:360:LEU:CB | 5:D:968:HOH:O | 2.12 | 0.56 |
| 4:D:780:PRO:CB | 5:D:975:HOH:O | 2.54 | 0.56 |
| 4:D:797:TRP:NE1 | 5:D:910:HOH:O | 2.38 | 0.56 |
| 4:D:684:SER:O | 4:D:687:VAL:HG22 | 2.06 | 0.56 |
| 4:D:454:LYS:HD3 | 5:D:948:HOH:O | 2.05 | 0.56 |
| 4:D:221:ILE:CD1 | 4:D:221:ILE:N | 2.69 | 0.55 |
| 4:D:255:ALA:CA | 5:D:942:HOH:O | 2.49 | 0.55 |
| 4:D:25:ALA:CB | 5:D:937:HOH:O | 2.54 | 0.55 |
| 4:D:134:VAL:HG11 | 4:D:229:LEU:CD2 | 2.36 | 0.55 |
| 4:D:300:HIS:O | 4:D:301:SER:HB2 | 2.06 | 0.55 |
| 1:T:114:DG:H4' | 4:D:396:ILE:HG21 | 1.88 | 0.55 |
| 4:D:710:VAL:CG1 | 4:D:720:ARG:HB3 | 2.37 | 0.55 |
| 4:D:726:HIS:HB2 | 4:D:736:TRP:CD1 | 2.41 | 0.55 |
| 4:D:804:ILE:CD1 | 5:D:1004:HOH:O | 2.50 | 0.55 |
| 4:D:264:ILE:HD12 | 4:D:264:ILE:O | 2.05 | 0.55 |
| 4:D:471:ASP:OD1 | 4:D:472:LYS:HE2 | 2.06 | 0.55 |
| 4:D:593:GLU:HG2 | 4:D:594:VAL:N | 2.22 | 0.55 |
| 4:D:619:GLN:OE1 | 4:D:667:PHE:HA | 2.06 | 0.55 |
| 1:T:127:DG:H1' | 1:T:126:DT:C5' | 2.36 | 0.55 |
| 4:D:16:LEU:CG | 4:D:20:PRO:HB2 | 2.37 | 0.55 |
| 4:D:689:VAL:HG23 | 4:D:689:VAL:O | 2.06 | 0.55 |
| 3:R:10:G:N2 | 5:R:46:HOH:O | 2.31 | 0.55 |
| 4:D:154:ILE:HD11 | 4:D:183:MET:HE3 | 1.89 | 0.55 |
| 4:D:779:ALA:O | 4:D:783:VAL:CG2 | 2.55 | 0.55 |

*Continued on next page...*

*Continued from previous page...*

| Atom-1 | Atom-2 | Interatomic distance (Å) | Clash overlap (Å) |
|---|---|---|---|
| 4:D:830:GLU:CB | 5:D:910:HOH:O | 2.43 | 0.55 |
| 4:D:428:ALA:HB1 | 5:D:944:HOH:O | 1.97 | 0.55 |
| 4:D:829:ARG:NH1 | 4:D:878:SER:O | 2.40 | 0.55 |
| 4:D:420:MET:CG | 5:D:957:HOH:O | 2.54 | 0.55 |
| 4:D:543:ILE:HD12 | 4:D:543:ILE:H | 1.71 | 0.54 |
| 4:D:655:ILE:O | 4:D:656:GLN:C | 2.44 | 0.54 |
| 4:D:590:THR:HG22 | 4:D:591:ASP:N | 2.22 | 0.54 |
| 4:D:160:LYS:HD3 | 4:D:163:LYS:HD2 | 1.88 | 0.54 |
| 4:D:853:LEU:O | 4:D:854:HIS:HB2 | 2.08 | 0.54 |
| 4:D:545:HIS:O | 4:D:549:MET:HG2 | 2.07 | 0.54 |
| 4:D:651:LEU:HG | 4:D:656:GLN:HE21 | 1.73 | 0.54 |
| 4:D:157:LEU:CD2 | 4:D:196:LEU:HD12 | 2.37 | 0.54 |
| 4:D:553:GLU:HB3 | 5:D:893:HOH:O | 2.06 | 0.54 |
| 4:D:13:ASP:OD2 | 4:D:14:ILE:HD12 | 2.07 | 0.54 |
| 4:D:347:CYS:HA | 5:D:1015:HOH:O | 2.06 | 0.54 |
| 4:D:400:PHE:O | 4:D:404:GLN:HB2 | 2.07 | 0.54 |
| 4:D:570:ILE:O | 4:D:574:VAL:HG23 | 2.08 | 0.54 |
| 4:D:160:LYS:HZ3 | 4:D:160:LYS:HB2 | 1.73 | 0.53 |
| 4:D:810:ILE:HD12 | 4:D:810:ILE:N | 2.23 | 0.53 |
| 4:D:292:ARG:CA | 4:D:292:ARG:HE | 2.18 | 0.53 |
| 4:D:410:ASN:N | 5:D:903:HOH:O | 2.21 | 0.53 |
| 4:D:806:SER:HB2 | 5:D:1008:HOH:O | 2.07 | 0.53 |
| 4:D:160:LYS:CD | 4:D:163:LYS:HD2 | 2.39 | 0.53 |
| 4:D:335:LEU:CD2 | 4:D:339:ASN:ND2 | 2.72 | 0.53 |
| 4:D:791:LEU:HA | 4:D:814:PHE:HE2 | 1.73 | 0.53 |
| 4:D:92:VAL:HG11 | 4:D:103:PHE:CD1 | 2.43 | 0.53 |
| 4:D:196:LEU:HD22 | 4:D:196:LEU:N | 2.23 | 0.53 |
| 4:D:2:ASN:O | 4:D:127:THR:HA | 2.09 | 0.53 |
| 1:T:126:DT:C2’ | 1:T:125:DG:H5’ | 2.33 | 0.53 |
| 4:D:790:HIS:HD2 | 5:D:904:HOH:O | 1.92 | 0.53 |
| 4:D:141:ILE:C | 4:D:145:ILE:HD13 | 2.25 | 0.52 |
| 4:D:6:ILE:HD12 | 4:D:6:ILE:N | 2.24 | 0.52 |
| 4:D:810:ILE:CD1 | 5:D:932:HOH:O | 2.57 | 0.52 |
| 4:D:153:ARG:NH2 | 4:D:201:TRP:HB2 | 2.24 | 0.52 |
| 5:T:131:HOH:O | 4:D:421:ASP:HB2 | 2.08 | 0.52 |
| 4:D:6:ILE:H | 4:D:6:ILE:HD12 | 1.75 | 0.52 |
| 1:T:120:DT:H2” | 5:T:141:HOH:O | 2.09 | 0.52 |
| 4:D:650:VAL:HG12 | 4:D:655:ILE:HD11 | 1.91 | 0.52 |
| 4:D:780:PRO:CA | 5:D:975:HOH:O | 2.58 | 0.52 |
| 1:T:122:DC:H2” | 1:T:121:DA:O5’ | 2.10 | 0.52 |
| 4:D:335:LEU:HD21 | 4:D:406:ASN:OD1 | 2.10 | 0.52 |

*Continued on next page...*

*Continued from previous page...*

| Atom-1 | Atom-2 | Interatomic distance (Å) | Clash overlap (Å) |
|---|---|---|---|
| 4:D:421:ASP:O | 5:D:1002:HOH:O | 2.18 | 0.52 |
| 4:D:147:ASP:HB3 | 4:D:751:PHE:CE2 | 2.45 | 0.52 |
| 4:D:226:MET:HA | 4:D:250:TYR:CD2 | 2.44 | 0.52 |
| 4:D:727:TRP:CZ3 | 5:D:1012:HOH:O | 2.54 | 0.52 |
| 4:D:380:ALA:C | 5:D:986:HOH:O | 2.47 | 0.52 |
| 4:D:533:PRO:CD | 5:D:974:HOH:O | 2.57 | 0.52 |
| 4:D:610:LYS:HA | 4:D:672:GLN:OE1 | 2.09 | 0.52 |
| 4:D:181:ALA:O | 4:D:185:VAL:HG23 | 2.10 | 0.51 |
| 4:D:360:LEU:C | 5:D:968:HOH:O | 2.44 | 0.51 |
| 3:R:10:G:C8 | 4:D:301:SER:OG | 2.63 | 0.51 |
| 4:D:41:HIS:O | 4:D:45:GLU:HG3 | 2.10 | 0.51 |
| 4:D:709:GLU:C | 5:D:940:HOH:O | 2.49 | 0.51 |
| 1:T:111:DT:H3' | 1:T:111:DT:P | 2.49 | 0.51 |
| 4:D:65:ALA:CA | 5:D:1017:HOH:O | 2.39 | 0.51 |
| 4:D:577:LYS:HZ3 | 4:D:577:LYS:HB2 | 1.75 | 0.51 |
| 4:D:2:ASN:OD1 | 4:D:245:GLU:HB2 | 2.11 | 0.51 |
| 4:D:461:LYS:HA | 5:D:1036:HOH:O | 2.11 | 0.51 |
| 4:D:182:PHE:O | 4:D:186:VAL:HG23 | 2.11 | 0.51 |
| 1:T:120:DT:C2' | 5:T:141:HOH:O | 2.59 | 0.51 |
| 4:D:595:VAL:CG2 | 4:D:608:LYS:H | 2.23 | 0.51 |
| 4:D:118:THR:HG22 | 4:D:220:LEU:HD12 | 1.93 | 0.51 |
| 4:D:433:ASN:HB2 | 4:D:434:PRO:CD | 2.41 | 0.51 |
| 4:D:652:GLU:O | 4:D:657:PRO:HD3 | 2.11 | 0.51 |
| 4:D:255:ALA:N | 5:D:942:HOH:O | 2.44 | 0.50 |
| 4:D:32:LEU:HG | 5:D:1047:HOH:O | 2.11 | 0.50 |
| 4:D:706:LEU:HA | 5:D:1029:HOH:O | 2.11 | 0.50 |
| 4:D:709:GLU:CG | 5:D:940:HOH:O | 2.60 | 0.50 |
| 4:D:215:ARG:O | 4:D:219:MET:HG3 | 2.11 | 0.50 |
| 4:D:30:GLU:HG3 | 4:D:34:ARG:HH21 | 1.76 | 0.50 |
| 4:D:339:ASN:OD1 | 4:D:402:LEU:HD11 | 2.11 | 0.50 |
| 4:D:804:ILE:HD12 | 4:D:820:ASP:HB3 | 1.93 | 0.50 |
| 4:D:210:ILE:O | 4:D:214:VAL:HG23 | 2.11 | 0.50 |
| 4:D:33:ALA:HB3 | 5:D:937:HOH:O | 2.12 | 0.50 |
| 4:D:550:LEU:HB2 | 4:D:691:ALA:HB1 | 1.92 | 0.50 |
| 4:D:143:ARG:HH11 | 4:D:143:ARG:HG2 | 1.76 | 0.50 |
| 4:D:30:GLU:HA | 5:D:937:HOH:O | 2.12 | 0.50 |
| 4:D:341:ILE:HD11 | 4:D:348:PRO:CG | 2.40 | 0.50 |
| 4:D:804:ILE:HD12 | 4:D:820:ASP:CB | 2.42 | 0.50 |
| 4:D:85:ILE:HG22 | 4:D:89:PHE:CE2 | 2.46 | 0.50 |
| 4:D:778:ILE:HG23 | 4:D:779:ALA:N | 2.26 | 0.50 |
| 4:D:551:ARG:HB2 | 4:D:868:GLY:H | 1.76 | 0.50 |

*Continued on next page...*

*Continued from previous page...*

| Atom-1 | Atom-2 | Interatomic distance (Å) | Clash overlap (Å) |
|---|---|---|---|
| 4:D:655:ILE:HG23 | 4:D:659:ILE:HD13 | 1.93 | 0.50 |
| 1:T:119:DT:C6 | 5:T:141:HOH:O | 2.55 | 0.50 |
| 4:D:217:ILE:CA | 5:D:1005:HOH:O | 2.57 | 0.50 |
| 4:D:276:LYS:CE | 5:D:1032:HOH:O | 2.07 | 0.50 |
| 4:D:631:LYS:HD3 | 4:D:635:MET:SD | 2.51 | 0.50 |
| 4:D:711:LYS:NZ | 5:D:1013:HOH:O | 2.45 | 0.50 |
| 4:D:707:ALA:O | 4:D:722:ARG:HG2 | 2.10 | 0.50 |
| 4:D:417:PRO:HG2 | 4:D:429:VAL:HB | 1.94 | 0.50 |
| 4:D:651:LEU:HG | 4:D:656:GLN:NE2 | 2.26 | 0.50 |
| 4:D:312:TYR:HD2 | 5:D:976:HOH:O | 1.94 | 0.49 |
| 4:D:379:ARG:C | 4:D:381:ALA:H | 2.15 | 0.49 |
| 4:D:379:ARG:C | 4:D:381:ALA:N | 2.65 | 0.49 |
| 4:D:727:TRP:CE3 | 5:D:1012:HOH:O | 2.64 | 0.49 |
| 4:D:404:GLN:HA | 5:D:973:HOH:O | 2.12 | 0.49 |
| 4:D:157:LEU:HD12 | 4:D:158:GLU:H | 1.78 | 0.49 |
| 4:D:117:ILE:HG12 | 4:D:752:LEU:HD12 | 1.95 | 0.49 |
| 4:D:875:ILE:CG1 | 5:D:1014:HOH:O | 2.50 | 0.49 |
| 4:D:159:ALA:HA | 4:D:195:LEU:HD21 | 1.94 | 0.49 |
| 4:D:254:ILE:C | 5:D:942:HOH:O | 2.49 | 0.49 |
| 4:D:291:ARG:C | 4:D:293:PRO:HD3 | 2.32 | 0.49 |
| 4:D:495:SER:HA | 5:D:929:HOH:O | 2.13 | 0.49 |
| 4:D:710:VAL:HG13 | 4:D:720:ARG:N | 2.28 | 0.49 |
| 4:D:417:PRO:O | 5:D:944:HOH:O | 2.20 | 0.49 |
| 4:D:593:GLU:CG | 4:D:594:VAL:H | 2.25 | 0.49 |
| 4:D:653:ASP:O | 4:D:657:PRO:HG3 | 2.12 | 0.49 |
| 1:T:127:DG:H2” | 1:T:126:DT:OP2 | 2.13 | 0.49 |
| 4:D:47:GLY:HA3 | 4:D:265:SER:O | 2.12 | 0.49 |
| 4:D:352:ILE:CG2 | 4:D:352:ILE:O | 2.61 | 0.49 |
| 4:D:854:HIS:O | 4:D:855:GLU:O | 2.31 | 0.49 |
| 4:D:654:THR:O | 4:D:657:PRO:HG2 | 2.13 | 0.48 |
| 4:D:13:ASP:OD1 | 4:D:14:ILE:HD12 | 2.12 | 0.48 |
| 4:D:195:LEU:C | 4:D:197:GLY:H | 2.16 | 0.48 |
| 4:D:473:VAL:HG11 | 4:D:477:GLU:OE2 | 2.13 | 0.48 |
| 4:D:207:GLU:HG2 | 4:D:211:HIS:CE1 | 2.45 | 0.48 |
| 4:D:543:ILE:N | 4:D:543:ILE:HD12 | 2.27 | 0.48 |
| 4:D:620:TRP:O | 4:D:623:TYR:HB3 | 2.13 | 0.48 |
| 4:D:744:GLN:NE2 | 4:D:758:GLN:NE2 | 2.61 | 0.48 |
| 4:D:833:VAL:O | 4:D:837:GLU:HB2 | 2.12 | 0.48 |
| 4:D:873:ARG:CB | 5:D:1018:HOH:O | 2.57 | 0.48 |
| 4:D:349:VAL:HG13 | 4:D:508:PRO:HG3 | 1.96 | 0.48 |
| 4:D:360:LEU:CD2 | 4:D:360:LEU:N | 2.76 | 0.48 |

*Continued on next page...*

*Continued from previous page...*

| Atom-1 | Atom-2 | Interatomic distance (Å) | Clash overlap (Å) |
|---|---|---|---|
| 4:D:584:ALA:O | 4:D:588:ASN:HB3 | 2.14 | 0.48 |
| 4:D:88:TRP:O | 4:D:91:GLU:HG2 | 2.14 | 0.48 |
| 4:D:162:PHE:C | 4:D:164:LYS:H | 2.17 | 0.48 |
| 4:D:330:ILE:CD1 | 5:D:895:HOH:O | 2.33 | 0.48 |
| 4:D:729:THR:CB | 5:D:1041:HOH:O | 2.61 | 0.48 |
| 4:D:533:PRO:HA | 4:D:816:THR:O | 2.13 | 0.48 |
| 4:D:707:ALA:O | 4:D:722:ARG:NH1 | 2.43 | 0.48 |
| 2:N:28:DG:H2” | 2:N:29:DG:H8 | 1.79 | 0.48 |
| 4:D:468:ALA:HA | 5:D:952:HOH:O | 2.13 | 0.48 |
| 4:D:828:VAL:HG11 | 4:D:882:PHE:O | 2.13 | 0.48 |
| 2:N:22:DG:H2” | 2:N:23:DC:C5 | 2.48 | 0.48 |
| 4:D:276:LYS:CD | 5:D:1032:HOH:O | 2.55 | 0.48 |
| 4:D:320:ILE:O | 4:D:324:GLN:HG2 | 2.14 | 0.48 |
| 4:D:286:TYR:CZ | 4:D:417:PRO:HG3 | 2.48 | 0.48 |
| 1:T:117:DG:H2” | 1:T:116:DC:C5’ | 2.43 | 0.48 |
| 4:D:278:TRP:CH2 | 4:D:294:LEU:HB3 | 2.50 | 0.47 |
| 4:D:595:VAL:CB | 4:D:607:GLU:HA | 2.43 | 0.47 |
| 4:D:375:THR:O | 4:D:376:ALA:HB3 | 2.14 | 0.47 |
| 4:D:650:VAL:O | 4:D:655:ILE:HD12 | 2.14 | 0.47 |
| 4:D:25:ALA:C | 4:D:27:HIS:H | 2.17 | 0.47 |
| 4:D:669:GLN:HG2 | 4:D:672:GLN:NE2 | 2.30 | 0.47 |
| 4:D:537:ASP:N | 4:D:882:PHE:HD1 | 2.13 | 0.47 |
| 1:T:128:DC:H42 | 2:N:27:DC:H42 | 1.61 | 0.47 |
| 3:R:6:C:H5’ | 4:D:171:ASN:O | 2.15 | 0.47 |
| 4:D:46:MET:O | 4:D:267:MET:HE2 | 2.15 | 0.47 |
| 4:D:690:VAL:N | 5:D:972:HOH:O | 2.48 | 0.47 |
| 4:D:109:ILE:HD12 | 4:D:109:ILE:H | 1.80 | 0.47 |
| 4:D:93:LYS:HE3 | 4:D:103:PHE:HZ | 1.79 | 0.47 |
| 4:D:20:PRO:O | 4:D:23:THR:HB | 2.14 | 0.47 |
| 4:D:330:ILE:CD1 | 4:D:330:ILE:N | 2.78 | 0.47 |
| 4:D:735:VAL:CG2 | 5:D:1037:HOH:O | 2.63 | 0.47 |
| 4:D:254:ILE:HG22 | 5:D:942:HOH:O | 2.15 | 0.46 |
| 4:D:676:TYR:O | 4:D:679:LYS:HB3 | 2.16 | 0.46 |
| 4:D:828:VAL:CG1 | 4:D:882:PHE:O | 2.63 | 0.46 |
| 4:D:577:LYS:HD3 | 4:D:687:VAL:CG2 | 2.45 | 0.46 |
| 4:D:700:LYS:NZ | 4:D:700:LYS:HB3 | 2.30 | 0.46 |
| 4:D:226:MET:O | 4:D:246:LEU:HD12 | 2.16 | 0.46 |
| 4:D:473:VAL:HG22 | 4:D:474:PRO:CD | 2.40 | 0.46 |
| 4:D:4:ILE:HD13 | 4:D:254:ILE:HG21 | 1.98 | 0.46 |
| 4:D:696:MET:O | 4:D:700:LYS:HG3 | 2.16 | 0.46 |
| 4:D:85:ILE:HD13 | 4:D:219:MET:SD | 2.55 | 0.46 |

*Continued on next page...*

*Continued from previous page...*

| Atom-1 | Atom-2 | Interatomic distance (Å) | Clash overlap (Å) |
|---|---|---|---|
| 4:D:296:LEU:CD2 | 4:D:320:ILE:HD13 | 2.32 | 0.46 |
| 4:D:802:TYR:OH | 4:D:826:LYS:HD3 | 2.15 | 0.46 |
| 4:D:817:ILE:CG1 | 5:D:947:HOH:O | 2.60 | 0.46 |
| 2:N:27:DC:H2" | 2:N:28:DG:H5" | 1.98 | 0.46 |
| 4:D:70:ALA:HB1 | 5:D:1017:HOH:O | 2.15 | 0.46 |
| 4:D:481:PHE:HE2 | 5:D:987:HOH:O | 1.98 | 0.46 |
| 4:D:765:LYS:HD3 | 4:D:766:ASP:H | 1.78 | 0.46 |
| 4:D:379:ARG:O | 5:D:986:HOH:O | 2.21 | 0.45 |
| 4:D:669:GLN:HG2 | 4:D:672:GLN:HE21 | 1.81 | 0.45 |
| 4:D:855:GLU:HB3 | 4:D:856:SER:H | 1.44 | 0.45 |
| 1:T:126:DT:H1' | 1:T:125:DG:H5" | 1.98 | 0.45 |
| 4:D:157:LEU:HG | 4:D:196:LEU:HD12 | 1.98 | 0.45 |
| 4:D:421:ASP:C | 4:D:421:ASP:OD1 | 2.54 | 0.45 |
| 4:D:763:THR:CG2 | 5:D:962:HOH:O | 0.76 | 0.45 |
| 4:D:360:LEU:O | 4:D:362:MET:SD | 2.75 | 0.45 |
| 1:T:112:DG:N3 | 1:T:112:DG:O4' | 2.50 | 0.45 |
| 4:D:505:GLN:CA | 5:D:952:HOH:O | 2.64 | 0.45 |
| 1:T:113:DT:H2' | 4:D:57:ARG:NH1 | 2.31 | 0.45 |
| 4:D:105:PHE:O | 4:D:108:GLU:HG2 | 2.17 | 0.45 |
| 4:D:169:GLN:CD | 4:D:169:GLN:N | 2.69 | 0.45 |
| 4:D:577:LYS:HZ2 | 4:D:577:LYS:HB2 | 1.80 | 0.45 |
| 4:D:659:ILE:HD11 | 4:D:667:PHE:HD2 | 1.81 | 0.45 |
| 4:D:330:ILE:HD11 | 4:D:408:PHE:CB | 2.47 | 0.45 |
| 4:D:398:LEU:O | 4:D:402:LEU:HB2 | 2.17 | 0.45 |
| 4:D:461:LYS:HE2 | 4:D:479:ILE:HD12 | 1.97 | 0.45 |
| 4:D:621:LEU:C | 4:D:623:TYR:H | 2.19 | 0.45 |
| 4:D:655:ILE:CG2 | 4:D:659:ILE:HD13 | 2.47 | 0.45 |
| 4:D:74:ILE:HD11 | 5:D:1017:HOH:O | 2.11 | 0.45 |
| 4:D:875:ILE:CA | 5:D:1014:HOH:O | 2.64 | 0.45 |
| 4:D:162:PHE:O | 4:D:167:GLU:N | 2.49 | 0.45 |
| 4:D:270:PRO:CB | 5:D:935:HOH:O | 2.62 | 0.45 |
| 4:D:655:ILE:N | 4:D:655:ILE:HD12 | 2.30 | 0.45 |
| 4:D:816:THR:HG22 | 4:D:817:ILE:H | 1.82 | 0.45 |
| 1:T:126:DT:H1' | 1:T:125:DG:C5' | 2.46 | 0.45 |
| 4:D:160:LYS:C | 4:D:162:PHE:N | 2.68 | 0.45 |
| 4:D:229:LEU:HD23 | 4:D:244:ILE:HG23 | 1.98 | 0.45 |
| 4:D:321:ASN:O | 4:D:325:ASN:HB2 | 2.17 | 0.45 |
| 1:T:115:DC:O5' | 1:T:115:DC:H2' | 2.17 | 0.45 |
| 4:D:727:TRP:CZ2 | 4:D:782:PHE:HA | 2.52 | 0.45 |
| 4:D:533:PRO:HD2 | 5:D:974:HOH:O | 2.16 | 0.44 |
| 4:D:633:SER:CA | 4:D:649:GLN:HE22 | 2.30 | 0.44 |

*Continued on next page...*

*Continued from previous page...*

| Atom-1 | Atom-2 | Interatomic distance (Å) | Clash overlap (Å) |
|---|---|---|---|
| 4:D:706:LEU:O | 4:D:723:CYS:N | 2.43 | 0.44 |
| 1:T:123:DG:H2” | 1:T:122:DC:C5’ | 2.47 | 0.44 |
| 4:D:276:LYS:CG | 5:D:1032:HOH:O | 2.50 | 0.44 |
| 4:D:416:PHE:HE1 | 5:D:935:HOH:O | 2.01 | 0.44 |
| 4:D:651:LEU:HA | 4:D:655:ILE:HD13 | 1.99 | 0.44 |
| 4:D:73:LEU:O | 4:D:77:LEU:HG | 2.17 | 0.44 |
| 2:N:17:DA:C3’ | 2:N:18:DC:H5” | 2.46 | 0.44 |
| 4:D:154:ILE:HG22 | 4:D:196:LEU:HD21 | 2.00 | 0.44 |
| 4:D:395:ARG:O | 4:D:399:GLU:HG3 | 2.17 | 0.44 |
| 4:D:566:THR:HG23 | 4:D:568:GLN:HE22 | 1.83 | 0.44 |
| 4:D:655:ILE:HG22 | 4:D:656:GLN:N | 2.33 | 0.44 |
| 4:D:145:ILE:N | 4:D:145:ILE:HD12 | 2.32 | 0.44 |
| 4:D:278:TRP:HH2 | 4:D:294:LEU:HB3 | 1.81 | 0.44 |
| 4:D:873:ARG:HH11 | 4:D:873:ARG:HG3 | 1.83 | 0.44 |
| 4:D:9:ASN:OD1 | 4:D:48:GLU:OE2 | 2.35 | 0.44 |
| 4:D:655:ILE:HG23 | 4:D:659:ILE:CD1 | 2.48 | 0.44 |
| 4:D:727:TRP:CD1 | 5:D:1037:HOH:O | 2.56 | 0.44 |
| 1:T:118:DC:H2” | 1:T:117:DG:OP2 | 2.18 | 0.44 |
| 1:T:123:DG:H1’ | 1:T:122:DC:H5” | 1.99 | 0.44 |
| 4:D:281:ILE:HD13 | 4:D:281:ILE:N | 2.30 | 0.44 |
| 4:D:208:ASP:O | 4:D:210:ILE:N | 2.51 | 0.44 |
| 4:D:221:ILE:H | 4:D:221:ILE:CD1 | 2.30 | 0.44 |
| 4:D:33:ALA:CB | 5:D:937:HOH:O | 2.64 | 0.44 |
| 2:N:24:DG:H2” | 2:N:25:DC:C6 | 2.53 | 0.44 |
| 4:D:153:ARG:C | 4:D:155:ARG:N | 2.71 | 0.43 |
| 4:D:78:LEU:HB3 | 4:D:79:PRO:HD3 | 2.00 | 0.43 |
| 4:D:109:ILE:CD1 | 4:D:109:ILE:H | 2.30 | 0.43 |
| 4:D:275:PRO:HD3 | 4:D:415:TRP:HB3 | 2.00 | 0.43 |
| 4:D:452:ILE:HG23 | 4:D:453:GLY:N | 2.32 | 0.43 |
| 4:D:661:SER:C | 4:D:663:LYS:H | 2.21 | 0.43 |
| 4:D:117:ILE:CG2 | 4:D:145:ILE:HD12 | 2.46 | 0.43 |
| 4:D:353:PRO:HG3 | 4:D:394:ARG:CB | 2.47 | 0.43 |
| 4:D:846:TYR:HA | 4:D:849:PHE:CE1 | 2.53 | 0.43 |
| 4:D:157:LEU:O | 4:D:158:GLU:C | 2.55 | 0.43 |
| 4:D:157:LEU:CG | 4:D:196:LEU:HD12 | 2.48 | 0.43 |
| 4:D:356:GLU:HG2 | 4:D:357:ARG:N | 2.33 | 0.43 |
| 4:D:652:GLU:OE2 | 5:D:1028:HOH:O | 2.21 | 0.43 |
| 4:D:744:GLN:HE21 | 4:D:758:GLN:NE2 | 2.17 | 0.43 |
| 4:D:39:LEU:HD12 | 4:D:39:LEU:HA | 1.73 | 0.43 |
| 4:D:109:ILE:HD13 | 4:D:114:VAL:HG22 | 2.00 | 0.43 |
| 4:D:180:LYS:O | 4:D:184:GLN:HG3 | 2.18 | 0.43 |

*Continued on next page...*

*Continued from previous page...*

| Atom-1 | Atom-2 | Interatomic distance (Å) | Clash overlap (Å) |
|---|---|---|---|
| 4:D:17:ALA:O | 4:D:21:PHE:HB2 | 2.18 | 0.43 |
| 4:D:386:ARG:CG | 5:D:933:HOH:O | 2.34 | 0.43 |
| 4:D:632:ARG:HE | 4:D:632:ARG:HA | 1.84 | 0.42 |
| 2:N:27:DC:C2' | 2:N:28:DG:H5" | 2.49 | 0.42 |
| 1:T:119:DT:H4' | 5:T:139:HOH:O | 2.15 | 0.42 |
| 4:D:204:TRP:O | 4:D:205:HIS:CB | 2.66 | 0.42 |
| 4:D:324:GLN:CD | 5:D:928:HOH:O | 2.53 | 0.42 |
| 4:D:439:MET:O | 4:D:443:LEU:HD22 | 2.18 | 0.42 |
| 4:D:579:ASN:O | 4:D:583:GLN:HG2 | 2.18 | 0.42 |
| 4:D:582:LEU:HB3 | 4:D:621:LEU:HD21 | 2.01 | 0.42 |
| 4:D:612:GLY:O | 4:D:615:ALA:N | 2.52 | 0.42 |
| 4:D:160:LYS:HA | 4:D:160:LYS:NZ | 2.34 | 0.42 |
| 4:D:463:HIS:CB | 4:D:534:LEU:HD22 | 2.48 | 0.42 |
| 4:D:593:GLU:CG | 4:D:594:VAL:N | 2.82 | 0.42 |
| 4:D:138:ALA:O | 4:D:213:GLY:HA3 | 2.19 | 0.42 |
| 4:D:302:LYS:HG2 | 4:D:303:LYS:N | 2.35 | 0.42 |
| 4:D:464:GLY:CA | 5:D:1036:HOH:O | 2.44 | 0.42 |
| 4:D:166:VAL:HG12 | 4:D:166:VAL:O | 2.20 | 0.42 |
| 4:D:575:ALA:O | 4:D:578:VAL:HB | 2.19 | 0.42 |
| 4:D:78:LEU:HA | 4:D:119:ILE:HD11 | 2.02 | 0.42 |
| 4:D:32:LEU:O | 4:D:33:ALA:C | 2.58 | 0.42 |
| 4:D:64:VAL:HG21 | 4:D:127:THR:OG1 | 2.20 | 0.42 |
| 4:D:154:ILE:HD11 | 4:D:183:MET:HE1 | 1.99 | 0.42 |
| 4:D:84:ARG:HB2 | 4:D:223:SER:HB3 | 2.00 | 0.42 |
| 4:D:130:ASP:C | 4:D:132:THR:N | 2.71 | 0.42 |
| 4:D:729:THR:OG1 | 4:D:733:PHE:HB3 | 2.20 | 0.42 |
| 4:D:758:GLN:HA | 4:D:759:PRO:HD3 | 1.85 | 0.42 |
| 4:D:729:THR:HB | 4:D:789:SER:HB2 | 2.02 | 0.42 |
| 2:N:16:DT:H2" | 2:N:17:DA:O5' | 2.20 | 0.42 |
| 2:N:26:DA:H2" | 2:N:27:DC:O5' | 2.19 | 0.42 |
| 1:T:128:DC:H42 | 2:N:27:DC:N4 | 2.17 | 0.42 |
| 4:D:65:ALA:HB2 | 4:D:123:LEU:HD23 | 2.01 | 0.42 |
| 4:D:735:VAL:C | 5:D:1012:HOH:O | 2.57 | 0.42 |
| 4:D:446:LEU:HD13 | 4:D:806:SER:HB3 | 2.02 | 0.42 |
| 2:N:29:DG:H2" | 2:N:30:DC:H5 | 1.85 | 0.42 |
| 4:D:537:ASP:CG | 5:D:984:HOH:O | 2.55 | 0.42 |
| 4:D:611:LEU:HD22 | 4:D:615:ALA:CB | 2.48 | 0.42 |
| 4:D:698:TRP:HZ3 | 4:D:845:PHE:HD2 | 1.68 | 0.42 |
| 4:D:828:VAL:HG21 | 4:D:883:ALA:HA | 2.01 | 0.42 |
| 4:D:17:ALA:C | 4:D:20:PRO:HD2 | 2.40 | 0.41 |
| 4:D:330:ILE:HD11 | 4:D:408:PHE:HB2 | 2.02 | 0.41 |

*Continued on next page...*

*Continued from previous page...*

| Atom-1 | Atom-2 | Interatomic distance (Å) | Clash overlap (Å) |
|---|---|---|---|
| 4:D:63:GLU:O | 4:D:66:ASP:HB2 | 2.19 | 0.41 |
| 4:D:141:ILE:HG23 | 4:D:145:ILE:HD11 | 2.02 | 0.41 |
| 4:D:157:LEU:HG | 4:D:158:GLU:N | 2.36 | 0.41 |
| 4:D:221:ILE:HD13 | 4:D:221:ILE:H | 1.85 | 0.41 |
| 4:D:41:HIS:CE1 | 4:D:45:GLU:OE2 | 2.72 | 0.41 |
| 4:D:86:ASN:HA | 4:D:86:ASN:HD22 | 1.69 | 0.41 |
| 4:D:208:ASP:C | 4:D:210:ILE:N | 2.74 | 0.41 |
| 4:D:749:LEU:HD23 | 4:D:749:LEU:HA | 1.89 | 0.41 |
| 4:D:790:HIS:CD2 | 5:D:904:HOH:O | 2.70 | 0.41 |
| 4:D:869:ASN:N | 5:D:893:HOH:O | 2.15 | 0.41 |
| 4:D:16:LEU:HD21 | 4:D:20:PRO:HB2 | 2.02 | 0.41 |
| 4:D:560:ASN:HA | 5:D:938:HOH:O | 2.19 | 0.41 |
| 4:D:690:VAL:HG22 | 5:D:972:HOH:O | 2.20 | 0.41 |
| 4:D:710:VAL:HG12 | 4:D:720:ARG:HB3 | 2.02 | 0.41 |
| 4:D:561:LEU:HD12 | 4:D:561:LEU:HA | 1.88 | 0.41 |
| 4:D:605:ILE:O | 4:D:606:SER:HB3 | 2.21 | 0.41 |
| 4:D:744:GLN:HB3 | 4:D:757:LEU:O | 2.21 | 0.41 |
| 2:N:27:DC:H2” | 2:N:28:DG:OP1 | 2.21 | 0.41 |
| 4:D:333:LYS:HB3 | 4:D:516:PHE:CE1 | 2.56 | 0.41 |
| 4:D:608:LYS:HG3 | 4:D:609:VAL:N | 2.36 | 0.41 |
| 4:D:71:LYS:N | 4:D:72:PRO:HD2 | 2.36 | 0.41 |
| 1:T:127:DG:N7 | 5:T:134:HOH:O | 2.50 | 0.41 |
| 4:D:378:LYS:HD2 | 4:D:379:ARG:H | 1.77 | 0.41 |
| 4:D:550:LEU:HD11 | 4:D:695:ALA:HB2 | 2.02 | 0.41 |
| 4:D:828:VAL:CG2 | 4:D:883:ALA:HA | 2.50 | 0.41 |
| 4:D:131:ASN:C | 4:D:133:THR:H | 2.24 | 0.41 |
| 4:D:142:GLY:HA3 | 5:D:924:HOH:O | 2.21 | 0.41 |
| 4:D:139:SER:O | 4:D:143:ARG:HG2 | 2.20 | 0.41 |
| 4:D:161:HIS:CE1 | 4:D:165:ASN:HB2 | 2.55 | 0.41 |
| 2:N:17:DA:H2’ | 4:D:173:ARG:NH2 | 2.35 | 0.41 |
| 4:D:551:ARG:HD3 | 4:D:551:ARG:HA | 1.84 | 0.41 |
| 4:D:88:TRP:CZ2 | 4:D:215:ARG:HD3 | 2.55 | 0.41 |
| 4:D:285:GLY:CA | 5:D:928:HOH:O | 2.35 | 0.41 |
| 4:D:42:GLU:CD | 5:D:1035:HOH:O | 2.59 | 0.41 |
| 4:D:595:VAL:HG23 | 4:D:608:LYS:H | 1.84 | 0.41 |
| 4:D:622:ALA:O | 4:D:666:MET:HG2 | 2.21 | 0.41 |
| 4:D:360:LEU:CD2 | 5:D:917:HOH:O | 2.31 | 0.41 |
| 4:D:404:GLN:NE2 | 4:D:404:GLN:HA | 2.36 | 0.41 |
| 4:D:577:LYS:HD3 | 4:D:687:VAL:HG21 | 2.03 | 0.41 |
| 4:D:857:GLN:NE2 | 4:D:859:ASP:HB2 | 2.34 | 0.41 |
| 4:D:36:GLN:HE21 | 4:D:40:GLU:CG | 2.34 | 0.40 |

*Continued on next page...*

*Continued from previous page...*

| Atom-1 | Atom-2 | Interatomic distance (Å) | Clash overlap (Å) |
|---|---|---|---|
| 4:D:817:ILE:O | 4:D:817:ILE:HG13 | 2.21 | 0.40 |
| 4:D:330:ILE:CD1 | 4:D:330:ILE:H | 2.34 | 0.40 |
| 1:T:125:DG:H2" | 1:T:124:DC:OP2 | 2.20 | 0.40 |
| 4:D:590:THR:CG2 | 4:D:591:ASP:N | 2.83 | 0.40 |
| 4:D:647:ARG:CD | 5:D:913:HOH:O | 2.29 | 0.40 |
| 4:D:74:ILE:HB | 5:D:1034:HOH:O | 2.21 | 0.40 |
| 4:D:765:LYS:C | 4:D:765:LYS:HZ2 | 2.24 | 0.40 |

There are no symmetry-related clashes.

## 5.3 Torsion angles (i)

### 5.3.1 Protein backbone (i)

In the following table, the Percentiles column shows the percent Ramachandran outliers of the chain as a percentile score with respect to all X-ray entries followed by that with respect to entries of similar resolution.

The Analysed column shows the number of residues for which the backbone conformation was analysed, and the total number of residues.

| Mol | Chain | Analysed | Favoured | Allowed | Outliers | Percentiles |
|---|---|---|---|---|---|---|
| 4 | D | 857/883 (97%) | 736 (86%) | 86 (10%) | 35 (4%) | 3 1 |

All (35) Ramachandran outliers are listed below:

| Mol | Chain | Res | Type |
|---|---|---|---|
| 4 | D | 16 | LEU |
| 4 | D | 158 | GLU |
| 4 | D | 302 | LYS |
| 4 | D | 303 | LYS |
| 4 | D | 353 | PRO |
| 4 | D | 588 | ASN |
| 4 | D | 592 | ASN |
| 4 | D | 655 | ILE |
| 4 | D | 852 | GLN |
| 4 | D | 854 | HIS |
| 4 | D | 855 | GLU |
| 4 | D | 61 | ALA |
| 4 | D | 203 | SER |
| 4 | D | 206 | LYS |

*Continued on next page...*

*Continued from previous page...*

| Mol | Chain | Res | Type |
|---|---|---|---|
| 4 | D | 209 | SER |
| 4 | D | 290 | GLY |
| 4 | D | 358 | GLU |
| 4 | D | 404 | GLN |
| 4 | D | 595 | VAL |
| 4 | D | 602 | THR |
| 4 | D | 606 | SER |
| 4 | D | 767 | SER |
| 4 | D | 851 | ASP |
| 4 | D | 198 | GLY |
| 4 | D | 376 | ALA |
| 4 | D | 853 | LEU |
| 4 | D | 30 | GLU |
| 4 | D | 859 | ASP |
| 4 | D | 882 | PHE |
| 4 | D | 656 | GLN |
| 4 | D | 662 | GLY |
| 4 | D | 766 | ASP |
| 4 | D | 154 | ILE |
| 4 | D | 603 | GLY |
| 4 | D | 664 | GLY |

### 5.3.2 Protein sidechains (i)

In the following table, the Percentiles column shows the percent sidechain outliers of the chain as a percentile score with respect to all X-ray entries followed by that with respect to entries of similar resolution.

The Analysed column shows the number of residues for which the sidechain conformation was analysed, and the total number of residues.

| Mol | Chain | Analysed | Rotameric | Outliers | Percentiles | |
|---|---|---|---|---|---|---|
| 4 | D | 711/729 (98%) | 659 (93%) | 52 (7%) | 14 | 11 |

All (52) residues with a non-rotameric sidechain are listed below:

| Mol | Chain | Res | Type |
|---|---|---|---|
| 4 | D | 27 | HIS |
| 4 | D | 50 | ARG |
| 4 | D | 73 | LEU |
| 4 | D | 91 | GLU |
| 4 | D | 107 | GLN |

*Continued on next page...*

*Continued from previous page...*

| Mol | Chain | Res | Type |
|---|---|---|---|
| 4 | D | 109 | ILE |
| 4 | D | 120 | LYS |
| 4 | D | 122 | THR |
| 4 | D | 131 | ASN |
| 4 | D | 160 | LYS |
| 4 | D | 170 | LEU |
| 4 | D | 180 | LYS |
| 4 | D | 221 | ILE |
| 4 | D | 261 | LEU |
| 4 | D | 281 | ILE |
| 4 | D | 292 | ARG |
| 4 | D | 296 | LEU |
| 4 | D | 335 | LEU |
| 4 | D | 337 | VAL |
| 4 | D | 340 | VAL |
| 4 | D | 346 | HIS |
| 4 | D | 353 | PRO |
| 4 | D | 355 | ILE |
| 4 | D | 358 | GLU |
| 4 | D | 360 | LEU |
| 4 | D | 362 | MET |
| 4 | D | 378 | LYS |
| 4 | D | 402 | LEU |
| 4 | D | 433 | ASN |
| 4 | D | 443 | LEU |
| 4 | D | 514 | PHE |
| 4 | D | 561 | LEU |
| 4 | D | 577 | LYS |
| 4 | D | 616 | LEU |
| 4 | D | 666 | MET |
| 4 | D | 672 | GLN |
| 4 | D | 686 | SER |
| 4 | D | 741 | LYS |
| 4 | D | 743 | ILE |
| 4 | D | 754 | GLN |
| 4 | D | 757 | LEU |
| 4 | D | 765 | LYS |
| 4 | D | 783 | VAL |
| 4 | D | 787 | ASP |
| 4 | D | 805 | GLU |
| 4 | D | 816 | THR |
| 4 | D | 834 | ASP |

*Continued on next page...*

*Continued from previous page...*

| Mol | Chain | Res | Type |
|---|---|---|---|
| 4 | D | 837 | GLU |
| 4 | D | 847 | ASP |
| 4 | D | 857 | GLN |
| 4 | D | 859 | ASP |
| 4 | D | 871 | ASN |

Some sidechains can be flipped to improve hydrogen bonding and reduce clashes. All (24) such sidechains are listed below:

| Mol | Chain | Res | Type |
|---|---|---|---|
| 4 | D | 9 | ASN |
| 4 | D | 41 | HIS |
| 4 | D | 86 | ASN |
| 4 | D | 107 | GLN |
| 4 | D | 131 | ASN |
| 4 | D | 161 | HIS |
| 4 | D | 184 | GLN |
| 4 | D | 321 | ASN |
| 4 | D | 433 | ASN |
| 4 | D | 544 | GLN |
| 4 | D | 568 | GLN |
| 4 | D | 649 | GLN |
| 4 | D | 656 | GLN |
| 4 | D | 697 | ASN |
| 4 | D | 726 | HIS |
| 4 | D | 744 | GLN |
| 4 | D | 754 | GLN |
| 4 | D | 758 | GLN |
| 4 | D | 772 | HIS |
| 4 | D | 781 | ASN |
| 4 | D | 784 | HIS |
| 4 | D | 811 | HIS |
| 4 | D | 848 | GLN |
| 4 | D | 871 | ASN |

### 5.3.3 RNA (i)

| Mol | Chain | Analysed | Backbone Outliers | Pucker Outliers |
|---|---|---|---|---|
| 3 | R | 9/10 (90%) | 1 (11%) | 0 |

All (1) RNA backbone outliers are listed below:

| Mol | Chain | Res | Type |
|---|---|---|---|
| 3 | R | 8 | C |

There are no RNA pucker outliers to report.

### 5.4 Non-standard residues in protein, DNA, RNA chains (i)

There are no non-standard protein/DNA/RNA residues in this entry.

### 5.5 Carbohydrates (i)

There are no carbohydrates in this entry.

### 5.6 Ligand geometry (i)

There are no ligands in this entry.

### 5.7 Other polymers (i)

There are no such residues in this entry.

### 5.8 Polymer linkage issues (i)

There are no chain breaks in this entry.

# 6 Fit of model and data (i)

## 6.1 Protein, DNA and RNA chains (i)

EDS was not executed - this section is therefore empty.

## 6.2 Non-standard residues in protein, DNA, RNA chains (i)

EDS was not executed - this section is therefore empty.

## 6.3 Carbohydrates (i)

EDS was not executed - this section is therefore empty.

## 6.4 Ligands (i)

EDS was not executed - this section is therefore empty.

## 6.5 Other polymers (i)

EDS was not executed - this section is therefore empty.
