# Supplementary material for: An engineered T7 RNA polymerase that produces mRNA free of immunostimulatory byproducts
Source: Nat Biotechnol. 2022 Nov 10;41(4):560–8. doi: 10.1038/s41587-022-01525-6 (PMC10110463; doi:10.1038/s41587-022-01525-6)
Supplement: Supplementary file 6 — Full validation report for PDB entry 1QLN. [file 41587_2022_1525_MOESM6_ESM.pdf]

Full wwPDB X-ray Structure Validation Report (i)

May 23, 2020 – 05:23 am BST

PDB ID	:	1QLN
Title	:	STRUCTURE OF A TRANSCRIBING T7 RNA POLYMERASE INITIATION COMPLEX
Authors	:	Cheetham, G.M.T.; Steitz, T.A.
Deposited on	:	1999-09-01
Resolution	:	2.40 Å(reported)

This is a Full wwPDB X-ray Structure Validation Report for a publicly released PDB entry.

We welcome your comments at *validation@mail.wwpdb.org*
A user guide is available at
https://www.wwpdb.org/validation/2017/XrayValidationReportHelp
with specific help available everywhere you see the (i) symbol.

The following versions of software and data (see references (i)) were used in the production of this report:

MolProbity	:	4.02b-467
Mogul	:	1.8.5 (274361), CSD as541be (2020)
Xtriage (Phenix)	:	1.13
EDS	:	2.11
Percentile statistics	:	20191225.v01 (using entries in the PDB archive December 25th 2019)
Refmac	:	5.8.0158
CCP4	:	7.0.044 (Gargrove)
Ideal geometry (proteins)	:	Engh & Huber (2001)
Ideal geometry (DNA, RNA)	:	Parkinson et al. (1996)
Validation Pipeline (wwPDB-VP)	:	2.11

1 Overall quality at a glance (i)

The following experimental techniques were used to determine the structure:
X-RAY DIFFRACTION

The reported resolution of this entry is 2.40 Å.

Percentile scores (ranging between 0-100) for global validation metrics of the entry are shown in the following graphic. The table shows the number of entries on which the scores are based.

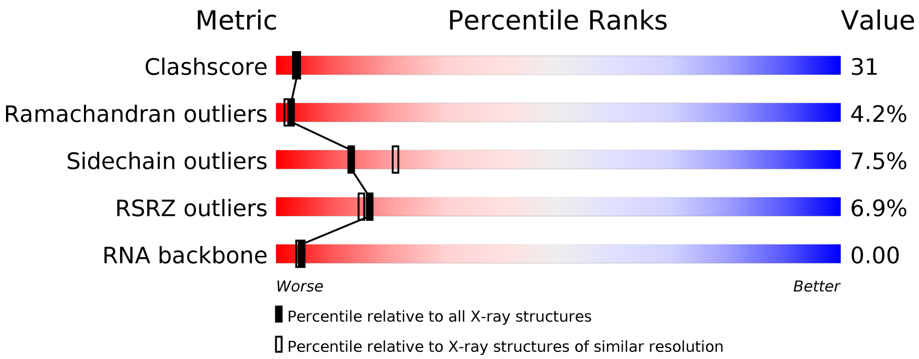

Metric	Whole archive (#Entries)	Similar resolution (#Entries, resolution range(Å))
Clashscore	141614	4398 (2.40-2.40)
Ramachandran outliers	138981	4318 (2.40-2.40)
Sidechain outliers	138945	4319 (2.40-2.40)
RSRZ outliers	127900	3811 (2.40-2.40)
RNA backbone	3102	1174 (2.80-2.00)

The table below summarises the geometric issues observed across the polymeric chains and their fit to the electron density. The red, orange, yellow and green segments on the lower bar indicate the fraction of residues that contain outliers for >=3, 2, 1 and 0 types of geometric quality criteria respectively. A grey segment represents the fraction of residues that are not modelled. The numeric value for each fraction is indicated below the corresponding segment, with a dot representing fractions <=5% The upper red bar (where present) indicates the fraction of residues that have poor fit to the electron density. The numeric value is given above the bar.

Mol	Chain	Length	Quality of chain
1	A	883	7%<br>53% 37% 8% •
2	N	17	6%<br>24% 59% 18%
3	R	3	67% 33%
4	T	22	5%<br>23% 68% 9%

2 Entry composition (i)

There are 5 unique types of molecules in this entry. The entry contains 7992 atoms, of which 0 are hydrogens and 0 are deuteriums.

In the tables below, the ZeroOcc column contains the number of atoms modelled with zero occupancy, the AltConf column contains the number of residues with at least one atom in alternate conformation and the Trace column contains the number of residues modelled with at most 2 atoms.

- Molecule 1 is a protein called BACTERIOPHAGE T7 RNA POLYMERASE.

Mol	Chain	Residues	Atoms					ZeroOcc	AltConf	Trace
1	A	862	Total 6696	C 4269	N 1167	O 1224	S 36	0	0	0

- Molecule 2 is a DNA chain called DNA (5-D(P*TP*AP*AP*TP*AP*CP*GP*AP*CP*TP *CP*AP*CP*TP*A)-3).

Mol	Chain	Residues	Atoms					ZeroOcc	AltConf	Trace
2	N	14	Total 283	C 136	N 50	O 83	P 14	0	0	0

- Molecule 3 is a RNA chain called RNA (5- R(PPP*GP*GP*G)-3).

Mol	Chain	Residues	Atoms					ZeroOcc	AltConf	Trace
3	R	3	Total 78	C 30	N 15	O 28	P 5	0	0	0

- Molecule 4 is a DNA chain called DNA (5- D (P*CP*TP*CP*CP*CP*TP*AP*TP*AP*G P*TP*GP*AP*GP*TP*CP*GP*TP* AP*TP*TP*A)-3).

Mol	Chain	Residues	Atoms					ZeroOcc	AltConf	Trace
4	T	22	Total 448	C 215	N 76	O 135	P 22	0	0	0

- Molecule 5 is water.

Mol	Chain	Residues	Atoms		ZeroOcc	AltConf
5	A	429	Total 429	O 429	0	0
5	N	21	Total 21	O 21	0	0
5	R	2	Total 2	O 2	0	0

Continued on next page...

Continued from previous page...

Mol	Chain	Residues	Atoms	ZeroOcc	AltConf
5	T	35	Total O<br>35 35	0	0

3 Residue-property plots ⓘ

These plots are drawn for all protein, RNA and DNA chains in the entry. The first graphic for a chain summarises the proportions of the various outlier classes displayed in the second graphic. The second graphic shows the sequence view annotated by issues in geometry and electron density. Residues are color-coded according to the number of geometric quality criteria for which they contain at least one outlier: green = 0, yellow = 1, orange = 2 and red = 3 or more. A red dot above a residue indicates a poor fit to the electron density (RSRZ > 2). Stretches of 2 or more consecutive residues without any outlier are shown as a green connector. Residues present in the sample, but not in the model, are shown in grey.

- Molecule 1: BACTERIOPHAGE T7 RNA POLYMERASE

Chain A:

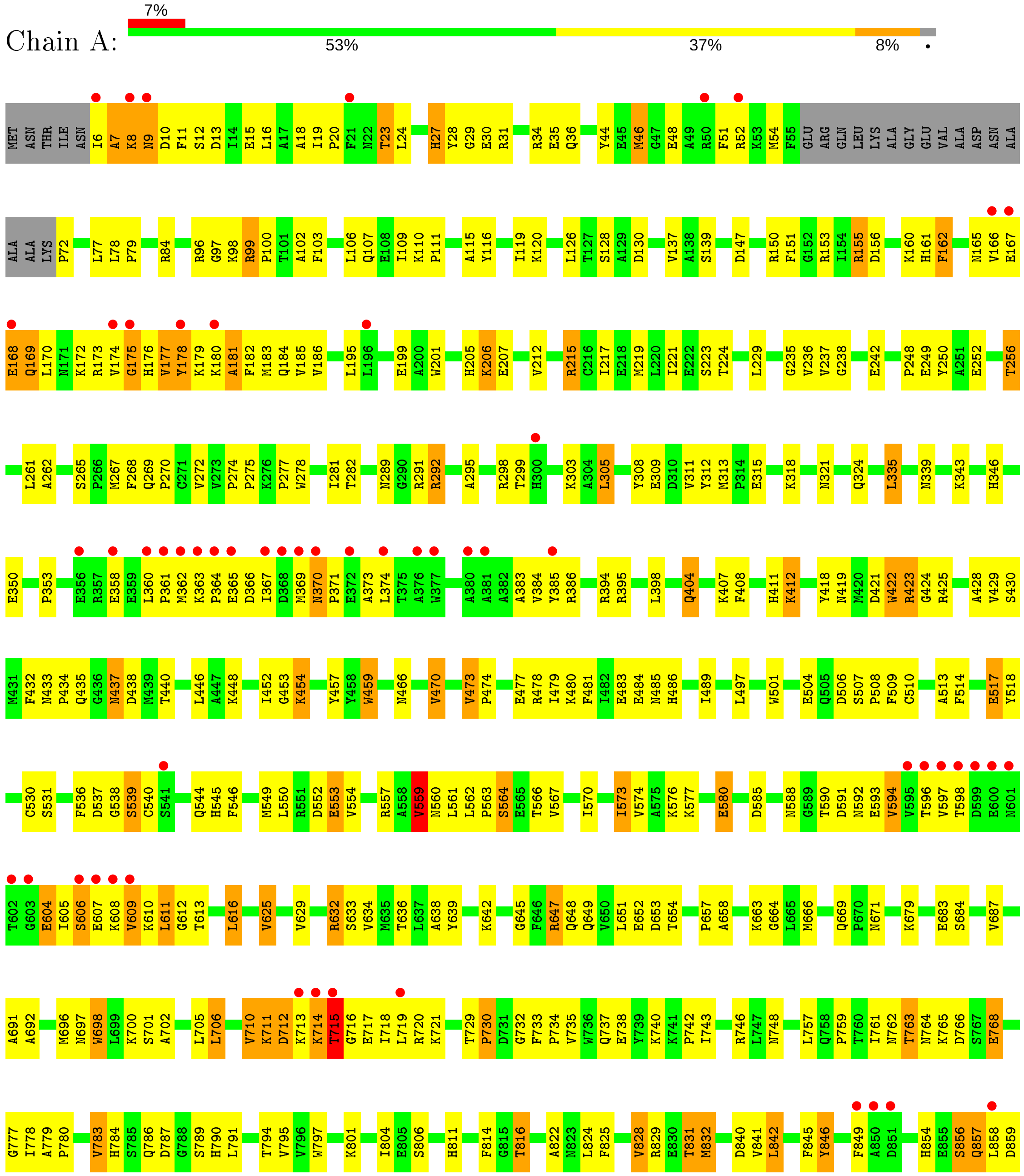

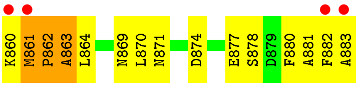

• Molecule 2: DNA (5-D(P*TP*AP*AP*TP*AP*CP*GP*AP*CP*TP*CP*AP*CP*TP*A)-3)

Chain N:

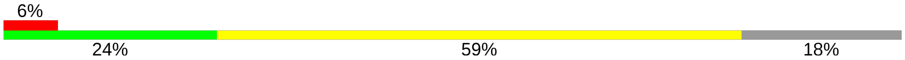

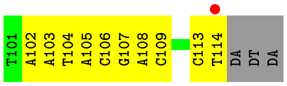

• Molecule 3: RNA (5- R(PPP*GP*GP*G)-3)

Chain R:

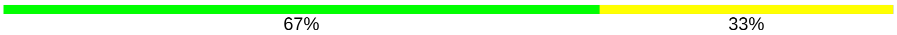

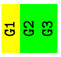

• Molecule 4: DNA (5- D (P*CP*TP*CP*CP*CP*TP*AP*TP*AP*GP*TP*GP*AP*GP*TP*C P*GP*TP* AP*TP*TP*A)-3)

Chain T:

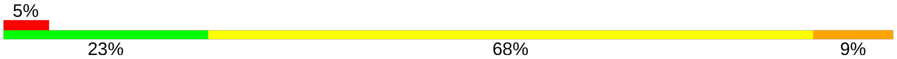

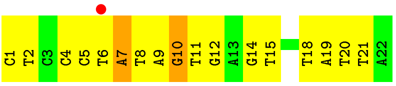

4 Data and refinement statistics (i)

Property	Value	Source
Space group	P 21 21 2	Depositor
Cell constants<br>a, b, c, α, β, γ	221.20Å 73.60Å 80.90Å<br>90.00° 90.00° 90.00°	Depositor
Resolution (Å)	40.00 – 2.40<br>39.79 – 2.40	Depositor<br>EDS
% Data completeness<br>(in resolution range)	97.8 (40.00-2.40)<br>97.5 (39.79-2.40)	Depositor<br>EDS
R_{merge}	0.08	Depositor
R_{sym}	0.08	Depositor
$< I/\sigma(I) >$ [1]	1.16 (at 2.39Å)	Xtriage
Refinement program	CNS 0.5	Depositor
R, R_{free}	0.223 , 0.267<br>0.241 , (Not available)	Depositor<br>DCC
R_{free} test set	No test flags present.	wwPDB-VP
Wilson B-factor (Å^2)	56.2	Xtriage
Anisotropy	0.225	Xtriage
Bulk solvent k_{sol}(e/Å^3), B_{sol}(Å^2)	0.34 , 47.9	EDS
L-test for twinning[2]	$< \|L\| >= 0.48$, $< L^2 >= 0.32$	Xtriage
Estimated twinning fraction	No twinning to report.	Xtriage
F_o,F_c correlation	0.93	EDS
Total number of atoms	7992	wwPDB-VP
Average B, all atoms (Å^2)	47.0	wwPDB-VP

Xtriage's analysis on translational NCS is as follows: *The largest off-origin peak in the Patterson function is 3.93% of the height of the origin peak. No significant pseudotranslation is detected.*

[1] Intensities estimated from amplitudes.

[2] Theoretical values of $< |L| >$, $< L^2 >$ for acentric reflections are 0.5, 0.333 respectively for untwinned datasets, and 0.375, 0.2 for perfectly twinned datasets.

5 Model quality (i)

5.1 Standard geometry (i)

Bond lengths and bond angles in the following residue types are not validated in this section: GTP

The Z score for a bond length (or angle) is the number of standard deviations the observed value is removed from the expected value. A bond length (or angle) with $|Z| > 5$ is considered an outlier worth inspection. RMSZ is the root-mean-square of all Z scores of the bond lengths (or angles).

Mol	Chain	Bond lengths		Bond angles	
		RMSZ	#|Z| >5	RMSZ	#|Z| >5
1	A	0.58	0/6851	0.78	4/9281 (0.0%)
2	N	0.83	0/316	0.96	0/484
3	R	0.79	0/51	0.75	0/78
4	T	0.79	0/500	0.93	0/769
All	All	0.61	0/7718	0.80	4/10612 (0.0%)

Chiral center outliers are detected by calculating the chiral volume of a chiral center and verifying if the center is modelled as a planar moiety or with the opposite hand.A planarity outlier is detected by checking planarity of atoms in a peptide group, atoms in a mainchain group or atoms of a sidechain that are expected to be planar.

Mol	Chain	#Chirality outliers	#Planarity outliers
4	T	0	2

There are no bond length outliers.

All (4) bond angle outliers are listed below:

Mol	Chain	Res	Type	Atoms	Z	Observed(^{o})	Ideal(^{o})
1	A	559	VAL	N-CA-C	-5.83	95.26	111.00
1	A	715	THR	N-CA-C	-5.71	95.60	111.00
1	A	346	HIS	N-CA-C	-5.52	96.10	111.00
1	A	181	ALA	N-CA-C	-5.41	96.41	111.00

There are no chirality outliers.

All (2) planarity outliers are listed below:

Mol	Chain	Res	Type	Group
4	T	10	DG	Sidechain
4	T	7	DA	Sidechain

5.2 Too-close contacts (i)

In the following table, the Non-H and H(model) columns list the number of non-hydrogen atoms and hydrogen atoms in the chain respectively. The H(added) column lists the number of hydrogen atoms added and optimized by MolProbity. The Clashes column lists the number of clashes within the asymmetric unit, whereas Symm-Clashes lists symmetry related clashes.

Mol	Chain	Non-H	H(model)	H(added)	Clashes	Symm-Clashes
1	A	6696	0	6561	416	0
2	N	283	0	159	13	0
3	R	78	0	34	2	0
4	T	448	0	251	42	0
5	A	429	0	0	17	0
5	N	21	0	0	0	0
5	R	2	0	0	0	0
5	T	35	0	0	2	0
All	All	7992	0	7005	451	0

The all-atom clashscore is defined as the number of clashes found per 1000 atoms (including hydrogen atoms). The all-atom clashscore for this structure is 31.

All (451) close contacts within the same asymmetric unit are listed below, sorted by their clash magnitude.

Atom-1	Atom-2	Interatomic distance (Å)	Clash overlap (Å)
1:A:713:LYS:HD3	1:A:714:LYS:H	1.05	1.11
1:A:710:VAL:HG11	1:A:720:ARG:H	1.03	1.10
1:A:710:VAL:HG11	1:A:720:ARG:N	1.65	1.08
4:T:4:DC:H2'	4:T:5:DC:O4'	1.54	1.08
1:A:298:ARG:HD2	4:T:6:DT:H5'	1.35	1.07
1:A:663:LYS:HG2	1:A:664:GLY:H	1.21	1.01
1:A:710:VAL:HG21	1:A:719:LEU:HB3	1.40	1.01
1:A:714:LYS:HA	1:A:714:LYS:HE2	1.40	1.00
1:A:560:ASN:O	1:A:881:ALA:HB2	1.62	0.99
4:T:6:DT:H4'	4:T:7:DA:H5"	1.41	0.99
1:A:155:ARG:HG3	1:A:155:ARG:HH11	1.29	0.96
1:A:713:LYS:HD3	1:A:714:LYS:N	1.81	0.94
4:T:6:DT:H4'	4:T:7:DA:C5'	1.99	0.91
1:A:298:ARG:HD3	4:T:6:DT:H2'	1.51	0.91
1:A:412:LYS:HE2	1:A:412:LYS:HA	1.53	0.90
1:A:645:GLY:HA3	4:T:2:DT:OP1	1.71	0.89
1:A:369:MET:C	1:A:371:PRO:HD2	1.92	0.88
4:T:20:DT:H2"	4:T:21:DT:H5'	1.56	0.87
1:A:710:VAL:CG1	1:A:720:ARG:H	1.86	0.87

Continued on next page...

Continued from previous page...

Atom-1	Atom-2	Interatomic distance (Å)	Clash overlap (Å)
1:A:729:THR:HG22	1:A:733:PHE:O	1.76	0.86
1:A:278:TRP:H	1:A:321:ASN:HD21	1.22	0.86
1:A:206:LYS:HE2	4:T:9:DA:N1	1.90	0.85
1:A:729:THR:HG21	1:A:733:PHE:HB3	1.60	0.83
1:A:446:LEU:HD22	1:A:806:SER:HB3	1.61	0.83
1:A:363:LYS:HD3	1:A:367:ILE:CB	2.08	0.82
1:A:281:ILE:HG23	1:A:282:THR:HG23	1.61	0.82
1:A:763:THR:CG2	1:A:765:LYS:H	1.92	0.82
1:A:763:THR:HG23	1:A:765:LYS:H	1.44	0.81
1:A:30:GLU:OE1	1:A:173:ARG:HD2	1.79	0.81
1:A:632:ARG:HH11	1:A:632:ARG:HB2	1.46	0.80
1:A:715:THR:O	1:A:717:GLU:N	2.15	0.80
1:A:730:PRO:CD	1:A:786:GLN:HE22	1.93	0.80
1:A:363:LYS:NZ	1:A:374:LEU:HD11	1.96	0.80
4:T:5:DC:H3'	4:T:7:DA:OP1	1.81	0.80
1:A:553:GLU:CD	1:A:553:GLU:H	1.86	0.79
1:A:715:THR:C	1:A:717:GLU:H	1.85	0.79
1:A:806:SER:O	1:A:816:THR:HG23	1.84	0.78
1:A:269:GLN:HE22	1:A:407:LYS:NZ	1.82	0.77
1:A:729:THR:CG2	1:A:733:PHE:HB3	2.15	0.76
1:A:109:ILE:HD12	1:A:109:ILE:H	1.52	0.75
1:A:155:ARG:NH1	1:A:155:ARG:HG3	1.99	0.75
1:A:861:MET:H	1:A:862:PRO:HD2	1.50	0.75
1:A:663:LYS:HG2	1:A:664:GLY:N	1.99	0.75
1:A:794:THR:OG1	1:A:831:THR:HG21	1.86	0.74
1:A:858:LEU:O	1:A:858:LEU:HG	1.86	0.74
1:A:398:LEU:C	1:A:398:LEU:HD23	2.08	0.74
2:N:103:DA:H2"	2:N:104:DT:H5'	1.69	0.73
1:A:298:ARG:CD	4:T:6:DT:H5'	2.18	0.73
1:A:324:GLN:HE21	1:A:418:TYR:H	1.35	0.71
2:N:108:DA:H2"	2:N:109:DC:C5'	2.20	0.71
4:T:19:DA:H1'	4:T:20:DT:H5"	1.72	0.71
1:A:170:LEU:O	1:A:174:VAL:HG23	1.90	0.71
1:A:176:HIS:O	1:A:177:VAL:O	2.10	0.70
1:A:335:LEU:HD22	1:A:339:ASN:ND2	2.06	0.70
1:A:647:ARG:HH11	1:A:671:ASN:HD21	1.40	0.69
1:A:743:ILE:O	1:A:763:THR:HB	1.93	0.69
1:A:486:HIS:HD2	1:A:518:TYR:OH	1.76	0.69
1:A:846:TYR:HA	1:A:849:PHE:CE1	2.27	0.69
1:A:229:LEU:HD11	1:A:242:GLU:HG3	1.75	0.69
1:A:710:VAL:O	1:A:710:VAL:HG13	1.92	0.68

Continued on next page...

Continued from previous page...

Atom-1	Atom-2	Interatomic distance (Å)	Clash overlap (Å)
1:A:281:ILE:HD11	1:A:305:LEU:O	1.94	0.68
1:A:299:THR:H	4:T:6:DT:H72	1.59	0.68
1:A:156:ASP:OD1	1:A:160:LYS:HE2	1.94	0.68
1:A:713:LYS:HA	1:A:713:LYS:HE2	1.76	0.68
1:A:72:PRO:HB3	1:A:262:ALA:HB3	1.76	0.67
1:A:252:GLU:O	1:A:256:THR:HB	1.94	0.67
1:A:269:GLN:HE22	1:A:407:LYS:HZ3	1.43	0.66
1:A:438:ASP:OD1	1:A:507:SER:HB3	1.96	0.66
1:A:545:HIS:O	1:A:549:MET:HG2	1.95	0.66
4:T:18:DT:H2"	4:T:19:DA:C8	2.30	0.66
1:A:169:GLN:HG2	1:A:172:LYS:HE3	1.76	0.66
1:A:761:ILE:HG13	1:A:761:ILE:O	1.94	0.65
1:A:608:LYS:O	1:A:609:VAL:HB	1.96	0.65
1:A:109:ILE:HD12	1:A:109:ILE:N	2.11	0.65
1:A:647:ARG:NH1	1:A:671:ASN:HD21	1.95	0.65
1:A:801:LYS:HD3	1:A:801:LYS:O	1.96	0.65
1:A:19:ILE:O	1:A:23:THR:HG23	1.98	0.64
1:A:221:ILE:O	1:A:224:THR:O	2.15	0.64
1:A:480:LYS:O	1:A:484:GLU:HG3	1.97	0.64
1:A:363:LYS:HZ2	1:A:374:LEU:HD11	1.61	0.64
1:A:854:HIS:CB	1:A:858:LEU:HD13	2.27	0.64
1:A:829:ARG:NH1	1:A:878:SER:O	2.31	0.64
1:A:632:ARG:HB2	1:A:632:ARG:NH1	2.12	0.64
1:A:19:ILE:HG22	1:A:195:LEU:HD21	1.79	0.64
1:A:638:ALA:O	1:A:780:PRO:HB3	1.98	0.64
1:A:437:ASN:C	1:A:437:ASN:HD22	2.00	0.63
1:A:679:LYS:O	1:A:683:GLU:HB2	1.99	0.63
1:A:730:PRO:HD3	1:A:786:GLN:HE22	1.62	0.63
2:N:108:DA:H2"	2:N:109:DC:H5'	1.81	0.63
1:A:177:VAL:O	1:A:179:LYS:HG3	1.98	0.62
1:A:371:PRO:HG2	1:A:373:ALA:CB	2.30	0.62
1:A:452:ILE:HD11	1:A:457:TYR:HA	1.82	0.62
1:A:102:ALA:HB3	1:A:215:ARG:HG2	1.82	0.61
1:A:738:GLU:O	1:A:738:GLU:HG3	2.00	0.61
1:A:308:TYR:HA	1:A:311:VAL:CG2	2.30	0.61
1:A:308:TYR:HA	1:A:311:VAL:HG23	1.82	0.61
1:A:394:ARG:HG2	1:A:394:ARG:HH11	1.64	0.61
1:A:517:GLU:HG3	1:A:530:CYS:SG	2.41	0.61
1:A:804:ILE:HG23	1:A:816:THR:HG21	1.83	0.61
1:A:182:PHE:CE2	1:A:448:LYS:HB3	2.36	0.61
1:A:19:ILE:HG22	1:A:20:PRO:N	2.16	0.61

Continued on next page...

Continued from previous page...

Atom-1	Atom-2	Interatomic distance (Å)	Clash overlap (Å)
1:A:715:THR:C	1:A:717:GLU:N	2.51	0.61
4:T:1:DC:H4’	4:T:1:DC:OP1	2.00	0.61
1:A:278:TRP:H	1:A:321:ASN:ND2	1.98	0.61
1:A:710:VAL:CG2	1:A:719:LEU:HB3	2.25	0.60
1:A:545:HIS:CD2	1:A:787:ASP:HB3	2.35	0.60
1:A:647:ARG:HG3	1:A:648:GLN:N	2.15	0.60
3:R:1:GTP:O1G	3:R:1:GTP:O3A	2.13	0.60
4:T:5:DC:H5’	4:T:7:DA:H4’	1.84	0.60
1:A:120:LYS:HE2	1:A:265:SER:O	2.00	0.60
1:A:710:VAL:HG12	1:A:720:ARG:O	2.02	0.60
2:N:108:DA:H2”	2:N:109:DC:H5”	1.83	0.60
1:A:185:VAL:HG22	1:A:186:VAL:N	2.17	0.60
1:A:18:ALA:HA	1:A:161:HIS:CD2	2.37	0.60
1:A:383:ALA:HA	1:A:386:ARG:NH1	2.17	0.60
1:A:713:LYS:O	1:A:714:LYS:C	2.39	0.60
1:A:861:MET:N	1:A:862:PRO:HD2	2.10	0.59
1:A:364:PRO:C	1:A:366:ASP:H	2.05	0.59
1:A:358:GLU:OE1	1:A:384:VAL:HG13	2.02	0.59
1:A:778:ILE:HG23	1:A:779:ALA:N	2.17	0.59
1:A:593:GLU:O	1:A:594:VAL:O	2.20	0.59
1:A:97:GLY:HA2	4:T:20:DT:O2	2.02	0.59
1:A:562:LEU:HD21	1:A:870:LEU:CD1	2.32	0.59
1:A:177:VAL:O	1:A:179:LYS:N	2.31	0.59
1:A:34:ARG:HH22	1:A:165:ASN:HD22	1.51	0.59
1:A:596:THR:O	1:A:598:THR:N	2.36	0.59
1:A:12:SER:HB3	1:A:15:GLU:HG3	1.85	0.59
1:A:369:MET:C	1:A:371:PRO:CD	2.67	0.59
1:A:501:TRP:HA	1:A:504:GLU:OE1	2.02	0.58
1:A:701:SER:HB3	1:A:861:MET:HG2	1.85	0.58
1:A:199:GLU:OE1	1:A:199:GLU:HA	2.03	0.58
1:A:278:TRP:HE1	1:A:324:GLN:HE22	1.50	0.58
1:A:485:ASN:O	1:A:489:ILE:HG13	2.03	0.58
1:A:36:GLN:NE2	1:A:272:VAL:H	2.01	0.58
1:A:298:ARG:HH11	4:T:6:DT:C2’	2.15	0.58
1:A:272:VAL:HG12	1:A:272:VAL:O	2.04	0.58
4:T:14:DG:H2”	4:T:15:DT:H5’	1.86	0.57
1:A:72:PRO:HB3	1:A:262:ALA:CB	2.34	0.57
1:A:423:ARG:NH2	1:A:784:HIS:ND1	2.51	0.57
1:A:298:ARG:HH11	4:T:6:DT:H2’	1.68	0.57
1:A:174:VAL:HG12	1:A:177:VAL:HG22	1.86	0.57
1:A:115:ALA:O	1:A:119:ILE:HG12	2.04	0.57

Continued on next page...

Continued from previous page...

Atom-1	Atom-2	Interatomic distance (Å)	Clash overlap (Å)
1:A:311:VAL:O	1:A:312:TYR:HB3	2.03	0.57
1:A:828:VAL:O	1:A:831:THR:HG22	2.05	0.57
1:A:365:GLU:C	1:A:367:ILE:H	2.08	0.57
1:A:593:GLU:HB3	1:A:610:LYS:CB	2.35	0.57
1:A:99:ARG:HD2	1:A:99:ARG:N	2.19	0.57
1:A:103:PHE:CZ	1:A:107:GLN:HG3	2.40	0.56
1:A:24:LEU:O	1:A:27:HIS:O	2.23	0.56
1:A:371:PRO:HG2	1:A:373:ALA:HB3	1.88	0.56
1:A:806:SER:O	1:A:816:THR:CG2	2.52	0.56
4:T:14:DG:H1'	4:T:15:DT:H5"	1.85	0.56
1:A:363:LYS:HZ3	1:A:374:LEU:HD11	1.68	0.56
1:A:147:ASP:OD1	1:A:292:ARG:HD2	2.04	0.56
1:A:169:GLN:HG2	1:A:172:LYS:CE	2.34	0.56
1:A:281:ILE:CG2	1:A:282:THR:HG23	2.34	0.56
1:A:816:THR:OG1	1:A:824:LEU:HD22	2.05	0.56
1:A:34:ARG:HH22	1:A:165:ASN:ND2	2.03	0.56
1:A:184:GLN:HE21	1:A:185:VAL:HG12	1.70	0.56
1:A:364:PRO:O	1:A:366:ASP:N	2.34	0.56
1:A:109:ILE:CD1	1:A:109:ILE:H	2.18	0.55
1:A:362:MET:CE	1:A:363:LYS:H	2.19	0.55
1:A:473:VAL:HG21	1:A:477:GLU:OE1	2.07	0.55
1:A:179:LYS:O	1:A:181:ALA:N	2.34	0.55
1:A:470:VAL:HG12	1:A:470:VAL:O	2.06	0.55
1:A:29:GLY:CA	1:A:175:GLY:HA3	2.37	0.55
1:A:859:ASP:O	1:A:860:LYS:HB2	2.05	0.55
4:T:4:DC:H2'	4:T:5:DC:C4'	2.37	0.55
1:A:147:ASP:O	1:A:150:ARG:O	2.25	0.55
1:A:714:LYS:HA	1:A:714:LYS:CE	2.26	0.55
2:N:108:DA:C2'	2:N:109:DC:H5"	2.36	0.55
1:A:371:PRO:C	1:A:373:ALA:H	2.09	0.54
1:A:421:ASP:HB3	4:T:6:DT:OP2	2.06	0.54
1:A:642:LYS:NZ	1:A:697:ASN:HD21	2.06	0.54
1:A:421:ASP:OD2	1:A:423:ARG:NH1	2.40	0.54
1:A:730:PRO:CD	1:A:786:GLN:NE2	2.68	0.54
1:A:763:THR:HG22	1:A:765:LYS:H	1.72	0.54
1:A:201:TRP:NE1	4:T:6:DT:H73	2.23	0.54
1:A:269:GLN:HE22	1:A:407:LYS:HZ2	1.56	0.54
1:A:298:ARG:HH11	4:T:6:DT:H3'	1.73	0.54
1:A:361:PRO:HB3	5:A:2057:HOH:O	2.08	0.54
1:A:454:LYS:C	1:A:454:LYS:HE3	2.28	0.54
1:A:178:TYR:HE1	1:A:412:LYS:HG2	1.73	0.54

Continued on next page...

Continued from previous page...

Atom-1	Atom-2	Interatomic distance (Å)	Clash overlap (Å)
1:A:862:PRO:O	1:A:863:ALA:HB3	2.07	0.53
1:A:713:LYS:CD	1:A:714:LYS:H	1.98	0.53
1:A:363:LYS:CG	1:A:364:PRO:HD2	2.39	0.53
1:A:715:THR:O	1:A:715:THR:HG22	2.08	0.53
1:A:27:HIS:O	1:A:28:TYR:HB2	2.09	0.53
1:A:713:LYS:CD	1:A:714:LYS:N	2.66	0.53
1:A:856:SER:O	1:A:857:GLN:C	2.47	0.53
1:A:235:GLY:HA2	2:N:113:DC:O2	2.09	0.53
1:A:398:LEU:HD23	1:A:398:LEU:O	2.07	0.53
1:A:23:THR:O	1:A:27:HIS:HD2	1.92	0.53
1:A:705:LEU:HD11	1:A:861:MET:HB2	1.92	0.52
1:A:669:GLN:NE2	1:A:669:GLN:HA	2.23	0.52
1:A:265:SER:HB2	5:A:2015:HOH:O	2.09	0.52
1:A:398:LEU:C	1:A:398:LEU:CD2	2.78	0.52
1:A:150:ARG:O	1:A:151:PHE:HB2	2.10	0.52
1:A:871:ASN:HB3	1:A:874:ASP:OD2	2.10	0.52
1:A:9:ASN:O	1:A:11:PHE:N	2.43	0.52
1:A:608:LYS:O	1:A:609:VAL:CB	2.58	0.52
1:A:696:MET:O	1:A:700:LYS:HG3	2.10	0.52
1:A:369:MET:O	1:A:371:PRO:HD2	2.08	0.51
1:A:295:ALA:HB1	1:A:305:LEU:HD11	1.92	0.51
1:A:367:ILE:O	1:A:374:LEU:HD22	2.11	0.51
1:A:557:ARG:O	1:A:559:VAL:O	2.27	0.51
1:A:651:LEU:HD23	1:A:651:LEU:O	2.10	0.51
1:A:652:GLU:O	1:A:657:PRO:HD3	2.10	0.51
1:A:422:TRP:C	1:A:422:TRP:CD1	2.84	0.51
1:A:433:ASN:HB2	1:A:434:PRO:CD	2.40	0.51
1:A:362:MET:HE2	1:A:363:LYS:H	1.75	0.51
1:A:466:ASN:OD1	1:A:478:ARG:NH1	2.44	0.51
1:A:459:TRP:CZ3	1:A:822:ALA:HB2	2.46	0.51
1:A:35:GLU:OE2	1:A:411:HIS:HE1	1.92	0.51
1:A:609:VAL:O	1:A:609:VAL:HG13	2.11	0.51
1:A:573:ILE:CD1	1:A:577:LYS:HE2	2.41	0.51
1:A:607:GLU:O	1:A:609:VAL:N	2.40	0.51
1:A:248:PRO:O	1:A:252:GLU:HG3	2.10	0.51
1:A:371:PRO:C	1:A:373:ALA:N	2.64	0.51
1:A:437:ASN:ND2	1:A:437:ASN:C	2.64	0.51
1:A:576:LYS:O	1:A:580:GLU:HG2	2.10	0.51
1:A:669:GLN:HE21	1:A:669:GLN:HA	1.75	0.51
1:A:861:MET:HB3	1:A:862:PRO:CD	2.41	0.51
2:N:107:DG:H2”	2:N:108:DA:H5’	1.92	0.51

Continued on next page...

Continued from previous page...

Atom-1	Atom-2	Interatomic distance (Å)	Clash overlap (Å)
1:A:166:VAL:HG13	1:A:173:ARG:HD3	1.92	0.51
1:A:609:VAL:HG21	1:A:669:GLN:HG3	1.91	0.51
1:A:8:LYS:O	1:A:9:ASN:CB	2.59	0.51
1:A:473:VAL:HG22	1:A:474:PRO:HD2	1.92	0.50
1:A:185:VAL:CG2	1:A:277:PRO:HG3	2.41	0.50
2:N:106:DC:H1'	2:N:107:DG:C8	2.46	0.50
1:A:538:GLY:HA3	1:A:883:ALA:HB3	1.94	0.50
1:A:563:PRO:HB3	1:A:877:GLU:O	2.11	0.50
1:A:605:ILE:O	1:A:606:SER:O	2.30	0.50
1:A:562:LEU:HD21	1:A:870:LEU:HD12	1.94	0.50
1:A:814:PHE:HE1	1:A:883:ALA:HB2	1.77	0.50
1:A:840:ASP:HA	5:A:2399:HOH:O	2.12	0.50
4:T:5:DC:C5'	4:T:7:DA:H4'	2.42	0.50
1:A:564:SER:HB3	1:A:566:THR:O	2.11	0.50
1:A:712:ASP:CG	1:A:713:LYS:N	2.64	0.50
1:A:370:ASN:N	1:A:371:PRO:CD	2.75	0.50
1:A:692:ALA:O	1:A:696:MET:HG3	2.11	0.50
1:A:78:LEU:HB3	1:A:79:PRO:CD	2.42	0.50
1:A:831:THR:CG2	1:A:832:MET:N	2.75	0.50
1:A:120:LYS:NZ	1:A:267:MET:SD	2.85	0.49
1:A:452:ILE:HG23	1:A:453:GLY:N	2.27	0.49
1:A:861:MET:O	1:A:862:PRO:O	2.30	0.49
1:A:553:GLU:OE1	1:A:869:ASN:N	2.45	0.49
1:A:31:ARG:HD3	1:A:176:HIS:CE1	2.47	0.49
1:A:178:TYR:HE1	1:A:412:LYS:CG	2.26	0.49
1:A:632:ARG:HH11	1:A:632:ARG:CB	2.20	0.49
1:A:562:LEU:HD21	1:A:870:LEU:HD11	1.95	0.49
1:A:173:ARG:C	1:A:175:GLY:N	2.65	0.49
1:A:175:GLY:HA2	5:A:2070:HOH:O	2.13	0.49
1:A:19:ILE:HG22	1:A:195:LEU:CD2	2.43	0.49
1:A:412:LYS:CA	1:A:412:LYS:HE2	2.35	0.49
1:A:6:ILE:O	1:A:7:ALA:C	2.50	0.49
1:A:706:LEU:HD21	1:A:849:PHE:HB2	1.93	0.49
1:A:358:GLU:OE1	1:A:384:VAL:HG22	2.14	0.48
1:A:437:ASN:ND2	1:A:440:THR:H	2.11	0.48
1:A:737:GLN:HE22	1:A:777:GLY:C	2.16	0.48
1:A:585:ASP:O	1:A:588:ASN:O	2.31	0.48
1:A:270:PRO:HD2	1:A:408:PHE:CE2	2.48	0.48
2:N:102:DA:H1'	2:N:103:DA:C8	2.48	0.48
4:T:7:DA:H2"	4:T:8:DT:O5'	2.14	0.48
1:A:236:VAL:HG12	1:A:236:VAL:O	2.12	0.48

Continued on next page...

Continued from previous page...

Atom-1	Atom-2	Interatomic distance (Å)	Clash overlap (Å)
1:A:281:ILE:HD12	1:A:309:GLU:N	2.28	0.48
1:A:369:MET:CB	1:A:371:PRO:HD2	2.43	0.48
1:A:364:PRO:C	1:A:366:ASP:N	2.67	0.48
1:A:730:PRO:CG	1:A:786:GLN:HE22	2.26	0.48
1:A:446:LEU:HB2	1:A:531:SER:O	2.14	0.48
1:A:861:MET:HE3	1:A:862:PRO:HD3	1.95	0.48
1:A:236:VAL:O	1:A:238:GLY:N	2.47	0.48
1:A:27:HIS:O	1:A:28:TYR:CB	2.60	0.48
1:A:433:ASN:HB2	1:A:434:PRO:HD3	1.96	0.48
1:A:459:TRP:HZ3	1:A:822:ALA:HB2	1.78	0.48
1:A:36:GLN:HE22	1:A:272:VAL:H	1.62	0.47
1:A:404:GLN:HA	1:A:404:GLN:HE21	1.78	0.47
1:A:861:MET:SD	1:A:862:PRO:N	2.87	0.47
1:A:711:LYS:HG3	5:A:2350:HOH:O	2.12	0.47
1:A:360:LEU:HD11	1:A:385:TYR:OH	2.14	0.47
1:A:430:SER:O	1:A:433:ASN:HB3	2.14	0.47
1:A:84:ARG:HB2	1:A:223:SER:HB3	1.96	0.47
1:A:370:ASN:O	1:A:370:ASN:OD1	2.33	0.47
1:A:268:PHE:O	1:A:430:SER:HB2	2.15	0.47
1:A:298:ARG:HH11	4:T:6:DT:C3’	2.27	0.47
1:A:536:PHE:HB3	1:A:882:PHE:HB3	1.97	0.47
1:A:651:LEU:HD23	1:A:651:LEU:C	2.35	0.47
1:A:663:LYS:HE3	1:A:666:MET:HE1	1.97	0.47
1:A:215:ARG:O	1:A:219:MET:HG3	2.15	0.47
1:A:473:VAL:CG2	1:A:477:GLU:OE1	2.63	0.47
1:A:790:HIS:CD2	1:A:831:THR:HG23	2.50	0.47
1:A:404:GLN:HG2	1:A:432:PHE:HB3	1.96	0.47
1:A:44:TYR:HB2	1:A:155:ARG:HH12	1.80	0.47
1:A:610:LYS:O	1:A:611:LEU:C	2.53	0.47
1:A:611:LEU:HB3	1:A:616:LEU:HD13	1.96	0.47
1:A:537:ASP:N	1:A:882:PHE:HD2	2.12	0.47
1:A:48:GLU:OE2	1:A:52:ARG:HB2	2.14	0.46
1:A:710:VAL:HG11	1:A:719:LEU:N	2.30	0.46
1:A:882:PHE:CD1	1:A:882:PHE:N	2.80	0.46
1:A:881:ALA:C	1:A:882:PHE:O	2.52	0.46
1:A:281:ILE:HG23	1:A:282:THR:CG2	2.40	0.46
1:A:394:ARG:NH1	1:A:394:ARG:HG2	2.30	0.46
3:R:1:GTP:O6	4:T:5:DC:O2	2.33	0.46
1:A:116:TYR:CZ	1:A:120:LYS:HD2	2.51	0.46
1:A:343:LYS:HD3	5:A:2174:HOH:O	2.14	0.46
1:A:139:SER:OG	1:A:206:LYS:HE3	2.15	0.46

Continued on next page...

Continued from previous page...

Atom-1	Atom-2	Interatomic distance (Å)	Clash overlap (Å)
1:A:311:VAL:O	1:A:312:TYR:CB	2.64	0.46
1:A:479:ILE:O	1:A:483:GLU:HG3	2.16	0.46
1:A:831:THR:HG23	1:A:832:MET:N	2.30	0.46
1:A:353:PRO:O	1:A:395:ARG:NH1	2.49	0.46
1:A:363:LYS:HG3	1:A:364:PRO:HD2	1.98	0.46
1:A:732:GLY:O	1:A:734:PRO:HD3	2.16	0.46
4:T:2:DT:OP2	4:T:2:DT:H4'	2.16	0.46
1:A:654:THR:O	1:A:658:ALA:HB2	2.16	0.45
1:A:606:SER:C	1:A:608:LYS:N	2.67	0.45
1:A:452:ILE:HG23	1:A:453:GLY:H	1.80	0.45
1:A:729:THR:HG23	1:A:733:PHE:H	1.80	0.45
1:A:791:LEU:O	1:A:795:VAL:HG23	2.17	0.45
1:A:570:ILE:O	1:A:574:VAL:HG23	2.17	0.45
1:A:77:LEU:HD11	1:A:250:TYR:CE1	2.51	0.45
1:A:729:THR:OG1	1:A:789:SER:HB3	2.17	0.45
1:A:185:VAL:CG2	1:A:186:VAL:N	2.79	0.45
1:A:663:LYS:HE3	1:A:666:MET:CE	2.46	0.45
1:A:763:THR:HG23	1:A:764:ASN:N	2.31	0.45
1:A:281:ILE:CD1	1:A:305:LEU:HD22	2.47	0.45
1:A:324:GLN:NE2	1:A:418:TYR:H	2.11	0.45
1:A:510:CYS:O	1:A:513:ALA:N	2.50	0.45
1:A:606:SER:O	1:A:607:GLU:C	2.54	0.45
1:A:649:GLN:O	1:A:653:ASP:HB2	2.17	0.45
1:A:422:TRP:O	1:A:422:TRP:CD1	2.70	0.45
1:A:710:VAL:CG1	1:A:720:ARG:N	2.55	0.45
1:A:746:ARG:HA	1:A:759:PRO:O	2.17	0.45
1:A:717:GLU:CG	1:A:718:ILE:N	2.80	0.44
1:A:215:ARG:H	1:A:215:ARG:HD2	1.82	0.44
1:A:315:GLU:OE2	1:A:318:LYS:HE2	2.16	0.44
1:A:205:HIS:O	1:A:206:LYS:C	2.55	0.44
1:A:768:GLU:HB2	5:A:2146:HOH:O	2.17	0.44
1:A:714:LYS:CA	1:A:714:LYS:HE2	2.28	0.44
1:A:590:THR:HG22	1:A:591:ASP:N	2.32	0.44
1:A:797:TRP:CH2	1:A:801:LYS:HG3	2.53	0.44
1:A:786:GLN:NE2	1:A:841:VAL:HG11	2.32	0.44
1:A:567:VAL:HG22	1:A:880:PHE:CD2	2.53	0.44
1:A:205:HIS:HB3	1:A:207:GLU:OE1	2.18	0.44
1:A:23:THR:HG21	1:A:195:LEU:HD11	1.99	0.44
1:A:592:ASN:HA	1:A:612:GLY:H	1.81	0.44
1:A:96:ARG:HD2	2:N:103:DA:H1'	1.99	0.44
1:A:778:ILE:HG23	1:A:779:ALA:H	1.83	0.44

Continued on next page...

Continued from previous page...

Atom-1	Atom-2	Interatomic distance (Å)	Clash overlap (Å)
1:A:242:GLU:HG2	1:A:757:LEU:CD1	2.48	0.43
4:T:2:DT:OP2	4:T:2:DT:C4'	2.66	0.43
1:A:742:PRO:HD2	5:A:2364:HOH:O	2.18	0.43
2:N:104:DT:H2"	2:N:105:DA:C8	2.53	0.43
1:A:249:GLU:CD	1:A:249:GLU:H	2.21	0.43
1:A:538:GLY:CA	1:A:883:ALA:HB3	2.47	0.43
1:A:669:GLN:HE21	1:A:669:GLN:CA	2.32	0.43
1:A:698:TRP:CZ2	1:A:864:LEU:HD23	2.53	0.43
1:A:825:PHE:O	1:A:829:ARG:HG2	2.18	0.43
1:A:702:ALA:O	1:A:706:LEU:HD12	2.19	0.43
1:A:546:PHE:CZ	1:A:783:VAL:HG13	2.54	0.43
1:A:590:THR:HB	1:A:613:THR:H	1.84	0.43
1:A:299:THR:H	4:T:6:DT:C7	2.29	0.43
1:A:165:ASN:HA	1:A:165:ASN:HD22	1.69	0.43
1:A:604:GLU:C	1:A:606:SER:H	2.21	0.43
1:A:849:PHE:HE2	1:A:861:MET:HE2	1.83	0.43
1:A:183:MET:HG3	5:A:2081:HOH:O	2.19	0.43
1:A:269:GLN:NE2	1:A:407:LYS:HZ3	2.13	0.43
1:A:609:VAL:HA	5:A:2303:HOH:O	2.19	0.43
1:A:738:GLU:O	1:A:740:LYS:HG3	2.19	0.43
1:A:609:VAL:HG11	1:A:669:GLN:OE1	2.19	0.43
1:A:766:ASP:HB2	5:A:2361:HOH:O	2.18	0.43
1:A:497:LEU:HA	1:A:497:LEU:HD23	1.82	0.42
1:A:552:ASP:HB2	1:A:691:ALA:HB2	2.01	0.42
1:A:168:GLU:HG3	5:A:2068:HOH:O	2.19	0.42
1:A:550:LEU:HD21	1:A:842:LEU:CD2	2.48	0.42
1:A:98:LYS:HD3	5:T:2031:HOH:O	2.19	0.42
1:A:748:ASN:HB3	2:N:106:DC:H2"	2.00	0.42
4:T:2:DT:H72	5:T:2007:HOH:O	2.18	0.42
1:A:27:HIS:CD2	1:A:27:HIS:N	2.87	0.42
1:A:470:VAL:HG21	1:A:481:PHE:CG	2.54	0.42
1:A:636:THR:HG23	5:A:2322:HOH:O	2.19	0.42
1:A:711:LYS:O	1:A:712:ASP:CB	2.66	0.42
1:A:126:LEU:HD23	1:A:126:LEU:HA	1.81	0.42
1:A:162:PHE:C	1:A:162:PHE:CD1	2.93	0.42
1:A:28:TYR:HB3	1:A:183:MET:HE1	2.00	0.42
1:A:371:PRO:HG2	1:A:373:ALA:HB2	2.01	0.42
1:A:473:VAL:HG13	1:A:477:GLU:HB2	2.01	0.42
1:A:647:ARG:NH1	1:A:671:ASN:ND2	2.65	0.42
1:A:779:ALA:N	1:A:780:PRO:HD2	2.34	0.42
1:A:215:ARG:HD2	1:A:215:ARG:N	2.34	0.42

Continued on next page...

Continued from previous page...

Atom-1	Atom-2	Interatomic distance (Å)	Clash overlap (Å)
1:A:51:PHE:O	1:A:54:MET:HG3	2.20	0.42
1:A:604:GLU:C	1:A:606:SER:N	2.72	0.42
1:A:684:SER:O	1:A:687:VAL:HG22	2.18	0.42
1:A:849:PHE:CD2	1:A:849:PHE:O	2.72	0.42
1:A:130:ASP:HB2	5:A:2051:HOH:O	2.20	0.42
1:A:155:ARG:NH1	1:A:155:ARG:CG	2.70	0.42
1:A:30:GLU:HG3	1:A:31:ARG:N	2.33	0.42
1:A:438:ASP:OD2	1:A:509:PHE:HB2	2.19	0.42
4:T:10:DG:H2”	4:T:11:DT:O5’	2.18	0.42
1:A:298:ARG:HD3	4:T:6:DT:C2’	2.35	0.42
1:A:825:PHE:CE2	1:A:829:ARG:NH2	2.88	0.42
1:A:560:ASN:C	1:A:881:ALA:HB2	2.33	0.42
1:A:639:TYR:CD2	4:T:2:DT:O2	2.73	0.42
1:A:206:LYS:HG3	1:A:762:ASN:O	2.20	0.42
1:A:242:GLU:HG2	1:A:757:LEU:HD11	2.02	0.42
1:A:634:VAL:HG22	1:A:634:VAL:O	2.20	0.42
1:A:166:VAL:HG23	1:A:167:GLU:OE2	2.20	0.41
1:A:424:GLY:O	1:A:425:ARG:C	2.59	0.41
1:A:429:VAL:O	1:A:429:VAL:CG1	2.68	0.41
1:A:573:ILE:HD12	1:A:577:LYS:HE2	2.01	0.41
4:T:11:DT:H2”	4:T:12:DG:C8	2.55	0.41
4:T:14:DG:H1’	4:T:15:DT:C5’	2.48	0.41
1:A:106:LEU:HD11	1:A:212:VAL:HG13	2.03	0.41
1:A:537:ASP:H	1:A:882:PHE:HD2	1.67	0.41
4:T:6:DT:O5’	4:T:7:DA:OP1	2.38	0.41
1:A:29:GLY:HA3	1:A:175:GLY:HA3	2.01	0.41
1:A:206:LYS:HA	1:A:206:LYS:HD3	1.82	0.41
1:A:46:MET:HG2	1:A:51:PHE:HB2	2.02	0.41
1:A:634:VAL:N	5:A:2322:HOH:O	2.53	0.41
1:A:881:ALA:O	1:A:882:PHE:C	2.58	0.41
1:A:625:VAL:HA	1:A:629:VAL:HG21	2.03	0.41
1:A:20:PRO:O	1:A:24:LEU:HB2	2.19	0.41
1:A:610:LYS:O	1:A:611:LEU:O	2.39	0.41
1:A:567:VAL:HG22	1:A:880:PHE:CG	2.55	0.41
2:N:113:DC:H4’	2:N:114:DT:OP2	2.21	0.41
1:A:573:ILE:HD11	1:A:577:LYS:HE2	2.02	0.41
1:A:611:LEU:HD12	1:A:669:GLN:HB2	2.03	0.41
1:A:110:LYS:HA	1:A:111:PRO:HD2	1.93	0.41
1:A:428:ALA:H	1:A:435:GLN:NE2	2.19	0.41
1:A:570:ILE:HA	1:A:573:ILE:HG23	2.03	0.41
1:A:698:TRP:HH2	1:A:846:TYR:CD2	2.39	0.41

Continued on next page...

Continued from previous page...

Atom-1	Atom-2	Interatomic distance (Å)	Clash overlap (Å)
1:A:842:LEU:O	1:A:845:PHE:HB3	2.20	0.41
4:T:10:DG:H2'	4:T:11:DT:H72	2.03	0.41
1:A:100:PRO:HG2	1:A:103:PHE:HB2	2.02	0.41
1:A:16:LEU:HD23	1:A:16:LEU:HA	1.82	0.41
1:A:486:HIS:CD2	1:A:518:TYR:OH	2.66	0.41
1:A:137:VAL:HG12	1:A:217:ILE:HD11	2.03	0.41
1:A:274:PRO:HA	1:A:275:PRO:HD3	1.72	0.41
1:A:825:PHE:CZ	1:A:829:ARG:NH2	2.88	0.41
1:A:173:ARG:C	1:A:175:GLY:H	2.24	0.40
1:A:862:PRO:O	1:A:863:ALA:CB	2.69	0.40
1:A:126:LEU:C	1:A:128:SER:N	2.74	0.40
1:A:421:ASP:C	1:A:421:ASP:OD1	2.60	0.40
1:A:298:ARG:HD2	4:T:6:DT:C5'	2.26	0.40
1:A:350:GLU:HG3	5:A:2177:HOH:O	2.20	0.40
1:A:729:THR:CG2	1:A:733:PHE:H	2.35	0.40
1:A:425:ARG:HD3	1:A:811:HIS:CD2	2.56	0.40
1:A:13:ASP:CG	1:A:153:ARG:HH22	2.25	0.40
1:A:206:LYS:HE2	4:T:9:DA:C2	2.55	0.40
1:A:544:GLN:HG2	1:A:561:LEU:CD1	2.52	0.40
1:A:303:LYS:HD3	5:A:2149:HOH:O	2.20	0.40
1:A:729:THR:CG2	1:A:733:PHE:CB	2.95	0.40
4:T:5:DC:O2	4:T:5:DC:H2'	2.22	0.40

There are no symmetry-related clashes.

5.3 Torsion angles (i)

5.3.1 Protein backbone (i)

In the following table, the Percentiles column shows the percent Ramachandran outliers of the chain as a percentile score with respect to all X-ray entries followed by that with respect to entries of similar resolution.

The Analysed column shows the number of residues for which the backbone conformation was analysed, and the total number of residues.

Mol	Chain	Analysed	Favoured	Allowed	Outliers	Percentiles
1	A	858/883 (97%)	734 (86%)	88 (10%)	36 (4%)	3 2

All (36) Ramachandran outliers are listed below:

Mol	Chain	Res	Type
1	A	9	ASN
1	A	168	GLU
1	A	169	GLN
1	A	175	GLY
1	A	178	TYR
1	A	237	VAL
1	A	539	SER
1	A	594	VAL
1	A	604	GLU
1	A	606	SER
1	A	609	VAL
1	A	611	LEU
1	A	712	ASP
1	A	715	THR
1	A	857	GLN
1	A	862	PRO
1	A	10	ASP
1	A	177	VAL
1	A	180	LYS
1	A	597	VAL
1	A	716	GLY
1	A	721	LYS
1	A	559	VAL
1	A	711	LYS
1	A	714	LYS
1	A	370	ASN
1	A	863	ALA
1	A	7	ALA
1	A	8	LYS
1	A	632	ARG
1	A	856	SER
1	A	861	MET
1	A	625	VAL
1	A	508	PRO
1	A	470	VAL
1	A	710	VAL

5.3.2 Protein sidechains ⓘ

In the following table, the Percentiles column shows the percent sidechain outliers of the chain as a percentile score with respect to all X-ray entries followed by that with respect to entries of similar resolution.

The Analysed column shows the number of residues for which the sidechain conformation was

analysed, and the total number of residues.

Mol	Chain	Analysed	Rotameric	Outliers	Percentiles
1	A	680/729 (93%)	629 (92%)	51 (8%)	13 21

All (51) residues with a non-rotameric sidechain are listed below:

Mol	Chain	Res	Type
1	A	23	THR
1	A	27	HIS
1	A	46	MET
1	A	99	ARG
1	A	155	ARG
1	A	162	PHE
1	A	206	LYS
1	A	215	ARG
1	A	256	THR
1	A	261	LEU
1	A	289	ASN
1	A	291	ARG
1	A	292	ARG
1	A	305	LEU
1	A	313	MET
1	A	335	LEU
1	A	404	GLN
1	A	412	LYS
1	A	419	ASN
1	A	422	TRP
1	A	423	ARG
1	A	437	ASN
1	A	454	LYS
1	A	459	TRP
1	A	473	VAL
1	A	506	ASP
1	A	514	PHE
1	A	517	GLU
1	A	539	SER
1	A	540	CYS
1	A	553	GLU
1	A	554	VAL
1	A	564	SER
1	A	573	ILE
1	A	580	GLU
1	A	616	LEU

Continued on next page...

Continued from previous page...

Mol	Chain	Res	Type
1	A	633	SER
1	A	647	ARG
1	A	698	TRP
1	A	706	LEU
1	A	730	PRO
1	A	735	VAL
1	A	763	THR
1	A	768	GLU
1	A	783	VAL
1	A	816	THR
1	A	828	VAL
1	A	831	THR
1	A	832	MET
1	A	842	LEU
1	A	846	TYR

Some sidechains can be flipped to improve hydrogen bonding and reduce clashes. All (42) such sidechains are listed below:

Mol	Chain	Res	Type
1	A	22	ASN
1	A	27	HIS
1	A	36	GLN
1	A	86	ASN
1	A	107	GLN
1	A	161	HIS
1	A	165	ASN
1	A	171	ASN
1	A	176	HIS
1	A	184	GLN
1	A	233	ASN
1	A	239	GLN
1	A	269	GLN
1	A	289	ASN
1	A	300	HIS
1	A	321	ASN
1	A	324	GLN
1	A	339	ASN
1	A	370	ASN
1	A	406	ASN
1	A	410	ASN
1	A	411	HIS
1	A	435	GLN

Continued on next page...

Continued from previous page...

Mol	Chain	Res	Type
1	A	437	ASN
1	A	463	HIS
1	A	485	ASN
1	A	486	HIS
1	A	522	GLN
1	A	523	HIS
1	A	583	GLN
1	A	669	GLN
1	A	671	ASN
1	A	697	ASN
1	A	726	HIS
1	A	737	GLN
1	A	744	GLN
1	A	764	ASN
1	A	781	ASN
1	A	786	GLN
1	A	811	HIS
1	A	823	ASN
1	A	869	ASN

5.3.3 RNA (i)

Mol	Chain	Analysed	Backbone Outliers	Pucker Outliers
3	R	1/3 (33%)	0	0

There are no RNA backbone outliers to report.

There are no RNA pucker outliers to report.

5.4 Non-standard residues in protein, DNA, RNA chains (i)

1 non-standard protein/DNA/RNA residue is modelled in this entry.

In the following table, the Counts columns list the number of bonds (or angles) for which Mogul statistics could be retrieved, the number of bonds (or angles) that are observed in the model and the number of bonds (or angles) that are defined in the Chemical Component Dictionary. The Link column lists molecule types, if any, to which the group is linked. The Z score for a bond length (or angle) is the number of standard deviations the observed value is removed from the expected value. A bond length (or angle) with $|Z| > 2$ is considered an outlier worth inspection. RMSZ is the root-mean-square of all Z scores of the bond lengths (or angles).

Mol	Type	Chain	Res	Link	Bond lengths			Bond angles		
					Counts	RMSZ	#|Z| > 2	Counts	RMSZ	#|Z| > 2
3	GTP	R	1	3,4	26,34,34	1.30	5 (19%)	33,54,54	3.60	9 (27%)

In the following table, the Chirals column lists the number of chiral outliers, the number of chiral centers analysed, the number of these observed in the model and the number defined in the Chemical Component Dictionary. Similar counts are reported in the Torsion and Rings columns. '-' means no outliers of that kind were identified.

Mol	Type	Chain	Res	Link	Chirals	Torsions	Rings
3	GTP	R	1	3,4	-	8/18/38/38	0/3/3/3

All (5) bond length outliers are listed below:

Mol	Chain	Res	Type	Atoms	Z	Observed(Å)	Ideal(Å)
3	R	1	GTP	C6-N1	3.92	1.39	1.33
3	R	1	GTP	PG-O2G	2.42	1.64	1.54
3	R	1	GTP	C8-N7	-2.16	1.30	1.34
3	R	1	GTP	PG-O3G	2.07	1.62	1.54
3	R	1	GTP	O4'-C1'	2.04	1.43	1.41

All (9) bond angle outliers are listed below:

Mol	Chain	Res	Type	Atoms	Z	Observed(^{o})	Ideal(^{o})
3	R	1	GTP	O3G-PG-O3B	14.53	153.38	104.64
3	R	1	GTP	C5-C6-N1	-8.49	111.82	123.43
3	R	1	GTP	O3B-PG-O1G	-5.81	78.97	111.19
3	R	1	GTP	C6-N1-C2	5.74	125.06	115.93
3	R	1	GTP	PA-O3A-PB	4.64	148.75	132.83
3	R	1	GTP	O2G-PG-O3B	-3.80	91.88	104.64
3	R	1	GTP	C2-N3-C4	-2.85	112.10	115.36
3	R	1	GTP	N3-C2-N1	-2.64	123.70	127.22
3	R	1	GTP	PB-O3B-PG	-2.21	125.25	132.83

There are no chirality outliers.

All (8) torsion outliers are listed below:

Mol	Chain	Res	Type	Atoms
3	R	1	GTP	C5'-O5'-PA-O3A
3	R	1	GTP	O4'-C4'-C5'-O5'
3	R	1	GTP	C3'-C4'-C5'-O5'
3	R	1	GTP	C5'-O5'-PA-O1A
3	R	1	GTP	C5'-O5'-PA-O2A

Continued on next page...

Continued from previous page...

Mol	Chain	Res	Type	Atoms
3	R	1	GTP	PG-O3B-PB-O3A
3	R	1	GTP	PG-O3B-PB-O1B
3	R	1	GTP	PB-O3A-PA-O2A

There are no ring outliers.

1 monomer is involved in 2 short contacts:

Mol	Chain	Res	Type	Clashes	Symm-Clashes
3	R	1	GTP	2	0

5.5 Carbohydrates (i)

There are no carbohydrates in this entry.

5.6 Ligand geometry (i)

There are no ligands in this entry.

5.7 Other polymers (i)

There are no such residues in this entry.

5.8 Polymer linkage issues (i)

There are no chain breaks in this entry.

6 Fit of model and data (i)

6.1 Protein, DNA and RNA chains (i)

In the following table, the column labelled '#RSRZ> 2' contains the number (and percentage) of RSRZ outliers, followed by percent RSRZ outliers for the chain as percentile scores relative to all X-ray entries and entries of similar resolution. The OWAB column contains the minimum, median, 95th percentile and maximum values of the occupancy-weighted average B-factor per residue. The column labelled 'Q< 0.9' lists the number of (and percentage) of residues with an average occupancy less than 0.9.

Mol	Chain	Analysed	<RSRZ>	#RSRZ>2			OWAB(Å^2)	Q<0.9
1	A	862/883 (97%)	0.32	60 (6%)	16	15	18, 41, 85, 105	0
2	N	14/17 (82%)	0.18	1 (7%)	16	14	31, 49, 71, 108	0
3	R	2/3 (66%)	0.16	0	100	100	59, 59, 59, 61	0
4	T	22/22 (100%)	0.19	1 (4%)	33	31	39, 51, 95, 101	0
All	All	900/925 (97%)	0.32	62 (6%)	16	15	18, 42, 86, 108	0

All (62) RSRZ outliers are listed below:

Mol	Chain	Res	Type	RSRZ
1	A	362	MET	10.1
1	A	175	GLY	8.0
1	A	599	ASP	7.9
1	A	601	ASN	6.5
1	A	600	GLU	6.5
1	A	602	THR	6.3
1	A	598	THR	6.1
2	N	114	DT	5.9
1	A	603	GLY	5.8
1	A	608	LYS	5.3
1	A	363	LYS	5.2
1	A	361	PRO	5.1
1	A	364	PRO	5.0
1	A	372	GLU	4.9
1	A	606	SER	4.9
4	T	6	DT	4.7
1	A	858	LEU	4.7
1	A	180	LYS	4.6
1	A	596	THR	4.5
1	A	166	VAL	4.4
1	A	374	LEU	4.4

Continued on next page...

Continued from previous page...

Mol	Chain	Res	Type	RSRZ
1	A	713	LYS	4.2
1	A	597	VAL	4.2
1	A	851	ASP	4.0
1	A	178	TYR	3.9
1	A	719	LEU	3.9
1	A	370	ASN	3.8
1	A	174	VAL	3.7
1	A	367	ILE	3.6
1	A	167	GLU	3.5
1	A	376	ALA	3.5
1	A	52	ARG	3.5
1	A	385	TYR	3.5
1	A	368	ASP	3.3
1	A	607	GLU	3.1
1	A	358	GLU	3.0
1	A	883	ALA	2.8
1	A	6	ILE	2.8
1	A	595	VAL	2.7
1	A	369	MET	2.7
1	A	8	LYS	2.7
1	A	196	LEU	2.6
1	A	609	VAL	2.6
1	A	380	ALA	2.5
1	A	715	THR	2.5
1	A	381	ALA	2.5
1	A	714	LYS	2.5
1	A	9	ASN	2.3
1	A	850	ALA	2.3
1	A	168	GLU	2.3
1	A	860	LYS	2.3
1	A	377	TRP	2.3
1	A	21	PHE	2.3
1	A	365	GLU	2.2
1	A	861	MET	2.2
1	A	300	HIS	2.2
1	A	541	SER	2.2
1	A	360	LEU	2.1
1	A	50	ARG	2.1
1	A	849	PHE	2.1
1	A	356	GLU	2.1
1	A	882	PHE	2.0

6.2 Non-standard residues in protein, DNA, RNA chains (i)

In the following table, the Atoms column lists the number of modelled atoms in the group and the number defined in the chemical component dictionary. The B-factors column lists the minimum, median, 95th percentile and maximum values of B factors of atoms in the group. The column labelled 'Q< 0.9' lists the number of atoms with occupancy less than 0.9.

Mol	Type	Chain	Res	Atoms	RSCC	RSR	B-factors(Å^2)	Q<0.9
3	GTP	R	1	32/32	0.56	0.32	81,92,115,115	0

6.3 Carbohydrates (i)

There are no carbohydrates in this entry.

6.4 Ligands (i)

There are no ligands in this entry.

6.5 Other polymers (i)

There are no such residues in this entry.
