# Supplementary material for: An engineered T7 RNA polymerase that produces mRNA free of immunostimulatory byproducts
Source: Nat Biotechnol. 2022 Nov 10;41(4):560–8. doi: 10.1038/s41587-022-01525-6 (PMC10110463; doi:10.1038/s41587-022-01525-6)
Supplement: Supplementary file 7 — Full validation report for PDB entry 2PI4. [file 41587_2022_1525_MOESM7_ESM.pdf]

Full wwPDB X-ray Structure Validation Report (i)

May 17, 2020 – 11:09 pm BST

PDB ID	:	2PI4
Title	:	T7RNAP complexed with a phi10 protein and initiating GTPs.
Authors	:	Kennedy, W.P.; Momand, J.R.; Yin, Y.W.
Deposited on	:	2007-04-12
Resolution	:	2.50 Å(reported)

This is a Full wwPDB X-ray Structure Validation Report for a publicly released PDB entry.

We welcome your comments at *validation@mail.wwpdb.org*
A user guide is available at
https://www.wwpdb.org/validation/2017/XrayValidationReportHelp
with specific help available everywhere you see the (i) symbol.

The following versions of software and data (see references (i)) were used in the production of this report:

MolProbity	:	4.02b-467
Mogul	:	1.8.5 (274361), CSD as541be (2020)
Xtriage (Phenix)	:	1.13
EDS	:	2.11
buster-report	:	1.1.7 (2018)
Percentile statistics	:	20191225.v01 (using entries in the PDB archive December 25th 2019)
Refmac	:	5.8.0158
CCP4	:	7.0.044 (Gargrove)
Ideal geometry (proteins)	:	Engh & Huber (2001)
Ideal geometry (DNA, RNA)	:	Parkinson et al. (1996)
Validation Pipeline (wwPDB-VP)	:	2.11

1 Overall quality at a glance (i)

The following experimental techniques were used to determine the structure:
X-RAY DIFFRACTION

The reported resolution of this entry is 2.50 Å.

Percentile scores (ranging between 0-100) for global validation metrics of the entry are shown in the following graphic. The table shows the number of entries on which the scores are based.

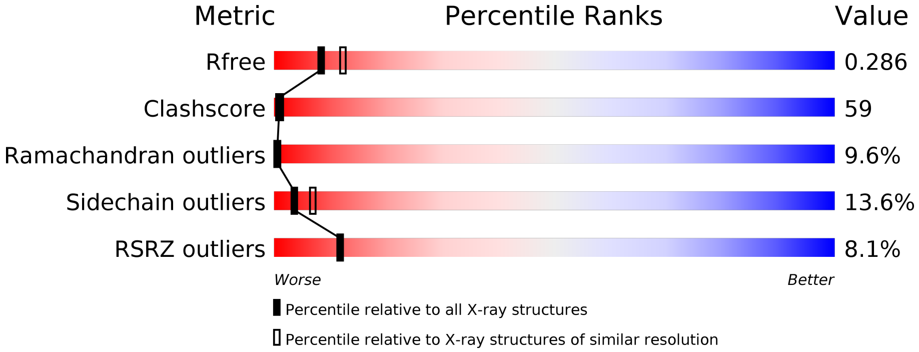

Metric	Whole archive (#Entries)	Similar resolution (#Entries, resolution range(Å))
R_{free}	130704	4661 (2.50-2.50)
Clashscore	141614	5346 (2.50-2.50)
Ramachandran outliers	138981	5231 (2.50-2.50)
Sidechain outliers	138945	5233 (2.50-2.50)
RSRZ outliers	127900	4559 (2.50-2.50)

The table below summarises the geometric issues observed across the polymeric chains and their fit to the electron density. The red, orange, yellow and green segments on the lower bar indicate the fraction of residues that contain outliers for >=3, 2, 1 and 0 types of geometric quality criteria respectively. A grey segment represents the fraction of residues that are not modelled. The numeric value for each fraction is indicated below the corresponding segment, with a dot representing fractions <=5% The upper red bar (where present) indicates the fraction of residues that have poor fit to the electron density. The numeric value is given above the bar.

Mol	Chain	Length	Quality of chain
1	T	22	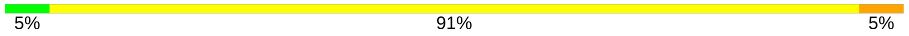
2	P	14	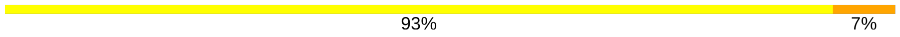
3	A	878	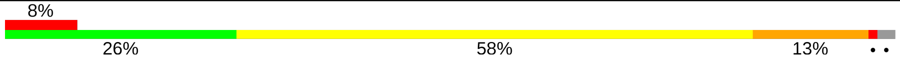

The following table lists non-polymeric compounds, carbohydrate monomers and non-standard residues in protein, DNA, RNA chains that are outliers for geometric or electron-density-fit criteria:

Mol	Type	Chain	Res	Chirality	Geometry	Clashes	Electron density
4	MG	A	885	-	-	-	X

2 Entry composition (i)

There are 6 unique types of molecules in this entry. The entry contains 7722 atoms, of which 0 are hydrogens and 0 are deuteriums.

In the tables below, the ZeroOcc column contains the number of atoms modelled with zero occupancy, the AltConf column contains the number of residues with at least one atom in alternate conformation and the Trace column contains the number of residues modelled with at most 2 atoms.

- Molecule 1 is a DNA chain called 5'-D(*CP*TP*TP*CP*CP*TP*AP*TP*AP*GP*TP*GP *AP*GP*TP*CP*GP*TP*AP*TP*TP*A)-3'.

Mol	Chain	Residues	Atoms					ZeroOcc	AltConf	Trace
1	T	22	Total<br>446	C<br>216	N<br>75	O<br>134	P<br>21	0	0	0

- Molecule 2 is a DNA chain called 5'-D(*TP*AP*AP*TP*AP*CP*GP*AP*CP*TP*CP*AP *CP*T)-3'.

Mol	Chain	Residues	Atoms					ZeroOcc	AltConf	Trace
2	P	14	Total<br>280	C<br>136	N<br>50	O<br>81	P<br>13	0	0	0

- Molecule 3 is a protein called DNA-directed RNA polymerase.

Mol	Chain	Residues	Atoms					ZeroOcc	AltConf	Trace
3	A	862	Total<br>6803	C<br>4334	N<br>1178	O<br>1254	S<br>37	0	0	0

- Molecule 4 is MAGNESIUM ION (three-letter code: MG) (formula: Mg).

Mol	Chain	Residues	Atoms		ZeroOcc	AltConf
4	A	2	Total<br>2	Mg<br>2	0	0

- Molecule 5 is 3'-DEOXY-GUANOSINE-5'-TRIPHOSPHATE (three-letter code: GH3) (formula: $C_{10}H_{16}N_5O_{13}P_3$).

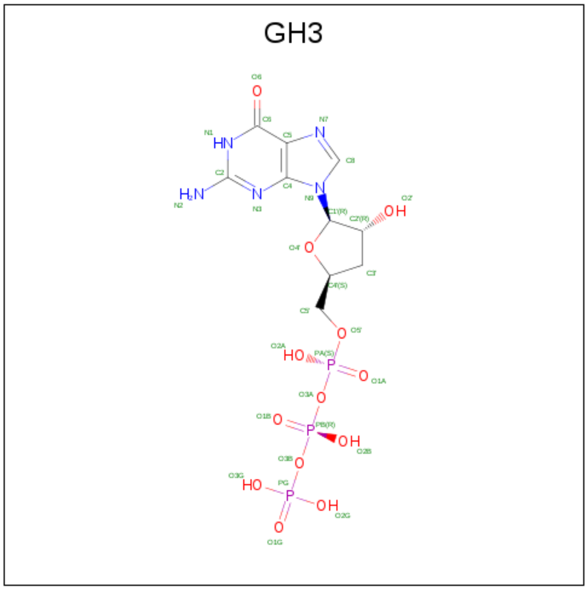

Mol	Chain	Residues	Atoms					ZeroOcc	AltConf
			Total	C	N	O	P		
5	A	1	31	10	5	13	3	0	0
5	A	1	31	10	5	13	3	0	0

- Molecule 6 is water.

Mol	Chain	Residues	Atoms		ZeroOcc	AltConf
			Total	O		
6	T	7	7	7	0	0
6	P	7	7	7	0	0
6	A	115	115	115	0	0

3 Residue-property plots (i)

These plots are drawn for all protein, RNA and DNA chains in the entry. The first graphic for a chain summarises the proportions of the various outlier classes displayed in the second graphic. The second graphic shows the sequence view annotated by issues in geometry and electron density. Residues are color-coded according to the number of geometric quality criteria for which they contain at least one outlier: green = 0, yellow = 1, orange = 2 and red = 3 or more. A red dot above a residue indicates a poor fit to the electron density (RSRZ > 2). Stretches of 2 or more consecutive residues without any outlier are shown as a green connector. Residues present in the sample, but not in the model, are shown in grey.

- Molecule 1: 5'-D(*CP*TP*TP*CP*CP*TP*AP*TP*AP*GP*TP*GP*AP*GP*TP*CP*GP*TP*AP*TP*TP*A)-3'

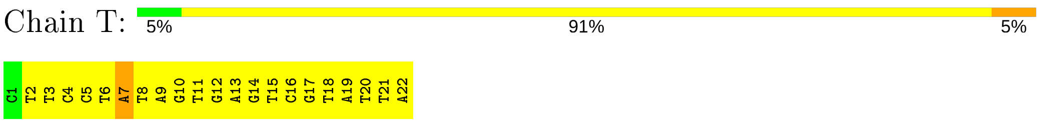

- Molecule 2: 5'-D(*TP*AP*AP*TP*AP*CP*GP*AP*CP*TP*CP*AP*CP*T)-3'

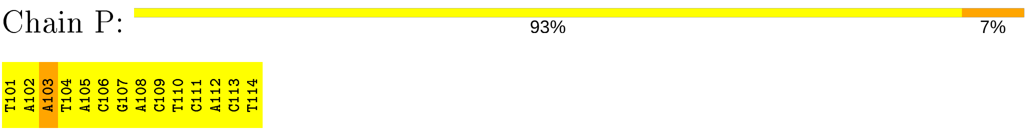

- Molecule 3: DNA-directed RNA polymerase

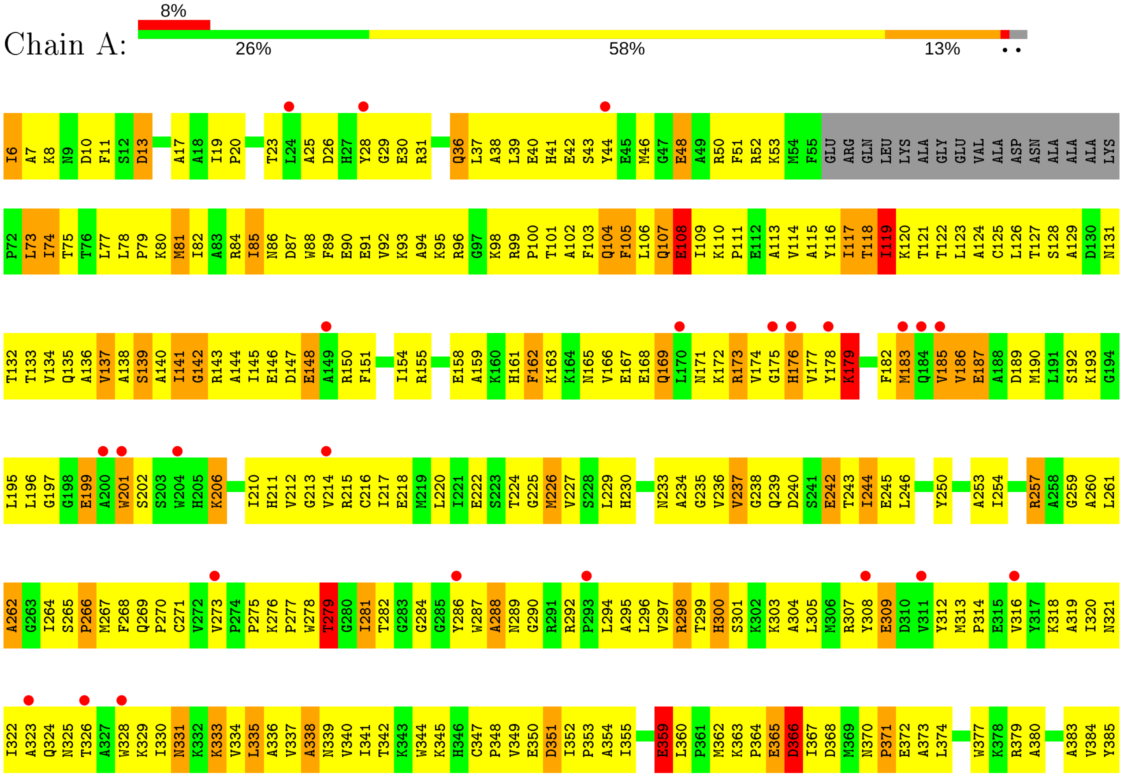

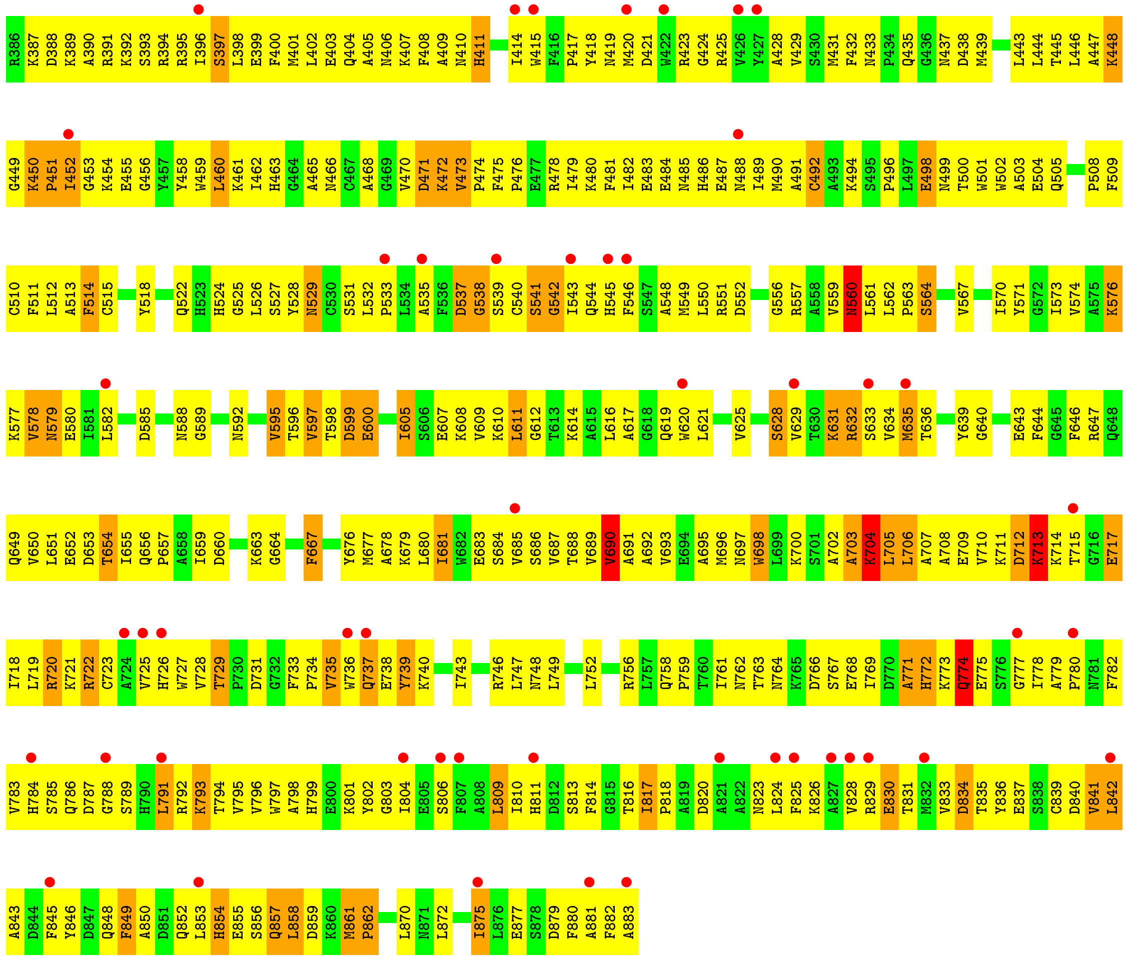
R386 K387 D388 K389 A390 R391 K392 S393 R394 R395 I396 S397 L398 E399 F400 M401 L402 E403 Q404 A405 N406 K407 F408 A409 N410 H411 I414 W415 F416 P417 Y418 N419 M420 D421 W422 R423 G424 R425 V426 Y427 A428 V429 S430 M431 F432 N433 P434 Q435 G436 N437 D438 M439 L443 L444 T445 L446 A447 K448
G449 K450 P451 I452 G453 K454 E455 G456 Y457 Y458 W459 L460 K461 I462 H463 G464 A465 N466 C467 A468 G469 V470 D471 K472 V473 P474 F475 P476 E477 R478 I479 K480 F481 I482 E483 E484 N485 H486 E487 N488 I489 M490 A491 C492 A493 K494 S495 P496 L497 E498 N499 T500 W501 W502 A503 E504 Q505 P508 F509
C510 F511 L512 A513 F514 C515 Y518 Q522 H523 H524 G525 L526 S527 Y528 N529 C530 S531 L532 P533 L534 A535 F536 D537 G538 S539 C540 S541 G542 I543 Q544 H545 F546 S547 A548 M549 L550 R551 D552 G556 R557 A558 V559 N560 L561 L562 P563 S564 V567 I570 Y571 G572 I573 V574 A575 K576
K577 V578 N579 E580 I581 L582 D585 N588 G589 N592 V595 T596 V597 T598 D599 E600 I605 S606 E607 K608 V609 K610 L611 G612 T613 K614 A615 L616 A617 G618 Q619 W620 L621 V625 S628 V629 T630 K631 R632 S633 V634 M635 T636 Y639 G640 E643 F644 G645 F646 R647 Q648
Q649 V650 L651 E652 D653 T654 I655 Q656 P657 A658 I659 D660 K663 G664 F667 Y676 M677 A678 K679 L680 I681 W682 E683 S684 V685 S686 V687 T688 V689 V690 A691 A692 V693 E694 A695 M696 N697 W698 L699 K700 S701 A702 A703 K704 L705 L706 A707 A708 E709 V710 K711 D712 K713 K714 T715 G716 E717
I718 L719 R720 K721 R722 C723 A724 V725 H726 W727 V728 T729 P730 D731 G732 F733 P734 V735 W736 Q737 E738 Y739 K740 I743 R746 L747 N748 L749 L752 R756 L757 Q758 P759 T760 I761 N762 T763 N764 K765 D766 S767 E768 I769 D770 A771 H772 K773 Q774 E775 S776 G777 I778 A779 P780 N781 F782
V783 H784 S785 Q786 D787 G788 S789 H790 L791 R792 K793 T794 V795 V796 W797 A798 H799 E800 K801 Y802 G803 I804 E805 S806 F807 A808 L809 I810 H811 D812 S813 F814 G815 T816 I817 P818 A819 D820 A821 A822 N823 L824 F825 K826 A827 V828 R829 E830 T831 M832 V833 D834 T835 Y836 E837 S838 C839 D840 V841 L842
A843 D844 F845 Y846 D847 Q848 F849 A850 D851 Q852 L853 H854 E855 S856 Q857 L858 D859 K860 M861 P862 L870 N871 L872 I875 L876 E877 S878 D879 F880 A881 F882 A883

4 Data and refinement statistics (i)

Property	Value	Source
Space group	P 21 21 2	Depositor
Cell constants<br>a, b, c, α, β, γ	224.45Å 73.79Å 80.31Å<br>90.00° 90.00° 90.00°	Depositor
Resolution (Å)	39.04 – 2.50<br>39.04 – 2.50	Depositor<br>EDS
% Data completeness<br>(in resolution range)	78.0 (39.04-2.50)<br>97.8 (39.04-2.50)	Depositor<br>EDS
R_{merge}	(Not available)	Depositor
R_{sym}	(Not available)	Depositor
$< I/\sigma(I) >$ [1]	3.91 (at 2.51Å)	Xtriage
Refinement program	CNS 1.2	Depositor
R, R_{free}	0.265 , 0.297<br>0.283 , 0.286	Depositor<br>DCC
R_{free} test set	2301 reflections (4.98%)	wwPDB-VP
Wilson B-factor (Å^2)	46.6	Xtriage
Anisotropy	0.504	Xtriage
Bulk solvent k_{sol}(e/Å^3), B_{sol}(Å^2)	0.01 , 2.6	EDS
L-test for twinning[2]	$< \|L\| > = 0.46$, $< L^2 > = 0.29$	Xtriage
Estimated twinning fraction	No twinning to report.	Xtriage
F_o,F_c correlation	0.90	EDS
Total number of atoms	7722	wwPDB-VP
Average B, all atoms (Å^2)	64.0	wwPDB-VP

Xtriage's analysis on translational NCS is as follows: *The largest off-origin peak in the Patterson function is 4.88% of the height of the origin peak. No significant pseudotranslation is detected.*

[1] Intensities estimated from amplitudes.

[2] Theoretical values of $< |L| >$, $< L^2 >$ for acentric reflections are 0.5, 0.333 respectively for untwinned datasets, and 0.375, 0.2 for perfectly twinned datasets.

5 Model quality (i)

5.1 Standard geometry (i)

Bond lengths and bond angles in the following residue types are not validated in this section: MG, GH3

The Z score for a bond length (or angle) is the number of standard deviations the observed value is removed from the expected value. A bond length (or angle) with $|Z| > 5$ is considered an outlier worth inspection. RMSZ is the root-mean-square of all Z scores of the bond lengths (or angles).

Mol	Chain	Bond lengths		Bond angles	
		RMSZ	#|Z| >5	RMSZ	#|Z| >5
1	T	0.68	0/498	0.85	0/767
2	P	0.69	0/313	0.87	0/480
3	A	0.58	1/6959 (0.0%)	0.66	0/9416
All	All	0.59	1/7770 (0.0%)	0.68	0/10663

Chiral center outliers are detected by calculating the chiral volume of a chiral center and verifying if the center is modelled as a planar moiety or with the opposite hand.A planarity outlier is detected by checking planarity of atoms in a peptide group, atoms in a mainchain group or atoms of a sidechain that are expected to be planar.

Mol	Chain	#Chirality outliers	#Planarity outliers
1	T	0	2
2	P	0	1
All	All	0	3

All (1) bond length outliers are listed below:

Mol	Chain	Res	Type	Atoms	Z	Observed(Å)	Ideal(Å)
3	A	222	GLU	CG-CD	5.41	1.60	1.51

There are no bond angle outliers.

There are no chirality outliers.

All (3) planarity outliers are listed below:

Mol	Chain	Res	Type	Group
2	P	103	DA	Sidechain
1	T	6	DT	Sidechain
1	T	7	DA	Sidechain

5.2 Too-close contacts (i)

In the following table, the Non-H and H(model) columns list the number of non-hydrogen atoms and hydrogen atoms in the chain respectively. The H(added) column lists the number of hydrogen atoms added and optimized by MolProbity. The Clashes column lists the number of clashes within the asymmetric unit, whereas Symm-Clashes lists symmetry related clashes.

Mol	Chain	Non-H	H(model)	H(added)	Clashes	Symm-Clashes
1	T	446	0	253	34	0
2	P	280	0	160	23	0
3	A	6803	0	6764	831	1
4	A	2	0	0	0	0
5	A	62	0	24	0	0
6	A	115	0	0	33	0
6	P	7	0	0	0	0
6	T	7	0	0	0	0
All	All	7722	0	7201	873	1

The all-atom clashscore is defined as the number of clashes found per 1000 atoms (including hydrogen atoms). The all-atom clashscore for this structure is 59.

All (873) close contacts within the same asymmetric unit are listed below, sorted by their clash magnitude.

Atom-1	Atom-2	Interatomic distance (Å)	Clash overlap (Å)
3:A:452:ILE:CD1	3:A:456:GLY:HA3	1.59	1.31
3:A:402:LEU:HD11	6:A:931:HOH:O	1.34	1.27
3:A:267:MET:CE	6:A:984:HOH:O	1.83	1.25
3:A:452:ILE:HD11	3:A:456:GLY:CA	1.73	1.19
3:A:304:ALA:HB1	6:A:937:HOH:O	1.45	1.14
3:A:452:ILE:HD13	3:A:818:PRO:HB2	1.19	1.10
3:A:798:ALA:HB1	3:A:804:ILE:HD12	1.34	1.09
3:A:402:LEU:CD1	6:A:931:HOH:O	1.88	1.07
3:A:578:VAL:HG22	3:A:680:LEU:HB3	1.36	1.07
1:T:14:DG:H2”	1:T:15:DT:H5’	1.26	1.06
3:A:473:VAL:HG22	3:A:474:PRO:HD2	1.40	1.03
3:A:253:ALA:HB1	3:A:257:ARG:HE	1.22	1.03
3:A:861:MET:H	3:A:862:PRO:HD2	1.22	1.03
3:A:141:ILE:HG22	3:A:145:ILE:HD11	1.37	1.02
1:T:19:DA:H2”	1:T:20:DT:H5’	1.41	1.02
3:A:693:VAL:HG12	3:A:697:ASN:HD21	1.21	1.00
2:P:112:DA:H2”	2:P:113:DC:H5”	1.44	0.99
3:A:677:MET:O	3:A:681:ILE:HG13	1.63	0.98
3:A:210:ILE:O	3:A:214:VAL:HG23	1.64	0.97

Continued on next page...

Continued from previous page...

Atom-1	Atom-2	Interatomic distance (Å)	Clash overlap (Å)
3:A:281:ILE:H	3:A:281:ILE:HD13	1.28	0.96
3:A:269:GLN:HE21	3:A:404:GLN:NE2	1.61	0.96
3:A:269:GLN:NE2	3:A:404:GLN:HE22	1.63	0.95
3:A:131:ASN:ND2	6:A:980:HOH:O	1.99	0.95
3:A:269:GLN:HE21	3:A:404:GLN:HE22	1.01	0.94
3:A:726:HIS:CD2	6:A:982:HOH:O	2.20	0.94
3:A:740:LYS:HA	3:A:769:ILE:HA	1.51	0.93
3:A:137:VAL:HG21	3:A:244:ILE:HD13	1.51	0.92
3:A:739:TYR:HB2	3:A:774:GLN:NE2	1.85	0.92
3:A:452:ILE:HD13	3:A:818:PRO:CB	1.99	0.92
3:A:557:ARG:NH2	3:A:562:LEU:HD13	1.84	0.92
3:A:579:ASN:HA	3:A:582:LEU:HD12	1.51	0.92
3:A:726:HIS:NE2	6:A:982:HOH:O	2.01	0.91
3:A:452:ILE:CD1	3:A:818:PRO:HB2	2.01	0.91
3:A:333:LYS:H	3:A:333:LYS:HD2	1.35	0.90
3:A:725:VAL:HB	3:A:737:GLN:HB2	1.53	0.90
3:A:849:PHE:HB2	3:A:853:LEU:HB2	1.52	0.90
3:A:816:THR:HG22	3:A:817:ILE:H	1.38	0.88
3:A:855:GLU:HG3	3:A:856:SER:H	1.38	0.88
3:A:123:LEU:HA	3:A:126:LEU:HD12	1.55	0.88
3:A:452:ILE:HD11	3:A:456:GLY:HA3	0.89	0.88
3:A:557:ARG:HB2	3:A:562:LEU:HD12	1.56	0.87
3:A:663:LYS:HG2	3:A:664:GLY:H	1.40	0.86
3:A:748:ASN:HD21	3:A:756:ARG:HH21	1.18	0.86
3:A:145:ILE:HD13	3:A:212:VAL:HG12	1.56	0.85
3:A:303:LYS:HD3	3:A:303:LYS:H	1.38	0.85
2:P:107:DG:H2"	2:P:108:DA:H5'	1.58	0.85
3:A:329:LYS:NZ	6:A:968:HOH:O	2.09	0.85
3:A:629:VAL:HG13	3:A:654:THR:HG21	1.57	0.85
3:A:475:PHE:O	3:A:479:ILE:HD12	1.75	0.84
3:A:261:LEU:N	3:A:261:LEU:HD12	1.93	0.84
3:A:77:LEU:H	3:A:77:LEU:HD12	1.42	0.84
3:A:120:LYS:HG2	3:A:264:ILE:HG23	1.57	0.83
3:A:560:ASN:HB3	3:A:881:ALA:HB2	1.60	0.83
3:A:711:LYS:HA	3:A:718:ILE:HG22	1.58	0.83
3:A:276:LYS:HE3	3:A:287:TRP:HA	1.60	0.83
3:A:367:ILE:HG23	3:A:368:ASP:H	1.43	0.81
3:A:227:VAL:HG12	3:A:246:LEU:HA	1.62	0.81
3:A:213:GLY:O	3:A:217:ILE:HG12	1.79	0.81
3:A:678:ALA:HA	3:A:681:ILE:HD12	1.63	0.81
3:A:236:VAL:O	3:A:240:ASP:HB2	1.81	0.81

Continued on next page...

Continued from previous page...

Atom-1	Atom-2	Interatomic distance (Å)	Clash overlap (Å)
3:A:649:GLN:O	3:A:653:ASP:HB2	1.81	0.80
3:A:861:MET:N	3:A:862:PRO:HD2	1.97	0.80
3:A:605:ILE:HD13	3:A:605:ILE:O	1.83	0.79
3:A:23:THR:HG23	6:A:1010:HOH:O	1.82	0.78
3:A:177:VAL:HB	3:A:179:LYS:HD2	1.63	0.78
3:A:261:LEU:H	3:A:261:LEU:HD12	1.46	0.78
3:A:417:PRO:HG2	3:A:429:VAL:HB	1.65	0.78
3:A:377:TRP:HA	3:A:380:ALA:HB3	1.65	0.78
3:A:448:LYS:HD3	3:A:448:LYS:N	1.99	0.77
3:A:185:VAL:HG22	3:A:186:VAL:H	1.49	0.77
3:A:576:LYS:O	3:A:580:GLU:HG3	1.84	0.77
3:A:339:ASN:HA	3:A:402:LEU:HD21	1.66	0.77
3:A:677:MET:HG3	3:A:681:ILE:HD11	1.65	0.77
3:A:452:ILE:CD1	3:A:818:PRO:CB	2.62	0.77
3:A:324:GLN:HE21	3:A:418:TYR:H	1.31	0.76
3:A:841:VAL:HG23	3:A:842:LEU:H	1.50	0.76
3:A:345:LYS:HD3	3:A:355:ILE:HD11	1.68	0.76
3:A:73:LEU:HD22	3:A:260:ALA:HB1	1.68	0.76
3:A:816:THR:HG22	3:A:817:ILE:HG13	1.68	0.75
3:A:872:LEU:O	3:A:875:ILE:HD13	1.87	0.75
3:A:253:ALA:HB1	3:A:257:ARG:NE	1.98	0.75
3:A:253:ALA:CB	3:A:257:ARG:HE	2.00	0.75
3:A:303:LYS:HD3	3:A:303:LYS:N	2.01	0.75
3:A:131:ASN:CG	6:A:980:HOH:O	2.23	0.75
3:A:159:ALA:HA	3:A:162:PHE:CD2	2.20	0.75
1:T:15:DT:H1’	1:T:16:DC:H5”	1.68	0.75
3:A:281:ILE:H	3:A:281:ILE:CD1	2.00	0.74
3:A:363:LYS:HD2	3:A:364:PRO:HD2	1.68	0.74
3:A:141:ILE:HG22	3:A:145:ILE:CD1	2.15	0.74
1:T:10:DG:C6	3:A:237:VAL:HG13	2.23	0.73
3:A:746:ARG:HA	3:A:759:PRO:O	1.88	0.73
3:A:145:ILE:HD13	3:A:212:VAL:CG1	2.18	0.73
3:A:267:MET:HE2	6:A:984:HOH:O	1.66	0.73
3:A:461:LYS:HB3	3:A:482:ILE:HG13	1.70	0.73
3:A:791:LEU:HD23	3:A:792:ARG:N	2.04	0.73
3:A:141:ILE:HG13	3:A:217:ILE:HD11	1.68	0.73
3:A:573:ILE:HG13	3:A:574:VAL:N	2.04	0.73
3:A:739:TYR:HB2	3:A:774:GLN:HE21	1.53	0.73
3:A:333:LYS:N	3:A:333:LYS:HD2	2.04	0.72
3:A:300:HIS:CD2	3:A:300:HIS:H	2.06	0.72
3:A:474:PRO:HA	3:A:880:PHE:HZ	1.53	0.72

Continued on next page...

Continued from previous page...

Atom-1	Atom-2	Interatomic distance (Å)	Clash overlap (Å)
3:A:206:LYS:O	3:A:210:ILE:HG12	1.89	0.72
3:A:363:LYS:HE3	3:A:367:ILE:HD12	1.71	0.72
3:A:393:SER:O	3:A:397:SER:OG	2.06	0.72
3:A:44:TYR:HD2	3:A:148:GLU:HG2	1.53	0.72
3:A:756:ARG:HB3	3:A:758:GLN:HE22	1.52	0.72
3:A:816:THR:HG22	3:A:817:ILE:N	2.04	0.72
3:A:540:CYS:H	3:A:544:GLN:HE21	1.34	0.72
3:A:475:PHE:CE2	3:A:879:ASP:HB2	2.25	0.72
1:T:13:DA:H1'	1:T:14:DG:H5"	1.69	0.72
3:A:798:ALA:O	3:A:804:ILE:HG13	1.88	0.71
3:A:166:VAL:HG12	3:A:167:GLU:H	1.55	0.71
3:A:28:TYR:OH	3:A:185:VAL:HA	1.90	0.71
3:A:747:LEU:HD13	3:A:759:PRO:HG2	1.71	0.71
1:T:20:DT:H2"	1:T:21:DT:H5"	1.71	0.71
3:A:775:GLU:O	3:A:778:ILE:HG22	1.91	0.71
3:A:118:THR:HG22	3:A:220:LEU:HD22	1.72	0.71
3:A:330:ILE:HD12	3:A:330:ILE:N	2.05	0.71
3:A:633:SER:HB2	6:A:910:HOH:O	1.91	0.71
3:A:185:VAL:HG12	3:A:325:ASN:HD21	1.57	0.70
3:A:110:LYS:O	3:A:114:VAL:HG23	1.90	0.70
1:T:14:DG:C2'	1:T:15:DT:H5'	2.15	0.70
3:A:408:PHE:HA	3:A:411:HIS:HD2	1.57	0.69
3:A:452:ILE:HD12	3:A:456:GLY:HA3	1.71	0.69
2:P:102:DA:H2"	2:P:103:DA:OP2	1.90	0.69
3:A:448:LYS:CD	3:A:448:LYS:H	2.06	0.69
3:A:709:GLU:HA	3:A:720:ARG:HE	1.56	0.69
3:A:236:VAL:HG12	3:A:239:GLN:H	1.56	0.69
3:A:703:ALA:O	3:A:706:LEU:HG	1.93	0.69
3:A:118:THR:O	3:A:122:THR:N	2.18	0.69
3:A:557:ARG:HH21	3:A:562:LEU:HD13	1.57	0.69
3:A:448:LYS:H	3:A:448:LYS:HD3	1.58	0.69
3:A:703:ALA:HA	3:A:706:LEU:HG	1.73	0.69
3:A:425:ARG:NH2	6:A:903:HOH:O	2.25	0.69
3:A:445:THR:HG23	3:A:532:LEU:HA	1.75	0.69
3:A:159:ALA:HA	3:A:162:PHE:CE2	2.26	0.69
3:A:740:LYS:HG2	3:A:769:ILE:HG13	1.76	0.68
3:A:417:PRO:O	3:A:429:VAL:HG23	1.94	0.68
3:A:551:ARG:HG2	3:A:870:LEU:O	1.94	0.68
3:A:265:SER:HB3	3:A:269:GLN:NE2	2.09	0.68
3:A:692:ALA:O	3:A:696:MET:HG3	1.94	0.68
2:P:105:DA:H1'	2:P:106:DC:H5"	1.74	0.68

Continued on next page...

Continued from previous page...

Atom-1	Atom-2	Interatomic distance (Å)	Clash overlap (Å)
3:A:269:GLN:HG2	3:A:404:GLN:OE1	1.94	0.67
3:A:29:GLY:HA3	3:A:175:GLY:HA2	1.76	0.67
3:A:328:TRP:CE3	3:A:445:THR:O	2.48	0.67
3:A:439:MET:HA	3:A:509:PHE:CG	2.29	0.67
3:A:659:ILE:HG13	3:A:660:ASP:OD2	1.93	0.67
3:A:578:VAL:CG2	3:A:680:LEU:HB3	2.18	0.67
3:A:244:ILE:HD12	3:A:244:ILE:H	1.60	0.67
3:A:19:ILE:HB	3:A:20:PRO:HD3	1.76	0.67
3:A:388:ASP:O	3:A:392:LYS:HG3	1.95	0.67
3:A:308:TYR:CE2	3:A:734:PRO:HG2	2.29	0.67
3:A:743:ILE:HG13	3:A:763:THR:OG1	1.95	0.67
1:T:5:DC:H4’	3:A:423:ARG:HD2	1.76	0.67
1:T:14:DG:H2”	1:T:15:DT:C5’	2.16	0.66
3:A:828:VAL:HG23	3:A:829:ARG:H	1.60	0.66
3:A:154:ILE:HG22	3:A:290:GLY:HA2	1.77	0.66
3:A:303:LYS:H	3:A:303:LYS:CD	2.09	0.66
3:A:318:LYS:O	3:A:322:ILE:HG13	1.96	0.66
3:A:117:ILE:HG22	3:A:118:THR:N	2.11	0.66
3:A:330:ILE:HD12	3:A:330:ILE:H	1.59	0.66
3:A:612:GLY:O	3:A:616:LEU:HD13	1.95	0.66
3:A:278:TRP:HE1	3:A:324:GLN:HE22	1.44	0.66
3:A:328:TRP:HE3	3:A:445:THR:O	1.79	0.66
3:A:747:LEU:O	3:A:758:GLN:HA	1.95	0.65
3:A:236:VAL:HG11	3:A:239:GLN:HB2	1.79	0.65
3:A:616:LEU:HD23	3:A:676:TYR:HB2	1.78	0.65
3:A:331:ASN:ND2	3:A:334:VAL:H	1.94	0.65
3:A:353:PRO:HD3	3:A:394:ARG:NH1	2.12	0.65
3:A:651:LEU:HD12	3:A:655:ILE:HB	1.78	0.65
3:A:84:ARG:HH12	3:A:88:TRP:HB2	1.61	0.65
1:T:9:DA:O4’	3:A:762:ASN:ND2	2.29	0.65
3:A:578:VAL:HG23	3:A:684:SER:OG	1.98	0.64
2:P:103:DA:H2”	2:P:104:DT:H5”	1.77	0.64
3:A:794:THR:HA	3:A:831:THR:HG21	1.80	0.64
3:A:276:LYS:CE	3:A:287:TRP:HA	2.28	0.64
3:A:313:MET:HB2	3:A:316:VAL:HG23	1.78	0.64
3:A:711:LYS:HD2	3:A:712:ASP:H	1.63	0.64
1:T:21:DT:H2”	1:T:22:DA:C8	2.33	0.64
3:A:804:ILE:HG23	3:A:820:ASP:OD2	1.98	0.63
3:A:39:LEU:O	3:A:42:GLU:HB2	1.97	0.63
3:A:37:LEU:HG	3:A:288:ALA:HB2	1.80	0.63
3:A:693:VAL:HG12	3:A:697:ASN:ND2	2.05	0.63

Continued on next page...

Continued from previous page...

Atom-1	Atom-2	Interatomic distance (Å)	Clash overlap (Å)
3:A:802:TYR:CG	3:A:823:ASN:HB3	2.34	0.63
3:A:479:ILE:HG22	3:A:483:GLU:OE1	1.98	0.63
3:A:475:PHE:N	3:A:476:PRO:HD2	2.12	0.63
3:A:132:THR:O	3:A:244:ILE:HD12	1.99	0.63
3:A:229:LEU:HD21	3:A:242:GLU:OE1	1.99	0.63
3:A:335:LEU:N	3:A:443:LEU:HD21	2.14	0.63
3:A:768:GLU:CD	3:A:769:ILE:H	2.02	0.63
3:A:538:GLY:HA3	3:A:883:ALA:HB3	1.81	0.63
2:P:111:DC:H2”	2:P:112:DA:H5’	1.81	0.62
3:A:120:LYS:O	3:A:124:ALA:N	2.28	0.62
3:A:709:GLU:H	3:A:722:ARG:HE	1.47	0.62
3:A:688:THR:O	3:A:690:VAL:HG22	1.99	0.62
3:A:739:TYR:H	3:A:774:GLN:HE22	1.48	0.62
3:A:73:LEU:O	3:A:77:LEU:HD12	2.00	0.62
3:A:341:ILE:HA	3:A:344:TRP:NE1	2.14	0.62
3:A:579:ASN:O	3:A:582:LEU:HB2	2.00	0.62
3:A:229:LEU:HD11	3:A:242:GLU:HG2	1.81	0.61
3:A:585:ASP:O	3:A:614:LYS:HA	2.00	0.61
3:A:663:LYS:HG2	3:A:664:GLY:N	2.11	0.61
3:A:872:LEU:HA	3:A:875:ILE:HD13	1.80	0.61
3:A:144:ALA:O	3:A:147:ASP:N	2.32	0.61
3:A:617:ALA:O	3:A:621:LEU:HG	2.00	0.61
3:A:782:PHE:O	3:A:786:GLN:HG2	2.00	0.61
3:A:828:VAL:HG23	3:A:829:ARG:HG2	1.82	0.61
3:A:650:VAL:O	3:A:654:THR:HG23	2.01	0.61
3:A:227:VAL:HG12	3:A:246:LEU:CA	2.31	0.61
3:A:324:GLN:HE21	3:A:418:TYR:N	1.98	0.61
3:A:620:TRP:CZ2	3:A:677:MET:HB2	2.35	0.61
3:A:371:PRO:HG2	3:A:372:GLU:H	1.65	0.61
3:A:6:ILE:N	3:A:6:ILE:HD13	2.16	0.61
3:A:703:ALA:O	3:A:706:LEU:N	2.31	0.61
3:A:341:ILE:HA	3:A:344:TRP:CD1	2.35	0.61
3:A:576:LYS:NZ	3:A:577:LYS:HB2	2.16	0.61
3:A:799:HIS:O	3:A:803:GLY:HA2	2.00	0.61
3:A:448:LYS:CD	3:A:448:LYS:N	2.61	0.61
3:A:118:THR:HA	3:A:141:ILE:HD12	1.83	0.60
3:A:339:ASN:N	6:A:931:HOH:O	2.33	0.60
3:A:559:VAL:HG23	3:A:561:LEU:HB2	1.82	0.60
3:A:333:LYS:O	3:A:336:ALA:HB3	1.99	0.60
3:A:747:LEU:HD12	3:A:747:LEU:N	2.16	0.60
3:A:185:VAL:HG22	3:A:186:VAL:N	2.16	0.60

Continued on next page...

Continued from previous page...

Atom-1	Atom-2	Interatomic distance (Å)	Clash overlap (Å)
3:A:450:LYS:HE2	3:A:817:ILE:CD1	2.32	0.60
3:A:628:SER:O	3:A:631:LYS:HB2	2.01	0.60
3:A:120:LYS:HD3	3:A:265:SER:H	1.67	0.60
3:A:644:PHE:HA	3:A:647:ARG:NH2	2.17	0.60
3:A:705:LEU:HD22	6:A:904:HOH:O	2.00	0.60
3:A:446:LEU:HD22	3:A:806:SER:HB3	1.83	0.60
3:A:448:LYS:H	3:A:448:LYS:CE	2.15	0.60
3:A:452:ILE:O	3:A:452:ILE:HD12	2.02	0.60
3:A:169:GLN:NE2	3:A:172:LYS:H	1.99	0.59
3:A:574:VAL:HG13	3:A:684:SER:HB2	1.83	0.59
3:A:265:SER:HB3	3:A:269:GLN:CD	2.22	0.59
3:A:121:THR:CG2	3:A:141:ILE:HD13	2.33	0.59
3:A:154:ILE:H	3:A:154:ILE:HD12	1.67	0.59
3:A:341:ILE:HA	3:A:344:TRP:CE2	2.37	0.59
3:A:410:ASN:O	3:A:411:HIS:C	2.39	0.59
3:A:458:TYR:CE1	3:A:479:ILE:HD11	2.37	0.59
3:A:539:SER:H	3:A:544:GLN:NE2	2.00	0.59
3:A:397:SER:O	3:A:400:PHE:HB2	2.03	0.59
3:A:320:ILE:HD11	3:A:420:MET:HE2	1.84	0.59
3:A:359:GLU:OE1	3:A:360:LEU:HD23	2.03	0.59
3:A:324:GLN:NE2	3:A:418:TYR:H	1.99	0.59
3:A:634:VAL:HG23	6:A:910:HOH:O	2.02	0.59
3:A:855:GLU:HG3	3:A:856:SER:N	2.15	0.59
3:A:367:ILE:HG23	3:A:368:ASP:N	2.16	0.59
3:A:401:MET:HE1	3:A:432:PHE:HA	1.83	0.59
3:A:449:GLY:HA2	3:A:817:ILE:HG21	1.85	0.59
3:A:220:LEU:O	3:A:224:THR:O	2.21	0.58
3:A:463:HIS:CD2	3:A:535:ALA:H	2.20	0.58
3:A:652:GLU:HA	3:A:656:GLN:HB3	1.84	0.58
3:A:363:LYS:HE3	3:A:367:ILE:HA	1.84	0.58
3:A:552:ASP:HB2	3:A:691:ALA:HB2	1.85	0.58
3:A:78:LEU:O	3:A:82:ILE:HG13	2.03	0.58
3:A:195:LEU:O	3:A:196:LEU:HD12	2.03	0.58
3:A:842:LEU:HG	3:A:843:ALA:H	1.66	0.58
3:A:261:LEU:H	3:A:261:LEU:CD1	2.15	0.58
3:A:705:LEU:CD2	6:A:904:HOH:O	2.51	0.58
3:A:562:LEU:HD21	3:A:870:LEU:HD11	1.85	0.58
3:A:44:TYR:CD2	3:A:148:GLU:HG2	2.37	0.58
3:A:353:PRO:HD3	3:A:394:ARG:HH11	1.68	0.58
3:A:234:ALA:HA	3:A:240:ASP:OD2	2.03	0.58
3:A:313:MET:HE3	3:A:316:VAL:HG11	1.86	0.58

Continued on next page...

Continued from previous page...

Atom-1	Atom-2	Interatomic distance (Å)	Clash overlap (Å)
3:A:538:GLY:H	3:A:883:ALA:HB2	1.67	0.58
3:A:185:VAL:HG21	3:A:277:PRO:HD3	1.86	0.58
3:A:359:GLU:CD	3:A:360:LEU:H	2.07	0.58
3:A:450:LYS:HE2	3:A:817:ILE:HD12	1.86	0.58
3:A:492:CYS:SG	3:A:499:ASN:HB3	2.43	0.58
3:A:120:LYS:NZ	3:A:267:MET:SD	2.73	0.58
3:A:639:TYR:HE1	3:A:784:HIS:CE1	2.21	0.58
3:A:116:TYR:HA	3:A:119:ILE:CD1	2.34	0.57
3:A:577:LYS:HA	3:A:580:GLU:OE1	2.04	0.57
3:A:695:ALA:O	3:A:698:TRP:HB3	2.05	0.57
3:A:792:ARG:O	3:A:795:VAL:HG22	2.03	0.57
3:A:845:PHE:O	3:A:848:GLN:HB2	2.04	0.57
1:T:3:DT:H5"	3:A:640:GLY:O	2.04	0.57
3:A:532:LEU:HD12	3:A:533:PRO:HD2	1.86	0.57
3:A:557:ARG:CZ	3:A:562:LEU:HD13	2.34	0.57
3:A:768:GLU:CD	3:A:769:ILE:N	2.57	0.57
3:A:84:ARG:NH1	3:A:88:TRP:HB2	2.18	0.57
3:A:408:PHE:HA	3:A:411:HIS:CD2	2.40	0.57
3:A:475:PHE:HE2	3:A:879:ASP:HB2	1.67	0.57
3:A:545:HIS:NE2	3:A:787:ASP:HA	2.19	0.57
3:A:842:LEU:H	3:A:842:LEU:HD23	1.69	0.57
3:A:872:LEU:C	3:A:875:ILE:HD13	2.25	0.57
2:P:108:DA:H2"	2:P:109:DC:H5'	1.85	0.57
3:A:38:ALA:HB2	3:A:162:PHE:HE1	1.70	0.57
3:A:350:GLU:CD	3:A:350:GLU:N	2.58	0.57
3:A:394:ARG:HH11	3:A:394:ARG:HG2	1.68	0.57
3:A:706:LEU:C	3:A:720:ARG:HH22	2.07	0.57
3:A:79:PRO:HA	3:A:82:ILE:HD12	1.85	0.57
3:A:244:ILE:N	3:A:244:ILE:HD12	2.20	0.57
3:A:297:VAL:HA	3:A:420:MET:O	2.05	0.57
3:A:491:ALA:HA	3:A:494:LYS:HE2	1.85	0.57
3:A:556:GLY:HA2	3:A:559:VAL:HG22	1.85	0.57
3:A:646:PHE:O	3:A:650:VAL:HG12	2.05	0.57
3:A:503:ALA:HA	3:A:508:PRO:HB3	1.86	0.56
3:A:454:LYS:HD3	3:A:455:GLU:N	2.19	0.56
3:A:512:LEU:O	3:A:513:ALA:C	2.43	0.56
3:A:861:MET:H	3:A:862:PRO:CD	2.04	0.56
2:P:111:DC:H2"	2:P:112:DA:C8	2.40	0.56
3:A:124:ALA:HB1	6:A:984:HOH:O	2.05	0.56
3:A:234:ALA:O	3:A:236:VAL:HG23	2.05	0.56
3:A:331:ASN:HD22	3:A:331:ASN:C	2.09	0.56

Continued on next page...

Continued from previous page...

Atom-1	Atom-2	Interatomic distance (Å)	Clash overlap (Å)
3:A:261:LEU:HD21	3:A:396:ILE:HD12	1.86	0.56
3:A:398:LEU:HD21	3:A:402:LEU:HD22	1.87	0.56
3:A:474:PRO:HB2	3:A:476:PRO:HD2	1.87	0.56
3:A:639:TYR:HE1	3:A:784:HIS:HE1	1.53	0.56
3:A:142:GLY:CA	3:A:145:ILE:HD12	2.36	0.56
3:A:229:LEU:HD21	3:A:242:GLU:CD	2.25	0.56
3:A:659:ILE:HA	3:A:663:LYS:O	2.06	0.56
3:A:828:VAL:HG23	3:A:829:ARG:N	2.21	0.56
3:A:335:LEU:O	3:A:339:ASN:OD1	2.23	0.56
3:A:702:ALA:C	3:A:706:LEU:HD23	2.26	0.56
3:A:90:GLU:O	3:A:93:LYS:N	2.39	0.56
3:A:729:THR:HG22	3:A:733:PHE:H	1.70	0.56
3:A:176:HIS:NE2	3:A:178:TYR:HA	2.20	0.56
3:A:19:ILE:HG22	3:A:195:LEU:HD11	1.88	0.56
3:A:74:ILE:CD1	3:A:78:LEU:HB2	2.36	0.56
3:A:560:ASN:OD1	3:A:880:PHE:HB2	2.07	0.55
3:A:141:ILE:CG2	3:A:145:ILE:HD11	2.24	0.55
3:A:353:PRO:HD3	3:A:394:ARG:HG2	1.87	0.55
3:A:300:HIS:H	3:A:300:HIS:HD2	1.52	0.55
3:A:706:LEU:HD12	3:A:706:LEU:C	2.26	0.55
3:A:190:MET:SD	6:A:1010:HOH:O	2.59	0.55
3:A:229:LEU:HD21	3:A:242:GLU:CG	2.37	0.55
3:A:271:CYS:O	3:A:414:ILE:HA	2.07	0.55
3:A:480:LYS:HA	3:A:480:LYS:NZ	2.21	0.55
3:A:849:PHE:HB2	3:A:853:LEU:CB	2.31	0.55
3:A:48:GLU:OE1	6:A:912:HOH:O	2.18	0.55
3:A:756:ARG:CB	3:A:758:GLN:HE22	2.19	0.55
3:A:108:GLU:C	3:A:109:ILE:HD13	2.27	0.55
3:A:576:LYS:HG3	3:A:577:LYS:H	1.72	0.55
3:A:574:VAL:HG13	3:A:684:SER:CB	2.37	0.55
3:A:722:ARG:NH1	3:A:771:ALA:HB2	2.22	0.55
3:A:313:MET:HB2	3:A:316:VAL:CG2	2.37	0.54
3:A:573:ILE:O	3:A:576:LYS:HG3	2.05	0.54
2:P:107:DG:H2”	2:P:108:DA:C5’	2.32	0.54
3:A:73:LEU:HB3	3:A:260:ALA:O	2.07	0.54
1:T:5:DC:H4’	3:A:423:ARG:CD	2.37	0.54
1:T:4:DC:H2”	1:T:5:DC:H5’	1.89	0.54
3:A:711:LYS:HD2	3:A:712:ASP:N	2.21	0.54
3:A:747:LEU:O	3:A:758:GLN:HG3	2.08	0.54
3:A:8:LYS:C	3:A:10:ASP:H	2.11	0.54
3:A:74:ILE:HD11	3:A:78:LEU:HD22	1.89	0.54

Continued on next page...

Continued from previous page...

Atom-1	Atom-2	Interatomic distance (Å)	Clash overlap (Å)
3:A:769:ILE:HD12	3:A:769:ILE:N	2.22	0.54
3:A:474:PRO:HA	3:A:880:PHE:CZ	2.39	0.54
3:A:816:THR:CG2	3:A:817:ILE:H	2.16	0.54
2:P:103:DA:H2”	2:P:104:DT:C5’	2.38	0.54
3:A:213:GLY:O	3:A:216:CYS:HB2	2.08	0.54
3:A:330:ILE:CD1	3:A:330:ILE:H	2.20	0.54
3:A:446:LEU:HD12	3:A:531:SER:O	2.07	0.54
3:A:447:ALA:H	3:A:448:LYS:NZ	2.06	0.54
1:T:18:DT:H2”	1:T:19:DA:C8	2.42	0.54
3:A:488:ASN:HB3	3:A:501:TRP:CZ3	2.43	0.54
3:A:475:PHE:CZ	3:A:879:ASP:HB2	2.42	0.54
3:A:700:LYS:HD3	3:A:700:LYS:O	2.07	0.54
3:A:245:GLU:OE2	3:A:389:LYS:HE2	2.08	0.53
3:A:351:ASP:OD1	3:A:351:ASP:N	2.39	0.53
3:A:129:ALA:HB2	3:A:393:SER:CB	2.38	0.53
3:A:335:LEU:CA	3:A:443:LEU:HD21	2.38	0.53
3:A:562:LEU:CD2	3:A:563:PRO:HD2	2.38	0.53
3:A:308:TYR:HE2	3:A:734:PRO:HG2	1.72	0.53
3:A:330:ILE:CD1	3:A:330:ILE:N	2.71	0.53
3:A:393:SER:O	3:A:396:ILE:HG22	2.08	0.53
3:A:492:CYS:HA	3:A:499:ASN:CB	2.38	0.53
3:A:74:ILE:HD11	3:A:78:LEU:HB2	1.90	0.53
3:A:544:GLN:HG2	3:A:559:VAL:HB	1.90	0.53
3:A:861:MET:N	3:A:862:PRO:CD	2.68	0.53
1:T:2:DT:H2”	3:A:644:PHE:CD2	2.44	0.53
3:A:169:GLN:HE21	3:A:172:LYS:H	1.56	0.53
3:A:541:SER:O	3:A:543:ILE:N	2.41	0.53
3:A:211:HIS:O	3:A:215:ARG:HD3	2.08	0.53
3:A:31:ARG:HB2	3:A:173:ARG:HH21	1.74	0.53
3:A:445:THR:HG22	3:A:446:LEU:H	1.72	0.53
3:A:576:LYS:HG3	3:A:577:LYS:N	2.24	0.53
3:A:174:VAL:HG11	3:A:177:VAL:HG22	1.89	0.53
3:A:374:LEU:HA	3:A:377:TRP:HB2	1.90	0.53
3:A:595:VAL:HG21	3:A:597:VAL:HG23	1.90	0.53
3:A:729:THR:HG22	3:A:733:PHE:N	2.24	0.53
3:A:748:ASN:ND2	3:A:756:ARG:HH21	1.97	0.53
3:A:539:SER:H	3:A:544:GLN:HE22	1.57	0.53
3:A:708:ALA:O	3:A:720:ARG:NH2	2.42	0.53
3:A:108:GLU:O	3:A:109:ILE:HD13	2.08	0.52
1:T:7:DA:OP2	3:A:298:ARG:HD3	2.09	0.52
3:A:632:ARG:NH1	3:A:632:ARG:HB3	2.24	0.52

Continued on next page...

Continued from previous page...

Atom-1	Atom-2	Interatomic distance (Å)	Clash overlap (Å)
3:A:721:LYS:O	3:A:723:CYS:N	2.41	0.52
3:A:706:LEU:HD12	3:A:706:LEU:O	2.10	0.52
3:A:254:ILE:HG23	3:A:261:LEU:HD11	1.91	0.52
3:A:438:ASP:CG	3:A:509:PHE:HB2	2.30	0.52
3:A:816:THR:HG22	3:A:817:ILE:CG1	2.38	0.52
1:T:21:DT:H2”	1:T:22:DA:N7	2.24	0.52
3:A:171:ASN:O	3:A:174:VAL:HG12	2.09	0.52
3:A:458:TYR:HE1	3:A:479:ILE:HD11	1.75	0.52
3:A:688:THR:C	3:A:690:VAL:H	2.12	0.52
2:P:112:DA:C2’	2:P:113:DC:H5”	2.27	0.52
3:A:725:VAL:O	3:A:736:TRP:HA	2.09	0.52
3:A:176:HIS:O	3:A:179:LYS:HB2	2.09	0.52
3:A:278:TRP:H	3:A:321:ASN:HD21	1.58	0.52
3:A:573:ILE:CG1	3:A:574:VAL:N	2.73	0.52
3:A:137:VAL:O	3:A:140:ALA:N	2.42	0.52
3:A:229:LEU:CG	3:A:242:GLU:HG2	2.40	0.52
3:A:259:GLY:O	3:A:262:ALA:HB2	2.09	0.52
3:A:451:PRO:HA	3:A:529:ASN:HA	1.91	0.52
3:A:715:THR:HG22	3:A:717:GLU:OE2	2.09	0.52
3:A:545:HIS:HB3	3:A:836:TYR:OH	2.09	0.52
3:A:229:LEU:HD21	3:A:242:GLU:HG2	1.92	0.52
3:A:789:SER:HA	3:A:792:ARG:NH2	2.24	0.52
3:A:86:ASN:HA	3:A:89:PHE:CD2	2.44	0.52
1:T:10:DG:H2”	1:T:11:DT:OP2	2.10	0.52
3:A:324:GLN:HG3	3:A:417:PRO:HA	1.93	0.51
3:A:446:LEU:HB3	3:A:448:LYS:HZ3	1.74	0.51
3:A:703:ALA:CA	3:A:706:LEU:HG	2.37	0.51
3:A:576:LYS:HZ1	3:A:577:LYS:HB2	1.73	0.51
3:A:846:TYR:HA	3:A:849:PHE:CZ	2.46	0.51
3:A:639:TYR:HA	3:A:780:PRO:HB3	1.91	0.51
3:A:703:ALA:O	3:A:704:LYS:C	2.48	0.51
3:A:107:GLN:C	3:A:109:ILE:H	2.14	0.51
3:A:143:ARG:HD3	6:A:966:HOH:O	2.10	0.51
3:A:151:PHE:O	3:A:155:ARG:HB2	2.10	0.51
3:A:636:THR:HA	3:A:639:TYR:CD2	2.45	0.51
3:A:40:GLU:OE2	3:A:286:TYR:HB3	2.11	0.51
3:A:103:PHE:O	3:A:105:PHE:N	2.43	0.51
3:A:703:ALA:O	3:A:707:ALA:N	2.43	0.51
3:A:711:LYS:HG2	3:A:717:GLU:O	2.11	0.51
3:A:841:VAL:HG23	3:A:842:LEU:N	2.24	0.51
3:A:254:ILE:HG22	3:A:399:GLU:HG3	1.93	0.51

Continued on next page...

Continued from previous page...

Atom-1	Atom-2	Interatomic distance (Å)	Clash overlap (Å)
3:A:308:TYR:CD2	3:A:734:PRO:HG2	2.46	0.51
3:A:402:LEU:CD2	6:A:931:HOH:O	2.57	0.51
3:A:79:PRO:O	3:A:80:LYS:C	2.49	0.51
3:A:826:LYS:O	3:A:830:GLU:HG3	2.11	0.51
1:T:20:DT:C2'	1:T:21:DT:H5"	2.41	0.51
3:A:676:TYR:O	3:A:679:LYS:HB3	2.11	0.51
3:A:740:LYS:HB3	3:A:768:GLU:O	2.10	0.51
3:A:281:ILE:N	3:A:281:ILE:HD13	2.12	0.51
3:A:511:PHE:O	3:A:514:PHE:HB3	2.10	0.51
3:A:632:ARG:HH11	3:A:632:ARG:HB3	1.76	0.51
3:A:712:ASP:O	3:A:714:LYS:N	2.44	0.51
1:T:22:DA:H4'	3:A:94:ALA:O	2.10	0.51
3:A:193:LYS:O	3:A:196:LEU:HD13	2.10	0.51
3:A:320:ILE:HD11	3:A:420:MET:CE	2.41	0.51
3:A:169:GLN:NE2	3:A:171:ASN:N	2.59	0.50
3:A:133:THR:HA	3:A:243:THR:HG22	1.93	0.50
3:A:525:GLY:C	3:A:527:SER:H	2.13	0.50
3:A:743:ILE:HG13	3:A:763:THR:HG1	1.75	0.50
3:A:810:ILE:HB	3:A:813:SER:HB3	1.93	0.50
3:A:121:THR:HG22	3:A:141:ILE:HD13	1.93	0.50
3:A:275:PRO:HD3	3:A:415:TRP:CG	2.46	0.50
3:A:452:ILE:CD1	3:A:456:GLY:CA	2.54	0.50
3:A:872:LEU:CA	3:A:875:ILE:HD13	2.41	0.50
3:A:169:GLN:HE21	3:A:172:LYS:N	2.08	0.50
3:A:281:ILE:HD12	3:A:309:GLU:HA	1.92	0.50
3:A:424:GLY:O	3:A:425:ARG:C	2.49	0.50
3:A:447:ALA:C	3:A:449:GLY:H	2.13	0.50
3:A:595:VAL:CG2	3:A:597:VAL:HG23	2.41	0.50
2:P:109:DC:H2"	2:P:110:DT:H5'	1.92	0.50
3:A:225:GLY:O	3:A:226:MET:C	2.49	0.50
3:A:596:THR:OG1	3:A:608:LYS:HG2	2.11	0.50
3:A:791:LEU:O	3:A:794:THR:HB	2.11	0.50
3:A:150:ARG:O	3:A:151:PHE:HB2	2.11	0.50
3:A:390:ALA:O	3:A:393:SER:HB3	2.11	0.50
3:A:330:ILE:HD13	3:A:408:PHE:O	2.10	0.50
3:A:195:LEU:C	3:A:196:LEU:HD12	2.32	0.50
3:A:227:VAL:HB	3:A:244:ILE:HG22	1.92	0.50
3:A:39:LEU:O	3:A:42:GLU:N	2.44	0.50
3:A:107:GLN:O	3:A:109:ILE:N	2.45	0.50
3:A:77:LEU:HD23	3:A:224:THR:HG21	1.94	0.50
3:A:120:LYS:HD2	3:A:265:SER:O	2.12	0.50

Continued on next page...

Continued from previous page...

Atom-1	Atom-2	Interatomic distance (Å)	Clash overlap (Å)
3:A:338:ALA:O	3:A:342:THR:HG23	2.12	0.50
3:A:443:LEU:O	3:A:444:LEU:HD23	2.12	0.50
3:A:875:ILE:N	3:A:875:ILE:CD1	2.75	0.50
3:A:342:THR:HG21	3:A:439:MET:HE1	1.94	0.50
3:A:561:LEU:HD23	3:A:561:LEU:O	2.11	0.50
3:A:709:GLU:HB3	3:A:722:ARG:HG3	1.93	0.50
3:A:784:HIS:HA	3:A:787:ASP:OD1	2.12	0.50
3:A:234:ALA:C	3:A:236:VAL:H	2.15	0.49
3:A:405:ALA:O	3:A:406:ASN:C	2.51	0.49
3:A:685:VAL:HG23	3:A:686:SER:N	2.27	0.49
3:A:856:SER:C	3:A:858:LEU:HD22	2.33	0.49
3:A:141:ILE:C	3:A:145:ILE:HD12	2.33	0.49
3:A:334:VAL:HB	3:A:443:LEU:HD23	1.93	0.49
3:A:51:PHE:HE1	3:A:75:THR:HA	1.77	0.49
3:A:779:ALA:N	3:A:780:PRO:HD2	2.26	0.49
3:A:792:ARG:O	3:A:793:LYS:C	2.48	0.49
2:P:101:DT:H1'	2:P:102:DA:H5'	1.93	0.49
3:A:710:VAL:HG22	3:A:711:LYS:N	2.27	0.49
3:A:771:ALA:O	3:A:772:HIS:C	2.50	0.49
3:A:17:ALA:HB1	3:A:158:GLU:HA	1.94	0.49
3:A:374:LEU:HD12	3:A:377:TRP:HB3	1.93	0.49
3:A:445:THR:HG22	3:A:446:LEU:N	2.27	0.49
3:A:773:LYS:O	3:A:775:GLU:N	2.46	0.49
3:A:13:ASP:OD2	3:A:13:ASP:N	2.45	0.49
3:A:300:HIS:CG	6:A:913:HOH:O	2.66	0.49
3:A:337:VAL:O	3:A:340:VAL:HB	2.11	0.49
3:A:78:LEU:HD13	3:A:119:ILE:HD11	1.95	0.49
3:A:267:MET:SD	3:A:267:MET:N	2.85	0.49
3:A:341:ILE:HG13	3:A:342:THR:N	2.28	0.49
3:A:778:ILE:HG23	3:A:779:ALA:N	2.28	0.49
3:A:217:ILE:O	3:A:220:LEU:HB3	2.13	0.49
3:A:275:PRO:HD3	3:A:415:TRP:CB	2.43	0.49
3:A:116:TYR:HA	3:A:119:ILE:HD11	1.94	0.49
3:A:174:VAL:HG13	3:A:176:HIS:H	1.76	0.49
1:T:12:DG:OP1	3:A:242:GLU:OE2	2.31	0.49
3:A:398:LEU:HD23	3:A:398:LEU:C	2.33	0.49
3:A:404:GLN:O	3:A:407:LYS:HB3	2.13	0.49
3:A:481:PHE:HA	3:A:484:GLU:OE1	2.12	0.49
3:A:775:GLU:C	3:A:777:GLY:H	2.16	0.49
3:A:142:GLY:HA2	3:A:145:ILE:HD12	1.95	0.49
3:A:237:VAL:HG12	3:A:237:VAL:O	2.11	0.49

Continued on next page...

Continued from previous page...

Atom-1	Atom-2	Interatomic distance (Å)	Clash overlap (Å)
3:A:73:LEU:CD2	3:A:260:ALA:HB1	2.39	0.49
3:A:452:ILE:CD1	3:A:818:PRO:HB3	2.43	0.48
3:A:552:ASP:HB2	3:A:691:ALA:CB	2.42	0.48
3:A:779:ALA:HB3	3:A:780:PRO:CD	2.42	0.48
3:A:81:MET:HB3	3:A:115:ALA:HB1	1.95	0.48
1:T:7:DA:H2"	1:T:8:DT:O4'	2.12	0.48
3:A:124:ALA:O	3:A:125:CYS:C	2.52	0.48
1:T:19:DA:H2'	1:T:20:DT:H71	1.96	0.48
3:A:562:LEU:HD22	3:A:563:PRO:HD2	1.94	0.48
3:A:439:MET:HA	3:A:509:PHE:CD2	2.48	0.48
3:A:771:ALA:O	3:A:774:GLN:HB3	2.14	0.48
3:A:347:CYS:SG	3:A:348:PRO:HD2	2.54	0.48
3:A:421:ASP:CG	3:A:423:ARG:HD3	2.34	0.48
3:A:479:ILE:O	3:A:483:GLU:OE1	2.31	0.48
3:A:620:TRP:CE3	3:A:677:MET:HE3	2.48	0.48
3:A:81:MET:O	3:A:85:ILE:HG12	2.13	0.48
3:A:841:VAL:HG23	3:A:842:LEU:HD23	1.96	0.48
2:P:102:DA:H1'	2:P:103:DA:C8	2.49	0.48
1:T:19:DA:C2'	1:T:20:DT:H5'	2.29	0.48
3:A:229:LEU:CD1	3:A:242:GLU:HG2	2.44	0.48
3:A:257:ARG:O	3:A:260:ALA:HB2	2.14	0.48
3:A:261:LEU:HB3	3:A:264:ILE:HB	1.96	0.48
3:A:373:ALA:O	3:A:377:TRP:HB2	2.13	0.48
3:A:449:GLY:C	3:A:450:LYS:HD3	2.34	0.48
3:A:474:PRO:C	3:A:476:PRO:HD2	2.33	0.48
3:A:711:LYS:HZ3	3:A:714:LYS:HA	1.77	0.48
3:A:192:SER:O	3:A:195:LEU:HD12	2.14	0.48
3:A:360:LEU:HD13	3:A:385:TYR:HE1	1.79	0.48
3:A:398:LEU:HD22	3:A:399:GLU:OE2	2.13	0.48
3:A:460:LEU:HD21	3:A:518:TYR:HB2	1.95	0.48
3:A:579:ASN:HA	3:A:582:LEU:CD1	2.34	0.48
3:A:142:GLY:HA2	3:A:145:ILE:CD1	2.44	0.48
3:A:195:LEU:HA	3:A:289:ASN:HD21	1.79	0.48
3:A:518:TYR:O	3:A:522:GLN:HG3	2.13	0.48
3:A:350:GLU:C	3:A:352:ILE:H	2.16	0.48
3:A:254:ILE:CG2	3:A:399:GLU:HG3	2.44	0.48
1:T:22:DA:O4'	3:A:96:ARG:HB3	2.13	0.48
3:A:401:MET:HE1	3:A:431:MET:O	2.14	0.48
3:A:448:LYS:O	3:A:450:LYS:HD3	2.14	0.48
3:A:496:PRO:O	3:A:498:GLU:N	2.47	0.47
3:A:468:ALA:HA	3:A:505:GLN:CG	2.44	0.47

Continued on next page...

Continued from previous page...

Atom-1	Atom-2	Interatomic distance (Å)	Clash overlap (Å)
3:A:543:ILE:HG22	3:A:559:VAL:HG11	1.96	0.47
3:A:433:ASN:ND2	6:A:905:HOH:O	2.30	0.47
3:A:796:VAL:O	3:A:799:HIS:HB3	2.14	0.47
3:A:90:GLU:O	3:A:92:VAL:N	2.47	0.47
3:A:230:HIS:HA	6:A:963:HOH:O	2.14	0.47
3:A:299:THR:OG1	3:A:305:LEU:HB2	2.14	0.47
3:A:446:LEU:HD11	3:A:533:PRO:HB3	1.94	0.47
3:A:713:LYS:HD2	3:A:713:LYS:O	2.14	0.47
3:A:116:TYR:O	3:A:117:ILE:O	2.32	0.47
3:A:126:LEU:HD11	3:A:227:VAL:HG11	1.95	0.47
3:A:278:TRP:HZ3	3:A:282:THR:HA	1.80	0.47
3:A:486:HIS:HD2	3:A:518:TYR:OH	1.96	0.47
3:A:786:GLN:C	3:A:788:GLY:H	2.16	0.47
3:A:846:TYR:HA	3:A:849:PHE:CE1	2.49	0.47
3:A:20:PRO:HG2	3:A:289:ASN:HB2	1.97	0.47
3:A:459:TRP:HA	3:A:462:ILE:HB	1.96	0.47
3:A:478:ARG:O	3:A:481:PHE:HB3	2.15	0.47
3:A:702:ALA:O	3:A:706:LEU:HD23	2.14	0.47
3:A:775:GLU:C	3:A:777:GLY:N	2.67	0.47
3:A:124:ALA:CB	6:A:984:HOH:O	2.61	0.47
3:A:144:ALA:O	3:A:147:ASP:HB2	2.14	0.47
3:A:402:LEU:HD13	6:A:931:HOH:O	1.82	0.47
3:A:740:LYS:CB	3:A:768:GLU:O	2.62	0.47
1:T:9:DA:C4'	3:A:762:ASN:HD21	2.28	0.47
3:A:835:THR:C	3:A:837:GLU:H	2.18	0.47
3:A:108:GLU:HG3	3:A:108:GLU:H	1.31	0.47
3:A:141:ILE:O	3:A:142:GLY:C	2.53	0.47
3:A:117:ILE:HD12	3:A:144:ALA:HB1	1.97	0.47
3:A:349:VAL:CG1	3:A:352:ILE:HG12	2.44	0.47
3:A:39:LEU:O	3:A:40:GLU:C	2.52	0.47
3:A:465:ALA:HB1	3:A:470:VAL:HB	1.95	0.47
3:A:116:TYR:O	3:A:117:ILE:C	2.51	0.47
3:A:401:MET:CE	3:A:432:PHE:HA	2.44	0.47
3:A:740:LYS:HE2	3:A:767:SER:O	2.14	0.47
1:T:7:DA:H2"	1:T:8:DT:O5'	2.15	0.47
3:A:370:ASN:N	3:A:371:PRO:CD	2.77	0.47
3:A:573:ILE:HD11	3:A:688:THR:HG21	1.96	0.47
3:A:137:VAL:HG21	3:A:244:ILE:CD1	2.34	0.46
3:A:254:ILE:O	3:A:254:ILE:HG22	2.15	0.46
3:A:452:ILE:C	3:A:452:ILE:HD12	2.34	0.46
3:A:454:LYS:HD3	3:A:454:LYS:C	2.36	0.46

Continued on next page...

Continued from previous page...

Atom-1	Atom-2	Interatomic distance (Å)	Clash overlap (Å)
3:A:51:PHE:CE1	3:A:75:THR:HA	2.49	0.46
3:A:545:HIS:O	3:A:549:MET:HG2	2.14	0.46
3:A:134:VAL:HA	3:A:244:ILE:HD11	1.97	0.46
1:T:3:DT:P	1:T:3:DT:H3'	2.55	0.46
3:A:154:ILE:HD12	3:A:154:ILE:N	2.29	0.46
3:A:250:TYR:O	3:A:254:ILE:HG13	2.14	0.46
3:A:338:ALA:C	6:A:931:HOH:O	2.53	0.46
3:A:579:ASN:CA	3:A:582:LEU:HD12	2.35	0.46
3:A:829:ARG:HH21	3:A:882:PHE:HA	1.80	0.46
3:A:90:GLU:OE2	3:A:90:GLU:HA	2.15	0.46
3:A:351:ASP:O	3:A:394:ARG:NH1	2.48	0.46
3:A:746:ARG:C	3:A:747:LEU:HD12	2.36	0.46
3:A:266:PRO:C	3:A:268:PHE:H	2.19	0.46
3:A:316:VAL:O	3:A:319:ALA:HB3	2.15	0.46
3:A:387:LYS:HD3	3:A:387:LYS:O	2.15	0.46
3:A:582:LEU:HB3	3:A:621:LEU:HD21	1.97	0.46
3:A:809:LEU:HD12	3:A:809:LEU:O	2.15	0.46
3:A:174:VAL:HG13	3:A:175:GLY:N	2.31	0.46
3:A:226:MET:HA	3:A:250:TYR:CE2	2.51	0.46
2:P:113:DC:H3'	2:P:114:DT:H4'	1.98	0.46
3:A:524:HIS:HB2	3:A:528:TYR:HB2	1.98	0.46
3:A:679:LYS:O	3:A:683:GLU:HG3	2.15	0.46
3:A:711:LYS:HD2	3:A:712:ASP:O	2.15	0.46
3:A:850:ALA:HA	3:A:854:HIS:NE2	2.30	0.46
3:A:148:GLU:CD	3:A:155:ARG:HD3	2.36	0.46
3:A:224:THR:HG22	3:A:250:TYR:OH	2.16	0.46
3:A:631:LYS:O	3:A:634:VAL:HG23	2.15	0.46
3:A:773:LYS:C	3:A:775:GLU:N	2.68	0.46
3:A:775:GLU:O	3:A:777:GLY:N	2.49	0.46
3:A:345:LYS:CD	3:A:355:ILE:HD11	2.40	0.46
3:A:496:PRO:C	3:A:498:GLU:N	2.70	0.46
3:A:677:MET:HG3	3:A:681:ILE:CD1	2.43	0.46
3:A:830:GLU:O	3:A:834:ASP:HB3	2.16	0.46
3:A:726:HIS:CD2	3:A:852:GLN:HE22	2.34	0.46
2:P:105:DA:OP1	3:A:95:LYS:HE2	2.16	0.46
2:P:105:DA:N3	3:A:98:LYS:HE2	2.31	0.46
3:A:371:PRO:HG2	3:A:373:ALA:H	1.81	0.45
3:A:651:LEU:HA	3:A:655:ILE:HB	1.97	0.45
3:A:703:ALA:HA	3:A:706:LEU:CG	2.45	0.45
3:A:425:ARG:CZ	6:A:903:HOH:O	2.63	0.45
3:A:729:THR:HG21	3:A:733:PHE:HB3	1.99	0.45

Continued on next page...

Continued from previous page...

Atom-1	Atom-2	Interatomic distance (Å)	Clash overlap (Å)
3:A:849:PHE:O	3:A:850:ALA:C	2.53	0.45
3:A:176:HIS:CD2	3:A:178:TYR:HA	2.51	0.45
3:A:107:GLN:HG3	3:A:108:GLU:H	1.81	0.45
3:A:135:GLN:N	3:A:135:GLN:OE1	2.46	0.45
3:A:269:GLN:HE21	3:A:404:GLN:CD	2.18	0.45
3:A:687:VAL:HA	3:A:690:VAL:HG13	1.99	0.45
3:A:771:ALA:O	3:A:774:GLN:N	2.49	0.45
3:A:359:GLU:O	3:A:384:VAL:HG11	2.17	0.45
3:A:210:ILE:HG13	3:A:761:ILE:HD13	1.98	0.45
3:A:861:MET:O	3:A:862:PRO:O	2.35	0.45
3:A:458:TYR:HE1	3:A:479:ILE:CD1	2.29	0.45
3:A:489:ILE:HA	3:A:492:CYS:HB2	1.98	0.45
3:A:496:PRO:C	3:A:498:GLU:H	2.19	0.45
3:A:488:ASN:HB3	3:A:501:TRP:CE3	2.52	0.45
3:A:502:TRP:CE3	3:A:512:LEU:HB2	2.51	0.45
3:A:711:LYS:NZ	3:A:714:LYS:HA	2.32	0.45
3:A:727:TRP:CD1	3:A:728:VAL:N	2.85	0.45
3:A:875:ILE:N	3:A:875:ILE:HD12	2.31	0.45
3:A:141:ILE:HG13	3:A:217:ILE:CD1	2.44	0.45
3:A:20:PRO:HA	3:A:195:LEU:HD21	1.99	0.45
3:A:393:SER:O	3:A:396:ILE:CG2	2.65	0.45
3:A:545:HIS:CE1	3:A:787:ASP:HB3	2.52	0.45
3:A:492:CYS:HA	3:A:499:ASN:HB2	1.97	0.45
3:A:734:PRO:HB2	3:A:736:TRP:CH2	2.52	0.45
3:A:159:ALA:C	3:A:161:HIS:N	2.70	0.45
3:A:557:ARG:CB	3:A:562:LEU:HD12	2.38	0.45
3:A:712:ASP:CG	3:A:713:LYS:N	2.69	0.45
2:P:103:DA:C2’	2:P:104:DT:H5”	2.46	0.45
3:A:25:ALA:HA	3:A:29:GLY:O	2.17	0.45
3:A:611:LEU:CD2	3:A:612:GLY:H	2.30	0.45
3:A:77:LEU:H	3:A:77:LEU:CD1	2.21	0.45
3:A:786:GLN:C	3:A:788:GLY:N	2.71	0.45
3:A:31:ARG:HG2	3:A:31:ARG:HH11	1.83	0.44
3:A:756:ARG:HB3	3:A:758:GLN:NE2	2.25	0.44
3:A:537:ASP:O	3:A:882:PHE:HD2	2.01	0.44
3:A:192:SER:HB3	3:A:195:LEU:CD1	2.47	0.44
3:A:196:LEU:HB3	3:A:197:GLY:H	1.57	0.44
3:A:118:THR:CG2	3:A:220:LEU:HD22	2.43	0.44
3:A:28:TYR:CD1	3:A:183:MET:HG3	2.52	0.44
3:A:126:LEU:C	3:A:128:SER:H	2.20	0.44
3:A:300:HIS:HB3	6:A:913:HOH:O	2.17	0.44

Continued on next page...

Continued from previous page...

Atom-1	Atom-2	Interatomic distance (Å)	Clash overlap (Å)
3:A:300:HIS:O	3:A:301:SER:HB2	2.17	0.44
3:A:576:LYS:HA	3:A:579:ASN:HB2	1.99	0.44
3:A:631:LYS:HE2	3:A:635:MET:SD	2.58	0.44
3:A:772:HIS:O	3:A:775:GLU:HB3	2.17	0.44
3:A:779:ALA:HB3	3:A:780:PRO:HD3	2.00	0.44
3:A:639:TYR:CE1	3:A:784:HIS:HE1	2.34	0.44
2:P:103:DA:H5'	3:A:96:ARG:NH2	2.32	0.44
3:A:574:VAL:O	3:A:578:VAL:HB	2.18	0.44
3:A:227:VAL:HA	3:A:246:LEU:HA	2.00	0.44
3:A:278:TRP:CE2	3:A:284:GLY:HA3	2.52	0.44
3:A:532:LEU:HD12	3:A:533:PRO:CD	2.47	0.44
3:A:739:TYR:H	3:A:774:GLN:NE2	2.13	0.44
3:A:391:ARG:O	3:A:392:LYS:C	2.55	0.44
3:A:556:GLY:HA2	3:A:559:VAL:CG2	2.48	0.44
3:A:709:GLU:N	3:A:722:ARG:HG2	2.33	0.44
3:A:719:LEU:N	3:A:719:LEU:HD12	2.31	0.44
3:A:748:ASN:O	3:A:749:LEU:HD23	2.18	0.44
3:A:105:PHE:HA	3:A:108:GLU:OE1	2.18	0.44
3:A:119:ILE:O	3:A:123:LEU:HG	2.17	0.44
3:A:224:THR:HG22	3:A:226:MET:H	1.81	0.44
3:A:363:LYS:HZ3	3:A:364:PRO:HG2	1.82	0.44
3:A:394:ARG:HG2	3:A:394:ARG:NH1	2.31	0.44
3:A:423:ARG:HH22	3:A:784:HIS:CD2	2.35	0.44
3:A:512:LEU:O	3:A:515:CYS:N	2.50	0.44
3:A:562:LEU:C	3:A:564:SER:H	2.21	0.44
3:A:472:LYS:C	3:A:567:VAL:HG21	2.38	0.44
1:T:16:DC:H2"	1:T:17:DG:C8	2.52	0.44
3:A:313:MET:SD	3:A:734:PRO:HD2	2.58	0.44
3:A:842:LEU:HG	3:A:843:ALA:N	2.32	0.44
3:A:245:GLU:HG3	3:A:389:LYS:HD3	2.00	0.44
3:A:475:PHE:N	3:A:476:PRO:CD	2.81	0.44
3:A:605:ILE:HD11	3:A:608:LYS:HD2	1.99	0.44
3:A:704:LYS:C	3:A:706:LEU:H	2.21	0.44
2:P:105:DA:H1'	2:P:106:DC:C5'	2.44	0.44
3:A:186:VAL:HB	3:A:187:GLU:H	1.70	0.43
3:A:461:LYS:HG2	3:A:482:ILE:HG21	1.99	0.43
3:A:428:ALA:H	3:A:435:GLN:HE22	1.67	0.43
3:A:795:VAL:HG23	3:A:796:VAL:N	2.33	0.43
3:A:826:LYS:HB3	3:A:826:LYS:HZ2	1.84	0.43
1:T:17:DG:N2	2:P:107:DG:C2	2.86	0.43
3:A:501:TRP:HA	3:A:504:GLU:OE2	2.17	0.43

Continued on next page...

Continued from previous page...

Atom-1	Atom-2	Interatomic distance (Å)	Clash overlap (Å)
3:A:505:GLN:O	3:A:508:PRO:HD3	2.18	0.43
3:A:809:LEU:HD12	3:A:809:LEU:N	2.33	0.43
3:A:167:GLU:O	3:A:167:GLU:HG3	2.18	0.43
3:A:17:ALA:HB1	3:A:158:GLU:HG3	2.00	0.43
3:A:297:VAL:O	3:A:299:THR:HG23	2.17	0.43
3:A:402:LEU:HD21	6:A:931:HOH:O	2.18	0.43
3:A:50:ARG:O	3:A:53:LYS:HB3	2.17	0.43
3:A:138:ALA:O	3:A:139:SER:C	2.54	0.43
3:A:211:HIS:O	3:A:215:ARG:HB2	2.18	0.43
3:A:377:TRP:C	3:A:379:ARG:N	2.72	0.43
3:A:785:SER:O	3:A:789:SER:HB2	2.18	0.43
3:A:835:THR:O	3:A:839:CYS:HB2	2.18	0.43
3:A:501:TRP:HB2	6:A:935:HOH:O	2.18	0.43
3:A:73:LEU:O	3:A:77:LEU:CD1	2.66	0.43
3:A:798:ALA:HB1	3:A:804:ILE:CD1	2.26	0.43
3:A:828:VAL:O	3:A:831:THR:OG1	2.35	0.43
3:A:549:MET:SD	3:A:841:VAL:HG21	2.59	0.43
3:A:142:GLY:N	3:A:145:ILE:HD12	2.34	0.43
3:A:363:LYS:CE	3:A:367:ILE:HA	2.49	0.43
3:A:374:LEU:HA	3:A:377:TRP:CB	2.49	0.43
3:A:491:ALA:HB3	3:A:499:ASN:OD1	2.18	0.43
3:A:512:LEU:HA	3:A:512:LEU:HD12	1.76	0.43
3:A:731:ASP:OD2	3:A:789:SER:OG	2.32	0.43
3:A:187:GLU:N	3:A:187:GLU:CD	2.72	0.43
3:A:723:CYS:HB3	3:A:853:LEU:CD1	2.49	0.43
3:A:643:GLU:CD	3:A:679:LYS:HA	2.39	0.43
3:A:136:ALA:O	3:A:137:VAL:C	2.57	0.43
3:A:161:HIS:C	3:A:163:LYS:H	2.20	0.43
3:A:36:GLN:HE21	3:A:40:GLU:HG3	1.84	0.43
3:A:631:LYS:HB3	3:A:632:ARG:H	1.73	0.43
3:A:465:ALA:O	3:A:466:ASN:C	2.57	0.42
3:A:610:LYS:HA	3:A:610:LYS:HD2	1.79	0.42
3:A:855:GLU:O	3:A:858:LEU:HD21	2.19	0.42
3:A:201:TRP:HE3	3:A:202:SER:H	1.68	0.42
3:A:278:TRP:O	3:A:279:THR:HG23	2.19	0.42
3:A:355:ILE:HG22	3:A:395:ARG:HH21	1.85	0.42
3:A:465:ALA:HA	3:A:468:ALA:HB3	2.00	0.42
3:A:473:VAL:CG2	3:A:474:PRO:HD2	2.29	0.42
3:A:559:VAL:C	3:A:561:LEU:H	2.22	0.42
3:A:729:THR:CG2	3:A:733:PHE:HB3	2.49	0.42
3:A:425:ARG:HD3	3:A:811:HIS:CD2	2.54	0.42

Continued on next page...

Continued from previous page...

Atom-1	Atom-2	Interatomic distance (Å)	Clash overlap (Å)
3:A:824:LEU:O	3:A:826:LYS:N	2.52	0.42
3:A:849:PHE:HA	3:A:852:GLN:HB3	2.00	0.42
3:A:169:GLN:HE21	3:A:171:ASN:N	2.17	0.42
3:A:28:TYR:HD1	3:A:183:MET:HB2	1.84	0.42
3:A:286:TYR:HD2	3:A:294:LEU:HD21	1.84	0.42
3:A:295:ALA:O	3:A:419:ASN:OD1	2.37	0.42
3:A:383:ALA:O	3:A:387:LYS:HB2	2.19	0.42
3:A:403:GLU:O	3:A:407:LYS:N	2.51	0.42
3:A:465:ALA:O	3:A:468:ALA:N	2.53	0.42
3:A:545:HIS:O	3:A:548:ALA:HB3	2.19	0.42
3:A:718:ILE:HD12	3:A:718:ILE:C	2.39	0.42
2:P:110:DT:H2'	2:P:110:DT:H6	1.74	0.42
3:A:323:ALA:O	3:A:326:THR:OG1	2.32	0.42
3:A:335:LEU:HA	3:A:443:LEU:HD21	2.01	0.42
3:A:471:ASP:HA	3:A:478:ARG:HH21	1.85	0.42
3:A:774:GLN:HE21	3:A:774:GLN:HA	1.84	0.42
3:A:855:GLU:CG	3:A:856:SER:H	2.14	0.42
3:A:616:LEU:HD23	3:A:676:TYR:CG	2.55	0.42
3:A:133:THR:HB	3:A:135:GLN:OE1	2.20	0.42
3:A:349:VAL:HG12	3:A:352:ILE:HG12	2.02	0.42
3:A:438:ASP:OD2	3:A:509:PHE:HB2	2.19	0.42
3:A:693:VAL:HA	3:A:696:MET:HE2	2.01	0.42
3:A:103:PHE:O	3:A:104:GLN:C	2.58	0.42
3:A:298:ARG:HE	3:A:419:ASN:HD22	1.67	0.42
3:A:485:ASN:O	3:A:488:ASN:HB2	2.19	0.42
3:A:549:MET:O	3:A:550:LEU:HD23	2.20	0.42
3:A:646:PHE:HB2	3:A:678:ALA:CB	2.50	0.42
3:A:147:ASP:OD1	3:A:292:ARG:HD3	2.19	0.42
3:A:344:TRP:N	3:A:344:TRP:CD1	2.88	0.42
3:A:460:LEU:HD23	3:A:461:LYS:N	2.34	0.42
3:A:857:GLN:O	3:A:858:LEU:HB2	2.20	0.42
1:T:17:DG:H2"	1:T:18:DT:H5"	2.02	0.42
3:A:159:ALA:HA	3:A:162:PHE:HD2	1.80	0.42
3:A:174:VAL:HG13	3:A:177:VAL:H	1.82	0.42
3:A:154:ILE:O	3:A:158:GLU:HB2	2.20	0.41
3:A:177:VAL:HB	3:A:179:LYS:CD	2.43	0.41
3:A:269:GLN:HE22	3:A:407:LYS:HE3	1.85	0.41
3:A:595:VAL:HG23	3:A:596:THR:N	2.35	0.41
3:A:740:LYS:HB3	3:A:766:ASP:OD2	2.20	0.41
3:A:261:LEU:HD21	3:A:396:ILE:CD1	2.49	0.41
3:A:312:TYR:C	3:A:314:PRO:HD3	2.40	0.41

Continued on next page...

Continued from previous page...

Atom-1	Atom-2	Interatomic distance (Å)	Clash overlap (Å)
3:A:365:GLU:HB2	3:A:366:ASP:H	1.67	0.41
3:A:360:LEU:HB3	3:A:384:VAL:HG21	2.02	0.41
3:A:693:VAL:O	3:A:697:ASN:ND2	2.53	0.41
1:T:8:DT:OP1	1:T:8:DT:H4'	2.20	0.41
3:A:133:THR:C	3:A:135:GLN:N	2.74	0.41
3:A:371:PRO:HG2	3:A:372:GLU:N	2.35	0.41
3:A:487:GLU:HA	3:A:490:MET:HG2	2.03	0.41
3:A:42:GLU:HG2	3:A:50:ARG:NH1	2.35	0.41
3:A:452:ILE:HG22	3:A:528:TYR:O	2.20	0.41
3:A:620:TRP:CH2	3:A:677:MET:HB2	2.56	0.41
3:A:84:ARG:HD2	3:A:84:ARG:HA	1.91	0.41
3:A:560:ASN:CB	3:A:881:ALA:HB2	2.41	0.41
3:A:275:PRO:O	3:A:277:PRO:HD3	2.20	0.41
3:A:308:TYR:CE2	3:A:736:TRP:HZ3	2.38	0.41
3:A:453:GLY:HA2	3:A:526:LEU:O	2.20	0.41
3:A:542:GLY:HA2	3:A:783:VAL:HG13	2.03	0.41
3:A:120:LYS:HA	3:A:123:LEU:HD12	2.03	0.41
3:A:159:ALA:O	3:A:163:LYS:HG2	2.21	0.41
3:A:324:GLN:C	3:A:326:THR:H	2.24	0.41
3:A:452:ILE:HG23	3:A:453:GLY:N	2.35	0.41
3:A:505:GLN:OE1	3:A:505:GLN:N	2.54	0.41
3:A:570:ILE:HG23	3:A:571:TYR:N	2.35	0.41
3:A:725:VAL:CB	3:A:737:GLN:HB2	2.38	0.41
3:A:826:LYS:CB	3:A:826:LYS:HZ2	2.33	0.41
3:A:879:ASP:N	3:A:879:ASP:OD1	2.54	0.41
3:A:88:TRP:O	3:A:92:VAL:HG23	2.21	0.41
3:A:105:PHE:CD1	3:A:105:PHE:N	2.89	0.41
3:A:297:VAL:HG11	3:A:308:TYR:CE1	2.56	0.41
3:A:396:ILE:O	3:A:396:ILE:HG13	2.20	0.41
3:A:537:ASP:OD1	3:A:813:SER:HB2	2.21	0.41
3:A:651:LEU:O	3:A:656:GLN:N	2.51	0.41
2:P:106:DC:H2"	2:P:107:DG:C8	2.54	0.41
3:A:111:PRO:C	3:A:113:ALA:N	2.74	0.41
3:A:234:ALA:C	3:A:236:VAL:N	2.74	0.41
3:A:773:LYS:O	3:A:774:GLN:C	2.59	0.41
1:T:17:DG:C2'	1:T:18:DT:H5"	2.51	0.41
3:A:30:GLU:HG2	3:A:166:VAL:HG21	2.03	0.41
3:A:215:ARG:NH1	3:A:218:GLU:OE1	2.54	0.41
3:A:546:PHE:CZ	3:A:696:MET:HG2	2.56	0.41
3:A:570:ILE:O	3:A:573:ILE:HG12	2.21	0.41
3:A:797:TRP:HZ2	3:A:801:LYS:HZ2	1.67	0.41

Continued on next page...

Continued from previous page...

Atom-1	Atom-2	Interatomic distance (Å)	Clash overlap (Å)
3:A:100:PRO:O	3:A:103:PHE:HB3	2.21	0.41
3:A:121:THR:HA	3:A:267:MET:HE3	2.03	0.41
3:A:339:ASN:N	3:A:339:ASN:OD1	2.54	0.41
3:A:364:PRO:O	3:A:366:ASP:N	2.54	0.41
3:A:468:ALA:HA	3:A:505:GLN:HB3	2.02	0.41
3:A:646:PHE:HB2	3:A:678:ALA:HB1	2.03	0.41
3:A:784:HIS:O	3:A:788:GLY:N	2.54	0.41
3:A:329:LYS:HG2	3:A:330:ILE:N	2.36	0.40
3:A:359:GLU:OE1	3:A:360:LEU:N	2.54	0.40
3:A:238:GLY:C	3:A:239:GLN:HE21	2.25	0.40
3:A:480:LYS:O	3:A:484:GLU:HG3	2.20	0.40
3:A:599:ASP:HB3	3:A:600:GLU:H	1.72	0.40
3:A:639:TYR:HA	3:A:780:PRO:CB	2.52	0.40
3:A:652:GLU:O	3:A:657:PRO:HD3	2.20	0.40
3:A:6:ILE:N	3:A:6:ILE:CD1	2.82	0.40
3:A:739:TYR:HB2	3:A:774:GLN:HE22	1.77	0.40
3:A:331:ASN:ND2	3:A:331:ASN:C	2.75	0.40
3:A:475:PHE:C	3:A:479:ILE:HD12	2.41	0.40
3:A:655:ILE:HD12	3:A:667:PHE:CE1	2.56	0.40
3:A:542:GLY:CA	3:A:783:VAL:HG13	2.52	0.40
3:A:102:ALA:C	3:A:106:LEU:HD12	2.42	0.40
3:A:195:LEU:N	3:A:195:LEU:HD12	2.36	0.40
3:A:350:GLU:C	3:A:352:ILE:N	2.75	0.40
3:A:41:HIS:C	3:A:43:SER:N	2.74	0.40
3:A:308:TYR:OH	3:A:735:VAL:HA	2.22	0.40
3:A:78:LEU:HB3	3:A:79:PRO:CD	2.51	0.40
3:A:809:LEU:HA	3:A:813:SER:O	2.21	0.40
3:A:882:PHE:O	3:A:883:ALA:C	2.59	0.40
3:A:8:LYS:C	3:A:10:ASP:N	2.75	0.40
3:A:234:ALA:O	3:A:236:VAL:N	2.55	0.40
3:A:267:MET:HE1	6:A:984:HOH:O	1.80	0.40
3:A:335:LEU:HD22	3:A:406:ASN:OD1	2.21	0.40
3:A:582:LEU:O	3:A:617:ALA:HB1	2.22	0.40
3:A:816:THR:CG2	3:A:817:ILE:N	2.75	0.40

All (1) symmetry-related close contacts are listed below. The label for Atom-2 includes the symmetry operator and encoded unit-cell translations to be applied.

Atom-1	Atom-2	Interatomic distance (Å)	Clash overlap (Å)
3:A:498:GLU:OE1	3:A:619:GLN:NE2[3_646]	2.08	0.12

5.3 Torsion angles (i)

5.3.1 Protein backbone (i)

In the following table, the Percentiles column shows the percent Ramachandran outliers of the chain as a percentile score with respect to all X-ray entries followed by that with respect to entries of similar resolution.

The Analysed column shows the number of residues for which the backbone conformation was analysed, and the total number of residues.

Mol	Chain	Analysed	Favoured	Allowed	Outliers	Percentiles
3	A	858/878 (98%)	558 (65%)	218 (25%)	82 (10%)	0 0

All (82) Ramachandran outliers are listed below:

Mol	Chain	Res	Type
3	A	362	MET
3	A	371	PRO
3	A	595	VAL
3	A	598	THR
3	A	609	VAL
3	A	704	LYS
3	A	722	ARG
3	A	861	MET
3	A	862	PRO
3	A	104	GLN
3	A	108	GLU
3	A	119	ILE
3	A	137	VAL
3	A	176	HIS
3	A	189	ASP
3	A	354	ALA
3	A	359	GLU
3	A	365	GLU
3	A	366	ASP
3	A	541	SER
3	A	592	ASN
3	A	689	VAL
3	A	690	VAL
3	A	713	LYS
3	A	7	ALA
3	A	91	GLU
3	A	117	ILE
3	A	118	THR

Continued on next page...

Continued from previous page...

Mol	Chain	Res	Type
3	A	165	ASN
3	A	182	PHE
3	A	262	ALA
3	A	279	THR
3	A	288	ALA
3	A	338	ALA
3	A	409	ALA
3	A	411	HIS
3	A	448	LYS
3	A	538	GLY
3	A	560	ASN
3	A	667	PHE
3	A	738	GLU
3	A	764	ASN
3	A	774	GLN
3	A	825	PHE
3	A	830	GLU
3	A	841	VAL
3	A	36	GLN
3	A	142	GLY
3	A	169	GLN
3	A	179	LYS
3	A	199	GLU
3	A	226	MET
3	A	270	PRO
3	A	309	GLU
3	A	625	VAL
3	A	681	ILE
3	A	703	ALA
3	A	705	LEU
3	A	772	HIS
3	A	834	ASP
3	A	73	LEU
3	A	185	VAL
3	A	257	ARG
3	A	266	PRO
3	A	298	ARG
3	A	451	PRO
3	A	628	SER
3	A	771	ALA
3	A	833	VAL
3	A	849	PHE

Continued on next page...

Continued from previous page...

Mol	Chain	Res	Type
3	A	857	GLN
3	A	859	ASP
3	A	141	ILE
3	A	233	ASN
3	A	564	SER
3	A	607	GLU
3	A	235	GLY
3	A	237	VAL
3	A	85	ILE
3	A	589	GLY
3	A	597	VAL
3	A	542	GLY

5.3.2 Protein sidechains ⓘ

In the following table, the Percentiles column shows the percent sidechain outliers of the chain as a percentile score with respect to all X-ray entries followed by that with respect to entries of similar resolution.

The Analysed column shows the number of residues for which the sidechain conformation was analysed, and the total number of residues.

Mol	Chain	Analysed	Rotameric	Outliers	Percentiles
3	A	714/724 (99%)	617 (86%)	97 (14%)	3 7

All (97) residues with a non-rotameric sidechain are listed below:

Mol	Chain	Res	Type
3	A	6	ILE
3	A	11	PHE
3	A	13	ASP
3	A	26	ASP
3	A	46	MET
3	A	48	GLU
3	A	52	ARG
3	A	74	ILE
3	A	81	MET
3	A	87	ASP
3	A	99	ARG
3	A	101	THR
3	A	105	PHE
3	A	107	GLN

Continued on next page...

Continued from previous page...

Mol	Chain	Res	Type
3	A	108	GLU
3	A	119	ILE
3	A	127	THR
3	A	139	SER
3	A	146	GLU
3	A	148	GLU
3	A	162	PHE
3	A	168	GLU
3	A	173	ARG
3	A	179	LYS
3	A	183	MET
3	A	186	VAL
3	A	187	GLU
3	A	199	GLU
3	A	201	TRP
3	A	206	LYS
3	A	242	GLU
3	A	244	ILE
3	A	273	VAL
3	A	279	THR
3	A	281	ILE
3	A	296	LEU
3	A	300	HIS
3	A	307	ARG
3	A	331	ASN
3	A	333	LYS
3	A	335	LEU
3	A	351	ASP
3	A	359	GLU
3	A	366	ASP
3	A	397	SER
3	A	437	ASN
3	A	450	LYS
3	A	452	ILE
3	A	460	LEU
3	A	471	ASP
3	A	472	LYS
3	A	473	VAL
3	A	492	CYS
3	A	498	GLU
3	A	500	THR
3	A	510	CYS

Continued on next page...

Continued from previous page...

Mol	Chain	Res	Type
3	A	514	PHE
3	A	529	ASN
3	A	537	ASP
3	A	560	ASN
3	A	576	LYS
3	A	578	VAL
3	A	579	ASN
3	A	588	ASN
3	A	599	ASP
3	A	600	GLU
3	A	605	ILE
3	A	611	LEU
3	A	631	LYS
3	A	632	ARG
3	A	635	MET
3	A	654	THR
3	A	690	VAL
3	A	698	TRP
3	A	704	LYS
3	A	706	LEU
3	A	712	ASP
3	A	713	LYS
3	A	717	GLU
3	A	720	ARG
3	A	729	THR
3	A	735	VAL
3	A	737	GLN
3	A	739	TYR
3	A	752	LEU
3	A	774	GLN
3	A	791	LEU
3	A	793	LYS
3	A	809	LEU
3	A	814	PHE
3	A	817	ILE
3	A	840	ASP
3	A	842	LEU
3	A	854	HIS
3	A	858	LEU
3	A	875	ILE
3	A	877	GLU

Some sidechains can be flipped to improve hydrogen bonding and reduce clashes. All (28) such

sidechains are listed below:

Mol	Chain	Res	Type
3	A	22	ASN
3	A	36	GLN
3	A	107	GLN
3	A	169	GLN
3	A	239	GLN
3	A	269	GLN
3	A	289	ASN
3	A	321	ASN
3	A	324	GLN
3	A	325	ASN
3	A	331	ASN
3	A	435	GLN
3	A	485	ASN
3	A	486	HIS
3	A	544	GLN
3	A	560	ASN
3	A	648	GLN
3	A	697	ASN
3	A	737	GLN
3	A	748	ASN
3	A	754	GLN
3	A	758	GLN
3	A	762	ASN
3	A	774	GLN
3	A	781	ASN
3	A	784	HIS
3	A	852	GLN
3	A	857	GLN

5.3.3 RNA (i)

There are no RNA molecules in this entry.

5.4 Non-standard residues in protein, DNA, RNA chains (i)

There are no non-standard protein/DNA/RNA residues in this entry.

5.5 Carbohydrates (i)

There are no carbohydrates in this entry.

5.6 Ligand geometry (i)

Of 4 ligands modelled in this entry, 2 are monoatomic - leaving 2 for Mogul analysis.

In the following table, the Counts columns list the number of bonds (or angles) for which Mogul statistics could be retrieved, the number of bonds (or angles) that are observed in the model and the number of bonds (or angles) that are defined in the Chemical Component Dictionary. The Link column lists molecule types, if any, to which the group is linked. The Z score for a bond length (or angle) is the number of standard deviations the observed value is removed from the expected value. A bond length (or angle) with $|Z| > 2$ is considered an outlier worth inspection. RMSZ is the root-mean-square of all Z scores of the bond lengths (or angles).

Mol	Type	Chain	Res	Link	Bond lengths			Bond angles		
					Counts	RMSZ	$\#\|Z\| > 2$	Counts	RMSZ	$\#\|Z\| > 2$
5	GH3	A	902	-	25,33,33	1.69	5 (20%)	30,52,52	2.43	8 (26%)
5	GH3	A	901	4	25,33,33	1.59	4 (16%)	30,52,52	2.49	8 (26%)

In the following table, the Chirals column lists the number of chiral outliers, the number of chiral centers analysed, the number of these observed in the model and the number defined in the Chemical Component Dictionary. Similar counts are reported in the Torsion and Rings columns. '-' means no outliers of that kind were identified.

Mol	Type	Chain	Res	Link	Chirals	Torsions	Rings
5	GH3	A	902	-	-	5/18/34/34	0/3/3/3
5	GH3	A	901	4	-	3/18/34/34	0/3/3/3

All (9) bond length outliers are listed below:

Mol	Chain	Res	Type	Atoms	Z	Observed(Å)	Ideal(Å)
5	A	902	GH3	C6-N1	4.45	1.40	1.33
5	A	901	GH3	C6-N1	4.06	1.40	1.33
5	A	902	GH3	C2-N1	3.51	1.41	1.35
5	A	902	GH3	O2'-C2'	2.60	1.48	1.43
5	A	901	GH3	C2-N1	2.57	1.40	1.35
5	A	902	GH3	C8-N7	-2.31	1.30	1.34
5	A	901	GH3	O2'-C2'	2.21	1.48	1.43
5	A	902	GH3	C2'-C1'	2.20	1.56	1.54
5	A	901	GH3	O4'-C1'	2.15	1.44	1.41

All (16) bond angle outliers are listed below:

Mol	Chain	Res	Type	Atoms	Z	Observed(^{o})	Ideal(^{o})
5	A	902	GH3	C5-C6-N1	-7.25	113.51	123.43
5	A	901	GH3	C5-C6-N1	-7.14	113.67	123.43

Continued on next page...

Continued from previous page...

Mol	Chain	Res	Type	Atoms	Z	Observed(°)	Ideal(°)
5	A	901	GH3	C6-N1-C2	5.64	124.89	115.93
5	A	902	GH3	C6-N1-C2	5.63	124.87	115.93
5	A	901	GH3	O4'-C4'-C5'	5.19	118.05	109.52
5	A	902	GH3	N3-C2-N1	-4.44	121.31	127.22
5	A	901	GH3	N3-C2-N1	-4.02	121.86	127.22
5	A	902	GH3	O4'-C4'-C5'	3.80	115.77	109.52
5	A	901	GH3	O4'-C4'-C3'	3.52	109.61	105.07
5	A	902	GH3	PB-O3B-PG	-3.52	120.74	132.83
5	A	902	GH3	O4'-C4'-C3'	2.95	108.88	105.07
5	A	901	GH3	C6-C5-C4	-2.94	117.99	120.80
5	A	901	GH3	PB-O3B-PG	-2.92	122.79	132.83
5	A	902	GH3	O2G-PG-O3B	2.41	112.72	104.64
5	A	902	GH3	C6-C5-C4	-2.13	118.76	120.80
5	A	901	GH3	O2G-PG-O3B	2.02	111.42	104.64

There are no chirality outliers.

All (8) torsion outliers are listed below:

Mol	Chain	Res	Type	Atoms
5	A	902	GH3	PB-O3A-PA-O5'
5	A	901	GH3	C3'-C4'-C5'-O5'
5	A	902	GH3	O4'-C4'-C5'-O5'
5	A	901	GH3	O4'-C4'-C5'-O5'
5	A	902	GH3	C3'-C4'-C5'-O5'
5	A	902	GH3	PA-O3A-PB-O2B
5	A	901	GH3	PG-O3B-PB-O2B
5	A	902	GH3	C5'-O5'-PA-O1A

There are no ring outliers.

No monomer is involved in short contacts.

The following is a two-dimensional graphical depiction of Mogul quality analysis of bond lengths, bond angles, torsion angles, and ring geometry for all instances of the Ligand of Interest. In addition, ligands with molecular weight > 250 and outliers as shown on the validation Tables will also be included. For torsion angles, if less then 5% of the Mogul distribution of torsion angles is within 10 degrees of the torsion angle in question, then that torsion angle is considered an outlier. Any bond that is central to one or more torsion angles identified as an outlier by Mogul will be highlighted in the graph. For rings, the root-mean-square deviation (RMSD) between the ring in question and similar rings identified by Mogul is calculated over all ring torsion angles. If the average RMSD is greater than 60 degrees and the minimal RMSD between the ring in question and any Mogul-identified rings is also greater than 60 degrees, then that ring is considered an outlier. The outliers are highlighted in purple. The color gray indicates Mogul did not find sufficient

equivalents in the CSD to analyse the geometry.

Ligand GH3 A 902

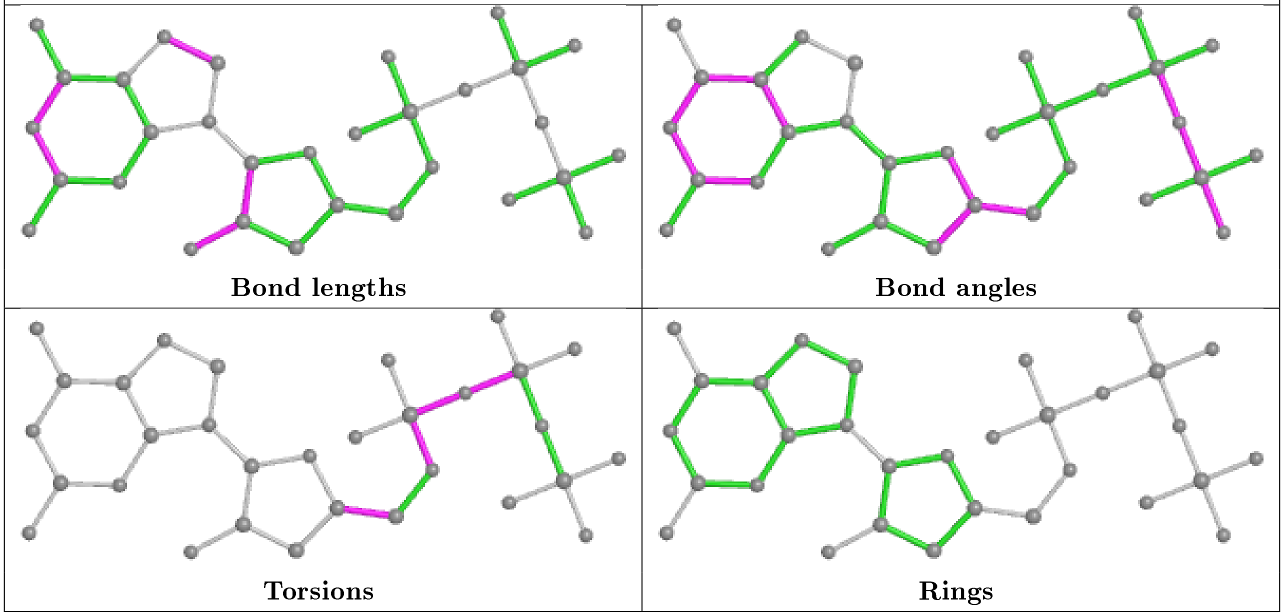

Ligand GH3 A 901

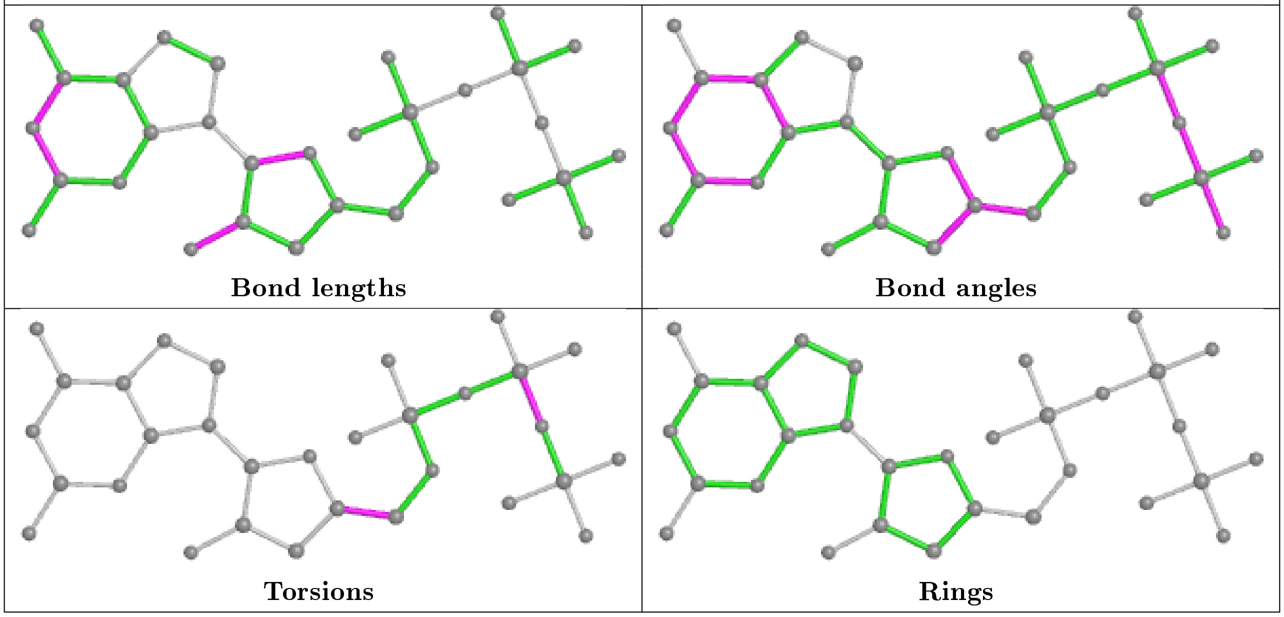

5.7 Other polymers (i)

There are no such residues in this entry.

5.8 Polymer linkage issues (i)

There are no chain breaks in this entry.

6 Fit of model and data (i)

6.1 Protein, DNA and RNA chains (i)

In the following table, the column labelled '#RSRZ> 2' contains the number (and percentage) of RSRZ outliers, followed by percent RSRZ outliers for the chain as percentile scores relative to all X-ray entries and entries of similar resolution. The OWAB column contains the minimum, median, 95th percentile and maximum values of the occupancy-weighted average B-factor per residue. The column labelled 'Q< 0.9' lists the number of (and percentage) of residues with an average occupancy less than 0.9.

Mol	Chain	Analysed	<RSRZ>	#RSRZ>2			OWAB(Å^2)	Q<0.9
1	T	22/22 (100%)	-0.31	0	100	100	48, 60, 79, 120	0
2	P	14/14 (100%)	-0.77	0	100	100	37, 47, 95, 126	0
3	A	862/878 (98%)	0.48	73 (8%)	10	10	0, 58, 121, 160	0
All	All	898/914 (98%)	0.44	73 (8%)	12	12	0, 58, 121, 160	0

All (73) RSRZ outliers are listed below:

Mol	Chain	Res	Type	RSRZ
3	A	273	VAL	5.6
3	A	845	PHE	4.8
3	A	629	VAL	4.7
3	A	543	ILE	4.3
3	A	175	GLY	4.3
3	A	200	ALA	4.1
3	A	24	LEU	4.0
3	A	535	ALA	3.9
3	A	176	HIS	3.8
3	A	788	GLY	3.6
3	A	533	PRO	3.6
3	A	881	ALA	3.5
3	A	725	VAL	3.4
3	A	178	TYR	3.4
3	A	633	SER	3.3
3	A	780	PRO	3.3
3	A	825	PHE	3.3
3	A	821	ALA	3.2
3	A	827	ALA	3.1
3	A	28	TYR	3.0
3	A	415	TRP	3.0
3	A	308	TYR	3.0
3	A	715	THR	3.0

Continued on next page...

Continued from previous page...

Mol	Chain	Res	Type	RSRZ
3	A	737	GLN	3.0
3	A	546	PHE	3.0
3	A	875	ILE	2.9
3	A	777	GLY	2.9
3	A	685	VAL	2.9
3	A	620	TRP	2.8
3	A	828	VAL	2.8
3	A	414	ILE	2.8
3	A	807	PHE	2.8
3	A	724	ALA	2.8
3	A	539	SER	2.7
3	A	286	TYR	2.7
3	A	791	LEU	2.6
3	A	804	ILE	2.6
3	A	545	HIS	2.5
3	A	853	LEU	2.5
3	A	201	TRP	2.5
3	A	214	VAL	2.5
3	A	488	ASN	2.5
3	A	426	VAL	2.4
3	A	183	MET	2.4
3	A	427	TYR	2.4
3	A	736	TRP	2.4
3	A	326	THR	2.4
3	A	452	ILE	2.3
3	A	184	GLN	2.3
3	A	883	ALA	2.3
3	A	582	LEU	2.3
3	A	784	HIS	2.2
3	A	832	MET	2.2
3	A	420	MET	2.2
3	A	635	MET	2.1
3	A	806	SER	2.1
3	A	323	ALA	2.1
3	A	422	TRP	2.1
3	A	726	HIS	2.1
3	A	328	TRP	2.1
3	A	293	PRO	2.1
3	A	316	VAL	2.1
3	A	44	TYR	2.1
3	A	396	ILE	2.1
3	A	842	LEU	2.1

Continued on next page...

Continued from previous page...

Mol	Chain	Res	Type	RSRZ
3	A	204	TRP	2.1
3	A	149	ALA	2.0
3	A	311	VAL	2.0
3	A	811	HIS	2.0
3	A	185	VAL	2.0
3	A	829	ARG	2.0
3	A	170	LEU	2.0
3	A	824	LEU	2.0

6.2 Non-standard residues in protein, DNA, RNA chains (i)

There are no non-standard protein/DNA/RNA residues in this entry.

6.3 Carbohydrates (i)

There are no carbohydrates in this entry.

6.4 Ligands (i)

In the following table, the Atoms column lists the number of modelled atoms in the group and the number defined in the chemical component dictionary. The B-factors column lists the minimum, median, 95th percentile and maximum values of B factors of atoms in the group. The column labelled 'Q< 0.9' lists the number of atoms with occupancy less than 0.9.

Mol	Type	Chain	Res	Atoms	RSCC	RSR	B-factors($Å^2$)	Q<0.9
4	MG	A	885	1/1	0.16	2.31	42,42,42,42	0
4	MG	A	884	1/1	0.26	0.28	23,23,23,23	0
5	GH3	A	902	31/31	0.85	0.20	60,63,70,72	0
5	GH3	A	901	31/31	0.87	0.24	70,78,90,91	0

The following is a graphical depiction of the model fit to experimental electron density of all instances of the Ligand of Interest. In addition, ligands with molecular weight > 250 and outliers as shown on the geometry validation Tables will also be included. Each fit is shown from different orientation to approximate a three-dimensional view.

Electron density around GH3 A 902:

$2mF_o$-DF_c (at 0.7 rmsd) in gray
mF_o-DF_c (at 3 rmsd) in purple (negative)
and green (positive)

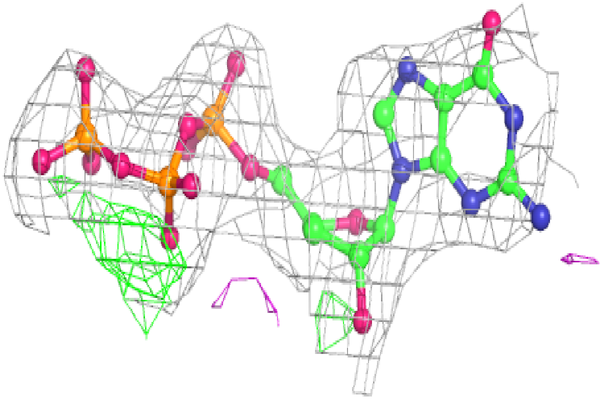

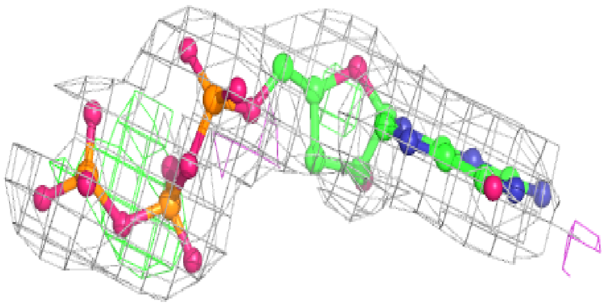

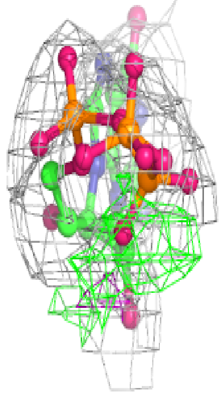

Electron density around GH3 A 901:

$2mF_o$-DF_c (at 0.7 rmsd) in gray
mF_o-DF_c (at 3 rmsd) in purple (negative)
and green (positive)

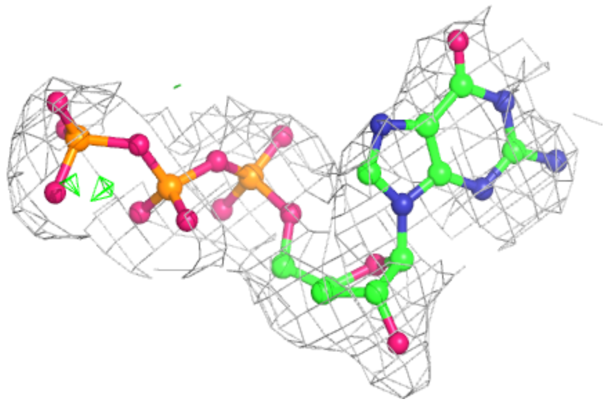

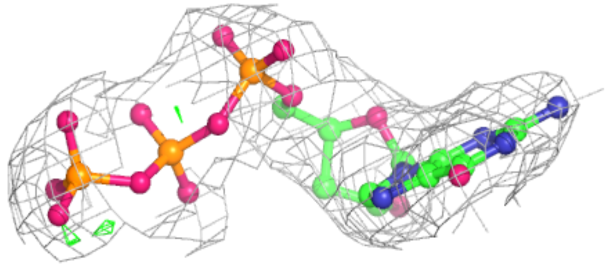

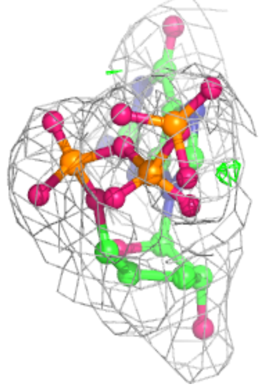

6.5 Other polymers ⓘ

There are no such residues in this entry.
