# Supplementary material for: An engineered T7 RNA polymerase that produces mRNA free of immunostimulatory byproducts
Source: Nat Biotechnol. 2022 Nov 10;41(4):560–8. doi: 10.1038/s41587-022-01525-6 (PMC10110463; doi:10.1038/s41587-022-01525-6)
Supplement: Supplementary file 8 — Full validation report for PDB entry 3E2E. [file 41587_2022_1525_MOESM8_ESM.pdf]

Full wwPDB X-ray Structure Validation Report (i)

Oct 10, 2021 – 08:04 PM EDT

PDB ID	:	3E2E
Title	:	Crystal Structure of an Intermediate Complex of T7 RNAP and 7nt of RNA
Authors	:	Durniak, K.J.; Bailey, S.; Steitz, T.A.
Deposited on	:	2008-08-05
Resolution	:	3.00 Å(reported)

This is a Full wwPDB X-ray Structure Validation Report for a publicly released PDB entry.

We welcome your comments at *validation@mail.wwpdb.org*
A user guide is available at
https://www.wwpdb.org/validation/2017/XrayValidationReportHelp
with specific help available everywhere you see the (i) symbol.

The following versions of software and data (see references (i)) were used in the production of this report:

MolProbity	:	4.02b-467
Xtriage (Phenix)	:	1.13
EDS	:	2.23.2
Percentile statistics	:	20191225.v01 (using entries in the PDB archive December 25th 2019)
Refmac	:	5.8.0158
CCP4	:	7.0.044 (Gargrove)
Ideal geometry (proteins)	:	Engh & Huber (2001)
Ideal geometry (DNA, RNA)	:	Parkinson et al. (1996)
Validation Pipeline (wwPDB-VP)	:	2.23.2

1 Overall quality at a glance (i)

The following experimental techniques were used to determine the structure:
X-RAY DIFFRACTION

The reported resolution of this entry is 3.00 Å.

Percentile scores (ranging between 0-100) for global validation metrics of the entry are shown in the following graphic. The table shows the number of entries on which the scores are based.

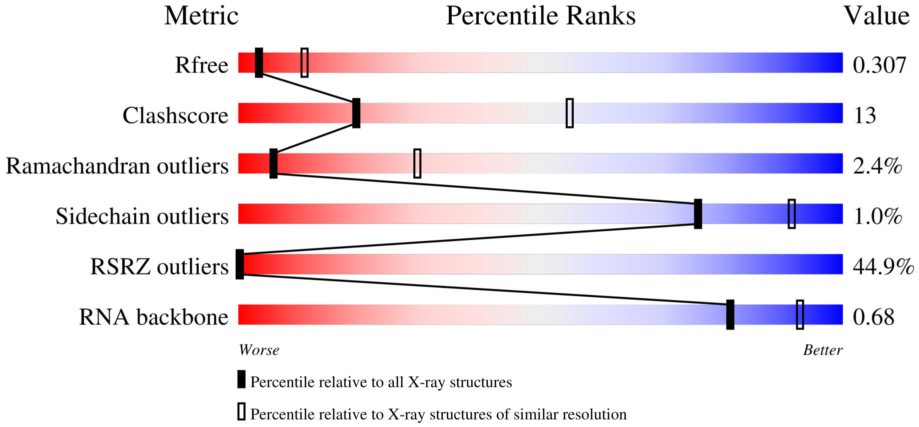

Metric	Whole archive (#Entries)	Similar resolution (#Entries, resolution range(Å))
R_{free}	130704	2092 (3.00-3.00)
Clashscore	141614	2416 (3.00-3.00)
Ramachandran outliers	138981	2333 (3.00-3.00)
Sidechain outliers	138945	2336 (3.00-3.00)
RSRZ outliers	127900	1990 (3.00-3.00)
RNA backbone	3102	1173 (3.30-2.70)

The table below summarises the geometric issues observed across the polymeric chains and their fit to the electron density. The red, orange, yellow and green segments of the lower bar indicate the fraction of residues that contain outliers for >=3, 2, 1 and 0 types of geometric quality criteria respectively. A grey segment represents the fraction of residues that are not modelled. The numeric value for each fraction is indicated below the corresponding segment, with a dot representing fractions <=5% The upper red bar (where present) indicates the fraction of residues that have poor fit to the electron density. The numeric value is given above the bar.

Mol	Chain	Length	Quality of chain
1	A	889	38%<br>72% 19% • 6%
2	R	7	100%<br>86% 14%
3	T	33	91%<br>52% 39% • 6%
4	N	33	82%<br>55% 24% 6% 15%

2 Entry composition (i)

There are 5 unique types of molecules in this entry. The entry contains 8013 atoms, of which 0 are hydrogens and 0 are deuteriums.

In the tables below, the ZeroOcc column contains the number of atoms modelled with zero occupancy, the AltConf column contains the number of residues with at least one atom in alternate conformation and the Trace column contains the number of residues modelled with at most 2 atoms.

- Molecule 1 is a protein called DNA-directed RNA polymerase.

Mol	Chain	Residues	Atoms					ZeroOcc	AltConf	Trace
			Total	C	N	O	S			
1	A	832	6589	4206	1139	1207	37	0	0	0

There are 7 discrepancies between the modelled and reference sequences:

Chain	Residue	Modelled	Actual	Comment	Reference
A	-5	HIS	-	expression tag	UNP P00573
A	-4	HIS	-	expression tag	UNP P00573
A	-3	HIS	-	expression tag	UNP P00573
A	-2	HIS	-	expression tag	UNP P00573
A	-1	HIS	-	expression tag	UNP P00573
A	0	HIS	-	expression tag	UNP P00573
A	266	LEU	PRO	engineered mutation	UNP P00573

- Molecule 2 is a RNA chain called RNA (5'-R(*GP*GP*GP*AP*GP*UP*G)-3').

Mol	Chain	Residues	Atoms					ZeroOcc	AltConf	Trace
			Total	C	N	O	P			
2	R	7	153	69	32	46	6	0	0	0

- Molecule 3 is a DNA chain called DNA (31-MER).

Mol	Chain	Residues	Atoms					ZeroOcc	AltConf	Trace
			Total	C	N	O	P			
3	T	31	633	301	113	188	31	0	0	0

- Molecule 4 is a DNA chain called DNA (28-MER).

Mol	Chain	Residues	Atoms					ZeroOcc	AltConf	Trace
			Total	C	N	O	P			
4	N	28	565	271	101	166	27	0	0	0

- Molecule 5 is water.

Mol	Chain	Residues	Atoms	ZeroOcc	AltConf
5	A	64	Total O<br>64 64	0	0
5	R	1	Total O<br>1 1	0	0
5	T	3	Total O<br>3 3	0	0
5	N	5	Total O<br>5 5	0	0

3 Residue-property plots (i)

These plots are drawn for all protein, RNA, DNA and oligosaccharide chains in the entry. The first graphic for a chain summarises the proportions of the various outlier classes displayed in the second graphic. The second graphic shows the sequence view annotated by issues in geometry and electron density. Residues are color-coded according to the number of geometric quality criteria for which they contain at least one outlier: green = 0, yellow = 1, orange = 2 and red = 3 or more. A red dot above a residue indicates a poor fit to the electron density (RSRZ > 2). Stretches of 2 or more consecutive residues without any outlier are shown as a green connector. Residues present in the sample, but not in the model, are shown in grey.

- Molecule 1: DNA-directed RNA polymerase

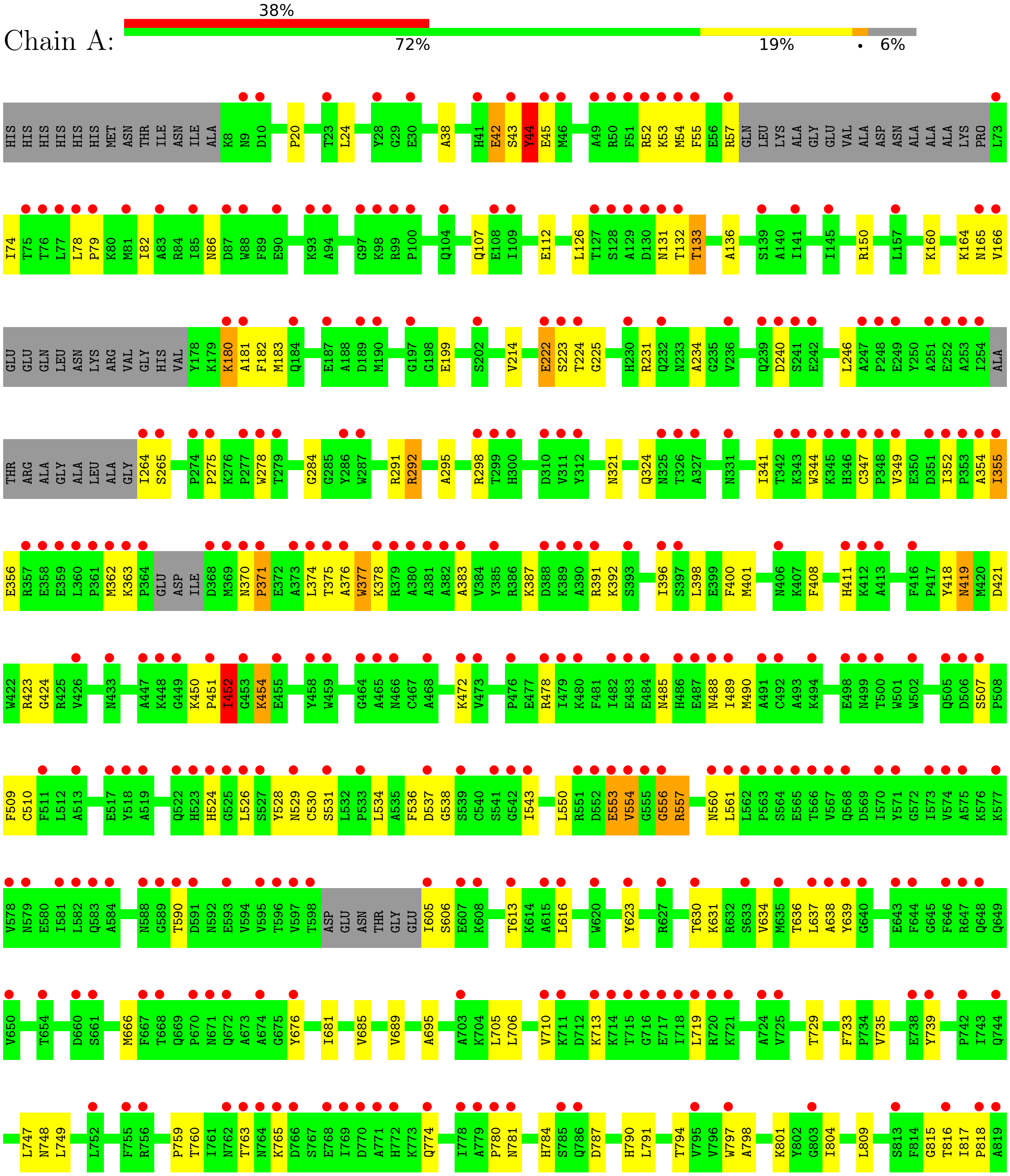

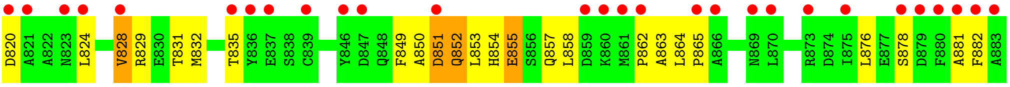

• Molecule 2: RNA (5'-R(*GP*GP*GP*AP*GP*UP*G)-3')

Chain R:

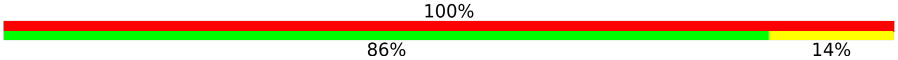

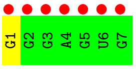

• Molecule 3: DNA (31-MER)

Chain T:

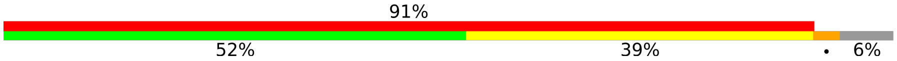

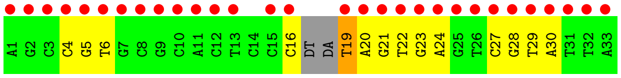

• Molecule 4: DNA (28-MER)

Chain N:

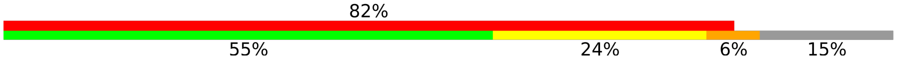

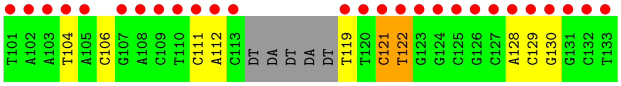

4 Data and refinement statistics (i)

Property	Value	Source
Space group	P 61	Depositor
Cell constants<br>a, b, c, α, β, γ	81.01Å 81.01Å 358.79Å<br>90.00° 90.00° 120.00°	Depositor
Resolution (Å)	37.80 – 3.00<br>37.79 – 3.00	Depositor<br>EDS
% Data completeness<br>(in resolution range)	98.5 (37.80-3.00)<br>98.5 (37.79-3.00)	Depositor<br>EDS
R_{merge}	0.08	Depositor
R_{sym}	(Not available)	Depositor
$< I/\sigma(I) >$ [1]	2.31 (at 3.01Å)	Xtriage
Refinement program	REFMAC 5.2.0019	Depositor
R, R_{free}	0.240 , 0.292<br>0.251 , 0.307	Depositor<br>DCC
R_{free} test set	1330 reflections (5.10%)	wwPDB-VP
Wilson B-factor (Å^2)	37.9	Xtriage
Anisotropy	0.104	Xtriage
Bulk solvent k_{sol}(e/Å^3), B_{sol}(Å^2)	0.27 , 85.5	EDS
L-test for twinning[2]	$< \|L\| > = 0.48$, $< L^2 > = 0.31$	Xtriage
Estimated twinning fraction	0.079 for h,-h-k,-l	Xtriage
F_o,F_c correlation	0.84	EDS
Total number of atoms	8013	wwPDB-VP
Average B, all atoms (Å^2)	99.0	wwPDB-VP

Xtriage's analysis on translational NCS is as follows: *The largest off-origin peak in the Patterson function is 2.89% of the height of the origin peak. No significant pseudotranslation is detected.*

[1]Intensities estimated from amplitudes.

[2]Theoretical values of $< |L| >$, $< L^2 >$ for acentric reflections are 0.5, 0.333 respectively for untwinned datasets, and 0.375, 0.2 for perfectly twinned datasets.

5 Model quality (i)

5.1 Standard geometry (i)

The Z score for a bond length (or angle) is the number of standard deviations the observed value is removed from the expected value. A bond length (or angle) with $|Z| > 5$ is considered an outlier worth inspection. RMSZ is the root-mean-square of all Z scores of the bond lengths (or angles).

Mol	Chain	Bond lengths		Bond angles	
		RMSZ	#$\|Z\|$ >5	RMSZ	#$\|Z\|$ >5
1	A	0.34	0/6738	0.58	4/9109 (0.0%)
2	R	0.65	0/172	1.12	0/269
3	T	0.63	0/707	1.24	1/1086 (0.1%)
4	N	0.65	0/631	1.33	4/969 (0.4%)
All	All	0.41	0/8248	0.77	9/11433 (0.1%)

There are no bond length outliers.

All (9) bond angle outliers are listed below:

Mol	Chain	Res	Type	Atoms	Z	Observed(^{o})	Ideal(^{o})
3	T	19	DT	O4'-C1'-N1	8.94	114.26	108.00
4	N	122	DT	O4'-C1'-N1	7.05	112.93	108.00
1	A	43	SER	N-CA-C	6.99	129.88	111.00
4	N	121	DC	P-O3'-C3'	6.39	127.37	119.70
4	N	104	DT	C1'-O4'-C4'	-6.28	103.82	110.10
4	N	119	DT	O4'-C4'-C3'	-6.05	102.08	104.50
1	A	553	GLU	C-N-CA	5.83	136.28	121.70
1	A	43	SER	C-N-CA	5.82	136.25	121.70
1	A	44	TYR	N-CA-C	5.36	125.46	111.00

There are no chirality outliers.

There are no planarity outliers.

5.2 Too-close contacts (i)

In the following table, the Non-H and H(model) columns list the number of non-hydrogen atoms and hydrogen atoms in the chain respectively. The H(added) column lists the number of hydrogen atoms added and optimized by MolProbity. The Clashes column lists the number of clashes within the asymmetric unit, whereas Symm-Clashes lists symmetry-related clashes.

Mol	Chain	Non-H	H(model)	H(added)	Clashes	Symm-Clashes
1	A	6589	0	6558	176	0
2	R	153	0	75	4	0
3	T	633	0	351	19	0
4	N	565	0	315	5	0
5	A	64	0	0	9	0
5	N	5	0	0	0	0
5	R	1	0	0	0	0
5	T	3	0	0	1	0
All	All	8013	0	7299	195	0

The all-atom clashscore is defined as the number of clashes found per 1000 atoms (including hydrogen atoms). The all-atom clashscore for this structure is 13.

All (195) close contacts within the same asymmetric unit are listed below, sorted by their clash magnitude.

Atom-1	Atom-2	Interatomic distance (Å)	Clash overlap (Å)
1:A:376:ALA:HB3	1:A:377:TRP:CB	1.72	1.19
1:A:553:GLU:CG	1:A:554:VAL:HG23	1.87	1.04
1:A:354:ALA:CA	1:A:355:ILE:HG12	1.88	1.04
1:A:131:ASN:HB3	1:A:132:THR:HA	1.06	1.03
1:A:354:ALA:HA	1:A:355:ILE:CG1	1.87	1.03
1:A:354:ALA:HB1	1:A:356:GLU:H	1.24	1.00
1:A:553:GLU:HG3	1:A:554:VAL:HG23	1.40	1.00
1:A:354:ALA:HA	1:A:355:ILE:HG12	1.01	1.00
1:A:131:ASN:CB	1:A:132:THR:HA	1.92	0.98
1:A:376:ALA:HB3	1:A:377:TRP:HB3	0.98	0.97
1:A:376:ALA:CB	1:A:377:TRP:HB3	1.94	0.96
1:A:451:PRO:HA	1:A:452:ILE:CB	1.99	0.93
1:A:451:PRO:HA	1:A:452:ILE:CG1	1.99	0.93
1:A:882:PHE:HA	5:A:907:HOH:O	1.69	0.92
1:A:556:GLY:HA3	1:A:557:ARG:HB2	1.51	0.91
3:T:19:DT:H2”	3:T:20:DA:OP1	1.69	0.89
1:A:370:ASN:HB2	1:A:371:PRO:HD2	1.55	0.89
1:A:131:ASN:HB3	1:A:132:THR:CA	2.00	0.85
1:A:349:VAL:CG2	1:A:352:ILE:HG12	2.06	0.85
1:A:850:ALA:HA	1:A:851:ASP:C	1.98	0.82
1:A:53:LYS:HB3	1:A:54:MET:HB2	1.63	0.79
1:A:556:GLY:HA3	1:A:557:ARG:CB	2.13	0.79
1:A:278:TRP:H	1:A:321:ASN:HD21	1.31	0.79
1:A:376:ALA:CB	1:A:377:TRP:CB	2.56	0.78
1:A:553:GLU:HG2	1:A:554:VAL:HG23	1.69	0.75
1:A:863:ALA:HB3	1:A:864:LEU:HA	1.68	0.74

Continued on next page...

Continued from previous page...

Atom-1	Atom-2	Interatomic distance (Å)	Clash overlap (Å)
1:A:451:PRO:HA	1:A:452:ILE:HB	1.70	0.73
1:A:354:ALA:HB3	1:A:391:ARG:HE	1.52	0.73
1:A:222:GLU:HB3	1:A:223:SER:HB3	1.68	0.73
1:A:354:ALA:HB1	1:A:356:GLU:N	2.03	0.72
1:A:451:PRO:HA	1:A:452:ILE:HG13	1.71	0.71
1:A:864:LEU:HD12	1:A:865:PRO:HD2	1.73	0.70
1:A:553:GLU:HG3	1:A:554:VAL:CG2	2.20	0.70
1:A:349:VAL:HG23	1:A:352:ILE:HG12	1.73	0.69
1:A:816:THR:HG22	1:A:817:ILE:N	2.08	0.69
1:A:710:VAL:HG21	1:A:719:LEU:HB2	1.76	0.68
1:A:550:LEU:HD11	1:A:695:ALA:HB2	1.75	0.68
1:A:784:HIS:HA	1:A:787:ASP:OD1	1.95	0.67
1:A:862:PRO:HB2	1:A:863:ALA:HB2	1.78	0.66
1:A:816:THR:HG22	1:A:817:ILE:H	1.60	0.65
1:A:375:THR:OG1	1:A:376:ALA:HA	1.97	0.65
1:A:739:TYR:H	1:A:774:GLN:NE2	1.95	0.65
2:R:1:G:C4	3:T:19:DT:H5”	2.32	0.65
1:A:452:ILE:HD11	1:A:529:ASN:HA	1.79	0.64
2:R:1:G:C5	3:T:19:DT:H5”	2.34	0.62
1:A:507:SER:HB3	1:A:510:CYS:HB2	1.80	0.62
1:A:556:GLY:CA	1:A:557:ARG:CB	2.77	0.62
1:A:224:THR:N	1:A:225:GLY:HA2	2.15	0.61
1:A:150:ARG:HH22	1:A:199:GLU:HB3	1.66	0.60
1:A:52:ARG:HA	1:A:55:PHE:HB2	1.84	0.59
1:A:180:LYS:HB3	1:A:181:ALA:HA	1.83	0.59
1:A:748:ASN:HB3	4:N:106:DC:H2”	1.84	0.59
1:A:489:ILE:HG22	1:A:490:MET:H	1.68	0.59
1:A:291:ARG:O	1:A:292:ARG:HB3	2.03	0.58
1:A:850:ALA:HA	1:A:851:ASP:O	2.02	0.58
1:A:452:ILE:CD1	1:A:529:ASN:HA	2.34	0.58
1:A:376:ALA:H	1:A:378:LYS:N	2.01	0.58
1:A:472:LYS:O	1:A:472:LYS:HG2	2.04	0.58
1:A:278:TRP:H	1:A:321:ASN:ND2	1.99	0.57
1:A:729:THR:HG22	1:A:733:PHE:HB3	1.86	0.57
1:A:710:VAL:HG11	1:A:719:LEU:N	2.19	0.57
1:A:863:ALA:HB3	1:A:864:LEU:CA	2.34	0.57
3:T:6:DT:H3	4:N:128:DA:H61	1.53	0.56
1:A:352:ILE:HD12	1:A:398:LEU:HD11	1.86	0.56
1:A:804:ILE:HG12	1:A:820:ASP:HB3	1.87	0.56
1:A:349:VAL:HG21	1:A:352:ILE:HG12	1.83	0.56
4:N:111:DC:H2”	4:N:112:DA:C8	2.40	0.56

Continued on next page...

Continued from previous page...

Atom-1	Atom-2	Interatomic distance (Å)	Clash overlap (Å)
1:A:710:VAL:HG11	1:A:719:LEU:H	1.71	0.56
1:A:829:ARG:NH2	5:A:907:HOH:O	2.38	0.56
1:A:180:LYS:HE3	1:A:183:MET:HG2	1.88	0.56
1:A:20:PRO:O	1:A:24:LEU:HB2	2.06	0.55
1:A:222:GLU:H	1:A:223:SER:C	2.09	0.55
3:T:22:DT:H2'	3:T:23:DG:C8	2.42	0.55
4:N:121:DC:H4'	4:N:122:DT:OP1	2.07	0.55
1:A:729:THR:CG2	1:A:733:PHE:HB3	2.37	0.55
1:A:224:THR:H	1:A:225:GLY:HA2	1.71	0.54
1:A:324:GLN:HA	1:A:418:TYR:HD1	1.72	0.54
1:A:760:THR:O	3:T:20:DA:H2'	2.08	0.54
1:A:639:TYR:HB3	1:A:780:PRO:HB3	1.89	0.54
1:A:681:ILE:O	1:A:685:VAL:HG12	2.08	0.54
1:A:560:ASN:O	1:A:881:ALA:HB2	2.08	0.53
1:A:383:ALA:O	1:A:387:LYS:HG3	2.09	0.53
1:A:424:GLY:HA3	5:A:897:HOH:O	2.08	0.53
1:A:408:PHE:HA	1:A:411:HIS:CD2	2.43	0.53
1:A:291:ARG:O	1:A:292:ARG:CB	2.57	0.53
1:A:375:THR:O	1:A:378:LYS:HB2	2.08	0.53
1:A:763:THR:HG22	1:A:765:LYS:H	1.73	0.53
1:A:180:LYS:HA	1:A:182:PHE:N	2.25	0.52
1:A:423:ARG:HH11	1:A:781:ASN:HD22	1.56	0.52
1:A:53:LYS:CB	1:A:54:MET:HB2	2.38	0.52
1:A:126:LEU:HD13	1:A:246:LEU:HB2	1.92	0.52
2:R:1:G:C4	3:T:19:DT:C5'	2.93	0.52
1:A:524:HIS:HB2	1:A:528:TYR:HB2	1.92	0.52
1:A:862:PRO:HB2	1:A:863:ALA:CB	2.40	0.51
3:T:20:DA:H8	3:T:20:DA:O5'	1.93	0.51
1:A:739:TYR:H	1:A:774:GLN:HE21	1.57	0.51
1:A:180:LYS:HG2	1:A:182:PHE:HD1	1.76	0.51
1:A:862:PRO:HB2	1:A:863:ALA:CA	2.41	0.51
1:A:264:ILE:N	1:A:265:SER:HB3	2.25	0.51
1:A:705:LEU:HB3	1:A:857:GLN:HE21	1.76	0.51
1:A:324:GLN:HE21	1:A:418:TYR:H	1.58	0.51
1:A:851:ASP:O	1:A:853:LEU:N	2.44	0.50
3:T:4:DC:H6	5:T:36:HOH:O	1.94	0.50
1:A:531:SER:HA	1:A:817:ILE:HG22	1.94	0.50
1:A:133:THR:HG23	1:A:136:ALA:HB2	1.93	0.50
1:A:537:ASP:O	1:A:882:PHE:HB2	2.12	0.49
1:A:362:MET:H	1:A:377:TRP:HE1	1.60	0.49
1:A:214:VAL:HG11	1:A:749:LEU:HD13	1.94	0.49

Continued on next page...

Continued from previous page...

Atom-1	Atom-2	Interatomic distance (Å)	Clash overlap (Å)
1:A:829:ARG:NH1	1:A:878:SER:O	2.45	0.49
1:A:543:ILE:HD13	1:A:689:VAL:HG11	1.94	0.49
1:A:341:ILE:HD11	1:A:509:PHE:CZ	2.47	0.49
1:A:222:GLU:N	1:A:223:SER:C	2.66	0.49
1:A:376:ALA:H	1:A:378:LYS:H	1.61	0.48
1:A:747:LEU:HB2	1:A:759:PRO:HD2	1.96	0.48
1:A:816:THR:CG2	1:A:817:ILE:N	2.76	0.48
1:A:538:GLY:HA2	5:A:887:HOH:O	2.13	0.48
1:A:421:ASP:O	1:A:423:ARG:O	2.32	0.47
1:A:298:ARG:HH21	1:A:419:ASN:HB2	1.79	0.47
1:A:454:LYS:H	1:A:526:LEU:HD22	1.80	0.47
1:A:829:ARG:HB2	1:A:876:LEU:HD23	1.97	0.47
1:A:831:THR:O	1:A:835:THR:HG23	2.15	0.47
1:A:706:LEU:HD11	1:A:849:PHE:HB2	1.97	0.47
1:A:798:ALA:HB1	1:A:804:ILE:HD12	1.96	0.47
1:A:536:PHE:HB3	1:A:882:PHE:HB3	1.96	0.46
1:A:107:GLN:HB2	5:A:938:HOH:O	2.15	0.46
1:A:133:THR:HG23	1:A:136:ALA:CB	2.45	0.46
1:A:623:TYR:HB2	1:A:666:MET:SD	2.54	0.46
1:A:637:LEU:C	1:A:639:TYR:H	2.19	0.46
1:A:347:CYS:SG	1:A:349:VAL:HG22	2.56	0.46
3:T:16:DC:H2"	3:T:19:DT:O4	2.16	0.46
1:A:864:LEU:HD12	1:A:865:PRO:CD	2.44	0.46
1:A:530:CYS:SG	1:A:818:PRO:HG2	2.56	0.45
1:A:354:ALA:CB	1:A:355:ILE:HG12	2.42	0.45
1:A:794:THR:OG1	1:A:831:THR:HG21	2.15	0.45
1:A:223:SER:HA	1:A:224:THR:HA	1.70	0.45
1:A:278:TRP:CD2	1:A:284:GLY:HA3	2.52	0.45
1:A:854:HIS:O	1:A:855:GLU:HB2	2.16	0.45
1:A:816:THR:CG2	1:A:817:ILE:H	2.27	0.45
1:A:131:ASN:CB	1:A:132:THR:CA	2.76	0.45
1:A:165:ASN:HA	1:A:166:VAL:HA	1.67	0.45
1:A:376:ALA:CB	1:A:377:TRP:HB2	2.45	0.45
1:A:400:PHE:HD1	1:A:401:MET:HE2	1.82	0.45
1:A:790:HIS:NE2	1:A:832:MET:HB2	2.32	0.45
2:R:1:G:C2	3:T:19:DT:H5'	2.52	0.45
1:A:489:ILE:HG22	1:A:490:MET:N	2.32	0.45
1:A:824:LEU:O	1:A:828:VAL:HG22	2.17	0.45
3:T:20:DA:O5'	3:T:20:DA:C8	2.70	0.45
4:N:129:DC:H2"	4:N:130:DG:C8	2.52	0.45
1:A:82:ILE:HD13	1:A:112:GLU:HG3	1.98	0.44

Continued on next page...

Continued from previous page...

Atom-1	Atom-2	Interatomic distance (Å)	Clash overlap (Å)
1:A:791:LEU:HD21	1:A:809:LEU:HD13	1.99	0.44
1:A:797:TRP:CZ2	1:A:801:LYS:HD2	2.52	0.44
3:T:23:DG:H2”	3:T:24:DA:C8	2.53	0.44
1:A:376:ALA:N	1:A:378:LYS:H	2.15	0.44
1:A:534:LEU:O	1:A:815:GLY:HA2	2.18	0.44
1:A:354:ALA:CB	1:A:356:GLU:H	2.12	0.44
1:A:231:ARG:HE	1:A:234:ALA:HB2	1.83	0.43
1:A:760:THR:O	3:T:20:DA:C2’	2.65	0.43
3:T:4:DC:H2”	3:T:5:DG:C8	2.52	0.43
1:A:392:LYS:O	1:A:396:ILE:HG12	2.19	0.43
1:A:849:PHE:O	1:A:852:GLN:HB2	2.18	0.43
1:A:705:LEU:O	1:A:857:GLN:NE2	2.52	0.43
1:A:160:LYS:O	1:A:164:LYS:HG3	2.19	0.43
1:A:451:PRO:CA	1:A:452:ILE:HB	2.45	0.43
1:A:324:GLN:HA	1:A:418:TYR:CD1	2.52	0.43
1:A:53:LYS:O	1:A:57:ARG:HB3	2.19	0.43
1:A:605:ILE:HA	1:A:606:SER:HA	1.77	0.43
3:T:19:DT:H3’	3:T:20:DA:H5’	2.00	0.43
1:A:485:ASN:HB3	1:A:488:ASN:HB2	2.00	0.42
1:A:590:THR:HB	1:A:613:THR:H	1.84	0.42
1:A:616:LEU:HD13	1:A:676:TYR:HB2	2.00	0.42
1:A:374:LEU:C	1:A:376:ALA:HB2	2.40	0.42
3:T:27:DC:H2”	3:T:28:DG:C8	2.54	0.42
1:A:862:PRO:HB2	1:A:863:ALA:HA	2.02	0.42
1:A:344:TRP:O	1:A:355:ILE:HD12	2.19	0.42
1:A:354:ALA:HA	1:A:355:ILE:CB	2.46	0.42
1:A:295:ALA:O	1:A:419:ASN:ND2	2.53	0.42
1:A:556:GLY:O	1:A:561:LEU:HB2	2.19	0.42
1:A:636:THR:HA	1:A:639:TYR:HD2	1.84	0.42
1:A:355:ILE:HA	5:A:918:HOH:O	2.19	0.41
1:A:478:ARG:HH12	1:A:882:PHE:HZ	1.67	0.41
3:T:29:DT:H2”	3:T:30:DA:C8	2.55	0.41
1:A:42:GLU:HA	1:A:45:GLU:HB2	2.03	0.41
1:A:450:LYS:O	1:A:452:ILE:HG13	2.21	0.41
1:A:78:LEU:N	1:A:79:PRO:HD2	2.35	0.41
1:A:452:ILE:HG22	1:A:526:LEU:O	2.20	0.41
1:A:38:ALA:O	1:A:42:GLU:HB2	2.21	0.41
1:A:630:THR:O	1:A:634:VAL:HG23	2.21	0.41
1:A:451:PRO:HA	1:A:452:ILE:CD1	2.49	0.40
1:A:240:ASP:O	3:T:21:DG:H2”	2.21	0.40
1:A:53:LYS:HB3	1:A:54:MET:CB	2.43	0.40

Continued on next page...

Continued from previous page...

Atom-1	Atom-2	Interatomic distance (Å)	Clash overlap (Å)
1:A:150:ARG:NH1	5:A:940:HOH:O	2.54	0.40
1:A:275:PRO:HG2	1:A:324:GLN:HG2	2.03	0.40
1:A:86:ASN:HD22	1:A:86:ASN:HA	1.74	0.40
1:A:74:ILE:HG13	5:A:903:HOH:O	2.20	0.40
1:A:452:ILE:HG23	5:A:933:HOH:O	2.21	0.40

There are no symmetry-related clashes.

5.3 Torsion angles (i)

5.3.1 Protein backbone (i)

In the following table, the Percentiles column shows the percent Ramachandran outliers of the chain as a percentile score with respect to all X-ray entries followed by that with respect to entries of similar resolution.

The Analysed column shows the number of residues for which the backbone conformation was analysed, and the total number of residues.

Mol	Chain	Analysed	Favoured	Allowed	Outliers	Percentiles	
1	A	820/889 (92%)	738 (90%)	62 (8%)	20 (2%)	6	29

All (20) Ramachandran outliers are listed below:

Mol	Chain	Res	Type
1	A	292	ARG
1	A	371	PRO
1	A	452	ILE
1	A	554	VAL
1	A	557	ARG
1	A	851	ASP
1	A	852	GLN
1	A	42	GLU
1	A	180	LYS
1	A	556	GLY
1	A	855	GLU
1	A	222	GLU
1	A	377	TRP
1	A	454	LYS
1	A	44	TYR
1	A	638	ALA

Continued on next page...

Continued from previous page...

Mol	Chain	Res	Type
1	A	713	LYS
1	A	355	ILE
1	A	631	LYS
1	A	363	LYS

5.3.2 Protein sidechains (i)

In the following table, the Percentiles column shows the percent sidechain outliers of the chain as a percentile score with respect to all X-ray entries followed by that with respect to entries of similar resolution.

The Analysed column shows the number of residues for which the sidechain conformation was analysed, and the total number of residues.

Mol	Chain	Analysed	Rotameric	Outliers	Percentiles
1	A	693/735 (94%)	686 (99%)	7 (1%)	76 91

All (7) residues with a non-rotameric sidechain are listed below:

Mol	Chain	Res	Type
1	A	44	TYR
1	A	133	THR
1	A	419	ASN
1	A	452	ILE
1	A	735	VAL
1	A	828	VAL
1	A	858	LEU

Sometimes sidechains can be flipped to improve hydrogen bonding and reduce clashes. All (15) such sidechains are listed below:

Mol	Chain	Res	Type
1	A	22	ASN
1	A	86	ASN
1	A	269	GLN
1	A	321	ASN
1	A	324	GLN
1	A	410	ASN
1	A	419	ASN
1	A	522	GLN
1	A	697	ASN
1	A	774	GLN

Continued on next page...

Continued from previous page...

Mol	Chain	Res	Type
1	A	781	ASN
1	A	786	GLN
1	A	811	HIS
1	A	823	ASN
1	A	857	GLN

5.3.3 RNA (i)

Mol	Chain	Analysed	Backbone Outliers	Pucker Outliers
2	R	6/7 (85%)	0	0

There are no RNA backbone outliers to report.

There are no RNA pucker outliers to report.

5.4 Non-standard residues in protein, DNA, RNA chains (i)

There are no non-standard protein/DNA/RNA residues in this entry.

5.5 Carbohydrates (i)

There are no monosaccharides in this entry.

5.6 Ligand geometry (i)

There are no ligands in this entry.

5.7 Other polymers (i)

There are no such residues in this entry.

5.8 Polymer linkage issues (i)

There are no chain breaks in this entry.

6 Fit of model and data (i)

6.1 Protein, DNA and RNA chains (i)

In the following table, the column labelled '#RSRZ> 2' contains the number (and percentage) of RSRZ outliers, followed by percent RSRZ outliers for the chain as percentile scores relative to all X-ray entries and entries of similar resolution. The OWAB column contains the minimum, median, 95th percentile and maximum values of the occupancy-weighted average B-factor per residue. The column labelled 'Q< 0.9' lists the number of (and percentage) of residues with an average occupancy less than 0.9.

Mol	Chain	Analysed	<RSRZ>	#RSRZ>2			OWAB(Å^2)	Q<0.9
1	A	832/889 (93%)	2.08	339 (40%)	0	0	30, 100, 101, 102	0
2	R	7/7 (100%)	5.46	7 (100%)	0	0	99, 100, 100, 101	0
3	T	31/33 (93%)	3.79	30 (96%)	0	0	97, 100, 101, 102	0
4	N	28/33 (84%)	4.28	27 (96%)	0	0	99, 100, 101, 101	0
All	All	898/962 (93%)	2.24	403 (44%)	0	0	30, 100, 101, 102	0

All (403) RSRZ outliers are listed below:

Mol	Chain	Res	Type	RSRZ
2	R	1	G	13.1
1	A	131	ASN	8.5
4	N	120	DT	8.4
1	A	598	THR	8.0
1	A	646	PHE	7.8
1	A	716	GLY	7.8
1	A	357	ARG	7.7
1	A	527	SER	7.7
1	A	374	LEU	7.7
4	N	128	DA	7.3
1	A	223	SER	7.2
1	A	385	TYR	7.2
1	A	224	THR	6.9
1	A	128	SER	6.9
1	A	375	THR	6.8
1	A	714	LYS	6.8
4	N	121	DC	6.7
1	A	241	SER	6.7
1	A	715	THR	6.7
1	A	369	MET	6.6
1	A	248	PRO	6.6

Continued on next page...

Continued from previous page...

Mol	Chain	Res	Type	RSRZ
1	A	597	VAL	6.5
3	T	5	DG	6.5
1	A	222	GLU	6.4
1	A	671	ASN	6.2
1	A	325	ASN	6.2
1	A	230	HIS	6.2
1	A	54	MET	6.2
1	A	130	ASP	6.1
1	A	479	ILE	5.9
1	A	638	ALA	5.9
1	A	10	ASP	5.8
4	N	125	DC	5.8
2	R	7	G	5.8
3	T	3	DC	5.8
4	N	130	DG	5.8
1	A	265	SER	5.8
1	A	523	HIS	5.8
3	T	9	DG	5.6
1	A	596	THR	5.5
1	A	353	PRO	5.5
1	A	591	ASP	5.5
1	A	526	LEU	5.5
4	N	126	DG	5.2
3	T	33	DA	5.2
1	A	376	ALA	5.2
4	N	129	DC	5.2
1	A	489	ILE	5.2
1	A	358	GLU	5.2
1	A	364	PRO	5.1
4	N	127	DC	5.0
3	T	19	DT	5.0
3	T	2	DG	5.0
1	A	98	LYS	5.0
1	A	99	ARG	4.9
1	A	605	ILE	4.9
3	T	1	DA	4.8
3	T	20	DA	4.8
2	R	3	G	4.8
1	A	252	GLU	4.8
1	A	299	THR	4.8
1	A	518	TYR	4.8
1	A	346	HIS	4.8

Continued on next page...

Continued from previous page...

Mol	Chain	Res	Type	RSRZ
3	T	29	DT	4.7
4	N	119	DT	4.7
1	A	382	ALA	4.7
1	A	879	ASP	4.7
1	A	312	TYR	4.7
1	A	393	SER	4.7
1	A	311	VAL	4.7
1	A	568	GLN	4.7
4	N	123	DG	4.6
1	A	354	ALA	4.5
1	A	78	LEU	4.5
4	N	122	DT	4.5
4	N	101	DT	4.5
1	A	710	VAL	4.5
2	R	6	U	4.4
1	A	181	ALA	4.4
4	N	104	DT	4.4
4	N	131	DG	4.4
1	A	506	ASP	4.4
1	A	348	PRO	4.3
4	N	111	DC	4.3
1	A	381	ALA	4.3
2	R	2	G	4.3
1	A	370	ASN	4.3
1	A	573	ILE	4.3
1	A	488	ASN	4.2
1	A	41	HIS	4.2
1	A	355	ILE	4.2
1	A	127	THR	4.2
1	A	623	TYR	4.2
1	A	582	LEU	4.2
3	T	15	DC	4.2
1	A	764	ASN	4.2
4	N	132	DC	4.1
1	A	878	SER	4.1
3	T	6	DT	4.1
1	A	473	VAL	4.1
1	A	165	ASN	4.1
1	A	588	ASN	4.1
1	A	253	ALA	4.0
1	A	390	ALA	4.0
3	T	11	DA	4.0

Continued on next page...

Continued from previous page...

Mol	Chain	Res	Type	RSRZ
1	A	239	GLN	4.0
1	A	371	PRO	4.0
3	T	26	DT	4.0
1	A	52	ARG	4.0
1	A	615	ALA	3.9
1	A	574	VAL	3.9
1	A	851	ASP	3.9
1	A	449	GLY	3.9
1	A	667	PHE	3.9
1	A	593	GLU	3.9
1	A	762	ASN	3.9
1	A	797	TRP	3.8
1	A	344	TRP	3.8
1	A	566	THR	3.8
4	N	124	DG	3.8
1	A	639	TYR	3.8
1	A	132	THR	3.8
1	A	711	LYS	3.7
1	A	482	ILE	3.7
1	A	249	GLU	3.7
1	A	567	VAL	3.7
1	A	373	ALA	3.7
3	T	4	DC	3.7
1	A	560	ASN	3.7
3	T	8	DC	3.7
1	A	345	LYS	3.7
1	A	608	LYS	3.7
1	A	522	GLN	3.6
1	A	869	ASN	3.6
1	A	360	LEU	3.6
1	A	555	GLY	3.6
1	A	76	THR	3.6
1	A	499	ASN	3.6
4	N	112	DA	3.5
1	A	721	LYS	3.5
1	A	459	TRP	3.5
1	A	589	GLY	3.5
1	A	476	PRO	3.5
1	A	388	ASP	3.5
1	A	279	THR	3.5
4	N	102	DA	3.5
1	A	343	LYS	3.5

Continued on next page...

Continued from previous page...

Mol	Chain	Res	Type	RSRZ
1	A	511	PHE	3.5
4	N	103	DA	3.5
1	A	554	VAL	3.5
1	A	389	LYS	3.5
1	A	564	SER	3.4
1	A	513	ALA	3.4
1	A	865	PRO	3.4
1	A	519	ALA	3.4
1	A	837	GLU	3.4
1	A	380	ALA	3.4
1	A	447	ALA	3.4
1	A	465	ALA	3.4
1	A	719	LEU	3.4
3	T	22	DT	3.4
1	A	835	THR	3.4
4	N	105	DA	3.4
1	A	81	MET	3.4
1	A	565	GLU	3.3
1	A	487	GLU	3.3
3	T	13	DT	3.3
3	T	21	DG	3.3
3	T	28	DG	3.3
1	A	347	CYS	3.3
1	A	502	TRP	3.3
1	A	644	PHE	3.3
3	T	23	DG	3.3
1	A	803	GLY	3.3
1	A	720	ARG	3.3
3	T	24	DA	3.3
1	A	492	CYS	3.3
1	A	139	SER	3.2
1	A	883	ALA	3.2
1	A	90	GLU	3.2
1	A	818	PRO	3.2
1	A	668	THR	3.2
1	A	517	GLU	3.2
1	A	575	ALA	3.2
1	A	744	GLN	3.2
1	A	361	PRO	3.2
1	A	813	SER	3.2
1	A	468	ALA	3.2
1	A	859	ASP	3.2

Continued on next page...

Continued from previous page...

Mol	Chain	Res	Type	RSRZ
1	A	647	ARG	3.2
4	N	133	DT	3.2
1	A	505	GLN	3.1
1	A	578	VAL	3.1
2	R	5	G	3.1
1	A	607	GLU	3.1
1	A	654	THR	3.1
1	A	251	ALA	3.1
1	A	819	ALA	3.1
1	A	453	GLY	3.1
1	A	552	ASP	3.1
1	A	570	ILE	3.1
1	A	362	MET	3.1
1	A	769	ILE	3.1
1	A	661	SER	3.0
1	A	779	ALA	3.0
1	A	79	PRO	3.0
1	A	232	GLN	3.0
1	A	480	LYS	3.0
1	A	765	LYS	3.0
3	T	7	DG	3.0
1	A	28	TYR	3.0
1	A	448	LYS	3.0
3	T	32	DT	3.0
1	A	846	TYR	3.0
1	A	157	LEU	3.0
1	A	616	LEU	3.0
1	A	756	ARG	3.0
1	A	451	PRO	3.0
3	T	10	DC	3.0
1	A	583	GLN	3.0
1	A	766	ASP	3.0
1	A	180	LYS	2.9
1	A	590	THR	2.9
1	A	406	ASN	2.9
1	A	51	PHE	2.9
1	A	563	PRO	2.9
1	A	824	LEU	2.9
1	A	660	ASP	2.9
1	A	396	ILE	2.9
1	A	236	VAL	2.9
3	T	30	DA	2.9

Continued on next page...

Continued from previous page...

Mol	Chain	Res	Type	RSRZ
1	A	466	ASN	2.9
1	A	326	THR	2.9
1	A	562	LEU	2.9
1	A	189	ASP	2.9
1	A	738	GLU	2.9
1	A	197	GLY	2.9
3	T	16	DC	2.9
3	T	25	DG	2.9
1	A	539	SER	2.9
1	A	94	ALA	2.9
1	A	771	ALA	2.9
1	A	768	GLU	2.9
1	A	472	LYS	2.8
1	A	561	LEU	2.8
1	A	240	ASP	2.8
1	A	640	GLY	2.8
1	A	717	GLU	2.8
1	A	543	ILE	2.8
1	A	770	ASP	2.8
1	A	752	LEU	2.8
1	A	498	GLU	2.8
1	A	352	ILE	2.8
1	A	778	ILE	2.7
1	A	57	ARG	2.7
1	A	551	ARG	2.7
1	A	379	ARG	2.7
1	A	676	TYR	2.7
1	A	725	VAL	2.7
1	A	104	GLN	2.7
1	A	718	ILE	2.7
1	A	630	THR	2.7
1	A	556	GLY	2.7
1	A	491	ALA	2.7
2	R	4	A	2.7
1	A	190	MET	2.7
1	A	49	ALA	2.7
1	A	724	ALA	2.7
1	A	635	MET	2.7
1	A	277	PRO	2.7
1	A	780	PRO	2.7
1	A	823	ASN	2.7
1	A	484	GLU	2.6

Continued on next page...

Continued from previous page...

Mol	Chain	Res	Type	RSRZ
1	A	247	ALA	2.6
1	A	202	SER	2.6
1	A	359	GLU	2.6
1	A	531	SER	2.6
4	N	113	DC	2.6
1	A	433	ASN	2.6
1	A	742	PRO	2.6
1	A	278	TRP	2.6
1	A	755	PHE	2.6
1	A	613	THR	2.6
1	A	342	THR	2.6
1	A	627	ARG	2.6
1	A	88	TRP	2.6
1	A	416	PHE	2.5
1	A	93	LYS	2.5
1	A	828	VAL	2.5
1	A	141	ILE	2.5
1	A	43	SER	2.5
1	A	483	GLU	2.5
1	A	100	PRO	2.5
1	A	275	PRO	2.5
1	A	368	ASP	2.5
4	N	108	DA	2.5
1	A	413	ALA	2.5
1	A	397	SER	2.5
1	A	486	HIS	2.5
1	A	97	GLY	2.5
1	A	739	TYR	2.5
1	A	75	THR	2.5
1	A	83	ALA	2.5
1	A	145	ILE	2.5
1	A	77	LEU	2.4
1	A	327	ALA	2.4
1	A	50	ARG	2.4
1	A	184	GLN	2.4
1	A	785	SER	2.4
4	N	107	DG	2.4
1	A	882	PHE	2.4
1	A	553	GLU	2.4
1	A	349	VAL	2.4
1	A	507	SER	2.4
1	A	525	GLY	2.4

Continued on next page...

Continued from previous page...

Mol	Chain	Res	Type	RSRZ
3	T	31	DT	2.4
1	A	85	ILE	2.4
1	A	620	TRP	2.4
3	T	27	DC	2.4
1	A	763	THR	2.4
1	A	836	TYR	2.4
1	A	633	SER	2.4
1	A	861	MET	2.4
1	A	781	ASN	2.4
1	A	109	ILE	2.4
1	A	464	GLY	2.4
1	A	786	GLN	2.4
1	A	880	PHE	2.4
1	A	30	GLU	2.4
1	A	166	VAL	2.4
1	A	300	HIS	2.4
1	A	298	ARG	2.3
1	A	378	LYS	2.3
1	A	577	LYS	2.3
1	A	73	LEU	2.3
1	A	363	LYS	2.3
1	A	866	ALA	2.3
1	A	331	ASN	2.3
1	A	287	TRP	2.3
1	A	537	ASP	2.3
1	A	264	ILE	2.3
1	A	383	ALA	2.3
1	A	873	ARG	2.3
1	A	9	ASN	2.3
1	A	713	LYS	2.3
1	A	541	SER	2.3
1	A	455	GLU	2.3
1	A	571	TYR	2.3
1	A	242	GLU	2.3
1	A	670	PRO	2.3
1	A	581	ILE	2.3
1	A	478	ARG	2.3
1	A	187	GLU	2.3
4	N	109	DC	2.3
1	A	821	ALA	2.3
1	A	23	THR	2.3
1	A	703	ALA	2.3

Continued on next page...

Continued from previous page...

Mol	Chain	Res	Type	RSRZ
1	A	46	MET	2.2
1	A	412	LYS	2.2
1	A	87	ASP	2.2
1	A	875	ILE	2.2
4	N	110	DT	2.2
1	A	533	PRO	2.2
1	A	579	ASN	2.2
1	A	870	LEU	2.2
1	A	310	ASP	2.2
1	A	862	PRO	2.2
1	A	584	ALA	2.2
1	A	650	VAL	2.2
1	A	286	TYR	2.2
1	A	391	ARG	2.2
1	A	847	ASP	2.2
1	A	254	ILE	2.1
1	A	643	GLU	2.1
1	A	839	CYS	2.1
1	A	129	ALA	2.1
1	A	595	VAL	2.1
1	A	795	VAL	2.1
1	A	458	TYR	2.1
1	A	351	ASP	2.1
1	A	648	GLN	2.1
1	A	674	ALA	2.1
1	A	816	THR	2.1
1	A	672	GLN	2.1
1	A	542	GLY	2.1
1	A	860	LYS	2.1
1	A	108	GLU	2.1
1	A	411	HIS	2.1
1	A	500	THR	2.1
1	A	774	GLN	2.1
1	A	637	LEU	2.1
1	A	45	GLU	2.1
1	A	426	VAL	2.1
1	A	274	PRO	2.1
1	A	524	HIS	2.1
1	A	529	ASN	2.0
1	A	55	PHE	2.0
1	A	772	HIS	2.0
1	A	881	ALA	2.0

Continued on next page...

Continued from previous page...

Mol	Chain	Res	Type	RSRZ
1	A	494	LYS	2.0
1	A	820	ASP	2.0
1	A	53	LYS	2.0
3	T	12	DC	2.0

6.2 Non-standard residues in protein, DNA, RNA chains (i)

There are no non-standard protein/DNA/RNA residues in this entry.

6.3 Carbohydrates (i)

There are no monosaccharides in this entry.

6.4 Ligands (i)

There are no ligands in this entry.

6.5 Other polymers (i)

There are no such residues in this entry.
