# Supplementary material for: An engineered T7 RNA polymerase that produces mRNA free of immunostimulatory byproducts
Source: Nat Biotechnol. 2022 Nov 10;41(4):560–8. doi: 10.1038/s41587-022-01525-6 (PMC10110463; doi:10.1038/s41587-022-01525-6)
Supplement: Supplementary file 9 — Full validation report for PDB entry 1S76. [file 41587_2022_1525_MOESM9_ESM.pdf]

Full wwPDB X-ray Structure Validation Report (i)

May 13, 2020 – 08:30 pm BST

PDB ID	:	1S76
Title	:	T7 RNA polymerase alpha beta methylene ATP elongation complex
Authors	:	Yin, Y.W.; Steitz, T.A.
Deposited on	:	2004-01-29
Resolution	:	2.88 Å(reported)

This is a Full wwPDB X-ray Structure Validation Report for a publicly released PDB entry.

We welcome your comments at *validation@mail.wwpdb.org*
A user guide is available at
https://www.wwpdb.org/validation/2017/XrayValidationReportHelp
with specific help available everywhere you see the (i) symbol.

The following versions of software and data (see references (i)) were used in the production of this report:

MolProbity	:	4.02b-467
Mogul	:	1.8.5 (274361), CSD as541be (2020)
Xtriage (Phenix)	:	1.13
EDS	:	2.11
buster-report	:	1.1.7 (2018)
Percentile statistics	:	20191225.v01 (using entries in the PDB archive December 25th 2019)
Refmac	:	5.8.0158
CCP4	:	7.0.044 (Gargrove)
Ideal geometry (proteins)	:	Engh & Huber (2001)
Ideal geometry (DNA, RNA)	:	Parkinson et al. (1996)
Validation Pipeline (wwPDB-VP)	:	2.11

1 Overall quality at a glance (i)

The following experimental techniques were used to determine the structure:
X-RAY DIFFRACTION

The reported resolution of this entry is 2.88 Å.

Percentile scores (ranging between 0-100) for global validation metrics of the entry are shown in the following graphic. The table shows the number of entries on which the scores are based.

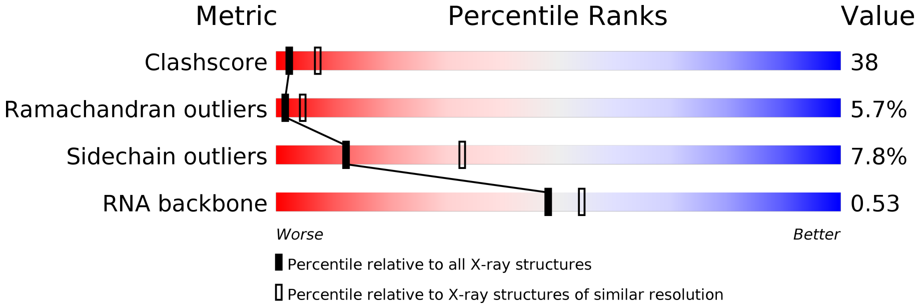

Metric	Whole archive (#Entries)	Similar resolution (#Entries, resolution range(Å))
Clashscore	141614	2947 (2.90-2.86)
Ramachandran outliers	138981	2868 (2.90-2.86)
Sidechain outliers	138945	2871 (2.90-2.86)
RNA backbone	3102	1121 (3.16-2.60)

The table below summarises the geometric issues observed across the polymeric chains and their fit to the electron density. The red, orange, yellow and green segments on the lower bar indicate the fraction of residues that contain outliers for >=3, 2, 1 and 0 types of geometric quality criteria respectively. A grey segment represents the fraction of residues that are not modelled. The numeric value for each fraction is indicated below the corresponding segment, with a dot representing fractions <=5%

Mol	Chain	Length	Quality of chain
1	T	21	10% 62% 29%
2	N	17	12% 88%
3	R	9	22% 78%
4	D	883	42% 43% 9% 6%

The following table lists non-polymeric compounds, carbohydrate monomers and non-standard residues in protein, DNA, RNA chains that are outliers for geometric or electron-density-fit criteria:

Mol	Type	Chain	Res	Chirality	Geometry	Clashes	Electron density
5	APC	R	901	-	-	X	-

2 Entry composition (i)

There are 6 unique types of molecules in this entry. The entry contains 7561 atoms, of which 0 are hydrogens and 0 are deuteriums.

In the tables below, the ZeroOcc column contains the number of atoms modelled with zero occupancy, the AltConf column contains the number of residues with at least one atom in alternate conformation and the Trace column contains the number of residues modelled with at most 2 atoms.

- Molecule 1 is a DNA chain called DNA (5'-D(P*GP*CP*CP*GP*TP*GP*CP*GP*CP*AP *TP*TP*CP*GP*CP*CP*GP*TP*GP*TP*T)-3').

Mol	Chain	Residues	Atoms					ZeroOcc	AltConf	Trace
			Total	C	N	O	P			
1	T	21	429	203	73	132	21	0	0	0

- Molecule 2 is a DNA chain called DNA (5'-D(P*TP*TP*TP*AP*CP*GP*TP*TP*GP*CP *GP*CP*AP*CP*GP*GP*C)-3').

Mol	Chain	Residues	Atoms					ZeroOcc	AltConf	Trace
			Total	C	N	O	P			
2	N	17	348	165	60	106	17	0	0	0

- Molecule 3 is a RNA chain called RNA (5'-R(P*AP*CP*AP*CP*GP*GP*CP*GP*A)-3').

Mol	Chain	Residues	Atoms					ZeroOcc	AltConf	Trace
			Total	C	N	O	P			
3	R	9	196	87	39	61	9	0	0	0

- Molecule 4 is a protein called DNA-directed RNA polymerase.

Mol	Chain	Residues	Atoms					ZeroOcc	AltConf	Trace
			Total	C	N	O	S			
4	D	829	6555	4178	1143	1198	36	0	0	0

- Molecule 5 is DIPHOSPHOMETHYLPHOSPHONIC ACID ADENOSYL ESTER (three-letter code: APC) (formula: $C_{11}H_{18}N_5O_{12}P_3$).

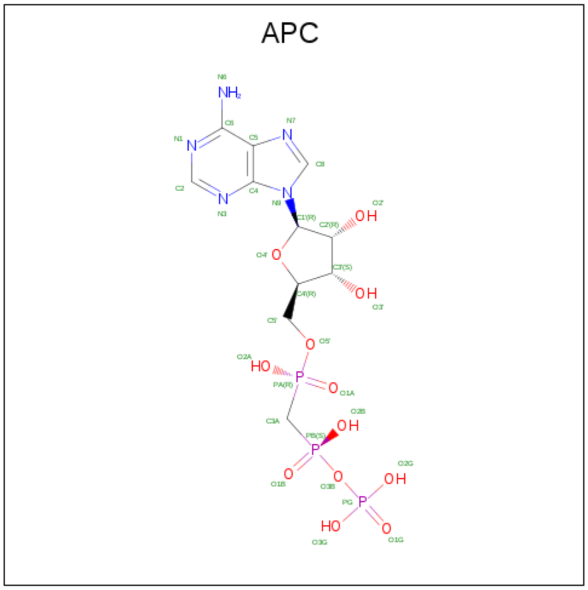

Mol	Chain	Residues	Atoms					ZeroOcc	AltConf
			Total	C	N	O	P		
5	R	1	31	11	5	12	3	0	0

- Molecule 6 is MAGNESIUM ION (three-letter code: MG) (formula: Mg).

Mol	Chain	Residues	Atoms		ZeroOcc	AltConf
			Total	Mg		
6	D	2	2	2	0	0

3 Residue-property plots ⓘ

These plots are drawn for all protein, RNA and DNA chains in the entry. The first graphic for a chain summarises the proportions of the various outlier classes displayed in the second graphic. The second graphic shows the sequence view annotated by issues in geometry. Residues are color-coded according to the number of geometric quality criteria for which they contain at least one outlier: green = 0, yellow = 1, orange = 2 and red = 3 or more. Stretches of 2 or more consecutive residues without any outlier are shown as a green connector. Residues present in the sample, but not in the model, are shown in grey.

• Molecule 1: DNA (5'-D(P*GP*CP*CP*GP*TP*GP*CP*GP*CP*AP*TP*TP*CP*GP*CP*CP*GP*TP*GP*TP*T)-3')

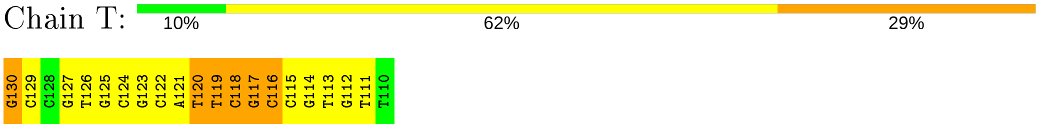

• Molecule 2: DNA (5'-D(P*TP*TP*TP*AP*CP*GP*TP*TP*GP*CP*GP*CP*AP*CP*GP*GP*C)-3')

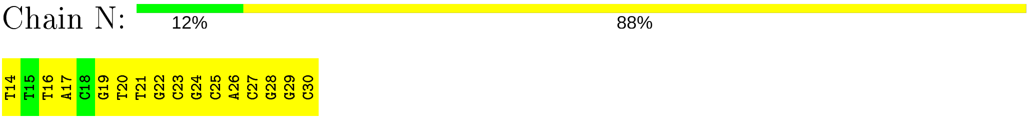

• Molecule 3: RNA (5'-R(P*AP*CP*AP*CP*GP*GP*CP*GP*A)-3')

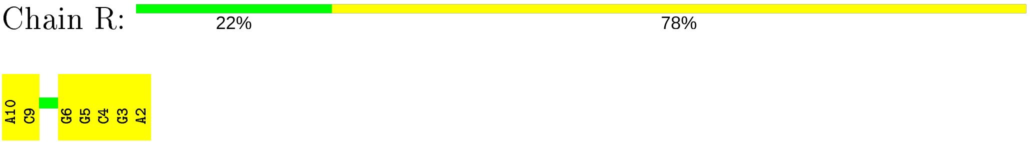

• Molecule 4: DNA-directed RNA polymerase

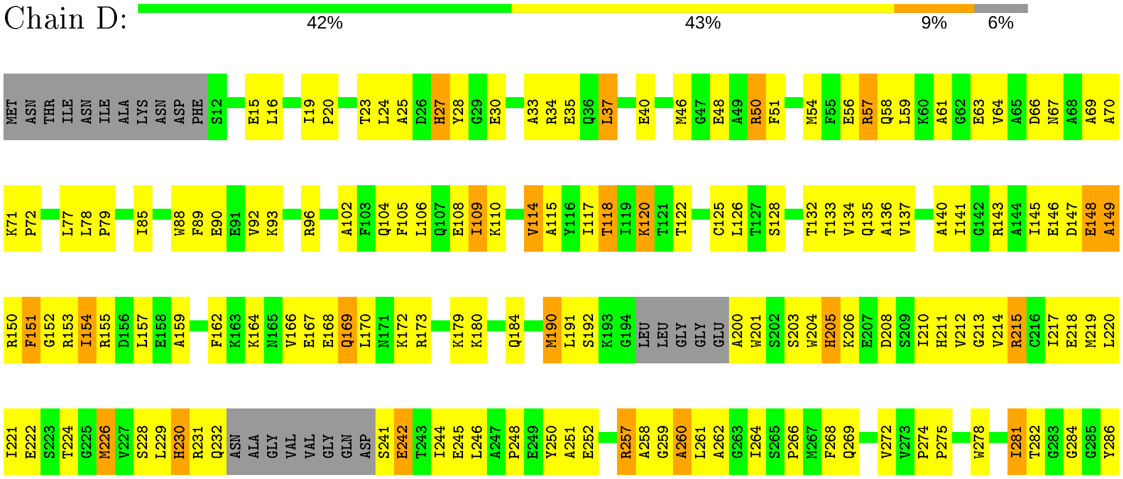

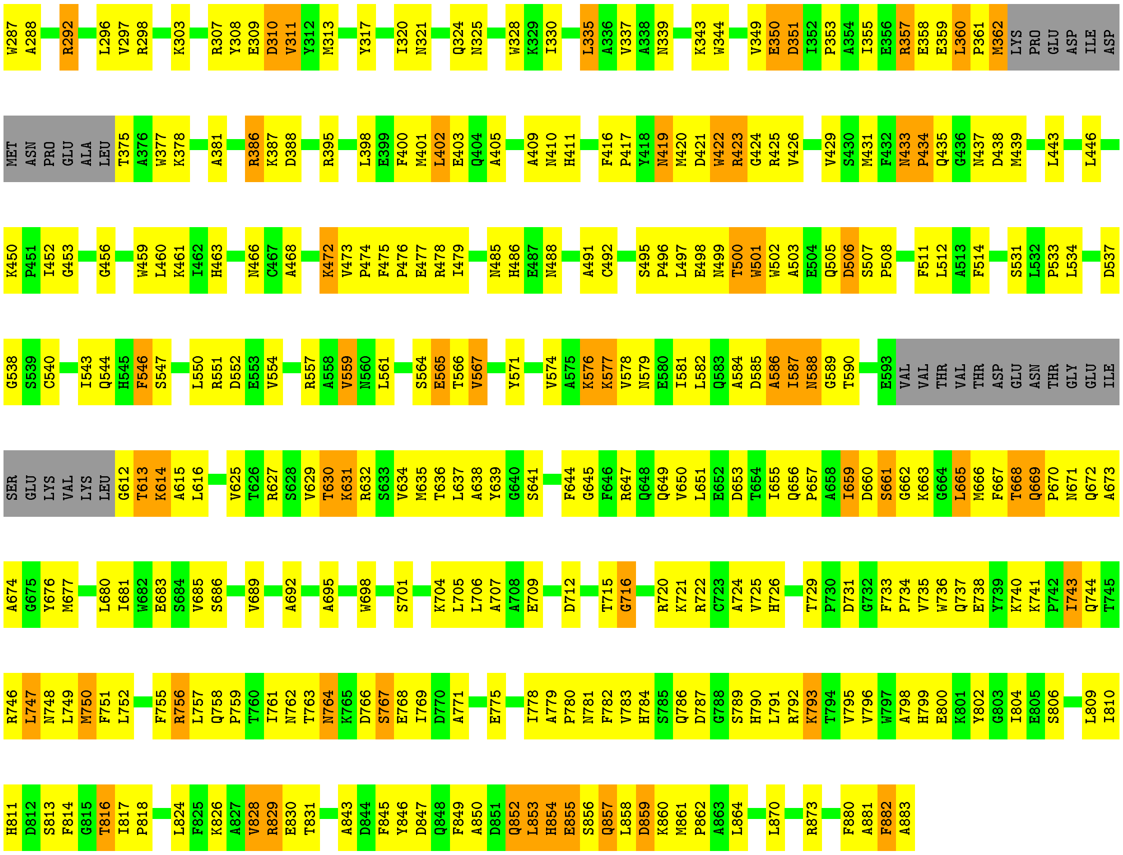
W287 A288 R292 L296 V297 R298 K303 R307 Y308 E309 D310 V311 Y312 M313 Y317 I320 N321 Q324 N325 W328 K329 I330 L335 A336 V337 A338 N339 K343 W344 V349 E350 D351 I352 P353 A354 I355 E356 R357 E358 E359 L360 P361 M362 LYS PRO GLU ASP ILE ASP
MET ASN PRO GLU ALA LEU T375 A376 W377 K378 A381 R386 K387 D388 R395 L398 E399 F400 M401 L402 E403 Q404 A405 A409 N410 H411 F416 P417 Y418 N419 M420 D421 W422 R423 G424 R425 V426 V429 S430 M431 F432 N433 P434 Q435 G436 N437 D438 M439 L443 L446
K450 P451 I452 G453 G456 W459 L460 K461 I462 H463 N466 C467 A468 K472 V473 P474 F475 P476 E477 R478 I479 N485 H486 E487 N488 A491 C492 S495 P496 L497 E498 N499 T500 W501 W502 A503 E504 Q505 D506 S507 P508 F511 L512 A513 F514 S531 L532 P533 L534 D537
G538 S539 C540 I543 Q544 H545 F546 S547 L550 R551 D552 E553 V554 R557 A558 V559 N560 L561 S564 E565 T566 V567 Y571 V574 A575 K576 K577 V578 N579 E580 I581 L582 Q583 A584 D585 A586 I587 N588 G589 T590 E593 VAL VAL THR VAL THR ASP GLU ASN THR GLY GLU ILE
SER GLU LYS VAL LYS LEU G612 T613 K614 A615 L616 V625 T626 R627 S628 V629 T630 K631 R632 S633 V634 M635 T636 L637 A638 Y639 G640 S641 F644 G645 F646 R647 Q648 Q649 V650 L651 E652 D653 T654 I655 Q656 P657 A658 I659 D660 S661 G662 K663 G664 L665 M666 F667 T668 Q669 P670 N671 Q672 A673
A674 G675 Y676 M677 L680 I681 W682 E683 S684 V685 S686 V689 A692 A695 W698 S701 K704 L705 L706 A707 A708 E709 D712 T715 G716 R720 K721 R722 C723 A724 V725 H726 T729 P730 D731 G732 F733 P734 V735 W736 Q737 E738 Y739 K740 K741 P742 I743 Q744 T745
R746 L747 N748 L749 M750 F751 L752 F755 R756 L757 Q758 P759 T760 I761 N762 T763 N764 K765 D766 S767 E768 I769 D770 A771 E775 I778 A779 P780 N781 F782 V783 H784 S785 Q786 D787 G788 S789 H790 L791 R792 K793 T794 V795 V796 W797 A798 H799 E800 K801 Y802 G803 I804 E805 S806 L809 I810
H811 D812 S813 F814 G815 T816 I817 P818 L824 F825 K826 A827 V828 R829 E830 T831 A843 D844 F845 Y846 D847 Q848 F849 A850 D851 Q852 L853 H854 E855 S856 Q857 L858 D859 K860 M861 P862 A863 L864 L870 R873 F880 A881 F882 A883

4 Data and refinement statistics (i)

Property	Value	Source
Space group	C 2 2 21	Depositor
Cell constants<br>a, b, c, α, β, γ	138.72Å 143.32Å 146.65Å<br>90.00° 90.00° 90.00°	Depositor
Resolution (Å)	30.00 – 2.88<br>29.53 – 2.88	Depositor<br>EDS
% Data completeness<br>(in resolution range)	99.8 (30.00-2.88)<br>91.2 (29.53-2.88)	Depositor<br>EDS
R_{merge}	(Not available)	Depositor
R_{sym}	(Not available)	Depositor
$< I/\sigma(I) >$ [1]	14.73 (at 2.90Å)	Xtriage
Refinement program	CNS 1.1	Depositor
R, R_{free}	0.248 , 0.290<br>0.211 , (Not available)	Depositor<br>DCC
R_{free} test set	No test flags present.	wwPDB-VP
Wilson B-factor (Å^2)	38.1	Xtriage
Anisotropy	0.090	Xtriage
Bulk solvent k_{sol}(e/Å^3), B_{sol}(Å^2)	0.06 , 458.1	EDS
L-test for twinning[2]	$< \|L\| >$ = 0.29, $< L^2 >$ = 0.13	Xtriage
Estimated twinning fraction	0.227 for -k,-h,-l	Xtriage
F_o,F_c correlation	0.90	EDS
Total number of atoms	7561	wwPDB-VP
Average B, all atoms (Å^2)	41.0	wwPDB-VP

Xtriage's analysis on translational NCS is as follows: *The largest off-origin peak in the Patterson function is 1.92% of the height of the origin peak. No significant pseudotranslation is detected.*

[1] Intensities estimated from amplitudes.

[2] Theoretical values of $< |L| >$, $< L^2 >$ for acentric reflections are 0.5, 0.333 respectively for untwinned datasets, and 0.375, 0.2 for perfectly twinned datasets.

5 Model quality (i)

5.1 Standard geometry (i)

Bond lengths and bond angles in the following residue types are not validated in this section: APC, MG

The Z score for a bond length (or angle) is the number of standard deviations the observed value is removed from the expected value. A bond length (or angle) with $|Z| > 5$ is considered an outlier worth inspection. RMSZ is the root-mean-square of all Z scores of the bond lengths (or angles).

Mol	Chain	Bond lengths		Bond angles	
		RMSZ	#$\|Z\|$ >5	RMSZ	#$\|Z\|$ >5
1	T	0.57	1/478 (0.2%)	0.85	0/734
2	N	0.50	1/388 (0.3%)	0.69	0/595
3	R	0.72	1/219 (0.5%)	0.83	0/338
4	D	0.36	2/6706 (0.0%)	0.58	1/9068 (0.0%)
All	All	0.40	5/7791 (0.1%)	0.62	1/10735 (0.0%)

Chiral center outliers are detected by calculating the chiral volume of a chiral center and verifying if the center is modelled as a planar moiety or with the opposite hand.A planarity outlier is detected by checking planarity of atoms in a peptide group, atoms in a mainchain group or atoms of a sidechain that are expected to be planar.

Mol	Chain	#Chirality outliers	#Planarity outliers
1	T	0	5

All (5) bond length outliers are listed below:

Mol	Chain	Res	Type	Atoms	Z	Observed(Å)	Ideal(Å)
2	N	14	DT	OP3-P	-7.30	1.52	1.61
3	R	10	A	OP3-P	-7.28	1.52	1.61
1	T	130	DG	OP3-P	-7.24	1.52	1.61
4	D	292	ARG	C-N	-6.38	1.22	1.34
4	D	251	ALA	C-N	5.80	1.47	1.34

All (1) bond angle outliers are listed below:

Mol	Chain	Res	Type	Atoms	Z	Observed(^{o})	Ideal(^{o})
4	D	828	VAL	N-CA-C	-5.01	97.48	111.00

There are no chirality outliers.

All (5) planarity outliers are listed below:

Mol	Chain	Res	Type	Group
1	T	116	DC	Sidechain
1	T	117	DG	Sidechain
1	T	118	DC	Sidechain
1	T	119	DT	Sidechain
1	T	120	DT	Sidechain

5.2 Too-close contacts (i)

In the following table, the Non-H and H(model) columns list the number of non-hydrogen atoms and hydrogen atoms in the chain respectively. The H(added) column lists the number of hydrogen atoms added and optimized by MolProbity. The Clashes column lists the number of clashes within the asymmetric unit, whereas Symm-Clashes lists symmetry related clashes.

Mol	Chain	Non-H	H(model)	H(added)	Clashes	Symm-Clashes
1	T	429	0	238	28	0
2	N	348	0	193	26	0
3	R	196	0	100	15	0
4	D	6555	0	6518	509	0
5	R	31	0	14	11	0
6	D	2	0	0	0	0
All	All	7561	0	7063	561	0

The all-atom clashscore is defined as the number of clashes found per 1000 atoms (including hydrogen atoms). The all-atom clashscore for this structure is 38.

All (561) close contacts within the same asymmetric unit are listed below, sorted by their clash magnitude.

Atom-1	Atom-2	Interatomic distance (Å)	Clash overlap (Å)
3:R:2:A:O3'	5:R:901:APC:H5'1	1.34	1.28
4:D:759:PRO:HG2	4:D:764:ASN:ND2	1.61	1.15
4:D:724:ALA:HB2	4:D:738:GLU:HB3	1.36	1.06
4:D:763:THR:O	4:D:764:ASN:CG	1.96	1.04
3:R:2:A:O3'	5:R:901:APC:C5'	2.09	1.01
4:D:861:MET:HE3	4:D:862:PRO:HD2	1.44	1.00
4:D:417:PRO:HG2	4:D:429:VAL:HB	1.48	0.96
4:D:278:TRP:H	4:D:321:ASN:HD21	0.98	0.95
4:D:798:ALA:HB1	4:D:804:ILE:HD12	1.49	0.94
4:D:360:LEU:H	4:D:361:PRO:HD2	1.30	0.94
4:D:109:ILE:HG23	4:D:150:ARG:H	1.31	0.93
2:N:16:DT:O3'	4:D:378:LYS:HD3	1.69	0.93
4:D:349:VAL:HG22	4:D:503:ALA:HB1	1.51	0.92

Continued on next page...

Continued from previous page...

Atom-1	Atom-2	Interatomic distance (Å)	Clash overlap (Å)
2:N:27:DC:H1'	2:N:28:DG:H5"	1.52	0.91
4:D:763:THR:O	4:D:764:ASN:OD1	1.88	0.90
4:D:162:PHE:HA	4:D:166:VAL:HB	1.52	0.89
4:D:756:ARG:HE	4:D:756:ARG:H	1.20	0.88
4:D:755:PHE:HA	4:D:756:ARG:HH21	1.38	0.87
4:D:72:PRO:HG3	4:D:257:ARG:HG3	1.56	0.86
4:D:746:ARG:HG3	4:D:756:ARG:HG3	1.56	0.86
4:D:756:ARG:N	4:D:756:ARG:HE	1.74	0.85
1:T:120:DT:H2"	1:T:119:DT:OP2	1.76	0.85
4:D:450:LYS:HE2	4:D:817:ILE:HD11	1.56	0.85
4:D:856:SER:O	4:D:857:GLN:HB3	1.77	0.84
1:T:116:DC:H2"	1:T:115:DC:OP2	1.77	0.84
4:D:759:PRO:CG	4:D:764:ASN:ND2	2.38	0.84
1:T:124:DC:N4	2:N:24:DG:H1	1.76	0.83
4:D:473:VAL:HG22	4:D:474:PRO:HD2	1.61	0.83
3:R:5:G:H2'	3:R:4:C:H6	1.42	0.82
4:D:152:GLY:HA2	4:D:155:ARG:HD3	1.61	0.82
4:D:126:LEU:HA	4:D:132:THR:HG21	1.60	0.82
4:D:584:ALA:HB1	4:D:587:ILE:HG23	1.62	0.81
4:D:358:GLU:OE1	4:D:387:LYS:HD2	1.81	0.81
2:N:17:DA:P	4:D:378:LYS:HD3	2.19	0.81
4:D:461:LYS:HD2	4:D:479:ILE:HG23	1.62	0.81
4:D:724:ALA:CB	4:D:738:GLU:HB3	2.11	0.81
4:D:433:ASN:HD22	4:D:435:GLN:H	1.28	0.80
4:D:559:VAL:HG23	4:D:561:LEU:HD13	1.61	0.80
4:D:278:TRP:H	4:D:321:ASN:ND2	1.79	0.80
4:D:759:PRO:CB	4:D:764:ASN:HD21	1.96	0.79
4:D:386:ARG:HD3	4:D:387:LYS:N	1.97	0.79
4:D:873:ARG:HH11	4:D:873:ARG:HG3	1.47	0.78
1:T:124:DC:H42	2:N:24:DG:H1	1.29	0.78
4:D:120:LYS:HE2	4:D:752:LEU:HD23	1.66	0.78
4:D:651:LEU:CD1	4:D:670:PRO:HB2	2.13	0.78
4:D:67:ASN:HD22	4:D:69:ALA:H	1.30	0.78
4:D:630:THR:O	4:D:634:VAL:HG23	1.84	0.77
4:D:662:GLY:O	4:D:665:LEU:HD22	1.83	0.77
4:D:759:PRO:CG	4:D:764:ASN:HD21	1.97	0.77
4:D:213:GLY:O	4:D:217:ILE:HG12	1.85	0.76
2:N:23:DC:H2"	2:N:24:DG:H5'	1.68	0.76
4:D:826:LYS:O	4:D:830:GLU:HG3	1.85	0.75
4:D:846:TYR:HA	4:D:849:PHE:CE1	2.22	0.75
4:D:23:THR:O	4:D:27:HIS:HB2	1.86	0.75

Continued on next page...

Continued from previous page...

Atom-1	Atom-2	Interatomic distance (Å)	Clash overlap (Å)
3:R:2:A:H2'	5:R:901:APC:H8	1.67	0.75
4:D:473:VAL:CG2	4:D:474:PRO:HD2	2.17	0.74
4:D:663:LYS:H	4:D:663:LYS:HD2	1.50	0.74
4:D:705:LEU:HD22	4:D:858:LEU:HD22	1.69	0.74
2:N:20:DT:H2"	2:N:21:DT:H72	1.70	0.74
3:R:5:G:H2'	3:R:4:C:C6	2.21	0.73
4:D:550:LEU:HD11	4:D:695:ALA:HB2	1.69	0.73
4:D:154:ILE:HG22	4:D:162:PHE:HB2	1.70	0.72
4:D:109:ILE:HG21	4:D:148:GLU:HB3	1.71	0.72
4:D:298:ARG:NE	4:D:419:ASN:HD21	1.87	0.72
4:D:689:VAL:HG12	4:D:692:ALA:HB3	1.70	0.72
4:D:359:GLU:HB3	4:D:361:PRO:HD2	1.71	0.72
4:D:584:ALA:HB1	4:D:587:ILE:CG2	2.19	0.72
4:D:828:VAL:O	4:D:829:ARG:HB2	1.88	0.72
4:D:261:LEU:O	4:D:264:ILE:HG13	1.89	0.72
4:D:335:LEU:HD22	4:D:339:ASN:ND2	2.05	0.72
4:D:125:CYS:O	4:D:132:THR:HG22	1.90	0.71
4:D:651:LEU:HD11	4:D:670:PRO:HB2	1.71	0.71
4:D:631:LYS:HD2	4:D:632:ARG:N	2.05	0.71
4:D:78:LEU:HB3	4:D:79:PRO:HD3	1.73	0.71
4:D:577:LYS:HE3	4:D:577:LYS:HA	1.73	0.71
4:D:639:TYR:CE1	4:D:783:VAL:HG21	2.26	0.71
4:D:495:SER:HB3	4:D:498:GLU:HB2	1.73	0.70
4:D:456:GLY:O	4:D:459:TRP:O	2.09	0.70
4:D:655:ILE:HD12	4:D:674:ALA:HB2	1.72	0.70
4:D:766:ASP:O	4:D:767:SER:HB2	1.92	0.69
4:D:864:LEU:HD12	4:D:864:LEU:H	1.56	0.69
4:D:154:ILE:H	4:D:154:ILE:HD12	1.56	0.69
3:R:2:A:O2'	5:R:901:APC:O4'	2.10	0.69
4:D:806:SER:O	4:D:816:THR:HG23	1.92	0.69
4:D:756:ARG:H	4:D:756:ARG:NE	1.91	0.69
4:D:565:GLU:OE2	4:D:566:THR:HG22	1.93	0.68
4:D:360:LEU:H	4:D:361:PRO:CD	2.04	0.68
4:D:574:VAL:O	4:D:578:VAL:HG23	1.93	0.68
4:D:629:VAL:HG12	4:D:630:THR:H	1.59	0.68
4:D:308:TYR:HA	4:D:311:VAL:HG13	1.76	0.68
4:D:298:ARG:HE	4:D:419:ASN:HD21	1.40	0.68
4:D:466:ASN:HD21	4:D:478:ARG:HH11	1.40	0.68
4:D:85:ILE:HA	4:D:219:MET:HE2	1.76	0.68
4:D:126:LEU:HA	4:D:132:THR:CG2	2.24	0.68
4:D:360:LEU:N	4:D:361:PRO:HD2	2.07	0.68

Continued on next page...

Continued from previous page...

Atom-1	Atom-2	Interatomic distance (Å)	Clash overlap (Å)
4:D:298:ARG:HE	4:D:419:ASN:ND2	1.91	0.68
4:D:85:ILE:HA	4:D:219:MET:CE	2.23	0.68
4:D:109:ILE:HD13	4:D:114:VAL:HG22	1.76	0.68
4:D:117:ILE:HG12	4:D:752:LEU:HD22	1.75	0.68
4:D:433:ASN:ND2	4:D:435:GLN:HB2	2.09	0.68
4:D:500:THR:O	4:D:501:TRP:HB3	1.94	0.67
4:D:853:LEU:H	4:D:853:LEU:HD12	1.60	0.67
4:D:829:ARG:NH2	4:D:882:PHE:H	1.93	0.67
4:D:109:ILE:HG23	4:D:150:ARG:N	2.08	0.67
4:D:278:TRP:CD2	4:D:284:GLY:HA3	2.30	0.67
4:D:154:ILE:N	4:D:154:ILE:HD12	2.09	0.67
4:D:726:HIS:HD2	4:D:735:VAL:O	1.78	0.67
4:D:828:VAL:HG21	4:D:883:ALA:HA	1.77	0.66
4:D:219:MET:HA	4:D:222:GLU:HB2	1.77	0.66
2:N:27:DC:H2”	2:N:28:DG:C5’	2.25	0.66
4:D:88:TRP:CZ2	4:D:215:ARG:HD3	2.31	0.66
4:D:423:ARG:HB2	4:D:423:ARG:NH1	2.11	0.65
4:D:873:ARG:HG3	4:D:873:ARG:NH1	2.11	0.65
4:D:589:GLY:HA2	4:D:614:LYS:H	1.60	0.65
4:D:438:ASP:OD2	4:D:508:PRO:HG2	1.97	0.65
4:D:581:ILE:HG21	4:D:680:LEU:HD22	1.80	0.64
4:D:210:ILE:O	4:D:214:VAL:HG23	1.98	0.64
4:D:208:ASP:O	4:D:212:VAL:HG23	1.97	0.64
4:D:307:ARG:HG2	4:D:736:TRP:CZ3	2.32	0.64
4:D:281:ILE:HG12	4:D:282:THR:HG23	1.80	0.64
4:D:534:LEU:HD12	4:D:818:PRO:HA	1.78	0.64
4:D:663:LYS:HD2	4:D:663:LYS:N	2.13	0.64
4:D:767:SER:O	4:D:768:GLU:HB3	1.98	0.63
4:D:419:ASN:C	4:D:419:ASN:HD22	2.01	0.63
4:D:639:TYR:HE1	4:D:783:VAL:HG21	1.63	0.63
4:D:631:LYS:C	4:D:631:LYS:HD2	2.20	0.62
4:D:854:HIS:O	4:D:858:LEU:HB2	1.99	0.62
4:D:104:GLN:HE21	4:D:104:GLN:HA	1.63	0.62
1:T:124:DC:H1’	1:T:123:DG:H5’	1.80	0.62
4:D:46:MET:HE1	4:D:269:GLN:NE2	2.15	0.62
3:R:2:A:C3’	5:R:901:APC:H5’1	2.28	0.62
4:D:204:TRP:O	4:D:208:ASP:HB2	1.99	0.62
4:D:810:ILE:HB	4:D:813:SER:HB3	1.82	0.62
4:D:122:THR:HG22	4:D:141:ILE:HD11	1.81	0.62
1:T:117:DG:H5”	4:D:421:ASP:HB2	1.81	0.62
4:D:828:VAL:O	4:D:829:ARG:CB	2.47	0.61

Continued on next page...

Continued from previous page...

Atom-1	Atom-2	Interatomic distance (Å)	Clash overlap (Å)
4:D:488:ASN:HB3	4:D:501:TRP:CZ3	2.35	0.61
4:D:117:ILE:HG23	4:D:752:LEU:HD21	1.82	0.61
4:D:473:VAL:HG21	4:D:477:GLU:OE1	2.00	0.61
4:D:565:GLU:HG2	4:D:566:THR:N	2.15	0.61
4:D:298:ARG:CZ	4:D:419:ASN:HD21	2.14	0.61
4:D:362:MET:C	4:D:381:ALA:HB2	2.22	0.60
4:D:720:ARG:HH12	4:D:854:HIS:HA	1.67	0.60
4:D:359:GLU:O	4:D:360:LEU:HG	2.02	0.60
4:D:546:PHE:CE2	4:D:638:ALA:HB1	2.36	0.60
4:D:298:ARG:NH2	4:D:419:ASN:HD21	1.98	0.60
4:D:286:TYR:CZ	4:D:417:PRO:HG3	2.37	0.60
4:D:579:ASN:HA	4:D:582:LEU:HD12	1.83	0.60
4:D:50:ARG:HG3	4:D:51:PHE:N	2.16	0.60
4:D:729:THR:OG1	4:D:733:PHE:HB3	2.01	0.60
4:D:706:LEU:HD12	4:D:725:VAL:HG12	1.83	0.60
4:D:743:ILE:HD12	4:D:744:GLN:H	1.66	0.60
2:N:24:DG:H2”	2:N:25:DC:C5	2.37	0.59
4:D:378:LYS:C	4:D:378:LYS:HD2	2.22	0.59
4:D:40:GLU:CD	4:D:286:TYR:HB3	2.22	0.59
4:D:71:LYS:N	4:D:72:PRO:HD2	2.17	0.59
4:D:747:LEU:N	4:D:747:LEU:HD23	2.18	0.59
4:D:576:LYS:HA	4:D:576:LYS:NZ	2.17	0.59
4:D:118:THR:O	4:D:122:THR:HG23	2.03	0.59
4:D:151:PHE:C	4:D:153:ARG:H	2.06	0.59
4:D:169:GLN:NE2	4:D:169:GLN:H	2.00	0.59
4:D:230:HIS:O	4:D:231:ARG:HG3	2.02	0.59
4:D:423:ARG:HB2	4:D:423:ARG:HH11	1.68	0.59
4:D:349:VAL:HG22	4:D:503:ALA:CB	2.30	0.58
4:D:663:LYS:CD	4:D:663:LYS:H	2.16	0.58
4:D:784:HIS:HA	4:D:787:ASP:OD2	2.03	0.58
4:D:421:ASP:O	4:D:422:TRP:HB3	2.03	0.58
4:D:402:LEU:HD13	4:D:439:MET:HE3	1.84	0.58
4:D:452:ILE:HG23	4:D:453:GLY:N	2.19	0.58
4:D:463:HIS:HB2	4:D:534:LEU:HD22	1.85	0.58
4:D:567:VAL:HB	4:D:880:PHE:CD1	2.39	0.58
4:D:88:TRP:O	4:D:92:VAL:HG23	2.04	0.58
4:D:551:ARG:O	4:D:870:LEU:HB3	2.04	0.57
1:T:120:DT:H5’	4:D:641:SER:HA	1.86	0.57
4:D:731:ASP:HB3	4:D:793:LYS:HD2	1.84	0.57
4:D:755:PHE:HA	4:D:756:ARG:NH2	2.15	0.57
4:D:143:ARG:HH21	4:D:206:LYS:HG2	1.67	0.57

Continued on next page...

Continued from previous page...

Atom-1	Atom-2	Interatomic distance (Å)	Clash overlap (Å)
4:D:544:GLN:HG2	4:D:559:VAL:HB	1.86	0.57
4:D:54:MET:HA	4:D:57:ARG:HG3	1.86	0.57
4:D:54:MET:O	4:D:57:ARG:HG3	2.04	0.57
4:D:126:LEU:HD22	4:D:246:LEU:HD23	1.86	0.57
4:D:150:ARG:NE	4:D:150:ARG:HA	2.19	0.57
2:N:28:DG:H2”	2:N:29:DG:N7	2.19	0.57
4:D:218:GLU:C	4:D:220:LEU:H	2.07	0.57
4:D:759:PRO:HG2	4:D:764:ASN:HD22	1.61	0.57
4:D:125:CYS:HA	4:D:128:SER:OG	2.04	0.57
2:N:17:DA:OP1	4:D:378:LYS:CD	2.53	0.57
4:D:115:ALA:O	4:D:118:THR:HB	2.04	0.57
2:N:27:DC:C1’	2:N:28:DG:H5”	2.30	0.57
4:D:495:SER:CB	4:D:498:GLU:HB2	2.33	0.57
4:D:565:GLU:CG	4:D:566:THR:H	2.18	0.57
4:D:712:ASP:O	4:D:716:GLY:HA2	2.05	0.57
4:D:741:LYS:HD2	4:D:768:GLU:OE1	2.05	0.56
4:D:154:ILE:CG2	4:D:162:PHE:HB2	2.34	0.56
4:D:228:SER:HB2	4:D:245:GLU:CG	2.36	0.56
4:D:226:MET:HA	4:D:250:TYR:HD1	1.71	0.56
4:D:540:CYS:O	4:D:544:GLN:HG3	2.04	0.56
4:D:571:TYR:HD2	4:D:627:ARG:NH2	2.03	0.56
4:D:155:ARG:H	4:D:155:ARG:HD2	1.69	0.56
4:D:308:TYR:CE2	4:D:734:PRO:HG2	2.41	0.56
4:D:473:VAL:HG21	4:D:477:GLU:CD	2.25	0.56
4:D:629:VAL:O	4:D:631:LYS:N	2.36	0.56
4:D:613:THR:HG23	4:D:676:TYR:CE1	2.41	0.56
4:D:219:MET:H	4:D:222:GLU:HG3	1.69	0.56
4:D:423:ARG:HD2	4:D:781:ASN:ND2	2.21	0.56
2:N:19:DG:H21	4:D:670:PRO:HD2	1.71	0.55
4:D:816:THR:OG1	4:D:824:LEU:HD22	2.05	0.55
4:D:473:VAL:HG22	4:D:474:PRO:CD	2.34	0.55
4:D:61:ALA:O	4:D:63:GLU:HG3	2.07	0.55
4:D:105:PHE:HB3	4:D:204:TRP:CZ3	2.40	0.55
4:D:298:ARG:HB2	4:D:421:ASP:HA	1.88	0.55
4:D:468:ALA:HA	4:D:505:GLN:HB3	1.89	0.55
4:D:109:ILE:HD12	4:D:109:ILE:H	1.72	0.55
4:D:725:VAL:HG23	4:D:737:GLN:HB3	1.88	0.55
4:D:400:PHE:HA	4:D:403:GLU:OE2	2.07	0.55
4:D:589:GLY:HA2	4:D:613:THR:HB	1.88	0.55
4:D:725:VAL:CG2	4:D:737:GLN:HB3	2.36	0.55
4:D:752:LEU:HD12	4:D:752:LEU:H	1.71	0.55

Continued on next page...

Continued from previous page...

Atom-1	Atom-2	Interatomic distance (Å)	Clash overlap (Å)
4:D:472:LYS:NZ	4:D:472:LYS:HB2	2.21	0.55
4:D:70:ALA:C	4:D:72:PRO:HD2	2.26	0.55
4:D:143:ARG:NH2	4:D:206:LYS:HG2	2.22	0.55
4:D:48:GLU:HG2	4:D:262:ALA:O	2.07	0.55
4:D:69:ALA:HA	4:D:257:ARG:HD2	1.89	0.55
1:T:115:DC:H2"	1:T:114:DG:H5'	1.88	0.55
2:N:22:DG:H2"	2:N:23:DC:C5	2.41	0.54
1:T:120:DT:C4	4:D:636:THR:HG21	2.41	0.54
4:D:48:GLU:HG2	4:D:262:ALA:C	2.26	0.54
4:D:565:GLU:CG	4:D:566:THR:N	2.69	0.54
4:D:500:THR:O	4:D:501:TRP:CB	2.55	0.54
1:T:119:DT:H2"	1:T:118:DC:OP2	2.07	0.54
4:D:218:GLU:O	4:D:219:MET:HB2	2.07	0.54
4:D:409:ALA:O	4:D:410:ASN:HB2	2.08	0.54
4:D:416:PHE:CZ	4:D:434:PRO:HD3	2.43	0.54
4:D:190:MET:CE	4:D:191:LEU:H	2.21	0.54
4:D:309:GLU:HG3	4:D:310:ASP:OD1	2.07	0.54
4:D:543:ILE:HB	4:D:559:VAL:HG11	1.89	0.54
4:D:579:ASN:HD22	4:D:582:LEU:HD12	1.72	0.54
4:D:629:VAL:C	4:D:631:LYS:H	2.11	0.54
2:N:27:DC:H2"	2:N:28:DG:O5'	2.08	0.54
4:D:40:GLU:OE2	4:D:288:ALA:HB3	2.08	0.54
4:D:298:ARG:HH21	4:D:419:ASN:HD21	1.56	0.54
4:D:28:TYR:CZ	4:D:274:PRO:HD2	2.43	0.53
4:D:433:ASN:HD22	4:D:435:GLN:N	2.01	0.53
4:D:534:LEU:CD1	4:D:818:PRO:HA	2.38	0.53
4:D:757:LEU:HD23	4:D:758:GLN:N	2.24	0.53
4:D:110:LYS:O	4:D:114:VAL:HG23	2.08	0.53
4:D:668:THR:O	4:D:669:GLN:HB3	2.08	0.53
4:D:740:LYS:HD3	4:D:769:ILE:HA	1.90	0.53
4:D:796:VAL:O	4:D:800:GLU:HG3	2.08	0.53
4:D:613:THR:O	4:D:616:LEU:N	2.35	0.53
4:D:629:VAL:HG12	4:D:630:THR:N	2.21	0.53
4:D:855:GLU:O	4:D:856:SER:HB2	2.08	0.53
2:N:20:DT:H2"	2:N:21:DT:C7	2.37	0.53
3:R:4:C:O2'	3:R:3:G:H5'	2.09	0.53
4:D:133:THR:HG23	4:D:136:ALA:H	1.72	0.53
4:D:612:GLY:O	4:D:616:LEU:HB2	2.09	0.53
4:D:748:ASN:O	4:D:749:LEU:HD23	2.08	0.53
4:D:759:PRO:CB	4:D:764:ASN:ND2	2.65	0.53
4:D:25:ALA:C	4:D:27:HIS:H	2.11	0.53

Continued on next page...

Continued from previous page...

Atom-1	Atom-2	Interatomic distance (Å)	Clash overlap (Å)
3:R:2:A:H2'	5:R:901:APC:C8	2.35	0.53
4:D:466:ASN:HD21	4:D:478:ARG:NH1	2.07	0.53
4:D:585:ASP:O	4:D:586:ALA:CB	2.57	0.53
4:D:746:ARG:N	4:D:746:ARG:HD3	2.22	0.53
4:D:162:PHE:CE1	4:D:167:GLU:HB2	2.43	0.53
4:D:468:ALA:HB2	4:D:511:PHE:CE1	2.44	0.53
4:D:585:ASP:O	4:D:586:ALA:HB2	2.08	0.53
4:D:810:ILE:O	4:D:811:HIS:HB2	2.09	0.53
4:D:852:GLN:OE1	4:D:853:LEU:HB2	2.08	0.53
4:D:852:GLN:OE1	4:D:853:LEU:HD13	2.09	0.53
4:D:169:GLN:NE2	4:D:169:GLN:N	2.56	0.53
4:D:576:LYS:HZ1	4:D:576:LYS:HA	1.74	0.53
4:D:416:PHE:CE2	4:D:434:PRO:HD3	2.43	0.52
4:D:77:LEU:HD22	4:D:224:THR:HB	1.91	0.52
4:D:180:LYS:HA	4:D:751:PHE:CE1	2.45	0.52
2:N:27:DC:H1'	2:N:28:DG:OP1	2.08	0.52
4:D:360:LEU:N	4:D:361:PRO:CD	2.70	0.52
4:D:791:LEU:HD21	4:D:809:LEU:HD13	1.92	0.52
4:D:264:ILE:HD12	4:D:264:ILE:O	2.08	0.52
4:D:421:ASP:OD1	4:D:423:ARG:NH1	2.37	0.52
4:D:104:GLN:NE2	4:D:104:GLN:HA	2.24	0.52
4:D:881:ALA:O	4:D:882:PHE:HB2	2.09	0.52
4:D:538:GLY:HA3	4:D:883:ALA:HB3	1.91	0.52
4:D:743:ILE:CD1	4:D:744:GLN:H	2.23	0.52
4:D:704:LYS:HB2	4:D:775:GLU:OE2	2.09	0.52
4:D:152:GLY:CA	4:D:155:ARG:HD3	2.38	0.52
4:D:550:LEU:HD11	4:D:695:ALA:CB	2.38	0.52
4:D:155:ARG:CD	4:D:155:ARG:H	2.23	0.52
4:D:85:ILE:HD13	4:D:219:MET:SD	2.49	0.52
4:D:141:ILE:O	4:D:145:ILE:HG12	2.10	0.52
4:D:147:ASP:O	4:D:148:GLU:HG2	2.10	0.51
4:D:665:LEU:HD23	4:D:665:LEU:H	1.74	0.51
4:D:791:LEU:O	4:D:795:VAL:HG23	2.11	0.51
4:D:102:ALA:O	4:D:106:LEU:HB2	2.10	0.51
4:D:228:SER:HB2	4:D:245:GLU:HG3	1.92	0.51
4:D:655:ILE:CD1	4:D:674:ALA:HB2	2.40	0.51
4:D:655:ILE:HG21	4:D:670:PRO:HB3	1.90	0.51
4:D:151:PHE:O	4:D:153:ARG:HG3	2.10	0.51
4:D:278:TRP:CE2	4:D:284:GLY:HA3	2.45	0.51
4:D:248:PRO:O	4:D:252:GLU:HG3	2.10	0.51
4:D:707:ALA:O	4:D:722:ARG:HG2	2.10	0.51

Continued on next page...

Continued from previous page...

Atom-1	Atom-2	Interatomic distance (Å)	Clash overlap (Å)
4:D:421:ASP:O	4:D:422:TRP:CB	2.58	0.51
4:D:856:SER:O	4:D:857:GLN:CB	2.53	0.51
4:D:85:ILE:HD13	4:D:219:MET:CE	2.41	0.51
4:D:24:LEU:O	4:D:28:TYR:N	2.39	0.51
4:D:731:ASP:OD2	4:D:792:ARG:NE	2.43	0.51
4:D:459:TRP:O	4:D:534:LEU:HD11	2.11	0.50
4:D:613:THR:O	4:D:615:ALA:N	2.45	0.50
4:D:308:TYR:HE2	4:D:734:PRO:HG2	1.75	0.50
4:D:343:LYS:HA	4:D:355:ILE:HD11	1.94	0.50
4:D:67:ASN:ND2	4:D:69:ALA:H	2.03	0.50
4:D:492:CYS:HB3	4:D:502:TRP:CD1	2.47	0.50
4:D:698:TRP:HZ3	4:D:845:PHE:HD2	1.58	0.50
4:D:108:GLU:O	4:D:150:ARG:HB2	2.11	0.50
4:D:266:PRO:HG2	4:D:268:PHE:CZ	2.46	0.50
4:D:419:ASN:C	4:D:419:ASN:ND2	2.64	0.50
4:D:547:SER:HA	4:D:552:ASP:HB3	1.94	0.50
2:N:17:DA:P	4:D:378:LYS:CD	2.98	0.50
2:N:26:DA:H2”	2:N:27:DC:C6	2.46	0.50
4:D:303:LYS:H	4:D:303:LYS:HE2	1.77	0.50
4:D:35:GLU:HG2	4:D:272:VAL:HG21	1.92	0.50
4:D:655:ILE:O	4:D:659:ILE:HG12	2.12	0.50
4:D:307:ARG:HG2	4:D:736:TRP:CE3	2.47	0.50
4:D:852:GLN:O	4:D:853:LEU:C	2.50	0.50
4:D:308:TYR:HA	4:D:311:VAL:CG1	2.40	0.50
4:D:720:ARG:NH1	4:D:854:HIS:HA	2.26	0.50
4:D:816:THR:HG22	4:D:817:ILE:H	1.76	0.50
4:D:855:GLU:CD	4:D:856:SER:H	2.14	0.50
4:D:554:VAL:O	4:D:557:ARG:HB3	2.11	0.50
5:R:901:APC:H3A1	5:R:901:APC:H3’	1.94	0.50
4:D:766:ASP:O	4:D:767:SER:CB	2.57	0.49
4:D:146:GLU:C	4:D:148:GLU:H	2.14	0.49
4:D:85:ILE:HA	4:D:219:MET:HE1	1.94	0.49
4:D:378:LYS:HD2	4:D:378:LYS:O	2.13	0.49
4:D:357:ARG:HG2	4:D:357:ARG:HH11	1.77	0.49
4:D:126:LEU:HD22	4:D:246:LEU:CD2	2.43	0.49
4:D:155:ARG:HG3	4:D:162:PHE:CE2	2.48	0.49
4:D:57:ARG:HG2	4:D:57:ARG:HH11	1.78	0.49
4:D:630:THR:HA	4:D:681:ILE:HD13	1.95	0.49
4:D:855:GLU:OE1	4:D:856:SER:N	2.45	0.49
4:D:353:PRO:HD2	4:D:398:LEU:CD1	2.43	0.49
3:R:2:A:C2’	5:R:901:APC:O4’	2.61	0.49

Continued on next page...

Continued from previous page...

Atom-1	Atom-2	Interatomic distance (Å)	Clash overlap (Å)
4:D:724:ALA:HB2	4:D:738:GLU:CB	2.25	0.49
1:T:122:DC:H2”	1:T:121:DA:OP2	2.12	0.49
4:D:565:GLU:HG2	4:D:566:THR:H	1.75	0.49
4:D:613:THR:HG22	4:D:614:LYS:N	2.27	0.48
4:D:709:GLU:HB2	4:D:722:ARG:HG3	1.95	0.48
4:D:645:GLY:O	4:D:649:GLN:HG3	2.14	0.48
1:T:130:DG:H2”	1:T:129:DC:H5	1.78	0.48
4:D:228:SER:C	4:D:229:LEU:HD22	2.34	0.48
4:D:423:ARG:HH21	4:D:784:HIS:CD2	2.31	0.48
4:D:66:ASP:O	4:D:71:LYS:NZ	2.44	0.48
2:N:22:DG:H2”	2:N:23:DC:H5	1.78	0.48
4:D:120:LYS:HE2	4:D:752:LEU:HB3	1.96	0.48
4:D:828:VAL:CG2	4:D:883:ALA:HA	2.44	0.48
3:R:9:C:H5’	4:D:746:ARG:HH22	1.78	0.48
4:D:257:ARG:O	4:D:260:ALA:HB3	2.14	0.48
4:D:557:ARG:HH12	4:D:564:SER:HB2	1.79	0.48
4:D:756:ARG:O	4:D:757:LEU:HB2	2.14	0.48
4:D:231:ARG:HA	4:D:242:GLU:OE1	2.13	0.48
4:D:488:ASN:HB3	4:D:501:TRP:CE3	2.49	0.48
4:D:590:THR:H	4:D:613:THR:HB	1.79	0.48
4:D:586:ALA:H	4:D:589:GLY:H	1.62	0.48
4:D:783:VAL:O	4:D:786:GLN:HB2	2.14	0.48
4:D:613:THR:O	4:D:614:LYS:C	2.52	0.48
4:D:744:GLN:HB3	4:D:757:LEU:H	1.79	0.48
4:D:798:ALA:O	4:D:802:TYR:HB2	2.14	0.48
1:T:126:DT:H1’	1:T:125:DG:H5”	1.96	0.48
4:D:122:THR:O	4:D:126:LEU:HB2	2.14	0.47
4:D:298:ARG:HH21	4:D:419:ASN:ND2	2.11	0.47
4:D:761:ILE:O	4:D:763:THR:N	2.46	0.47
4:D:109:ILE:CD1	4:D:109:ILE:H	2.25	0.47
4:D:298:ARG:HE	4:D:419:ASN:CG	2.18	0.47
4:D:790:HIS:ND1	4:D:831:THR:HG22	2.29	0.47
4:D:446:LEU:O	4:D:531:SER:HB2	2.14	0.47
4:D:707:ALA:HB1	4:D:771:ALA:HA	1.96	0.47
1:T:118:DC:H2”	1:T:117:DG:OP2	2.13	0.47
4:D:229:LEU:HD22	4:D:229:LEU:N	2.29	0.47
4:D:57:ARG:HD3	4:D:58:GLN:N	2.30	0.47
4:D:631:LYS:HE3	4:D:635:MET:CE	2.44	0.47
4:D:102:ALA:O	4:D:106:LEU:HD12	2.15	0.47
4:D:671:ASN:C	4:D:673:ALA:H	2.18	0.47
4:D:567:VAL:HB	4:D:880:PHE:CG	2.50	0.47

Continued on next page...

Continued from previous page...

Atom-1	Atom-2	Interatomic distance (Å)	Clash overlap (Å)
4:D:689:VAL:CG1	4:D:692:ALA:HB3	2.43	0.46
2:N:20:DT:O4	4:D:168:GLU:OE1	2.33	0.46
4:D:452:ILE:HG23	4:D:453:GLY:H	1.79	0.46
4:D:491:ALA:HB1	4:D:499:ASN:OD1	2.15	0.46
3:R:2:A:H2'	5:R:901:APC:O4'	2.15	0.46
4:D:109:ILE:N	4:D:109:ILE:HD12	2.30	0.46
4:D:537:ASP:O	4:D:882:PHE:HD1	1.98	0.46
1:T:111:DT:H3'	1:T:111:DT:OP2	2.15	0.46
4:D:126:LEU:HD11	4:D:244:ILE:HG22	1.96	0.46
4:D:353:PRO:HD2	4:D:398:LEU:HD12	1.98	0.46
4:D:298:ARG:HG3	4:D:420:MET:O	2.15	0.46
4:D:416:PHE:CE1	4:D:433:ASN:HA	2.50	0.46
2:N:28:DG:H2"	2:N:29:DG:C8	2.50	0.46
1:T:117:DG:H5"	4:D:421:ASP:CB	2.46	0.46
4:D:104:GLN:CA	4:D:104:GLN:NE2	2.79	0.46
4:D:150:ARG:O	4:D:150:ARG:HG3	2.15	0.46
4:D:588:ASN:O	4:D:613:THR:HG21	2.15	0.46
4:D:656:GLN:N	4:D:657:PRO:HD2	2.31	0.46
4:D:779:ALA:HB3	4:D:780:PRO:CD	2.45	0.46
4:D:565:GLU:CD	4:D:566:THR:H	2.18	0.46
4:D:57:ARG:HD3	4:D:58:GLN:HG3	1.96	0.46
4:D:307:ARG:HG2	4:D:736:TRP:CH2	2.51	0.46
4:D:150:ARG:HE	4:D:150:ARG:HA	1.81	0.46
4:D:109:ILE:HG13	4:D:149:ALA:HA	1.97	0.46
4:D:248:PRO:O	4:D:252:GLU:CG	2.64	0.46
2:N:29:DG:H2"	2:N:30:DC:OP2	2.15	0.46
4:D:576:LYS:CA	4:D:576:LYS:NZ	2.79	0.45
4:D:375:THR:O	4:D:375:THR:OG1	2.33	0.45
4:D:655:ILE:HG21	4:D:670:PRO:CB	2.46	0.45
4:D:34:ARG:HG2	4:D:34:ARG:HH11	1.81	0.45
4:D:353:PRO:O	4:D:395:ARG:NH1	2.48	0.45
4:D:398:LEU:HD23	4:D:398:LEU:C	2.36	0.45
1:T:130:DG:H8	1:T:130:DG:P	2.40	0.45
4:D:744:GLN:HE21	4:D:758:GLN:HA	1.81	0.45
4:D:861:MET:HE3	4:D:862:PRO:CD	2.30	0.45
4:D:137:VAL:O	4:D:140:ALA:HB3	2.16	0.45
4:D:162:PHE:O	4:D:167:GLU:N	2.48	0.45
4:D:40:GLU:OE1	4:D:287:TRP:N	2.48	0.45
4:D:421:ASP:O	4:D:422:TRP:CD1	2.68	0.45
4:D:507:SER:O	4:D:511:PHE:HB2	2.17	0.45
4:D:210:ILE:HG13	4:D:211:HIS:N	2.31	0.45

Continued on next page...

Continued from previous page...

Atom-1	Atom-2	Interatomic distance (Å)	Clash overlap (Å)
5:R:901:APC:H1'	4:D:784:HIS:HE1	1.80	0.45
4:D:551:ARG:C	4:D:870:LEU:HB3	2.37	0.45
4:D:89:PHE:O	4:D:92:VAL:N	2.50	0.45
4:D:213:GLY:O	4:D:217:ILE:N	2.42	0.45
4:D:218:GLU:C	4:D:220:LEU:N	2.71	0.45
2:N:17:DA:OP1	4:D:378:LYS:HD3	2.11	0.45
4:D:496:PRO:HG2	4:D:497:LEU:HG	1.99	0.45
3:R:6:G:OP1	4:D:172:LYS:NZ	2.50	0.45
4:D:210:ILE:HG13	4:D:211:HIS:H	1.82	0.44
4:D:671:ASN:O	4:D:672:GLN:HB3	2.17	0.44
4:D:625:VAL:HG21	4:D:677:MET:CE	2.47	0.44
1:T:127:DG:H2"	1:T:126:DT:C5	2.52	0.44
4:D:502:TRP:CZ3	4:D:512:LEU:HD22	2.52	0.44
4:D:446:LEU:HD13	4:D:806:SER:HB3	1.98	0.44
4:D:705:LEU:HD13	4:D:854:HIS:HE1	1.82	0.44
4:D:756:ARG:N	4:D:756:ARG:NE	2.54	0.44
4:D:778:ILE:HG23	4:D:779:ALA:N	2.32	0.44
4:D:625:VAL:HG21	4:D:677:MET:HE1	1.98	0.44
4:D:782:PHE:O	4:D:786:GLN:HG2	2.17	0.44
4:D:737:GLN:HE22	4:D:778:ILE:N	2.15	0.44
4:D:855:GLU:CD	4:D:856:SER:N	2.71	0.44
4:D:90:GLU:O	4:D:93:LYS:HB2	2.18	0.44
4:D:763:THR:C	4:D:764:ASN:CG	2.74	0.44
4:D:96:ARG:HA	4:D:96:ARG:NE	2.31	0.44
4:D:184:GLN:NE2	4:D:184:GLN:HA	2.33	0.44
4:D:50:ARG:HG2	4:D:50:ARG:NH1	2.33	0.44
4:D:433:ASN:C	4:D:435:GLN:H	2.22	0.43
4:D:104:GLN:CA	4:D:104:GLN:HE21	2.26	0.43
4:D:463:HIS:CB	4:D:534:LEU:HD22	2.47	0.43
4:D:631:LYS:CD	4:D:632:ARG:N	2.77	0.43
4:D:720:ARG:HD2	4:D:721:LYS:N	2.34	0.43
1:T:116:DC:O5'	1:T:116:DC:H2'	2.19	0.43
1:T:124:DC:H1'	1:T:123:DG:C5'	2.48	0.43
4:D:135:GLN:HE21	4:D:241:SER:HA	1.83	0.43
4:D:172:LYS:HB3	4:D:173:ARG:NH2	2.33	0.43
4:D:126:LEU:HD21	4:D:244:ILE:O	2.18	0.43
4:D:120:LYS:HE2	4:D:752:LEU:CD2	2.42	0.43
4:D:88:TRP:HB2	4:D:219:MET:HE3	2.01	0.43
4:D:59:LEU:HD12	4:D:64:VAL:HG22	1.99	0.43
4:D:644:PHE:HE1	4:D:647:ARG:NH1	2.16	0.43
1:T:119:DT:H5"	4:D:780:PRO:HG3	2.00	0.43

Continued on next page...

Continued from previous page...

Atom-1	Atom-2	Interatomic distance (Å)	Clash overlap (Å)
3:R:5:G:O2'	3:R:4:C:H5'	2.18	0.43
4:D:151:PHE:C	4:D:153:ARG:N	2.71	0.43
1:T:126:DT:H2"	1:T:125:DG:H5'	2.00	0.43
4:D:154:ILE:HA	4:D:159:ALA:HB3	2.01	0.43
4:D:201:TRP:HZ2	4:D:204:TRP:HE1	1.66	0.43
1:T:113:DT:H2"	1:T:112:DG:H5'	1.99	0.43
1:T:124:DC:H2"	1:T:123:DG:OP2	2.19	0.43
4:D:24:LEU:HD21	4:D:287:TRP:CD2	2.54	0.43
4:D:402:LEU:HA	4:D:402:LEU:HD12	1.83	0.43
4:D:475:PHE:HB2	4:D:476:PRO:HD3	2.01	0.43
4:D:650:VAL:HG13	4:D:651:LEU:N	2.33	0.43
4:D:752:LEU:HD12	4:D:752:LEU:N	2.33	0.43
4:D:814:PHE:N	4:D:814:PHE:CD1	2.87	0.43
4:D:775:GLU:O	4:D:778:ILE:HG22	2.19	0.43
4:D:204:TRP:HZ3	4:D:212:VAL:HG11	1.84	0.43
4:D:217:ILE:O	4:D:221:ILE:HG13	2.19	0.42
4:D:46:MET:CE	4:D:269:GLN:NE2	2.80	0.42
4:D:810:ILE:O	4:D:810:ILE:HG22	2.19	0.42
4:D:155:ARG:HH21	4:D:750:MET:HG2	1.84	0.42
4:D:386:ARG:HD3	4:D:387:LYS:H	1.80	0.42
4:D:500:THR:HA	4:D:502:TRP:NE1	2.34	0.42
4:D:698:TRP:CZ3	4:D:845:PHE:HD2	2.35	0.42
4:D:149:ALA:HB2	4:D:204:TRP:CZ2	2.54	0.42
4:D:30:GLU:O	4:D:34:ARG:HG3	2.19	0.42
4:D:190:MET:HE3	4:D:191:LEU:H	1.83	0.42
4:D:320:ILE:HD11	4:D:426:VAL:HG22	2.00	0.42
4:D:631:LYS:CG	4:D:632:ARG:N	2.82	0.42
4:D:296:LEU:HD22	4:D:317:TYR:CE1	2.55	0.42
4:D:402:LEU:HD13	4:D:439:MET:CE	2.49	0.42
4:D:502:TRP:CD2	4:D:512:LEU:HD13	2.54	0.42
2:N:19:DG:N2	4:D:670:PRO:HD2	2.35	0.42
4:D:248:PRO:O	4:D:252:GLU:CD	2.58	0.42
4:D:791:LEU:HD12	4:D:811:HIS:O	2.20	0.42
4:D:162:PHE:CD1	4:D:167:GLU:HB2	2.55	0.42
4:D:828:VAL:HG13	4:D:882:PHE:O	2.20	0.42
4:D:132:THR:O	4:D:244:ILE:N	2.45	0.42
4:D:421:ASP:CG	4:D:423:ARG:NH1	2.73	0.42
4:D:485:ASN:O	4:D:486:HIS:C	2.57	0.42
4:D:420:MET:HA	4:D:425:ARG:O	2.20	0.42
4:D:685:VAL:HG13	4:D:686:SER:N	2.34	0.42
4:D:117:ILE:HG23	4:D:752:LEU:CD2	2.49	0.41

Continued on next page...

Continued from previous page...

Atom-1	Atom-2	Interatomic distance (Å)	Clash overlap (Å)
4:D:170:LEU:HD11	4:D:179:LYS:HB3	2.02	0.41
4:D:203:SER:C	4:D:205:HIS:H	2.23	0.41
4:D:533:PRO:HA	4:D:816:THR:O	2.20	0.41
4:D:747:LEU:HD23	4:D:755:PHE:O	2.20	0.41
1:T:120:DT:C2’	1:T:119:DT:OP2	2.53	0.41
4:D:143:ARG:HH11	4:D:143:ARG:HG2	1.85	0.41
4:D:219:MET:H	4:D:222:GLU:CG	2.34	0.41
4:D:653:ASP:O	4:D:657:PRO:HG3	2.20	0.41
4:D:712:ASP:O	4:D:716:GLY:CA	2.67	0.41
2:N:20:DT:C2’	2:N:21:DT:H72	2.47	0.41
3:R:2:A:C2’	5:R:901:APC:H5’1	2.50	0.41
1:T:115:DC:H4’	4:D:431:MET:HE2	2.01	0.41
4:D:40:GLU:OE2	4:D:286:TYR:HB3	2.20	0.41
4:D:506:ASP:N	4:D:506:ASP:OD2	2.43	0.41
4:D:54:MET:C	4:D:56:GLU:N	2.74	0.41
4:D:799:HIS:HD2	4:D:804:ILE:O	2.03	0.41
4:D:843:ALA:O	4:D:847:ASP:OD2	2.38	0.41
4:D:853:LEU:CD1	4:D:853:LEU:H	2.30	0.41
1:T:120:DT:H2’	1:T:120:DT:O5’	2.20	0.41
4:D:151:PHE:O	4:D:153:ARG:N	2.43	0.41
4:D:749:LEU:O	4:D:750:MET:HB2	2.20	0.41
4:D:424:GLY:O	4:D:792:ARG:NH1	2.54	0.41
4:D:268:PHE:HB3	4:D:286:TYR:OH	2.21	0.41
4:D:576:LYS:CE	4:D:576:LYS:HA	2.50	0.41
4:D:860:LYS:O	4:D:862:PRO:HD3	2.20	0.41
4:D:258:ALA:O	4:D:259:GLY:C	2.56	0.41
4:D:272:VAL:HG12	4:D:411:HIS:CD2	2.55	0.41
4:D:297:VAL:CG1	4:D:422:TRP:HA	2.50	0.41
4:D:459:TRP:O	4:D:534:LEU:CD1	2.68	0.41
4:D:579:ASN:HD22	4:D:579:ASN:HA	1.70	0.41
4:D:19:ILE:HG23	4:D:19:ILE:O	2.20	0.41
4:D:357:ARG:HA	4:D:388:ASP:OD2	2.20	0.41
4:D:826:LYS:O	4:D:828:VAL:O	2.39	0.41
4:D:789:SER:O	4:D:793:LYS:HB2	2.20	0.41
4:D:378:LYS:HE2	4:D:378:LYS:HB3	1.84	0.41
4:D:134:VAL:HG23	4:D:135:GLN:N	2.36	0.41
4:D:264:ILE:HG22	4:D:292:ARG:HG3	2.03	0.41
4:D:361:PRO:O	4:D:362:MET:CB	2.69	0.41
4:D:701:SER:O	4:D:705:LEU:HG	2.21	0.41
4:D:275:PRO:HB2	4:D:324:GLN:HG3	2.01	0.40
4:D:357:ARG:HG2	4:D:357:ARG:NH1	2.36	0.40

Continued on next page...

Continued from previous page...

Atom-1	Atom-2	Interatomic distance (Å)	Clash overlap (Å)
4:D:667:PHE:O	4:D:668:THR:C	2.59	0.40
1:T:126:DT:H2"	1:T:125:DG:OP2	2.21	0.40
4:D:311:VAL:CG2	4:D:313:MET:SD	3.09	0.40
4:D:349:VAL:O	4:D:351:ASP:N	2.54	0.40
4:D:551:ARG:HG3	4:D:551:ARG:HH11	1.85	0.40
4:D:659:ILE:O	4:D:661:SER:N	2.54	0.40
4:D:274:PRO:HA	4:D:275:PRO:HD3	1.96	0.40
4:D:328:TRP:CH2	4:D:434:PRO:HB3	2.56	0.40
4:D:544:GLN:HA	4:D:559:VAL:HG21	2.03	0.40
4:D:644:PHE:HE1	4:D:647:ARG:CZ	2.34	0.40
4:D:337:VAL:HG21	4:D:512:LEU:HD21	2.03	0.40
4:D:33:ALA:O	4:D:37:LEU:HD22	2.22	0.40
4:D:297:VAL:HG13	4:D:422:TRP:HA	2.03	0.40
4:D:637:LEU:HD22	4:D:685:VAL:HG13	2.02	0.40
4:D:330:ILE:HD12	4:D:405:ALA:HA	2.04	0.40

There are no symmetry-related clashes.

5.3 Torsion angles (i)

5.3.1 Protein backbone (i)

In the following table, the Percentiles column shows the percent Ramachandran outliers of the chain as a percentile score with respect to all X-ray entries followed by that with respect to entries of similar resolution.

The Analysed column shows the number of residues for which the backbone conformation was analysed, and the total number of residues.

Mol	Chain	Analysed	Favoured	Allowed	Outliers	Percentiles
4	D	821/883 (93%)	663 (81%)	111 (14%)	47 (6%)	1 5

All (47) Ramachandran outliers are listed below:

Mol	Chain	Res	Type
4	D	15	GLU
4	D	149	ALA
4	D	226	MET
4	D	360	LEU
4	D	422	TRP
4	D	460	LEU

Continued on next page...

Continued from previous page...

Mol	Chain	Res	Type
4	D	613	THR
4	D	614	LYS
4	D	716	GLY
4	D	764	ASN
4	D	767	SER
4	D	829	ARG
4	D	857	GLN
4	D	16	LEU
4	D	20	PRO
4	D	437	ASN
4	D	501	TRP
4	D	586	ALA
4	D	661	SER
4	D	762	ASN
4	D	854	HIS
4	D	859	ASP
4	D	192	SER
4	D	205	HIS
4	D	230	HIS
4	D	350	GLU
4	D	351	ASP
4	D	357	ARG
4	D	500	THR
4	D	630	THR
4	D	668	THR
4	D	850	ALA
4	D	853	LEU
4	D	148	GLU
4	D	157	LEU
4	D	164	LYS
4	D	200	ALA
4	D	344	TRP
4	D	659	ILE
4	D	669	GLN
4	D	882	PHE
4	D	118	THR
4	D	260	ALA
4	D	660	ASP
4	D	114	VAL
4	D	434	PRO
4	D	559	VAL

5.3.2 Protein sidechains (i)

In the following table, the Percentiles column shows the percent sidechain outliers of the chain as a percentile score with respect to all X-ray entries followed by that with respect to entries of similar resolution.

The Analysed column shows the number of residues for which the sidechain conformation was analysed, and the total number of residues.

Mol	Chain	Analysed	Rotameric	Outliers	Percentiles
4	D	683/729 (94%)	630 (92%)	53 (8%)	12 33

All (53) residues with a non-rotameric sidechain are listed below:

Mol	Chain	Res	Type
4	D	27	HIS
4	D	37	LEU
4	D	50	ARG
4	D	57	ARG
4	D	109	ILE
4	D	120	LYS
4	D	151	PHE
4	D	154	ILE
4	D	169	GLN
4	D	190	MET
4	D	215	ARG
4	D	232	GLN
4	D	242	GLU
4	D	257	ARG
4	D	281	ILE
4	D	310	ASP
4	D	311	VAL
4	D	325	ASN
4	D	335	LEU
4	D	350	GLU
4	D	362	MET
4	D	377	TRP
4	D	386	ARG
4	D	401	MET
4	D	402	LEU
4	D	419	ASN
4	D	423	ARG
4	D	433	ASN
4	D	443	LEU
4	D	472	LYS

Continued on next page...

Continued from previous page...

Mol	Chain	Res	Type
4	D	506	ASP
4	D	514	PHE
4	D	546	PHE
4	D	565	GLU
4	D	567	VAL
4	D	576	LYS
4	D	577	LYS
4	D	587	ILE
4	D	588	ASN
4	D	631	LYS
4	D	665	LEU
4	D	666	MET
4	D	683	GLU
4	D	715	THR
4	D	743	ILE
4	D	747	LEU
4	D	750	MET
4	D	756	ARG
4	D	793	LYS
4	D	816	THR
4	D	852	GLN
4	D	855	GLU
4	D	859	ASP

Some sidechains can be flipped to improve hydrogen bonding and reduce clashes. All (34) such sidechains are listed below:

Mol	Chain	Res	Type
4	D	22	ASN
4	D	67	ASN
4	D	86	ASN
4	D	104	GLN
4	D	135	GLN
4	D	169	GLN
4	D	184	GLN
4	D	211	HIS
4	D	321	ASN
4	D	339	ASN
4	D	406	ASN
4	D	419	ASN
4	D	433	ASN
4	D	435	GLN
4	D	466	ASN

Continued on next page...

Continued from previous page...

Mol	Chain	Res	Type
4	D	485	ASN
4	D	488	ASN
4	D	579	ASN
4	D	592	ASN
4	D	648	GLN
4	D	649	GLN
4	D	669	GLN
4	D	697	ASN
4	D	726	HIS
4	D	737	GLN
4	D	744	GLN
4	D	762	ASN
4	D	764	ASN
4	D	781	ASN
4	D	784	HIS
4	D	799	HIS
4	D	852	GLN
4	D	854	HIS
4	D	871	ASN

5.3.3 RNA ⓘ

Mol	Chain	Analysed	Backbone Outliers	Pucker Outliers
3	R	8/9 (88%)	0	0

There are no RNA backbone outliers to report.

There are no RNA pucker outliers to report.

5.4 Non-standard residues in protein, DNA, RNA chains ⓘ

There are no non-standard protein/DNA/RNA residues in this entry.

5.5 Carbohydrates ⓘ

There are no carbohydrates in this entry.

5.6 Ligand geometry ⓘ

Of 3 ligands modelled in this entry, 2 are monoatomic - leaving 1 for Mogul analysis.

In the following table, the Counts columns list the number of bonds (or angles) for which Mogul statistics could be retrieved, the number of bonds (or angles) that are observed in the model and the number of bonds (or angles) that are defined in the Chemical Component Dictionary. The Link column lists molecule types, if any, to which the group is linked. The Z score for a bond length (or angle) is the number of standard deviations the observed value is removed from the expected value. A bond length (or angle) with $|Z| > 2$ is considered an outlier worth inspection. RMSZ is the root-mean-square of all Z scores of the bond lengths (or angles).

Mol	Type	Chain	Res	Link	Bond lengths			Bond angles		
					Counts	RMSZ	$\#\|Z\| > 2$	Counts	RMSZ	$\#\|Z\| > 2$
5	APC	R	901	6	27,33,33	1.49	4 (14%)	31,52,52	2.12	7 (22%)

In the following table, the Chirals column lists the number of chiral outliers, the number of chiral centers analysed, the number of these observed in the model and the number defined in the Chemical Component Dictionary. Similar counts are reported in the Torsion and Rings columns. '-' means no outliers of that kind were identified.

Mol	Type	Chain	Res	Link	Chirals	Torsions	Rings
5	APC	R	901	6	-	3/15/38/38	0/3/3/3

All (4) bond length outliers are listed below:

Mol	Chain	Res	Type	Atoms	Z	Observed(Å)	Ideal(Å)
5	R	901	APC	PB-O3B	3.96	1.62	1.58
5	R	901	APC	PA-O2A	-3.24	1.48	1.56
5	R	901	APC	PB-O2B	-3.22	1.48	1.56
5	R	901	APC	O3'-C3'	-2.02	1.38	1.43

All (7) bond angle outliers are listed below:

Mol	Chain	Res	Type	Atoms	Z	Observed(^{o})	Ideal(^{o})
5	R	901	APC	C1'-N9-C4	7.72	140.21	126.64
5	R	901	APC	O1B-PB-C3A	-5.06	95.69	109.07
5	R	901	APC	O2A-PA-O1A	2.91	119.78	110.07
5	R	901	APC	O2B-PB-O1B	2.75	119.25	110.07
5	R	901	APC	O2B-PB-C3A	2.34	116.16	106.58
5	R	901	APC	O2G-PG-O1G	2.27	119.57	110.68
5	R	901	APC	C5-C6-N6	2.16	123.63	120.35

There are no chirality outliers.

All (3) torsion outliers are listed below:

Mol	Chain	Res	Type	Atoms
5	R	901	APC	PB-C3A-PA-O1A
5	R	901	APC	PB-C3A-PA-O5’
5	R	901	APC	PB-C3A-PA-O2A

There are no ring outliers.

1 monomer is involved in 11 short contacts:

Mol	Chain	Res	Type	Clashes	Symm-Clashes
5	R	901	APC	11	0

The following is a two-dimensional graphical depiction of Mogul quality analysis of bond lengths, bond angles, torsion angles, and ring geometry for all instances of the Ligand of Interest. In addition, ligands with molecular weight > 250 and outliers as shown on the validation Tables will also be included. For torsion angles, if less then 5% of the Mogul distribution of torsion angles is within 10 degrees of the torsion angle in question, then that torsion angle is considered an outlier. Any bond that is central to one or more torsion angles identified as an outlier by Mogul will be highlighted in the graph. For rings, the root-mean-square deviation (RMSD) between the ring in question and similar rings identified by Mogul is calculated over all ring torsion angles. If the average RMSD is greater than 60 degrees and the minimal RMSD between the ring in question and any Mogul-identified rings is also greater than 60 degrees, then that ring is considered an outlier. The outliers are highlighted in purple. The color gray indicates Mogul did not find sufficient equivalents in the CSD to analyse the geometry.

Ligand APC R 901

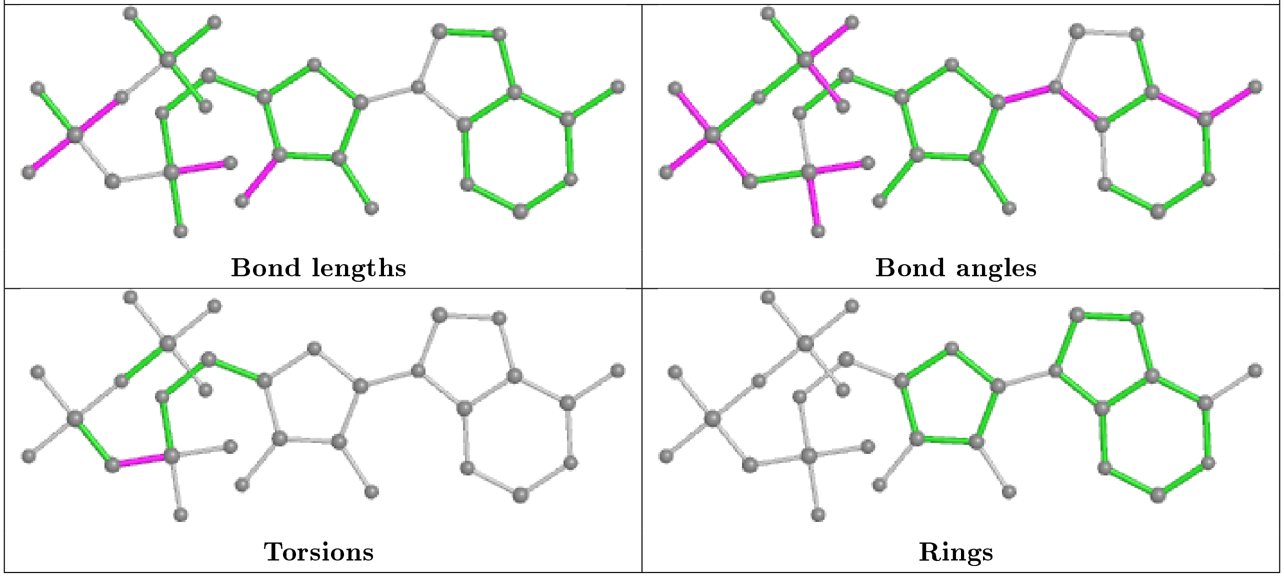

5.7 Other polymers (i)

There are no such residues in this entry.

5.8 Polymer linkage issues (i)

There are no chain breaks in this entry.

6 Fit of model and data (i)

6.1 Protein, DNA and RNA chains (i)

Unable to reproduce the depositors R factor - this section is therefore empty.

6.2 Non-standard residues in protein, DNA, RNA chains (i)

Unable to reproduce the depositors R factor - this section is therefore empty.

6.3 Carbohydrates (i)

Unable to reproduce the depositors R factor - this section is therefore empty.

6.4 Ligands (i)

Unable to reproduce the depositors R factor - this section is therefore empty.

The following is a graphical depiction of the model fit to experimental electron density of all instances of the Ligand of Interest. In addition, ligands with molecular weight > 250 and outliers as shown on the geometry validation Tables will also be included. Each fit is shown from different orientation to approximate a three-dimensional view.

Electron density around APC R 901:

$2mF_o$-DF_c (at 0.7 rmsd) in gray
mF_o-DF_c (at 3 rmsd) in purple (negative) and green (positive)

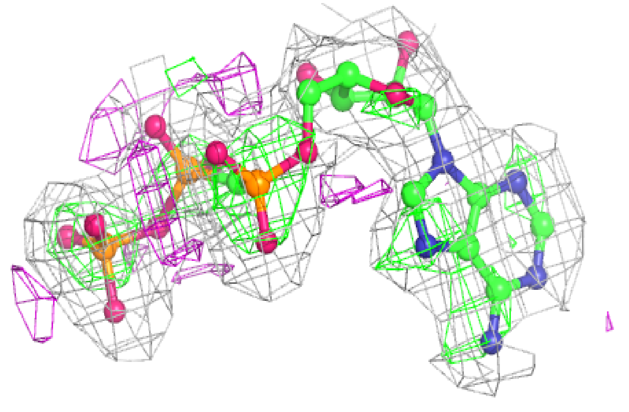

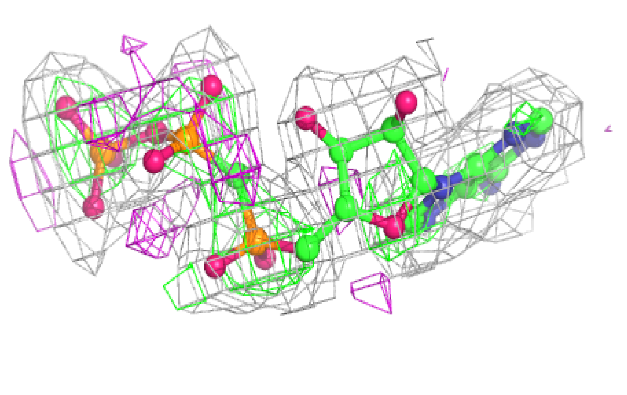

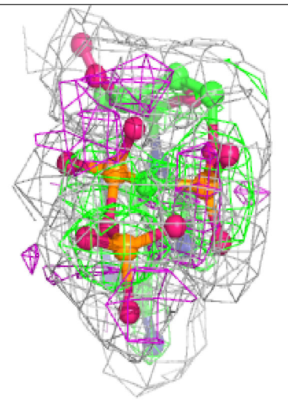

6.5 Other polymers (i)

Unable to reproduce the depositors R factor - this section is therefore empty.
